# Fetal growth restriction followed by early catch-up growth impairs pancreatic islet morphology in male rats

**DOI:** 10.1038/s41598-023-28584-2

**Published:** 2023-02-15

**Authors:** Mahboba Jabary, Atsuto Onoda, Yuma Kitase, Kazuto Ueda, Haruka Mimatsu, Shoji Go, Ryosuke Miura, Masahiro Tsuji, Yoshiyuki Takahashi, Masahiro Hayakawa, Yoshiaki Sato

**Affiliations:** 1 Division of Neonatology, Center for Maternal-Neonatal Care, Nagoya University Hospital, 65 Tsurumai-Cho Showa-Ku, Nagoya, 466-8550 Japan; 2 Department of Pediatrics, Nagoya University Graduate School of Medicine, Nagoya, Japan; 3 Department of Toxicology and Health Science, Faculty of Pharmaceutical Sciences, Sanyo-Onoda City University, Sanyoonoda, Japan; 4 Department of Food and Nutrition, Kyoto Women’s University, Kyoto, Japan

**Keywords:** Intrauterine growth, Neonatology, Paediatric research, Preterm birth

## Abstract

Fetal growth restriction (FGR), followed by postnatal early catch-up growth, is associated with an increased risk of metabolic dysfunction, including type 2 diabetes in humans. This study aims to determine the effects of FGR and early catch-up growth after birth on the pathogenesis of type 2 diabetes, with particular attention to glucose tolerance, pancreatic islet morphology, and fibrosis, and to elucidate its mechanism using proteomics analysis. The FGR rat model was made by inducing mild intrauterine hypoperfusion using ameroid constrictors (ACs). On day 17 of pregnancy, ACs were affixed to the uterine and ovarian arteries bilaterally, causing a 20.9% reduction in birth weight compared to sham pups. On postnatal day 4 (P4), the pups were assigned to either the good nutrition (GN) groups with 5 pups per dam to ensure postnatal catch-up growth or poor nutrition groups with 15 pups per dam to maintain lower body weight. After weaning, all pups were fed regular chow food ad libitum (P21). Rats in both FGR groups developed glucose intolerance; however, male rats in the FGR good nutrition (FGR-GN) group also developed hypertriglyceridemia and dysmorphic pancreatic islets with fibrosis. A comprehensive and functional analysis of proteins expressed in the pancreas showed that FGR, followed by early catch-up growth, severely aggravated cell adhesion-related protein expression in male offspring. Thus, FGR and early catch-up growth caused pancreatic islet morphological abnormalities and fibrosis associated with the disturbance of cell adhesion-related protein expressions. These changes likely induce glucose intolerance and dyslipidemia in male rats.

## Introduction

Fetal growth restriction (FGR) is a condition wherein a fetus does not reach its biological growth potential, mainly due to placental insufficiency^1–3^. FGR affects around 30 million newborns yearly^4^ and remains a major cause of perinatal mortality and morbidity^5^. It is associated with risks of adverse health outcomes in later life, including neurodevelopmental disorders^1,6^, cardiovascular disease, type 2 diabetes, and obesity^7,8^. The causes of FGR include maternal factors, such as undernutrition^9^ or overnutrition^10^, smoking^11^, emotional stress^12^, and diabetes^13^, and fetal factors, such as intrauterine infections^14^, multiple gestations^15,16^, and genetics^17^. In FGR, which is because of poor nutrition (PN), inadequate nutrient supplies during intrauterine life cause selective growth of major organs such as the brain at the expense of others like the liver and kidney as well as permanent changes in endocrine or metabolic settings, including tissue insulin sensitivity and pancreatic β-cell mass. Consequently, the fetus adapts to increase survival chance, however, predisposes to type 2 diabetes in later life^18–20^. In addition, postnatal nutrition significantly contributes to these abnormal developments and maladaptive changes, inducing chronic diseases such as type 2 diabetes in adulthood^21,22^. Most infants born with FGR demonstrate spontaneous catch-up growth within the first two years of life and exhibit rapid weight gain^23,24^. Catch-up growth is defined as the compensatory accelerated growth that occurs after a period of poor intrauterine growth to reach the reference growth of term-born infants. It is recognized when a child exhibits accelerated growth, crossing its centiles in length or weight growth^25,26^. In preterm infants, rapid postnatal weight gain has been associated with type 2 diabetes and metabolic syndrome in later life^7,26,27^. Furthermore, low birth weight is associated with elevated blood pressure in adult life^28,29^.

Several FGR animal models have been studied to explore the impact of FGR on postnatal development, including those involving protein restriction caused impaired islet development and functional alterations in pancreatic islets^30,31^, administration of synthetic glucocorticoids resulted in decreased insulin sensitivity^32^, and bilateral uterine artery ligation caused declines in insulin secretion, decreased pancreatic β-cell mass, and diabetes^33–35^. Islet fibrosis is commonly seen in people with type 2 diabetes^36^ and various rodent models of spontaneous type 2 diabetes, such as diabetic nonobese Goto-Kakizaki, Zucker diabetic fatty, spontaneously diabetic Torii rats, and diabetic mutant mouse^37^. All of these animal models exhibited pancreatic islet fibrosis with altered islet architecture. The spontaneous diabetic rat exhibited dysmorphic pancreatic islets and fibrosis associated with hyperglycemia, impaired glucose tolerance at 6 months, and islet lesions by 18 months^38^. Recently, pancreatic islet inflammation and fibrosis, hyperinsulinemia, dyslipidemia, and glucose intolerance were reported in FGR induced by bilateral uterine artery ligation and high-fat-diet rat models, in which the high-fat diet was introduced at five weeks of age (juvenile period)^39^. In addition, FGR followed by a postnatal fructose diet increased plasma total cholesterol, triglyceride, and high-density lipoprotein (HDL) cholesterol levels in female rats and triglyceride levels in male rats^40^. However, the FGR rat model induced by a low-protein diet (LPD) showed increased whole-body insulin sensitivity (47% increase in males and 38% increase in females) in the absence of postnatal catch-up growth; meanwhile, blood pressure remained within normal limits^41^.

Epidemiological studies have found that FGR due to placental insufficiency increases the risk of type 2 diabetes because of impaired β-cell development during the perinatal period. Importantly, β-cells help regulate glucose tolerance^42^. Infants with restricted growth who are also exposed to unfavorable nutritional environments may develop insulin resistance, dyslipidemia, high blood pressure, obesity, and type 2 diabetes^19^. Low weight at birth, followed by a period of catch-up growth in infants, has been strongly associated with increased risk of insulin resistance and type 2 diabetes^43–46^. Despite these findings, it is unknown how the nutritional environment after birth until weaning [Postnatal day 21 (P21)] and the early period of catch-up growth during the lactation period impact glucose and lipid metabolism, islet morphology and fibrosis, and blood pressure in FGR rats. In addition, the mechanisms that control these effects are unknown.

We hypothesized that FGR, followed by good postnatal nutrition, affects postnatal growth, blood glucose levels, serum lipid levels, and blood pressure; such effects might influence pathogenesis of type 2 diabetes.

We sought to determine the sex-specific effects of FGR and postnatal catch-up growth on the development of type 2 diabetes. We focused on islet morphology and fibrosis, the development of hypertension, and determining which mechanisms of pancreatic protein dysregulation were responsible for the FGR-induced morphological changes in the pancreatic islets, followed by a period of early, postnatal catch-up growth. We used our recently developed rat FGR model of chronic ischemia in utero since it more closely mimics placental insufficiency^47^.

## Results

### Body weight

Compared to sham-operated dams, the birth litter size of FGR-operated dams was reduced by 52% (*P* < 0.001) (6 ± 0.2 vs. 10 ± 0.8). At P1, the FGR groups had significantly lower average body weights than the sham groups (*P* < 0.001). By P4, four pups died in FGR group.

**Figure 1:**
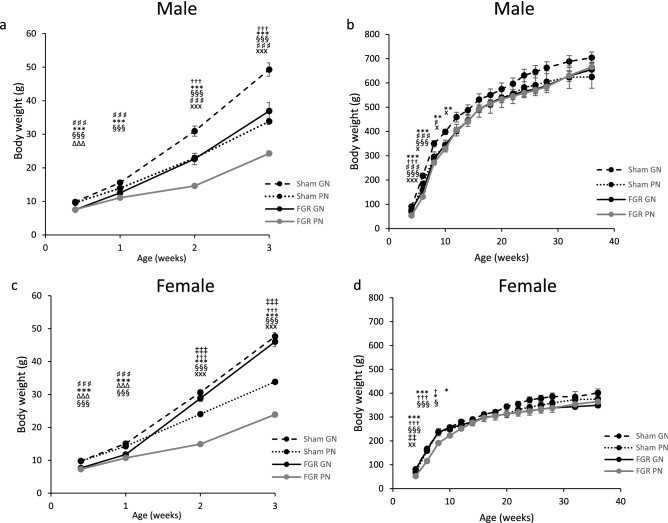
Effects of fetal growth restriction and rapid catch-up growth on body weight. (**a**) Average body weights of male pups from P4 until 3 weeks (weaning on P21), (**b**) average body weights of male offspring from 4 to 36 weeks. The FGR groups had significantly lower body weights than the sham groups *P* < 0.001. At 2 weeks, body weight was significantly higher in the FGR-GN group than in the FGR-PN group and in the sham-GN group than in the sham-PN group, *P* < 0.001. At 12 weeks, there was no differences among all groups. Number of male pups from P4 until weaning: sham-GN: n = 10 (2 groups of 5 pups), sham-PN: n = 7, FGR-PN: n = 8, and FGR-GN: n = 10 (2 groups of 5 pups). After weaning the number of GN pups reduced to unify with PN groups as shown below: Number of male offspring from 4 to 36 weeks: sham-GN: n = 7, sham-PN: n = 7, FGR-PN: n = 8, and FGR-GN: n = 7. (**c**) Average body weights of female pups from P4 until 3 weeks (weaning on P 21), (**d**) average body weights of female offspring from 4 to 36 weeks. At 2 weeks, body weight was significantly higher in the FGR-GN group than in the FGR-PN and sham-PN groups, *P* < 0.001. Also, the sham-GN group had a significantly higher body weight than the sham-PN group, *P* < 0.001. At 12 weeks, there was no differences among all groups. Number of female offspring from P4 until weaning (P21): sham-GN: n = 10 (2 groups of 5 pups), sham-PN: n = 8, FGR-PN: n = 7, and FGR-GN: n = 10 (2 groups of 5 pups). After weaning the number of GN pups reduced to unify with PN groups as shown below: Number of female offspring from 4–36 weeks: sham-GN: n = 8, sham- PN: n = 8, FGR-PN: n = 7, and FGR-GN: n = 8. FGR-GN vs. sham-PN: ‡*P* < 0.05, ‡‡*P* < 0.01, ‡‡‡*P* < 0.001; FGR-GN vs. FGR-PN: †*P* < 0.05, ††*P* < 0.01, †††*P* < 0.001; sham-GN vs. FGR-GN: ♯*P* < 0.05, ♯♯*P* < 0.01, ♯♯♯*P* < 0.001; sham-GN vs. FGR-PN: *P < 0.05, **P < 0.01, ***P < 0.001; sham-PN vs. FGR-GN: Δ*P* < 0.05, ΔΔ*P* < 0.01, ΔΔΔ*P* < 0.001; sham-PN vs. FGR-PN: §*P* < 0.05, §§*P* < 0.01, §§§*P* < 0.001, sham-GN vs. sham-PN: x*P* < 0.05, xx*P* < 0.01, xxx*P* < 0.001.

Among male pups, the body weights of the sham-GN and sham-PN groups were higher than those of the FGR-GN and FGR-PN groups at P4 (*P* < 0.001). However, there was no difference between the sham-PN and FGR-GN groups at 1 week old. At 2 weeks old, the FGR-GN group was significantly heavier than the FGR-PN group (*P* < 0.001). In addition, the sham-GN group was significantly heavier than the sham-PN group (*P* < 0.01) (Fig. 1a).

There were no between-group differences in body weight at 6 weeks for the FGR-GN and FGR-PN groups, at 8 weeks for the sham-PN and FGR-PN groups, and by 10 weeks for the sham-GN and FGR-GN groups. By 12 weeks, there were not statistically significant between-group differences for any of the groups (Fig. 1b). One male rat died at 12 weeks in the sham group.

Among the female pups, sham-GN and sham-PN group pups weighed more than FGR-GN and FGR-PN group pups at P4 (*P* < 0.001). At 2 weeks, the FGR-GN group had a higher average body weight than the FGR-PN or sham-PN groups (*P* < 0.001). The sham-GN group was significantly heavier than the sham-PN group (*P* < 0.001); however, there was no difference between the sham-GN and FGR-GN groups (Fig. 1c). By 6 weeks, the sham-GN and sham-PN groups and the FGR-GN and sham-PN groups had similar body weights. By 12 weeks, there were no between-group differences (Fig. 1d).

### Intraperitoneal glucose tolerance test (IPGTT)

**Figure 2:**
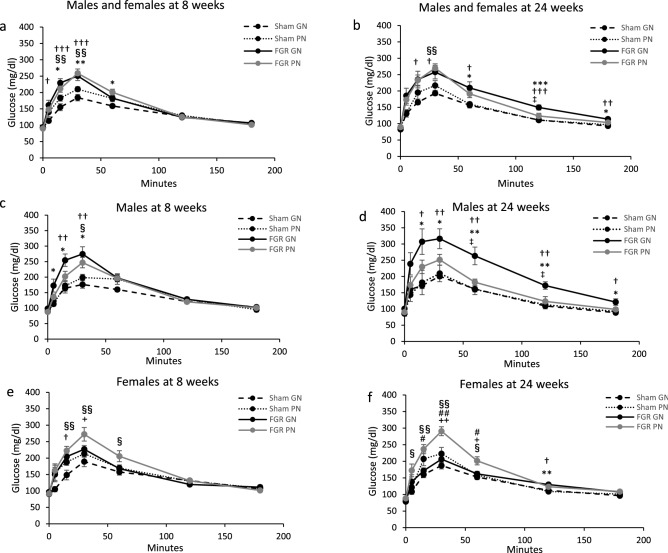
Intraperitoneal glucose tolerance test. (**a**) IPGTT males and females (not disaggregated) at 8 weeks. (**b**) IPGTT males and females (not disaggregated) at 24 weeks. (**c**) IPGTT males at 8 weeks. (**d**) IPGTT males at 24 weeks. (**e**) IPGTT females at 8 weeks. (**f**) IPGTT females at 24 weeks. Catch-up growth following FGR caused impaired glucose tolerance on the IPGTT after overnight fasting and glucose loading. FGR-GN vs. sham-PN: *P < 0.05, **P < 0.01; FGR-PN vs. sham-GN: §*P* < 0.05, §§*P* < 0.01; FGR-GN vs. sham-GN: †*P* < 0.05, ††*P* < 0.01, †††*P* < 0.001. FGR-GN vs. FGR-PN: ‡*P* < 0.05; FGR-PN vs. sham-PN: +*P* < 0.05, ++*P* < 0.01; FGR-PN vs. sham-PN: ♯*P* < 0.05, ♯♯*P* < 0.01. The number of male offspring: sham-GN: n = 7, sham-PN: n = 7, FGR-PN: n = 8, and FGR-GN: n = 7. Number of female offspring: sham-GN: n = 8, sham-PN: n = 8, FGR-PN: n = 7, and FGR-GN: n = 8. FGR: fetal growth restriction, IPGTT: intraperitoneal glucose tolerance test, GN: good nutrition, PN: poor nutrition.

We evaluated glucose tolerance by injecting glucose intraperitoneally at 8 and 24 weeks. When males and females were analyzed together, the glucose level in FGR groups were higher than in the sham groups at 8 and 24 weeks of age (Fig. 2a and b). However, when males and females were analyzed separately, glucose tolerance differed between males and females.

At 8 weeks, blood glucose levels were higher in male FGR-GN group rats compared to male sham-PN group (*P* < 0.05, at 5, 15, 30 min) and sham-GN group rats (*P* < 0.01, at 15, 30 min). The blood glucose levels were higher in the FGR-PN group than in the sham-GN group (*P* < 0.05, at 30 min) (Fig. 2c). At 24 weeks, blood glucose levels were higher in the FGR-GN group than in the sham-GN, sham-PN (*P* < 0.01, at 60, 120 min), or FGR-PN groups (*P* < 0.05, at 60, 120 min) (Fig. 2d).

On the other hand, among 8-week-old female rats, blood glucose levels were higher in the FGR-PN than in the sham-GN (*P* < 0.01, at 15, 30 min) or the sham-PN groups (*P* < 0.05, at 30 min). The FGR-GN group had a higher average blood glucose level than the sham-GN group (*P* < 0.05, at 15 min) (Fig. 2e). At 24 weeks, the blood glucose levels were higher in the FGR-PN than in the sham-GN group (*P* < 0.01, at 15, 30 min). The FGR-PN group had higher blood glucose than the sham-PN group (*P* < 0.01, at 30 min) and the FGR-GN group (*P* < 0.01, at 30 min) (Fig. 2f).

### Pancreatic insulin-positive and total area

**Figure 3:**
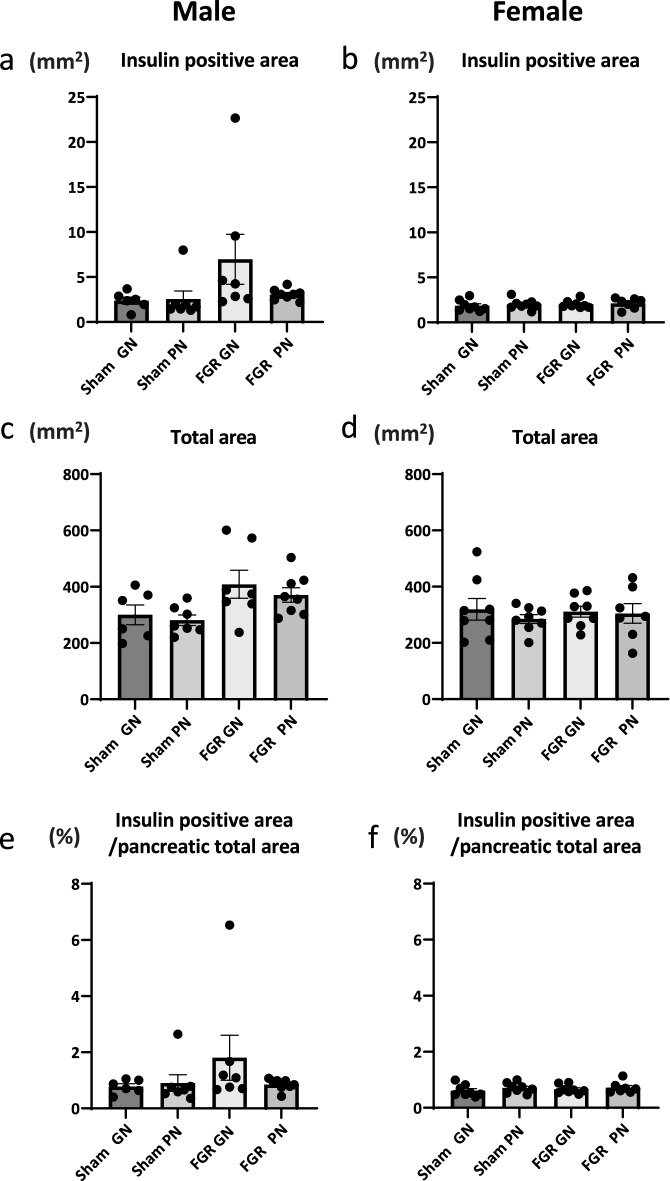
Effects of fetal growth restriction and early catch-up growth on the insulin-positive area. In both male and female rats, there were no significant differences in insulin-positive areas (**a**, **b**), pancreatic total area (**c**, **d**), and the ratio (insulin-positive area to pancreatic total area) (**e**, **f**) among the four groups. The number of male offspring: sham-GN: n = 6, sham-PN: n = 7, FGR-PN: n = 8, and FGR-GN: n = 7. Number of female offspring: sham-GN: n = 8, sham-PN: n = 8, FGR-PN: n = 7, and FGR-GN: n = 8. FGR: fetal growth restriction, GN: good nutrition.

We evaluated insulin-positive and total pancreatic areas using immunohistochemistry at 36 weeks in male and female rats. There were no between-group differences for the insulin-positive area, total pancreatic area, or in the ratio of insulin-positive to total pancreatic areas (Fig. 3).

### Islet morphology and fibrosis

**Figure 4:**
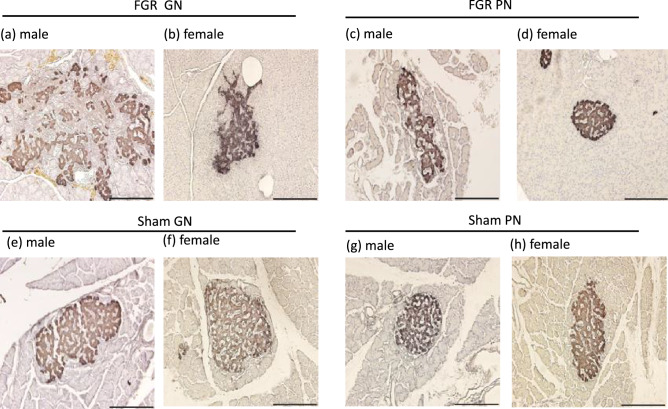
Representative photographs of pancreatic islets from 36-week-old male and female rats immunostained with an anti-insulin antibody and counterstained with hematoxylin and eosin at an original magnification of 400 × . (**a**) Islets of male FGR-GN rats exhibited a dysmorphic structure, large size, and scattered islet tissues. (**b**) Islets of female FGR-GN rats. (**c**) Islets of male FGR-PN rats showed slight dysmorphisms. The islets appeared normal in female FGR-PN rats (**d**) and in male and female sham-GN and sham-PN rats (**e**–**h**). Scale bars indicate 200 µm. The number of male offspring: sham-GN: n = 6, sham-PN: n = 7, FGR-PN: n = 8, and FGR-GN: n = 7. Number of female offspring: sham-GN: n = 7, sham-PN: n = 8, FGR-PN: n = 7, and FGR-GN: n = 8. FGR: fetal growth restriction, GN: good nutrition, PN: poor nutrition.

**Figure 5:**
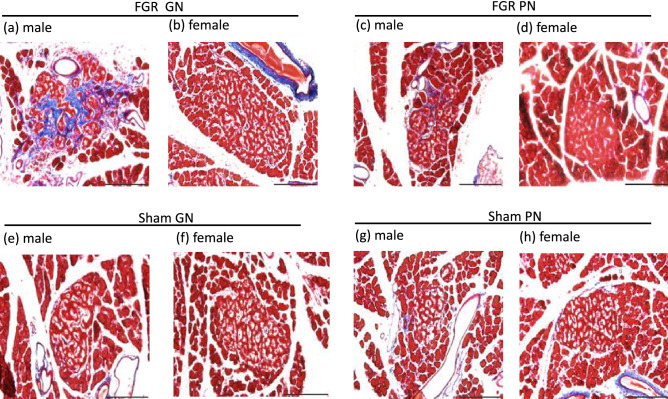
Representative photographs of pancreatic islets from 36-week-old male and female rats, Masson’s trichrome staining for fibrosis at an original magnification of 400 × . Fibrosis was evident in the islets of male FGR-GN rats (**a**). Fibrosis was not observed in the islets of other groups (**b**–**h**). Scale bars indicate 200 µm. The number of male offspring: sham-GN: n = 6, sham-PN: n = 7, FGR-PN: n = 8, and FGR-GN: n = 7. Number of female offspring: sham-GN: n = 7, sham-PN: n = 8, FGR-PN: n = 7, and FGR-GN: n = 8. FGR: fetal growth restriction, GN: good nutrition.

Severely dysmorphic (i.e., large size, scattered tissue, and irregular structure) islets of Langerhans were evident in male FGR-GN rats. In contrast, slightly dysmorphic islets were observed in female FGR-GN and male FGR-PN rats (Fig. 4a–c). The islets were structurally normal in female FGR-PN rats and in all sham groups for both sexes (Fig. 4d–h). Markedly fibrotic tissue was evident in male FGR-GN rats (Fig. 5a); however, no signs of fibrosis were observed in the sham groups of either sex: male FGR-PN rats and female FGR-GN and PN rats (Fig. 5b–h).

### Blood biochemistry

**Figure 6:**
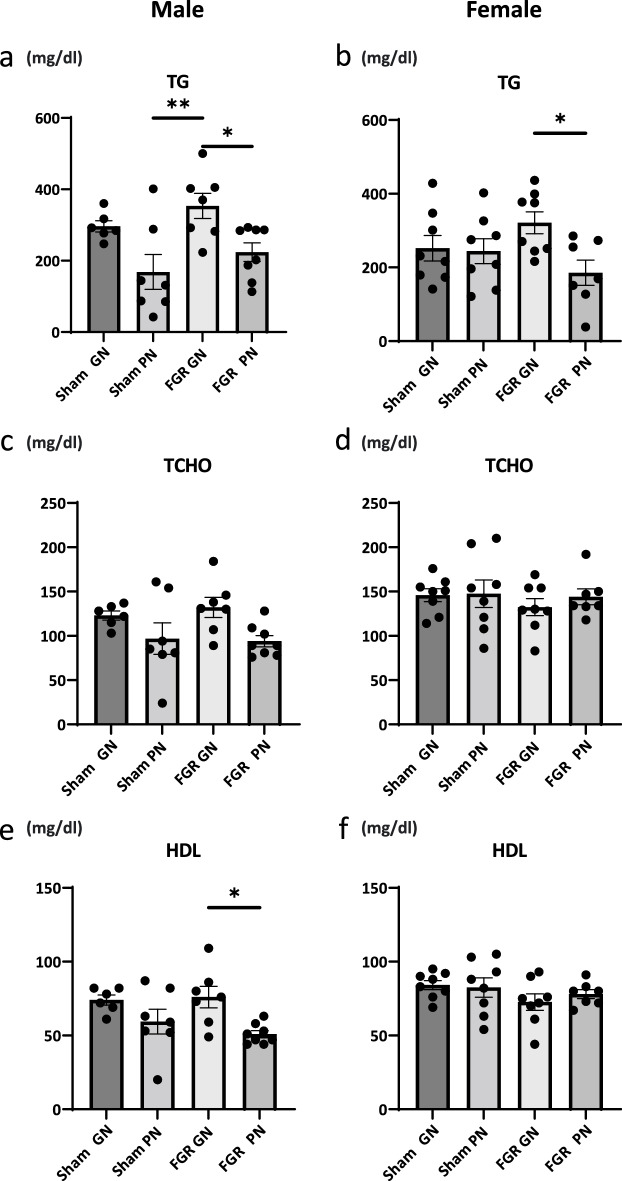
Effects of FGR and early catch-up growth on serum lipid parameters. (**a**) In male rats, the FGR-GN group had a higher TG level than the sham-PN and FGR-PN groups. (**b**) In female rats, the FGR-GN group had a higher TG level than the FGR-PN group. (**e**) The average HDL level was higher in the FGR-GN group than in the FGR-PN group. TCHO was similar across all four groups and for both male and female rats (**c**, **d**). *P < 0.05, **P < 0.01. The number of male offspring: sham-GN: n = 6, sham-PN: n = 7, FGR-PN: n = 8, and FGR-GN: n = 7. Number of female offspring: sham-GN: n = 8, sham-PN: n = 8, FGR-PN: n = 7, and FGR-GN: n = 8. FGR: fetal growth restriction, GN: good nutrition, TG: triglyceride, PN: poor nutrition, TCHO: total cholesterol, HDL: high-density lipoprotein cholesterol.

Serum parameters related to dyslipidemia [e.g., triglycerides (TG), total cholesterol (TCHO), and HDL cholesterol] were evaluated at 36 weeks. In male rats, FGR-GN rats had a higher average TG level than their sham-PN (*P* < 0.01) and FGR-PN (*P* < 0.05) same-sex equivalents (Fig. 6a). HDL levels were higher in FGR-GN than in FGR-PN rats (Fig. 6e, *P* < 0.05). There were no significant between-group differences in TCHO levels (Fig. 6c). Among female rats, average TG levels were higher for FGR-GN than for FGR-PN same-sex equivalents (*P* < 0.05, Fig. 6b). There were no significant between-group differences for TCHO or HDL (Fig. 6d, f).

### Proteomics

**Figure 7:**
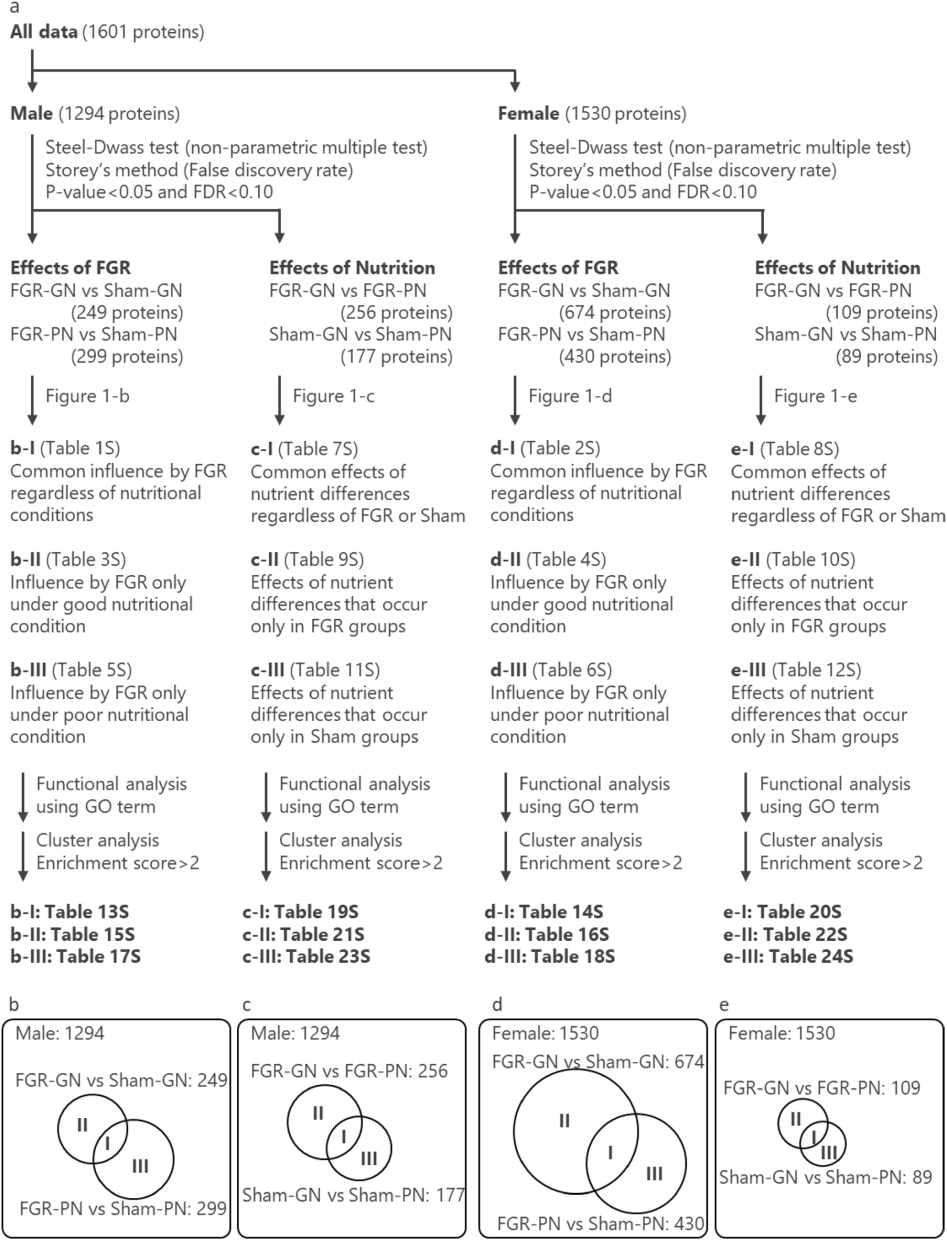
Visualization of the analytical process of proteomics. (**a**) Flowchart of the analytical process and extraction criteria for the pancreatic proteins that were abnormally expressed in each group. (**b**–**e**) Venn diagram of the number of extracted proteins. (**b**, **c**) Results of the male rats. (**d**, **e**) Results of the female rats. (**b**, **d**) Results of the comparison between the FGR and sham groups. (**c**, **e**) Results of the comparison between the GN and PN groups. FGR: fetal growth restriction, GN: good nutrition, PN: poor nutrition, GO: gene ontology.

**Figure 8:**
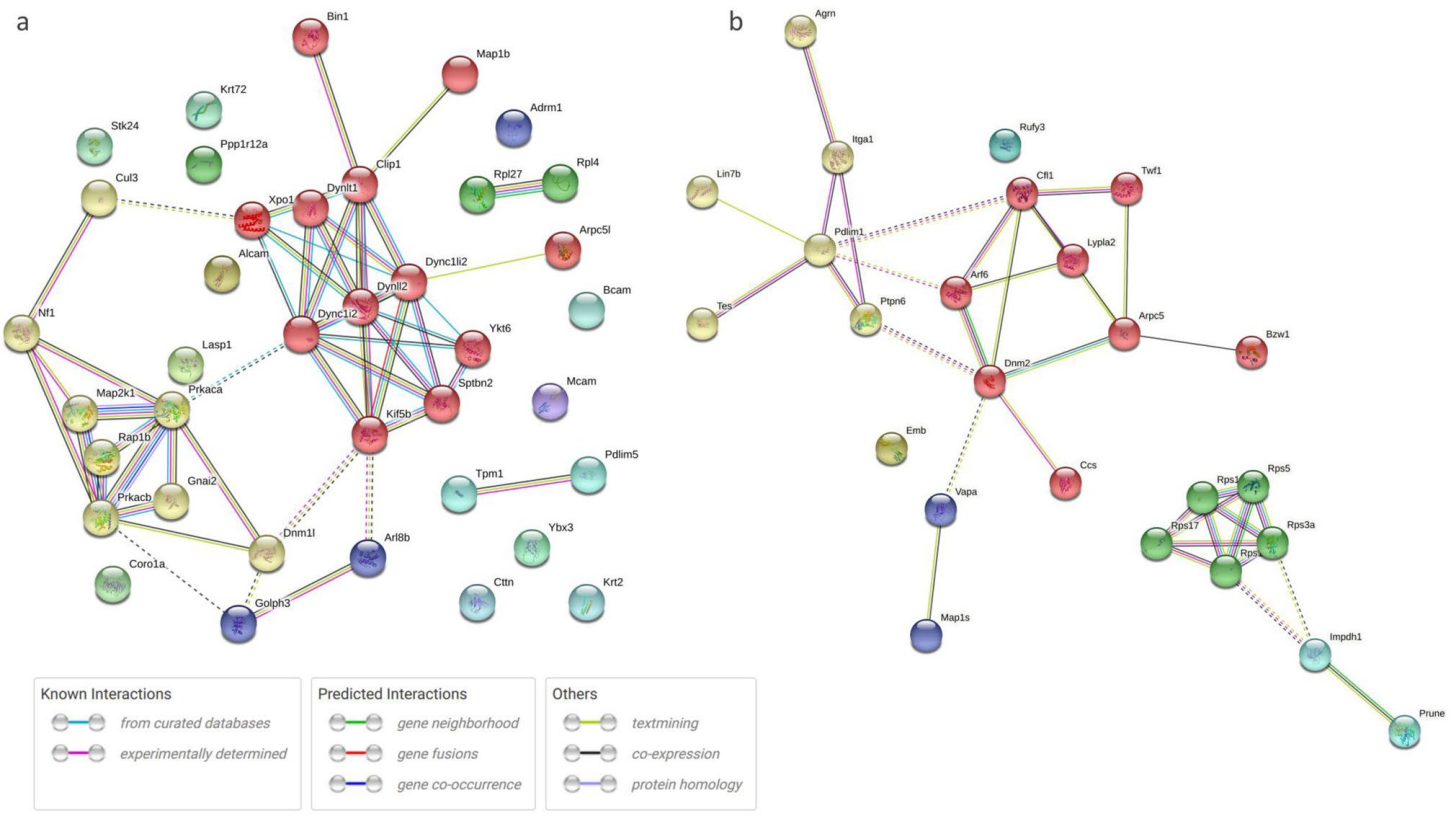
Interaction networks of extracted proteins. (**a**) Interaction of proteins categorized into the microtubule/cytoskeleton-related and cell adhesion-related clusters, as observed in the annotation analysis of the b-I profile (proteins affected by FGR regardless of nutritional conditions). (**b**) Interaction of proteins categorized into the cell adhesion-related cluster, as observed in the annotation analysis of the b-II profile (proteins affected by FGR-GN only). The figures were generated using the Search Tool for the Retrieval of Interacting Genes/Proteins 11.0 (STRING 11.0), (https://string-db.org/) with MCL clustering (inflation parameter: 3). The minimum required interaction scores were set at (**a**) 0.400 and (**b**) 0.150. An interaction was indicated according to eight criteria: neighborhood, gene fusion, co-occurrence, co-expression, experiments, databases, text mining, and homology. Solid lines: specific and meaningful associations like shared function in the MCL cluster. Dashed lines: edges between MCL clusters. FGR: fetal growth restriction, GN: good nutrition, Markov cluster algorithm (MCL), PN: poor nutrition.

The proteins extracted from the pancreas lysates of 36-week-old rats were subjected to proteomics and annotation analysis to reveal target molecules associated with mechanisms of the histopathological abnormalities and to comprehensively understand the alteration of protein expression in the pancreas. We comprehensively investigated differences in the protein profiles among the four groups (FGR-GN, FGR-PN, sham-GN, and sham-PN) of each sex. LC/MS/MS detected 1601 proteins with high-quality signals and quantified the protein content within the lysate. Of the 1601 proteins, 1294 and 1530 were detected in pancreatic tissue obtained from male and female offspring, respectively (Fig. 7a). Of the 1294 proteins, 249 significantly differed between the FGR-GN and sham-GN groups (Fig. 7b-II). Likewise, 299 of the 1294 proteins exhibited significant differences between the FGR-PN and sham-PN groups (Fig. 7b-III). There were 94 common proteins between these 249 and 299 proteins (Fig. 7b-I). Using the same profiles with pancreatic tissue samples obtained from male rats, 256 proteins were significantly different between the FGR-GN and FGR-PN groups (Fig. 7c-II), and 177 proteins were significantly different between the sham-GN and sham-PN groups (Fig. 7c-III). There were 67 common proteins between these 256 and 177 proteins (Fig. 7c-I). Regarding the protein profiles of female pancreas samples, of 1530 proteins in total, 674, 430, 109, and 89 proteins were significantly different between the FGR-GN and sham-GN groups (Fig. 7d-II), FGR-PN and sham-PN groups (Fig. 7d-III), FGR-GN and FGR-PN groups (Fig. 7e-II), and sham-GN and sham-PN groups (Fig. 7e-III), respectively. There were 254 common proteins from these 674 and 430 proteins (Fig. 7d-I) and 8 common proteins from the 109 and 89 proteins (Fig. 7e-I).

The proteins classified into each group based on these comparative analyses have the following characteristics. Proteins classified as b-I and d-I were affected by FGR regardless of nutritional condition (Tables S1 and S2). Proteins classified as b-II and d-II were affected by FGR only under good nutritional conditions (Tables S3 and S4), whereas those classified as b-III and d-III were affected by FGR under poor nutritional conditions (Tables S5 and S6). Similarly, proteins classified as c-I and e-I were affected by the differences in the nutritional conditions regardless of being in the FGR or sham group (Tables S7 and S8). Proteins classified as c-II, c-III, e-II, and e-III were also affected by the differences in nutritional conditions. The proteins in c-II and e-II were observed only in FGR offspring (Tables S9 and S10), whereas the proteins in c-III and e-III were observed only in sham offspring (Tables S11 and S12).

### Functional analysis

The extracted protein profiles (Tables S1–S12) were processed for functional analysis using the gene ontology (GO) term database to understand the biological significance of the proteome results. The results of the functional annotation analysis are detailed in the supplementary tables (Tables S13–S24). The annotation analysis and clustering of the annotation have shown that the proteins categorized into the extracellular-related cluster (including ontologies associated with the extracellular exosome, extracellular vesicle, extracellular organelle, and extracellular region) were commonly enriched as the top of each profile (Tables S13–S19 and S21–S24), except for e-I (Tables S20). However, the second and subsequent clusters differed by profile. Notably, b-I, b-II, and c-II proteins were categorized into the cell adhesion-related cluster (including ontologies associated with the adherens junction, anchoring junction, focal adhesion, cell-substrate adherens junction, cell-substrate junction, and cell junction). They were ranked 3, 2, and 3, respectively (Tables S13, S15, and S21). Moreover, the b-I proteins are categorized into the microtubule/cytoskeleton-related cluster (including ontologies associated with the supramolecular fiber, polymeric cytoskeletal fiber, microtubule, microtubule cytoskeleton, microtubule-based process, microtubule organizing center, and centrosome) and were rank 2 (Table S13). These categories were not observed in the b-III and c-III profiles (Tables S17 and S23). In addition, the rank of these clusters was very low in the female profiles, although the clusters were observed in the profiles of d-I and d-II (Tables S14 and S16). For example, the cell adhesion-related cluster was ranked 36 in the d-I profile (Table S14). A microtubule/cytoskeleton-related cluster was detected at cluster rank 3 only in the e-II profile (Table S22).

**Figure 9:**
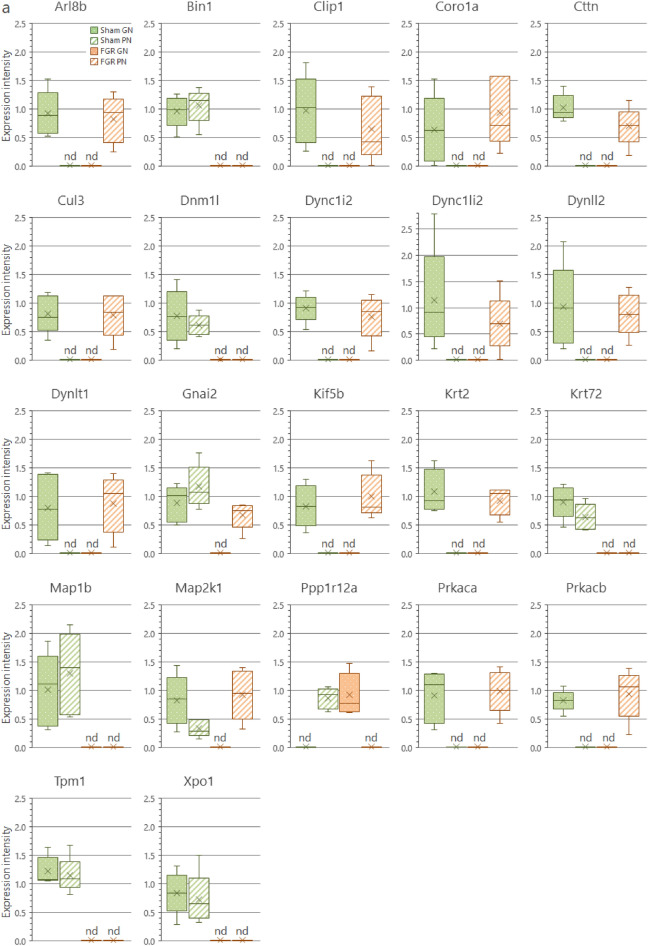

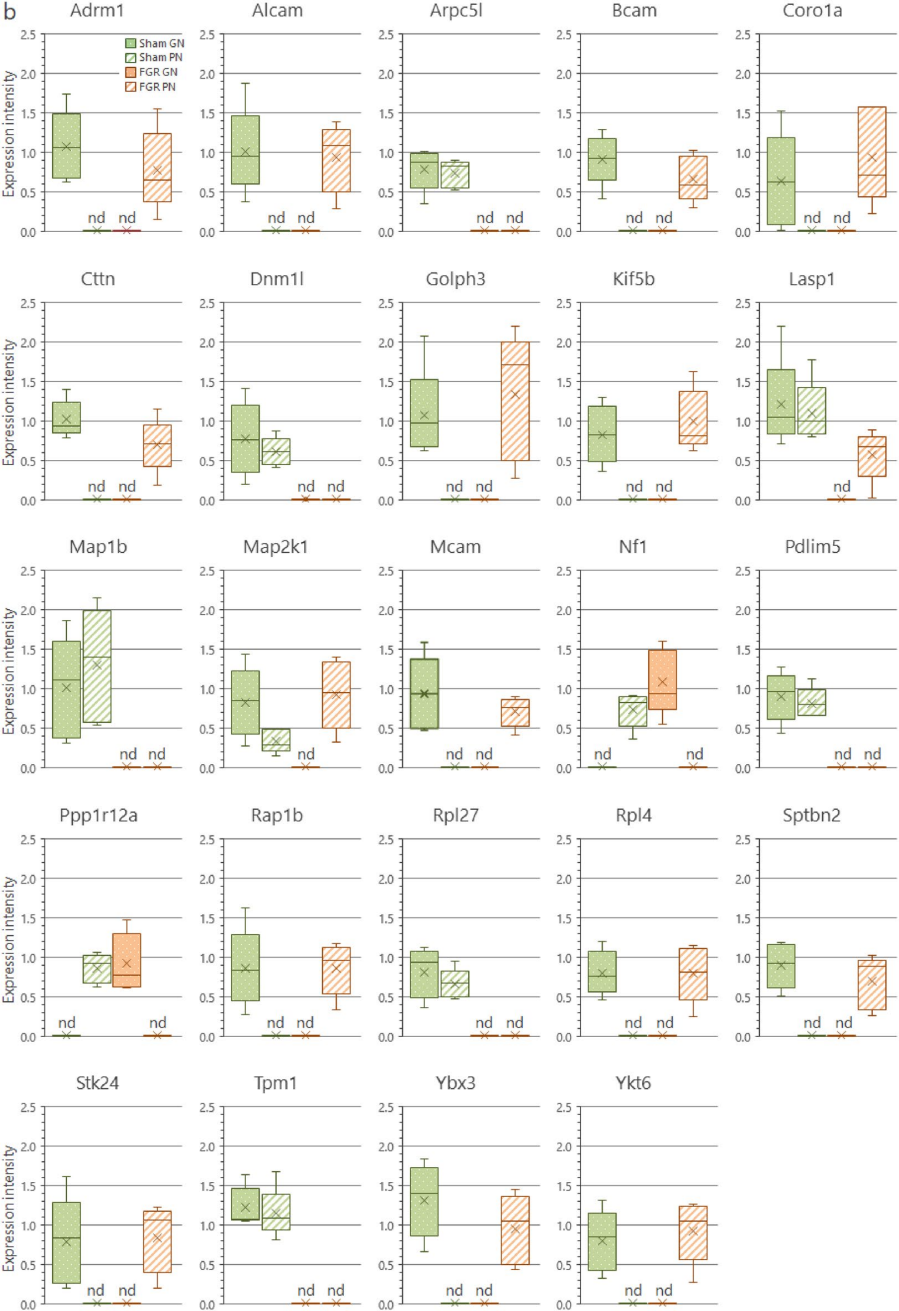

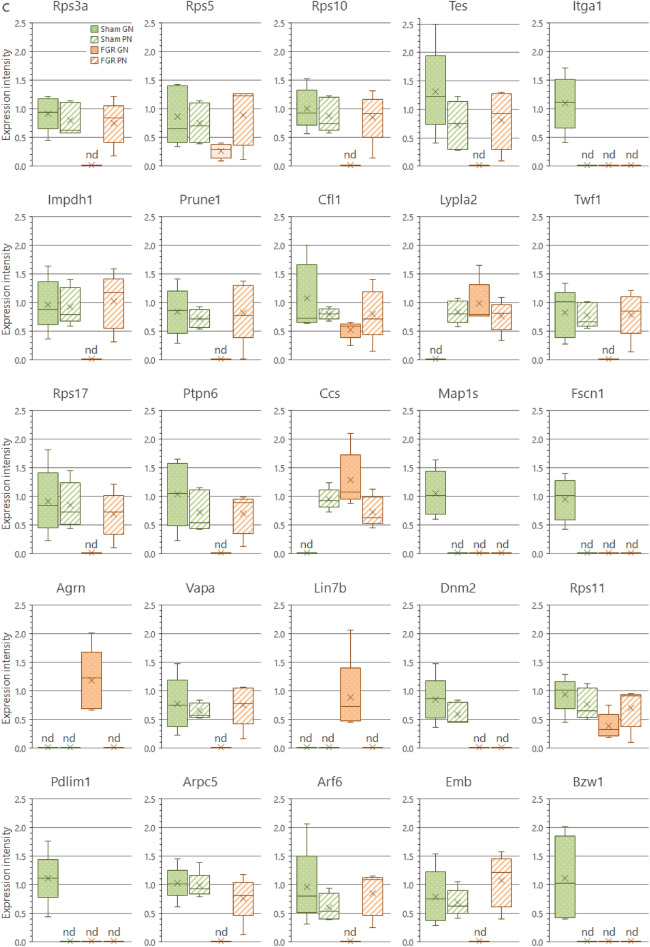
Expression levels of each protein in the clusters found during the functional analysis. (**a**) Expression levels of microtubule/cytoskeleton-related proteins affected by FGR regardless of nutritional conditions (Cluster rank 2 in the b-I profile). (**b**) Expression levels of cell adhesion-related proteins affected by FGR regardless of nutritional conditions (Cluster rank 3 in the b-I profile). (**c**) Expression levels of cell adhesion-related proteins affected by FGR only under good nutritional conditions (Cluster rank 2 in the b-II profile). Expression intensity is the relative expression levels to the average value of all proteins detected by liquid chromatography/tandem mass spectrometry (LC/MS/MS) as 1. The detection limit of the LC/MS/MS in terms of relative expression intensity is 0.013. Each box plot means the following groups: Green filled (Left); sham-GN group, Green stripe (Left in the middle); sham-PN group, Orange filled (Right in the middle); FGR-GN group, Orange stripe (Right); FGR-PN group. *Abbreviations* FGR: fetal growth restriction, GN: good nutrition, nd: not detected, PN: poor nutrition.

We analyzed the protein interaction networks using functional annotation analysis to enhance our understanding of their functional associations (Fig. 8). This network features molecular interactions among proteins in the microtubule/cytoskeleton-related and cell adhesion-related clusters, as shown in the annotation analysis of the b-I profile of male offspring (Fig. 8a). Similarly, molecular interactions among proteins in the cell adhesion-related cluster are shown in the annotation analysis of the b-II profile from male offspring (Fig. 8b). The figures depict the close relationships of some proteins (e.g., Prkaca, Map2k1, Rap1b, Prkacb, and Gnai2) (Fig. 8a, yellow nodes). These proteins categorized into similar ontologies can be further grouped. For example, Prkaca (Fig. 8a, yellow node) and Dync1i2 (Fig. 8a, red node) were categorized into different groups by STRING and MCL clustering. Furthermore, expression levels of these proteins are presented in Fig. 9; additional information (i.e., distribution in the pancreas, relative expression levels, and main function) is presented in Tables 1 and 2.

**Table 1:**
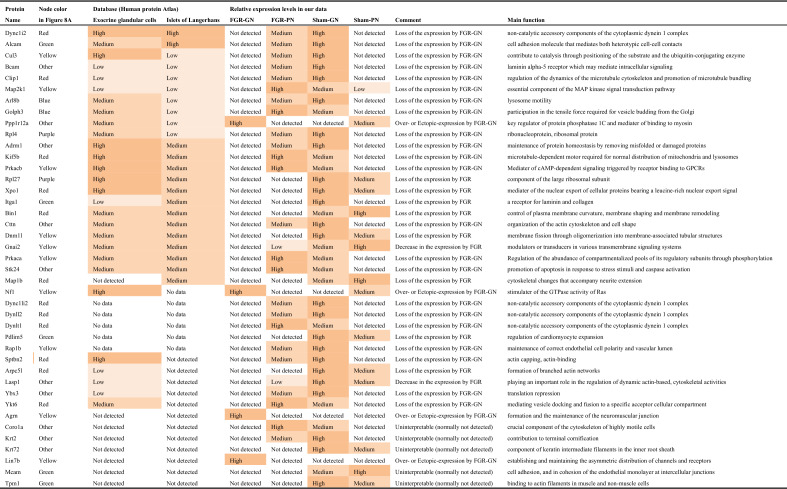
Proteins categorized into the microtubule/cytoskeleton-related and adhesion-related clusters in the b-I profile (influenced by FGR, regardless of nutritional conditions).

**Table 2:**
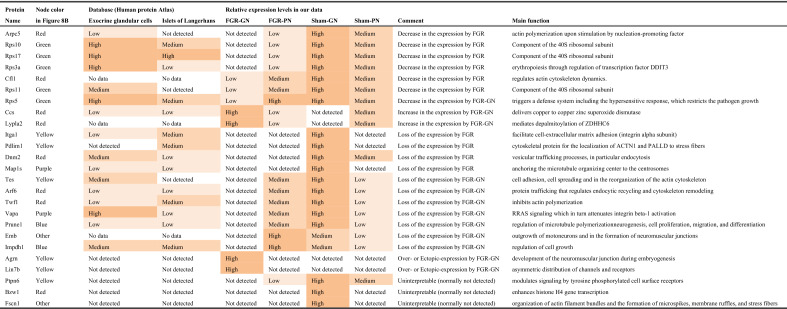
Proteins categorized into the cell adhesion-related cluster in the b-II profile (influenced by FGR only under good nutritional condition).

### Blood pressure

Blood pressure was measured at 8, 16, 24, and 35 weeks. Heart rate, systolic, diastolic, and mean blood pressures were similar across groups (Table S25).

## Discussion

We evaluated the effects of FGR, followed by an early catch-up growth period, on islet morphology and metabolic functions using the novel FGR model in male and female rats. FGR led to the development of glucose intolerance. Male FGR-GN rats demonstrated dysmorphic and fibrotic pancreatic islets and dyslipidemia. A comprehensive and functional analysis of proteins expressed in the pancreas showed that FGR and nutrition status altered extracellular-related protein expression. Male, but not female, FGR rats had dysregulated expression of proteins related to the microtubule/cytoskeleton and cell adhesion. In FGR rats, having GN severely aggravated the expression of proteins associated with cell adhesion.

FGR, followed by GN immediately after birth until weaning, caused dysmorphic and fibrotic islets, leading to glucose intolerance and dyslipidemia in male offspring. Previous studies reported abnormal glucose tolerance in malnourished rat models, accompanied by insulin resistance and reduced islet size/number^34,48^. In humans, poor prenatal nutrition and rapid postnatal growth are associated with glucose intolerance, type 2 diabetes, and obesity in adulthood^49–51^. In recent years, male sex has been considered a risk factor for type 2 diabetes. This is owing to a larger amount of visceral fat in men, which is a risk factor for glucose intolerance, insulin resistance, hyperinsulinemia, and type 2 diabetes^52–55^. Studies show that gender is an independent risk factor for the components of metabolic syndrome (MetS). The prevalence of MetS components reported in men and women as impaired glucose tolerance 18.1% and 7.9%, hypertension 5.3% and 3%, hypertriglyceridemia 17.7% and 9.4%, high HDL level 13.6% and 17% and type 2 diabetes 2.4% and 1.3% respectively^56^ The incidence of type 2 diabetes is reported 7.9% in men and 5.4% in women by the cohort study in Finland who were born low birth weight and demonstrated accelerated growth during childhood^50^. Another cohort study reported the higher prevalence of type 2 diabetes in men 14.6% vs. women 9.1%^55^. Low birth weight is associated with obesity and type 2 diabetes in adult life. Obesity is known as an independent risk factor for type 2 diabetes^8^. Experimental studies suggest that the MetS induced by a high sucrose diet causes severe metabolic changes in males compared with female rats^57^. Female rats are partly protected against type 2 diabetes due to the presence of estrogens^58^. Epidemiological studies suggest that low birth weight and accelerated postnatal growth are associated with elevated blood pressure in later life^28,59–61^. An association between FGR and high blood pressure (BP) has also been reported in Sprague–Dawley (SD) rats. Low birth weight due to placental insufficiency predisposes offspring to hypertension in adulthood^62^. Animal models of fetal programming for cardiovascular adult disease demonstrate that sex hormones modulate the activity of regulatory systems resulting in a lower incidence of cardiovascular dysfunctions and hypertension in females than in males^63^. However, our FGR rat model had no hypertension, consistent with previously published data^41^.

Furthermore, islet inflammation and fibrosis have been demonstrated in human type 2 diabetes^64^ and its rodent models^37,39^. A study demonstrated using a rat model that embryo/fetus was susceptible to intrauterine changes, including islet development, caused by nutritional alterations^65^. The pancreas continues to develop after birth through complex cell replication and neogenesis processes. This replication is high during the prenatal period and gradually decreases within the first month of postnatal life. A stable β-cell mass is maintained through replication, apoptosis, and neogenesis^66^. Past rat studies found that nutritional alterations during gestation were associated with divergent islet development in the fetus. This leads to decreased β-cells, less insulin secretion, and accelerated islet aging^65^. Even after birth, nutritional alterations can dramatically change islet morphology, potentially leading to pancreatic dysfunction that persists into adulthood. To prevent pancreatic dysfunction in adulthood, it may be important to avoid early catch-up of weight after birth.

To determine the molecular mechanisms underlying abnormal morphological changes and identify their key molecules, we performed a comprehensive and functional analysis of protein expression profiles in the pancreas. Our results indicated that FGR and nutritional alterations dysregulated extracellular-related proteins. Because the pancreas is the representative secretory organ, extracellular-related proteins may be vulnerable to these changes. Changes in extracellular-related protein expression might relate to glucose metabolism abnormalities, as demonstrated by the IPGTT test (Fig. 2). Moreover, FGR disrupted the expression levels of microtubule/cytoskeleton-related and cell adhesion-related protein clusters, generally responsible for regulating pancreatic tissue morphogenesis, in the pancreas of male offspring (Fig. 8a and Table 1). Notably, abnormal expressions of proteins related to the microtubule/cytoskeleton and cell adhesion were more pronounced in males than in females, who exhibited milder morphological changes. Thus, microtubule/cytoskeleton-related and cell adhesion-related proteins were associated with the morphological abnormalities observed in pancreatic tissue obtained from male FGR offspring. Furthermore, some proteins related to cell adhesion were only abnormally expressed by FGR under GN, but not under PN, conditions (Fig. 8b and Table 2). In other words, the GN condition worsened the abnormal expression of cell adhesion-related proteins induced by FGR. The histopathological analysis revealed that the most severe morphological abnormalities of the pancreatic islets of Langerhans were observed in male FGR-GN offspring, followed by male FGR-PN offspring (Fig. 4a, c). Therefore, the abnormal expression of the cell adhesion-related proteins in male FGR-GN rats might aggravate existing morphological abnormalities observed in this group. In summary, disrupting protein expression patterns related to the microtubule/cytoskeleton and cell adhesion might reveal the molecular mechanisms underlying the abnormal islet morphology in male FGR rats. Future studies will likely determine how its aggravation is induced by GN. Moreover, additional studies are needed to understand the roles of the microtubule/cytoskeleton and cell adhesion proteins determined in the comprehensive functional analysis of this study (Fig. 8 and Tables 1, 2) and their roles in pancreatic development and morphogenesis. The information will contribute to a better understanding of pediatric diabetes pathology at the molecular level, which will lead to establishing preventive and therapeutic strategies for this disease. In particular, the differences in the results of each group obtained by proteomics suggest that the type of pancreatic injury may be estimated by evaluating molecular expression patterns. Verification using human data is needed in further studies to achieve this.

We also compared changes in protein expression levels depending on nutritional conditions in female offspring. In the e-II profile, the proteins were categorized according to their relations to molecular binding, cytoskeletal fibers (microtubules and fibers), catabolism, and proteolysis (Table 22S). Proteins in the e-I and e-III profiles had no characteristic ontology and no cluster (Tables 20S and 24S). We found that nutrition had greater effects on protein expression in pancreatic tissue samples obtained from female offspring in the FGR group, compared to the sham group. Thus, proteins related to biomolecular binding and catabolism in FGR offspring appear sensitive to nutrition-related factors. The abnormal protein expression may be associated with the histopathological denaturation of the islets of Langerhans in the pancreas and the poor IPGTT results observed in female FGR-PN offspring. Future, focused studies of these protein groups will elucidate the molecular mechanisms that adversely affect the development of FGR female offspring in a PN environment.

No evidence indicates that the expression of cell adhesion-related and microtubule/cytoskeleton-related molecules in the pancreas differs depending on sex. However, since sex affects the expression of cell adhesion and cytoskeletal molecules in other organs, it may affect the expression of these molecules in the pancreas^66,67^. Thus, the effects of FGR and nutritional conditions on the expression of these molecules are also likely to differ. Hence, different morphological abnormalities of the pancreas may have been observed in males and females. Further investigation of sex differences in the expression of cell adhesion- and microtubule/cytoskeleton-related molecules in the pancreas is important for a clearer understanding of the morphological changes observed in this study.

Our results confirmed the existence of a critical period for nutrition intervention in FGR rats. Although introducing normal chow after weaning did not normalize FGR-PN rats’ glucose tolerance, the group’s islet morphology was unaffected. In contrast, islet morphology was affected in the FGR-GN group. Previous studies focused on the effects of FGR and high-fat diets on glucose metabolism and the pathogenesis of type 2 diabetes^39^. In contrast, we focused on the duration of malnutrition in the FGR-PN group and the effects of FGR, followed by GN immediately after birth. Exposure to a GN environment immediately after birth increased body weight, glucose intolerance, dysmorphic and fibrotic islets, and dysregulated pancreatic proteins. In contrast, PN immediately after birth lowered body weight but did not normalize glucose tolerance (or affect islet morphology). In low-birth-weight humans, catch-up growth mostly occurs within the first two years of life^67^. These results affirm a critical period for catch-up growth in FGR, similar to the suckling period in humans^23^.

This study has some limitations. First, we could not confirm high BP in our FGR and catch-up growth rats, while FGR rats induced by reduced uterine perfusion developed elevated arterial BP by eight weeks in another study^62^. The reason for difference of BP between the two models are not known, but the difference in instruments used for the measurement could be a possible reason for not confirming high BP in our laboratory. We used a tail cuff sphygmomanometer while that laboratory inserted catheters into femoral vein and carotid artery. In addition, we did not limit the nutrition of FGR PN after weaning (P21) to maintain a low body weight at the end of the study. However, in this study, we focused on nutrition before weaning and found that early nutrition before weaning could impact later life even when the nutritional environment was the same after weaning. Only the volume of breast milk between the GN and PN groups was different in this study, although there might be a small difference in the composition of breast milk among dams. We did not examine the composition of the breast milk of each dam because of technical difficulties. Finally, we evaluated only FGR induced by owing to placental insufficiency, we will evaluate the other types of FGR in the following studies.

In conclusion, environmental factors—particularly nutrition during gestation and neonatal life—can permanently alter pancreatic islet morphology and dysregulate the expression of certain proteins. FGR, followed by catch-up growth immediately after birth, is a risk factor for islet fibrosis in male offspring and can impact pancreatic endocrine function well into adulthood. Moreover, FGR without catch-up growth caused dysregulated expression of microtubule/cytoskeleton- and cell adhesion-related proteins. In contrast, FGR followed by catch-up growth severely aggravated the expression of the cell adhesion-related proteins in male offspring. In this case, GN may actually be detrimental to FGR offspring. Thus, balanced nutrition that does not lead to excessive weight gain before weaning may protect the islets from fibrosis and metabolic dysfunction later in life, particularly in male offspring.

## Materials and methods

### Animals

We purchased 13 female pregnant SD rats on their 15^th^ day of pregnancy from Japan SLC Inc. (Shizuoka, Japan). All animals were maintained under controlled room temperature (23 °C) with a 12-h light/dark cycle. All rats were allowed free access to normal chow and tap water. All animal experiments were approved by the Institutional Review Board of Nagoya University (project numbers 31107 and 20033) and conducted according to the Regulations on Animal Experiments in Nagoya University. An investigator blinded to the group collected and analyzed the data. While the rat cages were numbered and each pup earmarked, these identifiers were not visible during the experiment. This study is reported following the ARRIVE guidelines (Animal Research: Reporting of in Vivo Experiments)^68^.

### Fetal growth restriction rat model

The FGR rat model was made by employing the method we previously reported^47^. On day 17 of pregnancy, pregnant rats were anesthetized with 2.5% isoflurane and underwent surgery. We operated on eight pregnant female rats for the FGR groups, and ameroid constrictors 0.4 mm in diameter were attached to the ovarian and uterine arteries bilaterally, while five sham mothers underwent laparotomy and gently handled the uterine horns. After surgery, the rats were returned to their clean individual cages under their normal housing conditions. All pregnant rats delivered naturally, and on P1, all pups were weighed.

**Figure 10:**
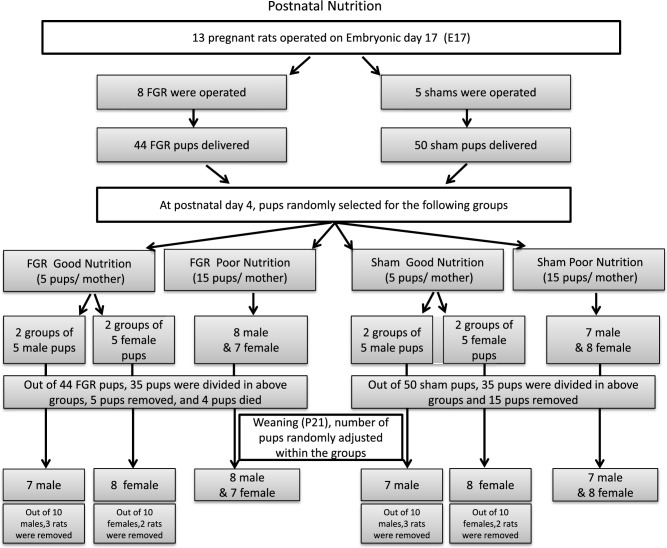
Animal participation in the experiments.

The minimal sample size of seven in each group was calculated to achieve an 80% power of testing with an error rate of 0.83% under the assumption based on the preliminary experiments that the difference of 100 mg/dl and standard deviation of 50 mg/dl in the intraperitoneal glucose tolerance test (IPGTT) is a primary endpoint. To ensure a minimum of 7 pups in each group for both males and females, we started with 13 pregnant rats, as shown in the flowchart (Fig. 10).

We reduced the number of pups per litter to induce rapid body weight gain after birth^69,70^. On P4, a group of 5 FGR pups was randomly allocated to one mother for good nutritional support. These pups were considered FGR pups with good nutrition (FGR-GN). For comparison, 15 pups were randomly allocated to another mother to increase the competition among pups (thereby limiting their access nutritional support). These rats were considered FGR pups with poor nutrition (FGR-PN). The pups of sham mothers were allocated into sham good nutrition (sham-GN) and sham poor nutrition (sham-PN) groups, with an adjusted number of pups immediately after birth, as in the FGR groups. On P21, the number of pups in the GN groups was randomly reduced to assure uniformity between the GN and PN groups; all pups were weaned, housed two pups per cage, and fed with standard chow food (food compositions: moisture 9.1%, crude protein 24.9%, crude fat 4.8%, crude fiber 4.6%, crude ash 7.1%, nitrogen-free extract 49.5%, and energy 340 kcal/100 g) ad libitum (Fig. 10, Flowchart).

### Body weight measurement

The offspring were weighed weekly until weaning (P21), every 2 weeks (14 days) until week 28, and every 4 weeks (28 days) until week 36 (Figure S1).

### Blood pressure measurement

Heart rate, systolic, diastolic, and mean blood pressures were measured using the cuff-tail technique with a sphygmomanometer (BP 98 AW, Softron, Tokyo, Japan) at 8, 16, 24, and 36 weeks (Figure S1). The rats were restrained during blood pressure measurements.

### Intraperitoneal glucose tolerance test

The IPGTT was performed after overnight fasting (16–18 h) at 8 and 24 weeks. A glucose solution (20% glucose; 2 g/kg body weight) was injected intraperitoneally, and blood glucose levels were measured using blood from the rat’s tail vein at 0 (just before glucose injection), 5, 15, 30, 60, 120, and 180 min after glucose administration^71^, using the FreeStyle Precision Neo (Abbot Japan Co., Ltd., 2105). The rat was restrained at each time point during blood draws. Different points on the tail vein were used for venipuncture at each time point. A drop of blood was added to the FreeStyle glucometer strip to measure fasting blood glucose levels.

### Tissue harvesting and blood collection

At 36 weeks, the rats were euthanized by exsanguination under 2.5% isoflurane inhalation. We dissected 0.05–0.1 g of pancreatic tissue from the same lobe. The samples were frozen with liquid nitrogen and stored at − 80 °C for proteome analysis. Blood was collected from the right atrium, centrifuged at 3000 revolutions/min for 15 min for serum isolation, then stored at − 80 °C until further analysis. Intracardial perfusion was performed using serum saline for 5 min and continued with 4% paraformaldehyde (PFA) in phosphate-buffered saline (PBS) for 6–8 min. Afterward, the remaining pancreatic tissue was dissected and steeped in 4% PFA overnight at 4 °C. The pancreatic tissue samples were transferred to 70% alcohol for soaking overnight at 4 °C, dehydrated with a graded series of ethanol and xylene, and embedded in paraffin. Next, 5-μm sections were cut every 200 μm and mounted on glass slides. The sections were used for immunohistochemical analysis of islet morphology and the insulin-positive area. Some sections were stained with Masson trichrome for fibrosis evaluation using a Masson trichrome stain kit (Modified Masson’s, ScyTek Laboratories, Inc., Logan, USA).

### Blood chemical analysis

Blood serum levels of TG, TCHO, and HDL were measured at 36 weeks using a dry chemistry analyzer (DRI-CHEM 7000v, Fujifilm Corp., Tokyo, Japan).

### Immunohistochemistry

Immunostaining was performed as previously described^72^. The sections were deparaffinized in Hemo-De (Falma Co., Ltd., Tokyo, Japan) and graded ethanol, washed with PBS, blocked with 4% Normal Donkey Serum and 10% Triton X-100 in PBS for 30 min, and incubated overnight at 4 °C with mouse anti-insulin antibody (1:200, 2D-11-H5, sc-8033, mouse monoclonal IgG, Santa Cruz, Biotechnology, Dallas, TX, USA) in 4% Normal Donkey Serum and 10% Triton X-100 in PBS. The following day, the sections were washed with PBS and incubated with a secondary antibody (1:400, Biotin-SP-conjugated AffiniPure Donkey, Anti-Mouse IgG (H + L), Jackson ImmunoResearch Laboratories, Inc., West Grove, PA, USA) in 4% Normal Donkey Serum and 10% Triton X-100 in PBS for 60 min. After washing with PBS, we blocked all endogenous peroxidase activity using 3% H_2_O_2_ in PBS for 10 min. Then, the sections were treated with an avidin–biotin-peroxidase complex (VECTASTAIN Elite ABC standard kit, Vector Laboratories, Inc., Burlingame, CA, USA) for 60 min and incubated with 0.1 M sodium acetate buffer at pH 6.0 for 5 min. This was followed by peroxidase detection for 10 min (0.12 mg/mL 3,3-diaminobenzidine, 0.01% H_2_O_2_, and 0.04% NiCl_2_) and then counterstained with hematoxylin and eosin to make the nuclei visible. Next, we measured the insulin-positive and total pancreatic areas using Cellsens Dimension (version 1.18, Olympus Corp., Tokyo, Japan) with three sections taken 200 μm apart.

### Protein preparation

The pancreatic tissue 0.05 g–0.1 g were collected from 36-week-old male and female offspring, randomly selected (n = 5/group). The samples were frozen using liquid nitrogen, ground to a powdered form using Multi-bead shocker (Yasui Kikai Co., Ltd., Osaka, Japan), and then homogenized in T-PER protein extraction reagent (20 mL/g of tissue) (Takara Bio. Inc., Shiga, Japan) containing a protease inhibitor cocktail (Complete Tablet, EDTA-free, Roche Diagnostics, Basel, Switzerland) at 4 °C. The homogenates were centrifuged at 10,000 × *g* for 10 min at 4 °C to remove debris, and then, the supernatants of the protein lysates were collected. To remove the surfactant from the supernatants, we used a Pierce detergent removal spin column (Thermo Fisher Scientific K.K., MA, USA). Total protein concentrations of the surfactant-free supernatants were quantified via the bicinchoninic acid method using the Pierce BCA Protein Assay kit (Thermo Fisher Scientific K.K., MA, USA). The protein lysate supernatants were adjusted to 100 µg/200 µL and labeled with the Tandem Mass Tag™ system (Sixplex TMT, Thermo Fisher Scientific, Waltham, MA, USA) for liquid chromatography/tandem mass spectrometry (LC/MS/MS).

### Proteomics

The expression levels of all types of proteins in the pancreas were comprehensively evaluated via LC/MS/MS. We used the Orbitrap Fusion mass spectrometry system (Thermo Fisher Scientific) in combination with the UltiMate 3000 RSLCnano LC system (Dionex Co., Amsterdam, Netherlands) and a nanocapillary column (150 mm × 75 μm i.d., Nikkyo Technos Co., Tokyo, Japan) with a nanoelectrospray ion source. In reversed-phase chromatography, the linear gradient flow rate (0 min, 5% B; 100 min, 40% B) of 2% acetonitrile with 0.1% formic acid solvent, and 95% acetonitrile solvent with 0.1% formic acid, was set at 300 nL/min. Prior to tandem MS analysis, a precursor ion scan was conducted at a 400–1600 mass-to-charge ratio (m/z). Tandem MS was carried out by quadrupole isolation at 0.8 Th, HCD fragmentation at 30% normalized collision energy, and rapid scan MS analysis in an ion trap. Only precursors with charge states of 2–6 were sampled for tandem MS. The dynamic exclusion time was set to 15 s, with a tolerance of 10 ppm. The instrument was run at maximum speed with a 3-s cycle. After quantification of the protein concentration, proteome software Scaffold (version 4.4.8, Proteome Software Inc., Portland, OR) was used to validate the tandem MS-based peptide and for protein identification. The proteome data were analyzed with Proteome Discoverer 1.4 (Thermo Fisher Scientific) and the MASCOT search engine (version 2.6.0, Matrix Science Inc., Boston, MA) to identify the proteins and peptides. For identification, we referred to a protein database in UniProt (release 2020_04) and set the precursor mass tolerance to 10 ppm and the fragment ion mass tolerance to 0.8 Da. The raw data were submitted to the Japan Proteome Standard Repository/Database (jPOST). The accession number of jPOST for the proteomics data used in this study is JPST001209 (PXD026502).

### Analytical process and extraction criteria for proteomics data

The expression levels of the detected proteins were statistically compared among the four groups (FGR GN, FGR PN, Sham GN, and Sham PN) of each sex using the nonparametric multiple Steel–Dwass test. The false discovery rate (FDR) was calculated using Storey’s method based on the p-value. The proteins affected by FGR and/or nutrition were extracted by threshold levels set at *P* < 0.05 and FDR < 0.10. The protein groups extracted from proteome data were divided as follows: proteins commonly affected by FGR regardless of nutritional condition (Fig. 7b-I, d-I), proteins affected by FGR only under GN (Fig. 7b-II, d-II), proteins affected by FGR only under PN (Fig. 7-III, d-III), proteins commonly affected by nutritional condition regardless of FGR or sham grouping (Fig. 7c-I, e-I), proteins affected by a nutritional condition were only observed in the FGR group (Fig. 7c-II, e-II), and proteins affected by a nutritional condition that were observed only in the sham group (Fig. 7c-III, e-III).

### Functional analysis

The protein profiles extracted by the 12 conditions (Fig. 7b-I–e-III) were used for the functional annotation analysis on the Database for Annotation, Visualization, and Integrated Discovery 6.8 (DAVID 6.8) (https://david.ncifcrf.gov/)^73^. We used the annotations database on April 20, 2020. Functional analysis using DAVID 6.8 to enrich the proteins of these 12 protein profiles into gene ontology (GO term). Then, the flagged GO terms were clustered to clarify the relationships among similar annotation and co-association proteins. The clustered GO terms with cluster enrichment scores ≥ 2 were considered significant categories. Furthermore, we visualized molecular networks among the proteins extracted in the clustered ontologies using the Search Tool for the Retrieval of Interacting Genes/Proteins 11.0 (STRING 11.0) (https://string-db.org/). The results, analyzed by STRING, were clustered using the Markov cluster algorithm (MCL clustering; the inflation parameter is 3). The minimum required interaction score was set to 0.400 and 0.150. This interaction was indicated by eight criteria for linkage, including neighborhood, gene fusion, co-occurrence, co-expression, experiments, databases, text mining, and homology. In addition, the distribution information of the extracted proteins in the pancreas was investigated using the histological database within the Human Protein Atlas (https://www.proteinatlas.org/).

### Statistics

All statistical analyses were performed using JMP Pro 14 software (SAS Institute Inc., Cary, NC, USA). Quantitative data are presented as mean ± SEM. Group comparisons were performed using one-way ANOVA, followed by comparisons of all pairs using Tukey’s test. We compared protein expression levels using the Steel–Dwass test. Statistical significance was set at *P* < 0.05.

## Data availability

The other datasets and materials generated during the current study are available from the corresponding author upon reasonable request.

## Supplementary Information

Supplementary Information 1.

Supplementary Information 2.

Supplementary Information 3.

Supplementary Information 4.

## Abbreviations

FGR: Fetal growth restriction
GN: Good nutrition
PN: Poor nutrition
GO: Gene ontology
