# Supplementary figures and images for: Fetal growth restriction followed by early catch-up growth impairs pancreatic islet morphology in male rats
Source: Sci Rep. 2023 Feb 15;13:2732. doi: 10.1038/s41598-023-28584-2 (PMC9932152; doi:10.1038/s41598-023-28584-2)

## Body weight and blood pressure measurement timeline

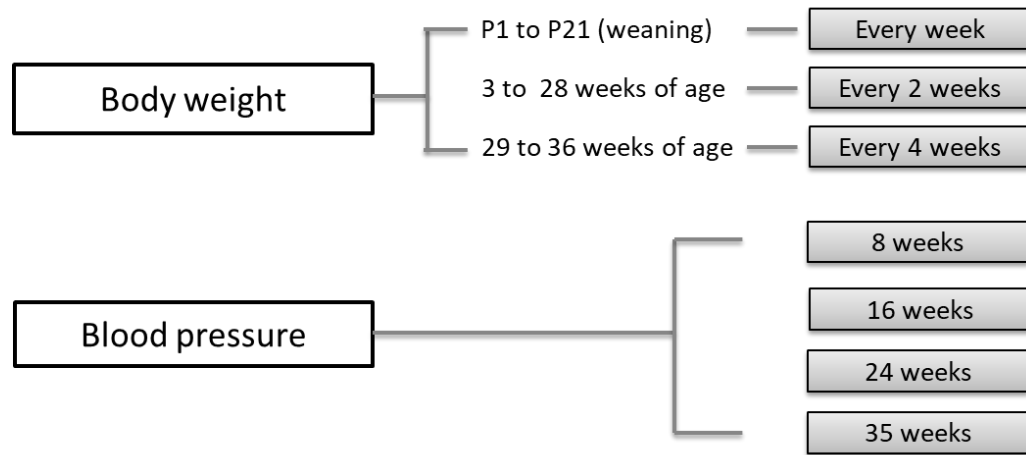

Supplement: Supplementary file 1 — Supplementary Information 1. [file 41598_2023_28584_MOESM1_ESM.pdf]
