# Supplementary material for: Fetal growth restriction followed by early catch-up growth impairs pancreatic islet morphology in male rats
Source: Sci Rep. 2023 Feb 15;13:2732. doi: 10.1038/s41598-023-28584-2 (PMC9932152; doi:10.1038/s41598-023-28584-2)
Supplement: Supplementary file 2 — Supplementary Information 2. [file 41598_2023_28584_MOESM2_ESM.pdf]

Table S1. Extracted protein profile of b-I (Effect of FGR, common, Male)

Accession	Description	MW [kDa]	Expression level																				p<0.05, q<0.10											
			FGR-Good-Male					FGR-Poor-Male					Sham-Good-Male					Sham-Poor-Male					Steel-Dwass & FDR											
			#21	#22	#23	#24	#25	#26	#27	#28	#29	#30	#31	#32	#33	#34	#35	#36	#37	#38	#39	#40	FGR-GN vs FGR-PN		FGR-GN vs Sham-GN		FGR-GN vs Sham-PN		FGR-PN vs Sham-GN		FGR-PN vs Sham-PN		Sham-GN vs Sham-PN	
Filter	Filter	Filter	Filter	Filter	Filter	Filter	Filter	Filter	Filter	Filter	Filter	Filter	Filter	Filter	Filter	Filter	Filter	Filter	Filter	Filter	Filter	Filter	p-value	q-value	p	q-value	p	q-value	p	q-value	p	q-value	p	q-value
A1L108	Actin-related protein 2/3 complex subunit 5-like protein OS=Rattus norvegicus GN=Arpc5l PE=1 SV=2 - [ARP5L_RAT]	17.0	0.013	0.013	0.013	0.013	0.013	0.013	0.013	0.013	0.013	0.013	0.874	0.959	0.763	1.022	0.349	0.523	0.905	0.830	0.585	0.861	#DIV/0!	#DIV/0!	0.009	0.087	0.009	0.096	0.009	0.108	0.009	0.043	0.470	0.859
B0K020	CDGSH iron-sulfur domain-containing protein 1 OS=Rattus norvegicus OX=10116 GN=Cisd1 PE=3 SV=1 - [CISD1_RAT]	12.1	0.013	0.013	0.013	0.013	0.013	0.013	0.013	0.013	0.013	0.013	0.622	0.800	0.850	1.637	0.280	0.808	0.668	0.954	0.538	0.866	#DIV/0!	#DIV/0!	0.009	0.062	0.009	0.087	0.009	0.084	0.009	0.036	0.756	0.810
B0LT89	Serine/threonine-protein kinase 24 OS=Rattus norvegicus GN=Stk24 PE=2 SV=1 - [STK24_RAT]	48.0	0.013	0.013	0.013	0.013	0.013	0.599	1.232	1.117	0.201	1.065	0.334	0.951	0.845	1.621	0.203	0.013	0.013	0.013	0.013	0.013	0.009	0.0391	0.009	0.089	#DIV/0!	#DIV/0!	0.756	0.785	0.009	0.026	0.009	0.110
B2RYD2	Epithelial splicing regulatory protein 1 OS=Rattus norvegicus OX=10116 GN=Esrp1 PE=2 SV=2 - [ESRP1_RAT]	75.0	0.013	0.013	0.013	0.013	0.013	0.013	0.013	0.013	0.013	0.013	0.537	0.970	1.000	1.559	0.408	0.569	0.827	1.180	0.703	1.017	#DIV/0!	#DIV/0!	0.009	0.045	0.009	0.064	0.009	0.066	0.009	0.025	0.756	0.753
B2RZD7	LYR motif-containing protein 9 OS=Rattus norvegicus OX=10116 GN=Lyrm9 PE=3 SV=1 - [LYRM9_RAT]	9.4	0.721	0.696	1.452	0.709	0.545	0.013	0.013	0.013	0.013	0.013	0.013	0.013	0.013	0.013	0.013	0.486	0.793	1.130	0.989	0.895	0.009	0.0426	0.009	0.068	0.470	0.801	#DIV/0!	#DIV/0!	0.009	0.025	0.009	0.074
B5DF89	Cullin-3 OS=Rattus norvegicus GN=Cul3 PE=1 SV=2 - [CUL3_RAT]	88.9	0.013	0.013	0.013	0.013	0.013	0.688	1.133	1.116	0.188	0.847	0.703	1.073	0.756	1.188	0.356	0.013	0.013	0.013	0.013	0.013	0.009	0.0504	0.009	0.071	#DIV/0!	#DIV/0!	0.917	0.796	0.009	0.032	0.009	0.090
D3ZC96	Tetratricopeptide repeat protein 39B OS=Rattus norvegicus GN=Ttc39b PE=3 SV=1 - [TT39B_RAT]	70.1	0.013	0.013	0.013	0.013	0.013	0.899	1.786	0.798	0.670	1.390	0.655	0.697	1.209	1.654	0.246	0.013	0.013	0.013	0.013	0.013	0.009	0.0231	0.009	0.046	#DIV/0!	#DIV/0!	0.354	0.590	0.009	0.016	0.009	0.063
G3V9R8	Heterogeneous nuclear ribonucleoprotein C OS=Rattus norvegicus GN=Hnrnpc PE=1 SV=2 - [HNRPC_RAT]	32.8	0.013	0.013	0.013	0.013	0.013	0.013	0.013	0.013	0.013	0.013	0.808	0.944	1.025	2.143	0.432	0.761	0.530	1.281	0.572	0.926	#DIV/0!	#DIV/0!	0.009	0.027	0.009	0.073	0.009	0.042	0.009	0.029	0.470	0.715
O08839	Myc box-dependent-interacting protein 1 OS=Rattus norvegicus GN=Bin1 PE=1 SV=1 - [BIN1_RAT]	64.5	0.013	0.013	0.013	0.013	0.013	0.013	0.013	0.013	0.013	0.013	0.916	1.270	0.994	1.124	0.512	0.549	1.152	1.376	1.048	1.188	#DIV/0!	#DIV/0!	0.009	0.034	0.009	0.046	0.009	0.050	0.009	0.017	0.354	0.755
O35112	CD166 antigen OS=Rattus norvegicus GN=Alcam PE=1 SV=1 - [CD166_RAT]	65.0	0.013	0.013	0.013	0.013	0.013	0.717	1.390	1.199	0.290	1.090	0.841	1.054	0.955	1.881	0.376	0.013	0.013	0.013	0.013	0.013	0.009	0.0288	0.009	0.030	#DIV/0!	#DIV/0!	0.917	0.719	0.009	0.020	0.009	0.043
O35303	Dynamin-1-like protein OS=Rattus norvegicus GN=Dnm1l PE=1 SV=1 - [DNM1L_RAT]	83.9	0.013	0.013	0.013	0.013	0.013	0.013	0.013	0.013	0.013	0.013	0.501	0.761	0.989	1.422	0.200	0.414	0.612	0.678	0.494	0.880	#DIV/0!	#DIV/0!	0.009	0.099	0.009	0.190	0.009	0.124	0.009	0.097	0.470	0.926
O88637	Ethanolamine-phosphate cytidylyltransferase OS=Rattus norvegicus GN=Pcyt2 PE=1 SV=1 - [PCY2_RAT]	45.2	0.013	0.013	0.013	0.013	0.013	0.716	1.076	1.252	0.115	0.909	0.712	0.956	0.883	1.435	0.258	0.013	0.013	0.013	0.013	0.013	0.009	0.0455	0.009	0.059	#DIV/0!	#DIV/0!	0.917	0.783	0.009	0.029	0.009	0.078
P04646	60S ribosomal protein L35a OS=Rattus norvegicus OX=10116 GN=Rpl35a PE=3 SV=1 - [RL35A_RAT]	12.5	0.013	0.013	0.013	0.013	0.013	0.013	0.013	0.013	0.013	0.013	0.676	1.160	1.319	0.702	0.526	0.502	0.624	1.260	0.700	0.565	#DIV/0!	#DIV/0!	0.009	0.050	0.009	0.102	0.009	0.070	0.009	0.045	0.354	0.824
P04692	Tropomyosin alpha-1 chain OS=Rattus norvegicus GN=Tpm1 PE=1 SV=3 - [TPM1_RAT]	32.7	0.013	0.013	0.013	0.013	0.013	0.013	0.013	0.013	0.013	0.013	1.057	1.283	1.070	1.644	1.080	1.050	1.097	1.673	0.821	1.119	#DIV/0!	#DIV/0!	0.009	0.025	0.009	0.044	0.009	0.038	0.009	0.016	0.756	0.715
P04897	Guanine nucleotide-binding protein G(i) subunit alpha-2 OS=Rattus norvegicus GN=Gnai2 PE=1 SV=3 - [GNAI2_RAT]	40.5	0.013	0.013	0.013	0.013	0.013	0.666	0.833	0.853	0.262	0.752	1.012	1.226	0.611	1.081	0.499	0.773	0.978	1.766	1.079	1.277	0.009	0.117	0.009	0.047	0.009	0.043	0.354	0.672	0.036	0.051	0.354	0.753
P11466	Peroxisomal carnitine O-octanoyltransferase OS=Rattus norvegicus GN=Crot PE=1 SV=3 - [OCTC_RAT]	70.3	0.929	0.874	1.115	0.469	0.600	1.076	1.233	1.410	0.730	0.985	0.013	0.013	0.013	0.013	0.013	0.013	0.013	0.013	0.013	0.013	0.127	0.221	0.009	0.081	0.009	0.077	0.009	0.041	0.009	0.017	#DIV/0!	#DIV/0!
P13437	3-ketoacyl-CoA thiolase, mitochondrial OS=Rattus norvegicus OX=10116 GN=Acaa2 PE=2 SV=1 - [THIM_RAT]	41.8	0.013	0.013	0.013	0.013	0.013	0.456	0.922	0.769	0.190	0.811	0.887	0.919	0.941	1.120	0.508	0.013	0.013	0.013	0.013	0.013	0.009	0.179	0.009	0.051	#DIV/0!	#DIV/0!	0.127	0.404	0.009	0.092	0.009	0.068
P14272	Plasma kallikrein OS=Rattus norvegicus GN=Klkb1 PE=1 SV=1 - [KLKB1_RAT]	71.2	0.013	0.013	0.013	0.013	0.013	1.219	1.414	1.292	0.613	1.578	0.583	0.963	1.113	1.108	0.246	0.013	0.013	0.013	0.013	0.013	0.009	0.0223	0.009	0.079	#DIV/0!	#DIV/0!	0.056	0.194	0.009	0.016	0.009	0.101
P15205	Microtubule-associated protein 1B OS=Rattus norvegicus OX=10116 GN=Map1b PE=1 SV=3 - [MAP1B_RAT]	269.5	0.013	0.013	0.013	0.013	0.013	0.013	0.013	0.013	0.013	0.013	1.350	1.860	0.439	0.323	1.121	0.543	1.825	1.402	2.155	0.609	#DIV/0!	#DIV/0!	0.009	0.030	0.009	0.041	0.009	0.046	0.009	0.015	0.354	0.750
P15865	Histone H1.4 OS=Rattus norvegicus GN=Hist1h1e PE=1 SV=3 - [H14_RAT]	22.0	0.973	0.428	1.470	0.857	0.649	1.545	2.219	1.265	0.095	1.270	0.013	0.013	0.013	0.013	0.013	0.013	0.013	0.013	0.013	0.013	0.259	0.346	0.009	0.050	0.009	0.061	0.009	0.037	0.009	0.015	#DIV/0!	#DIV/0!
P18645	UDP-glucose 4-epimerase OS=Rattus norvegicus GN=Gale PE=2 SV=1 - [GALE_RAT]	38.2	0.013	0.013	0.013	0.013	0.013	0.844	1.160	1.296	0.291	1.082	0.743	1.237	0.854	1.465	0.626	0.013	0.013	0.013	0.013	0.013	0.009	0.0295	0.009	0.033	#DIV/0!	#DIV/0!	0.917	0.722	0.009	0.021	0.009	0.046
P20611	Lysosomal acid phosphatase OS=Rattus norvegicus GN=Acp2 PE=1 SV=1 - [PPAL_RAT]	48.3	0.724	0.638	1.353	0.687	0.548	0.800	0.944	1.205	0.530	0.775	0.013	0.013	0.013	0.013	0.013	0.013	0.013	0.013	0.013	0.013	0.470	0.462	0.009	0.090	0.009	0.080	0.009	0.078	0.009	0.025	#DIV/0!	#DIV/0!
P23514	Coatomer subunit beta OS=Rattus norvegicus GN=Copb1 PE=1 SV=1 - [COPB_RAT]	106.9	0.013	0.013	0.013	0.013	0.013	0.013	0.013	0.013	0.013	0.013	0.722	0.842	1.135	1.519	0.182	0.660	0.315	1.183	0.352	1.266	#DIV/0!	#DIV/0!	0.009	0.048	0.009	0.089	0.009	0.068	0.009	0.038	0.756	0.797
P27791	cAMP-dependent protein kinase catalytic subunit alpha OS=Rattus norvegicus GN=Prkaca PE=1 SV=2 - [KAPCA_RAT]	40.6	0.013	0.013	0.013	0.013	0.013	0.876	1.219	1.415	0.427	0.999	0.551	1.272	1.108	1.309	0.313	0.013	0.013	0.013	0.013	0.013	0.009	0.0263	0.009	0.041	#DIV/0!	#DIV/0!	0.917	0.723	0.009	0.018	0.009	0.059
P28492	Glutaminase liver isoform, mitochondrial OS=Rattus norvegicus GN=Gls2 PE=2 SV=3 - [GLSL_RAT]	66.2	0.013	0.013	0.013	0.013	0.013	0.813	1.167	1.486	0.309	0.978	0.730	0.907	0.958	1.239	0.444	0.013	0.013	0.013	0.013	0.013	0.009	0.0282	0.009	0.056	#DIV/0!	#DIV/0!	0.605	0.693	0.009	0.020	0.009	0.075
P33124	Long-chain-fatty-acid--CoA ligase 6 OS=Rattus norvegicus OX=10116 GN=Acsl6 PE=1 SV=1 - [ACSL6_RAT]	78.1	0.013	0.013	0.013	0.013	0.013	0.520	1.405	0.981	0.069	1.087	1.206	1.277	1.040	1.703	0.703	0.013	0.013	0.013	0.013	0.013	0.009	0.0458	0.009	0.025	#DIV/0!	#DIV/0!	0.259	0.520	0.009	0.029	0.009	0.035
P35231	Regenerating islet-derived protein 3-alpha OS=Rattus norvegicus OX=10116 GN=Reg3a PE=2 SV=1 - [REG3A_RAT]	19.6	0.013	0.013	0.013	0.013	0.013	0.194	0.214	0.368	0.112	0.264	0.866	0.681	4.803	1.481	0.524	0.475	0.704	0.746	2.508	0.458	0.009	1.016	0.009	0.023	0.009	0.050	0.014	0.053	0.014	0.022	0.354	0.747
P35815	Protein phosphatase 1B OS=Rattus norvegicus GN=Ppm1b PE=2 SV=1 - [PPM1B_RAT]	42.9	0.013	0.013	0.013	0.013	0.013	0.013	0.013	0.013	0.013	0.013	0.533	1.102	0.906	1.193	0.252	0.636	0.949	1.041	0.895	1.137	#DIV/0!	#DIV/0!	0.009	0.080	0.009	0.054	0.009	0.102	0.009	0.021	0.756	0.759
P41565	Isocitrate dehydrogenase [NAD] subunit gamma 1, mitochondrial OS=Rattus norvegicus GN=Idh3g PE=2 SV=2 - [IDHG1_RAT]	42.8	0.013	0.013	0.013	0.013	0.013	0.552	1.243	0.920	0.027	1.106	0.534	0.851	1.075	1.369	0.236	0.013	0.013	0.013	0.013	0.013	0.009	0.0540	0.009	0.072	#DIV/0!	#DIV/0!	0.917	0.804	0.009	0.035	0.009	0.092
P50878	60S ribosomal protein L4 OS=Rattus norvegicus GN=Rpl4 PE=1 SV=3 - [RL4_RAT]	47.2	0.013	0.013	0.013	0.013	0.013	0.689	0.815	1.086	0.253	1.154	0.764	1.207	0.946	0.669	0.464	0.013	0.013	0.013	0.013	0.013	0.009	0.0492	0.009	0.076	#DIV/0!	#DIV/0!	0.917	0.796	0.009	0.031	0.009	0.096
P54311	Guanine nucleotide-binding protein G(I)/G(S)/G(T) subunit beta-1 OS=Rattus norvegicus GN=Gnb1 PE=1 SV=4 - [GBB1_RAT]	37.4	0.013	0.013	0.013	0.013	0.013	0.700	1.523	1.122	0.292	1.217	0.851	1.300	1.122	1.729	0.334	0.013	0.013	0.013	0.013	0.013	0.009	0.0272	0.009	0.027	#DIV/0!	#DIV/0!	0.605	0.672	0.009	0.019	0.009	0.039
P61354	60S ribosomal protein L27 OS=Rattus norvegicus OX=10116 GN=Rpl27 PE=2 SV=2 - [RL27_RAT]	15.8	0.013	0.013	0.013	0.013	0.013	0.013	0.013	0.013	0.013	0.013	0.609	0.939	1.126	1.020	0.371	0.483	0.711	0.954	0.532	0.676	#DIV/0!	#DIV/0!	0.009	0.074	0.009	0.131	0.009	0.097	0.009	0.069	0.470	0.889
P61515	Putative 60S ribosomal protein L37a OS=Rattus norvegicus OX=10116 GN=Rpl37a-ps1 PE=5 SV=2 - [RL37P_RAT]	10.3	0.470	0.768	0.727	0.390	0.398	0.709	0.865	1.161	0.165	0.928	0.914	1.159	1.078	0.826	0.542	0.013	0.013	0.013	0.013	0.013	0.259	0.384	0.036	0.090	0.009	0.337	0.756	0.766	0.009	0.036	0.009	0.060
P68182	cAMP-dependent protein kinase catalytic subunit beta OS=Rattus norvegicus GN=Prkacb PE=1 SV=2 - [KAPCB_RAT]	40.7	0.013	0.013	0.013	0.013	0.013	0.862	1.141	1.387	0.235	1.063	0.806	0.854	0.835	1.082	0.554	0.013	0.013	0.013	0.013	0.013	0.009	0.0286	0.009	0.067	#DIV/0!	#DIV/0!	0.259	0.552	0.009	0.020	0.009	0.086
P81795	Eukaryotic translation initiation factor 2 subunit 3 OS=Rattus norvegicus GN=Eif2s3 PE=1 SV=2 - [IF2G_RAT]	51.0	0.013	0.013	0.013	0.013	0.013	0.628	0.831	0.967	0.084	0.709	1.075	1.109	0.856	1.246	0.626	0.013	0.013	0.013	0.013	0.013	0.009	0.149	0.009	0.033	#DIV/0!	#DIV/0!	0.127	0.401	0.009	0.079	0.009	0.046
P82450	Sialate O-acetylesterase OS=Rattus norvegicus OX=10116 GN=Siae PE=1 SV=2 - [SIAE_RAT]	60.4	0.943	1.098	1.091	1.077	0.842	0.734	0.697	0.886	0.452	0.640	0.013	0.013	0.013	0.013	0.013	0.013	0.013	0.013	0.013	0.013	0.022	0.0511	0.009	0.031	0.009	0.048	0.009	0.267	0.009	0.065	#DIV/0!	#DIV/0!
P83941	Transcription elongation factor B polypeptide 1 OS=Rattus norvegicus GN=Tceb1 PE=1 SV=1 - [ELOC_RAT]	12.5	0.013	0.013	0.013	0.013	0.013	0.799	0.786	1.218	0.109	0.657	0.775	1.162	1.060	1.297	0.420	0.013	0.013	0.013	0.013	0.013	0.009	0.0772	0.009	0.036	#DIV/0!	#DIV/0!	0.470	0.714	0.009	0.049	0.009	0.050
P84817	Mitochondrial fission 1 protein OS=Rattus norvegicus GN=Fis1 PE=1 SV=1 - [FIS1_RAT]	17.0	0.013	0.013	0.013	0.013	0.013	0.593	1.319	0.875	0.117	0.771	0.742	0.881	0.830	1.139	0.458	0.013	0.013	0.013	0.013	0.013	0.009	0.0693	0.009	0.075	#DIV/0!	#DIV/0!	0.756	0.813	0.009	0.044	0.009	0.095
P85125	Polymerase I and transcript release factor OS=Rattus norvegicus GN=Ptrf PE=1 SV=1 - [PTRF_RAT]	43.9	0.013	0.013	0.013	0.013	0.013	0.787	1.209	1.116	0.294	1.041	0.672	0.913	0.999	1.278	0.430	0.013	0.013	0.013	0.013	0.013	0.009	0.0339	0.009	0.055	#DIV/0!	#DIV/0!	0.756	0.729	0.009	0.023	0.009	0.073
P97526	Neurofibromin OS=Rattus norvegicus OX=10116 GN=Nf1 PE=1 SV=1 - [NF1_RAT]	316.9	1.389	0.936	1.600	0.947	0.558	0.013	0.013	0.013	0.013	0.013	0.013	0.013	0.013	0.013	0.013	0.367	0.914	0.831	0.703	0.890	0.009	0.0234	0.009	0.027	0.085	0.326	#DIV/0!	#DIV/0!	0.009	0.043	0.009	0.156
P97675	Ectonucleotide pyrophosphatase/phosphodiesterase family member 3 OS=Rattus norvegicus GN=Enpp3 PE=1 SV=2 - [ENPP3_RAT]	99.0	0.013	0.013	0.013	0.013	0.013	0.712	1.810	1.112	0.562	1.235	1.051	1.186	1.113	1.740	0.817	0.013	0.013	0.013	0.013	0.013	0.009	0.0233	0.009	0.025	#DIV/0!	#DIV/0!	0.756	0.689	0.009	0.017	0.009	0.035
P97687	Ectonucleoside triphosphate diphosphohydrolase 1 OS=Rattus norvegicus OX=10116 GN=Entpd1 PE=1 SV=1 - [ENTP1_RAT]	57.4	0.013	0.013	0.013	0.013	0.013	0.013	0.013	0.013	0.013	0.013	1.005	1.105	0.960	1.725	0.442	0.962	0.420	0.994	0.312	1.244	#DIV/0!	#DIV/0!	0.009	0.028	0.009	0.081	0.009	0.044	0.009	0.032	0.354	0.774
Q01986	Dual specificity mitogen-activated protein kinase kinase 1 OS=Rattus norvegicus GN=Map2k1 PE=1 SV=2 - [MP2K1_RAT]	43.4	0.013	0.013	0.013	0.013	0.013	0.675	1.281	0.950	0.330	1.404	0.575	1.020	0.848	1.435	0.276	0.160	0.273	0.296	0.487	0.492	0.009	0.0308	0.009	0.065	0.009	1.095	0.756	0.725	0.036	0.053	0.056	0.203
Q07984	Translocon-associated protein subunit delta OS=Rattus norvegicus OX=10116 GN=Ssr4 PE=2 SV=1 - [SSRD_RAT]	19.0	0.013	0.013	0.013	0.013	0.013	0.661	1.414	1.123	0.290	1.117	0.634	1.024	1.411	2.133	0.352	0.013	0.013	0.013	0.013	0.013	0.009	0.0311	0.009	0.026	#DIV/0!	#DIV/0!	0.917	0.717	0.009	0.022	0.009	0.037
Q10728	Protein phosphatase 1 regulatory subunit 12A OS=Rattus norvegicus GN=Ppp1r12a PE=1 SV=2 - [MYPT1_RAT]	115.2	1.135	0.784	1.478	0.642	0.613	0.013	0.013	0.013	0.013	0.013	0.013	0.013	0.013	0.013	0.013	0.630	0.977	1.072	0.735	0.933	0.009	0.0302	0.009	0.038	0.917	0.865	#DIV/0!	#DIV/0!	0.009	0.024	0.009	0.069
Q2PQA9	Kinesin-1 heavy chain OS=Rattus norvegicus GN=Kif5b PE=1 SV=1 - [KINH_RAT]	109.5	0.013	0.013	0.013	0.013	0.013	0.810	1.625	0.822	0.631	1.125	0.601	0.829	1.067	1.307	0.372	0.013	0.013	0.013	0.013	0.013	0.009	0.0257	0.009	0.064	#DIV/0!	#DIV/0!	0.605	0.687	0.009	0.018	0.009	0.081
Q3B8N8	Pescadillo homolog OS=Rattus norvegicus OX=10116 GN=Pes1 PE=2 SV=1 - [PESC_RAT]	67.6	0.013	0.013	0.013	0.013	0.013	0.013	0.013	0.013	0.013	0.013	0.908	0.997	1.306	1.981	0.454	0.933	1.193	1.183	0.751	1.939	#DIV/0!	#DIV/0!	0.009	0.026	0.009	0.042	0.009	0.040	0.009	0.016	0.917	0.738
Q4FZV0	Beta-mannosidase OS=Rattus norvegicus GN=Manba PE=2 SV=1 - [MANBA_RAT]	101.2	0.804	0.677	1.759	1.040	0.732	0.864	0.884	1.049	0.703	0.891	0.013	0.013	0.013	0.013	0.013	0.013	0.013	0.013	0.013	0.013	0.756	0.511	0.009	0.032	0.009	0.049	0.009	0.069	0.009	0.023	#DIV/0!	#DIV/0!
Q5EGY4	Synaptobrevin homolog YKT6 OS=Rattus norvegicus GN=Ykt6 PE=1 SV=1 - [YKT6_RAT]	22.4	0.013	0.013	0.013	0.013	0.013	0.842	1.228	1.265	0.285	1.054	0.539	0.996	0.851	1.319	0.331	0.013	0.013	0.013	0.013	0.013	0.009	0.0293	0.009	0.078	#DIV/0!	#DIV/0!	0.756	0.732	0.009	0.021	0.009	0.0998
Q5TKR9	Histone acetyltransferase KAT6A OS=Rattus norvegicus OX=10116 GN=Kat6a PE=1 SV=2 - [KAT6A_RAT]	223.2	0.013	0.013	0.013	0.013	0.013	0.724	0.850	0.934	0.841	4.188	0.755	0.977	0.906	1.388	2.835	0.013	0.013	0.013	0.013	0.013	0.009	0.0216	0.009	0.024	#DIV/0!	#DIV/0!	0.470	0.636	0.009	0.015	0.009	0.034
Q5U2Z3	Nucleosome assembly protein 1-like 4 OS=Rattus norvegicus GN=Nap1l4 PE=1 SV=1 - [NP1L4_RAT]	43.9	0.013	0.013	0.013	0.013	0.013	0.852	1.202	0.963	0.447	0.765	0.554	0.728	0.779	1.564	0.291	0.013	0.013	0.013	0.013	0.013	0.009	0.0381	0.009	0.093	#DIV/0!	#DIV/0!	0.470	0.724	0.009	0.026	0.009	0.113
Q5XIM5	Protein CDV3 homolog OS=Rattus norvegicus OX=10116 GN=Cdv3 PE=1 SV=1 - [CDV3_RAT]	24.3	0.013	0.013	0.013	0.013	0.013	0.013	0.013	0.013	0.013	0.013	1.286	0.856	1.045	1.684	1.050	0.811	1.306	1.614	0.864	1.013	#DIV/0!	#DIV/0!	0.009	0.025	0.009	0.044	0.009	0.038	0.009	0.016	0.605	0.721
Q5XIN6	Mitochondrial proton/calcium exchanger protein OS=Rattus norvegicus OX=10116 GN=Letm1 PE=1 SV=1 - [LETM1_RAT]	83.0	0.013	0.013	0.013	0.013	0.013	0.013	0.013	0.013	0.013	0.013	0.744	1.102	0.674	1.396	0.332	0.709	0.696	0.896	0.675	1.262	#DIV/0!	#DIV/0!	0.009	0.058	0.009	0.066	0.009	0.079	0.009	0.026	0.917	0.781
Q62636	Ras-related protein Rap-1b OS=Rattus norvegicus GN=Rap1b PE=2 SV=2 - [RAP1B_RAT]	20.8	0.013	0.013	0.013	0.013	0.013	0.736	1.088	1.174	0.340	0.972	0.620	0.961	0.846	1.624	0.286	0.013	0.013	0.013	0.013	0.013	0.009	0.0354	0.009	0.052	#DIV/0!	#DIV/0!	0.605	0.716	0.009	0.024	0.009	0.070
Q62698	Cytoplasmic dynein 1 light intermediate chain 2 OS=Rattus norvegicus GN=Dync1li2 PE=1 SV=1 - [DC1L2_RAT]	54.7	0.013	0.013	0.013	0.013	0.013	0.527	0.696	1.511	0.013	0.762	0.910	0.672	1.153	2.786	0.225	0.013	0.013	0.013	0.013	0.013	0.025	0.0557	0.009	0.026	#DIV/0!	#DIV/0!	0.470	0.647	0.025	0.037	0.009	0.036
Q62764	Y-box-binding protein 3 OS=Rattus norvegicus OX=10116 GN=Ybx3 PE=1 SV=1 - [YBOX3_RAT]	38.8	0.013	0.013	0.013	0.013	0.013	1.270	1.058	1.457	0.443	0.566	1.834	1.612	1.070	1.409	0.664	0.013	0.013	0.013	0.013	0.013	0.009	0.0278	0.009	0.024	#DIV/0!	#DIV/0!	0.184	0.451	0.009	0.019	0.009	0.034
Q62826	Heterogeneous nuclear ribonucleoprotein M OS=Rattus norvegicus GN=Hnrnpm PE=1 SV=4 - [HNRPM_RAT]	73.7	0.013	0.013	0.013	0.013	0.013	0.013	0.013	0.013	0.013	0.013	0.566	0.752	1.390	1.599	0.255	0.648	0.446	1.010	0.452	0.891	#DIV/0!	#DIV/0!	0.009	0.040	0.009	0.123	0.009	0.060	0.009	0.060	0.605	0.799
Q62871	Cytoplasmic dynein 1 intermediate chain 2 OS=Rattus norvegicus GN=Dync1i2 PE=1 SV=1 - [DC1I2_RAT]	71.1	0.013	0.013	0.013	0.013	0.013	0.689	0.959	1.152	0.167	0.853	0.889	0.932	0.996	1.220	0.546	0.013	0.013	0.013	0.013	0.013	0.009	0.0570	0.009	0.040	#DIV/0!	#DIV/0!	0.470	0.703	0.009	0.037	0.009	0.058
Q62920	PDZ and LIM domain protein 5 OS=Rattus norvegicus GN=Pdlim5 PE=1 SV=2 - [PDLI5_RAT]	63.2	0.013	0.013	0.013	0.013	0.013	0.013	0.013	0.013	0.013	0.013	0.790	1.043	0.964	1.283	0.440	0.676	0.800	1.125	0.661	0.850	#DIV/0!	#DIV/0!	0.009	0.042	0.009	0.071	0.009	0.063	0.009	0.027	0.605	0.760
Q62951	Dihydropyrimidinase-related protein 4 (Fragment) OS=Rattus norvegicus GN=Dpysl4 PE=1 SV=1 - [DPYL4_RAT]	61.0	1.872	0.805	2.583	1.132	1.067	0.013	0.013	0.013	0.013	0.013	0.013	0.013	0.013	0.013	0.013	0.558	1.544	1.378	1.266	0.922	0.009	0.0217	0.009	0.024	0.605	0.810	#DIV/0!	#DIV/0!	0.009	0.016	0.009	0.036
Q63450	Calcium/calmodulin-dependent protein kinase type 1 OS=Rattus norvegicus GN=Camk1 PE=1 SV=2 - [KCC1A_RAT]	41.6	0.013	0.013	0.013	0.013	0.013	0.013	0.013	0.013	0.013	0.013	0.983	1.487	1.356	1.975	0.368	0.629	0.795	1.153	0.612	1.169	#DIV/0!	#DIV/0!	0.009	0.024	0.009	0.062	0.009	0.038	0.009	0.024	0.259	0.682
Q63798	Proteasome activator complex subunit 2 OS=Rattus norvegicus GN=Psme2 PE=2 SV=3 - [PSME2_RAT]	26.8	0.013	0.013	0.013	0.013	0.013	0.750	1.034	1.186	0.212	0.744	0.647	0.822	0.988	1.170	0.677	0.013	0.013	0.013	0.013	0.013	0.009	0.0521	0.009	0.054	#DIV/0!	#DIV/0!	0.917	0.789	0.009	0.033	0.009	0.071
Q64560	Tripeptidyl-peptidase 2 OS=Rattus norvegicus GN=Tpp2 PE=2 SV=3 - [TPP2_RAT]	138.2	0.740	0.879	1.259	0.401	0.612	0.013	0.013	0.013	0.013	0.013	0.013	0.013	0.013	0.013	0.013	0.442	0.649	0.884	0.629	0.967	0.009	0.0535	0.009	0.095	0.917	0.935	#DIV/0!	#DIV/0!	0.009	0.049	0.009	0.204
Q64620	Serine/threonine-protein phosphatase 6 catalytic subunit OS=Rattus norvegicus GN=Ppp6c PE=2 SV=2 - [PPP6_RAT]	35.1	0.013	0.013	0.013	0.013	0.013	0.763	0.825	0.978	0.461	0.697	0.989	1.055	1.000	1.188	0.433	0.013	0.013	0.013	0.013	0.013	0.009	0.0642	0.009	0.038	#DIV/0!	#DIV/0!	0.127	0.392	0.009	0.042	0.009	0.054
Q66HA6	ADP-ribosylation factor-like protein 8B OS=Rattus norvegicus GN=Arl8b PE=2 SV=1 - [ARL8B_RAT]	21.5	0.013	0.013	0.013	0.013	0.013	0.579	1.067	0.946	0.251	1.297	0.887	1.060	0.629	1.533	0.532	0.013	0.013	0.013	0.013	0.013	0.009	0.0423	0.009	0.038	#DIV/0!	#DIV/0!	0.917	0.744	0.009	0.027	0.009	0.055
Q66HL2	Src substrate cortactin OS=Rattus norvegicus GN=Cttn PE=1 SV=1 - [SRC8_RAT]	56.9	0.013	0.013	0.013	0.013	0.013	0.722	0.762	1.152	0.192	0.670	0.925	1.083	0.938	1.400	0.788	0.013	0.013	0.013	0.013	0.013	0.009	0.0884	0.009	0.029	#DIV/0!	#DIV/0!	0.085	0.280	0.009	0.054	0.009	0.042
Q6AY23	Pyrroline-5-carboxylate reductase 2 OS=Rattus norvegicus OX=10116 GN=Pycr2 PE=2 SV=1 - [P5CR2_RAT]	33.7	0.013	0.013	0.013	0.013	0.013	0.673	1.638	1.008	0.168	1.508	0.702	1.098	1.317	1.768	0.256	0.013	0.013	0.013	0.013	0.013	0.009	0.0258	0.009	0.029	#DIV/0!	#DIV/0!	0.756	0.689	0.009	0.018	0.009	0.041
Q6AY46	tRNA (adenine(58)-N(1))-methyltransferase catalytic subunit TRMT61A OS=Rattus norvegicus OX=10116 GN=Trmt61a PE=2 SV=1 - [TRM61_RAT]	31.6	0.013	0.013	0.013	0.013	0.013	0.798	0.702	1.008	1.023	0.581	0.883	0.904	1.020	1.393	0.284	0.013	0.013	0.013	0.013	0.013	0.009	0.0435	0.009	0.045	#DIV/0!	#DIV/0!	0.605	0.722	0.009	0.028	0.009	0.063
Q6AYB5	Signal recognition particle 54 kDa protein OS=Rattus norvegicus GN=Srp54 PE=2 SV=1 - [SRP54_RAT]	55.7	0.013	0.013	0.013	0.013	0.013	0.013	0.013	0.013	0.013	0.013	0.489	0.938	1.074	1.688	0.237	0.598	0.426	1.004	0.472	1.022	#DIV/0!	#DIV/0!	0.009	0.048	0.009	0.114	0.009	0.068	0.009	0.051	0.605	0.806
Q6AYT3	tRNA-splicing ligase RtcB homolog OS=Rattus norvegicus GN=Rtcb PE=2 SV=1 - [RTCB_RAT]	55.2	0.874	0.636	1.378	0.562	0.534	0.659	0.854	1.091	0.316	1.113	0.013	0.013	0.013	0.013	0.013	0.013	0.013	0.013	0.013	0.013	0.756	0.553	0.009	0.082	0.009	0.078	0.009	0.099	0.009	0.030	#DIV/0!	#DIV/0!
Q6B345	Protein S100-A11 OS=Rattus norvegicus GN=S100a11 PE=3 SV=1 - [S10AB_RAT]	11.1	0.013	0.013	0.013	0.013	0.013	0.806	1.205	1.133	0.207	1.156	0.515	0.563	1.105	1.926	0.264	0.013	0.013	0.013	0.013	0.013	0.009	0.0326	0.009	0.051	#DIV/0!	#DIV/0!	0.605	0.702	0.009	0.023	0.009	0.069
Q6IG02	Keratin, type II cytoskeletal 2 epidermal OS=Rattus norvegicus GN=Krt2 PE=3 SV=1 - [K22E_RAT]	69.1	0.013	0.013	0.013	0.013	0.013	1.056	1.117	0.802	0.560	1.108	0.932	0.795	1.322	1.623	0.756	0.013	0.013	0.013	0.013	0.013	0.009	0.0306	0.009	0.027	#DIV/0!	#DIV/0!	0.756	0.690	0.009	0.021	0.009	0.038
Q6IG04	Keratin, type II cytoskeletal 72 OS=Rattus norvegicus GN=Krt72 PE=3 SV=2 - [K2C72_RAT]	56.8	0.013	0.013	0.013	0.013	0.013	0.013	0.013	0.013	0.013	0.013	0.838	0.939	1.083	1.215	0.461	0.420	0.755	0.630	0.429	0.971	#DIV/0!	#DIV/0!	0.009	0.041	0.009	0.165	0.009	0.062	0.009	0.080	0.127	0.434
Q6P7A9	Lysosomal alpha-glucosidase OS=Rattus norvegicus GN=Gaa PE=2 SV=1 - [LYAG_RAT]	106.1	0.891	0.653	1.429	0.692	0.601	0.013	0.013	0.013	0.013	0.013	0.013	0.013	0.013	0.013	0.013	0.716	0.996	1.276	0.856	1.220	0.009	0.0367	0.009	0.058	0.259	0.638	#DIV/0!	#DIV/0!	0.009	0.018	0.009	0.044
Q6QI44	N-alpha-acetyltransferase 25, NatB auxiliary subunit OS=Rattus norvegicus GN=Naa25 PE=2 SV=1 - [NAA25_RAT]	111.2	0.013	0.013	0.013	0.013	0.013	0.608	0.967	1.119	0.065	0.740	1.551	0.918	0.705	1.161	0.873	0.013	0.013	0.013	0.013	0.013	0.009	0.0871	0.009	0.029	#DIV/0!	#DIV/0!	0.259	0.563	0.009	0.053	0.009	0.041
Q6UPE1	Electron transfer flavoprotein-ubiquinone oxidoreductase, mitochondrial OS=Rattus norvegicus OX=10116 GN=Etfdh PE=1 SV=1 - [ETFD_RAT]	68.2	0.013	0.013	0.013	0.013	0.013	0.633	1.055	0.722	0.332	0.970	0.896	0.969	0.843	1.445	0.431	0.013	0.013	0.013	0.013	0.013	0.009	0.0663	0.009	0.039	#DIV/0!	#DIV/0!	0.605	0.751	0.009	0.042	0.009	0.058
Q6VV72	Eukaryotic translation initiation factor 1A OS=Rattus norvegicus OX=10116 GN=Eif1a PE=2 SV=3 - [IF1A_RAT]	16.5	0.013	0.013	0.013	0.013	0.013	0.681	0.584	0.988	0.101	0.396	1.462	1.291	0.916	1.230	0.976	0.721	1.337	1.646	1.272	0.977	0.009	0.277	0.009	0.025	0.009	0.043	0.036	0.132	0.036	0.051	0.756	0.716
Q78P75	Dynein light chain 2, cytoplasmic OS=Rattus norvegicus GN=Dynll2 PE=1 SV=1 - [DYL2_RAT]	10.3	0.013	0.013	0.013	0.013	0.013	0.701	1.000	1.273	0.271	0.805	0.417	0.913	1.076	2.074	0.200	0.013	0.013	0.013	0.013	0.013	0.009	0.0469	0.009	0.037	#DIV/0!	#DIV/0!	0.917	0.747	0.009	0.030	0.009	0.053
Q7TNY6	Golgi resident protein GCP60 OS=Rattus norvegicus OX=10116 GN=Acbd3 PE=1 SV=3 - [GCP60_RAT]	60.4	0.013	0.013	0.013	0.013	0.013	0.665	1.072	1.081	0.478	0.879	1.077	1.009	0.694	1.280	0.509	0.013	0.013	0.013	0.013	0.013	0.009	0.0415	0.009	0.040	#DIV/0!	#DIV/0!	0.605	0.710	0.009	0.027	0.009	0.058
Q80U96	Exportin-1 OS=Rattus norvegicus GN=Xpo1 PE=1 SV=1 - [XPO1_RAT]	123.0	0.013	0.013	0.013	0.013	0.013	0.013	0.013	0.013	0.013	0.013	0.777	0.981	0.845	1.319	0.287	0.473	0.649	0.703	0.333	1.507	#DIV/0!	#DIV/0!	0.009	0.060	0.009	0.0995	0.009	0.081	0.009	0.045	0.470	0.814
Q8CGV7	Thiamine-triphosphatase OS=Rattus norvegicus GN=Thtpa PE=2 SV=3 - [THTPA_RAT]	24.5	1.150	0.952	1.704	0.455	0.799	0.013	0.013	0.013	0.013	0.013	0.013	0.013	0.013	0.013	0.013	0.730	1.031	1.409	0.899	1.064	0.009	0.0254	0.009	0.031	0.917	0.844	#DIV/0!	#DIV/0!	0.009	0.017	0.009	0.042
Q91ZN1	Coronin-1A OS=Rattus norvegicus GN=Coro1a PE=1 SV=3 - [COR1A_RAT]	51.0	0.013	0.013	0.013	0.013	0.013	0.713	1.578	1.572	0.226	0.647	0.178	0.625	0.855	1.526	0.013	0.013	0.013	0.013	0.013	0.013	0.009	0.0284	0.025	0.064	#DIV/0!	#DIV/0!	0.259	0.601	0.009	0.020	0.025	0.092
Q99MZ8	LIM and SH3 domain protein 1 OS=Rattus norvegicus GN=Lasp1 PE=1 SV=1 - [LASP1_RAT]	30.0	0.013	0.013	0.013	0.013	0.013	0.718	0.681	0.889	0.032	0.568	1.118	1.059	0.959	2.198	0.720	0.880	1.003	1.773	0.799	1.089	0.009	0.234	0.009	0.025	0.009	0.044	0.022	0.086	0.036	0.052	0.756	0.717
Q99P74	Ras-related protein Rab-27B OS=Rattus norvegicus OX=10116 GN=Rab27b PE=2 SV=3 - [RB27B_RAT]	24.6	0.013	0.013	0.013	0.013	0.013	0.575	1.522	1.302	0.251	1.139	0.875	1.104	0.814	1.904	0.412	0.013	0.013	0.013	0.013	0.013	0.009	0.0281	0.009	0.030	#DIV/0!	#DIV/0!	0.917	0.718	0.009	0.020	0.009	0.043
Q9EPF2	Cell surface glycoprotein MUC18 OS=Rattus norvegicus OX=10116 GN=Mcam PE=1 SV=2 - [MUC18_RAT]	71.3	0.013	0.013	0.013	0.013	0.013	0.769	0.819	0.908	0.420	0.640	0.537	0.942	1.169	1.592	0.484	0.013	0.013	0.013	0.013	0.013	0.009	0.0792	0.009	0.036	#DIV/0!	#DIV/0!	0.354	0.647	0.009	0.050	0.009	0.050
Q9EQP5	Prolargin OS=Rattus norvegicus GN=Prelp PE=2 SV=1 - [PRELP_RAT]	43.2	0.013	0.013	0.013	0.013	0.013	0.807	1.347	1.266	0.223	1.149	0.512	0.779	0.969	1.812	0.200	0.013	0.013	0.013	0.013	0.013	0.009	0.0280	0.009	0.056	#DIV/0!	#DIV/0!	0.470	0.659	0.009	0.020	0.009	0.076
Q9ERE4	Golgi phosphoprotein 3 OS=Rattus norvegicus GN=Golph3 PE=1 SV=1 - [GOLP3_RAT]	33.8	0.013	0.013	0.013	0.013	0.013	0.729	2.200	1.804	0.280	1.717	0.728	0.980	0.975	2.073	0.632	0.013	0.013	0.013	0.013	0.013	0.009	0.0219	0.009	0.027	#DIV/0!	#DIV/0!	0.605	0.670	0.009	0.015	0.009	0.038
Q9ESS6	Basal cell adhesion molecule OS=Rattus norvegicus OX=10116 GN=Bcam PE=2 SV=1 - [BCAM_RAT]	67.5	0.013	0.013	0.013	0.013	0.013	0.544	1.033	0.880	0.301	0.587	0.888	1.059	0.926	1.297	0.421	0.013	0.013	0.013	0.013	0.013	0.009	0.122	0.009	0.039	#DIV/0!	#DIV/0!	0.184	0.505	0.009	0.071	0.009	0.057
Q9JIL3	Interleukin enhancer-binding factor 3 OS=Rattus norvegicus GN=Ilf3 PE=1 SV=2 - [ILF3_RAT]	95.9	0.013	0.013	0.013	0.013	0.013	0.895	0.954	1.183	0.321	0.770	0.710	0.994	1.037	1.146	0.380	0.013	0.013	0.013	0.013	0.013	0.009	0.0429	0.009	0.057	#DIV/0!	#DIV/0!	0.756	0.762	0.009	0.027	0.009	0.077
Q9JJ50	Hepatocyte growth factor-regulated tyrosine kinase substrate OS=Rattus norvegicus GN=Hgs PE=1 SV=1 - [HGS_RAT]	86.2	0.013	0.013	0.013	0.013	0.013	0.620	0.985	1.068	0.216	1.151	0.980	1.159	0.819	1.540	0.565	0.013	0.013	0.013	0.013	0.013	0.009	0.0476	0.009	0.030	#DIV/0!	#DIV/0!	0.605	0.692	0.009	0.030	0.009	0.044
Q9JK25	CAP-Gly domain-containing linker protein 1 OS=Rattus norvegicus OX=10116 GN=Clip1 PE=1 SV=2 - [CLIP1_RAT]	148.2	0.013	0.013	0.013	0.013	0.013	1.063	0.395	1.392	0.013	0.429	0.567	1.024	1.250	1.816	0.269	0.013	0.013	0.013	0.013	0.013	0.025	0.0559	0.009	0.033	#DIV/0!	#DIV/0!	0.470	0.720	0.025	0.037	0.009	0.046
Q9JMB5	Proteasomal ubiquitin receptor ADRM1 OS=Rattus norvegicus GN=Adrm1 PE=2 SV=2 - [ADRM1_RAT]	42.1	0.013	0.013	0.013	0.013	0.013	0.919	0.654	1.556	0.161	0.604	0.725	1.232	1.071	1.747	0.632	0.013	0.013	0.013	0.013	0.013	0.009	0.0530	0.009	0.027	#DIV/0!	#DIV/0!	0.259	0.530	0.009	0.034	0.009	0.038
Q9QWN8	Spectrin beta chain, non-erythrocytic 2 OS=Rattus norvegicus GN=Sptbn2 PE=1 SV=2 - [SPTN2_RAT]	270.9	0.013	0.013	0.013	0.013	0.013	0.427	1.030	0.886	0.269	0.890	0.926	1.158	0.709	1.185	0.512	0.013	0.013	0.013	0.013	0.013	0.009	0.0835	0.009	0.043	#DIV/0!	#DIV/0!	0.259	0.595	0.009	0.053	0.009	0.061
Q9Z336	Dynein light chain Tctex-type 1 OS=Rattus norvegicus GN=Dynlt1 PE=1 SV=1 - [DYLT1_RAT]	12.4	0.013	0.013	0.013	0.013	0.013	0.636	1.401	1.058	0.118	1.189	0.338	0.784	1.418	1.356	0.142	0.013	0.013	0.013	0.013	0.013	0.009	0.0341	0.009	0.078	#DIV/0!	#DIV/0!	0.917	0.771	0.009	0.023	0.009	0.099

Table S2. Extracted protein profile of d-I (Effect of FGR, common, Female)

Accession	Description	MW [kDa]	Expression level																				Steel-Dwass & FDR											
			FGR-Good-Female					FGR-Poor_Female					Sham-Good-Female					Sham-Poor-Female					FGR-GN vs FGR-PN		FGR-GN vs Sham-GN		FGR-GN vs Sham-PN		FGR-PN vs Sham-GN		FGR-PN vs Sham-PN		Sham-GN vs Sham-PN	
			#01	#02	#03	#04	#05	#06	#07	#08	#09	#10	#11	#12	#13	#14	#15	#16	#17	#18	#19	#20	p-value	q-value	p	q-value	p	q-value	p	q-value	p	q-value	p	q-value
Filter	Filter	Filter	Filter	Filter	Filter	Filter	Filter	Filter	Filter	Filter	Filter	Filter	Filter	Filter	Filter	Filter	Filter	Filter	Filter	Filter	Filter	Filter												
A0A0G2K047	Acyl-CoA synthetase short-chain family member 3, mitochondrial OS=Rattus norvegicus OX=10116 GN=Acss3 PE=1 SV=1 - [ACSS3_RAT]	74.6	0.013	0.013	0.013	0.013	0.013	0.013	0.013	0.013	0.013	0.013	0.946	0.985	1.668	3.885	0.904	3.118	0.617	1.281	1.149	0.889	#DIV/0!	#DIV/0!	0.009	0.042	0.009	0.048	0.009	0.033	0.009	0.038	0.605	1.381
B0BNC9	Quinone oxidoreductase-like protein 2 OS=Rattus norvegicus OX=10116 PE=2 SV=1 - [QORL2_RAT]	37.5	0.013	0.013	0.013	0.013	0.013	0.013	0.013	0.013	0.013	0.013	1.556	1.357	1.938	3.283	0.984	4.461	0.462	1.651	1.514	1.408	#DIV/0!	#DIV/0!	0.009	0.036	0.009	0.031	0.009	0.028	0.009	0.024	0.917	0.940
B1H267	Sorting nexin-5 OS=Rattus norvegicus GN=Snx5 PE=1 SV=1 - [SNX5_RAT]	46.8	0.897	1.029	0.537	1.011	1.012	1.281	0.548	0.628	0.864	0.866	1.257	1.252	2.079	1.751	1.022	1.486	1.106	1.702	1.574	0.924	0.470	1.000	0.022	0.041	0.056	0.096	0.056	0.069	0.036	0.071	0.605	1.444
B2GV54	Neutral cholesterol ester hydrolase 1 OS=Rattus norvegicus OX=10116 GN=Nceh1 PE=1 SV=1 - [NCEH1_RAT]	45.8	0.013	0.013	0.013	0.013	0.013	0.013	0.013	0.013	0.013	0.013	1.120	1.052	2.102	1.185	0.871	1.189	1.123	2.112	1.874	1.238	#DIV/0!	#DIV/0!	0.009	0.068	0.009	0.042	0.009	0.060	0.009	0.033	0.127	0.770
B2RYD2	Epithelial splicing regulatory protein 1 OS=Rattus norvegicus OX=10116 GN=Esrp1 PE=2 SV=2 - [ESRP1_RAT]	75.0	0.013	0.013	0.013	0.013	0.013	1.607	0.430	0.729	1.389	1.272	1.411	1.655	2.491	1.256	1.454	0.013	0.013	0.013	0.013	0.013	0.009	0.055	0.009	0.042	#DIV/0!	#DIV/0!	0.127	0.115	0.009	0.067	0.009	0.081
B5DEQ3	Xaa-Pro aminopeptidase 3 OS=Rattus norvegicus OX=10116 GN=Xpnpep3 PE=1 SV=1 - [XPP3_RAT]	56.4	0.013	0.013	0.013	0.013	0.013	0.013	0.013	0.013	0.013	0.013	1.381	1.375	2.320	2.994	1.382	2.364	0.261	1.831	1.570	1.536	#DIV/0!	#DIV/0!	0.009	0.034	0.009	0.042	0.009	0.026	0.009	0.032	0.917	0.975
D3ZC96	Tetratricopeptide repeat protein 39B OS=Rattus norvegicus GN=Ttc39b PE=3 SV=1 - [TT39B_RAT]	70.1	0.013	0.013	0.013	0.013	0.013	1.116	0.467	0.353	0.787	1.151	1.278	1.161	2.422	1.157	0.773	1.567	0.986	1.762	1.388	1.495	0.009	0.263	0.009	0.061	0.009	0.046	0.056	0.074	0.036	0.070	0.354	1.471
D3ZLZ7	Inosine-5'-monophosphate dehydrogenase 1 OS=Rattus norvegicus GN=Impdh1 PE=3 SV=1 - [IMDH1_RAT]	55.3	0.898	1.134	0.925	1.194	1.279	0.013	0.013	0.013	0.013	0.013	1.372	1.295	2.358	1.234	1.212	0.840	0.583	1.802	1.350	0.834	0.009	0.055	0.036	0.055	0.605	0.540	0.009	0.044	0.009	0.069	0.259	1.331
D3ZVU1	SprT-like domain-containing protein Spartan OS=Rattus norvegicus OX=10116 GN=Sprtn PE=3 SV=1 - [SPRTN_RAT]	55.0	0.013	0.013	0.013	0.013	0.013	0.013	0.013	0.013	0.013	0.013	0.706	0.681	1.572	2.630	0.762	2.485	0.901	0.762	0.652	0.844	#DIV/0!	#DIV/0!	0.009	0.067	0.009	0.076	0.009	0.060	0.009	0.064	0.917	1.242
D4AAT7	ATP-dependent (S)-NAD(P)H-hydrate dehydratase OS=Rattus norvegicus GN=Carkd PE=3 SV=1 - [NNRD_RAT]	36.5	0.527	1.029	0.553	1.082	0.966	0.013	0.013	0.013	0.013	0.013	1.297	1.161	2.025	3.715	1.115	1.817	0.688	1.982	2.199	1.284	0.009	0.142	0.014	0.029	0.056	0.090	0.009	0.027	0.009	0.029	0.917	0.968
F1LU71	Methylglutaconyl-CoA hydratase, mitochondrial OS=Rattus norvegicus OX=10116 GN=Auh PE=1 SV=2 - [AUHM_RAT]	33.3	0.888	0.896	0.730	0.862	0.772	0.013	0.013	0.013	0.013	0.013	0.937	1.012	1.969	2.685	1.177	2.133	0.993	1.325	1.094	0.842	0.009	0.150	0.014	0.033	0.056	0.112	0.009	0.039	0.009	0.048	0.605	1.440
G3V7P1	Syntaxin-12 OS=Rattus norvegicus GN=Stx12 PE=1 SV=1 - [STX12_RAT]	31.2	0.013	0.013	0.013	0.013	0.013	0.013	0.013	0.013	0.013	0.013	2.354	1.482	2.882	2.859	1.520	2.504	0.818	2.646	2.460	1.530	#DIV/0!	#DIV/0!	0.009	0.029	0.009	0.030	0.009	0.023	0.009	0.023	0.756	1.102
O08839	Myc box-dependent-interacting protein 1 OS=Rattus norvegicus GN=Bin1 PE=1 SV=1 - [BIN1_RAT]	64.5	0.013	0.013	0.013	0.013	0.013	0.013	0.013	0.013	0.013	0.013	1.669	1.537	3.113	1.905	1.203	0.945	0.985	1.208	1.122	1.219	#DIV/0!	#DIV/0!	0.009	0.034	0.009	0.080	0.009	0.027	0.009	0.065	0.036	0.230
O09171	Betaine--homocysteine S-methyltransferase 1 OS=Rattus norvegicus OX=10116 GN=Bhmt PE=1 SV=1 - [BHMT1_RAT]	44.9	0.013	0.013	0.013	0.013	0.013	0.013	0.013	0.013	0.013	0.013	1.036	1.203	2.043	4.002	0.998	4.113	0.363	1.522	1.388	0.983	#DIV/0!	#DIV/0!	0.009	0.035	0.009	0.035	0.009	0.027	0.009	0.027	0.756	1.134
O35077	Glycerol-3-phosphate dehydrogenase [NAD(+)], cytoplasmic OS=Rattus norvegicus GN=Gpd1 PE=1 SV=4 - [GPDA_RAT]	37.4	0.638	0.481	0.409	0.709	0.659	0.405	0.140	0.258	0.410	0.401	0.695	0.787	1.357	5.465	0.739	4.578	0.538	1.051	0.931	0.735	0.022	0.124	0.022	0.041	0.056	0.106	0.014	0.032	0.014	0.033	0.756	1.151
O35078	D-amino-acid oxidase OS=Rattus norvegicus GN=Dao PE=2 SV=1 - [OXDA_RAT]	38.8	0.098	0.210	0.134	0.188	0.190	0.138	0.116	0.135	0.178	0.191	0.690	0.808	1.298	5.903	0.672	5.573	0.424	1.091	0.989	0.737	0.756	1.141	0.014	0.040	0.014	0.042	0.014	0.032	0.014	0.033	0.917	0.948
O55012	Phosphatidylinositol-binding clathrin assembly protein OS=Rattus norvegicus GN=Picalm PE=1 SV=1 - [PICAL_RAT]	69.2	0.320	1.017	0.120	1.022	0.957	0.013	0.013	0.013	0.013	0.013	1.096	1.252	1.870	4.784	1.044	4.305	0.506	1.517	1.474	1.233	0.009	0.674	0.014	0.029	0.056	0.089	0.009	0.025	0.009	0.024	0.917	0.936
O55159	Epithelial cell adhesion molecule OS=Rattus norvegicus OX=10116 GN=Epcam PE=1 SV=1 - [EPCAM_RAT]	35.2	0.013	0.013	0.013	0.013	0.013	0.013	0.013	0.013	0.013	0.013	1.382	1.124	2.213	3.837	0.615	3.590	0.365	1.736	1.529	1.178	#DIV/0!	#DIV/0!	0.009	0.035	0.009	0.035	0.009	0.028	0.009	0.027	0.917	0.962
O70196	Prolyl endopeptidase OS=Rattus norvegicus GN=Prep PE=1 SV=1 - [PPCE_RAT]	80.7	0.768	0.894	0.597	0.944	0.905	1.052	0.547	0.716	0.825	1.021	1.071	1.083	2.080	1.742	1.169	1.448	0.842	1.290	1.235	1.179	0.917	0.990	0.014	0.036	0.056	0.121	0.014	0.030	0.036	0.074	0.917	1.170
O70199	UDP-glucose 6-dehydrogenase OS=Rattus norvegicus GN=Ugdh PE=1 SV=1 - [UGDH_RAT]	54.9	0.013	0.013	0.013	0.013	0.013	0.013	0.013	0.013	0.013	0.013	1.056	1.021	2.094	3.891	0.844	3.227	0.570	1.243	1.266	1.070	#DIV/0!	#DIV/0!	0.009	0.037	0.009	0.044	0.009	0.029	0.009	0.034	0.917	1.003
O70244	Cubilin OS=Rattus norvegicus OX=10116 GN=Cubn PE=1 SV=2 - [CUBN_RAT]	398.7	0.013	0.013	0.013	0.013	0.013	0.013	0.013	0.013	0.013	0.013	0.702	0.677	1.314	2.803	0.821	2.089	0.785	0.959	0.922	0.672	#DIV/0!	#DIV/0!	0.009	0.069	0.009	0.083	0.009	0.061	0.009	0.067	0.917	1.252
O70257	Syntaxin-7 OS=Rattus norvegicus GN=Stx7 PE=1 SV=4 - [STX7_RAT]	29.8	0.013	0.013	0.013	0.013	0.013	0.013	0.013	0.013	0.013	0.013	1.833	1.215	2.765	2.366	1.053	2.163	0.481	1.850	1.788	1.259	#DIV/0!	#DIV/0!	0.009	0.035	0.009	0.042	0.009	0.027	0.009	0.033	0.605	1.335
O88339	Epsin-1 OS=Rattus norvegicus GN=Epn1 PE=1 SV=1 - [EPN1_RAT]	60.1	0.013	0.013	0.013	0.013	0.013	0.013	0.013	0.013	0.013	0.013	0.940	1.038	1.989	1.817	1.140	1.405	1.231	0.835	0.773	0.772	#DIV/0!	#DIV/0!	0.009	0.060	0.009	0.111	0.009	0.051	0.009	0.093	0.184	1.062
O88656	Actin-related protein 2/3 complex subunit 1B OS=Rattus norvegicus GN=Arpc1b PE=2 SV=3 - [ARC1B_RAT]	41.0	0.013	0.013	0.013	0.013	0.013	0.013	0.013	0.013	0.013	0.013	0.777	1.034	1.669	1.583	1.138	1.479	0.926	1.071	0.869	0.996	#DIV/0!	#DIV/0!	0.009	0.071	0.009	0.085	0.009	0.062	0.009	0.072	0.354	1.635
O88797	Disabled homolog 2 OS=Rattus norvegicus OX=10116 GN=Dab2 PE=1 SV=1 - [DAB2_RAT]	82.3	0.123	0.384	0.171	0.412	0.356	0.337	0.184	0.241	0.443	0.490	0.615	0.587	0.934	6.799	0.535	5.351	0.511	0.882	0.763	0.728	0.470	1.147	0.014	0.038	0.014	0.042	0.014	0.031	0.014	0.033	0.917	0.958
P00388	NADPH--cytochrome P450 reductase OS=Rattus norvegicus OX=10116 GN=Por PE=1 SV=3 - [NCPR_RAT]	76.9	1.126	0.622	0.718	0.802	0.674	0.013	0.013	0.013	0.013	0.013	1.204	1.164	1.772	2.336	1.286	1.772	0.640	1.307	1.410	1.006	0.009	0.225	0.014	0.034	0.127	0.170	0.009	0.039	0.009	0.052	0.605	1.461
P00502	Glutathione S-transferase alpha-1 OS=Rattus norvegicus GN=Gsta1 PE=1 SV=3 - [GSTA1_RAT]	25.6	0.148	0.230	0.162	0.254	0.231	0.134	0.073	0.094	0.173	0.173	0.460	0.473	0.728	11.267	0.422	9.854	0.167	0.685	0.609	0.450	0.085	0.441	0.014	0.028	0.056	0.089	0.014	0.025	0.036	0.068	0.756	1.093
P00697	Lysozyme C-1 OS=Rattus norvegicus GN=Lyz1 PE=1 SV=2 - [LYSC1_RAT]	16.7	0.102	0.185	0.099	0.285	0.259	0.214	0.119	0.194	0.295	0.368	0.624	0.554	1.044	12.953	0.515	8.780	0.291	0.583	0.586	0.507	0.259	0.968	0.014	0.028	0.014	0.039	0.014	0.025	0.036	0.069	0.354	1.391
P00786	Pro-cathepsin H OS=Rattus norvegicus GN=Ctsh PE=1 SV=1 - [CATH_RAT]	37.1	0.347	0.288	0.302	0.383	0.376	0.488	0.279	0.387	0.313	0.455	0.459	0.482	0.752	8.046	0.488	6.904	0.511	0.624	0.701	0.565	0.354	1.105	0.014	0.033	0.014	0.039	0.036	0.051	0.014	0.032	0.470	1.379
P00884	Fructose-bisphosphate aldolase B OS=Rattus norvegicus GN=Aldob PE=1 SV=2 - [ALDOB_RAT]	39.6	0.347	0.477	0.316	0.555	0.507	0.646	0.306	0.407	0.462	0.507	0.541	0.617	0.866	6.571	0.535	5.913	0.536	0.860	0.748	0.595	0.917	1.083	0.036	0.059	0.022	0.059	0.056	0.069	0.036	0.071	0.917	0.956
P01946	Hemoglobin subunit alpha-1/2 OS=Rattus norvegicus GN=Hba1 PE=1 SV=3 - [HBA_RAT]	15.3	0.702	0.478	0.477	0.682	0.605	0.783	0.295	0.759	0.521	0.451	0.827	0.647	1.899	2.525	0.732	3.653	0.567	1.302	0.980	0.991	0.917	1.075	0.036	0.061	0.056	0.115	0.085	0.097	0.036	0.074	0.756	1.295
P02761	Major urinary protein OS=Rattus norvegicus OX=10116 PE=1 SV=1 - [MUP_RAT]	20.7	0.013	0.013	0.013	0.013	0.013	0.013	0.013	0.013	0.013	0.013	0.257	0.339	0.589	4.691	0.200	7.258	0.406	0.801	0.747	0.813	#DIV/0!	#DIV/0!	0.009	0.073	0.009	0.030	0.009	0.065	0.009	0.023	0.127	0.748
P04176	Phenylalanine-4-hydroxylase OS=Rattus norvegicus OX=10116 GN=Pah PE=1 SV=3 - [PH4H_RAT]	51.8	0.013	0.013	0.013	0.013	0.013	0.013	0.013	0.013	0.013	0.013	0.767	0.851	1.436	5.840	0.794	6.083	0.490	1.032	0.989	0.835	#DIV/0!	#DIV/0!	0.009	0.033	0.009	0.031	0.009	0.026	0.009	0.024	0.917	0.935
P04182	Ornithine aminotransferase, mitochondrial OS=Rattus norvegicus GN=Oat PE=1 SV=1 - [OAT_RAT]	48.3	0.137	0.343	0.141	0.329	0.317	0.293	0.147	0.207	0.350	0.345	0.531	0.585	0.861	8.292	0.515	8.927	0.458	0.852	0.743	0.681	0.470	1.149	0.014	0.032	0.014	0.039	0.014	0.028	0.014	0.032	0.756	1.098
P04256	Heterogeneous nuclear ribonucleoprotein A1 OS=Rattus norvegicus GN=Hnrnpa1 PE=1 SV=3 - [ROA1_RAT]	34.2	0.570	1.138	0.423	0.922	0.996	0.939	0.422	0.639	1.029	1.062	1.093	1.011	2.292	1.841	1.194	1.388	0.973	1.284	1.327	1.232	0.917	0.998	0.036	0.058	0.036	0.087	0.036	0.053	0.036	0.074	0.917	1.134
P04797	Glyceraldehyde-3-phosphate dehydrogenase OS=Rattus norvegicus GN=Gapdh PE=1 SV=3 - [G3P_RAT]	35.8	0.590	0.737	0.401	0.806	0.784	0.611	0.286	0.470	0.809	0.492	0.957	1.002	1.882	4.859	0.793	4.921	0.501	1.445	1.262	0.916	0.470	1.128	0.022	0.040	0.085	0.118	0.022	0.038	0.036	0.069	0.917	0.942
P04903	Glutathione S-transferase alpha-2 OS=Rattus norvegicus GN=Gsta2 PE=2 SV=2 - [GSTA2_RAT]	25.5	0.013	0.013	0.013	0.013	0.013	0.013	0.013	0.013	0.013	0.013	0.362	0.451	0.814	4.647	0.439	5.197	0.568	1.119	0.841	0.636	#DIV/0!	#DIV/0!	0.009	0.062	0.009	0.035	0.009	0.053	0.009	0.027	0.184	1.038
P04916	Retinol-binding protein 4 OS=Rattus norvegicus GN=Rbp4 PE=1 SV=1 - [RET4_RAT]	23.2	0.247	0.326	0.218	0.291	0.333	0.369	0.288	0.243	0.339	0.360	0.600	0.626	1.102	7.377	0.545	8.092	0.515	0.908	0.787	0.789	0.259	0.965	0.014	0.034	0.014	0.039	0.014	0.029	0.014	0.032	0.756	1.101
P05545	Serine protease inhibitor A3K OS=Rattus norvegicus GN=Serpina3k PE=1 SV=3 - [SPA3K_RAT]	46.5	0.248	0.837	0.207	0.789	0.674	0.452	0.218	0.347	0.834	0.815	1.364	0.915	2.565	1.835	0.969	2.698	0.519	1.907	0.912	2.121	0.917	1.079	0.014	0.039	0.056	0.102	0.014	0.032	0.036	0.071	0.917	1.019
P05708	Hexokinase-1 OS=Rattus norvegicus GN=Hk1 PE=1 SV=4 - [HXK1_RAT]	102.3	0.712	0.991	0.630	0.885	0.881	0.959	0.651	0.699	0.782	0.942	0.918	1.053	2.027	1.525	1.072	1.669	0.858	1.307	1.106	1.059	0.917	0.999	0.022	0.044	0.056	0.121	0.036	0.056	0.036	0.075	0.917	1.214
P07150	Annexin A1 OS=Rattus norvegicus GN=Anxa1 PE=1 SV=2 - [ANXA1_RAT]	38.8	0.500	0.899	0.375	0.817	0.834	0.623	0.339	0.498	0.823	0.896	0.953	0.947	2.134	2.169	1.180	1.973	0.781	1.363	1.258	1.396	0.605	1.121	0.014	0.038	0.056	0.120	0.014	0.032	0.036	0.076	0.917	1.099
P07153	Dolichyl-diphosphooligosaccharide--protein glycosyltransferase subunit 1 OS=Rattus norvegicus GN=Rpn1 PE=2 SV=1 - [RPN1_RAT]	68.3	0.982	0.887	0.672	1.215	1.144	0.013	0.013	0.013	0.013	0.013	1.346	1.083	2.825	1.319	1.332	1.429	0.962	1.209	1.412	0.851	0.009	0.065	0.036	0.056	0.354	0.353	0.009	0.038	0.009	0.057	0.470	1.517
P07171	Calbindin OS=Rattus norvegicus GN=Calb1 PE=1 SV=2 - [CALB1_RAT]	30.0	0.120	0.178	0.120	0.162	0.175	0.173	0.211	0.149	0.171	0.197	0.549	0.547	0.898	7.796	0.504	6.915	0.403	0.866	0.812	0.612	0.259	0.971	0.014	0.037	0.014	0.040	0.014	0.031	0.014	0.033	0.917	0.927
P07340	Sodium/potassium-transporting ATPase subunit beta-1 OS=Rattus norvegicus OX=10116 GN=Atp1b1 PE=1 SV=1 - [AT1B1_RAT]	35.2	0.496	0.582	0.332	0.603	0.534	0.013	0.013	0.013	0.013	0.013	0.628	0.676	1.031	5.528	0.632	5.476	0.454	1.007	0.910	0.599	0.009	1.796	0.014	0.037	0.127	0.165	0.009	0.032	0.009	0.027	0.605	1.320
P07379	Phosphoenolpyruvate carboxykinase, cytosolic [GTP] OS=Rattus norvegicus OX=10116 GN=Pck1 PE=1 SV=1 - [PCKGC_RAT]	69.4	0.164	0.296	0.128	0.253	0.238	0.270	0.145	0.201	0.369	0.340	0.561	0.666	1.084	6.852	0.618	6.337	0.420	0.952	0.931	0.824	0.354	1.113	0.014	0.038	0.014	0.039	0.014	0.031	0.014	0.032	0.917	0.934
P07687	Epoxide hydrolase 1 OS=Rattus norvegicus OX=10116 GN=Ephx1 PE=1 SV=1 - [HYEP_RAT]	52.5	0.013	0.013	0.013	0.013	0.013	0.013	0.013	0.013	0.013	0.013	1.071	0.982	1.645	2.480	0.811	2.486	0.782	1.394	1.119	1.084	#DIV/0!	#DIV/0!	0.009	0.058	0.009	0.051	0.009	0.049	0.009	0.041	0.756	1.315
P07861	Neprilysin OS=Rattus norvegicus OX=10116 GN=Mme PE=1 SV=2 - [NEP_RAT]	85.7	0.013	0.013	0.013	0.013	0.013	0.013	0.013	0.013	0.013	0.013	1.022	0.748	1.612	4.467	0.825	4.464	0.541	1.476	1.293	1.325	#DIV/0!	#DIV/0!	0.009	0.040	0.009	0.032	0.009	0.031	0.009	0.024	0.917	0.957
P08289	Alkaline phosphatase, tissue-nonspecific isozyme OS=Rattus norvegicus OX=10116 GN=Alpl PE=1 SV=2 - [PPBT_RAT]	57.6	0.013	0.013	0.013	0.013	0.013	0.013	0.013	0.013	0.013	0.013	0.727	0.530	0.978	6.187	0.287	6.665	0.260	1.134	0.848	0.720	#DIV/0!	#DIV/0!	0.009	0.039	0.009	0.030	0.009	0.031	0.009	0.023	0.756	1.121
P08424	Renin OS=Rattus norvegicus OX=10116 GN=Ren1 PE=1 SV=2 - [RENI_RAT]	44.2	0.013	0.013	0.013	0.013	0.013	0.013	0.013	0.013	0.013	0.013	1.071	0.793	1.496	4.949	0.767	3.988	0.787	0.922	0.952	0.881	#DIV/0!	#DIV/0!	0.009	0.036	0.009	0.043	0.009	0.028	0.009	0.033	0.756	1.158
P08430	UDP-glucuronosyltransferase 1-6 OS=Rattus norvegicus OX=10116 GN=Ugt1a6 PE=1 SV=1 - [UD16_RAT]	60.1	0.013	0.013	0.013	0.013	0.013	0.013	0.013	0.013	0.013	0.013	1.114	0.977	1.987	4.502	1.111	2.537	0.659	1.133	1.407	1.033	#DIV/0!	#DIV/0!	0.009	0.033	0.009	0.054	0.009	0.025	0.009	0.043	0.756	1.167
P08699	Galectin-3 OS=Rattus norvegicus GN=Lgals3 PE=1 SV=4 - [LEG3_RAT]	27.2	0.762	0.752	0.475	0.928	0.826	0.972	0.601	0.652	0.676	0.804	0.862	0.933	2.020	1.941	1.049	1.477	0.940	1.310	1.082	0.988	0.756	1.088	0.022	0.044	0.014	0.042	0.036	0.056	0.022	0.052	0.917	1.213
P09034	Argininosuccinate synthase OS=Rattus norvegicus GN=Ass1 PE=2 SV=1 - [ASSY_RAT]	46.5	0.275	0.220	0.239	0.282	0.248	0.325	0.246	0.204	0.437	0.447	0.604	0.644	0.945	7.083	0.592	6.321	0.482	0.837	0.835	0.650	0.354	1.108	0.014	0.037	0.014	0.039	0.014	0.030	0.014	0.032	0.917	0.938
P09606	Glutamine synthetase OS=Rattus norvegicus GN=Glul PE=1 SV=3 - [GLNA_RAT]	42.2	0.013	0.013	0.013	0.013	0.013	0.013	0.013	0.013	0.013	0.013	0.828	0.651	1.366	3.891	0.752	3.040	0.689	1.079	0.840	0.862	#DIV/0!	#DIV/0!	0.009	0.051	0.009	0.057	0.009	0.043	0.009	0.046	0.756	1.301
P09811	Glycogen phosphorylase, liver form OS=Rattus norvegicus GN=Pygl PE=1 SV=5 - [PYGL_RAT]	97.4	0.013	0.013	0.013	0.013	0.013	0.013	0.013	0.013	0.013	0.013	1.227	1.121	2.013	4.016	1.166	5.026	0.575	1.532	1.586	1.154	#DIV/0!	#DIV/0!	0.009	0.033	0.009	0.030	0.009	0.026	0.009	0.023	0.917	0.930
P0C2X9	Delta-1-pyrroline-5-carboxylate dehydrogenase, mitochondrial OS=Rattus norvegicus GN=Aldh4a1 PE=1 SV=1 - [AL4A1_RAT]	61.8	0.415	0.565	0.382	0.614	0.531	0.548	0.320	0.342	0.646	0.636	0.722	0.780	1.178	7.632	0.646	4.880	0.591	1.035	0.998	0.885	0.917	1.081	0.014	0.029	0.022	0.059	0.014	0.025	0.036	0.071	0.917	0.931
P10687	1-phosphatidylinositol 4,5-bisphosphate phosphodiesterase beta-1 OS=Rattus norvegicus GN=Plcb1 PE=1 SV=1 - [PLCB1_RAT]	138.3	0.414	1.089	0.460	1.011	0.759	0.013	0.013	0.013	0.013	0.013	1.040	1.023	2.080	2.129	1.135	1.803	0.362	2.081	1.637	1.513	0.009	0.327	0.036	0.059	0.127	0.165	0.009	0.044	0.009	0.034	0.917	1.064
P11980	Pyruvate kinase PKM OS=Rattus norvegicus GN=Pkm PE=1 SV=3 - [KPYM_RAT]	57.8	0.645	0.904	0.599	0.952	0.866	1.105	0.491	0.802	0.854	0.848	0.952	1.056	2.106	1.719	1.138	1.751	0.864	1.374	1.283	1.240	0.756	1.036	0.022	0.044	0.056	0.113	0.036	0.055	0.022	0.051	0.917	1.146
P12007	Isovaleryl-CoA dehydrogenase, mitochondrial OS=Rattus norvegicus GN=Ivd PE=1 SV=2 - [IVD_RAT]	46.4	0.781	0.824	0.663	0.912	0.787	1.243	0.647	0.749	0.851	0.809	0.918	1.094	1.817	1.702	1.083	1.644	0.894	1.490	1.379	0.934	0.917	0.991	0.014	0.039	0.022	0.060	0.056	0.073	0.036	0.072	0.605	1.528
P13084	Nucleophosmin OS=Rattus norvegicus GN=Npm1 PE=1 SV=1 - [NPM_RAT]	32.5	0.493	0.884	0.331	1.050	0.877	0.804	0.389	0.392	1.092	1.061	1.243	1.048	2.543	1.388	1.049	1.800	0.812	1.688	1.465	1.259	0.756	1.093	0.036	0.060	0.056	0.109	0.085	0.092	0.036	0.072	0.605	1.428
P13697	NADP-dependent malic enzyme OS=Rattus norvegicus GN=Me1 PE=1 SV=2 - [MAOX_RAT]	64.0	0.013	0.013	0.013	0.013	0.013	0.013	0.013	0.013	0.013	0.013	0.779	0.927	1.605	3.990	0.940	4.143	0.536	1.660	1.241	1.045	#DIV/0!	#DIV/0!	0.009	0.042	0.009	0.034	0.009	0.034	0.009	0.026	0.605	1.328
P14272	Plasma kallikrein OS=Rattus norvegicus GN=Klkb1 PE=1 SV=1 - [KLKB1_RAT]	71.2	0.013	0.013	0.013	0.013	0.013	0.013	0.013	0.013	0.013	0.013	0.968	0.803	1.594	2.468	1.009	2.302	0.504	1.455	1.156	1.601	#DIV/0!	#DIV/0!	0.009	0.061	0.009	0.049	0.009	0.052	0.009	0.039	0.756	1.314
P14740	Dipeptidyl peptidase 4 OS=Rattus norvegicus GN=Dpp4 PE=1 SV=2 - [DPP4_RAT]	88.0	0.521	0.381	0.408	0.526	0.497	0.406	0.247	0.270	0.220	0.238	0.605	0.621	0.999	6.338	0.576	5.657	0.485	0.861	0.825	0.689	0.022	0.124	0.014	0.035	0.056	0.103	0.014	0.032	0.014	0.033	0.917	0.961
P15684	Aminopeptidase N OS=Rattus norvegicus OX=10116 GN=Anpep PE=1 SV=2 - [AMPN_RAT]	109.4	0.013	0.013	0.013	0.013	0.013	0.013	0.013	0.013	0.013	0.013	0.883	0.867	1.589	4.308	0.751	4.306	0.495	1.322	1.204	0.913	#DIV/0!	#DIV/0!	0.009	0.041	0.009	0.036	0.009	0.033	0.009	0.027	0.917	0.989
P17136	Small nuclear ribonucleoprotein-associated protein B OS=Rattus norvegicus OX=10116 GN=Snrpb PE=2 SV=2 - [RSMB_RAT]	23.6	0.013	0.013	0.013	0.013	0.013	0.013	0.013	0.013	0.013	0.013	1.486	1.277	2.674	1.496	1.167	1.722	0.856	1.733	1.753	1.141	#DIV/0!	#DIV/0!	0.009	0.044	0.009	0.046	0.009	0.035	0.009	0.036	0.917	1.039
P17164	Tissue alpha-L-fucosidase OS=Rattus norvegicus GN=Fuca1 PE=1 SV=1 - [FUCO_RAT]	53.5	0.013	0.013	0.013	0.013	0.013	0.013	0.013	0.013	0.013	0.013	1.058	0.870	2.105	5.776	0.902	4.302	0.381	1.142	1.186	0.897	#DIV/0!	#DIV/0!	0.009	0.030	0.009	0.038	0.009	0.024	0.009	0.029	0.756	1.117
P18484	AP-2 complex subunit alpha-2 OS=Rattus norvegicus GN=Ap2a2 PE=1 SV=3 - [AP2A2_RAT]	104.0	0.013	0.013	0.013	0.013	0.013	0.013	0.013	0.013	0.013	0.013	1.254	1.259	2.325	2.863	1.117	2.157	0.721	2.009	2.502	1.568	#DIV/0!	#DIV/0!	0.009	0.038	0.009	0.032	0.009	0.030	0.009	0.025	0.917	0.956
P19139	Casein kinase II subunit alpha OS=Rattus norvegicus GN=Csnk2a1 PE=1 SV=2 - [CSK21_RAT]	45.0	0.013	0.013	0.013	0.013	0.013	0.013	0.013	0.013	0.013	0.013	1.277	1.242	2.693	2.957	1.139	2.218	0.574	1.431	1.527	1.317	#DIV/0!	#DIV/0!	0.009	0.035	0.009	0.048	0.009	0.027	0.009	0.038	0.917	1.000
P19468	Glutamate--cysteine ligase catalytic subunit OS=Rattus norvegicus GN=Gclc PE=1 SV=2 - [GSH1_RAT]	72.6	0.292	0.411	0.285	0.492	0.474	0.473	0.315	0.338	0.357	0.402	0.605	0.632	1.097	6.546	0.577	6.923	0.489	0.995	0.888	0.744	0.756	1.139	0.014	0.036	0.022	0.058	0.014	0.030	0.014	0.032	0.756	1.109
P20816	Cytochrome P450 4A2 OS=Rattus norvegicus OX=10116 GN=Cyp4a2 PE=1 SV=2 - [CP4A2_RAT]	57.9	0.013	0.013	0.013	0.013	0.013	0.013	0.013	0.013	0.013	0.013	0.845	0.724	1.440	5.126	0.757	7.108	0.585	1.203	1.058	0.905	#DIV/0!	#DIV/0!	0.009	0.038	0.009	0.029	0.009	0.030	0.009	0.022	0.756	1.107
P21139	Alpha-mannosidase 2C1 OS=Rattus norvegicus GN=Man2c1 PE=1 SV=1 - [MA2C1_RAT]	115.9	0.013	0.013	0.013	0.013	0.013	0.013	0.013	0.013	0.013	0.013	1.405	1.383	1.980	3.193	1.054	2.345	1.023	2.027	1.655	1.270	#DIV/0!	#DIV/0!	0.009	0.037	0.009	0.036	0.009	0.029	0.009	0.027	0.917	0.968
P23680	Serum amyloid P-component OS=Rattus norvegicus GN=Apcs PE=2 SV=2 - [SAMP_RAT]	26.2	0.013	0.013	0.013	0.013	0.013	0.013	0.013	0.013	0.013	0.013	0.769	0.766	1.558	1.930	0.842	1.405	1.521	0.705	0.689	0.677	#DIV/0!	#DIV/0!	0.009	0.079	0.009	0.115	0.009	0.069	0.009	0.094	0.184	1.067
P24090	Alpha-2-HS-glycoprotein OS=Rattus norvegicus GN=Ahsg PE=1 SV=2 - [FETUA_RAT]	38.0	0.781	0.687	0.506	0.882	0.822	0.696	0.498	0.712	1.056	0.823	1.335	0.877	2.758	2.704	0.927	3.167	0.768	1.611	1.233	1.547	0.917	1.043	0.022	0.041	0.056	0.091	0.036	0.051	0.036	0.069	0.917	0.979
P24268	Cathepsin D OS=Rattus norvegicus GN=Ctsd PE=1 SV=1 - [CATD_RAT]	44.7	0.727	0.923	0.558	0.940	0.842	1.136	0.432	0.679	0.658	0.826	1.108	1.090	2.126	2.080	0.933	2.072	0.833	1.340	1.194	0.918	0.470	1.083	0.022	0.043	0.127	0.169	0.056	0.072	0.036	0.075	0.470	1.520
P24470	Cytochrome P450 2C23 OS=Rattus norvegicus GN=Cyp2c23 PE=2 SV=2 - [CP2CN_RAT]	56.4	0.013	0.013	0.013	0.013	0.013	0.013	0.013	0.013	0.013	0.013	0.708	0.711	1.247	6.973	0.669	6.026	0.518	1.018	0.956	0.707	#DIV/0!	#DIV/0!	0.009	0.031	0.009	0.032	0.009	0.024	0.009	0.024	0.756	1.108
P24473	Glutathione S-transferase kappa 1 OS=Rattus norvegicus OX=10116 GN=Gstk1 PE=1 SV=3 - [GSTK1_RAT]	25.5	0.013	0.013	0.013	0.013	0.013	0.013	0.013	0.013	0.013	0.013	0.923	0.749	1.276	4.104	0.571	3.763	0.647	1.291	1.245	0.629	#DIV/0!	#DIV/0!	0.009	0.050	0.009	0.041	0.009	0.042	0.009	0.032	0.917	1.044
P25235	Dolichyl-diphosphooligosaccharide--protein glycosyltransferase subunit 2 OS=Rattus norvegicus OX=10116 GN=Rpn2 PE=2 SV=2 - [RPN2_RAT]	69.0	2.018	1.854	1.501	1.606	1.954	1.035	0.821	0.786	0.867	0.769	1.160	1.069	1.271	1.651	1.002	0.889	1.191	1.068	1.379	0.908	0.014	0.076	0.036	0.054	0.014	0.038	0.022	0.040	0.036	0.077	0.354	1.630
P25809	Creatine kinase U-type, mitochondrial OS=Rattus norvegicus GN=Ckmt1 PE=1 SV=1 - [KCRU_RAT]	47.0	0.013	0.013	0.013	0.013	0.013	0.013	0.013	0.013	0.013	0.013	0.801	0.843	1.219	4.861	0.869	4.167	0.414	1.046	0.883	0.737	#DIV/0!	#DIV/0!	0.009	0.041	0.009	0.045	0.009	0.032	0.009	0.035	0.605	1.364
P26453	Basigin OS=Rattus norvegicus GN=Bsg PE=1 SV=2 - [BASI_RAT]	42.4	0.767	1.000	0.471	0.996	0.799	0.013	0.013	0.013	0.013	0.013	1.198	1.212	2.034	2.761	0.841	2.994	0.708	1.154	1.185	0.974	0.009	0.186	0.036	0.057	0.184	0.214	0.009	0.036	0.009	0.039	0.354	1.446
P29457	Serpin H1 OS=Rattus norvegicus GN=Serpinh1 PE=1 SV=1 - [SERPH_RAT]	46.5	0.013	0.013	0.013	0.013	0.013	0.013	0.013	0.013	0.013	0.013	0.821	0.870	2.076	3.210	1.029	3.207	0.752	1.503	1.469	0.887	#DIV/0!	#DIV/0!	0.009	0.046	0.009	0.039	0.009	0.037	0.009	0.030	0.917	1.017
P32755	4-hydroxyphenylpyruvate dioxygenase OS=Rattus norvegicus OX=10116 GN=Hpd PE=1 SV=3 - [HPPD_RAT]	45.1	0.229	0.239	0.222	0.377	0.310	0.013	0.013	0.013	0.013	0.013	0.571	0.601	0.920	7.322	0.614	6.014	0.428	0.881	0.794	0.644	0.009	5.389	0.014	0.036	0.014	0.040	0.009	0.025	0.009	0.025	0.917	0.938
P34064	Proteasome subunit alpha type-5 OS=Rattus norvegicus GN=Psma5 PE=2 SV=1 - [PSA5_RAT]	26.4	0.552	0.861	0.535	0.948	0.867	1.021	0.405	0.616	0.862	0.930	0.935	1.115	1.974	1.714	1.228	1.673	0.908	1.359	1.211	1.281	0.756	1.084	0.022	0.044	0.022	0.060	0.022	0.040	0.036	0.074	0.756	1.348
P35213	14-3-3 protein beta/alpha OS=Rattus norvegicus GN=Ywhab PE=1 SV=3 - [1433B_RAT]	28.0	0.605	1.011	0.443	1.101	0.982	1.040	0.577	0.555	0.729	0.784	1.151	1.013	2.035	2.033	1.326	1.667	0.794	1.554	1.360	1.177	0.605	1.066	0.022	0.042	0.056	0.107	0.022	0.039	0.022	0.051	0.756	1.296
P35467	Protein S100-A1 OS=Rattus norvegicus OX=10116 GN=S100a1 PE=1 SV=3 - [S10A1_RAT]	10.6	0.013	0.013	0.013	0.013	0.013	0.013	0.013	0.013	0.013	0.013	0.909	0.977	1.849	3.184	1.166	4.048	0.274	1.499	1.294	1.376	#DIV/0!	#DIV/0!	0.009	0.044	0.009	0.035	0.009	0.036	0.009	0.026	0.756	1.162
P35859	Insulin-like growth factor-binding protein complex acid labile subunit OS=Rattus norvegicus GN=Igfals PE=1 SV=1 - [ALS_RAT]	66.8	0.013	0.013	0.013	0.013	0.013	0.013	0.013	0.013	0.013	0.013	2.091	2.026	3.997	3.100	1.495	2.726	0.718	1.662	1.523	1.890	#DIV/0!	#DIV/0!	0.009	0.027	0.009	0.034	0.009	0.022	0.009	0.026	0.184	1.006
P36365	Dimethylaniline monooxygenase [N-oxide-forming] 1 OS=Rattus norvegicus OX=10116 GN=Fmo1 PE=1 SV=2 - [FMO1_RAT]	59.8	0.013	0.013	0.013	0.013	0.013	0.013	0.013	0.013	0.013	0.013	0.637	0.713	0.957	5.501	0.605	4.366	0.580	0.994	0.881	0.676	#DIV/0!	#DIV/0!	0.009	0.041	0.009	0.043	0.009	0.033	0.009	0.033	0.917	1.015
P36860	Ras-related protein Ral-B OS=Rattus norvegicus GN=Ralb PE=2 SV=1 - [RALB_RAT]	23.3	0.013	0.013	0.013	0.013	0.013	0.013	0.013	0.013	0.013	0.013	1.410	1.204	2.327	4.092	0.724	3.308	0.345	1.764	1.655	0.926	#DIV/0!	#DIV/0!	0.009	0.032	0.009	0.038	0.009	0.025	0.009	0.029	0.756	1.132
P36953	Afamin OS=Rattus norvegicus GN=Afm PE=3 SV=1 - [AFAM_RAT]	69.3	0.576	0.823	0.389	0.912	0.797	0.656	0.416	0.620	0.882	0.801	0.974	0.937	2.077	1.982	0.862	2.019	0.669	1.210	1.039	1.214	0.917	1.062	0.022	0.044	0.056	0.127	0.022	0.040	0.036	0.078	0.917	1.188
P37397	Calponin-3 OS=Rattus norvegicus GN=Cnn3 PE=1 SV=1 - [CNN3_RAT]	36.4	0.489	0.884	0.478	0.858	0.955	0.013	0.013	0.013	0.013	0.013	1.100	0.936	2.049	2.656	1.095	2.919	0.761	1.462	1.665	1.180	0.009	0.385	0.022	0.042	0.056	0.092	0.009	0.039	0.009	0.029	0.756	1.197
P38918	Aflatoxin B1 aldehyde reductase member 3 OS=Rattus norvegicus OX=10116 GN=Akr7a3 PE=1 SV=2 - [ARK73_RAT]	36.7	0.354	0.520	0.318	0.634	0.528	0.729	0.397	0.470	0.544	0.478	0.618	0.653	1.245	6.246	0.649	5.803	0.506	0.935	0.940	0.897	0.605	1.151	0.022	0.042	0.056	0.095	0.056	0.067	0.036	0.070	0.917	0.944
P42123	L-lactate dehydrogenase B chain OS=Rattus norvegicus GN=Ldhb PE=1 SV=2 - [LDHB_RAT]	36.6	0.205	0.371	0.184	0.367	0.367	0.292	0.167	0.243	0.297	0.303	0.556	0.599	0.829	9.142	0.483	7.448	0.413	0.779	0.694	0.611	0.354	1.110	0.014	0.029	0.014	0.039	0.014	0.026	0.014	0.032	0.917	0.921
P42930	Heat shock protein beta-1 OS=Rattus norvegicus GN=Hspb1 PE=1 SV=1 - [HSPB1_RAT]	22.9	0.013	0.013	0.013	0.013	0.013	0.013	0.013	0.013	0.013	0.013	0.860	0.922	1.722	3.430	1.103	2.566	0.888	1.271	1.225	1.126	#DIV/0!	#DIV/0!	0.009	0.045	0.009	0.047	0.009	0.036	0.009	0.037	0.756	1.228
P45479	Palmitoyl-protein thioesterase 1 OS=Rattus norvegicus GN=Ppt1 PE=1 SV=1 - [PPT1_RAT]	34.4	0.013	0.013	0.013	0.013	0.013	0.013	0.013	0.013	0.013	0.013	1.173	1.179	2.227	2.651	1.059	2.087	0.815	1.444	1.525	1.416	#DIV/0!	#DIV/0!	0.009	0.042	0.009	0.044	0.009	0.034	0.009	0.035	0.917	1.029
P46418	Glutathione S-transferase alpha-5 OS=Rattus norvegicus GN=Gsta5 PE=1 SV=2 - [GSTA5_RAT]	25.3	0.013	0.013	0.013	0.013	0.013	0.013	0.013	0.013	0.013	0.013	1.269	1.415	2.254	8.328	0.682	3.676	0.811	0.925	0.926	0.896	#DIV/0!	#DIV/0!	0.009	0.027	0.009	0.045	0.009	0.022	0.009	0.036	0.354	1.395
P46953	3-hydroxyanthranilate 3,4-dioxygenase OS=Rattus norvegicus OX=10116 GN=Haao PE=1 SV=2 - [3HAO_RAT]	32.6	0.080	0.572	0.163	0.413	0.284	0.013	0.013	0.013	0.013	0.013	0.807	0.784	1.523	4.328	0.933	3.298	0.605	1.273	1.131	0.989	0.009	3.593	0.014	0.040	0.014	0.043	0.009	0.033	0.009	0.035	0.917	1.023
P48508	Glutamate--cysteine ligase regulatory subunit OS=Rattus norvegicus GN=Gclm PE=1 SV=1 - [GSH0_RAT]	30.5	0.013	0.013	0.013	0.013	0.013	0.013	0.013	0.013	0.013	0.013	0.741	0.726	1.144	4.739	0.740	4.827	0.578	1.017	0.966	0.788	#DIV/0!	#DIV/0!	0.009	0.044	0.009	0.037	0.009	0.036	0.009	0.028	0.756	1.175
P49134	Integrin beta-1 OS=Rattus norvegicus GN=Itgb1 PE=2 SV=1 - [ITB1_RAT]	88.4	0.013	0.013	0.013	0.013	0.013	0.013	0.013	0.013	0.013	0.013	1.213	1.114	2.219	4.272	0.823	3.336	0.525	1.563	1.515	0.986	#DIV/0!	#DIV/0!	0.009	0.033	0.009	0.038	0.009	0.026	0.009	0.029	0.756	1.136
P50123	Glutamyl aminopeptidase OS=Rattus norvegicus OX=10116 GN=Enpep PE=1 SV=2 - [AMPE_RAT]	107.9	0.013	0.013	0.013	0.013	0.013	0.013	0.013	0.013	0.013	0.013	0.670	0.697	1.297	4.683	0.791	3.775	0.525	1.043	1.043	0.891	#DIV/0!	#DIV/0!	0.009	0.043	0.009	0.045	0.009	0.035	0.009	0.035	0.917	1.037
P50408	V-type proton ATPase subunit F OS=Rattus norvegicus GN=Atp6v1f PE=1 SV=1 - [VATF_RAT]	13.4	0.013	0.013	0.013	0.013	0.013	0.013	0.013	0.013	0.013	0.013	1.260	1.166	2.022	4.330	0.951	3.906	0.511	1.539	1.640	1.149	#DIV/0!	#DIV/0!	0.009	0.032	0.009	0.033	0.009	0.025	0.009	0.025	0.756	1.120

P50430	Arylsulfatase B OS=Rattus norvegicus GN=Arsb PE=2 SV=2 - [ARSB_RAT]	58.9	0.013	0.013	0.013	0.013	0.013	0.013	0.013	0.013	0.013	0.013	0.608	0.532	1.012	4.347	0.651	4.440	0.783	0.516	0.680	0.398	#DIV/0!	#DIV/0!	0.009	0.055	0.009	0.053	0.009	0.047	0.009	0.042	0.756	1.303
P51652	Aldo-keto reductase family 1 member C18 OS=Rattus norvegicus GN=Akr1c18 PE=1 SV=1 - [AKC1H_RAT]	37.3	0.013	0.013	0.013	0.013	0.013	0.013	0.013	0.013	0.013	0.013	0.669	0.630	1.357	4.656	0.533	3.572	0.478	0.930	0.803	0.541	#DIV/0!	#DIV/0!	0.009	0.048	0.009	0.058	0.009	0.039	0.009	0.047	0.756	1.286
P52631	Signal transducer and activator of transcription 3 OS=Rattus norvegicus GN=Stat3 PE=1 SV=1 - [STAT3_RAT]	88.0	0.013	0.013	0.013	0.013	0.013	0.013	0.013	0.013	0.013	0.013	1.091	1.344	1.757	3.143	0.991	3.353	0.358	1.515	1.352	0.968	#DIV/0!	#DIV/0!	0.009	0.042	0.009	0.042	0.009	0.033	0.009	0.032	0.756	1.192
P52873	Pyruvate carboxylase, mitochondrial OS=Rattus norvegicus GN=Pc PE=1 SV=2 - [PYC_RAT]	129.7	0.013	0.013	0.013	0.013	0.013	0.013	0.013	0.013	0.013	0.013	0.793	0.773	1.284	2.405	0.774	3.271	0.638	1.403	1.084	0.923	#DIV/0!	#DIV/0!	0.009	0.063	0.009	0.044	0.009	0.054	0.009	0.034	0.605	1.456
P53676	AP-3 complex subunit mu-1 OS=Rattus norvegicus GN=Ap3m1 PE=1 SV=1 - [AP3M1_RAT]	47.0	0.013	0.013	0.013	0.013	0.013	0.013	0.013	0.013	0.013	0.013	1.047	1.914	2.301	3.106	1.452	2.065	0.767	1.729	1.316	0.758	#DIV/0!	#DIV/0!	0.009	0.032	0.009	0.055	0.009	0.025	0.009	0.044	0.184	1.018
P53812	Phosphatidylinositol transfer protein beta isoform OS=Rattus norvegicus GN=Pitpnb PE=1 SV=2 - [PIPNB_RAT]	31.4	0.013	0.013	0.013	0.013	0.013	0.013	0.013	0.013	0.013	0.013	1.160	1.120	2.129	3.417	1.540	1.039	1.866	0.690	2.147	2.258	1.102	#DIV/0!	#DIV/0!	0.009	0.058	0.009	0.038	0.009	0.049	0.009	0.028	0.756	1.230
P55159	Serum paraoxonase/arylesterase 1 OS=Rattus norvegicus GN=Pon1 PE=1 SV=3 - [PON1_RAT]	39.3	0.013	0.013	0.013	0.013	0.013	0.013	0.013	0.013	0.013	0.013	1.309	1.170	2.123	3.204	0.947	3.927	0.520	1.749	1.525	0.824	#DIV/0!	#DIV/0!	0.009	0.039	0.009	0.034	0.009	0.031	0.009	0.026	0.756	1.144
P61459	Pterin-4-alpha-carbinolamine dehydratase OS=Rattus norvegicus OX=10116 GN=Pcbd1 PE=1 SV=2 - [PHS_RAT]	12.0	0.013	0.013	0.013	0.013	0.013	0.517	0.576	0.623	0.904	0.928	0.951	1.061	1.918	2.673	1.052	2.346	0.858	1.228	1.271	1.084	0.009	0.513	0.009	0.049	0.009	0.053	0.014	0.030	0.036	0.073	0.917	1.080
P62749	Hippocalcin-like protein 1 OS=Rattus norvegicus GN=Hpcal1 PE=1 SV=2 - [HPCL1_RAT]	22.3	0.013	0.013	0.013	0.013	0.013	0.013	0.013	0.013	0.013	0.013	1.792	0.971	2.086	2.266	0.772	3.420	0.657	1.644	1.379	1.017	#DIV/0!	#DIV/0!	0.009	0.047	0.009	0.037	0.009	0.038	0.009	0.028	0.756	1.188
P62944	AP-2 complex subunit beta OS=Rattus norvegicus GN=Ap2b1 PE=1 SV=1 - [AP2B1_RAT]	104.5	0.013	0.013	0.013	0.013	0.013	0.013	0.013	0.013	0.013	0.013	1.172	1.173	2.107	3.709	1.077	2.788	0.588	1.720	1.627	1.213	#DIV/0!	#DIV/0!	0.009	0.035	0.009	0.038	0.009	0.027	0.009	0.029	0.917	0.971
P68511	14-3-3 protein eta OS=Rattus norvegicus GN=Ywhah PE=1 SV=2 - [1433F_RAT]	28.2	0.774	0.893	0.616	1.021	1.017	1.080	0.497	0.691	0.903	0.937	1.119	1.123	2.213	1.437	1.275	1.212	0.980	1.285	1.209	1.270	0.917	0.979	0.014	0.035	0.036	0.087	0.014	0.030	0.022	0.051	0.470	1.573
P70470	Acyl-protein thioesterase 1 OS=Rattus norvegicus GN=Lypla1 PE=1 SV=1 - [LYPA1_RAT]	24.7	0.751	0.822	0.565	0.880	0.872	1.037	0.568	0.750	0.834	0.943	0.980	0.914	1.734	2.649	1.212	1.977	0.907	1.195	1.124	1.277	0.605	1.043	0.014	0.036	0.014	0.040	0.056	0.068	0.036	0.072	0.756	1.304
P70580	Membrane-associated progesterone receptor component 1 OS=Rattus norvegicus OX=10116 GN=Pgrmc1 PE=1 SV=3 - [PGRC1_RAT]	21.6	0.013	0.013	0.013	0.013	0.013	0.013	0.013	0.013	0.013	0.013	1.352	1.119	3.075	0.802	6.233	0.013	2.677	2.297	1.405	#DIV/0!	#DIV/0!	0.009	0.033	0.025	0.065	0.009	0.026	0.025	0.055	0.756	1.099
P70618	Mitogen-activated protein kinase 14 OS=Rattus norvegicus GN=Mapk14 PE=2 SV=3 - [MK14_RAT]	41.3	0.013	0.013	0.013	0.013	0.013	0.013	0.013	0.013	0.013	0.013	1.115	1.137	2.107	2.770	0.991	3.340	0.466	1.749	1.335	0.832	#DIV/0!	#DIV/0!	0.009	0.043	0.009	0.040	0.009	0.035	0.009	0.031	0.756	1.193
P70627	Glutamate carboxypeptidase 2 OS=Rattus norvegicus GN=Folh1 PE=1 SV=1 - [FOLH1_RAT]	84.5	0.013	0.013	0.013	0.013	0.013	0.013	0.013	0.013	0.013	0.013	0.944	0.957	1.933	3.462	1.134	4.081	0.054	1.826	1.637	1.053	#DIV/0!	#DIV/0!	0.009	0.041	0.009	0.033	0.009	0.033	0.009	0.026	0.917	0.973
P80299	Bifunctional epoxide hydrolase 2 OS=Rattus norvegicus OX=10116 GN=Ephx2 PE=1 SV=1 - [HYES_RAT]	62.3	0.184	0.747	0.160	0.664	0.669	0.013	0.013	0.013	0.013	0.013	0.882	1.061	1.614	4.910	0.823	4.013	0.485	1.453	1.259	1.398	0.009	2.156	0.014	0.033	0.056	0.1003	0.009	0.027	0.009	0.026	0.917	0.953
P84079	ADP-ribosylation factor 1 OS=Rattus norvegicus GN=Arf1 PE=1 SV=2 - [ARF1_RAT]	20.7	0.013	0.013	0.013	0.013	0.013	0.013	0.013	0.013	0.013	0.013	1.831	1.371	3.784	3.402	1.455	3.648	0.633	3.649	3.205	2.128	#DIV/0!	#DIV/0!	0.009	0.028	0.009	0.029	0.009	0.023	0.009	0.022	0.756	1.094
P84855	Major urinary protein (Fragments) OS=Rattus rattus OX=10117 PE=1 SV=2 - [MUP_RATRT]	5.3	0.013	0.013	0.013	0.013	0.013	0.013	0.013	0.013	0.013	0.013	0.574	0.644	1.218	3.876	1.229	6.378	0.808	0.793	0.725	0.782	#DIV/0!	#DIV/0!	0.009	0.050	0.009	0.031	0.009	0.043	0.009	0.024	0.917	0.975
P85125	Polymerase I and transcript release factor OS=Rattus norvegicus GN=Ptrf PE=1 SV=1 - [PTRF_RAT]	43.9	0.694	0.653	0.674	0.833	0.954	0.013	0.013	0.013	0.013	0.013	0.881	1.063	1.970	2.037	1.019	1.872	0.896	1.158	1.227	1.024	0.009	0.317	0.022	0.044	0.022	0.061	0.009	0.049	0.009	0.052	0.917	1.169
P85845	Fascin OS=Rattus norvegicus GN=Fscn1 PE=1 SV=2 - [FSCN1_RAT]	54.5	0.013	0.013	0.013	0.013	0.013	0.013	0.013	0.013	0.013	0.013	0.926	0.915	1.895	2.861	1.152	3.147	0.660	1.067	2.254	1.227	#DIV/0!	#DIV/0!	0.009	0.048	0.009	0.035	0.009	0.040	0.009	0.027	0.756	1.182
Q01460	Di-N-acetylchitobiase OS=Rattus norvegicus GN=Ctbs PE=1 SV=1 - [DIAC_RAT]	41.5	0.013	0.013	0.013	0.013	0.013	0.013	0.013	0.013	0.013	0.013	1.297	1.159	1.837	3.083	0.841	3.586	0.652	1.425	1.465	1.066	#DIV/0!	#DIV/0!	0.009	0.043	0.009	0.036	0.009	0.034	0.009	0.027	0.917	0.995
Q01579	Glutathione S-transferase theta-1 OS=Rattus norvegicus OX=10116 GN=Gstt1 PE=1 SV=2 - [GSTT1_RAT]	27.5	0.013	0.013	0.013	0.013	0.013	0.013	0.013	0.013	0.013	0.013	0.787	0.935	1.227	4.997	0.800	4.019	0.550	1.356	1.194	0.684	#DIV/0!	#DIV/0!	0.009	0.039	0.009	0.040	0.009	0.031	0.009	0.030	0.756	1.163
Q02765	Cathepsin S OS=Rattus norvegicus OX=10116 GN=Ctss PE=2 SV=1 - [CATS_RAT]	36.8	0.013	0.013	0.013	0.013	0.013	0.013	0.013	0.013	0.013	0.013	0.620	0.620	1.202	4.112	0.747	4.147	0.493	1.155	1.400	1.168	#DIV/0!	#DIV/0!	0.009	0.053	0.009	0.035	0.009	0.045	0.009	0.027	0.605	1.368
Q03248	Beta-ureidopropionase OS=Rattus norvegicus OX=10116 GN=Upb1 PE=1 SV=1 - [BUP1_RAT]	44.0	0.013	0.013	0.013	0.013	0.013	0.013	0.013	0.013	0.013	0.013	0.649	0.725	1.195	5.685	0.644	6.049	0.390	1.108	0.945	0.686	#DIV/0!	#DIV/0!	0.009	0.038	0.009	0.032	0.009	0.030	0.009	0.024	0.917	0.949
Q03336	Regucalcin OS=Rattus norvegicus GN=Rgn PE=1 SV=3 - [RGN_RAT]	33.4	0.013	0.013	0.013	0.013	0.013	0.013	0.013	0.013	0.013	0.013	0.905	1.056	1.974	1.899	1.067	1.778	0.620	1.257	1.316	1.251	#DIV/0!	#DIV/0!	0.009	0.060	0.009	0.062	0.009	0.051	0.009	0.051	0.917	1.173
Q03555	Gephyrin OS=Rattus norvegicus GN=Gphn PE=1 SV=3 - [GEPH_RAT]	83.2	0.013	0.013	0.013	0.013	0.013	0.013	0.013	0.013	0.013	0.013	1.003	1.052	2.078	2.563	1.172	1.824	0.857	1.688	2.335	1.400	#DIV/0!	#DIV/0!	0.009	0.048	0.009	0.037	0.009	0.038	0.009	0.028	0.917	1.009
Q07439	Heat shock 70 kDa protein 1A/1B OS=Rattus norvegicus GN=Hspa1a PE=2 SV=2 - [HSP71_RAT]	70.1	0.013	0.013	0.013	0.013	0.013	0.013	0.013	0.013	0.013	0.013	1.139	1.247	2.129	3.147	1.070	2.753	0.495	1.391	1.431	0.687	#DIV/0!	#DIV/0!	0.009	0.039	0.009	0.054	0.009	0.031	0.009	0.043	0.605	1.379
Q07523	Hydroxyacid oxidase 2 OS=Rattus norvegicus GN=Hao2 PE=1 SV=2 - [HAOX2_RAT]	39.2	0.238	0.255	0.196	0.253	0.228	0.302	0.156	0.195	0.278	0.253	0.503	0.558	0.856	8.212	0.532	8.156	0.490	0.778	0.708	0.610	0.917	1.085	0.014	0.033	0.014	0.039	0.014	0.029	0.014	0.032	0.917	0.922
Q0VGK3	Glycerate kinase OS=Rattus norvegicus OX=10116 GN=Glyctk PE=1 SV=1 - [GLCTK_RAT]	55.2	0.013	0.013	0.013	0.013	0.013	0.013	0.013	0.013	0.013	0.013	0.867	1.414	1.505	5.723	1.022	6.371	0.231	1.462	1.368	1.164	#DIV/0!	#DIV/0!	0.009	0.030	0.009	0.030	0.009	0.024	0.009	0.023	0.917	0.923
Q10728	Protein phosphatase 1 regulatory subunit 12A OS=Rattus norvegicus GN=Ppp1r12a PE=1 SV=2 - [MYPT1_RAT]	115.2	0.013	0.013	0.013	0.013	0.013	0.013	0.013	0.013	0.013	0.013	1.234	1.203	3.210	2.456	1.047	2.133	0.943	1.637	1.552	1.635	#DIV/0!	#DIV/0!	0.009	0.035	0.009	0.039	0.009	0.028	0.009	0.029	0.917	0.973
Q32KJ6	N-acetylgalactosamine-6-sulfatase OS=Rattus norvegicus GN=Galns PE=2 SV=1 - [GALNS_RAT]	58.3	0.013	0.013	0.013	0.013	0.013	0.013	0.013	0.013	0.013	0.013	0.865	1.096	1.893	4.669	1.086	4.346	0.699	1.298	1.309	1.071	#DIV/0!	#DIV/0!	0.009	0.033	0.009	0.033	0.009	0.026	0.009	0.025	0.756	1.122
Q32PY9	Probable gluconokinase OS=Rattus norvegicus GN=Idnk PE=2 SV=1 - [GNTK_RAT]	20.2	0.013	0.013	0.013	0.013	0.013	0.013	0.013	0.013	0.013	0.013	0.756	1.100	1.107	5.319	0.714	2.449	0.714	1.158	0.965	0.655	#DIV/0!	#DIV/0!	0.009	0.037	0.009	0.068	0.009	0.029	0.009	0.056	0.605	1.401
Q3T1J9	MOB kinase activator 1A OS=Rattus norvegicus GN=Mob1a PE=2 SV=3 - [MOB1A_RAT]	25.1	0.013	0.013	0.013	0.013	0.013	0.013	0.013	0.013	0.013	0.013	0.951	0.899	1.656	1.874	1.000	1.085	0.779	1.811	2.199	0.756	#DIV/0!	#DIV/0!	0.009	0.066	0.009	0.074	0.009	0.059	0.009	0.061	0.605	1.618
Q4FZV0	Beta-mannosidase OS=Rattus norvegicus GN=Manba PE=2 SV=1 - [MANBA_RAT]	101.2	0.013	0.013	0.013	0.013	0.013	0.013	0.013	0.013	0.013	0.013	1.093	1.173	2.179	3.461	1.029	2.724	0.570	1.206	1.357	1.034	#DIV/0!	#DIV/0!	0.009	0.037	0.009	0.051	0.009	0.029	0.009	0.041	0.756	1.196
Q4G069	Regulator of microtubule dynamics protein 1 OS=Rattus norvegicus GN=Rmdn1 PE=2 SV=1 - [RMD1_RAT]	35.4	0.013	0.013	0.013	0.013	0.013	0.013	0.013	0.013	0.013	0.013	1.158	1.095	1.930	5.777	0.862	4.922	0.226	1.818	1.520	0.921	#DIV/0!	#DIV/0!	0.009	0.030	0.009	0.031	0.009	0.024	0.009	0.024	0.756	1.104
Q4KM35	Proteasome subunit beta type-10 OS=Rattus norvegicus GN=Psmb10 PE=2 SV=1 - [PSB10_RAT]	29.0	0.013	0.013	0.013	0.013	0.013	0.013	0.013	0.013	0.013	0.013	1.168	1.229	2.136	2.212	1.363	2.348	0.851	1.373	1.471	1.489	#DIV/0!	#DIV/0!	0.009	0.044	0.009	0.042	0.009	0.035	0.009	0.033	0.756	1.208
Q4KM65	Cleavage and polyadenylation specificity factor subunit 5 OS=Rattus norvegicus GN=Nudt21 PE=2 SV=1 - [CPSF5_RAT]	26.2	0.013	0.013	0.013	0.013	0.013	0.013	0.013	0.013	0.013	0.013	1.395	1.150	2.421	2.025	1.094	2.275	0.733	1.531	1.753	1.539	#DIV/0!	#DIV/0!	0.009	0.044	0.009	0.039	0.009	0.036	0.009	0.030	0.917	1.014
Q4KM86	Gamma-glutamylaminecyclotransferase OS=Rattus norvegicus GN=Ggact PE=2 SV=1 - [GGACT_RAT]	16.9	0.901	0.736	0.684	0.979	1.046	0.013	0.013	0.013	0.013	0.013	2.231	1.967	4.177	3.242	1.464	2.427	0.180	2.498	2.259	2.686	0.009	0.109	0.014	0.028	0.127	0.157	0.009	0.022	0.009	0.023	0.917	0.919
Q4QQT3	CUGBP Elav-like family member 1 OS=Rattus norvegicus GN=Celf1 PE=2 SV=1 - [CELF1_RAT]	52.2	0.013	0.013	0.013	0.013	0.013	0.013	0.013	0.013	0.013	0.013	1.717	1.241	3.189	3.448	1.818	3.005	0.663	1.768	1.912	1.588	#DIV/0!	#DIV/0!	0.009	0.029	0.009	0.032	0.009	0.023	0.009	0.025	0.470	1.373
Q4VSI4	Ubiquitin carboxyl-terminal hydrolase 7 OS=Rattus norvegicus GN=Usp7 PE=1 SV=1 - [UBP7_RAT]	128.3	0.620	1.110	0.456	1.042	0.975	0.013	0.013	0.013	0.013	0.013	1.209	1.271	2.013	2.308	1.145	2.600	0.470	1.824	1.624	1.197	0.009	0.130	0.014	0.030	0.085	0.119	0.009	0.032	0.009	0.024	0.756	1.174
Q561R9	Beta-lactamase-like protein 2 OS=Rattus norvegicus GN=Lactb2 PE=2 SV=1 - [LACB2_RAT]	32.5	0.266	0.857	0.165	0.799	0.708	0.013	0.013	0.013	0.013	0.013	0.877	0.833	1.563	3.705	0.829	3.002	0.648	1.229	1.153	1.014	0.009	1.347	0.036	0.061	0.056	0.125	0.009	0.039	0.009	0.039	0.917	1.061
Q566E5	KDEL motif-containing protein 2 OS=Rattus norvegicus GN=Kdelc2 PE=2 SV=1 - [KDEL2_RAT]	58.7	0.013	0.013	0.013	0.013	0.013	0.013	0.013	0.013	0.013	0.013	1.012	0.907	2.784	3.849	1.094	3.645	0.716	1.626	1.341	1.217	#DIV/0!	#DIV/0!	0.009	0.033	0.009	0.034	0.009	0.026	0.009	0.026	0.917	0.947
Q5BJP9	Phytanoyl-CoA dioxygenase domain-containing protein 1 OS=Rattus norvegicus OX=10116 GN=Phyhd1 PE=2 SV=1 - [PHYD1_RAT]	32.5	0.013	0.013	0.013	0.013	0.013	0.013	0.013	0.013	0.013	0.013	0.719	0.872	1.525	3.858	0.807	3.593	1.225	1.216	1.602	1.219	#DIV/0!	#DIV/0!	0.009	0.048	0.009	0.033	0.009	0.039	0.009	0.025	0.354	1.418
Q5FVI3	Leucine-rich repeat-containing protein 57 OS=Rattus norvegicus GN=Lrrc57 PE=2 SV=1 - [LRC57_RAT]	26.7	0.013	0.013	0.013	0.013	0.013	0.013	0.013	0.013	0.013	0.013	0.727	0.711	1.260	1.891	1.120	1.806	1.053	1.029	1.127	1.241	#DIV/0!	#DIV/0!	0.009	0.080	0.009	0.061	0.009	0.072	0.009	0.050	0.756	1.472
Q5FVI6	V-type proton ATPase subunit C 1 OS=Rattus norvegicus GN=Atp6v1c1 PE=2 SV=1 - [VATC1_RAT]	43.9	0.782	0.857	0.604	0.860	0.774	0.013	0.013	0.013	0.013	0.013	0.910	0.919	1.683	3.145	0.931	3.018	0.667	1.104	1.075	0.731	0.009	0.257	0.014	0.035	0.354	0.357	0.009	0.042	0.009	0.045	0.605	1.435
Q5HZE4	Methylthioribose-1-phosphate isomerase OS=Rattus norvegicus GN=Mri1 PE=2 SV=1 - [MTNA_RAT]	39.6	1.181	0.889	0.570	0.927	0.881	0.013	0.013	0.013	0.013	0.013	1.364	1.586	1.996	1.578	1.121	1.708	0.780	2.314	1.386	0.937	0.009	0.099	0.022	0.041	0.127	0.162	0.009	0.041	0.009	0.036	0.756	1.245
Q5HZV9	Protein phosphatase 1 regulatory subunit 7 OS=Rattus norvegicus GN=Ppp1r7 PE=1 SV=1 - [PP1R7_RAT]	41.3	0.013	0.013	0.013	0.013	0.013	1.158	0.465	0.695	1.020	0.752	1.152	1.069	2.293	1.699	1.082	2.320	0.767	1.623	1.482	1.474	0.009	0.174	0.009	0.054	0.009	0.041	0.056	0.070	0.036	0.069	0.756	1.239
Q5HZY0	UBX domain-containing protein 4 OS=Rattus norvegicus GN=Ubxn4 PE=1 SV=1 - [UBXN4_RAT]	56.4	0.013	0.013	0.013	0.013	0.013	0.013	0.013	0.013	0.013	0.013	1.469	1.489	2.953	1.631	1.473	1.267	0.627	2.105	1.565	2.039	#DIV/0!	#DIV/0!	0.009	0.036	0.009	0.041	0.009	0.029	0.009	0.032	0.756	1.157
Q5I0D5	Phospholysine phosphohistidine inorganic pyrophosphate phosphatase OS=Rattus norvegicus GN=Lhpp PE=2 SV=1 - [LHPP_RAT]	29.2	0.013	0.013	0.013	0.013	0.013	0.013	0.013	0.013	0.013	0.013	0.952	1.161	1.927	4.598	0.959	4.849	0.481	1.262	1.159	1.207	#DIV/0!	#DIV/0!	0.009	0.033	0.009	0.032	0.009	0.026	0.009	0.025	0.917	0.942
Q5I0D7	Xaa-Pro dipeptidase OS=Rattus norvegicus GN=Pepd PE=2 SV=1 - [PEPD_RAT]	54.7	0.573	0.724	0.474	0.829	0.706	0.944	0.351	0.559	0.722	0.823	0.819	0.821	1.606	2.834	0.927	2.693	0.819	1.109	1.078	1.033	0.917	1.066	0.036	0.061	0.022	0.061	0.127	0.123	0.036	0.075	0.917	1.128
Q5I0M2	Nicotinate-nucleotide pyrophosphorylase [carboxylating] OS=Rattus norvegicus OX=10116 GN=Qprt PE=2 SV=1 - [NADC_RAT]	31.3	0.013	0.013	0.013	0.013	0.013	0.013	0.013	0.013	0.013	0.013	0.525	0.545	0.887	5.020	0.610	5.829	0.470	0.737	0.759	0.578	#DIV/0!	#DIV/0!	0.009	0.050	0.009	0.035	0.009	0.042	0.009	0.027	0.917	1.009
Q5M7U6	Actin-related protein 2 OS=Rattus norvegicus GN=Actr2 PE=1 SV=1 - [ARP2_RAT]	44.7	0.673	0.811	0.627	0.839	0.790	1.142	0.484	0.736	0.766	0.843	0.889	1.103	2.265	1.850	1.227	1.647	0.858	1.499	1.151	1.233	0.756	1.075	0.014	0.037	0.014	0.040	0.036	0.054	0.022	0.051	0.756	1.326
Q5M876	N-acyl-aromatic-L-amino acid amidohydrolase (carboxylate-forming) OS=Rattus norvegicus GN=Acy3 PE=1 SV=1 - [ACY3_RAT]	35.4	0.460	0.604	0.325	0.534	0.544	0.463	0.328	0.356	0.686	0.551	0.768	0.797	1.296	6.436	0.734	5.679	0.566	0.957	0.957	0.750	0.917	1.082	0.014	0.031	0.022	0.059	0.014	0.027	0.022	0.050	0.756	1.113
Q5PQR8	EKC/KEOPS complex subunit Tprkb OS=Rattus norvegicus OX=10116 GN=Tprkb PE=2 SV=1 - [TPRKB_RAT]	17.6	0.013	0.013	0.013	0.013	0.013	0.013	0.013	0.013	0.013	0.013	1.683	1.821	3.098	2.336	1.491	2.281	0.341	2.174	1.801	1.280	#DIV/0!	#DIV/0!	0.009	0.031	0.009	0.039	0.009	0.024	0.009	0.030	0.354	1.402
Q5PQT3	Glycine N-acyltransferase OS=Rattus norvegicus GN=Glyat PE=2 SV=1 - [GLYAT_RAT]	33.9	0.013	0.013	0.013	0.013	0.013	0.013	0.013	0.013	0.013	0.013	0.667	0.656	1.081	7.150	0.649	5.340	0.402	0.962	0.962	0.705	#DIV/0!	#DIV/0!	0.009	0.031	0.009	0.035	0.009	0.025	0.009	0.027	0.917	0.941
Q5RJP0	Aldose reductase-related protein 1 OS=Rattus norvegicus GN=Akr1b7 PE=1 SV=1 - [ALD1_RAT]	36.1	0.013	0.013	0.013	0.013	0.013	0.013	0.013	0.013	0.013	0.013	0.604	0.684	1.339	5.662	0.686	4.448	0.598	0.941	0.937	0.898	#DIV/0!	#DIV/0!	0.009	0.037	0.009	0.039	0.009	0.029	0.009	0.030	0.917	0.980
Q5TKR9	Histone acetyltransferase KAT6A OS=Rattus norvegicus OX=10116 GN=Kat6a PE=1 SV=2 - [KAT6A_RAT]	223.2	0.013	0.013	0.013	0.013	0.013	0.013	0.013	0.013	0.013	0.013	0.544	0.766	1.180	0.513	4.996	0.617	1.153	0.842	1.792	4.481	#DIV/0!	#DIV/0!	0.009	0.046	0.009	0.032	0.009	0.029	0.009	0.025	0.470	1.410
Q5U204	Ragulator complex protein LAMTOR3 OS=Rattus norvegicus GN=Lamtor3 PE=2 SV=1 - [LTOR3_RAT]	13.6	0.013	0.013	0.013	0.013	0.013	0.013	0.013	0.013	0.013	0.013	1.101	1.104	1.997	3.648	1.109	3.976	0.542	1.497	1.369	1.483	#DIV/0!	#DIV/0!	0.009	0.037	0.009	0.033	0.009	0.029	0.009	0.025	0.756	1.127
Q5U2Z3	Nucleosome assembly protein 1-like 4 OS=Rattus norvegicus GN=Nap1l4 PE=1 SV=1 - [NP1L4_RAT]	43.9	0.438	1.458	0.292	1.026	1.042	0.013	0.013	0.013	0.013	0.013	1.728	1.298	2.505	2.222	1.418	1.994	0.549	2.263	1.877	1.574	0.009	0.121	0.036	0.055	0.056	0.089	0.009	0.028	0.009	0.027	0.917	0.965
Q5U316	Ras-related protein Rab-35 OS=Rattus norvegicus GN=Rab35 PE=1 SV=1 - [RAB35_RAT]	23.0	1.259	0.771	0.775	1.064	1.056	0.013	0.013	0.013	0.013	0.013	1.528	1.332	2.297	2.742	1.092	2.585	0.851	1.867	1.899	1.774	0.009	0.065	0.022	0.039	0.056	0.088	0.009	0.029	0.009	0.028	0.917	0.952
Q5U3Y7	Sigma intracellular receptor 2 OS=Rattus norvegicus OX=10116 GN=Tmem97 PE=2 SV=1 - [SGMR2_RAT]	20.9	0.013	0.013	0.013	0.013	0.013	1.333	0.436	0.749	1.572	0.997	1.070	0.940	2.397	0.160	1.236	0.013	0.013	0.013	0.013	0.013	0.009	0.060	0.009	0.079	#DIV/0!	#DIV/0!	0.917	0.656	0.009	0.087	0.009	0.165
Q5XI22	Acetyl-CoA acetyltransferase, cytosolic OS=Rattus norvegicus GN=Acat2 PE=1 SV=1 - [THIC_RAT]	41.1	0.013	0.013	0.013	0.013	0.013	0.013	0.013	0.013	0.013	0.013	1.082	1.066	2.093	5.089	1.307	3.486	0.537	1.611	1.475	1.424	#DIV/0!	#DIV/0!	0.009	0.030	0.009	0.034	0.009	0.024	0.009	0.026	0.917	0.934
Q5XI95	Alcohol dehydrogenase 6 OS=Rattus norvegicus GN=Adh6 PE=2 SV=1 - [ADH6_RAT]	39.7	0.013	0.013	0.013	0.013	0.013	0.013	0.013	0.013	0.013	0.013	0.671	0.717	1.145	5.600	0.652	4.278	0.405	1.215	1.001	0.793	#DIV/0!	#DIV/0!	0.009	0.038	0.009	0.041	0.009	0.030	0.009	0.031	0.756	1.164
Q60587	Trifunctional enzyme subunit beta, mitochondrial OS=Rattus norvegicus GN=Hadhb PE=1 SV=1 - [ECHB_RAT]	51.4	0.505	0.884	0.486	0.963	0.695	0.013	0.013	0.013	0.013	0.013	1.009	1.039	1.778	4.263	0.805	4.663	0.426	1.334	1.429	0.810	0.009	0.634	0.036	0.056	0.259	0.271	0.009	0.030	0.009	0.026	0.917	0.963
Q62638	Golgi apparatus protein 1 OS=Rattus norvegicus GN=Glg1 PE=1 SV=1 - [GSLG1_RAT]	133.5	0.013	0.013	0.013	0.013	0.013	0.013	0.013	0.013	0.013	0.013	1.519	1.352	2.678	2.825	1.192	2.565	0.550	1.977	1.928	1.042	#DIV/0!	#DIV/0!	0.009	0.033	0.009	0.038	0.009	0.026	0.009	0.029	0.470	1.398
Q62658	Peptidyl-prolyl cis-trans isomerase FKBP1A OS=Rattus norvegicus GN=Fkbp1a PE=1 SV=3 - [FKB1A_RAT]	11.9	0.013	0.013	0.013	0.013	0.013	0.013	0.013	0.013	0.013	0.013	0.756	0.743	1.627	1.429	0.803	1.210	0.943	1.071	1.110	0.933	#DIV/0!	#DIV/0!	0.009	0.099	0.009	0.088	0.009	0.088	0.009	0.075	0.605	1.729
Q62667	Major vault protein OS=Rattus norvegicus GN=Mvp PE=1 SV=4 - [MVP_RAT]	95.7	0.761	0.796	0.679	0.962	0.907	1.057	0.498	0.783	0.749	0.979	0.918	1.050	1.835	2.217	1.227	1.776	0.834	1.510	1.201	1.105	0.917	0.996	0.022	0.043	0.036	0.086	0.056	0.070	0.036	0.073	0.605	1.476
Q62687	Sodium-dependent neutral amino acid transporter B(0)AT3 OS=Rattus norvegicus OX=10116 GN=Slc6a18 PE=2 SV=1 - [S6A18_RAT]	69.5	0.013	0.013	0.013	0.013	0.013	0.013	0.013	0.013	0.013	0.013	1.088	0.721	1.525	1.390	1.350	1.000	0.963	1.431	1.155	0.874	#DIV/0!	#DIV/0!	0.009	0.074	0.009	0.083	0.009	0.065	0.009	0.068	0.470	1.724
Q62915	Peripheral plasma membrane protein CASK OS=Rattus norvegicus GN=Cask PE=1 SV=1 - [CSKP_RAT]	103.2	0.013	0.013	0.013	0.013	0.013	0.013	0.013	0.013	0.013	0.013	1.075	0.924	2.153	1.911	1.066	2.129	0.666	1.372	1.254	0.997	#DIV/0!	#DIV/0!	0.009	0.056	0.009	0.058	0.009	0.048	0.009	0.047	0.756	1.338
Q63010	Liver carboxylesterase B-1 OS=Rattus norvegicus PE=1 SV=1 - [EST5_RAT]	62.5	0.258	0.598	0.300	0.475	0.371	0.227	0.140	0.163	0.434	0.422	0.604	0.591	1.028	8.176	0.639	9.391	0.344	1.047	0.923	0.997	0.184	0.787	0.022	0.040	0.056	0.088	0.014	0.027	0.036	0.068	0.756	1.096
Q63028	Alpha-adducin OS=Rattus norvegicus GN=Add1 PE=2 SV=2 - [ADDA_RAT]	80.3	0.013	0.013	0.013	0.013	0.013	0.013	0.013	0.013	0.013	0.013	0.825	0.870	1.722	4.287	0.808	3.077	0.839	1.108	1.044	0.934	#DIV/0!	#DIV/0!	0.009	0.041	0.009	0.049	0.009	0.032	0.009	0.039	0.605	1.374
Q63060	Glycerol kinase OS=Rattus norvegicus GN=Gk PE=2 SV=1 - [GLPK_RAT]	57.4	0.013	0.013	0.013	0.013	0.013	0.013	0.013	0.013	0.013	0.013	0.637	0.698	1.158	5.574	0.654	4.754	0.509	0.943	0.882	0.727	#DIV/0!	#DIV/0!	0.009	0.039	0.009	0.039	0.009	0.031	0.009	0.030	0.917	0.994
Q63342	Dimethylglycine dehydrogenase, mitochondrial OS=Rattus norvegicus GN=Dmgdh PE=1 SV=1 - [M2GD_RAT]	96.0	0.013	0.013	0.013	0.013	0.013	0.013	0.013	0.013	0.013	0.013	0.762	0.744	1.406	6.096	0.837	3.166	0.767	1.085	0.976	0.823	#DIV/0!	#DIV/0!	0.009	0.032	0.009	0.053	0.009	0.025	0.009	0.043	0.756	1.154
Q63965	Sideroflexin-1 OS=Rattus norvegicus OX=10116 GN=Sfxn1 PE=2 SV=4 - [SFXN1_RAT]	35.5	0.013	0.013	0.013	0.013	0.013	0.013	0.013	0.013	0.013	0.013	1.529	1.300	2.545	3.926	1.186	4.258	0.013	2.162	2.795	0.917	#DIV/0!	#DIV/0!	0.009	0.030	0.025	0.065	0.009	0.024	0.025	0.055	0.917	0.924
Q64194	Lysosomal acid lipase/cholesteryl ester hydrolase OS=Rattus norvegicus GN=Lipa PE=2 SV=1 - [LICH_RAT]	45.2	0.013	0.013	0.013	0.013	0.013	0.013	0.013	0.013	0.013	0.013	1.114	0.921	2.126	4.214	1.109	3.214	1.172	1.441	1.488	1.022	#DIV/0!	#DIV/0!	0.009	0.034	0.009	0.036	0.009	0.026	0.009	0.027	0.756	1.128
Q641X3	Beta-hexosaminidase subunit alpha OS=Rattus norvegicus GN=Hexa PE=2 SV=1 - [HEXA_RAT]	60.5	0.013	0.013	0.013	0.013	0.013	0.013	0.013	0.013	0.013	0.013	0.845	0.856	1.329	4.971	0.831	3.821	0.597	1.031	0.948	0.945	#DIV/0!	#DIV/0!	0.009	0.038	0.009	0.044	0.009	0.030	0.009	0.034	0.917	1.006
Q641Z6	EH domain-containing protein 1 OS=Rattus norvegicus GN=Ehd1 PE=1 SV=1 - [EHD1_RAT]	60.6	0.013	0.013	0.013	0.013	0.013	0.013	0.013	0.013	0.013	0.013	1.548	1.301	1.944	3.856	0.932	4.282	0.475	2.420	1.807	0.853	#DIV/0!	#DIV/0!	0.009	0.033	0.009	0.030	0.009	0.026	0.009	0.023	0.917	0.930
Q64230	Meprin A subunit alpha OS=Rattus norvegicus OX=10116 GN=Mep1a PE=1 SV=2 - [MEP1A_RAT]	85.1	0.013	0.013	0.013	0.013	0.013	0.013	0.013	0.013	0.013	0.013	1.013	1.046	1.546	4.818	0.938	3.858	0.608	0.934	1.038	0.524	#DIV/0!	#DIV/0!	0.009	0.034	0.009	0.050	0.009	0.027	0.009	0.040	0.184	1.022
Q64268	Heparin cofactor 2 OS=Rattus norvegicus GN=Serpind1 PE=1 SV=1 - [HEP2_RAT]	54.5	0.764	0.702	0.553	0.924	0.899	0.013	0.013	0.013	0.013	0.013	1.037	0.925	1.834	3.104	0.833	2.680	0.898	1.309	1.046	0.773	0.009	0.291	0.036	0.058	0.085	0.128	0.009	0.040	0.009	0.044	0.756	1.268
Q642A7	Protein FAM151A OS=Rattus norvegicus GN=Fam151a PE=2 SV=1 - [F151A_RAT]	67.1	0.013	0.013	0.013	0.013	0.013	0.013	0.013	0.013	0.013	0.013	1.095	1.317	1.704	5.633	1.010	2.447	0.284	1.231	1.685	1.423	#DIV/0!	#DIV/0!	0.009	0.030	0.009	0.045	0.009	0.024	0.009	0.037	0.917	0.954
Q64319	Neutral and basic amino acid transport protein rBAT OS=Rattus norvegicus GN=Slc3a1 PE=1 SV=1 - [SLC31_RAT]	78.5	0.013	0.013	0.013	0.013	0.013	0.013	0.013	0.013	0.013	0.013	0.880	0.775	1.380	5.323	0.866	4.440	0.439	1.264	1.159	0.909	#DIV/0!	#DIV/0!	0.009	0.035	0.009	0.037	0.009	0.028	0.009	0.028	0.917	0.964
Q64550	UDP-glucuronosyltransferase 1-1 OS=Rattus norvegicus OX=10116 GN=Ugt1a1 PE=1 SV=1 - [UD11_RAT]	59.6	0.013	0.013	0.013	0.013	0.013	0.013	0.013	0.013	0.013	0.013	0.720	0.617	1.022	3.539	0.640	4.024	0.697	0.966	0.969	0.540	#DIV/0!	#DIV/0!	0.009	0.065	0.009	0.046	0.009	0.056	0.009	0.036	0.917	1.129
Q64560	Tripeptidyl-peptidase 2 OS=Rattus norvegicus GN=Tpp2 PE=2 SV=3 - [TPP2_RAT]	138.2	0.013	0.013	0.013	0.013	0.013	0.013	0.013	0.013	0.013	0.013	0.760	1.079	1.752	2.412	1.188	1.573	0.837	1.140	0.876	0.827	#DIV/0!	#DIV/0!	0.009	0.055	0.009	0.089	0.009	0.047	0.009	0.076	0.354	1.575
Q64565	Alanine--glyoxylate aminotransferase 2, mitochondrial OS=Rattus norvegicus OX=10116 GN=Agxt2 PE=1 SV=2 - [AGT2_RAT]	57.2	0.181	0.228	0.180	0.257	0.300	0.290	0.182	0.223	0.284	0.244	0.604	0.626	1.073	6.656	0.651	6.009	0.573	0.780	0.791	0.565	0.605	1.155	0.014	0.038	0.014	0.041	0.014	0.031	0.014	0.033	0.605	1.307
Q64573	Liver carboxylesterase 4 OS=Rattus norvegicus PE=2 SV=2 - [EST4_RAT]	62.3	0.053	0.275	0.109	0.406	0.270	0.209	0.056	0.179	0.347	0.356	0.518	0.590	0.894	5.929	0.705	6.246	0.424	0.877	0.789	0.615	0.917	1.086	0.014	0.040	0.014	0.040	0.014	0.032	0.014	0.033	0.917	0.961
Q64602	Kynurenine/alpha-aminoadipate aminotransferase, mitochondrial OS=Rattus norvegicus OX=10116 GN=Aadat PE=1 SV=1 - [AADAT_RAT]	47.8	0.013	0.013	0.013	0.013	0.013	0.013	0.013	0.013	0.013	0.013	0.718	0.779	1.282	3.688	0.838	3.124	0.640	1.103	1.202	0.771	#DIV/0!	#DIV/0!	0.009	0.053	0.009	0.052	0.009	0.045	0.009	0.042	0.756	1.293
Q64611	Cysteine sulfinic acid decarboxylase OS=Rattus norvegicus GN=Csad PE=1 SV=1 - [CSAD_RAT]	55.2	0.013	0.013	0.013	0.013	0.013	0.013	0.013	0.013	0.013	0.013	1.494	1.303	2.677	4.866	1.129	3.647	0.613	1.454	1.244	1.182	#DIV/0!	#DIV/0!	0.009	0.029	0.009	0.036	0.009	0.023	0.009	0.028	0.470	1.376
Q66H12	Alpha-N-acetylgalactosaminidase OS=Rattus norvegicus GN=Naga PE=2 SV=1 - [NAGAB_RAT]	46.8	0.524	0.844	0.520	0.790	0.854	1.007	0.359	0.555	0.929	1.040	0.908	1.042	1.856	2.206	0.929	2.276	1.013	1.315	1.367	1.295	0.354	1.043	0.014	0.039	0.014	0.039	0.184	0.159	0.022	0.050	0.470	1.481
Q66HG3	Beta-Ala-His dipeptidase OS=Rattus norvegicus GN=Cndp1 PE=1 SV=1 - [CNDP1_RAT]	54.9	0.013	0.013	0.013	0.013	0.013	0.013	0.013	0.013	0.013	0.013	0.681	0.777	1.371	5.084	0.746	6.111	0.512	1.364	1.539	1.113	#DIV/0!	#DIV/0!	0.009	0.040	0.009	0.029	0.009	0.032	0.009	0.023	0.605	1.299
Q66HG4	Aldose 1-epimerase OS=Rattus norvegicus OX=10116 GN=Galm PE=1 SV=1 - [GALM_RAT]	37.9	0.215	0.532	0.123	0.563	0.583	0.381	0.156	0.196	0.550	0.603	0.688	0.739	1.222	6.857	0.623	5.901	0.543	1.050	1.040	0.948	0.917	1.084	0.014	0.032	0.036	0.083	0.014	0.029	0.036	0.070	0.917	0.929
Q66HG9	Mitochondrial antiviral-signaling protein OS=Rattus norvegicus OX=10116 GN=Mavs PE=1 SV=1 - [MAVS_RAT]	53.8	1.006	0.768	0.619	1.192	0.974	0.013	0.013	0.013	0.013	0.013	1.339	1.150	2.668	2.302	1.398	1.591	0.636	2.036	2.044	1.410	0.009	0.082	0.022	0.040	0.085	0.118	0.009	0.030	0.009	0.031	0.917	0.991
Q68FT3	Pyridine nucleotide-disulfide oxidoreductase domain-containing protein 2 OS=Rattus norvegicus OX=10116 GN=Pyroxd2 PE=2 SV=1 - [PYRD2_RAT]	62.8	0.266	0.392	0.192	0.452	0.381	0.480	0.238	0.351	0.512	0.409	0.650	0.698	1.032	6.610	0.765	6.307	0.432	0.836	0.835	0.662	0.354	1.102	0.014	0.036	0.022	0.059	0.014	0.029	0.022	0.071	0.917	0.939
Q68FU4	Succinate--hydroxymethylglutarate CoA-transferase OS=Rattus norvegicus OX=10116 GN=Sugct PE=2 SV=1 - [SUCHY_RAT]	47.5	0.013	0.013	0.013	0.013	0.013	0.013	0.013	0.013	0.013	0.013	0.735	0.953	1.237	5.500	0.870	5.624	0.342	1.079	1.355	0.827	#DIV/0!	#DIV/0!	0.009	0.035	0.009	0.031	0.009	0.027	0.009	0.024	0.917	0.943
Q68FX1	Mannose-6-phosphate isomerase OS=Rattus norvegicus GN=Mpi PE=2 SV=1 - [MPI_RAT]	46.4	0.512	1.029	0.527	0.980	1.028	0.013	0.013	0.013	0.013	0.013	1.311	1.350	2.440	3.116	1.239	2.757	0.402	1.896	1.463	1.070	0.009	0.177	0.014	0.028	0.127	0.161	0.009	0.024	0.009	0.032	0.605	1.317
Q6AXM7	HBS1-like protein OS=Rattus norvegicus GN=Hbs1l PE=2 SV=1 - [HBS1L_RAT]	74.7	0.680	1.166	0.452	1.222	0.960	0.013	0.013	0.013	0.013	0.013	1.577	1.829	2.959	3.536	1.077	1.600	0.715	2.109	1.816	1.046	0.009	0.094	0.036	0.054	0.127	0.160	0.009	0.023	0.009	0.035	0.259	1.260
Q6AXM8	Serum paraoxonase/arylesterase 2 OS=Rattus norvegicus OX=10116 GN=Pon2 PE=2 SV=1 - [PON2_RAT]	39.6	0.013	0.013	0.013	0.013	0.013	0.013	0.013	0.013	0.013	0.013	1.658	1.501	3.573	1.469	1.183	2.195	0.392	2.859	3.110	1.033	#DIV/0!	#DIV/0!	0.009	0.040	0.009	0.031	0.009	0.031	0.009	0.023	0.756	1.125
Q6AXR4	Beta-hexosaminidase subunit beta OS=Rattus norvegicus GN=Hexb PE=2 SV=1 - [HEXB_RAT]	61.5	0.013	0.013	0.013	0.013	0.013	0.013	0.013	0.013	0.013	0.013	0.686	0.587	1.269	4.897	0.614	3.695	0.590	1.024	0.909	0.704	#DIV/0!	#DIV/0!	0.009	0.045	0.009	0.050	0.009	0.036	0.009	0.040	0.756	1.235
Q6AY80	Ribosyldihydronicotinamide dehydrogenase [quinone] OS=Rattus norvegicus GN=Nqo2 PE=1 SV=3 - [NQO2_RAT]	26.3	0.013	0.013	0.013	0.013	0.013	0.013	0.013	0.013	0.013	0.013	1.091	0.919	1.988	5.823	1.349	3.284	0.526	1.220	1.493	0.991	#DIV/0!	#DIV/0!	0.009	0.029	0.009	0.043	0.009	0.023	0.009	0.033	0.605	1.303
Q6AY86	Vacuolar protein sorting-associated protein 26A OS=Rattus norvegicus GN=Vps26a PE=1 SV=1 - [VP26A_RAT]	38.1	0.013	0.013	0.013	0.013	0.013	0.013	0.013	0.013	0.013	0.013	0.647	0.610	1.098	2.340	0.914	2.126	0.827	1.051	1.076	0.714	#DIV/0!	#DIV/0!	0.009	0.084	0.009	0.072	0.009	0.075	0.009	0.060	0.756	1.507
Q6AYP7	7-methylguanosine phosphate-specific 5'-nucleotidase OS=Rattus norvegicus GN=Nt5c3b PE=2 SV=2 - [5NT3B_RAT]	34.5	0.013	0.013	0.013	0.013	0.013	0.013	0.013	0.013	0.013	0.013	1.602	1.505	2.871	3.056	0.962	3.365	0.523	1.953	1.494	1.208	#DIV/0!	#DIV/0!	0.009	0.031	0.009	0.034	0.009	0.025	0.009	0.026	0.605	1.305
Q6DGG0	Peptidyl-prolyl cis-trans isomerase D OS=Rattus norvegicus GN=Ppid PE=1 SV=3 - [PPID_RAT]	40.7	0.779	0.891	0.597	0.965	0.877	1.084	0.521	0.722	0.821	0.867	0.974	1.040	2.138	1.682	1.098	1.662	0.893	1.127	1.139	0.897	0.605	1.031	0.014	0.037	0.036	0.090	0.036	0.055	0.036	0.077	0.470	1.610
Q6IUU3	Sulfhydryl oxidase 1 OS=Rattus norvegicus GN=Qsox1 PE=1 SV=1 - [QSOX1_RAT]	82.4	0.013	0.013	0.013	0.013	0.013	0.013	0.013	0.013	0.013	0.013	1.156	1.046	2.185	1.942	1.378	1.258	0.871	1.561	1.397	0.939	#DIV/0!	#DIV/0!	0.009	0.049	0.009	0.066	0.009	0.040	0.009	0.054	0.354	1.475
Q6P686	Osteoclast-stimulating factor 1 OS=Rattus norvegicus GN=Ostf1 PE=1 SV=1 - [OSTF1_RAT]	23.7	0.306	1.123	0.169	0.650	0.638	0.346	0.257	0.204	0.935	0.883	1.518	1.378	2.599	4.719	1.121	3.617	0.485	1.804	1.825	1.761	0.917	1.077	0.022	0.039	0.056	0.090	0.014	0.025	0.036	0.069	0.917	0.924

Q6P6S9	Ectonucleoside triphosphate diphosphohydrolase 5 OS=Rattus norvegicus OX=10116 GN=Entpd5 PE=2 SV=1 - [ENTP5_RAT]	47.3	0.013	0.013	0.013	0.013	0.013	0.013	0.013	0.013	0.013	0.013	1.067	0.927	1.642	4.096	0.999	6.293	0.396	1.494	1.329	1.055	#DIV/0!	#DIV/0!	0.009	0.039	0.009	0.030	0.009	0.031	0.009	0.023	0.917	0.932
Q6P7A9	Lysosomal alpha-glucosidase OS=Rattus norvegicus GN=Gaa PE=2 SV=1 - [LYAG_RAT]	106.1	0.013	0.013	0.013	0.013	0.013	0.013	0.013	0.013	0.013	0.013	1.072	0.944	2.049	3.212	1.124	2.207	0.657	1.317	1.171	1.278	#DIV/0!	#DIV/0!	0.009	0.041	0.009	0.055	0.009	0.033	0.009	0.045	0.917	1.051
Q6P7S1	Acid ceramidase OS=Rattus norvegicus GN=Asah1 PE=2 SV=1 - [ASAH1_RAT]	44.4	0.013	0.013	0.013	0.013	0.013	0.013	0.013	0.013	0.013	0.013	1.175	1.223	2.010	3.021	1.045	3.390	0.638	1.787	1.486	1.279	#DIV/0!	#DIV/0!	0.009	0.041	0.009	0.034	0.009	0.032	0.009	0.026	0.756	1.148
Q6P9T8	Tubulin beta-4B chain OS=Rattus norvegicus GN=Tubb4b PE=1 SV=1 - [TBB4B_RAT]	49.8	0.619	1.130	0.591	1.288	1.066	0.013	0.013	0.013	0.013	0.013	1.395	1.411	2.321	2.824	1.110	2.074	0.580	1.888	1.301	0.863	0.009	0.071	0.036	0.054	0.354	0.346	0.009	0.028	0.009	0.044	0.259	1.281
Q6UPE0	Choline dehydrogenase, mitochondrial OS=Rattus norvegicus OX=10116 GN=Chdh PE=1 SV=1 - [CHDH_RAT]	66.3	0.013	0.013	0.013	0.013	0.013	0.013	0.013	0.013	0.013	0.013	0.564	0.655	1.058	5.146	0.596	4.390	0.563	0.857	0.795	0.816	#DIV/0!	#DIV/0!	0.009	0.046	0.009	0.043	0.009	0.037	0.009	0.034	0.917	1.035
Q6XQN1	Nicotinate phosphoribosyltransferase OS=Rattus norvegicus GN=Naprt PE=2 SV=1 - [PNCB_RAT]	58.5	0.013	0.013	0.013	0.013	0.013	0.013	0.013	0.013	0.013	0.013	1.110	0.923	1.946	4.042	0.993	3.270	0.596	1.358	1.222	1.012	#DIV/0!	#DIV/0!	0.009	0.037	0.009	0.043	0.009	0.029	0.009	0.034	0.917	0.997
Q704S8	Carnitine O-acetyltransferase OS=Rattus norvegicus OX=10116 GN=Crat PE=1 SV=1 - [CACP_RAT]	70.8	0.013	0.013	0.013	0.013	0.013	0.013	0.013	0.013	0.013	0.013	0.677	0.755	1.501	3.325	0.826	2.434	0.820	0.889	0.859	0.717	#DIV/0!	#DIV/0!	0.009	0.057	0.009	0.074	0.009	0.048	0.009	0.061	0.917	1.199
Q80Z29	Nicotinamide phosphoribosyltransferase OS=Rattus norvegicus GN=Nampt PE=1 SV=1 - [NAMPT_RAT]	55.4	0.013	0.013	0.013	0.013	0.013	0.013	0.013	0.013	0.013	0.013	1.217	1.265	2.395	2.688	1.237	2.443	0.496	1.034	1.095	0.741	#DIV/0!	#DIV/0!	0.009	0.038	0.009	0.070	0.009	0.030	0.009	0.059	0.085	0.535
Q811X6	Lambda-crystallin homolog OS=Rattus norvegicus OX=10116 GN=Cryl1 PE=1 SV=3 - [CRYL1_RAT]	35.3	0.569	0.698	0.427	0.792	0.673	0.765	0.337	0.509	0.665	0.677	0.743	0.756	1.303	4.375	0.801	3.672	0.665	1.059	0.924	0.851	0.605	1.135	0.036	0.059	0.056	0.117	0.036	0.055	0.036	0.075	0.917	1.046
Q8K4D8	Aldehyde dehydrogenase family 1 member A3 OS=Rattus norvegicus GN=Aldh1a3 PE=2 SV=1 - [AL1A3_RAT]	56.1	0.013	0.013	0.013	0.013	0.013	0.013	0.013	0.013	0.013	0.013	1.159	1.094	2.226	1.471	1.243	2.138	0.404	1.878	1.369	1.804	#DIV/0!	#DIV/0!	0.009	0.055	0.009	0.041	0.009	0.046	0.009	0.032	0.605	1.407
Q8R431	Monoglyceride lipase OS=Rattus norvegicus GN=Mgll PE=1 SV=1 - [MGLL_RAT]	33.5	0.013	0.013	0.013	0.013	0.013	0.013	0.013	0.013	0.013	0.013	0.921	0.753	1.502	2.911	0.827	2.562	0.805	1.347	1.198	0.919	#DIV/0!	#DIV/0!	0.009	0.060	0.009	0.052	0.009	0.051	0.009	0.042	0.917	1.127
Q8R5M5	2-amino-3-carboxymuconate-6-semialdehyde decarboxylase OS=Rattus norvegicus OX=10116 GN=Acmsd PE=1 SV=1 - [ACMSD_RAT]	38.1	0.013	0.013	0.013	0.013	0.013	0.013	0.013	0.013	0.013	0.013	0.890	0.871	1.543	3.595	0.993	4.142	0.646	1.165	1.136	0.982	#DIV/0!	#DIV/0!	0.009	0.047	0.009	0.037	0.009	0.038	0.009	0.028	0.917	1.011
Q8VBU2	Protein NDRG2 OS=Rattus norvegicus GN=Ndrg2 PE=1 SV=1 - [NDRG2_RAT]	40.8	0.013	0.013	0.013	0.013	0.013	0.013	0.013	0.013	0.013	0.013	1.243	2.649	3.252	3.849	1.970	3.750	1.349	1.880	2.440	2.560	#DIV/0!	#DIV/0!	0.009	0.027	0.009	0.029	0.009	0.022	0.009	0.022	0.605	1.291
Q8VD87	Ribonuclease pancreatic gamma-type OS=Rattus rattus OX=10117 PE=3 SV=1 - [RNS1G_RATRT]	16.5	1.724	1.012	1.019	1.303	1.035	0.856	0.958	0.869	1.557	1.073	0.013	0.013	0.013	0.013	0.013	0.013	0.013	0.013	0.013	0.013	0.259	0.819	0.009	0.072	0.009	0.064	0.009	0.090	0.009	0.073	#DIV/0!	#DIV/0!
Q8VI04	Isoaspartyl peptidase/L-asparaginase OS=Rattus norvegicus GN=Asrgl1 PE=1 SV=1 - [ASGL1_RAT]	34.4	0.013	0.013	0.013	0.013	0.013	0.013	0.013	0.013	0.013	0.013	0.486	0.596	0.889	5.742	0.622	5.590	0.473	0.756	0.711	0.676	#DIV/0!	#DIV/0!	0.009	0.042	0.009	0.037	0.009	0.033	0.009	0.028	0.917	0.993
Q91Y78	Ubiquitin carboxyl-terminal hydrolase isozyme L3 OS=Rattus norvegicus GN=Uchl3 PE=1 SV=1 - [UCHL3_RAT]	26.1	0.779	0.914	0.554	1.088	0.955	1.156	0.414	0.696	0.858	0.898	1.023	1.141	1.920	2.001	1.094	1.266	1.054	1.108	1.352	1.174	0.605	1.016	0.022	0.043	0.022	0.060	0.056	0.071	0.036	0.076	0.917	1.171
Q91Y81	Septin-2 OS=Rattus norvegicus GN=Sept2 PE=1 SV=1 - [SEPT2_RAT]	41.6	0.501	0.929	0.542	0.850	0.753	0.930	0.377	0.549	0.961	0.972	0.996	1.133	2.245	1.954	1.147	1.468	0.962	1.316	1.202	0.997	0.354	1.048	0.014	0.037	0.014	0.042	0.014	0.030	0.022	0.051	0.605	1.499
Q91ZN1	Coronin-1A OS=Rattus norvegicus GN=Coro1a PE=1 SV=3 - [COR1A_RAT]	51.0	0.013	0.013	0.013	0.013	0.013	0.013	0.013	0.013	0.013	0.013	1.079	1.426	1.919	2.558	1.031	2.410	0.394	1.828	1.460	1.179	#DIV/0!	#DIV/0!	0.009	0.046	0.009	0.045	0.009	0.037	0.009	0.035	0.917	1.040
Q920D2	Dihydrofolate reductase OS=Rattus norvegicus GN=Dhfr PE=2 SV=3 - [DYR_RAT]	21.6	0.013	0.013	0.013	0.013	0.013	0.013	0.013	0.013	0.013	0.013	1.014	1.127	1.278	8.178	0.744	3.828	0.239	1.142	1.158	0.702	#DIV/0!	#DIV/0!	0.009	0.028	0.009	0.047	0.009	0.022	0.009	0.037	0.605	1.297
Q920F5	Malonyl-CoA decarboxylase, mitochondrial OS=Rattus norvegicus OX=10116 GN=Mlycd PE=1 SV=1 - [DCMC_RAT]	54.7	0.833	0.788	0.812	0.989	0.829	1.388	0.716	0.812	0.683	0.749	0.856	0.987	1.865	2.047	1.035	1.654	0.976	1.465	1.417	0.948	0.259	0.893	0.036	0.060	0.036	0.084	0.056	0.072	0.036	0.071	0.756	1.359
Q924K2	FAS-associated factor 1 OS=Rattus norvegicus GN=Faf1 PE=1 SV=1 - [FAF1_RAT]	73.7	0.013	0.013	0.013	0.013	0.013	0.013	0.013	0.013	0.013	0.013	1.658	1.878	3.584	4.876	1.734	3.049	0.788	2.547	2.726	2.045	#DIV/0!	#DIV/0!	0.009	0.027	0.009	0.029	0.009	0.022	0.009	0.022	0.917	0.917
Q924S5	Lon protease homolog, mitochondrial OS=Rattus norvegicus GN=Lonp1 PE=2 SV=1 - [LONM_RAT]	105.7	1.080	0.813	0.821	0.981	0.927	0.013	0.013	0.013	0.013	0.013	1.273	1.200	1.823	2.337	0.973	3.089	0.656	2.394	1.674	0.902	0.009	0.074	0.036	0.057	0.354	0.341	0.009	0.042	0.009	0.025	0.917	1.002
Q99MA2	Xaa-Pro aminopeptidase 2 OS=Rattus norvegicus OX=10116 GN=Xpnpep2 PE=1 SV=1 - [XPP2_RAT]	76.0	0.013	0.013	0.013	0.013	0.013	0.013	0.013	0.013	0.013	0.013	0.833	1.980	2.033	5.768	1.110	4.837	0.608	1.432	1.281	1.308	#DIV/0!	#DIV/0!	0.009	0.028	0.009	0.031	0.009	0.023	0.009	0.024	0.605	1.293
Q99MB4	COBW domain-containing protein 1 OS=Rattus norvegicus OX=10116 GN=Cbwd1 PE=2 SV=2 - [CBWD1_RAT]	43.9	0.013	0.013	0.013	0.013	0.013	0.013	0.013	0.013	0.013	0.013	1.737	2.093	2.947	2.278	1.744	2.704	0.331	2.120	1.818	1.716	#DIV/0!	#DIV/0!	0.009	0.030	0.009	0.033	0.009	0.024	0.009	0.025	0.470	1.381
Q99MI7	NEDD8-activating enzyme E1 catalytic subunit OS=Rattus norvegicus GN=Uba3 PE=1 SV=1 - [UBA3_RAT]	51.7	0.562	1.126	0.509	1.040	1.153	0.013	0.013	0.013	0.013	0.013	1.171	1.352	2.513	2.151	1.282	1.938	0.485	1.617	1.362	1.578	0.009	0.106	0.014	0.030	0.127	0.163	0.009	0.033	0.009	0.040	0.917	1.035
Q99N27	Sorting nexin-1 OS=Rattus norvegicus GN=Snx1 PE=1 SV=1 - [SNX1_RAT]	59.0	0.013	0.013	0.013	0.013	0.013	0.013	0.013	0.013	0.013	0.013	0.693	0.776	1.398	1.731	0.962	1.690	1.350	0.981	0.901	0.937	#DIV/0!	#DIV/0!	0.009	0.089	0.009	0.069	0.009	0.079	0.009	0.057	0.756	1.505
Q9EPB1	Dipeptidyl peptidase 2 OS=Rattus norvegicus GN=Dpp7 PE=1 SV=1 - [DPP2_RAT]	55.1	0.013	0.013	0.013	0.013	0.013	0.013	0.013	0.013	0.013	0.013	0.657	0.751	1.419	3.262	0.873	3.820	0.792	0.996	1.148	1.035	#DIV/0!	#DIV/0!	0.009	0.059	0.009	0.040	0.009	0.050	0.009	0.030	0.470	1.458
Q9EPC6	Profilin-2 OS=Rattus norvegicus GN=Pfn2 PE=1 SV=3 - [PROF2_RAT]	15.0	0.013	0.013	0.013	0.013	0.013	0.013	0.013	0.013	0.013	0.013	1.297	1.380	2.642	3.679	1.612	3.544	0.655	1.163	1.255	1.100	#DIV/0!	#DIV/0!	0.009	0.030	0.009	0.040	0.009	0.024	0.009	0.031	0.085	0.531
Q9EQV6	Tripeptidyl-peptidase 1 OS=Rattus norvegicus GN=Tpp1 PE=1 SV=1 - [TPP1_RAT]	61.3	0.013	0.013	0.013	0.013	0.013	0.013	0.013	0.013	0.013	0.013	1.209	1.073	2.385	4.701	0.923	3.831	0.669	1.893	1.483	1.098	#DIV/0!	#DIV/0!	0.009	0.031	0.009	0.032	0.009	0.025	0.009	0.025	0.917	0.933
Q9ERE4	Golgi phosphoprotein 3 OS=Rattus norvegicus GN=Golph3 PE=1 SV=1 - [GOLP3_RAT]	33.8	0.013	0.013	0.013	0.013	0.013	0.013	0.013	0.013	0.013	0.013	2.112	2.942	2.826	2.504	1.244	2.084	1.315	2.276	2.429	1.102	#DIV/0!	#DIV/0!	0.009	0.028	0.009	0.032	0.009	0.023	0.009	0.024	0.184	1.010
Q9ESG3	Collectrin OS=Rattus norvegicus OX=10116 GN=Cltrn PE=1 SV=2 - [CLTRN_RAT]	25.0	0.013	0.013	0.013	0.013	0.013	0.013	0.013	0.013	0.013	0.013	0.636	0.834	1.069	7.849	0.624	7.770	0.285	1.125	1.117	1.070	#DIV/0!	#DIV/0!	0.009	0.029	0.009	0.029	0.009	0.023	0.009	0.022	0.470	1.370
Q9ESS6	Basal cell adhesion molecule OS=Rattus norvegicus OX=10116 GN=Bcam PE=2 SV=1 - [BCAM_RAT]	67.5	0.013	0.013	0.013	0.013	0.013	0.013	0.013	0.013	0.013	0.013	1.036	1.024	1.693	2.669	0.896	2.482	0.937	1.358	1.171	1.110	#DIV/0!	#DIV/0!	0.009	0.052	0.009	0.048	0.009	0.045	0.009	0.038	0.756	1.274
Q9JI66	Electrogenic sodium bicarbonate cotransporter 1 OS=Rattus norvegicus GN=Slc4a4 PE=1 SV=1 - [S4A4_RAT]	121.3	0.013	0.013	0.013	0.013	0.013	0.013	0.013	0.013	0.013	0.013	1.172	1.005	1.890	6.525	0.980	4.901	0.772	1.550	1.877	1.159	#DIV/0!	#DIV/0!	0.009	0.028	0.009	0.030	0.009	0.023	0.009	0.023	0.917	0.921
Q9JIK1	Cadherin-related family member 5 OS=Rattus norvegicus GN=Cdhr5 PE=1 SV=1 - [CDHR5_RAT]	90.9	0.013	0.013	0.013	0.013	0.013	0.013	0.013	0.013	0.013	0.013	0.539	0.608	0.937	4.175	0.941	6.358	0.227	1.177	0.977	0.863	#DIV/0!	#DIV/0!	0.009	0.054	0.009	0.031	0.009	0.046	0.009	0.023	0.605	1.333
Q9JJ40	Na(+)/H(+) exchange regulatory cofactor NHE-RF3 OS=Rattus norvegicus OX=10116 GN=Pdzk1 PE=1 SV=2 - [NHRF3_RAT]	56.8	0.128	0.379	0.148	0.274	0.295	0.304	0.167	0.252	0.324	0.312	0.523	0.559	0.949	6.784	0.547	5.977	0.459	0.849	0.821	0.604	0.470	1.151	0.014	0.039	0.014	0.041	0.014	0.032	0.014	0.033	0.917	0.950
Q9QUL6	Vesicle-fusing ATPase OS=Rattus norvegicus GN=Nsf PE=1 SV=1 - [NSF_RAT]	82.6	1.606	0.939	1.002	1.312	1.048	0.013	0.013	0.013	0.013	0.013	0.013	0.013	0.013	0.013	0.013	2.238	0.013	3.671	1.577	1.981	0.009	0.052	0.009	0.077	0.184	0.204	#DIV/0!	#DIV/0!	0.025	0.055	0.025	0.161
Q9QWE9	Glutathione hydrolase 5 proenzyme OS=Rattus norvegicus OX=10116 GN=Ggt5 PE=2 SV=1 - [GGT5_RAT]	61.6	0.013	0.013	0.013	0.013	0.013	0.013	0.013	0.013	0.013	0.013	2.496	1.838	4.058	3.572	1.915	1.821	0.013	2.147	1.941	1.352	#DIV/0!	#DIV/0!	0.009	0.027	0.025	0.065	0.009	0.022	0.025	0.056	0.085	0.528
Q9QXN4	Inositol oxygenase OS=Rattus norvegicus OX=10116 GN=Miox PE=1 SV=2 - [MIOX_RAT]	33.2	0.013	0.013	0.013	0.013	0.013	0.013	0.013	0.013	0.013	0.013	0.516	0.563	0.802	8.002	0.597	3.754	0.643	0.957	0.953	0.841	#DIV/0!	#DIV/0!	0.009	0.031	0.009	0.046	0.009	0.024	0.009	0.036	0.184	1.014
Q9QYU4	Ketimine reductase mu-crystallin OS=Rattus norvegicus GN=Crym PE=1 SV=1 - [CRYM_RAT]	33.5	0.013	0.013	0.013	0.013	0.013	0.013	0.013	0.013	0.013	0.013	0.528	0.602	0.807	7.396	0.490	6.252	0.536	0.793	0.823	0.640	#DIV/0!	#DIV/0!	0.009	0.032	0.009	0.032	0.009	0.025	0.009	0.024	0.470	1.384
Q9QZ81	Protein argonaute-2 OS=Rattus norvegicus GN=Ago2 PE=2 SV=2 - [AGO2_RAT]	97.3	0.013	0.013	0.013	0.013	0.013	1.031	0.658	0.544	1.466	1.319	1.238	1.160	2.435	1.461	1.228	0.013	0.013	0.013	0.013	0.013	0.009	0.062	0.009	0.050	#DIV/0!	#DIV/0!	0.259	0.207	0.009	0.092	0.009	0.092
Q9QZA2	Programmed cell death 6-interacting protein OS=Rattus norvegicus GN=Pdcd6ip PE=1 SV=2 - [PDC6I_RAT]	96.6	0.681	0.922	0.587	0.999	0.950	1.193	0.529	0.721	0.861	1.068	1.079	1.092	2.154	1.879	1.233	1.739	0.863	1.294	1.219	1.201	0.756	0.984	0.014	0.034	0.056	0.114	0.036	0.052	0.036	0.072	0.605	1.469
Q9R1T5	Aspartoacylase OS=Rattus norvegicus OX=10116 GN=Aspa PE=1 SV=2 - [ACY2_RAT]	35.3	0.013	0.013	0.013	0.013	0.013	0.013	0.013	0.013	0.013	0.013	0.760	0.823	1.464	5.123	0.736	6.194	0.435	1.045	0.880	0.848	#DIV/0!	#DIV/0!	0.009	0.037	0.009	0.031	0.009	0.029	0.009	0.024	0.756	1.124
Q9R1Z0	Voltage-dependent anion-selective channel protein 3 OS=Rattus norvegicus OX=10116 GN=Vdac3 PE=1 SV=2 - [VDAC3_RAT]	30.8	0.530	1.483	0.371	1.899	1.355	0.830	0.355	0.661	1.744	1.444	0.013	0.013	0.013	0.013	0.013	0.013	0.013	0.013	0.013	0.013	0.756	0.898	0.009	0.082	0.009	0.077	0.009	0.117	0.009	0.091	#DIV/0!	#DIV/0!
Q9WTT6	Guanine deaminase OS=Rattus norvegicus GN=Gda PE=1 SV=1 - [GUAD_RAT]	51.0	0.492	0.956	0.467	0.852	0.856	0.829	0.351	0.597	0.878	0.983	0.984	1.038	2.127	1.086	1.133	1.245	0.839	1.307	1.211	1.316	0.917	1.051	0.014	0.040	0.056	0.127	0.014	0.032	0.036	0.078	0.470	1.653
Q9WUS0	Adenylate kinase 4, mitochondrial OS=Rattus norvegicus GN=Ak4 PE=2 SV=1 - [KAD4_RAT]	25.2	0.013	0.013	0.013	0.013	0.013	0.013	0.013	0.013	0.013	0.013	0.539	0.563	1.063	3.953	0.819	3.602	0.823	0.658	0.756	0.737	#DIV/0!	#DIV/0!	0.009	0.059	0.009	0.056	0.009	0.050	0.009	0.045	0.917	1.142
Q9WUW8	Sulfotransferase 1C2 OS=Rattus norvegicus OX=10116 GN=Sult1c2 PE=2 SV=1 - [ST1C2_RAT]	34.7	0.013	0.013	0.013	0.013	0.013	0.184	0.092	0.118	0.449	0.481	0.861	0.805	1.151	8.956	0.641	7.713	0.299	1.175	1.093	1.020	0.009	10.779	0.009	0.028	0.009	0.029	0.014	0.025	0.036	0.068	0.756	1.097
Q9WUW9	Sulfotransferase 1C2A OS=Rattus norvegicus OX=10116 GN=Sult1c2a PE=2 SV=2 - [S1C2A_RAT]	34.8	0.013	0.013	0.013	0.013	0.013	0.013	0.013	0.013	0.013	0.013	0.632	0.753	0.933	8.095	0.592	7.669	0.340	1.085	0.969	0.727	#DIV/0!	#DIV/0!	0.009	0.029	0.009	0.029	0.009	0.023	0.009	0.022	0.756	1.100
Q9WVS2	Probable tRNA N6-adenosine threonylcarbamoyltransferase OS=Rattus norvegicus GN=Osgep PE=2 SV=2 - [OSGEP_RAT]	36.3	0.013	0.013	0.013	0.013	0.013	0.013	0.013	0.013	0.013	0.013	0.818	1.015	1.966	1.603	0.967	2.505	0.819	1.821	1.266	1.043	#DIV/0!	#DIV/0!	0.009	0.067	0.009	0.043	0.009	0.059	0.009	0.034	0.470	1.510
Q9Z0T0	Thiopurine S-methyltransferase OS=Rattus norvegicus GN=Tpmt PE=2 SV=1 - [TPMT_RAT]	27.7	0.013	0.013	0.013	0.013	0.013	0.013	0.013	0.013	0.013	0.013	0.832	0.891	1.657	1.813	0.949	2.801	0.728	1.290	1.144	0.930	#DIV/0!	#DIV/0!	0.009	0.072	0.009	0.051	0.009	0.063	0.009	0.040	0.917	1.184
Q9Z2M4	Peroxisomal 2,4-dienoyl-CoA reductase OS=Rattus norvegicus OX=10116 GN=Decr2 PE=1 SV=1 - [DECR2_RAT]	31.3	0.013	0.013	0.013	0.013	0.013	0.013	0.013	0.013	0.013	0.013	0.595	0.708	1.236	3.118	0.783	2.991	0.700	1.067	0.851	0.881	#DIV/0!	#DIV/0!	0.009	0.065	0.009	0.057	0.009	0.058	0.009	0.046	0.756	1.385
Q9Z2Q1	Protein transport protein Sec31A OS=Rattus norvegicus GN=Sec31a PE=1 SV=2 - [SC31A_RAT]	135.2	0.756	1.019	0.569	1.059	0.899	1.385	0.513	0.727	0.864	0.801	1.194	1.411	2.130	1.440	1.260	1.419	0.973	1.788	1.486	0.900	0.605	0.992	0.014	0.033	0.085	0.126	0.036	0.052	0.036	0.071	0.756	1.300
Q9Z2Y0	Glycine N-acyltransferase-like protein Keg1 OS=Rattus norvegicus OX=10116 GN=Keg1 PE=1 SV=2 - [KEG1_RAT]	34.0	0.013	0.013	0.013	0.013	0.013	0.013	0.013	0.013	0.013	0.013	0.581	0.576	0.969	4.371	0.715	3.107	0.868	0.821	0.651	0.591	#DIV/0!	#DIV/0!	0.009	0.054	0.009	0.065	0.009	0.046	0.009	0.053	0.756	1.361
Q9Z336	Dynein light chain Tctex-type 1 OS=Rattus norvegicus GN=Dynlt1 PE=1 SV=1 - [DYLT1_RAT]	12.4	0.386	1.341	0.308	0.999	1.119	0.013	0.013	0.013	0.013	0.013	1.173	1.502	2.335	1.946	1.359	2.844	0.971	2.567	2.238	1.778	0.009	0.144	0.022	0.040	0.056	0.088	0.009	0.034	0.009	0.023	0.354	1.399

Table S3. Extracted protein profile of b-II (Effect of FGR, only Good nutrition, Male)

			Expression level																				p<0.05, q<0.10											
Accession	Description	MW [kDa]	FGR-Good-Male					FGR-Poor-Male					Sham-Good-Male					Sham-Poor-Male					Steel-Dwass & FDR											
			#21	#22	#23	#24	#25	#26	#27	#28	#29	#30	#31	#32	#33	#34	#35	#36	#37	#38	#39	#40	FGR-GN vs FGR-PN		FGR-GN vs Sham-GN		FGR-GN vs Sham-PN		FGR-PN vs Sham-GN		FGR-PN vs Sham-PN		Sham-GN vs Sham-PN	
Filter	Filter	Filter	Filter	Filter	Filter	Filter	Filter	Filter	Filter	Filter	Filter	Filter	Filter	Filter	Filter	Filter	Filter	Filter	Filter	Filter	Filter	Filter	p-value	q-value	p	q-value	p	q-value	p	q-value	p	q-value	p	q-value
A0MZ67	Shootin-1 OS=Rattus norvegicus OX=10116 GN=Shtn1 PE=1 SV=1 - [SHOT1_RAT]	71.4	0.013	0.013	0.013	0.013	0.013	0.013	0.013	0.013	0.013	0.013	0.979	1.099	0.989	1.394	0.628	0.013	0.013	0.013	0.013	0.013	#DIV/0!	#DIV/0!	0.009	0.030	#DIV/0!	#DIV/0!	0.009	0.047	#DIV/0!	#DIV/0!	0.009	0.043
A2VCW5	Sodium-coupled neutral amino acid transporter 5 OS=Rattus norvegicus OX=10116 GN=Slc38a5 PE=1 SV=1 - [S38A5_RAT]	52.4	0.013	0.013	0.013	0.013	0.013	0.952	1.223	1.227	0.360	1.163	0.508	0.969	0.917	1.827	0.127	0.422	0.339	0.558	0.349	1.011	0.009	0.0264	0.009	0.051	0.009	0.381	0.470	0.646	0.085	0.106	0.470	0.916
A9UMV8	Histone H2A.J OS=Rattus norvegicus GN=H2afj PE=2 SV=1 - [H2AJ_RAT]	14.0	0.441	0.335	0.540	0.222	0.251	0.745	0.840	1.043	0.155	1.282	0.761	0.807	1.170	2.210	0.348	0.818	0.379	0.662	0.256	0.772	0.127	0.244	0.036	0.091	0.127	0.477	0.756	0.698	0.259	0.212	0.259	0.709
B0BN18	Prefoldin subunit 2 OS=Rattus norvegicus GN=Pfdn2 PE=2 SV=1 - [PFD2_RAT]	16.6	0.013	0.013	0.013	0.013	0.013	0.816	1.021	1.164	0.222	0.956	0.505	0.771	1.178	1.769	0.167	1.147	0.810	1.710	0.788	0.897	0.009	0.0412	0.009	0.049	0.009	0.046	0.917	0.761	0.917	0.369	0.470	0.713
B0BN93	26S proteasome non-ATPase regulatory subunit 13 OS=Rattus norvegicus GN=Psmd13 PE=1 SV=1 - [PSD13_RAT]	42.8	0.013	0.013	0.013	0.013	0.013	0.013	0.013	0.013	0.013	0.013	0.479	0.964	0.833	1.433	0.258	0.013	0.013	0.013	0.013	0.013	#DIV/0!	#DIV/0!	0.009	0.088	#DIV/0!	#DIV/0!	0.009	0.110	#DIV/0!	#DIV/0!	0.009	0.109
B2GV24	E3 UFM1-protein ligase 1 OS=Rattus norvegicus GN=Ufl1 PE=1 SV=1 - [UFL1_RAT]	89.5	0.013	0.013	0.013	0.013	0.013	0.013	0.013	0.013	0.013	0.013	1.311	1.013	0.901	1.072	0.808	0.013	0.013	0.013	0.013	0.013	#DIV/0!	#DIV/0!	0.009	0.030	#DIV/0!	#DIV/0!	0.009	0.046	#DIV/0!	#DIV/0!	0.009	0.043
B2RYJ4	L-aminoadipate-semialdehyde dehydrogenase-phosphopantetheinyl transferase OS=Rattus norvegicus GN=Aasdhppt PE=2 SV=1 - [ADPPT_RAT]	35.8	0.689	0.599	2.123	1.042	0.753	0.013	0.013	0.013	0.013	0.013	0.013	0.013	0.013	0.013	0.013	0.013	0.013	0.013	0.013	0.013	0.009	0.0246	0.009	0.029	0.009	0.047	#DIV/0!	#DIV/0!	#DIV/0!	#DIV/0!	#DIV/0!	#DIV/0!
D3ZLZ7	Inosine-5'-monophosphate dehydrogenase 1 OS=Rattus norvegicus GN=Impdh1 PE=3 SV=1 - [IMDH1_RAT]	55.3	0.013	0.013	0.013	0.013	0.013	0.784	1.588	1.255	0.322	1.181	0.865	0.884	1.098	1.644	0.365	0.754	0.790	1.405	0.594	1.119	0.009	0.0250	0.009	0.034	0.009	0.054	0.917	0.718	0.605	0.296	0.756	0.730
D4AAT7	ATP-dependent (S)-NAD(P)H-hydrate dehydratase OS=Rattus norvegicus GN=Carkd PE=3 SV=1 - [NNRD_RAT]	36.5	0.013	0.013	0.013	0.013	0.013	0.013	0.013	0.013	0.013	0.013	0.409	0.973	1.327	1.650	0.155	0.379	0.491	0.675	0.490	0.864	#DIV/0!	#DIV/0!	0.009	0.042	0.009	0.274	0.009	0.064	0.009	0.142	0.470	0.891
F1LU71	Methylglutaconyl-CoA hydratase, mitochondrial OS=Rattus norvegicus OX=10116 GN=Auh PE=1 SV=2 - [AUHM_RAT]	33.3	0.013	0.013	0.013	0.013	0.013	0.866	1.251	1.113	0.172	0.983	0.635	1.083	1.086	1.077	0.502	0.487	0.806	1.123	0.852	1.064	0.009	0.0344	0.009	0.049	0.009	0.063	0.756	0.727	0.605	0.304	0.756	0.755
O35142	Coatomer subunit beta' OS=Rattus norvegicus GN=Copb2 PE=1 SV=3 - [COPB2_RAT]	102.5	0.013	0.013	0.013	0.013	0.013	0.667	0.779	0.930	0.774	0.697	0.903	0.653	1.206	1.115	0.228	0.456	0.481	1.029	0.652	1.060	0.009	0.0544	0.009	0.069	0.009	0.097	0.756	0.802	0.605	0.324	0.470	0.835
O35162	Heat shock 70 kDa protein 13 OS=Rattus norvegicus GN=Hspa13 PE=2 SV=2 - [HSP13_RAT]	51.8	0.013	0.013	0.013	0.013	0.013	0.857	1.016	0.877	0.276	1.022	0.540	0.978	0.803	1.506	0.214	0.567	0.540	0.845	0.470	0.984	0.009	0.0473	0.009	0.077	0.009	0.127	0.605	0.764	0.259	0.208	0.756	0.837
O70257	Syntaxin-7 OS=Rattus norvegicus GN=Stx7 PE=1 SV=4 - [STX7_RAT]	29.8	0.013	0.013	0.013	0.013	0.013	0.013	0.013	0.013	0.013	0.013	0.394	0.583	1.030	1.935	0.196	0.013	0.013	0.013	0.013	0.013	#DIV/0!	#DIV/0!	0.009	0.067	#DIV/0!	#DIV/0!	0.009	0.089	#DIV/0!	#DIV/0!	0.009	0.085
O70593	Small glutamine-rich tetratricopeptide repeat-containing protein alpha OS=Rattus norvegicus GN=Sgta PE=1 SV=1 - [SGTA_RAT]	34.1	0.724	0.831	1.617	0.490	0.674	0.735	0.790	1.129	0.085	0.611	0.013	0.013	0.013	0.013	0.013	0.657	1.015	1.419	1.063	1.025	0.756	0.560	0.009	0.053	0.354	0.718	0.009	0.295	0.184	0.161	0.009	0.041
O88775	Embigin OS=Rattus norvegicus GN=Emb PE=1 SV=1 - [EMB_RAT]	37.0	0.013	0.013	0.013	0.013	0.013	0.837	1.580	1.338	0.398	1.217	0.467	0.760	0.923	1.536	0.288	0.626	0.600	0.752	0.420	1.050	0.009	0.0236	0.009	0.084	0.009	0.125	0.354	0.600	0.184	0.158	0.756	0.840
O88807	Protein-arginine deiminase type-4 OS=Rattus norvegicus OX=10116 GN=Padi4 PE=2 SV=1 - [PADI4_RAT]	74.4	2.373	1.590	1.096	0.343	0.757	0.920	0.500	0.972	0.559	0.389	0.013	0.013	0.013	0.013	0.013	0.895	1.797	1.127	0.767	1.683	0.259	0.349	0.009	0.025	0.605	0.811	0.009	0.317	0.085	0.099	0.009	0.035
P01836	Ig kappa chain C region, A allele OS=Rattus norvegicus PE=1 SV=1 - [KACA_RAT]	11.7	0.772	1.211	0.773	0.359	0.288	0.439	0.539	0.530	2.196	2.196	0.180	0.318	0.287	0.333	0.183	0.196	0.254	0.306	0.205	0.760	0.470	0.432	0.036	0.091	0.056	0.242	0.014	0.055	0.056	0.076	0.756	0.892
P04644	40S ribosomal protein S17 OS=Rattus norvegicus GN=Rps17 PE=1 SV=3 - [RS17_RAT]	15.5	0.013	0.013	0.013	0.013	0.013	0.568	0.726	1.212	0.106	0.830	0.682	0.842	1.010	1.813	0.229	0.725	0.584	1.038	0.444	1.452	0.009	0.102	0.009	0.040	0.009	0.066	0.470	0.734	0.756	0.357	0.917	0.771
P05982	NAD(P)H dehydrogenase [quinone] 1 OS=Rattus norvegicus GN=Nqo1 PE=1 SV=4 - [NQO1_RAT]	30.9	0.013	0.013	0.013	0.013	0.013	0.013	0.013	0.013	0.013	0.013	0.929	1.020	1.150	1.430	0.489	0.013	0.013	0.013	0.013	0.013	#DIV/0!	#DIV/0!	0.009	0.031	#DIV/0!	#DIV/0!	0.009	0.047	#DIV/0!	#DIV/0!	0.009	0.044
P06238	Alpha-2-macroglobulin OS=Rattus norvegicus GN=A2m PE=2 SV=2 - [A2MG_RAT]	163.7	0.943	0.767	3.931	0.481	0.595	0.013	0.013	0.013	0.013	0.013	0.013	0.013	0.013	0.013	0.013	0.013	0.013	0.013	0.013	0.013	0.009	0.0220	0.009	0.024	0.009	0.041	#DIV/0!	#DIV/0!	#DIV/0!	#DIV/0!	#DIV/0!	#DIV/0!
P06866	Haptoglobin OS=Rattus norvegicus GN=Hp PE=1 SV=3 - [HPT_RAT]	38.5	1.594	1.029	0.800	0.656	0.745	0.705	1.242	0.809	1.628	0.960	0.415	0.470	0.470	0.515	0.351	0.303	1.352	1.003	0.590	1.074	0.470	0.423	0.014	0.036	0.756	0.858	0.014	0.055	0.605	0.297	0.127	0.435
P0C5W1	Microtubule-associated protein 1S OS=Rattus norvegicus GN=Map1s PE=1 SV=1 - [MAP1S_RAT]	102.7	0.013	0.013	0.013	0.013	0.013	0.013	0.013	0.013	0.013	0.013	1.640	1.252	0.603	0.787	1.017	0.013	0.013	0.013	0.013	0.013	#DIV/0!	#DIV/0!	0.009	0.028	#DIV/0!	#DIV/0!	0.009	0.043	#DIV/0!	#DIV/0!	0.009	0.039
P14562	Lysosome-associated membrane glycoprotein 1 OS=Rattus norvegicus GN=Lamp1 PE=1 SV=1 - [LAMP1_RAT]	43.9	0.013	0.013	0.013	0.013	0.013	0.629	0.918	0.874	0.351	0.759	0.816	0.960	0.817	1.294	0.498	0.490	0.625	0.871	0.543	1.040	0.009	0.0813	0.009	0.049	0.009	0.111	0.354	0.667	0.756	0.366	0.470	0.807
P15178	Aspartate--tRNA ligase, cytoplasmic OS=Rattus norvegicus GN=Dars PE=2 SV=1 - [SYDC_RAT]	57.1	0.013	0.013	0.013	0.013	0.013	0.589	0.577	1.046	0.053	0.568	1.024	1.000	1.030	1.463	0.513	0.752	0.360	1.315	0.510	1.232	0.009	0.265	0.009	0.031	0.009	0.069	0.259	0.611	0.470	0.285	0.470	0.724
P15800	Laminin subunit beta-2 OS=Rattus norvegicus GN=Lamb2 PE=2 SV=1 - [LAMB2_RAT]	196.3	1.161	0.740	1.715	0.778	0.688	0.013	0.013	0.013	0.013	0.013	0.013	0.013	0.013	0.013	0.013	0.013	0.013	0.013	0.013	0.013	0.009	0.0252	0.009	0.030	0.009	0.048	#DIV/0!	#DIV/0!	#DIV/0!	#DIV/0!	#DIV/0!	#DIV/0!
P16290	Phosphoglycerate mutase 2 OS=Rattus norvegicus GN=Pgam2 PE=2 SV=2 - [PGAM2_RAT]	28.7	1.462	0.967	1.225	0.451	0.400	1.018	1.524	0.664	0.196	0.882	0.013	0.013	0.013	0.013	0.013	0.766	1.280	0.973	0.604	1.190	0.917	0.568	0.009	0.043	0.756	0.853	0.009	0.077	0.756	0.331	0.009	0.047
P16303	Carboxylesterase 1D OS=Rattus norvegicus GN=Ces1d PE=1 SV=2 - [CES1D_RAT]	62.1	2.288	1.193	1.562	1.875	0.530	1.009	1.018	0.949	2.699	0.757	0.383	0.455	0.660	0.655	0.371	0.281	1.995	0.567	1.308	0.677	0.470	0.421	0.036	0.089	0.354	0.709	0.014	0.054	0.354	0.219	0.259	0.745
P17136	Small nuclear ribonucleoprotein-associated protein B OS=Rattus norvegicus OX=10116 GN=Snrpb PE=2 SV=2 - [RSMB_RAT]	23.6	0.013	0.013	0.013	0.013	0.013	0.013	0.013	0.013	0.013	0.013	0.836	1.149	1.445	2.290	0.416	0.013	0.013	0.013	0.013	0.013	#DIV/0!	#DIV/0!	0.009	0.025	#DIV/0!	#DIV/0!	0.009	0.038	#DIV/0!	#DIV/0!	0.009	0.035
P18163	Long-chain-fatty-acid--CoA ligase 1 OS=Rattus norvegicus OX=10116 GN=Acsl1 PE=1 SV=1 - [ACSL1_RAT]	78.1	0.013	0.013	0.013	0.013	0.013	0.570	1.230	1.050	0.242	1.095	0.805	0.940	1.002	1.396	0.367	0.596	0.761	1.048	0.626	0.943	0.009	0.0404	0.009	0.043	0.009	0.079	0.917	0.750	0.605	0.314	0.605	0.769
P18614	Integrin alpha-1 OS=Rattus norvegicus GN=Itga1 PE=1 SV=1 - [ITA1_RAT]	130.7	0.013	0.013	0.013	0.013	0.013	0.013	0.013	0.013	0.013	0.013	0.922	1.328	1.117	1.710	0.415	0.013	0.013	0.013	0.013	0.013	#DIV/0!	#DIV/0!	0.009	0.026	#DIV/0!	#DIV/0!	0.009	0.041	#DIV/0!	#DIV/0!	0.009	0.037
P20650	Protein phosphatase 1A OS=Rattus norvegicus GN=Ppm1a PE=1 SV=1 - [PPM1A_RAT]	42.4	0.013	0.013	0.013	0.013	0.013	0.013	0.013	0.013	0.013	0.013	0.860	1.046	1.007	1.224	0.645	0.013	0.013	0.013	0.013	0.013	#DIV/0!	#DIV/0!	0.009	0.035	#DIV/0!	#DIV/0!	0.009	0.052	#DIV/0!	#DIV/0!	0.009	0.048
P21396	Amine oxidase [flavin-containing] A OS=Rattus norvegicus GN=Maoa PE=1 SV=1 - [AOFA_RAT]	59.5	0.013	0.013	0.013	0.013	0.013	0.013	0.013	0.013	0.013	0.013	0.627	0.868	0.830	1.420	0.319	0.013	0.013	0.013	0.013	0.013	#DIV/0!	#DIV/0!	0.009	0.073	#DIV/0!	#DIV/0!	0.009	0.096	#DIV/0!	#DIV/0!	0.009	0.093
P22062	Protein-L-isoaspartate(D-aspartate) O-methyltransferase OS=Rattus norvegicus GN=Pcmt1 PE=1 SV=2 - [PIMT_RAT]	24.6	0.013	0.013	0.013	0.013	0.013	1.046	1.443	1.474	0.169	1.014	0.823	0.890	0.833	1.310	0.626	0.585	0.845	1.225	0.844	0.844	0.009	0.0249	0.009	0.045	0.009	0.063	0.259	0.524	0.259	0.184	0.917	0.769
P24050	40S ribosomal protein S5 OS=Rattus norvegicus GN=Rps5 PE=1 SV=3 - [RS5_RAT]	22.9	0.287	0.401	0.361	0.097	0.194	0.606	1.232	1.269	0.121	1.251	0.494	0.650	1.387	1.432	0.345	0.704	0.395	1.061	0.448	1.144	0.085	0.172	0.036	0.091	0.022	0.099	0.756	0.727	0.354	0.231	0.756	0.806
P24051	40S ribosomal protein S27-like OS=Rattus norvegicus GN=Rps27l PE=1 SV=3 - [RS27L_RAT]	9.5	0.013	0.013	0.013	0.013	0.013	0.770	0.965	1.446	0.268	1.021	0.936	1.139	1.117	1.019	0.427	0.592	0.713	1.228	0.744	1.097	0.009	0.0333	0.009	0.039	0.009	0.062	0.756	0.705	0.756	0.333	0.756	0.742
P25304	Agrin OS=Rattus norvegicus GN=Agrn PE=1 SV=2 - [AGRIN_RAT]	208.5	1.342	0.668	2.019	1.230	0.720	0.013	0.013	0.013	0.013	0.013	0.013	0.013	0.013	0.013	0.013	0.013	0.013	0.013	0.013	0.013	0.009	0.0223	0.009	0.025	0.009	0.043	#DIV/0!	#DIV/0!	#DIV/0!	#DIV/0!	#DIV/0!	#DIV/0!
P25886	60S ribosomal protein L29 OS=Rattus norvegicus OX=10116 GN=Rpl29 PE=1 SV=3 - [RL29_RAT]	17.3	0.013	0.013	0.013	0.013	0.013	0.772	0.722	1.192	0.285	0.653	1.127	0.942	0.840	1.470	0.517	0.889	0.857	1.419	0.757	1.217	0.009	0.0717	0.009	0.033	0.009	0.048	0.259	0.573	0.085	0.101	0.917	0.748
P26644	Beta-2-glycoprotein 1 OS=Rattus norvegicus GN=Apoh PE=2 SV=2 - [APOH_RAT]	33.2	0.013	0.013	0.013	0.013	0.013	0.834	1.209	1.002	0.306	1.185	0.768	1.000	0.858	1.276	0.500	0.641	0.885	1.059	0.541	1.198	0.009	0.0321	0.009	0.048	0.009	0.063	0.756	0.714	0.756	0.333	0.917	0.774
P27008	Poly [ADP-ribose] polymerase 1 OS=Rattus norvegicus GN=Parp1 PE=1 SV=4 - [PARP1_RAT]	112.6	0.013	0.013	0.013	0.013	0.013	0.013	0.013	0.013	0.013	0.013	1.025	1.766	0.967	1.031	0.570	0.013	0.013	0.013	0.013	0.013	#DIV/0!	#DIV/0!	0.009	0.027	#DIV/0!	#DIV/0!	0.009	0.042	#DIV/0!	#DIV/0!	0.009	0.038
P28023	Dynactin subunit 1 OS=Rattus norvegicus GN=Dctn1 PE=1 SV=2 - [DCTN1_RAT]	141.8	0.013	0.013	0.013	0.013	0.013	0.728	1.106	1.074	0.259	1.001	0.643	0.841	0.918	1.415	0.262	0.552	0.660	0.817	0.514	0.971	0.009	0.0418	0.009	0.070	0.009	0.115	0.756	0.780	0.259	0.205	0.605	0.837
P28648	CD63 antigen OS=Rattus norvegicus GN=Cd63 PE=1 SV=2 - [CD63_RAT]	25.7	0.013	0.013	0.013	0.013	0.013	0.013	0.013	0.013	0.013	0.013	1.433	1.144	0.863	1.941	0.367	0.013	0.013	0.013	0.013	0.013	#DIV/0!	#DIV/0!	0.009	0.026	#DIV/0!	#DIV/0!	0.009	0.039	#DIV/0!	#DIV/0!	0.009	0.036
P29418	ATP synthase subunit epsilon, mitochondrial OS=Rattus norvegicus OX=10116 GN=Atp5f1e PE=1 SV=2 - [ATP5E_RAT]	5.8	0.013	0.013	0.013	0.013	0.013	0.013	0.013	0.013	0.013	0.013	0.758	0.744	0.791	1.839	0.363	0.013	0.013	0.013	0.013	0.013	#DIV/0!	#DIV/0!	0.009	0.043	#DIV/0!	#DIV/0!	0.009	0.064	#DIV/0!	#DIV/0!	0.009	0.061
P29457	Serpin H1 OS=Rattus norvegicus GN=Serpinh1 PE=1 SV=1 - [SERPH_RAT]	46.5	0.013	0.013	0.013	0.013	0.013	0.013	0.013	0.013	0.013	0.013	0.941	1.047	2.508	1.349	0.462	0.013	0.013	0.013	0.013	0.013	#DIV/0!	#DIV/0!	0.009	0.024	#DIV/0!	#DIV/0!	0.009	0.037	#DIV/0!	#DIV/0!	0.009	0.034
P38650	Cytoplasmic dynein 1 heavy chain 1 OS=Rattus norvegicus GN=Dync1h1 PE=1 SV=1 - [DYHC1_RAT]	531.9	0.013	0.013	0.013	0.013	0.013	0.412	0.367	0.613	0.079	0.363	0.739	0.791	1.188	1.396	0.330	0.403	0.690	0.728	0.335	0.922	0.009	0.762	0.009	0.047	0.009	0.195	0.085	0.294	0.184	0.175	0.259	0.738
P39052	Dynamin-2 OS=Rattus norvegicus GN=Dnm2 PE=1 SV=1 - [DYN2_RAT]	98.2	0.013	0.013	0.013	0.013	0.013	0.013	0.013	0.013	0.013	0.013	0.690	0.885	0.868	1.480	0.367	0.457	0.462	0.773	0.452	0.842	#DIV/0!	#DIV/0!	0.009	0.055	0.009	0.230	0.009	0.076	0.009	0.118	0.259	0.752
P40329	Arginine--tRNA ligase, cytoplasmic OS=Rattus norvegicus GN=Rars PE=1 SV=2 - [SYRC_RAT]	75.8	0.013	0.013	0.013	0.013	0.013	0.013	0.013	0.013	0.013	0.013	1.077	0.983	1.187	1.469	0.503	0.013	0.013	0.013	0.013	0.013	#DIV/0!	#DIV/0!	0.009	0.028	#DIV/0!	#DIV/0!	0.009	0.044	#DIV/0!	#DIV/0!	0.009	0.040
P43424	Galactose-1-phosphate uridylyltransferase OS=Rattus norvegicus OX=10116 GN=Galt PE=2 SV=1 - [GALT_RAT]	43.3	0.013	0.013	0.013	0.013	0.013	0.013	0.013	0.013	0.013	0.013	0.963	0.865	0.868	0.894	1.094	0.013	0.013	0.013	0.013	0.013	#DIV/0!	#DIV/0!	0.009	0.037	#DIV/0!	#DIV/0!	0.009	0.054	#DIV/0!	#DIV/0!	0.009	0.052
P45592	Cofilin-1 OS=Rattus norvegicus GN=Cfl1 PE=1 SV=3 - [COF1_RAT]	18.5	0.524	0.604	0.596	0.258	0.651	0.722	0.993	1.398	0.160	0.721	0.652	0.733	1.330	2.002	0.647	0.673	0.752	0.932	0.859	0.810	0.127	0.241	0.022	0.058	0.014	0.061	0.756	0.699	0.917	0.383	0.756	0.732
P49242	40S ribosomal protein S3a OS=Rattus norvegicus GN=Rps3a PE=1 SV=2 - [RS3A_RAT]	29.9	0.013	0.013	0.013	0.013	0.013	0.664	0.839	1.217	0.177	0.896	0.841	1.221	1.139	0.945	0.457	0.629	0.578	1.136	0.584	1.098	0.009	0.0586	0.009	0.039	0.009	0.074	0.354	0.643	0.756	0.353	0.470	0.745
P52873	Pyruvate carboxylase, mitochondrial OS=Rattus norvegicus GN=Pc PE=1 SV=2 - [PYC_RAT]	129.7	0.013	0.013	0.013	0.013	0.013	0.435	0.652	0.757	0.126	0.769	0.806	0.869	1.047	1.322	0.638	0.013	0.013	0.013	0.013	0.013	0.009	0.290	0.009	0.037	#DIV/0!	#DIV/0!	0.056	0.201	0.009	0.185	0.009	0.052
P52944	PDZ and LIM domain protein 1 OS=Rattus norvegicus GN=Pdlim1 PE=1 SV=4 - [PDLI1_RAT]	35.6	0.013	0.013	0.013	0.013	0.013	0.013	0.013	0.013	0.013	0.013	1.108	1.128	1.111	1.764	0.440	0.013	0.013	0.013	0.013	0.013	#DIV/0!	#DIV/0!	0.009	0.026	#DIV/0!	#DIV/0!	0.009	0.041	#DIV/0!	#DIV/0!	0.009	0.037
P53534	Glycogen phosphorylase, brain form (Fragment) OS=Rattus norvegicus GN=Pygb PE=1 SV=3 - [PYGB_RAT]	96.1	0.013	0.013	0.013	0.013	0.013	0.599	1.263	1.223	0.192	1.287	0.801	0.942	0.931	1.388	0.416	0.561	0.639	0.952	0.528	1.092	0.009	0.0318	0.009	0.045	0.009	0.091	0.917	0.733	0.354	0.230	0.756	0.792
P54921	Alpha-soluble NSF attachment protein OS=Rattus norvegicus GN=Napa PE=1 SV=2 - [SNAA_RAT]	33.2	0.013	0.013	0.013	0.013	0.013	0.013	0.013	0.013	0.013	0.013	0.748	1.114	0.967	1.502	0.310	0.013	0.013	0.013	0.013	0.013	#DIV/0!	#DIV/0!	0.009	0.038	#DIV/0!	#DIV/0!	0.009	0.057	#DIV/0!	#DIV/0!	0.009	0.055
P58775	Tropomyosin beta chain OS=Rattus norvegicus GN=Tpm2 PE=3 SV=1 - [TPM2_RAT]	32.8	0.013	0.013	0.013	0.013	0.013	0.013	0.013	0.013	0.013	0.013	0.962	1.172	0.908	1.302	0.900	0.013	0.013	0.013	0.013	0.013	#DIV/0!	#DIV/0!	0.009	0.028	#DIV/0!	#DIV/0!	0.009	0.044	#DIV/0!	#DIV/0!	0.009	0.040
P60868	40S ribosomal protein S20 OS=Rattus norvegicus GN=Rps20 PE=3 SV=1 - [RS20_RAT]	13.4	0.294	0.362	1.143	0.128	0.427	0.812	0.877	1.344	0.203	0.793	1.145	0.996	1.406	1.508	0.557	0.930	0.450	1.789	0.610	0.929	0.259	0.386	0.036	0.090	0.085	0.344	0.184	0.458	0.605	0.304	0.354	0.752
P60892	Ribose-phosphate pyrophosphokinase 1 OS=Rattus norvegicus GN=Prps1 PE=1 SV=2 - [PRPS1_RAT]	34.8	0.013	0.013	0.013	0.013	0.013	0.631	1.414	1.018	0.214	1.378	0.562	0.809	1.305	1.296	0.297	0.461	0.455	0.547	0.332	1.042	0.009	0.0300	0.009	0.057	0.009	0.292	0.605	0.701	0.259	0.208	0.259	0.761
P62161	Calmodulin OS=Rattus norvegicus GN=Calm1 PE=1 SV=2 - [CALM_RAT]	16.8	0.013	0.013	0.013	0.013	0.013	1.476	1.676	1.764	0.077	1.143	0.223	0.589	1.465	2.235	0.028	0.353	0.592	0.291	0.156	0.937	0.009	0.0222	0.009	0.041	0.009	0.674	0.470	0.636	0.127	0.126	0.756	0.862
P62255	Ubiquitin-conjugating enzyme E2 G1 OS=Rattus norvegicus GN=Ube2g1 PE=2 SV=3 - [UB2G1_RAT]	19.5	0.013	0.013	0.013	0.013	0.013	0.013	0.013	0.013	0.013	0.013	0.664	0.966	0.843	1.239	0.258	0.013	0.013	0.013	0.013	0.013	#DIV/0!	#DIV/0!	0.009	0.085	#DIV/0!	#DIV/0!	0.009	0.106	#DIV/0!	#DIV/0!	0.009	0.107
P62268	40S ribosomal protein S23 OS=Rattus norvegicus GN=Rps23 PE=1 SV=3 - [RS23_RAT]	15.8	0.013	0.013	0.013	0.013	0.013	0.732	0.964	1.147	0.109	1.036	0.967	1.209	1.067	1.031	0.664	0.765	0.859	1.343	0.873	0.995	0.009	0.0496	0.009	0.032	0.009	0.052	0.470	0.666	0.756	0.334	0.605	0.727
P62282	40S ribosomal protein S11 OS=Rattus norvegicus GN=Rps11 PE=1 SV=3 - [RS11_RAT]	18.4	0.230	0.422	0.756	0.193	0.330	0.639	0.927	0.956	0.108	0.917	1.015	1.290	1.039	0.921	0.450	0.649	0.504	1.129	0.568	0.970	0.184	0.311	0.022	0.058	0.056	0.241	0.184	0.489	0.756	0.362	0.470	0.748
P62332	ADP-ribosylation factor 6 OS=Rattus norvegicus GN=Arf6 PE=1 SV=2 - [ARF6_RAT]	20.1	0.013	0.013	0.013	0.013	0.013	0.681	1.150	1.117	0.253	1.088	0.732	0.942	0.801	2.061	0.312	0.536	0.406	0.761	0.397	0.946	0.009	0.0361	0.009	0.034	0.009	0.208	0.917	0.729	0.259	0.209	0.470	0.811
P62850	40S ribosomal protein S24 OS=Rattus norvegicus GN=Rps24 PE=2 SV=1 - [RS24_RAT]	15.4	0.013	0.013	0.013	0.013	0.013	0.708	0.691	0.991	0.132	0.815	0.922	1.138	0.968	0.680	0.629	0.639	0.817	1.463	0.665	0.868	0.009	0.124	0.009	0.052	0.009	0.057	0.605	0.794	0.605	0.321	0.756	0.750
P62864	40S ribosomal protein S30 OS=Rattus norvegicus OX=10116 GN=Fau PE=1 SV=1 - [RS30_RAT]	6.6	0.013	0.013	0.013	0.013	0.013	1.232	0.830	1.338	0.160	0.768	0.824	1.080	1.794	1.064	0.466	0.599	1.023	1.576	0.719	1.233	0.009	0.0350	0.009	0.028	0.009	0.047	0.756	0.694	0.756	0.329	0.917	0.743
P62870	Transcription elongation factor B polypeptide 2 OS=Rattus norvegicus GN=Tceb2 PE=1 SV=1 - [ELOB_RAT]	13.2	0.013	0.013	0.013	0.013	0.013	0.013	0.013	0.013	0.013	0.013	0.871	1.010	0.969	1.541	0.410	0.013	0.013	0.013	0.013	0.013	#DIV/0!	#DIV/0!	0.009	0.035	#DIV/0!	#DIV/0!	0.009	0.051	#DIV/0!	#DIV/0!	0.009	0.048
P63326	40S ribosomal protein S10 OS=Rattus norvegicus GN=Rps10 PE=2 SV=1 - [RS10_RAT]	18.9	0.013	0.013	0.013	0.013	0.013	1.011	0.875	1.312	0.139	0.922	0.858	0.929	1.531	1.127	0.571	0.684	0.738	1.223	0.580	1.181	0.009	0.0372	0.009	0.031	0.009	0.060	0.756	0.701	0.756	0.336	0.756	0.731
P69897	Tubulin beta-5 chain OS=Rattus norvegicus GN=Tubb5 PE=1 SV=1 - [TBB5_RAT]	49.6	0.013	0.013	0.013	0.013	0.013	0.013	0.013	0.013	0.013	0.013	0.406	0.837	1.253	1.405	0.079	0.013	0.013	0.013	0.013	0.013	#DIV/0!	#DIV/0!	0.009	0.083	#DIV/0!	#DIV/0!	0.009	0.104	#DIV/0!	#DIV/0!	0.009	0.106
P70541	Translation initiation factor eIF-2B subunit gamma OS=Rattus norvegicus GN=Eif2b3 PE=2 SV=2 - [EI2BG_RAT]	50.4	0.013	0.013	0.013	0.013	0.013	0.013	0.013	0.013	0.013	0.013	0.979	1.283	0.801	1.581	0.404	0.013	0.013	0.013	0.013	0.013	#DIV/0!	#DIV/0!	0.009	0.031	#DIV/0!	#DIV/0!	0.009	0.047	#DIV/0!	#DIV/0!	0.009	0.044
P80432	Cytochrome c oxidase subunit 7C, mitochondrial OS=Rattus norvegicus OX=10116 GN=Cox7c PE=1 SV=2 - [COX7C_RAT]	7.4	0.013	0.013	0.013	0.013	0.013	0.657	0.766	1.112	0.307	0.550	0.801	0.773	1.073	1.323	0.515	0.649	0.808	1.429	0.673	0.866	0.009	0.111	0.009	0.044	0.009	0.058	0.259	0.610	0.259	0.202	0.917	0.766
P81718	Tyrosine-protein phosphatase non-receptor type 6 OS=Rattus norvegicus OX=10116 GN=Ptpn6 PE=1 SV=1 - [PTN6_RAT]	69.5	0.013	0.013	0.013	0.013	0.013	0.580	0.994	0.898	0.136	0.904	0.761	1.054	1.513	1.647	0.227	0.546	0.444	1.080	0.427	1.158	0.009	0.0824	0.009	0.029	0.009	0.101	0.259	0.562	0.917	0.395	0.470	0.735
P82808	Glutamine--fructose-6-phosphate aminotransferase [isomerizing] 1 OS=Rattus norvegicus GN=Gfpt1 PE=1 SV=3 - [GFPT1_RAT]	76.8	0.013	0.013	0.013	0.013	0.013	0.013	0.013	0.013	0.013	0.013	0.832	0.792	0.877	1.096	0.316	0.013	0.013	0.013	0.013	0.013	#DIV/0!	#DIV/0!	0.009	0.094	#DIV/0!	#DIV/0!	0.009	0.114	#DIV/0!	#DIV/0!	0.009	0.116
P83953	Importin subunit alpha-5 OS=Rattus norvegicus GN=Kpna1 PE=1 SV=1 - [IMA5_RAT]	60.1	0.871	0.805	1.516	0.541	0.492	0.013	0.013	0.013	0.013	0.013	0.013	0.013	0.013	0.013	0.013	0.013	0.013	0.013	0.013	0.013	0.009	0.0383	0.009	0.060	0.009	0.067	#DIV/0!	#DIV/0!	#DIV/0!	#DIV/0!	#DIV/0!	#DIV/0!
P85845	Fascin OS=Rattus norvegicus GN=Fscn1 PE=1 SV=2 - [FSCN1_RAT]	54.5	0.013	0.013	0.013	0.013	0.013	0.013	0.013	0.013	0.013	0.013	0.765	1.022	1.158	1.406	0.426	0.013	0.013	0.013	0.013	0.013	#DIV/0!	#DIV/0!	0.009	0.036	#DIV/0!	#DIV/0!	0.009	0.052	#DIV/0!	#DIV/0!	0.009	0.049
Q00715	Histone H2B type 1 OS=Rattus norvegicus PE=1 SV=2 - [H2B1_RAT]	14.0	0.013	0.013	0.013	0.013	0.013	0.748	0.893	1.024	0.319	0.914	0.583	0.773	0.963	1.528	0.266	0.349	0.915	0.944	0.782	1.033	0.009	0.0526	0.009	0.069	0.009	0.074	0.917	0.799	0.470	0.275	0.756	0.798
Q00729	Histone H2B type 1-A OS=Rattus norvegicus GN=Hist1h2ba PE=1 SV=2 - [H2B1A_RAT]	14.2	0.013	0.013	0.013	0.013	0.013	0.662	0.752	1.045	0.160	1.097	1.015	0.902	1.089	2.584	0.709	0.986	0.697	1.303	0.634	1.048	0.009	0.0656	0.009	0.024	0.009	0.053	0.354	0.589	0.605	0.309	0.470	0.707
Q02589	[Protein ADP-ribosylarginine] hydrolase OS=Rattus norvegicus GN=Adprh PE=1 SV=2 - [ADPRH_RAT]	39.9	0.891	0.823	1.503	0.362	0.602	0.013	0.013	0.013	0.013	0.013	0.013	0.013	0.013	0.013	0.013	0.013	0.013	0.013	0.013	0.013	0.009	0.0406	0.009	0.063	0.009	0.068	#DIV/0!	#DIV/0!	#DIV/0!	#DIV/0!	#DIV/0!	#DIV/0!
Q07116	Sulfite oxidase, mitochondrial OS=Rattus norvegicus GN=Suox PE=1 SV=2 - [SUOX_RAT]	60.8	0.824	0.624	1.632	0.992	0.713	0.645	0.601	0.792	0.205	0.532	0.013	0.013	0.013	0.013	0.013	0.574	0.959	1.330	0.898	0.830	0.056	0.117	0.009	0.035	0.917	0.856	0.009	0.658	0.056	0.077	0.009	0.057
Q07803	Elongation factor G, mitochondrial OS=Rattus norvegicus OX=10116 GN=Gfm1 PE=1 SV=2 - [EFGM_RAT]	83.4	0.802	0.678	1.263	0.674	0.634	0.715	0.915	1.039	0.397	0.845	0.013	0.013	0.013	0.013	0.013	0.438	0.728	0.958	0.576	0.881	0.605	0.514	0.009	0.074	0.756	0.918	0.009	0.116	0.756	0.360	0.009	0.200
Q2A121	Alpha-ketoglutarate-dependent dioxygenase FTO OS=Rattus norvegicus GN=Fto PE=2 SV=1 - [FTO_RAT]	57.9	0.013	0.013	0.013	0.013	0.013	0.955	1.172	0.997	0.196	0.997	0.629	0.920	1.067	1.284	0.346	0.449	0.892	0.877	0.651	1.005	0.009	0.0352	0.009	0.059	0.009	0.085	0.917	0.761	0.354	0.232	0.605	0.794
Q2LAP6	Testin OS=Rattus norvegicus GN=Tes PE=1 SV=1 - [TES_RAT]	47.6	0.013	0.013	0.013	0.013	0.013	0.487	1.296	0.917	0.098	1.260	1.225	1.066	1.398	2.496	0.409	0.749	0.279	1.225	0.320	1.039	0.009	0.0465	0.009	0.024	0.009	0.107	0.354	0.587	0.605	0.322	0.127	0.425
Q3B8Q0	Microtubule-associated protein RP/EB family member 2 OS=Rattus norvegicus GN=Mapre2 PE=2 SV=1 - [MARE2_RAT]	37.0	0.013	0.013	0.013	0.013	0.013	0.618	0.793	0.983	0.103	0.679	0.411	1.108	0.840	1.900	0.202	0.677	0.314	0.837	0.256	1.130	0.009	0.169	0.009	0.046	0.009	0.162	0.470	0.748	0.917	0.398	0.605	0.830
Q3B8Q2	Eukaryotic initiation factor 4A-III OS=Rattus norvegicus GN=Eif4a3 PE=1 SV=1 - [IF4A3_RAT]	46.8	0.013	0.013	0.013	0.013	0.013	0.013	0.013	0.013	0.013	0.013	0.681	0.927	1.065	1.469	0.308	0.013	0.013	0.013	0.013	0.013	#DIV/0!	#DIV/0!	0.009	0.046	#DIV/0!	#DIV/0!	0.009	0.067	#DIV/0!	#DIV/0!	0.009	0.064
Q4AEF8	Coatomer subunit gamma-1 OS=Rattus norvegicus GN=Copg1 PE=2 SV=1 - [COPG1_RAT]	97.6	0.013	0.013	0.013	0.013	0.013	0.698	1.036	1.110	0.356	1.085	0.667	0.988	1.078	1.243	0.346	0.425	0.532	0.802	0.485	1.093	0.009	0.0363	0.009	0.054	0.009	0.137	0.756	0.742	0.470	0.279	0.470	0.864
Q4FZU6	Annexin A8 OS=Rattus norvegicus GN=Anxa8 PE=1 SV=1 - [ANXA8_RAT]	36.7	1.061	1.084	1.606	0.926	0.756	0.013	0.013	0.013	0.013	0.013	0.013	0.013	0.013	0.013	0.013	0.013	0.013	0.013	0.013	0.013	0.009	0.0232	0.009	0.027	0.009	0.045	#DIV/0!	#DIV/0!	#DIV/0!	#DIV/0!	#DIV/0!	#DIV/0!
Q4FZX7	Signal recognition particle receptor subunit beta OS=Rattus norvegicus OX=10116 GN=Srprb PE=2 SV=1 - [SRPRB_RAT]	29.6	0.013	0.013	0.013	0.013	0.013	0.664	0.742	0.941	0.179	0.660	0.808	1.063	0.888	1.703	0.444	0.642	0.653	0.786	0.519	1.105	0.009	0.160	0.009	0.033	0.009	0.095	0.184	0.500	0.917	0.396	0.354	0.797
Q4KLF8	Actin-related protein 2/3 complex subunit 5 OS=Rattus norvegicus GN=Arpc5 PE=1 SV=3 - [ARPC5_RAT]	16.3	0.013	0.013	0.013	0.013	0.013	0.809	0.901	1.180	0.132	0.812	1.007	1.043	1.019	1.453	0.614	0.787	0.886	1.386	0.923	0.946	0.009	0.0554	0.009	0.029	0.009	0.049	0.259	0.544	0.354	0.224	0.354	0.757
Q4KLL0	Transcription elongation factor A protein 1 OS=Rattus norvegicus GN=Tcea1 PE=1 SV=1 - [TCEA1_RAT]	33.9	0.013	0.013	0.013	0.013	0.013	0.825	1.131	1.222	0.095	0.797	0.474	0.992	1.043	2.035	0.444	0.671	0.739	1.138	0.606	0.923	0.009	0.0452	0.009	0.032	0.009	0.072	0.917	0.732	0.605	0.315	0.917	0.764
Q4KM74	Vesicle-trafficking protein SEC22b OS=Rattus norvegicus GN=Sec22b PE=1 SV=3 - [SC22B_RAT]	24.7	0.013	0.013	0.013	0.013	0.013	0.501	0.867	1.028	0.171	0.567	0.809	1.018	1.173	1.406	0.389	0.548	0.734	1.144	1.684	0.761	0.009	0.191	0.009	0.035	0.009	0.051	0.259	0.604	0.259	0.198	0.756	0.727
Q4V8H8	EH domain-containing protein 2 OS=Rattus norvegicus GN=Ehd2 PE=1 SV=1 - [EHD2_RAT]	61.2	0.013	0.013	0.013	0.013	0.013	0.013	0.013	0.013	0.013	0.013	0.418	0.557	1.371	1.497	0.225	0.013	0.013	0.013	0.013	0.013	#DIV/0!	#DIV/0!	0.009	0.071	#DIV/0!	#DIV/0!	0.009	0.095	#DIV/0!	#DIV/0!	0.009	0.091
Q562C6	Leucine zipper transcription factor-like protein 1 OS=Rattus norvegicus OX=10116 GN=Lztfl1 PE=2 SV=1 - [LZTL1_RAT]	34.6	0.013	0.013	0.013	0.013	0.013	0.013	0.013	0.013	0.013	0.013	0.442	0.848	1.206	1.485	0.160	0.013	0.013	0.013	0.013	0.013	#DIV/0!	#DIV/0!	0.009	0.066	#DIV/0!	#DIV/0!	0.009	0.088	#DIV/0!	#DIV/0!	0.009	0.084
Q562C9	1,2-dihydroxy-3-keto-5-methylthiopentene dioxygenase OS=Rattus norvegicus OX=10116 GN=Adi1 PE=2 SV=1 - [MTND_RAT]	21.4	0.013	0.013	0.013	0.013	0.013	0.932	1.084	1.328	0.254	0.858	0.532	0.809	1.092	1.539	0.212	0.731	0.820	1.253	0.761	1.025	0.009	0.0337	0.009	0.063	0.009	0.055	0.756	0.740	0.605	0.300	0.756	0.752
Q5HZE4	Methylthioribose-1-phosphate isomerase OS=Rattus norvegicus GN=Mri1 PE=2 SV=1 - [MTNA_RAT]	39.6	0.013	0.013	0.013	0.013	0.013	0.013	0.013	0.013	0.013	0.013	1.030	1.137	0.993	1.562	0.560	0.013	0.013	0.013	0.013	0.013	#DIV/0!	#DIV/0!	0.009	0.028	#DIV/0!	#DIV/0!	0.009	0.043	#DIV/0!	#DIV/0!	0.009	0.040
Q5PQL8	Protein FAM149B1 OS=Rattus norvegicus OX=10116 GN=Fam149b1 PE=2 SV=1 - [F149B_RAT]	53.2	1.349	1.207	1.115	0.659	0.710	0.983	0.411	0.967	0.289	0.311	0.013	0.013	0.013	0.013	0.013	0.657	1.305	1.297	0.926	1.262	0.085	0.169	0.009	0.031	0.756	0.834	0.009	0.535	0.085	0.102	0.009	0.038
Q5QE80	Aldehyde oxidase 3 OS=Rattus norvegicus GN=Aox3 PE=1 SV=1 - [AOXC_RAT]	146.7	1.486	0.879	1.790	0.721	0.339	0.013	0.013	0.013	0.013	0.013	0.013	0.013	0.013	0.013	0.013	0.013	0.013	0.013	0.013	0.013	0.009	0.0245	0.009	0.028	0.009	0.047	#DIV/0!	#DIV/0!	#DIV/0!	#DIV/0!	#DIV/0!	#DIV/0!
Q5RJR2	Twinfilin-1 OS=Rattus norvegicus GN=Twf1 PE=2 SV=1 - [TWF1_RAT]	40.1	0.013	0.013	0.013	0.013	0.013	0.790	0.984	1.212	0.137	0.849	0.509	1.012	1.022	1.340	0.280	0.550	0.664	1.018	0.640	0.995	0.009	0.0508	0.009	0.065	0.009	0.086	0.605	0.760	0.756	0.353	0.756	0.808
Q5RK09	Eukaryotic translation initiation factor 3 subunit G OS=Rattus norvegicus GN=Eif3g PE=1 SV=1 - [EIF3G_RAT]	35.6	0.013	0.013	0.013	0.013	0.013	0.696	1.057	1.308	0.192	0.865	0.735	0.910	0.937	1.707	0.237	0.769	0.424	1.065	0.360	1.372	0.009	0.0432	0.009	0.041	0.009	0.077	0.756	0.739	0.917	0.382	0.917	0.779
Q5SGE0	Leucine-rich PPR motif-containing protein, mitochondrial OS=Rattus norvegicus GN=Lrpprc PE=1 SV=1 - [LPPRC_RAT]	156.6	0.013	0.013	0.013	0.013	0.013	0.013	0.013	0.013	0.013	0.013	0.993	1.035	0.734	1.110	0.395	0.324	0.354	0.566	0.239	0.730	#DIV/0!	#DIV/0!	0.009	0.058	0.009	0.876	0.009	0.078	0.009	0.473	0.036	0.130
Q5U209	Ribonuclease H2 subunit A OS=Rattus norvegicus OX=10116 GN=Rnaseh2a PE=2 SV=1 - [RNH2A_RAT]	33.3	1.253	0.748	1.614	0.853	0.692	0.013	0.013	0.013	0.013	0.013	0.013	0.013	0.013	0.013	0.013	0.013	0.013	0.013	0.013	0.013	0.009	0.0247	0.009	0.029	0.009	0.047	#DIV/0!	#DIV/0!	#DIV/0!	#DIV/0!	#DIV/0!	#DIV/0!
Q5XHZ0	Heat shock protein 75 kDa, mitochondrial OS=Rattus norvegicus GN=Trap1 PE=1 SV=1 - [TRAP1_RAT]	80.4	0.013	0.013	0.013	0.013	0.013	0.692	1.475	0.836	0.256	1.978	0.870	0.969	0.875	1.325	0.482	0.314	0.630	0.747	0.546	0.798	0.009	0.0241	0.009	0.042	0.009	0.225	0.917	0.720	0.259	0.195	0.085	0.301
Q5XIP1	Protein pelota homolog OS=Rattus norvegicus GN=Pelo PE=1 SV=1 - [PELO_RAT]	43.4	0.013	0.013	0.013	0.013	0.013	0.013	0.013	0.013	0.013	0.013	1.217	1.442	2.141	1.498	0.496	0.013	0.013	0.013	0.013	0.013	#DIV/0!	#DIV/0!	0.009	0.024	#DIV/0!	#DIV/0!	0.009	0.037	#DIV/0!	#DIV/0!	0.009	0.034
Q5XIU9	Membrane-associated progesterone receptor component 2 OS=Rattus norvegicus OX=10116 GN=Pgrmc2 PE=1 SV=1 - [PGRC2_RAT]	23.4	0.013	0.013	0.013	0.013	0.013	0.013	0.013	0.013	0.013	0.013	1.330	1.191	0.879	1.754	0.653	0.013	0.013	0.013	0.013	0.013	#DIV/0!	#DIV/0!	0.009	0.025	#DIV/0!	#DIV/0!	0.009	0.039	#DIV/0!	#DIV/0!	0.009	0.036
Q62639	GTP-binding protein Rheb OS=Rattus norvegicus OX=10116 GN=Rheb PE=1 SV=1 - [RHEB_RAT]	20.5	0.013	0.013	0.013	0.013	0.013	0.013	0.013	0.013	0.013	0.013	0.524	0.837	0.857	1.541	0.209	0.441	0.388	0.490	0.270	0.888	#DIV/0!	#DIV/0!	0.009	0.086	0.009	0.486	0.009	0.107	0.009	0.284	0.354	0.882

Q62862	Dual specificity mitogen-activated protein kinase kinase 5 OS=Rattus norvegicus OX=10116 GN=Map2k5 PE=1 SV=1 - [MP2K5_RAT]	50.2	1.737	0.695	2.138	2.366	1.043	1.109	0.451	0.564	2.615	0.351	0.890	1.006	0.667	0.677	0.662	0.501	1.487	1.128	0.953	0.810	0.259	0.345	0.036	0.089	0.184	0.515	0.605	0.696	0.470	0.259	0.354	0.788
Q62896	BET1 homolog OS=Rattus norvegicus OX=10116 GN=Bet1 PE=1 SV=1 - [BET1_RAT]	13.2	0.013	0.013	0.013	0.013	0.013	0.013	0.013	0.013	0.013	0.013	0.673	0.995	0.954	1.847	0.198	0.013	0.013	0.013	0.013	0.013	#DIV/0!	#DIV/0!	0.009	0.037	#DIV/0!	#DIV/0!	0.009	0.055	#DIV/0!	#DIV/0!	0.009	0.053
Q63092	CaM kinase-like vesicle-associated protein OS=Rattus norvegicus GN=Camkv PE=1 SV=1 - [CAMKV_RAT]	54.1	0.013	0.013	0.013	0.013	0.013	0.013	0.013	0.013	0.013	0.013	2.334	0.810	0.498	0.719	1.944	0.013	0.013	0.013	0.013	0.013	#DIV/0!	#DIV/0!	0.009	0.024	#DIV/0!	#DIV/0!	0.009	0.038	#DIV/0!	#DIV/0!	0.009	0.035
Q63135	Complement component receptor 1-like protein OS=Rattus norvegicus GN=Cr1l PE=1 SV=1 - [CR1L_RAT]	61.6	0.013	0.013	0.013	0.013	0.013	0.013	0.013	0.013	0.013	0.013	0.750	1.003	0.859	1.491	0.384	0.013	0.013	0.013	0.013	0.013	#DIV/0!	#DIV/0!	0.009	0.044	#DIV/0!	#DIV/0!	0.009	0.065	#DIV/0!	#DIV/0!	0.009	0.062
Q63159	Ubiquinone biosynthesis O-methyltransferase, mitochondrial OS=Rattus norvegicus OX=10116 GN=Coq3 PE=2 SV=2 - [COQ3_RAT]	38.7	0.013	0.013	0.013	0.013	0.013	0.013	0.013	0.013	0.013	0.013	0.698	1.092	0.897	1.013	0.645	0.013	0.013	0.013	0.013	0.013	#DIV/0!	#DIV/0!	0.009	0.052	#DIV/0!	#DIV/0!	0.009	0.072	#DIV/0!	#DIV/0!	0.009	0.070
Q63347	26S protease regulatory subunit 7 OS=Rattus norvegicus GN=Psmc2 PE=1 SV=3 - [PRS7_RAT]	48.5	0.013	0.013	0.013	0.013	0.013	0.762	0.932	1.284	0.261	0.860	0.901	1.122	0.948	1.249	0.450	0.544	0.497	1.091	0.558	0.982	0.009	0.0445	0.009	0.037	0.009	0.098	0.470	0.678	0.756	0.355	0.470	0.760
Q63362	NADH dehydrogenase [ubiquinone] 1 alpha subcomplex subunit 5 OS=Rattus norvegicus OX=10116 GN=Ndufa5 PE=1 SV=3 - [NDUA5_RAT]	13.4	0.013	0.013	0.013	0.013	0.013	0.648	0.846	1.219	0.180	0.724	0.793	0.830	1.057	1.801	0.405	0.783	0.328	1.473	0.314	1.103	0.009	0.0726	0.009	0.033	0.009	0.075	0.470	0.694	0.756	0.358	0.470	0.733
Q63486	Ras-related GTP-binding protein A OS=Rattus norvegicus GN=Rraga PE=1 SV=1 - [RRAGA_RAT]	36.5	0.013	0.013	0.013	0.013	0.013	0.013	0.013	0.013	0.013	0.013	0.498	0.717	0.978	1.254	0.497	0.013	0.013	0.013	0.013	0.013	#DIV/0!	#DIV/0!	0.009	0.091	#DIV/0!	#DIV/0!	0.009	0.111	#DIV/0!	#DIV/0!	0.009	0.111
Q63570	26S protease regulatory subunit 6B OS=Rattus norvegicus GN=Psmc4 PE=1 SV=1 - [PRS6B_RAT]	47.4	0.237	0.387	0.432	0.221	0.280	0.793	1.242	1.459	0.242	1.305	0.507	0.822	1.040	1.534	0.335	0.298	0.539	0.556	0.315	0.769	0.056	0.118	0.036	0.091	0.085	0.354	0.756	0.701	0.127	0.136	0.184	0.593
Q63610	Tropomyosin alpha-3 chain OS=Rattus norvegicus GN=Tpm3 PE=1 SV=2 - [TPM3_RAT]	29.0	0.013	0.013	0.013	0.013	0.013	0.758	1.154	1.234	0.134	0.718	1.091	1.291	1.273	1.838	0.589	1.130	1.361	1.707	1.010	1.153	0.009	0.0488	0.009	0.025	0.009	0.042	0.184	0.454	0.184	0.155	0.917	0.738
Q63644	Rho-associated protein kinase 1 OS=Rattus norvegicus GN=Rock1 PE=1 SV=1 - [ROCK1_RAT]	159.5	0.719	0.517	1.961	1.268	0.840	0.013	0.013	0.013	0.013	0.013	0.013	0.013	0.013	0.013	0.013	0.013	0.013	0.013	0.013	0.013	0.009	0.0238	0.009	0.027	0.009	0.046	#DIV/0!	#DIV/0!	#DIV/0!	#DIV/0!	#DIV/0!	#DIV/0!
Q63663	Guanylate-binding protein 1 OS=Rattus norvegicus GN=Gbp2 PE=1 SV=2 - [GBP2_RAT]	67.1	0.013	0.013	0.013	0.013	0.013	0.013	0.013	0.013	0.013	0.013	0.612	1.032	1.408	1.631	0.247	0.013	0.013	0.013	0.013	0.013	#DIV/0!	#DIV/0!	0.009	0.032	#DIV/0!	#DIV/0!	0.009	0.048	#DIV/0!	#DIV/0!	0.009	0.045
Q63862	Myosin-11 (Fragments) OS=Rattus norvegicus GN=Myh11 PE=1 SV=3 - [MYH11_RAT]	152.4	0.013	0.013	0.013	0.013	0.013	0.013	0.013	0.013	0.013	0.013	0.983	1.190	1.034	1.270	0.714	0.013	0.013	0.013	0.013	0.013	#DIV/0!	#DIV/0!	0.009	0.029	#DIV/0!	#DIV/0!	0.009	0.045	#DIV/0!	#DIV/0!	0.009	0.041
Q64119	Myosin light polypeptide 6 OS=Rattus norvegicus GN=Myl6 PE=1 SV=3 - [MYL6_RAT]	17.0	0.013	0.013	0.013	0.013	0.013	0.013	0.013	0.013	0.013	0.013	0.283	0.623	1.007	2.306	0.491	0.013	0.013	0.013	0.013	0.013	#DIV/0!	#DIV/0!	0.009	0.036	#DIV/0!	#DIV/0!	0.009	0.053	#DIV/0!	#DIV/0!	0.009	0.051
Q64537	Calpain small subunit 1 OS=Rattus norvegicus GN=Capns1 PE=1 SV=3 - [CPNS1_RAT]	28.6	0.013	0.013	0.013	0.013	0.013	0.013	0.013	0.013	0.013	0.013	0.680	1.061	1.035	1.291	0.453	0.013	0.013	0.013	0.013	0.013	#DIV/0!	#DIV/0!	0.009	0.042	#DIV/0!	#DIV/0!	0.009	0.063	#DIV/0!	#DIV/0!	0.009	0.060
Q66H68	RNA-binding protein 47 OS=Rattus norvegicus GN=Rbm47 PE=2 SV=1 - [RBM47_RAT]	64.1	0.013	0.013	0.013	0.013	0.013	0.013	0.013	0.013	0.013	0.013	0.759	0.981	0.868	1.270	0.505	0.013	0.013	0.013	0.013	0.013	#DIV/0!	#DIV/0!	0.009	0.050	#DIV/0!	#DIV/0!	0.009	0.070	#DIV/0!	#DIV/0!	0.009	0.068
Q66H74	Golgi phosphoprotein 3-like OS=Rattus norvegicus GN=Golph3l PE=2 SV=1 - [GLP3L_RAT]	32.9	0.013	0.013	0.013	0.013	0.013	0.013	0.013	0.013	0.013	0.013	0.904	1.306	1.080	1.327	0.593	0.013	0.013	0.013	0.013	0.013	#DIV/0!	#DIV/0!	0.009	0.028	#DIV/0!	#DIV/0!	0.009	0.044	#DIV/0!	#DIV/0!	0.009	0.040
Q68FU1	Pleckstrin homology domain-containing family F member 1 OS=Rattus norvegicus OX=10116 GN=Plekhf1 PE=2 SV=1 - [PKHF1_RAT]	31.4	1.554	0.896	2.552	2.218	1.053	0.013	0.013	0.013	0.013	0.013	0.013	0.013	0.013	0.013	0.013	0.013	0.013	0.013	0.013	0.013	0.009	0.0213	0.009	0.024	0.009	0.041	#DIV/0!	#DIV/0!	#DIV/0!	#DIV/0!	#DIV/0!	#DIV/0!
Q6AY20	Cation-dependent mannose-6-phosphate receptor OS=Rattus norvegicus OX=10116 GN=M6pr PE=1 SV=1 - [MPRD_RAT]	31.1	0.013	0.013	0.013	0.013	0.013	0.644	0.789	0.914	0.575	0.671	1.019	0.998	0.736	0.934	0.639	0.550	0.772	0.870	0.602	0.924	0.009	0.0735	0.009	0.053	0.009	0.094	0.184	0.506	0.917	0.394	0.184	0.581
Q6AY30	Saccharopine dehydrogenase-like oxidoreductase OS=Rattus norvegicus OX=10116 GN=Sccpdh PE=1 SV=1 - [SCPDL_RAT]	47.1	0.013	0.013	0.013	0.013	0.013	0.587	1.215	0.854	0.452	1.110	0.643	1.208	0.568	1.491	0.266	0.538	0.344	0.674	0.313	1.637	0.009	0.0388	0.009	0.064	0.009	0.118	0.917	0.775	0.354	0.240	0.756	0.828
Q6AY65	Arfaptin-2 OS=Rattus norvegicus GN=Arfip2 PE=2 SV=1 - [ARFP2_RAT]	37.7	0.013	0.013	0.013	0.013	0.013	0.013	0.013	0.013	0.013	0.013	0.916	1.111	0.926	1.339	0.574	0.013	0.013	0.013	0.013	0.013	#DIV/0!	#DIV/0!	0.009	0.034	#DIV/0!	#DIV/0!	0.009	0.049	#DIV/0!	#DIV/0!	0.009	0.047
Q6AYG3	Protein prune homolog OS=Rattus norvegicus GN=Prune PE=2 SV=1 - [PRUNE_RAT]	50.0	0.013	0.013	0.013	0.013	0.013	0.774	1.232	1.373	0.014	0.773	0.630	0.991	0.871	1.416	0.297	0.548	0.583	0.929	0.719	0.839	0.009	0.0173	0.009	0.061	0.009	0.105	0.917	0.777	0.470	0.277	0.470	0.826
Q6AYQ4	Transmembrane protein 109 OS=Rattus norvegicus OX=10116 GN=Tmem109 PE=2 SV=1 - [TM109_RAT]	26.2	0.013	0.013	0.013	0.013	0.013	0.668	1.740	0.990	0.254	1.032	1.166	1.403	0.643	1.756	0.489	0.880	0.541	1.060	0.499	1.453	0.009	0.0289	0.009	0.026	0.009	0.058	0.605	0.673	0.917	0.370	0.605	0.726
Q6AZ50	Ubiquitin-like-conjugating enzyme ATG3 OS=Rattus norvegicus GN=Atg3 PE=2 SV=1 - [ATG3_RAT]	35.8	0.013	0.013	0.013	0.013	0.013	0.681	1.148	1.458	0.148	1.035	0.574	0.997	1.100	1.950	0.301	0.614	0.426	0.726	0.413	0.913	0.009	0.0331	0.009	0.033	0.009	0.186	0.917	0.724	0.259	0.207	0.354	0.826
Q6GX84	Fidgetin-like protein 1 OS=Rattus norvegicus GN=Fignl1 PE=2 SV=1 - [FIGL1_RAT]	74.2	0.013	0.013	0.013	0.013	0.013	0.013	0.013	0.013	0.013	0.013	0.973	1.154	0.806	1.242	0.438	0.013	0.013	0.013	0.013	0.013	#DIV/0!	#DIV/0!	0.009	0.039	#DIV/0!	#DIV/0!	0.009	0.059	#DIV/0!	#DIV/0!	0.009	0.056
Q6IFU7	Keratin, type I cytoskeletal 42 OS=Rattus norvegicus GN=Krt42 PE=3 SV=1 - [K1C42_RAT]	50.2	0.013	0.013	0.013	0.013	0.013	0.013	0.013	0.013	0.013	0.013	0.770	0.818	5.742	0.882	0.581	0.013	0.013	0.013	0.013	0.013	#DIV/0!	#DIV/0!	0.009	0.023	#DIV/0!	#DIV/0!	0.009	0.036	#DIV/0!	#DIV/0!	0.009	0.033
Q6IFU8	Keratin, type I cytoskeletal 17 OS=Rattus norvegicus GN=Krt17 PE=2 SV=1 - [K1C17_RAT]	48.1	0.013	0.013	0.013	0.013	0.013	0.013	0.013	0.013	0.013	0.013	0.678	0.535	8.010	0.595	0.541	0.013	0.013	0.013	0.013	0.013	#DIV/0!	#DIV/0!	0.009	0.023	#DIV/0!	#DIV/0!	0.009	0.036	#DIV/0!	#DIV/0!	0.009	0.033
Q6P6Q2	Keratin, type II cytoskeletal 5 OS=Rattus norvegicus GN=Krt5 PE=1 SV=1 - [K2C5_RAT]	61.8	0.013	0.013	0.013	0.013	0.013	0.013	0.013	0.013	0.013	0.013	0.571	0.958	1.191	0.860	0.306	0.013	0.013	0.013	0.013	0.013	#DIV/0!	#DIV/0!	0.009	0.098	#DIV/0!	#DIV/0!	0.009	0.122	#DIV/0!	#DIV/0!	0.009	0.121
Q6P7P5	Basic leucine zipper and W2 domain-containing protein 1 OS=Rattus norvegicus GN=Bzw1 PE=2 SV=1 - [BZW1_RAT]	48.0	0.013	0.013	0.013	0.013	0.013	0.013	0.013	0.013	0.013	0.013	0.455	1.025	1.702	2.016	0.399	0.013	0.013	0.013	0.013	0.013	#DIV/0!	#DIV/0!	0.009	0.026	#DIV/0!	#DIV/0!	0.009	0.040	#DIV/0!	#DIV/0!	0.009	0.037
Q6PCT3	Tumor protein D54 OS=Rattus norvegicus GN=Tpd52l2 PE=2 SV=1 - [TPD54_RAT]	24.0	0.013	0.013	0.013	0.013	0.013	0.554	0.783	1.003	0.551	0.594	1.084	1.137	0.755	1.941	0.381	0.803	0.565	1.123	0.502	1.122	0.009	0.0910	0.009	0.027	0.009	0.071	0.259	0.553	0.470	0.279	0.605	0.734
Q6PDU1	Serine/arginine-rich splicing factor 2 OS=Rattus norvegicus GN=Srsf2 PE=1 SV=3 - [SRSF2_RAT]	25.5	0.013	0.013	0.013	0.013	0.013	0.013	0.013	0.013	0.013	0.013	0.993	1.013	1.526	1.775	0.548	0.013	0.013	0.013	0.013	0.013	#DIV/0!	#DIV/0!	0.009	0.025	#DIV/0!	#DIV/0!	0.009	0.039	#DIV/0!	#DIV/0!	0.009	0.036
Q71UF4	Histone-binding protein RBBP7 OS=Rattus norvegicus GN=Rbbp7 PE=2 SV=1 - [RBBP7_RAT]	47.8	0.749	0.847	1.636	0.630	0.665	0.654	0.741	1.031	0.333	0.684	0.013	0.013	0.013	0.013	0.013	0.624	0.952	1.516	1.149	1.062	0.470	0.465	0.009	0.041	0.470	0.757	0.009	0.244	0.127	0.124	0.009	0.039
Q75WE7	von Willebrand factor A domain-containing protein 5A OS=Rattus norvegicus GN=Vwa5a PE=2 SV=1 - [VWA5A_RAT]	91.4	0.860	0.635	1.688	0.887	0.759	0.013	0.013	0.013	0.013	0.013	0.013	0.013	0.013	0.013	0.013	0.347	0.612	0.765	0.464	0.723	0.009	0.0275	0.009	0.034	0.056	0.236	#DIV/0!	#DIV/0!	0.009	0.137	0.009	0.409
Q7TT51	Lengsin OS=Rattus norvegicus OX=10116 GN=Lgsn PE=2 SV=1 - [LGSN_RAT]	62.2	0.013	0.013	0.013	0.013	0.013	0.013	0.013	0.013	0.013	0.013	0.728	1.356	1.242	1.193	0.176	0.013	0.013	0.013	0.013	0.013	#DIV/0!	#DIV/0!	0.009	0.036	#DIV/0!	#DIV/0!	0.009	0.053	#DIV/0!	#DIV/0!	0.009	0.051
Q8CGS4	Charged multivesicular body protein 3 OS=Rattus norvegicus GN=Chmp3 PE=1 SV=3 - [CHMP3_RAT]	25.0	0.013	0.013	0.013	0.013	0.013	0.512	0.636	1.102	0.205	0.681	0.887	0.908	1.043	1.509	0.468	0.672	0.743	1.153	0.713	0.928	0.009	0.185	0.009	0.034	0.009	0.067	0.259	0.599	0.127	0.138	0.605	0.742
Q8K4G6	ADP-ribose glycohydrolase MACROD1 (Fragment) OS=Rattus norvegicus OX=10116 GN=Macrod1 PE=2 SV=2 - [MACD1_RAT]	28.6	0.013	0.013	0.013	0.013	0.013	0.792	0.819	1.164	0.114	0.678	0.707	1.006	0.935	1.371	0.424	0.707	1.089	1.371	0.567	1.085	0.009	0.0782	0.009	0.047	0.009	0.052	0.470	0.736	0.470	0.267	0.470	0.723
Q91ZV2	Discoidin, CUB and LCCL domain-containing protein 2 OS=Rattus norvegicus GN=Dcbld2 PE=2 SV=1 - [DCBD2_RAT]	83.9	0.013	0.013	0.013	0.013	0.013	0.013	0.013	0.013	0.013	0.013	1.106	1.171	1.023	1.581	0.692	0.013	0.013	0.013	0.013	0.013	#DIV/0!	#DIV/0!	0.009	0.026	#DIV/0!	#DIV/0!	0.009	0.041	#DIV/0!	#DIV/0!	0.009	0.037
Q920D2	Dihydrofolate reductase OS=Rattus norvegicus GN=Dhfr PE=2 SV=3 - [DYR_RAT]	21.6	0.013	0.013	0.013	0.013	0.013	0.013	0.013	0.013	0.013	0.013	0.277	0.486	0.670	1.172	0.013	0.429	0.375	0.660	0.140	0.750	#DIV/0!	#DIV/0!	0.025	0.064	0.009	0.584	0.025	0.093	0.009	0.355	0.917	0.862
Q9ES21	Phosphatidylinositide phosphatase SAC1 OS=Rattus norvegicus OX=10116 GN=Sacm1l PE=1 SV=1 - [SAC1_RAT]	67.0	0.013	0.013	0.013	0.013	0.013	0.013	0.013	0.013	0.013	0.013	0.930	0.979	0.919	1.323	0.509	0.013	0.013	0.013	0.013	0.013	#DIV/0!	#DIV/0!	0.009	0.038	#DIV/0!	#DIV/0!	0.009	0.056	#DIV/0!	#DIV/0!	0.009	0.054
Q9ES40	PRA1 family protein 3 OS=Rattus norvegicus OX=10116 GN=Arl6ip5 PE=1 SV=1 - [PRAF3_RAT]	21.5	0.013	0.013	0.013	0.013	0.013	0.013	0.013	0.013	0.013	0.013	0.965	1.250	0.753	1.517	0.312	0.013	0.013	0.013	0.013	0.013	#DIV/0!	#DIV/0!	0.009	0.035	#DIV/0!	#DIV/0!	0.009	0.052	#DIV/0!	#DIV/0!	0.009	0.048
Q9ES54	Nuclear protein localization protein 4 homolog OS=Rattus norvegicus GN=Nploc4 PE=1 SV=3 - [NPL4_RAT]	68.0	0.013	0.013	0.013	0.013	0.013	0.013	0.013	0.013	0.013	0.013	0.580	0.929	0.992	1.648	0.151	0.013	0.013	0.013	0.013	0.013	#DIV/0!	#DIV/0!	0.009	0.054	#DIV/0!	#DIV/0!	0.009	0.074	#DIV/0!	#DIV/0!	0.009	0.072
Q9ESN0	Protein Niban OS=Rattus norvegicus GN=Fam129a PE=1 SV=2 - [NIBAN_RAT]	103.4	0.396	0.400	1.124	0.212	0.382	0.787	1.294	1.334	0.192	1.287	1.142	1.183	0.969	1.548	0.444	0.576	0.493	0.939	0.417	1.159	0.184	0.300	0.036	0.090	0.085	0.352	0.917	0.716	0.259	0.190	0.184	0.574
Q9JIY6	Probable N-acetyltransferase CML6 OS=Rattus norvegicus OX=10116 GN=Cml6 PE=2 SV=1 - [CMLO6_RAT]	24.8	0.013	0.013	0.013	0.013	0.013	0.013	0.013	0.013	0.013	0.013	0.815	0.955	0.819	1.691	0.528	0.013	0.013	0.013	0.013	0.013	#DIV/0!	#DIV/0!	0.009	0.035	#DIV/0!	#DIV/0!	0.009	0.051	#DIV/0!	#DIV/0!	0.009	0.047
Q9JJW3	Up-regulated during skeletal muscle growth protein 5 OS=Rattus norvegicus OX=10116 GN=Atp5md PE=1 SV=1 - [USMG5_RAT]	6.4	0.013	0.013	0.013	0.013	0.013	0.535	0.731	0.857	0.197	0.624	0.773	1.245	0.768	1.783	0.443	0.013	0.013	0.013	0.013	0.013	0.009	0.226	0.009	0.032	#DIV/0!	#DIV/0!	0.184	0.502	0.009	0.125	0.009	0.045
Q9JK72	Copper chaperone for superoxide dismutase OS=Rattus norvegicus GN=Ccs PE=1 SV=1 - [CCS_RAT]	28.9	1.350	1.024	2.106	1.079	0.883	0.837	0.628	1.133	0.449	0.614	0.013	0.013	0.013	0.013	0.013	0.733	0.893	1.238	0.923	0.999	0.056	0.114	0.009	0.024	0.184	0.517	0.009	0.164	0.127	0.126	0.009	0.048
Q9JKU3	Intraflagellar transport protein 172 homolog OS=Rattus norvegicus GN=Ift172 PE=1 SV=1 - [IF172_RAT]	197.5	0.013	0.013	0.013	0.013	0.013	0.013	0.013	0.013	0.013	0.013	1.000	1.118	0.928	1.172	0.797	0.013	0.013	0.013	0.013	0.013	#DIV/0!	#DIV/0!	0.009	0.032	#DIV/0!	#DIV/0!	0.009	0.048	#DIV/0!	#DIV/0!	0.009	0.045
Q9JLD2	Neuroserpin OS=Rattus norvegicus GN=Serpini1 PE=1 SV=1 - [NEUS_RAT]	46.2	0.013	0.013	0.013	0.013	0.013	1.083	1.105	1.233	0.127	0.975	0.805	1.102	0.788	1.121	0.382	0.582	1.119	1.082	0.822	1.069	0.009	0.0324	0.009	0.061	0.009	0.053	0.605	0.711	0.605	0.299	0.756	0.747
Q9JLH7	CDK5 regulatory subunit-associated protein 3 OS=Rattus norvegicus OX=10116 GN=Cdk5rap3 PE=1 SV=1 - [CK5P3_RAT]	57.0	0.013	0.013	0.013	0.013	0.013	0.013	0.013	0.013	0.013	0.013	1.001	0.937	1.269	1.518	0.389	0.013	0.013	0.013	0.013	0.013	#DIV/0!	#DIV/0!	0.009	0.030	#DIV/0!	#DIV/0!	0.009	0.045	#DIV/0!	#DIV/0!	0.009	0.042
Q9JLT0	Myosin-10 OS=Rattus norvegicus GN=Myh10 PE=1 SV=1 - [MYH10_RAT]	228.8	0.013	0.013	0.013	0.013	0.013	0.013	0.013	0.013	0.013	0.013	2.177	1.271	0.603	0.590	1.074	0.013	0.013	0.013	0.013	0.013	#DIV/0!	#DIV/0!	0.009	0.026	#DIV/0!	#DIV/0!	0.009	0.040	#DIV/0!	#DIV/0!	0.009	0.036
Q9QUL6	Vesicle-fusing ATPase OS=Rattus norvegicus GN=Nsf PE=1 SV=1 - [NSF_RAT]	82.6	0.013	0.013	0.013	0.013	0.013	0.013	0.013	0.013	0.013	0.013	1.009	1.079	0.949	1.473	0.742	0.013	0.013	0.013	0.013	0.013	#DIV/0!	#DIV/0!	0.009	0.028	#DIV/0!	#DIV/0!	0.009	0.043	#DIV/0!	#DIV/0!	0.009	0.040
Q9QYL8	Acyl-protein thioesterase 2 OS=Rattus norvegicus GN=Lypla2 PE=1 SV=1 - [LYPA2_RAT]	24.8	0.982	0.763	1.651	0.761	0.789	0.815	0.845	1.091	0.346	0.715	0.013	0.013	0.013	0.013	0.013	0.579	1.073	0.993	0.733	0.808	0.605	0.487	0.009	0.032	0.756	0.860	0.009	0.136	0.917	0.385	0.009	0.079
Q9WUW3	Complement factor I OS=Rattus norvegicus GN=Cfi PE=2 SV=1 - [CFAI_RAT]	67.3	1.195	0.744	1.862	0.753	0.656	0.876	1.002	1.065	0.141	1.097	0.013	0.013	0.013	0.013	0.013	0.569	1.006	1.249	0.801	0.996	0.917	0.551	0.009	0.029	0.917	0.850	0.009	0.086	0.917	0.372	0.009	0.056
Q9Z252	Protein lin-7 homolog B OS=Rattus norvegicus GN=Lin7b PE=1 SV=1 - [LIN7B_RAT]	22.9	0.450	0.736	2.065	0.732	0.502	0.013	0.013	0.013	0.013	0.013	0.013	0.013	0.013	0.013	0.013	0.013	0.013	0.013	0.013	0.013	0.009	0.0330	0.009	0.044	0.009	0.056	#DIV/0!	#DIV/0!	#DIV/0!	#DIV/0!	#DIV/0!	#DIV/0!
Q9Z270	Vesicle-associated membrane protein-associated protein A OS=Rattus norvegicus OX=10116 GN=Vapa PE=1 SV=3 - [VAPA_RAT]	27.8	0.013	0.013	0.013	0.013	0.013	0.693	1.046	1.061	0.162	0.777	0.531	0.749	0.907	1.480	0.223	0.582	0.550	0.737	0.534	0.843	0.009	0.0635	0.009	0.096	0.009	0.154	0.917	0.818	0.354	0.246	0.756	0.852

Table S4. Extracted protein profile of d-II (Effect of FGR, only Good nutrition, Female)

Accession	Description	MW [kDa]	Expression level																				Steel-Dwass & FDR											
			FGR-Good-Female					FGR-Poor_Female					Sham-Good-Female					Sham-Poor-Female					FGR-GN vs FGR-PN		FGR-GN vs Sham-GN		FGR-GN vs Sham-PN		FGR-PN vs Sham-GN		FGR-PN vs Sham-PN		Sham-GN vs Sham-PN	
			#01	#02	#03	#04	#05	#06	#07	#08	#09	#10	#11	#12	#13	#14	#15	#16	#17	#18	#19	#20												
Filter	Filter	Filter	Filter	Filter	Filter	Filter	Filter	Filter	Filter	Filter	Filter	Filter	Filter	Filter	Filter	Filter	Filter	Filter	Filter	Filter	Filter	Filter	p-value	q-value	p	q-value	p	q-value	p	q-value	p	q-value	p	q-value
A0JPJ7	Obg-like ATPase 1 OS=Rattus norvegicus GN=Ola1 PE=2 SV=1 - [OLA1_RAT]	44.5	0.904	1.000	0.682	1.277	0.984	1.525	0.625	0.858	0.799	0.583	1.479	1.405	1.823	1.397	0.999	1.450	0.664	1.962	1.549	0.538	0.259	0.857	0.036	0.057	0.605	0.538	0.085	0.089	0.470	0.352	0.917	1.163
A2RUW1	Toll-interacting protein OS=Rattus norvegicus GN=Tollip PE=2 SV=1 - [TOLIP_RAT]	30.3	0.013	0.013	0.013	0.013	0.013	0.903	0.383	0.716	1.183	1.098	1.282	1.035	2.236	3.001	1.044	2.502	0.537	1.536	1.445	1.283	0.009	0.116	0.009	0.040	0.009	0.044	0.085	0.086	0.085	0.095	0.917	1.016
A2VCW9	Alpha-aminoadipic semialdehyde synthase, mitochondrial OS=Rattus norvegicus OX=10116 GN=Aass PE=2 SV=1 - [AASS_RAT]	102.8	0.435	0.823	0.363	0.780	0.704	1.673	0.424	0.829	0.907	1.130	0.881	0.935	1.875	3.520	1.239	2.962	0.852	1.399	1.201	0.850	0.085	0.432	0.014	0.035	0.014	0.040	0.184	0.153	0.354	0.273	0.470	1.431
B0BN18	Prefoldin subunit 2 OS=Rattus norvegicus GN=Pfdn2 PE=2 SV=1 - [PFD2_RAT]	16.6	0.680	1.146	0.382	1.233	1.071	0.801	0.341	0.408	1.111	0.995	1.359	1.155	2.496	2.896	1.294	2.855	0.523	1.642	1.463	1.409	0.354	0.999	0.022	0.039	0.085	0.118	0.014	0.026	0.056	0.076	0.917	0.972
B0BNE5	S-formylglutathione hydrolase OS=Rattus norvegicus GN=Esd PE=1 SV=1 - [ESTD_RAT]	31.3	0.599	1.046	0.516	1.078	1.019	1.075	0.442	0.675	1.044	1.009	1.093	1.098	1.939	2.044	1.153	2.030	0.795	1.296	1.349	1.207	0.756	0.987	0.014	0.034	0.056	0.102	0.014	0.030	0.056	0.083	0.917	1.108
B0LT89	Serine/threonine-protein kinase 24 OS=Rattus norvegicus GN=Stk24 PE=2 SV=1 - [STK24_RAT]	48.0	0.431	1.200	0.344	1.153	1.017	1.237	0.287	0.557	1.455	0.906	1.717	1.468	2.666	2.589	1.117	2.275	0.493	2.218	2.174	1.098	0.756	0.972	0.036	0.054	0.127	0.158	0.036	0.050	0.184	0.159	0.470	1.396
B2GUV7	Eukaryotic translation initiation factor 5B OS=Rattus norvegicus GN=Eif5b PE=1 SV=1 - [IF2P_RAT]	137.6	1.059	0.858	0.779	1.087	0.986	1.455	0.843	0.897	0.887	0.880	1.112	1.226	2.108	1.759	1.134	1.404	0.981	1.775	1.176	0.739	0.917	0.924	0.014	0.032	0.354	0.352	0.056	0.066	0.354	0.276	0.470	1.562
B3GNI6	Septin-11 OS=Rattus norvegicus GN=Sept11 PE=1 SV=1 - [SEP11_RAT]	49.7	1.046	0.845	0.711	1.077	0.994	1.478	0.742	0.866	0.788	0.888	1.486	1.573	3.185	3.633	1.044	2.197	0.948	1.752	1.360	1.038	0.756	0.918	0.036	0.054	0.127	0.160	0.022	0.036	0.056	0.074	0.259	1.264
B4F779	DCC-interacting protein 13-beta OS=Rattus norvegicus OX=10116 GN=Appl2 PE=2 SV=1 - [DP13B_RAT]	74.1	0.013	0.013	0.013	0.013	0.013	0.013	0.013	0.013	0.013	0.013	1.229	2.055	4.003	0.577	3.482	0.013	0.013	0.013	0.013	0.013	#DIV/0!	#DIV/0!	0.009	0.029	#DIV/0!	#DIV/0!	0.009	0.023	#DIV/0!	#DIV/0!	0.009	0.064
B5DF89	Cullin-3 OS=Rattus norvegicus GN=Cul3 PE=1 SV=2 - [CUL3_RAT]	88.9	0.717	1.052	0.578	1.019	0.933	1.565	0.597	0.670	0.887	0.715	1.503	1.929	2.163	2.791	0.953	1.940	0.755	1.824	1.589	0.861	0.605	0.984	0.036	0.054	0.184	0.212	0.036	0.050	0.085	0.096	0.259	1.273
B5DFC8	Eukaryotic translation initiation factor 3 subunit C OS=Rattus norvegicus GN=Eif3c PE=1 SV=1 - [EIF3C_RAT]	105.4	1.026	0.969	0.522	1.127	0.906	1.680	0.362	0.569	0.593	0.694	1.701	1.525	1.987	1.198	1.015	1.275	0.792	2.223	1.417	0.850	0.354	0.972	0.036	0.057	0.354	0.349	0.056	0.070	0.085	0.103	0.470	1.490
D3ZAW2	Phosphatidylserine decarboxylase proenzyme, mitochondrial OS=Rattus norvegicus OX=10116 GN=Pisd PE=1 SV=3 - [PISD_RAT]	45.8	0.013	0.013	0.013	0.013	0.013	0.013	0.013	0.013	0.013	0.013	0.810	0.699	1.439	3.167	0.824	0.013	0.013	0.013	0.013	0.013	#DIV/0!	#DIV/0!	0.009	0.059	#DIV/0!	#DIV/0!	0.009	0.050	#DIV/0!	#DIV/0!	0.009	0.108
D3ZDK7	Glycerol-3-phosphate phosphatase OS=Rattus norvegicus OX=10116 GN=Pgp PE=1 SV=1 - [PGP_RAT]	34.6	0.642	0.806	0.651	0.919	0.926	1.171	0.534	0.744	0.855	0.930	1.016	1.205	1.771	2.494	1.055	2.628	0.710	1.235	1.123	1.114	0.605	1.027	0.014	0.035	0.056	0.106	0.036	0.052	0.184	0.163	0.917	1.086
D3ZHA0	Filamin-C OS=Rattus norvegicus GN=Flnc PE=1 SV=1 - [FLNC_RAT]	290.8	0.744	0.837	0.461	0.754	0.609	1.136	0.544	0.496	0.823	0.823	0.909	1.099	1.748	2.661	0.920	2.755	0.637	1.648	1.203	0.772	0.605	1.104	0.014	0.038	0.085	0.129	0.056	0.071	0.184	0.164	0.756	1.275
D3ZKF5	Serine protease HTR4 OS=Rattus norvegicus OX=10116 GN=Htra4 PE=3 SV=1 - [HTRA4_RAT]	52.2	0.013	0.013	0.013	0.013	0.013	0.013	0.013	0.013	0.013	0.013	0.953	1.017	1.750	2.570	1.093	0.013	0.013	0.013	0.013	0.013	#DIV/0!	#DIV/0!	0.009	0.051	#DIV/0!	#DIV/0!	0.009	0.044	#DIV/0!	#DIV/0!	0.009	0.094
D3ZMK9	Inactive tyrosine-protein kinase PRAG1 OS=Rattus norvegicus OX=10116 GN=Prag1 PE=1 SV=1 - [PRAG1_RAT]	147.6	1.674	0.257	1.449	1.305	0.910	0.973	0.682	1.428	0.253	0.504	0.064	0.135	0.260	0.019	0.451	0.013	2.158	0.263	0.169	0.048	0.259	0.843	0.036	0.062	0.184	0.226	0.036	0.057	0.184	0.176	0.917	1.325
D3ZW55	Inosine triphosphate pyrophosphatase OS=Rattus norvegicus GN=Itpa PE=3 SV=1 - [ITPA_RAT]	21.9	0.772	0.886	0.585	1.009	0.881	1.145	0.525	0.696	0.978	1.060	1.129	1.158	2.036	1.734	1.276	1.709	0.864	1.368	1.347	1.179	0.605	0.998	0.014	0.035	0.056	0.111	0.022	0.039	0.056	0.084	0.756	1.320
D4A2Z8	ATP-dependent DNA/RNA helicase DHX36 OS=Rattus norvegicus OX=10116 GN=Dhx36 PE=1 SV=1 - [DHX36_RAT]	113.8	0.013	0.013	0.013	0.013	0.013	0.013	0.013	0.013	0.013	0.013	1.565	1.492	3.145	1.829	1.429	0.013	0.013	0.013	0.013	0.013	#DIV/0!	#DIV/0!	0.009	0.034	#DIV/0!	#DIV/0!	0.009	0.026	#DIV/0!	#DIV/0!	0.009	0.071
D4A4T9	Cysteine and histidine-rich domain-containing protein 1 OS=Rattus norvegicus GN=Chordc1 PE=3 SV=1 - [CHRD1_RAT]	37.3	0.013	0.013	0.013	0.013	0.013	1.291	0.548	0.687	0.970	0.994	1.065	0.995	2.010	2.225	1.015	0.013	0.013	0.013	0.013	0.013	0.009	0.091	0.009	0.053	#DIV/0!	#DIV/0!	0.056	0.068	0.009	0.142	0.009	0.095
F1LMZ8	26S proteasome non-ATPase regulatory subunit 11 OS=Rattus norvegicus GN=Psmd11 PE=3 SV=2 - [PSD11_RAT]	47.4	0.668	1.170	0.311	1.105	1.000	1.339	0.481	0.526	0.935	0.769	1.274	1.296	2.067	2.186	1.041	2.174	0.709	2.045	1.726	0.802	0.756	1.011	0.036	0.057	0.184	0.208	0.056	0.067	0.127	0.124	0.605	1.383
F1M775	Protein diaphanous homolog 1 OS=Rattus norvegicus OX=10116 GN=Diaph1 PE=1 SV=3 - [DIAP1_RAT]	140.3	0.714	1.061	0.426	1.072	1.022	1.292	0.497	0.701	1.104	1.076	1.444	1.582	2.576	2.384	1.300	1.710	0.758	1.972	1.947	1.084	0.354	0.916	0.014	0.028	0.056	0.092	0.014	0.025	0.127	0.123	0.470	1.416
G3V6S8	Serine/arginine-rich splicing factor 6 OS=Rattus norvegicus OX=10116 GN=Srsf6 PE=1 SV=1 - [SRSF6_RAT]	39.0	0.013	0.013	0.013	0.013	0.013	0.013	0.013	0.013	0.013	0.013	1.013	0.938	1.638	1.019	1.098	0.013	0.013	0.013	0.013	0.013	#DIV/0!	#DIV/0!	0.009	0.081	#DIV/0!	#DIV/0!	0.009	0.072	#DIV/0!	#DIV/0!	0.009	0.170
O08557	N(G),N(G)-dimethylarginine dimethylaminohydrolase 1 OS=Rattus norvegicus GN=Ddah1 PE=1 SV=3 - [DDAH1_RAT]	31.4	0.549	0.896	0.455	1.014	0.904	1.009	0.415	0.608	1.027	1.094	1.156	1.254	2.309	2.139	1.117	1.737	0.781	1.379	1.245	1.339	0.470	1.073	0.014	0.033	0.056	0.117	0.014	0.028	0.056	0.088	0.756	1.264
O08618	Phosphoribosyl pyrophosphate synthase-associated protein 2 OS=Rattus norvegicus GN=Prpsap2 PE=2 SV=1 - [KPRB_RAT]	40.8	0.013	0.013	0.013	0.013	0.013	0.013	0.013	0.013	0.013	0.013	0.744	0.768	1.352	1.795	0.900	0.013	0.013	0.013	0.013	0.013	#DIV/0!	#DIV/0!	0.009	0.088	#DIV/0!	#DIV/0!	0.009	0.078	#DIV/0!	#DIV/0!	0.009	0.196
O08651	D-3-phosphoglycerate dehydrogenase OS=Rattus norvegicus GN=Phgdh PE=1 SV=3 - [SERA_RAT]	56.5	0.767	1.032	0.655	1.126	0.931	1.542	0.617	0.750	0.899	0.766	1.151	1.291	1.795	1.427	0.981	1.112	0.883	1.638	1.376	0.571	0.470	0.953	0.036	0.059	0.470	0.447	0.085	0.091	0.470	0.354	0.354	1.599
O35112	CD166 antigen OS=Rattus norvegicus GN=Alcam PE=1 SV=1 - [CD166_RAT]	65.0	0.013	0.013	0.013	0.013	0.013	1.512	0.735	0.648	0.930	0.761	1.075	0.926	1.999	1.225	0.752	0.013	0.013	0.013	0.013	0.013	0.009	0.079	0.009	0.076	#DIV/0!	#DIV/0!	0.259	0.211	0.009	0.126	0.009	0.154
O35244	Peroxiredoxin-6 OS=Rattus norvegicus GN=Prdx6 PE=1 SV=3 - [PRDX6_RAT]	24.8	0.613	0.855	0.570	0.984	0.960	1.001	0.462	0.623	0.801	0.868	0.973	0.963	1.882	2.774	1.057	2.548	0.643	1.261	1.110	1.157	0.917	1.027	0.036	0.058	0.056	0.107	0.036	0.054	0.056	0.091	0.917	1.084
O35263	Platelet-activating factor acetylhydrolase IB subunit gamma OS=Rattus norvegicus GN=Pafah1b3 PE=2 SV=1 - [PA1B3_RAT]	25.8	0.013	0.013	0.013	0.013	0.013	0.013	0.013	0.013	0.013	0.013	1.776	1.636	3.759	3.312	1.896	1.172	0.999	0.989	1.007	0.572	#DIV/0!	#DIV/0!	0.009	0.028	0.009	0.139	0.009	0.022	0.009	0.113	0.014	0.091
O35264	Platelet-activating factor acetylhydrolase IB subunit beta OS=Rattus norvegicus GN=Pafah1b2 PE=1 SV=1 - [PA1B2_RAT]	25.6	0.450	1.364	0.314	1.351	1.165	0.904	0.235	0.460	1.232	1.369	1.502	1.275	2.724	1.870	1.472	2.004	0.526	1.684	1.755	1.783	0.917	0.948	0.036	0.055	0.056	0.090	0.022	0.037	0.056	0.075	0.917	0.990
O35331	Pyridoxal kinase OS=Rattus norvegicus GN=Pdxk PE=1 SV=1 - [PDXK_RAT]	34.9	0.607	0.844	0.437	0.968	0.882	0.861	0.400	0.546	1.033	0.948	0.907	0.989	1.780	3.399	1.067	2.959	0.621	1.334	1.290	1.191	0.917	1.039	0.022	0.041	0.056	0.099	0.056	0.067	0.056	0.080	0.917	1.031
O35567	Bifunctional purine biosynthesis protein PURH OS=Rattus norvegicus GN=Atic PE=1 SV=2 - [PUR9_RAT]	64.2	0.545	1.206	0.424	1.100	1.000	1.104	0.396	0.527	1.148	1.056	1.284	1.442	2.513	1.599	1.238	1.606	0.761	1.643	1.503	1.000	0.917	0.973	0.014	0.031	0.184	0.216	0.014	0.027	0.184	0.165	0.756	1.261
O35820	2'-deoxynucleoside 5'-phosphate N-hydrolase 1 OS=Rattus norvegicus OX=10116 GN=Dnph1 PE=1 SV=1 - [DNPH1_RAT]	17.8	0.660	0.965	0.484	1.111	0.986	1.064	0.473	0.601	1.028	0.927	1.008	1.123	2.048	1.790	1.207	1.750	0.806	1.617	1.499	1.295	0.756	1.012	0.022	0.043	0.056	0.099	0.036	0.054	0.056	0.081	0.917	1.100
O54975	Xaa-Pro aminopeptidase 1 OS=Rattus norvegicus GN=Xpnpep1 PE=1 SV=1 - [XPP1_RAT]	69.6	0.588	0.827	0.509	0.882	0.850	1.166	0.518	0.687	0.821	0.866	0.909	0.969	1.726	2.753	0.980	2.351	0.805	1.226	1.045	1.038	0.756	1.074	0.014	0.037	0.056	0.120	0.056	0.069	0.127	0.129	0.917	1.122
O55004	Ribonuclease 4 OS=Rattus norvegicus GN=Rnase4 PE=1 SV=1 - [RNAS4_RAT]	16.9	0.013	0.013	0.013	0.013	0.013	0.013	0.013	0.013	0.013	0.013	1.402	1.242	2.737	2.984	1.494	0.013	0.013	0.013	0.013	0.013	#DIV/0!	#DIV/0!	0.009	0.032	#DIV/0!	#DIV/0!	0.009	0.025	#DIV/0!	#DIV/0!	0.009	0.070
O55171	Acyl-coenzyme A thioesterase 2, mitochondrial OS=Rattus norvegicus GN=Acot2 PE=1 SV=1 - [ACOT2_RAT]	49.7	0.363	0.745	0.241	0.686	0.790	0.751	0.332	0.376	0.813	0.877	0.869	0.915	1.684	5.341	0.890	5.491	0.592	1.032	1.201	0.966	0.354	1.091	0.014	0.031	0.056	0.090	0.022	0.037	0.056	0.074	0.756	1.111
O70352	CD82 antigen OS=Rattus norvegicus OX=10116 GN=Cd82 PE=1 SV=1 - [CD82_RAT]	29.5	0.013	0.013	0.013	0.013	0.013	0.013	0.013	0.013	0.013	0.013	1.083	0.712	1.212	3.428	0.666	0.013	0.013	0.013	0.013	0.013	#DIV/0!	#DIV/0!	0.009	0.057	#DIV/0!	#DIV/0!	0.009	0.048	#DIV/0!	#DIV/0!	0.009	0.101
O70437	Mothers against decapentaplegic homolog 4 OS=Rattus norvegicus GN=Smad4 PE=1 SV=1 - [SMAD4_RAT]	60.4	0.013	0.013	0.013	0.013	0.013	0.013	0.013	0.013	0.013	0.013	1.348	1.258	1.979	2.168	1.271	0.013	0.013	0.013	0.013	0.013	#DIV/0!	#DIV/0!	0.009	0.045	#DIV/0!	#DIV/0!	0.009	0.037	#DIV/0!	#DIV/0!	0.009	0.085
O70490	Acyl-coenzyme A synthetase ACSM2, mitochondrial OS=Rattus norvegicus OX=10116 GN=Acsm2 PE=2 SV=2 - [ACSM2_RAT]	64.1	0.144	0.579	0.135	0.497	0.341	0.217	0.093	0.161	0.730	0.988	0.651	0.678	1.071	7.243	0.641	5.354	0.555	0.881	0.853	0.739	0.756	1.136	0.014	0.032	0.022	0.060	0.184	0.154	0.127	0.129	0.917	0.940
O70511	Ankyrin-3 OS=Rattus norvegicus GN=Ank3 PE=1 SV=3 - [ANK3_RAT]	284.3	0.013	0.013	0.013	0.013	0.013	0.013	0.013	0.013	0.013	0.013	1.297	1.190	1.500	7.790	1.036	0.013	0.013	0.013	0.013	0.013	#DIV/0!	#DIV/0!	0.009	0.027	#DIV/0!	#DIV/0!	0.009	0.022	#DIV/0!	#DIV/0!	0.009	0.062
O88267	Acyl-coenzyme A thioesterase 1 OS=Rattus norvegicus GN=Acot1 PE=1 SV=1 - [ACOT1_RAT]	46.0	0.013	0.013	0.013	0.013	0.013	0.661	0.638	0.713	0.706	0.858	0.968	0.972	1.762	5.917	0.842	5.116	0.483	1.392	1.275	1.243	0.009	0.449	0.009	0.031	0.009	0.031	0.022	0.036	0.127	0.122	0.917	0.926
O88350	Putative hydrolase RBBP9 OS=Rattus norvegicus GN=Rbbp9 PE=1 SV=2 - [RBBP9_RAT]	21.0	0.578	1.129	0.262	1.257	1.044	0.865	0.343	0.400	1.185	1.099	1.401	1.322	2.585	2.214	1.382	2.008	0.810	1.597	1.732	1.476	0.756	1.026	0.014	0.029	0.056	0.091	0.014	0.026	0.056	0.077	0.917	0.994
O88600	Heat shock 70 kDa protein 4 OS=Rattus norvegicus GN=Hspa4 PE=1 SV=1 - [HSP74_RAT]	94.0	0.688	0.842	0.598	0.953	0.879	1.133	0.499	0.719	0.917	0.918	0.886	1.027	1.840	1.589	1.102	1.632	0.858	1.206	1.099	1.025	0.605	1.030	0.022	0.044	0.036	0.091	0.127	0.122	0.127	0.132	0.605	1.591
O88761	26S proteasome non-ATPase regulatory subunit 1 OS=Rattus norvegicus GN=Psmd1 PE=2 SV=1 - [PSMD1_RAT]	105.7	0.979	0.969	0.657	1.117	0.975	1.313	0.678	0.721	0.988	0.732	1.390	1.658	2.360	2.437	1.233	2.316	0.521	2.421	1.820	0.979	0.917	0.935	0.014	0.028	0.259	0.270	0.022	0.037	0.259	0.210	0.605	1.313
O88767	Protein DJ-1 OS=Rattus norvegicus GN=Park7 PE=1 SV=1 - [PARK7_RAT]	20.0	0.682	0.982	0.566	1.084	1.018	0.936	0.465	0.663	1.107	1.116	1.221	1.174	2.396	1.900	1.203	1.686	0.864	1.410	1.530	1.395	0.917	0.965	0.014	0.031	0.056	0.098	0.014	0.028	0.056	0.080	0.917	1.067
O88807	Protein-arginine deiminase type-4 OS=Rattus norvegicus OX=10116 GN=Padi4 PE=2 SV=1 - [PADI4_RAT]	74.4	0.776	0.757	1.009	1.696	1.514	1.818	0.266	1.581	0.374	0.862	0.112	0.239	0.510	0.182	0.809	0.139	0.794	0.380	0.519	0.132	0.756	0.899	0.036	0.062	0.036	0.092	0.085	0.098	0.184	0.176	0.917	1.324
O88989	Malate dehydrogenase, cytoplasmic OS=Rattus norvegicus GN=Mdh1 PE=1 SV=3 - [MDHC_RAT]	36.5	0.633	0.864	0.516	0.900	0.860	0.987	0.475	0.680	0.852	0.925	0.881	0.921	1.778	3.270	0.920	2.797	0.755	1.103	1.034	1.149	0.756	1.076	0.022	0.042	0.056	0.110	0.184	0.156	0.056	0.086	0.917	1.074
O89049	Thioredoxin reductase 1, cytoplasmic OS=Rattus norvegicus GN=Txnrd1 PE=1 SV=5 - [TRXR1_RAT]	54.6	0.516	0.818	0.498	0.804	0.713	1.014	0.434	0.610	0.770	0.752	0.876	0.885	1.681	3.161	0.965	2.712	0.608	1.096	1.031	0.960	0.917	1.061	0.014	0.038	0.056	0.126	0.056	0.071	0.127	0.133	0.917	1.110
P00564	Creatine kinase M-type OS=Rattus norvegicus GN=Ckm PE=1 SV=2 - [KCRM_RAT]	43.0	0.013	0.013	0.013	0.013	0.013	0.712	0.405	0.538	0.757	2.981	0.887	1.119	1.966	0.835	1.513	1.571	0.653	1.477	1.225	0.843	0.009	0.056	0.009	0.068	0.009	0.073	0.127	0.117	0.259	0.212	0.756	1.461
P00762	Anionic trypsin-1 OS=Rattus norvegicus GN=Prss1 PE=1 SV=1 - [TRY1_RAT]	25.9	0.712	1.226	0.443	1.089	0.998	0.854	0.422	0.538	1.560	1.303	1.644	1.482	2.984	1.076	1.432	0.975	0.900	1.467	1.955	1.454	0.917	0.934	0.036	0.055	0.184	0.212	0.085	0.086	0.184	0.161	0.354	1.434
P00787	Cathepsin B OS=Rattus norvegicus GN=Ctsb PE=1 SV=2 - [CATB_RAT]	37.4	0.661	0.595	0.443	0.373	0.260	0.696	0.439	0.577	0.653	0.647	0.675	0.678	1.200	6.328	0.616	7.128	0.593	0.807	0.958	0.861	0.259	0.959	0.022	0.042	0.036	0.083	0.127	0.115	0.056	0.073	0.756	1.105
P01015	Angiotensinogen OS=Rattus norvegicus GN=Agt PE=1 SV=1 - [ANGT_RAT]	51.9	0.013	0.013	0.013	0.013	0.013	1.098	0.380	0.990	0.738	0.934	0.648	0.622	1.448	1.688	1.075	1.612	0.939	0.905	0.783	0.992	0.009	0.159	0.009	0.094	0.009	0.090	0.470	0.359	0.470	0.359	0.917	1.298
P01026	Complement C3 OS=Rattus norvegicus GN=C3 PE=1 SV=3 - [CO3_RAT]	186.3	0.536	0.630	0.471	0.694	0.724	0.682	0.394	0.728	0.768	0.781	0.819	0.833	1.734	2.051	1.022	2.902	0.676	1.357	1.023	1.017	0.259	0.950	0.014	0.040	0.036	0.088	0.014	0.032	0.085	0.104	0.917	1.151
P01048	T-kininogen 1 OS=Rattus norvegicus GN=Map1 PE=1 SV=2 - [KNT1_RAT]	47.7	0.871	0.888	0.759	1.164	1.037	0.882	0.507	0.920	0.996	0.978	1.271	0.910	2.040	2.185	1.263	3.226	0.847	1.409	1.050	0.797	0.756	0.940	0.036	0.056	0.470	0.431	0.056	0.067	0.354	0.273	0.470	1.446
P01835	Ig kappa chain C region, B allele OS=Rattus norvegicus PE=1 SV=1 - [KACB_RAT]	11.6	0.424	0.170	0.416	0.765	0.530	1.212	1.002	1.062	1.051	0.996	1.118	0.843	2.165	0.545	1.038	3.251	0.458	2.181	1.206	1.328	0.014	0.077	0.022	0.045	0.036	0.084	0.756	0.548	0.184	0.159	0.259	1.295
P02091	Hemoglobin subunit beta-1 OS=Rattus norvegicus GN=Hbb PE=1 SV=3 - [HBB1_RAT]	16.0	0.537	0.484	0.369	0.531	0.555	0.776	0.241	0.742	0.468	0.494	0.762	0.770	1.955	3.649	0.840	4.691	0.377	1.371	0.917	1.138	0.917	1.080	0.014	0.039	0.085	0.124	0.036	0.056	0.085	0.097	0.756	1.165
P02680	Fibrinogen gamma chain OS=Rattus norvegicus GN=Fgg PE=1 SV=3 - [FIBG_RAT]	50.6	0.474	0.721	0.564	0.738	0.765	0.465	0.286	0.459	1.285	0.798	0.775	0.833	1.397	2.346	0.915	3.567	0.554	1.138	0.812	1.141	0.605	1.126	0.014	0.040	0.085	0.128	0.085	0.097	0.127	0.129	0.917	1.147
P02764	Alpha-1-acid glycoprotein OS=Rattus norvegicus GN=Orm1 PE=2 SV=1 - [A1AG_RAT]	23.6	0.013	0.013	0.013	0.013	0.013	0.013	0.013	0.013	0.013	0.013	0.942	1.120	2.396	2.691	1.527	0.013	0.013	0.013	0.013	0.013	#DIV/0!	#DIV/0!	0.009	0.040	#DIV/0!	#DIV/0!	0.009	0.031	#DIV/0!	#DIV/0!	0.009	0.078
P02767	Transthyretin OS=Rattus norvegicus GN=Ttr PE=1 SV=1 - [TTHY_RAT]	15.7	0.426	0.590	0.423	0.656	0.518	0.570	0.248	0.422	0.536	0.538	0.762	0.655	1.630	3.178	0.623	4.414	0.436	1.024	0.769	0.957	0.470	1.135	0.036	0.062	0.056	0.119	0.014	0.033	0.056	0.10004	0.756	1.265
P02793	Ferritin light chain 1 OS=Rattus norvegicus GN=Ftl1 PE=1 SV=3 - [FRIL1_RAT]	20.7	0.513	0.812	0.414	0.731	0.623	0.676	0.305	0.519	0.843	0.775	1.288	1.169	2.490	2.454	1.067	3.774	0.545	1.397	1.286	1.135	0.756	1.125	0.014	0.035	0.056	0.097	0.014	0.030	0.056	0.079	0.756	1.159
P04157	Receptor-type tyrosine-protein phosphatase C OS=Rattus norvegicus OX=10116 GN=Ptprc PE=1 SV=2 - [PTPRC_RAT]	143.2	1.364	0.043	1.426	0.399	0.320	0.013	0.013	0.013	0.013	0.013	0.013	0.033	0.071	0.022	0.129	0.013	0.013	0.013	0.013	0.013	0.009	0.490	0.036	0.062	0.009	0.854	0.025	0.040	#DIV/0!	#DIV/0!	0.025	0.163
P04642	L-lactate dehydrogenase A chain OS=Rattus norvegicus GN=Ldha PE=1 SV=1 - [LDHA_RAT]	36.4	0.553	0.958	0.503	0.960	0.893	1.139	0.417	0.678	0.874	0.940	1.045	1.152	2.060	2.175	1.253	2.075	0.752	1.427	1.227	1.250	0.917	1.013	0.014	0.035	0.056	0.111	0.022	0.039	0.056	0.085	0.756	1.269
P04762	Catalase OS=Rattus norvegicus GN=Cat PE=1 SV=3 - [CATA_RAT]	59.7	0.385	0.436	0.355	0.564	0.453	0.665	0.317	0.536	0.560	0.563	0.665	0.696	1.204	5.914	0.635	5.521	0.461	0.962	0.872	0.768	0.354	1.099	0.014	0.036	0.022	0.059	0.022	0.039	0.085	0.097	0.917	0.958
P04764	Alpha-enolase OS=Rattus norvegicus GN=Eno1 PE=1 SV=4 - [ENOA_RAT]	47.1	0.522	0.821	0.393	0.846	0.841	0.822	0.396	0.589	0.776	0.847	0.832	0.884	1.692	4.470	0.874	3.948	0.589	1.101	1.083	1.099	0.917	1.063	0.036	0.057	0.056	0.097	0.022	0.038	0.056	0.079	0.917	0.992
P04904	Glutathione S-transferase alpha-3 OS=Rattus norvegicus GN=Gsta3 PE=1 SV=3 - [GSTA3_RAT]	25.3	0.457	0.851	0.455	0.843	0.802	0.653	0.269	0.338	0.705	0.731	1.087	0.963	2.439	7.000	0.881	6.862	0.451	1.513	1.347	1.252	0.184	0.784	0.014	0.028	0.127	0.156	0.014	0.025	0.056	0.073	0.917	0.919
P04906	Glutathione S-transferase P OS=Rattus norvegicus GN=Gstp1 PE=1 SV=2 - [GSTP1_RAT]	23.4	0.516	1.093	0.399	1.068	0.978	0.918	0.319	0.550	1.060	1.122	1.119	1.080	2.218	1.298	1.206	1.526	0.826	1.437	1.345	1.557	0.917	1.005	0.022	0.043	0.056	0.106	0.036	0.055	0.056	0.087	0.470	1.537
P05369	Farnesyl pyrophosphate synthase OS=Rattus norvegicus GN=Fdps PE=2 SV=2 - [FPPS_RAT]	40.8	0.504	1.125	0.357	1.012	0.961	0.942	0.419	0.483	1.144	1.186	1.214	1.139	2.299	1.604	1.351	1.223	0.899	1.734	1.550	1.321	0.756	1.029	0.014	0.034	0.056	0.108	0.036	0.052	0.056	0.084	0.756	1.280
P05370	Glucose-6-phosphate 1-dehydrogenase OS=Rattus norvegicus GN=G6pdx PE=1 SV=3 - [G6PD_RAT]	59.3	0.477	0.797	0.438	0.848	0.829	0.569	0.285	0.320	1.225	1.202	1.281	1.380	2.759	2.083	1.311	2.582	0.515	1.574	1.415	1.684	0.917	1.059	0.014	0.032	0.056	0.099	0.014	0.027	0.056	0.078	0.917	0.991
P06214	Delta-aminolevulinic acid dehydratase OS=Rattus norvegicus GN=Alad PE=1 SV=1 - [HEM2_RAT]	36.0	0.516	1.053	0.415	0.706	0.851	0.832	0.395	0.537	1.110	1.000	1.014	0.955	2.025	2.900	1.103	3.131	0.478	1.755	1.787	1.725	0.756	1.090	0.036	0.058	0.085	0.118	0.085	0.088	0.085	0.092	0.756	1.152
P06399	Fibrinogen alpha chain OS=Rattus norvegicus GN=Fga PE=1 SV=3 - [FIBA_RAT]	86.6	0.815	0.753	0.647	0.773	0.846	0.886	0.407	0.752	1.091	0.763	0.826	0.832	1.569	1.691	0.958	2.243	0.646	1.069	0.828	1.122	0.917	1.029	0.036	0.061	0.184	0.224	0.127	0.125	0.259	0.219	0.917	1.249
P06685	Sodium/potassium-transporting ATPase subunit alpha-1 OS=Rattus norvegicus GN=Atp1a1 PE=1 SV=1 - [AT1A1_RAT]	113.0	0.629	0.521	0.533	0.674	0.532	0.858	0.410	0.337	0.657	0.597	0.738	0.771	1.115	5.256	0.635	4.646	0.524	1.190	0.854	0.644	0.917	1.076	0.022	0.043	0.127	0.167	0.085	0.090	0.184	0.165	0.917	1.000
P06757	Alcohol dehydrogenase 1 OS=Rattus norvegicus GN=Adh1 PE=1 SV=3 - [ADH1_RAT]	39.6	0.333	0.485	0.240	0.407	0.429	0.416	0.163	0.279	0.578	0.520	0.449	0.505	0.711	6.047	0.558	7.288	0.371	0.850	0.795	0.571	0.756	1.138	0.022	0.045	0.056	0.092	0.127	0.124	0.085	0.094	0.470	1.393
P06866	Haptoglobin OS=Rattus norvegicus GN=Hp PE=1 SV=3 - [HPT_RAT]	38.5	0.561	0.515	0.395	0.544	0.688	0.627	0.394	0.886	0.783	0.773	0.737	0.626	1.333	1.252	1.144	4.554	0.603	1.300	0.989	1.284	0.184	0.781	0.022	0.045	0.022	0.059	0.259	0.215	0.085	0.092	0.605	1.464
P07633	Propionyl-CoA carboxylase beta chain, mitochondrial OS=Rattus norvegicus OX=10116 GN=Pccb PE=2 SV=1 - [PCCB_RAT]	58.6	0.537	1.057	0.400	1.013	0.851	1.007	0.420	0.526	0.943	0.910	1.002	1.278	2.373	2.138	1.375	1.768	0.804	1.657	1.389	1.341	0.756	1.079	0.036	0.057	0.056	0.105	0.022	0.038	0.056	0.085	0.756	1.227
P07943	Aldose reductase OS=Rattus norvegicus GN=Akr1b1 PE=1 SV=3 - [ALDR_RAT]	35.8	0.764	0.972	0.619	1.027	0.901	1.053	0.533	0.760	0.880	1.005	1.034	1.055	2.025	1.084	1.125	1.083	0.876	1.216	1.247	1.183	0.917	0.971	0.014	0.039	0.056	0.124	0.022	0.040	0.056	0.101	0.756	1.476
P08460	Nidogen-1 (Fragment) OS=Rattus norvegicus GN=Nid1 PE=1 SV=2 - [NID1_RAT]	35.6	0.013	0.013	0.013	0.013	0.013	0.899	0.357	0.477	0.971	0.971	0.904	0.843	1.786	2.687	0.889	2.363	0.731	1.354	1.278	1.112	0.009	0.359	0.009	0.056	0.009	0.052	0.354	0.276	0.056	0.090	0.917	1.114
P08461	Dihydrolipoyllysine-residue acetyltransferase component of pyruvate dehydrogenase complex, mitochondrial OS=Rattus norvegicus GN=Dlat PE=1 SV=3 - [ODP2_RAT]	67.1	0.448	0.918	0.321	0.867	0.774	0.956	0.365	0.486	0.952	0.824	1.105	1.238	2.029	4.261	1.158	3.240	0.598	1.533	1.314	1.184	0.470	1.122	0.014	0.029	0.056	0.098	0.014	0.026	0.056	0.077	0.917	0.960
P08503	Medium-chain specific acyl-CoA dehydrogenase, mitochondrial OS=Rattus norvegicus GN=Acadm PE=1 SV=1 - [ACADM_RAT]	46.5	0.645	0.622	0.692	0.793	0.695	1.019	0.606	0.737	0.674	0.761	0.698	0.822	1.562	2.680	1.083	2.236	0.841	1.131	0.990	0.996	0.605	1.103	0.022	0.044	0.014	0.042	0.085	0.093	0.056	0.0997	0.917	1.183
P08649	Complement C4 OS=Rattus norvegicus GN=C4 PE=1 SV=3 - [CO4_RAT]	192.0	0.723	0.970	0.416	1.020	0.838	0.993	0.508	0.674	0.876	0.911	0.982	1.169	2.084	2.186	1.063	2.482	0.702	1.335	1.238	1.022	0.917	1.012	0.022	0.043	0.085	0.127	0.022	0.039	0.056	0.086	0.917	1.092
P08753	Guanine nucleotide-binding protein G(k) subunit alpha OS=Rattus norvegicus GN=Gnai3 PE=1 SV=3 - [GNAI3_RAT]	40.5	1.059	0.784	0.657	1.055	0.910	1.455	0.712	0.757	0.785	0.714	1.178	1.159	2.026	3.146	1.068	2.141	0.673	2.237	1.662	0.969	0.605	0.965	0.014	0.029	0.184	0.206	0.056	0.065	0.184	0.160	0.756	1.177
P09006	Serine protease inhibitor A3N OS=Rattus norvegicus GN=Serpina3n PE=1 SV=3 - [SPA3N_RAT]	46.6	0.504	0.458	0.378	0.621	0.677	0.657	0.367	0.694	0.641	0.643	0.688	0.617	1.556	1.940	0.923	2.717	0.591	1.100	0.963	1.512	0.354	1.093	0.036	0.062	0.036	0.092	0.127	0.126	0.085	0.107	0.756	1.417
P09495	Tropomyosin alpha-4 chain OS=Rattus norvegicus GN=Tpm4 PE=1 SV=3 - [TPM4_RAT]	28.5	1.576	0.400	0.961	1.312	1.381	1.974	1.072	1.119	0.488	0.936	0.013	0.013	0.013	0.013	0.013	1.595	0.974	0.964	0.843	0.693	0.756	0.897	0.009	0.083	0.756	0.653	0.009	0.077	0.605	0.442	0.009	0.323
P10719	ATP synthase subunit beta, mitochondrial OS=Rattus norvegicus GN=Atp5b PE=1 SV=2 - [ATPB_RAT]	56.3	0.485	1.104	0.380	1.079	0.942	0.764	0.334	0.513	1.258	1.146	1.226	1.120	2.299	3.631	1.202	3.268	0.691	1.477	1.612	1.481	0.756	1.048	0.014	0.029	0.056	0.089	0.085	0.085	0.056	0.074	0.917	0.952
P10959	Carboxylesterase 1C OS=Rattus norvegicus GN=Ces1c PE=1 SV=3 - [EST1C_RAT]	60.1	0.356	1.118	0.262	0.912	0.799	0.566	0.206	0.374	1.024	0.992	1.183	0.928	2.421	2.856	1.169	3.023	0.377	1.468	1.262	1.405	0.917	1.069	0.022	0.041	0.056	0.101	0.036	0.052	0.056	0.087	0.756	1.184
P10960	Sulfated glycoprotein 1 OS=Rattus norvegicus GN=Psap PE=1 SV=1 - [SAP_RAT]	61.1	0.696	0.762	0.503	0.889	0.844	0.991	0.584	0.734	0.804	0.887	0.955	0.880	1.772	2.536	0.936	2.812	0.811	1.397	1.359	0.890	0.605	1.083	0.022	0.044	0.036	0.084	0.085	0.091	0.056	0.079	0.917	1.088
P11348	Dihydropteridine reductase OS=Rattus norvegicus GN=Qdpr PE=1 SV=1 - [DHPR_RAT]	25.5	0.575	0.851	0.409	0.874	0.856	0.839	0.433	0.581	0.918	0.997	0.933	0.960	1.841	3.517	1.028	3.097	0.588	1.240	1.201	1.190	0.605	1.099	0.014	0.033	0.056	0.103	0.036	0.051	0.056	0.081	0.917	1.026
P11442	Clathrin heavy chain 1 OS=Rattus norvegicus GN=Cltc PE=1 SV=3 - [CLH1_RAT]	191.5	0.625	0.909	0.549	0.995	0.781	1.460	0.453	0.634	0.821	0.706	1.094	1.277	1.898	3.437	1.092	2.947	0.687	1.625	1.319	1.052	0.917	1.010	0.014	0.030	0.056	0.094	0.056	0.065	0.127	0.123	0.605	1.345
P11517	Hemoglobin subunit beta-2 OS=Rattus norvegicus PE=1 SV=2 - [HBB2_RAT]	16.0	0.728	0.394	0.449	0.462	0.513	0.751	0.302	0.812	0.365	0.389	0.651	0.638	1.827	3.410	0.763	4.510	0.523	1.174	0.741	1.002	0.605	1.149	0.036	0.061	0.022	0.060	0.127	0.125	0.085	0.101	0.917	1.042
P11598	Protein disulfide-isomerase A3 OS=Rattus norvegicus GN=Pdia3 PE=1 SV=2 - [PDIA3_RAT]	56.6	0.716	0.919	0.605	0.919	0.927	1.343	0.532	0.757	0.924	0.969	0.971	1.084	1.732	1.220	1.260	1.144	0.970	1.473	1.165	0.912	0.470	1.010	0.014	0.040	0.056	0.126	0.085	0.093	0.184	0.169	0.354	1.619
P11884	Aldehyde dehydrogenase, mitochondrial OS=Rattus norvegicus GN=Aldh2 PE=1 SV=1 - [ALDH2_RAT]	56.5	0.413	0.915	0.369	0.761	0.732	0.819	0.394	0.539	0.978	0.979	0.949	1.072	1.990	3.864	1.134	3.207	0.685	1.329	1.178	1.365	0.354	1.066	0.014	0.032	0.056	0.102	0.036	0.050	0.056	0.077	0.917	0.982
P11915	Non-specific lipid-transfer protein OS=Rattus norvegicus GN=Scp2 PE=1 SV=3 - [NLTP_RAT]	58.8	0.693	0.913	0.381	1.116	0.905	0.799	0.383	0.573	0.655	0.716	1.187	0.997	2.457	3.490	0.935	3.611	0.655	1.196	1.362	1.256	0.259	0.944	0.036	0.055	0.085	0.119	0.014	0.028	0.056	0.080	0.756	1.146
P12711	Alcohol dehydrogenase class-3 OS=Rattus norvegicus GN=Adh5 PE=1 SV=2 - [ADHX_RAT]	39.6	0.807	0.904	0.644	1.033	1.024	1.258	0.583	0.776	0.866	0.839	0.908	1.060	1.868	1.495	1.084	1.366	0.963	1.175	1.064	0.929	0.605	0.983	0.036	0.060	0.085	0.131	0.056	0.074	0.085	0.108	0.605	1.639
P12785	Fatty acid synthase OS=Rattus norvegicus GN=Fasn PE=1 SV=3 - [FAS_RAT]	272.5	0.908	0.975	0.772	1.095	1.003	1.470	0.687	0.818	0.786	0.758	1.178	1.255	1.868	1.572	1.333	1.307	0.955	1.720	1.222	0.772	0.259	0.854	0.014	0.032	0.259	0.278	0.056	0.068	0.184	0.166	0.259	1.313
P12788	Trypsin-4 OS=Rattus norvegicus OX=10116 GN=Try4 PE=2 SV=1 - [TRY4_RAT]	26.6	0.391	0.900	0.330	0.880	0.681	0.847	0.365	0.405	1.094	0.779	1.726	2.861	2.911	1.201	1.022	0.013	0.013	0.013	0.013	0.013	0.756	1.117	0.014	0.030	0.009	2.051	0.022	0.037	0.009	0.883	0.009	0.071
P13255	Glycine N-methyltransferase OS=Rattus norvegicus OX=10116 GN=Gnmt PE=1 SV=2 - [GNMT_RAT]	32.5	0.577	1.139	0.472	1.127	0.992	1.044	0.471	0.647	1.162	1.177	1.281	1.380	2.449	1.191	1.315	1.124	0.765	1.566	1.415	1.403	0.605	0.970	0.014	0.033	0.127	0.167	0.014	0.028	0.127	0.128	0.917	1.117
P13635	Ceruloplasmin OS=Rattus norvegicus GN=Cp PE=1 SV=3 - [CERU_RAT]	120.8	0.576	0.817	0.509	0.892	0.947	0.756	0.393	0.724	0.982	0.933	1.033	0.849	1.747	2.052	1.098	3.180	0.692	1.572	1.021	1.162	0.917	1.038	0.036	0.061	0.056	0.095	0.036	0.056	0.085	0.096	0.917	1.081
P13676	Acylamino-acid-releasing enzyme OS=Rattus norvegicus GN=Apeh PE=1 SV=1 - [ACPH_RAT]	81.3	0.699	0.783	0.643	0.901	0.902	1.097	0.494	0.748	0.830	0.855	0.850	0.971	1.787	2.093	1.101	1.487	0.875	1.119	1.070	1.030	0.917	1.008	0.036	0.061	0.036	0.092	0.056	0.074	0.056	0.104	0.756	1.428
P14173	Aromatic-L-amino-acid decarboxylase OS=Rattus norvegicus OX=10116 GN=Ddc PE=1 SV=1 - [DDC_RAT]	54.0	0.607	0.664	0.486	0.797	0.764	1.040	0.511	0.585	0.954	0.889	0.969	0.912	1.448	3.562	0.742	2.783	0.620	1.245	1.114	0.854	0.354	1.061	0.036	0.060	0.056	0.123	0.184	0.156	0.184	0.165	0.756	1.284
P14480	Fibrinogen beta chain OS=Rattus norvegicus GN=Fgb PE=1 SV=4 - [FIBB_RAT]	54.2	0.725	0.658	0.605	0.623	0.746	0.842	0.471	0.731	1.159	0.772	0.917	0.952	1.667	2.529	0.890	4.259	0.543	1.112	0.885	1.076	0.184	0.779	0.014	0.040	0.127	0.163	0.056	0.073	0.259	0.211	0.756	1.242
P14668	Annexin A5 OS=Rattus norvegicus GN=Anxa5 PE=1 SV=3 - [ANXA5_RAT]	35.7	0.519	1.035	0.506	0.896	0.903	0.914	0.423	0.671	1.014	1.079	1.416	1.368	2.702	1.407	1.195	1.589	0.749	1.558	1.356	1.890	0.605	1.049	0.014	0.032	0.056	0.101	0.014	0.028	0.056	0.079	0.917	1.043

P14882	Propionyl-CoA carboxylase alpha chain, mitochondrial OS=Rattus norvegicus OX=10116 GN=Pcca PE=1 SV=3 - [PCCA_RAT]	81.6	0.620	0.837	0.706	0.942	0.834	1.199	0.432	0.644	0.790	0.825	0.842	1.032	1.921	1.889	1.245	1.395	0.917	1.317	1.094	1.070	0.605	1.064	0.022	0.044	0.022	0.062	0.036	0.056	0.056	0.103	0.756	1.405
P14942	Glutathione S-transferase alpha-4 OS=Rattus norvegicus GN=Gsta4 PE=1 SV=2 - [GSTA4_RAT]	25.5	0.595	0.856	0.433	0.990	0.879	0.934	0.404	0.570	0.928	0.892	1.077	1.155	1.958	3.545	1.151	2.902	0.709	1.379	1.452	1.418	0.917	1.042	0.014	0.030	0.056	0.093	0.014	0.027	0.056	0.076	0.917	0.984
P15651	Short-chain specific acyl-CoA dehydrogenase, mitochondrial OS=Rattus norvegicus GN=Acads PE=1 SV=2 - [ACADS_RAT]	44.7	0.605	0.843	0.654	0.868	0.790	1.205	0.565	0.709	0.827	0.895	0.805	0.993	1.584	3.099	1.015	2.520	0.798	1.251	1.215	1.099	0.605	1.050	0.036	0.059	0.036	0.085	0.127	0.117	0.085	0.098	0.917	1.083
P15800	Laminin subunit beta-2 OS=Rattus norvegicus GN=Lamb2 PE=2 SV=1 - [LAMB2_RAT]	196.3	0.013	0.013	0.013	0.013	0.013	0.013	0.013	0.013	0.013	0.013	0.837	0.737	1.648	1.571	0.728	0.013	0.013	0.013	0.013	0.013	#DIV/0!	#DIV/0!	0.009	0.092	#DIV/0!	#DIV/0!	0.009	0.080	#DIV/0!	#DIV/0!	0.009	0.205
P16290	Phosphoglycerate mutase 2 OS=Rattus norvegicus GN=Pgam2 PE=2 SV=2 - [PGAM2_RAT]	28.7	0.013	0.013	0.013	0.013	0.013	0.013	0.013	0.013	0.013	0.013	1.501	1.667	2.363	2.585	0.916	0.013	0.013	0.013	0.013	0.013	#DIV/0!	#DIV/0!	0.009	0.036	#DIV/0!	#DIV/0!	0.009	0.028	#DIV/0!	#DIV/0!	0.009	0.073
P16446	Phosphatidylinositol transfer protein alpha isoform OS=Rattus norvegicus GN=Pitpna PE=1 SV=2 - [PIPNA_RAT]	31.9	0.811	0.952	0.640	1.050	0.923	1.226	0.507	0.699	0.831	0.942	1.048	1.053	1.989	1.967	0.970	2.012	0.900	1.294	1.301	1.050	0.756	0.973	0.036	0.058	0.056	0.102	0.056	0.071	0.056	0.086	0.917	1.137
P17220	Proteasome subunit alpha type-2 OS=Rattus norvegicus GN=Psma2 PE=1 SV=3 - [PSA2_RAT]	25.9	0.400	1.093	0.544	0.759	0.707	0.689	0.333	0.480	1.174	1.318	1.232	1.798	2.563	2.526	1.567	1.960	0.437	1.840	1.286	1.637	0.917	1.041	0.014	0.029	0.085	0.127	0.022	0.036	0.127	0.126	0.470	1.413
P17702	60S ribosomal protein L28 OS=Rattus norvegicus GN=Rpl28 PE=1 SV=4 - [RL28_RAT]	15.8	0.013	0.013	0.013	0.013	0.013	0.013	0.013	0.013	0.013	0.013	1.046	1.133	1.930	0.186	1.136	0.013	0.013	0.013	0.013	0.013	#DIV/0!	#DIV/0!	0.009	0.097	#DIV/0!	#DIV/0!	0.009	0.083	#DIV/0!	#DIV/0!	0.009	0.226
P18266	Glycogen synthase kinase-3 beta OS=Rattus norvegicus GN=Gsk3b PE=1 SV=1 - [GSK3B_RAT]	46.7	0.013	0.013	0.013	0.013	0.013	0.013	0.013	0.013	0.013	0.013	2.114	1.772	3.349	4.342	1.332	0.013	0.013	0.013	0.013	0.013	#DIV/0!	#DIV/0!	0.009	0.027	#DIV/0!	#DIV/0!	0.009	0.022	#DIV/0!	#DIV/0!	0.009	0.062
P18297	Sepiapterin reductase OS=Rattus norvegicus GN=Spr PE=1 SV=1 - [SPRE_RAT]	28.1	0.411	0.920	0.333	0.919	0.940	0.782	0.308	0.606	0.956	1.002	1.011	1.009	2.088	3.175	1.088	2.531	0.669	1.256	1.192	1.115	0.756	1.100	0.014	0.033	0.056	0.118	0.014	0.028	0.056	0.092	0.917	1.047
P18421	Proteasome subunit beta type-1 OS=Rattus norvegicus GN=Psmb1 PE=1 SV=3 - [PSB1_RAT]	26.5	0.550	0.738	0.626	0.823	0.791	1.139	0.420	0.664	0.790	0.907	0.785	1.114	1.780	1.762	1.210	1.456	0.915	1.284	1.005	1.082	0.605	1.091	0.036	0.061	0.014	0.043	0.085	0.094	0.056	0.103	0.470	1.645
P18614	Integrin alpha-1 OS=Rattus norvegicus GN=Itga1 PE=1 SV=1 - [ITA1_RAT]	130.7	0.013	0.013	0.013	0.013	0.013	0.013	0.013	0.013	0.013	0.013	1.096	0.737	1.959	4.101	1.236	0.013	0.013	0.013	0.013	0.013	#DIV/0!	#DIV/0!	0.009	0.036	#DIV/0!	#DIV/0!	0.009	0.028	#DIV/0!	#DIV/0!	0.009	0.073
P18886	Carnitine O-palmitoyltransferase 2, mitochondrial OS=Rattus norvegicus OX=10116 GN=Cpt2 PE=1 SV=1 - [CPT2_RAT]	74.1	0.626	0.928	0.601	0.879	0.761	1.472	0.579	0.705	0.729	0.733	1.004	1.093	1.969	2.106	0.855	1.809	0.920	1.298	1.201	0.786	0.756	1.043	0.036	0.060	0.056	0.125	0.056	0.071	0.085	0.104	0.605	1.514
P19112	Fructose-1,6-bisphosphatase 1 OS=Rattus norvegicus OX=10116 GN=Fbp1 PE=1 SV=2 - [F16P1_RAT]	39.6	0.212	0.597	0.168	0.643	0.567	0.438	0.193	0.262	0.681	0.702	0.809	0.866	1.428	7.031	0.709	6.363	0.339	1.088	1.061	0.906	0.605	1.153	0.014	0.029	0.056	0.091	0.014	0.026	0.056	0.074	0.917	0.925
P19356	Porphobilinogen deaminase OS=Rattus norvegicus GN=Hmbs PE=1 SV=2 - [HEM3_RAT]	39.3	0.515	1.033	0.472	0.999	0.945	1.010	0.528	0.669	1.006	0.999	1.513	1.358	3.041	1.688	1.261	1.721	0.738	1.663	1.737	1.650	0.605	1.028	0.014	0.030	0.056	0.094	0.014	0.026	0.056	0.075	0.756	1.172
P19804	Nucleoside diphosphate kinase B OS=Rattus norvegicus GN=Nme2 PE=1 SV=1 - [NDKB_RAT]	17.3	0.288	1.519	0.199	1.226	1.112	0.760	0.255	0.313	1.355	1.312	1.629	1.494	2.976	1.614	1.359	1.535	0.506	1.882	1.881	1.570	0.917	0.988	0.036	0.055	0.056	0.092	0.014	0.026	0.056	0.078	0.917	0.998
P20611	Lysosomal acid phosphatase OS=Rattus norvegicus GN=Acp2 PE=1 SV=1 - [PPAL_RAT]	48.3	0.013	0.013	0.013	0.013	0.013	0.867	0.440	0.516	1.067	0.979	1.006	1.116	1.842	5.021	0.857	4.029	0.482	1.264	1.468	1.020	0.009	0.269	0.009	0.032	0.009	0.036	0.085	0.085	0.127	0.123	0.917	0.948
P20673	Argininosuccinate lyase OS=Rattus norvegicus GN=Asl PE=2 SV=1 - [ARLY_RAT]	51.5	0.372	0.807	0.309	0.764	0.676	0.750	0.329	0.514	0.654	0.771	0.808	0.800	1.392	4.721	0.857	3.552	0.565	1.057	0.991	0.902	0.917	1.073	0.022	0.042	0.056	0.122	0.014	0.029	0.056	0.098	0.917	1.024
P20760	Ig gamma-2A chain C region OS=Rattus norvegicus GN=Igg-2a PE=1 SV=1 - [IGG2A_RAT]	35.2	0.335	0.557	0.487	0.555	0.579	1.096	0.406	0.895	1.374	1.311	0.949	0.647	1.428	1.547	0.839	2.864	0.838	1.473	0.948	1.298	0.085	0.436	0.014	0.041	0.014	0.041	0.756	0.550	0.470	0.349	0.605	1.538
P20761	Ig gamma-2B chain C region OS=Rattus norvegicus GN=Igh-1a PE=1 SV=1 - [IGG2B_RAT]	36.5	0.631	0.757	0.503	0.519	0.513	1.054	0.566	0.587	0.930	0.987	0.820	0.758	1.452	1.301	1.123	2.356	0.787	1.424	1.095	0.853	0.085	0.439	0.014	0.040	0.014	0.042	0.184	0.164	0.184	0.165	0.756	1.471
P20767	Ig lambda-2 chain C region OS=Rattus norvegicus PE=4 SV=1 - [LAC2_RAT]	11.3	0.472	0.633	0.372	0.628	0.697	1.256	0.555	0.788	1.145	1.206	0.909	0.799	1.468	1.560	0.845	2.638	1.112	1.518	0.929	1.166	0.056	0.295	0.014	0.040	0.014	0.040	0.470	0.355	0.354	0.273	0.259	1.322
P21670	Proteasome subunit alpha type-4 OS=Rattus norvegicus GN=Psma4 PE=1 SV=1 - [PSA4_RAT]	29.5	0.374	1.040	0.307	0.833	0.726	0.572	0.263	0.372	1.035	1.125	1.223	1.515	2.561	2.167	1.513	2.150	0.400	1.825	1.474	1.700	0.917	1.068	0.014	0.031	0.056	0.104	0.014	0.027	0.056	0.083	0.605	1.339
P21708	Mitogen-activated protein kinase 3 OS=Rattus norvegicus GN=Mapk3 PE=1 SV=5 - [MK03_RAT]	43.1	0.013	0.013	0.013	0.013	0.013	1.223	0.620	0.891	0.770	0.973	0.793	0.711	1.682	1.330	0.922	1.300	1.023	0.944	1.009	0.893	0.009	0.095	0.009	0.096	0.009	0.095	0.470	0.358	0.184	0.173	0.756	1.565
P21775	3-ketoacyl-CoA thiolase A, peroxisomal OS=Rattus norvegicus GN=Acaa1a PE=1 SV=2 - [THIKA_RAT]	43.8	0.439	0.701	0.349	0.900	0.759	0.721	0.315	0.540	0.790	0.924	0.900	0.857	1.751	4.410	0.862	4.125	0.519	1.242	1.232	1.057	0.756	1.121	0.036	0.058	0.056	0.096	0.056	0.067	0.085	0.096	0.917	0.978
P22734	Catechol O-methyltransferase OS=Rattus norvegicus GN=Comt PE=1 SV=2 - [COMT_RAT]	29.6	0.337	0.606	0.265	0.665	0.671	0.526	0.258	0.311	0.551	0.582	0.984	0.985	1.756	6.933	0.782	5.726	0.329	1.340	1.098	1.106	0.259	0.962	0.014	0.028	0.085	0.119	0.014	0.025	0.056	0.075	0.917	0.923
P22791	Hydroxymethylglutaryl-CoA synthase, mitochondrial OS=Rattus norvegicus OX=10116 GN=Hmgcs2 PE=1 SV=1 - [HMCS2_RAT]	56.9	0.013	0.013	0.013	0.013	0.013	0.013	0.013	0.013	0.013	0.013	0.826	1.037	1.851	1.367	0.808	0.013	0.013	0.013	0.013	0.013	#DIV/0!	#DIV/0!	0.009	0.078	#DIV/0!	#DIV/0!	0.009	0.068	#DIV/0!	#DIV/0!	0.009	0.159
P22985	Xanthine dehydrogenase/oxidase OS=Rattus norvegicus GN=Xdh PE=1 SV=3 - [XDH_RAT]	146.1	0.565	0.943	0.487	0.796	0.783	0.889	0.465	0.588	0.889	0.995	1.096	1.222	2.167	1.690	1.134	1.479	0.703	1.293	1.091	1.235	0.605	1.094	0.014	0.038	0.056	0.128	0.014	0.031	0.056	0.104	0.470	1.577
P23457	3-alpha-hydroxysteroid dehydrogenase OS=Rattus norvegicus GN=Akr1c9 PE=1 SV=1 - [DIDH_RAT]	37.0	0.615	1.116	0.479	1.186	1.146	0.994	0.395	0.591	1.161	1.236	1.188	1.175	2.375	1.283	1.251	1.531	0.819	1.723	1.375	1.115	0.917	0.945	0.022	0.042	0.184	0.213	0.036	0.053	0.127	0.127	0.917	1.120
P23965	Enoyl-CoA delta isomerase 1, mitochondrial OS=Rattus norvegicus GN=Eci1 PE=1 SV=1 - [ECI1_RAT]	32.2	0.611	0.827	0.577	0.869	0.734	1.087	0.491	0.619	0.723	0.843	0.838	0.934	1.673	3.105	1.092	2.362	0.854	1.236	1.065	0.942	0.917	1.045	0.022	0.043	0.022	0.060	0.056	0.069	0.056	0.096	0.917	1.104
P24155	Thimet oligopeptidase OS=Rattus norvegicus GN=Thop1 PE=1 SV=4 - [THOP1_RAT]	78.3	0.598	0.935	0.442	0.935	0.979	1.084	0.439	0.562	1.073	1.063	1.016	1.244	2.113	1.532	1.299	1.369	0.830	1.442	1.290	1.395	0.470	1.061	0.014	0.037	0.056	0.119	0.056	0.069	0.056	0.089	0.756	1.339
P24329	Thiosulfate sulfurtransferase OS=Rattus norvegicus GN=Tst PE=1 SV=3 - [THTR_RAT]	33.4	0.613	0.973	0.546	0.967	0.897	1.073	0.470	0.656	0.898	1.000	0.947	0.987	1.890	2.635	1.000	2.153	0.811	1.256	1.141	1.072	0.605	1.034	0.036	0.058	0.056	0.115	0.127	0.118	0.085	0.102	0.917	1.116
P24464	Cytochrome P450 4A12 OS=Rattus norvegicus OX=10116 GN=Cyp4a12 PE=2 SV=2 - [CP4AC_RAT]	58.5	0.013	0.013	0.013	0.013	0.013	0.013	0.013	0.013	0.013	0.013	0.524	0.538	0.929	7.509	0.440	0.013	0.013	0.013	0.013	0.013	#DIV/0!	#DIV/0!	0.009	0.032	#DIV/0!	#DIV/0!	0.009	0.025	#DIV/0!	#DIV/0!	0.009	0.069
P25031	Regenerating islet-derived protein 3-beta OS=Rattus norvegicus OX=10116 GN=Reg3b PE=1 SV=1 - [REG3B_RAT]	19.6	0.013	0.013	0.013	0.013	0.013	0.013	0.013	0.013	0.013	0.013	1.107	0.789	2.940	7.594	0.886	0.013	0.013	0.013	0.013	0.013	#DIV/0!	#DIV/0!	0.009	0.027	#DIV/0!	#DIV/0!	0.009	0.022	#DIV/0!	#DIV/0!	0.009	0.061
P25093	Fumarylacetoacetase OS=Rattus norvegicus GN=Fah PE=1 SV=1 - [FAAA_RAT]	45.9	0.419	0.673	0.355	0.676	0.689	0.709	0.357	0.469	0.668	0.708	0.838	0.774	1.380	5.202	0.727	5.219	0.477	1.009	0.991	1.022	0.605	1.139	0.014	0.033	0.056	0.094	0.014	0.029	0.056	0.076	0.756	1.135
P25113	Phosphoglycerate mutase 1 OS=Rattus norvegicus GN=Pgam1 PE=1 SV=4 - [PGAM1_RAT]	28.8	0.466	1.076	0.363	0.983	0.958	0.975	0.335	0.545	1.054	1.058	1.080	1.153	2.227	2.862	1.084	2.357	0.635	1.342	1.354	1.320	0.917	1.022	0.014	0.031	0.056	0.103	0.014	0.027	0.056	0.083	0.917	1.036
P26644	Beta-2-glycoprotein 1 OS=Rattus norvegicus GN=Apoh PE=2 SV=2 - [APOH_RAT]	33.2	0.013	0.013	0.013	0.013	0.013	1.124	0.637	0.551	0.651	0.576	0.901	0.743	1.355	2.637	0.636	2.501	0.819	1.188	1.090	0.679	0.009	0.599	0.009	0.070	0.009	0.060	0.127	0.125	0.056	0.102	0.917	1.216
P27008	Poly [ADP-ribose] polymerase 1 OS=Rattus norvegicus GN=Parp1 PE=1 SV=4 - [PARP1_RAT]	112.6	0.013	0.013	0.013	0.013	0.013	0.013	0.013	0.013	0.013	0.013	1.161	1.419	2.534	1.866	0.979	0.013	0.013	0.013	0.013	0.013	#DIV/0!	#DIV/0!	0.009	0.047	#DIV/0!	#DIV/0!	0.009	0.037	#DIV/0!	#DIV/0!	0.009	0.085
P27139	Carbonic anhydrase 2 OS=Rattus norvegicus GN=Ca2 PE=1 SV=2 - [CAH2_RAT]	29.1	0.495	0.468	0.377	0.546	0.576	0.564	0.310	0.716	0.585	0.519	0.710	0.637	1.612	4.724	0.651	5.748	0.485	0.981	0.887	1.082	0.354	1.096	0.014	0.038	0.056	0.093	0.056	0.073	0.085	0.093	0.756	1.140
P27213	6-pyruvoyl tetrahydrobiopterin synthase OS=Rattus norvegicus OX=10116 GN=Pts PE=1 SV=1 - [PTPS_RAT]	16.2	0.013	0.013	0.013	0.013	0.013	0.013	0.013	0.013	0.013	0.013	1.021	1.059	2.080	2.867	1.071	1.161	0.703	0.910	0.858	0.546	#DIV/0!	#DIV/0!	0.009	0.044	0.009	0.354	0.009	0.035	0.009	0.245	0.056	0.353
P27867	Sorbitol dehydrogenase OS=Rattus norvegicus GN=Sord PE=1 SV=4 - [DHSO_RAT]	38.2	0.457	0.417	0.419	0.561	0.545	0.704	0.363	0.440	0.519	0.557	0.637	0.662	0.997	6.471	0.628	4.788	0.457	0.898	0.806	0.691	0.756	1.132	0.014	0.033	0.056	0.122	0.056	0.067	0.085	0.104	0.917	0.974
P28075	Proteasome subunit beta type-5 OS=Rattus norvegicus GN=Psmb5 PE=1 SV=3 - [PSB5_RAT]	28.6	0.641	0.751	0.668	0.901	0.810	1.125	0.542	0.691	0.859	0.979	1.165	1.191	1.918	1.876	1.306	1.823	0.695	1.563	1.044	0.971	0.470	1.075	0.014	0.036	0.056	0.124	0.014	0.029	0.184	0.168	0.184	1.042
P28480	T-complex protein 1 subunit alpha OS=Rattus norvegicus GN=Tcp1 PE=1 SV=1 - [TCPA_RAT]	60.3	0.855	0.978	0.747	1.121	0.984	1.389	0.591	0.733	0.898	0.770	1.032	1.204	1.753	2.340	1.128	1.844	0.918	1.363	1.300	0.841	0.354	0.908	0.022	0.041	0.259	0.277	0.056	0.067	0.127	0.128	0.605	1.474
P28492	Glutaminase liver isoform, mitochondrial OS=Rattus norvegicus GN=Gls2 PE=2 SV=3 - [GLSL_RAT]	66.2	0.678	0.832	0.725	1.096	0.874	1.334	0.504	0.744	0.627	0.811	0.897	1.354	2.139	1.768	1.105	1.172	0.952	1.428	0.809	0.709	0.470	1.049	0.022	0.043	0.354	0.367	0.036	0.054	0.259	0.222	0.184	1.054
P29266	3-hydroxyisobutyrate dehydrogenase, mitochondrial OS=Rattus norvegicus GN=Hibadh PE=1 SV=3 - [3HIDH_RAT]	35.3	0.516	0.932	0.509	0.941	0.795	0.933	0.369	0.571	1.005	1.103	0.959	1.062	1.932	2.827	1.112	2.427	0.845	1.299	1.222	1.138	0.470	1.087	0.014	0.034	0.036	0.085	0.056	0.067	0.056	0.083	0.917	1.062
P29410	Adenylate kinase 2, mitochondrial OS=Rattus norvegicus GN=Ak2 PE=2 SV=2 - [KAD2_RAT]	26.4	0.653	1.003	0.546	1.048	0.949	0.942	0.509	0.646	1.011	0.984	1.122	1.073	2.259	2.683	1.021	2.342	0.800	1.365	1.392	1.167	0.605	1.017	0.022	0.041	0.056	0.096	0.014	0.028	0.056	0.080	0.917	1.043
P30713	Glutathione S-transferase theta-2 OS=Rattus norvegicus GN=Gstt2 PE=1 SV=3 - [GSTT2_RAT]	27.4	0.555	1.083	0.483	0.927	0.827	1.188	0.480	0.669	1.019	0.925	1.345	1.452	1.757	3.003	1.197	2.662	0.506	1.810	1.465	0.686	0.756	1.027	0.014	0.030	0.259	0.276	0.014	0.026	0.259	0.211	0.756	1.189
P30839	Fatty aldehyde dehydrogenase OS=Rattus norvegicus GN=Aldh3a2 PE=1 SV=1 - [AL3A2_RAT]	54.0	1.117	0.713	0.623	0.836	0.734	2.010	0.733	0.460	0.688	0.606	1.459	1.144	1.808	2.035	0.833	1.503	0.717	1.752	1.135	0.736	0.354	0.968	0.036	0.059	0.127	0.171	0.085	0.088	0.184	0.167	0.259	1.317
P31977	Ezrin OS=Rattus norvegicus GN=Ezr PE=1 SV=3 - [EZRI_RAT]	69.3	0.482	0.495	0.435	0.579	0.555	0.653	0.328	0.497	0.494	0.475	0.640	0.665	1.220	6.878	0.641	6.064	0.476	0.918	0.835	0.771	0.756	1.131	0.014	0.030	0.085	0.121	0.036	0.051	0.056	0.077	0.917	0.936
P32232	Cystathionine beta-synthase OS=Rattus norvegicus GN=Cbs PE=1 SV=3 - [CBS_RAT]	61.4	0.659	0.868	0.557	0.998	0.885	1.301	0.528	0.672	0.906	1.012	1.140	1.226	2.235	1.452	1.228	1.275	0.955	1.322	1.171	1.186	0.470	1.034	0.014	0.036	0.022	0.061	0.056	0.068	0.127	0.131	0.354	1.510
P32362	Uroporphyrinogen decarboxylase (Fragment) OS=Rattus norvegicus GN=Urod PE=1 SV=1 - [DCUP_RAT]	40.4	0.609	1.624	0.395	0.956	0.577	0.721	0.662	0.325	1.233	1.137	1.564	1.604	3.086	1.637	1.697	1.946	0.632	1.603	1.791	1.720	0.756	1.016	0.036	0.054	0.056	0.091	0.014	0.025	0.085	0.094	0.917	0.969
P32821	Trypsin V-A OS=Rattus norvegicus OX=10116 PE=2 SV=1 - [TRYA_RAT]	26.9	0.459	0.694	0.367	0.883	0.488	1.170	0.354	0.323	1.395	0.898	2.682	2.899	3.931	0.705	1.006	0.507	0.616	1.135	1.032	0.852	0.605	1.108	0.022	0.039	0.127	0.174	0.127	0.114	0.917	0.657	0.127	0.754
P34058	Heat shock protein HSP 90-beta OS=Rattus norvegicus GN=Hsp90ab1 PE=1 SV=4 - [HS90B_RAT]	83.2	0.761	0.977	0.635	1.049	0.895	1.251	0.549	0.742	0.921	0.841	1.174	1.283	2.070	2.579	1.033	2.341	0.709	1.649	1.398	1.038	0.756	0.965	0.022	0.040	0.127	0.162	0.036	0.051	0.127	0.125	0.756	1.221
P34067	Proteasome subunit beta type-4 OS=Rattus norvegicus GN=Psmb4 PE=1 SV=2 - [PSB4_RAT]	29.2	0.354	0.993	0.455	0.841	0.853	0.875	0.401	0.499	1.073	0.970	0.951	1.162	1.893	1.797	1.319	1.637	0.752	1.413	1.089	1.089	0.470	1.098	0.022	0.044	0.056	0.127	0.036	0.055	0.056	0.102	0.354	1.519
P35704	Peroxiredoxin-2 OS=Rattus norvegicus GN=Prdx2 PE=1 SV=3 - [PRDX2_RAT]	21.8	0.257	0.882	0.355	0.801	0.875	0.621	0.185	0.320	0.986	0.984	0.895	0.929	2.158	3.123	1.041	3.529	0.492	1.252	1.144	1.516	0.917	1.071	0.014	0.036	0.056	0.099	0.085	0.091	0.056	0.082	0.756	1.186
P35738	2-oxoisovalerate dehydrogenase subunit beta, mitochondrial OS=Rattus norvegicus OX=10116 GN=Bckdhb PE=1 SV=3 - [ODBB_RAT]	42.8	0.649	1.024	0.554	1.088	0.985	1.210	0.424	0.729	0.946	0.961	1.069	1.287	2.182	2.294	1.307	1.994	0.637	1.707	1.530	1.352	0.756	0.974	0.022	0.041	0.085	0.121	0.022	0.038	0.085	0.095	0.917	1.038
P35815	Protein phosphatase 1B OS=Rattus norvegicus GN=Ppm1b PE=2 SV=1 - [PPM1B_RAT]	42.9	0.013	0.013	0.013	0.013	0.013	1.075	0.203	0.525	0.941	0.940	0.839	0.900	1.504	2.219	0.877	0.013	0.013	0.013	0.013	0.013	0.009	0.348	0.009	0.067	#DIV/0!	#DIV/0!	0.470	0.357	0.009	0.736	0.009	0.134
P36970	Phospholipid hydroperoxide glutathione peroxidase, mitochondrial OS=Rattus norvegicus GN=Gpx4 PE=1 SV=3 - [GPX41_RAT]	22.2	0.013	0.013	0.013	0.013	0.013	0.013	0.013	0.013	0.013	0.013	0.787	0.928	1.396	1.700	0.679	0.013	0.013	0.013	0.013	0.013	#DIV/0!	#DIV/0!	0.009	0.092	#DIV/0!	#DIV/0!	0.009	0.081	#DIV/0!	#DIV/0!	0.009	0.208
P36972	Adenine phosphoribosyltransferase OS=Rattus norvegicus GN=Aprt PE=1 SV=1 - [APT_RAT]	19.5	0.481	1.171	0.433	1.130	1.097	1.002	0.291	0.694	1.103	1.127	1.145	1.219	2.281	1.864	1.264	1.779	0.711	1.434	1.532	1.504	0.605	1.000	0.022	0.041	0.056	0.096	0.014	0.029	0.056	0.080	0.917	1.069
P38062	Methionine aminopeptidase 2 OS=Rattus norvegicus GN=Metap2 PE=1 SV=2 - [MAP2_RAT]	53.0	0.845	0.919	0.697	1.040	0.987	0.982	0.475	0.574	0.985	1.029	1.169	1.314	2.188	1.286	1.460	1.274	0.869	1.673	1.400	1.098	0.605	0.999	0.014	0.033	0.056	0.105	0.014	0.030	0.056	0.093	0.354	1.479
P38650	Cytoplasmic dynein 1 heavy chain 1 OS=Rattus norvegicus GN=Dync1h1 PE=1 SV=1 - [DYHC1_RAT]	531.9	0.734	1.212	0.355	1.085	0.957	1.656	0.629	0.671	0.804	0.840	1.535	1.374	1.946	1.718	1.014	2.103	0.698	2.097	1.635	1.039	0.756	0.943	0.036	0.058	0.184	0.207	0.056	0.066	0.127	0.122	0.756	1.225
P38656	Lupus La protein homolog OS=Rattus norvegicus GN=Ssb PE=1 SV=1 - [LA_RAT]	47.7	0.726	1.170	0.401	1.000	1.133	0.749	0.346	0.473	0.859	1.222	1.687	1.473	2.337	2.217	1.088	2.381	0.743	1.997	1.764	1.314	0.605	1.037	0.036	0.055	0.056	0.089	0.022	0.038	0.056	0.075	0.917	0.977
P40112	Proteasome subunit beta type-3 OS=Rattus norvegicus GN=Psmb3 PE=1 SV=1 - [PSB3_RAT]	22.9	0.497	1.035	0.367	1.003	0.926	0.888	0.308	0.538	1.136	1.193	0.939	1.251	2.053	1.898	1.521	1.609	0.884	1.480	1.261	1.396	0.756	1.065	0.036	0.058	0.056	0.114	0.036	0.052	0.056	0.087	0.470	1.474
P40307	Proteasome subunit beta type-2 OS=Rattus norvegicus GN=Psmb2 PE=1 SV=1 - [PSB2_RAT]	22.9	0.652	0.891	0.653	0.930	0.739	1.143	0.496	0.735	0.970	1.038	0.946	1.008	2.084	1.359	1.287	1.282	0.900	1.409	1.197	1.127	0.354	0.992	0.014	0.039	0.022	0.061	0.127	0.120	0.085	0.104	0.605	1.555
P42676	Neurolysin, mitochondrial OS=Rattus norvegicus GN=Nln PE=1 SV=1 - [NEUL_RAT]	80.2	0.544	1.015	0.395	1.020	0.933	1.106	0.482	0.530	0.975	1.090	1.004	1.152	2.068	1.682	1.379	1.447	0.898	1.283	1.117	1.424	0.605	1.036	0.036	0.059	0.056	0.122	0.036	0.053	0.056	0.093	0.470	1.551
P42854	Regenerating islet-derived protein 3-gamma OS=Rattus norvegicus OX=10116 GN=Reg3g PE=1 SV=1 - [REG3G_RAT]	19.1	0.013	0.013	0.013	0.013	0.013	0.013	0.013	0.013	0.013	0.013	0.839	0.617	1.529	6.039	0.785	0.013	0.013	0.013	0.013	0.013	#DIV/0!	#DIV/0!	0.009	0.032	#DIV/0!	#DIV/0!	0.009	0.025	#DIV/0!	#DIV/0!	0.009	0.070
P45592	Cofilin-1 OS=Rattus norvegicus GN=Cfl1 PE=1 SV=3 - [COF1_RAT]	18.5	0.481	1.058	0.358	1.088	0.757	0.773	0.437	0.429	1.045	0.883	1.226	1.099	2.693	2.435	0.970	2.085	0.569	1.456	1.439	1.286	0.756	1.094	0.036	0.057	0.056	0.111	0.022	0.038	0.056	0.092	0.917	1.041
P45953	Very long-chain specific acyl-CoA dehydrogenase, mitochondrial OS=Rattus norvegicus OX=10116 GN=Acadvl PE=1 SV=1 - [ACADV_RAT]	70.7	0.791	0.899	0.594	0.954	0.740	1.344	0.486	0.563	0.829	0.864	1.143	1.094	1.839	3.207	0.869	2.929	0.711	1.599	1.365	0.864	0.756	1.037	0.036	0.057	0.184	0.210	0.056	0.066	0.127	0.124	0.605	1.370
P46462	Transitional endoplasmic reticulum ATPase OS=Rattus norvegicus GN=Vcp PE=1 SV=3 - [TERA_RAT]	89.3	0.609	1.147	0.432	1.038	1.024	0.934	0.362	0.559	1.041	1.093	1.193	1.229	2.601	1.467	1.210	1.432	0.824	1.470	1.325	1.426	0.756	1.018	0.014	0.032	0.056	0.107	0.014	0.029	0.056	0.091	0.917	1.097
P46844	Biliverdin reductase A OS=Rattus norvegicus GN=Blvra PE=1 SV=1 - [BIEA_RAT]	33.5	0.654	0.861	0.469	0.855	0.828	0.954	0.495	0.616	0.834	0.877	0.860	0.911	1.629	3.570	0.886	3.837	0.753	1.331	1.215	1.023	0.605	1.093	0.022	0.042	0.056	0.092	0.085	0.089	0.056	0.075	0.756	1.187
P47245	Nardilysin OS=Rattus norvegicus GN=Nrd1 PE=1 SV=1 - [NRDC_RAT]	132.9	0.420	1.052	0.280	0.970	1.068	0.845	0.312	0.497	0.943	0.989	1.110	1.164	2.253	2.553	1.465	2.022	0.618	1.635	1.480	1.373	0.605	1.096	0.014	0.031	0.056	0.102	0.014	0.028	0.056	0.087	0.756	1.205
P48037	Annexin A6 OS=Rattus norvegicus GN=Anxa6 PE=1 SV=2 - [ANXA6_RAT]	75.7	0.637	1.026	0.513	1.021	0.989	1.085	0.444	0.639	0.941	1.101	1.131	1.264	2.365	1.214	1.304	1.055	1.086	1.533	1.336	0.516	0.756	1.000	0.014	0.035	0.085	0.132	0.014	0.030	0.259	0.220	0.354	1.546
P48199	C-reactive protein OS=Rattus norvegicus GN=Crp PE=1 SV=1 - [CRP_RAT]	25.5	0.218	0.874	0.156	0.821	0.752	0.536	0.199	0.300	0.910	0.927	1.283	1.054	2.181	3.294	0.993	4.814	0.372	1.871	1.356	1.584	0.605	1.140	0.014	0.034	0.056	0.089	0.014	0.029	0.056	0.073	0.756	1.114
P48721	Stress-70 protein, mitochondrial OS=Rattus norvegicus GN=Hspa9 PE=1 SV=3 - [GRP75_RAT]	73.8	0.620	0.911	0.541	0.977	0.872	1.124	0.485	0.609	0.964	0.973	0.952	1.084	1.965	2.952	0.991	2.525	0.810	1.423	1.198	1.040	0.756	1.034	0.022	0.041	0.056	0.102	0.127	0.116	0.085	0.097	0.917	1.056
P49186	Mitogen-activated protein kinase 9 OS=Rattus norvegicus GN=Mapk9 PE=1 SV=1 - [MK09_RAT]	48.0	0.924	1.113	0.991	1.119	1.335	0.013	0.013	0.013	0.013	0.013	1.178	1.580	3.210	1.604	1.201	0.013	0.013	0.013	0.013	0.013	0.009	0.054	0.036	0.054	0.009	0.079	0.009	0.031	#DIV/0!	#DIV/0!	0.009	0.076
P49432	Pyruvate dehydrogenase E1 component subunit beta, mitochondrial OS=Rattus norvegicus GN=Pdhb PE=1 SV=2 - [ODPB_RAT]	39.0	0.450	0.928	0.406	0.849	0.777	0.842	0.303	0.491	0.915	0.851	0.929	1.070	1.893	3.967	1.098	3.635	0.529	1.448	1.299	1.139	0.917	1.065	0.014	0.031	0.056	0.094	0.014	0.027	0.056	0.077	0.917	0.976
P50137	Transketolase OS=Rattus norvegicus GN=Tkt PE=1 SV=1 - [TKT_RAT]	67.6	0.474	1.025	0.387	0.919	0.935	0.895	0.332	0.521	0.893	0.992	1.064	1.108	2.236	3.441	1.112	2.974	0.557	1.550	1.342	1.257	0.605	1.098	0.014	0.030	0.056	0.095	0.014	0.027	0.056	0.079	0.917	0.988
P50398	Rab GDP dissociation inhibitor alpha OS=Rattus norvegicus GN=Gdi1 PE=1 SV=1 - [GDIA_RAT]	50.5	0.577	0.936	0.492	0.949	0.912	0.881	0.408	0.598	0.944	1.034	1.081	1.166	2.050	2.335	1.214	2.217	0.605	1.461	1.415	1.436	0.917	1.030	0.014	0.034	0.056	0.101	0.014	0.029	0.056	0.082	0.917	1.054
P50399	Rab GDP dissociation inhibitor beta OS=Rattus norvegicus GN=Gdi2 PE=1 SV=2 - [GDIB_RAT]	50.5	0.604	1.017	0.491	0.921	0.897	1.103	0.409	0.611	0.880	1.025	1.115	1.103	2.125	2.419	1.013	2.335	0.683	1.464	1.466	1.349	0.756	1.059	0.022	0.042	0.056	0.098	0.056	0.068	0.056	0.078	0.917	1.050
P50475	Alanine--tRNA ligase, cytoplasmic OS=Rattus norvegicus GN=Aars PE=1 SV=3 - [SYAC_RAT]	106.7	0.787	1.051	0.508	1.104	0.897	1.480	0.593	0.744	0.929	0.626	1.194	1.237	1.843	1.347	1.019	1.462	0.687	1.848	1.397	0.629	0.756	0.958	0.036	0.060	0.354	0.358	0.085	0.091	0.354	0.279	0.917	1.207
P50554	4-aminobutyrate aminotransferase, mitochondrial OS=Rattus norvegicus GN=Abat PE=1 SV=3 - [GABT_RAT]	56.4	0.295	0.326	0.231	0.437	0.394	0.540	0.269	0.436	0.458	0.503	0.600	0.691	1.083	6.980	0.630	6.082	0.404	0.955	0.835	0.768	0.127	0.625	0.014	0.034	0.022	0.059	0.014	0.028	0.085	0.096	0.917	0.937
P51635	Alcohol dehydrogenase [NADP(+)] OS=Rattus norvegicus GN=Akr1a1 PE=1 SV=2 - [AK1A1_RAT]	36.5	0.501	0.701	0.396	0.723	0.671	0.720	0.347	0.489	0.696	0.704	0.771	0.777	1.326	6.355	0.683	5.139	0.476	0.966	0.948	0.940	0.917	1.072	0.036	0.055	0.085	0.121	0.056	0.065	0.085	0.096	0.917	0.945
P51650	Succinate-semialdehyde dehydrogenase, mitochondrial OS=Rattus norvegicus GN=Aldh5a1 PE=1 SV=2 - [SSDH_RAT]	56.1	0.013	0.013	0.013	0.013	0.013	1.025	0.400	0.590	1.000	1.080	0.908	1.107	2.085	2.160	1.140	1.737	0.813	1.464	1.238	0.959	0.009	0.171	0.009	0.051	0.009	0.062	0.056	0.069	0.184	0.168	0.605	1.484
P51869	Cytochrome P450 4F4 OS=Rattus norvegicus OX=10116 GN=Cyp4f4 PE=2 SV=1 - [CP4F4_RAT]	60.0	0.013	0.013	0.013	0.013	0.013	0.013	0.013	0.013	0.013	0.013	1.174	1.174	1.592	5.101	1.322	0.013	0.013	0.013	0.013	0.013	#DIV/0!	#DIV/0!	0.009	0.031	#DIV/0!	#DIV/0!	0.009	0.024	#DIV/0!	#DIV/0!	0.009	0.067
P52296	Importin subunit beta-1 OS=Rattus norvegicus GN=Kpnb1 PE=1 SV=1 - [IMB1_RAT]	97.1	0.767	0.946	0.539	0.955	0.762	1.215	0.529	0.542	0.932	0.758	1.574	1.433	2.409	3.054	1.133	2.632	0.536	1.679	1.568	0.981	0.605	1.053	0.014	0.028	0.127	0.163	0.022	0.036	0.127	0.125	0.470	1.404
P54319	Phospholipase A-2-activating protein OS=Rattus norvegicus GN=Plaa PE=2 SV=3 - [PLAP_RAT]	87.0	0.834	1.202	0.559	1.103	0.885	1.079	0.542	0.990	1.049	0.755	1.297	1.574	1.895	2.351	0.901	2.160	0.480	1.573	1.478	0.916	0.605	0.953	0.036	0.056	0.259	0.275	0.056	0.065	0.354	0.276	0.470	1.465
P55260	Annexin A4 OS=Rattus norvegicus GN=Anxa4 PE=1 SV=3 - [ANXA4_RAT]	35.8	0.679	0.971	0.515	1.025	0.905	0.969	0.440	0.615	1.001	1.101	1.003	1.116	2.230	2.026	1.276	1.927	0.677	1.448	1.409	0.807	0.917	0.994	0.022	0.042	0.259	0.282	0.022	0.038	0.184	0.167	0.470	1.494
P57113	Maleylacetoacetate isomerase OS=Rattus norvegicus GN=Gstz1 PE=1 SV=2 - [MAAI_RAT]	23.9	0.402	1.045	0.296	1.134	0.853	0.967	0.513	0.505	0.915	0.606	1.050	1.068	2.088	3.876	1.216	3.221	0.594	1.652	1.586	1.085	0.917	1.052	0.036	0.055	0.085	0.120	0.014	0.026	0.056	0.076	0.917	0.963
P60711	Actin, cytoplasmic 1 OS=Rattus norvegicus GN=Actb PE=1 SV=1 - [ACTB_RAT]	41.7	0.318	1.089	0.330	0.969	0.807	0.618	0.201	0.368	1.061	0.732	1.116	1.117	2.007	3.560	0.981	3.609	0.531	1.676	1.479	0.901	0.605	1.130	0.022	0.041	0.127	0.161	0.022	0.039	0.085	0.097	0.756	1.150
P60825	Cold-inducible RNA-binding protein OS=Rattus norvegicus OX=10116 GN=Cirbp PE=2 SV=1 - [CIRBP_RAT]	18.6	0.013	0.013	0.013	0.013	0.013	0.013	0.013	0.013	0.013	0.013	1.663	1.611	3.606	2.534	2.041	0.013	0.013	0.013	0.013	0.013	#DIV/0!	#DIV/0!	0.009	0.029	#DIV/0!	#DIV/0!	0.009	0.023	#DIV/0!	#DIV/0!	0.009	0.064
P60892	Ribose-phosphate pyrophosphokinase 1 OS=Rattus norvegicus GN=Prps1 PE=1 SV=2 - [PRPS1_RAT]	34.8	0.448	1.045	0.427	1.017	0.860	1.102	0.218	0.440	0.900	0.897	1.496	1.437	2.302	3.315	1.007	3.154	0.608	2.083	1.379	0.963	0.917	1.046	0.036	0.055	0.127	0.160	0.022	0.037	0.085	0.094	0.354	1.406
P60901	Proteasome subunit alpha type-6 OS=Rattus norvegicus GN=Psma6 PE=1 SV=1 - [PSA6_RAT]	27.4	0.699	0.707	0.649	0.893	0.803	1.166	0.516	0.733	0.726	0.783	0.820	0.998	1.775	1.541	1.184	1.311	0.941	1.348	0.951	1.064	0.756	1.078	0.022	0.045	0.014	0.042	0.036	0.057	0.056	0.105	0.605	1.636
P60905	DnaJ homolog subfamily C member 5 OS=Rattus norvegicus OX=10116 GN=Dnajc5 PE=1 SV=1 - [DNJC5_RAT]	22.1	0.013	0.013	0.013	0.013	0.013	0.013	0.013	0.013	0.013	0.013	1.165	1.011	2.177	1.869	1.252	0.013	0.013	0.013	0.013	0.013	#DIV/0!	#DIV/0!	0.009	0.051	#DIV/0!	#DIV/0!	0.009	0.043	#DIV/0!	#DIV/0!	0.009	0.093
P61078	Ubiquitin-conjugating enzyme E2 D3 OS=Rattus norvegicus GN=Ube2d3 PE=2 SV=1 - [UB2D3_RAT]	16.7	0.658	0.957	0.549	1.037	0.966	1.146	0.548	0.745	0.893	0.940	1.259	1.211	2.384	1.418	1.268	1.401	0.874	1.568	1.880	1.406	0.756	0.993	0.014	0.034	0.056	0.096	0.014	0.029	0.056	0.077	0.756	1.255
P61203	COP9 signalosome complex subunit 2 OS=Rattus norvegicus GN=Cops2 PE=2 SV=1 - [CSN2_RAT]	51.6	0.714	1.015	0.597	1.060	0.855	1.353	0.363	0.645	0.939	0.881	1.199	1.272	2.153	2.296	1.419	1.941	0.870	1.606	1.417	1.057	0.917	0.980	0.014	0.030	0.056	0.099	0.036	0.051	0.085	0.098	0.354	1.442
P61206	ADP-ribosylation factor 3 OS=Rattus norvegicus GN=Arf3 PE=1 SV=2 - [ARF3_RAT]	20.6	0.013	0.013	0.013	0.013	0.013	0.013	0.013	0.013	0.013	0.013	2.140	1.707	4.424	6.705	2.021	0.013	0.013	0.013	0.013	0.013	#DIV/0!	#DIV/0!	0.009	0.027	#DIV/0!	#DIV/0!	0.009	0.022	#DIV/0!	#DIV/0!	0.009	0.061
P61621	Protein transport protein Sec61 subunit alpha isoform 1 OS=Rattus norvegicus OX=10116 GN=Sec61a1 PE=2 SV=2 - [S61A1_RAT]	52.2	0.013	0.013	0.013	0.013	0.013	0.013	0.013	0.013	0.013	0.013	1.224	1.106	2.583	1.445	1.389	0.013	0.013	0.013	0.013	0.013	#DIV/0!	#DIV/0!	0.009	0.049	#DIV/0!	#DIV/0!	0.009	0.040	#DIV/0!	#DIV/0!	0.009	0.088
P62138	Serine/threonine-protein phosphatase PP1-alpha catalytic subunit OS=Rattus norvegicus GN=Ppp1ca PE=1 SV=1 - [PP1A_RAT]	37.5	0.798	0.932	0.511	1.018	0.876	1.089	0.568	0.759	0.955	0.931	1.111	1.042	2.185	1.945	1.061	2.011	0.780	1.408	1.340	1.146	0.756	0.994	0.014	0.035	0.085	0.126	0.036	0.053	0.056	0.082	0.917	1.105
P62142	Serine/threonine-protein phosphatase PP1-beta catalytic subunit OS=Rattus norvegicus GN=Ppp1cb PE=1 SV=3 - [PP1B_RAT]	37.2	0.836	0.793	0.694	1.091	1.027	1.227	0.676	0.791	0.740	0.786	1.184	1.119	2.707	3.119	1.423	1.843	0.870	1.048	1.117	0.937	0.354	0.950	0.014	0.028	0.127	0.170	0.036	0.050	0.085	0.106	0.056	0.352
P62332	ADP-ribosylation factor 6 OS=Rattus norvegicus GN=Arf6 PE=1 SV=2 - [ARF6_RAT]	20.1	0.371	0.974	0.360	0.846	0.548	1.378	0.499	0.500	0.761	0.567	1.348	1.177	1.702	2.451	0.809	2.257	0.623	1.857	1.739	0.693	0.605	1.118	0.036	0.061	0.085	0.131	0.056	0.071	0.085	0.099	0.917	1.071
P62494	Ras-related protein Rab-11A OS=Rattus norvegicus GN=Rab11a PE=1 SV=3 - [RB11A_RAT]	24.4	0.013	0.013	0.013	0.013	0.013	0.013	0.013	0.013	0.013	0.013	1.760	1.894	2.920	2.222	2.133	0.013	0.013	0.013	0.013	0.013	#DIV/0!	#DIV/0!	0.009	0.029	#DIV/0!	#DIV/0!	0.009	0.024	#DIV/0!	#DIV/0!	0.009	0.065
P62744	AP-2 complex subunit sigma OS=Rattus norvegicus GN=Ap2s1 PE=1 SV=1 - [AP2S1_RAT]	17.0	0.013	0.013	0.013	0.013	0.013	0.013	0.013	0.013	0.013	0.013	1.043	0.766	1.668	2.100	0.966	0.013	0.013	0.013	0.013	0.013	#DIV/0!	#DIV/0!	0.009	0.064	#DIV/0!	#DIV/0!	0.009	0.056	#DIV/0!	#DIV/0!	0.009	0.124
P62747	Rho-related GTP-binding protein RhoB OS=Rattus norvegicus GN=Rhob PE=1 SV=1 - [RHOB_RAT]	22.1	0.013	1.682	0.013	0.972	0.644	0.417	0.115	0.191	1.373	1.409	3.051	2.081	4.641	6.570	1.289	3.698	0.013	2.586	2.820	1.867	0.755	1.141	0.022	0.044	0.082	0.128	0.036	0.049	0.127	0.122	0.354	1.387
P62755	40S ribosomal protein S6 OS=Rattus norvegicus OX=10116 GN=Rps6 PE=1 SV=1 - [RS6_RAT]	28.7	0.533	1.026	0.665	1.217	0.935	0.821	0.369	0.651	0.976	0.673	1.358	1.942	2.741	1.375	1.022	0.428	0.892	2.479	1.179	0.513	0.354	1.013	0.036	0.055	0.756	0.660	0.014	0.029	0.470	0.359	0.127	0.767
P62845	40S ribosomal protein S15 OS=Rattus norvegicus GN=Rps15 PE=1 SV=2 - [RS15_RAT]	17.0	0.013	0.013	0.013	0.013	0.013	0.696	0.193	0.516	0.871	0.927	1.462	2.259	3.751	1.666	1.095	0.013	0.013	0.013	0.013	0.013	0.009	1.078	0.009	0.031	#DIV/0!	#DIV/0!	0.014	0.025	0.009	1.471	0.009	0.068

P62963	Profilin-1 OS=Rattus norvegicus GN=Pfn1 PE=1 SV=2 - [PROF1_RAT]	14.9	0.613	0.973	0.533	1.016	0.997	1.052	0.399	0.694	0.973	1.045	0.999	1.007	2.172	1.491	1.147	1.483	0.850	1.319	1.240	1.283	0.756	1.013	0.036	0.060	0.056	0.117	0.085	0.092	0.056	0.094	0.917	1.187
P63018	Heat shock cognate 71 kDa protein OS=Rattus norvegicus GN=Hspa8 PE=1 SV=1 - [HSP7C_RAT]	70.8	0.652	0.921	0.524	1.006	0.930	0.997	0.433	0.633	0.920	0.990	1.089	1.155	2.340	2.402	1.108	2.140	0.839	1.397	1.272	1.157	0.756	1.047	0.014	0.032	0.056	0.103	0.014	0.028	0.056	0.085	0.917	1.059
P63095	Guanine nucleotide-binding protein G(s) subunit alpha isoforms short OS=Rattus norvegicus GN=Gnas PE=1 SV=1 - [GNAS2_RAT]	45.6	0.013	0.013	0.013	0.013	0.013	0.013	0.013	0.013	0.013	0.013	1.266	1.386	2.135	3.059	1.185	0.013	0.013	0.013	0.013	0.013	#DIV/0!	#DIV/0!	0.009	0.036	#DIV/0!	#DIV/0!	0.009	0.028	#DIV/0!	#DIV/0!	0.009	0.074
P63102	14-3-3 protein zeta/delta OS=Rattus norvegicus GN=Ywhaz PE=1 SV=1 - [1433Z_RAT]	27.8	0.669	0.926	0.527	0.966	0.965	0.943	0.455	0.677	0.901	0.927	1.069	1.012	2.106	2.273	1.034	2.103	0.807	1.263	1.211	1.228	0.605	1.052	0.014	0.035	0.056	0.109	0.014	0.030	0.056	0.089	0.917	1.103
P63140	Nuclear transcription factor Y subunit beta OS=Rattus norvegicus OX=10116 GN=Nfyb PE=1 SV=1 - [NFYB_RAT]	22.8	0.013	0.013	0.013	0.013	0.013	0.013	0.013	0.013	0.013	0.013	1.350	1.315	3.053	3.606	1.023	0.013	0.013	0.013	0.013	0.013	#DIV/0!	#DIV/0!	0.009	0.031	#DIV/0!	#DIV/0!	0.009	0.024	#DIV/0!	#DIV/0!	0.009	0.068
P63326	40S ribosomal protein S10 OS=Rattus norvegicus GN=Rps10 PE=2 SV=1 - [RS10_RAT]	18.9	0.914	0.796	0.705	1.315	1.016	0.682	0.248	0.425	0.985	1.034	1.950	1.945	4.018	0.968	1.387	1.048	0.806	1.745	1.231	0.918	0.259	0.925	0.036	0.054	0.259	0.280	0.036	0.050	0.085	0.110	0.085	0.533
P68035	Actin, alpha cardiac muscle 1 OS=Rattus norvegicus GN=Actc1 PE=2 SV=1 - [ACTC_RAT]	42.0	0.233	1.086	0.113	0.962	0.927	0.623	0.172	0.203	1.070	0.728	1.205	1.246	2.170	5.010	1.028	4.302	0.563	1.639	1.520	0.857	0.605	1.133	0.022	0.039	0.184	0.206	0.022	0.037	0.085	0.094	0.605	1.295
P68182	cAMP-dependent protein kinase catalytic subunit beta OS=Rattus norvegicus GN=Prkacb PE=1 SV=2 - [KAPCB_RAT]	40.7	0.013	0.013	0.013	0.013	0.013	1.051	0.139	0.417	1.224	1.311	1.683	1.590	2.750	3.689	1.138	1.558	0.826	1.507	0.879	1.116	0.009	0.154	0.009	0.030	0.009	0.068	0.036	0.049	0.354	0.280	0.036	0.229
P69060	N-acylneuraminate cytidylyltransferase OS=Rattus norvegicus GN=Cmas PE=2 SV=1 - [NEUA_RAT]	48.1	0.659	0.931	0.426	1.000	0.939	1.635	0.904	0.850	0.624	0.643	1.577	1.545	2.403	1.810	0.971	1.576	1.159	1.419	1.177	0.672	0.756	0.967	0.022	0.041	0.056	0.123	0.056	0.065	0.259	0.214	0.127	0.757
P70473	Alpha-methylacyl-CoA racemase OS=Rattus norvegicus OX=10116 GN=Amacr PE=1 SV=3 - [AMACR_RAT]	41.8	0.013	0.013	0.013	0.013	0.013	0.520	0.331	0.496	0.449	0.512	0.653	0.642	0.966	8.437	0.566	8.051	0.304	0.852	0.855	0.715	0.009	2.695	0.009	0.029	0.009	0.029	0.014	0.025	0.127	0.122	0.917	0.920
P70541	Translation initiation factor eIF-2B subunit gamma OS=Rattus norvegicus GN=Eif2b3 PE=2 SV=2 - [EI2BG_RAT]	50.4	0.013	0.013	0.013	0.013	0.013	1.795	0.763	0.930	1.050	0.928	1.157	1.140	1.788	0.862	0.852	0.688	1.016	1.325	1.287	0.453	0.009	0.054	0.009	0.080	0.009	0.137	0.756	0.547	0.756	0.544	0.605	1.733
P70619	Glutathione reductase (Fragment) OS=Rattus norvegicus GN=Gsr PE=2 SV=2 - [GSHR_RAT]	46.3	0.477	0.777	0.447	0.841	0.831	0.822	0.315	0.505	0.902	0.945	0.929	0.991	1.772	3.283	0.928	3.032	0.633	1.168	1.092	1.091	0.605	1.113	0.014	0.036	0.056	0.116	0.036	0.054	0.056	0.090	0.917	1.057
P70645	Bleomycin hydrolase OS=Rattus norvegicus GN=Blmh PE=1 SV=1 - [BLMH_RAT]	52.3	0.499	1.044	0.397	0.968	0.978	0.882	0.358	0.538	1.061	1.068	1.049	1.147	2.199	1.690	1.257	1.604	0.729	1.606	1.233	1.265	0.756	1.071	0.014	0.036	0.056	0.117	0.036	0.054	0.056	0.094	0.756	1.322
P70712	Kynureninase OS=Rattus norvegicus OX=10116 GN=Kynu PE=1 SV=2 - [KYNU_RAT]	52.4	0.013	0.013	0.013	0.013	0.013	0.013	0.013	0.013	0.013	0.013	0.767	0.850	1.678	3.527	0.827	0.013	0.013	0.013	0.013	0.013	#DIV/0!	#DIV/0!	0.009	0.050	#DIV/0!	#DIV/0!	0.009	0.041	#DIV/0!	#DIV/0!	0.009	0.090
P80067	Dipeptidyl peptidase 1 OS=Rattus norvegicus OX=10116 GN=Ctsc PE=1 SV=3 - [CATC_RAT]	52.2	0.440	1.154	0.290	1.130	1.012	0.650	0.222	0.309	1.275	1.284	1.590	1.578	3.148	1.932	1.371	1.770	0.507	1.808	1.907	1.388	0.917	1.026	0.014	0.028	0.056	0.095	0.014	0.026	0.056	0.080	0.605	1.318
P81799	N-acetyl-D-glucosamine kinase OS=Rattus norvegicus GN=Nagk PE=1 SV=4 - [NAGK_RAT]	37.2	0.352	1.211	0.302	0.906	1.005	0.676	0.208	0.254	1.036	1.061	1.292	1.385	2.588	3.027	1.267	2.834	0.442	1.894	1.620	1.495	0.605	1.109	0.014	0.029	0.056	0.091	0.014	0.026	0.056	0.076	0.917	0.954
P83868	Prostaglandin E synthase 3 OS=Rattus norvegicus GN=Ptges3 PE=1 SV=2 - [TEBP_RAT]	18.7	0.494	0.975	0.452	1.088	0.886	0.856	0.319	0.650	1.089	1.176	1.253	1.053	2.349	1.938	1.230	1.369	0.593	1.363	1.261	1.024	0.756	1.053	0.022	0.042	0.085	0.133	0.036	0.052	0.184	0.173	0.470	1.555
P83953	Importin subunit alpha-5 OS=Rattus norvegicus GN=Kpna1 PE=1 SV=1 - [IMA5_RAT]	60.1	0.013	0.013	0.013	0.013	0.013	0.013	0.013	0.013	0.013	0.013	1.410	1.237	2.304	1.933	1.356	0.013	0.013	0.013	0.013	0.013	#DIV/0!	#DIV/0!	0.009	0.043	#DIV/0!	#DIV/0!	0.009	0.034	#DIV/0!	#DIV/0!	0.009	0.081
P84245	Histone H3.3 OS=Rattus norvegicus GN=H3f3b PE=1 SV=2 - [H33_RAT]	15.3	0.013	0.013	0.013	0.013	0.013	0.013	0.013	0.013	0.013	0.013	1.450	2.033	3.107	3.203	1.830	0.013	0.013	0.013	0.013	0.013	#DIV/0!	#DIV/0!	0.009	0.028	#DIV/0!	#DIV/0!	0.009	0.023	#DIV/0!	#DIV/0!	0.009	0.064
P85515	Alpha-centractin OS=Rattus norvegicus GN=Actr1a PE=1 SV=1 - [ACTZ_RAT]	42.6	0.715	1.143	0.671	1.313	1.123	1.173	0.546	0.750	0.845	1.025	1.461	1.631	2.682	3.072	1.364	2.689	0.684	2.329	1.609	1.229	0.605	0.942	0.014	0.028	0.127	0.157	0.014	0.025	0.085	0.092	0.470	1.387
P85968	6-phosphogluconate dehydrogenase, decarboxylating OS=Rattus norvegicus GN=Pgd PE=1 SV=1 - [6PGD_RAT]	53.2	0.648	0.800	0.551	0.873	0.891	1.045	0.455	0.689	0.942	0.865	0.967	0.953	2.086	2.159	1.055	1.823	0.854	1.217	1.085	1.146	0.605	1.075	0.014	0.038	0.036	0.090	0.036	0.054	0.056	0.097	0.917	1.157
P85971	6-phosphogluconolactonase OS=Rattus norvegicus GN=Pgls PE=1 SV=1 - [6PGL_RAT]	27.2	0.646	1.142	0.571	1.083	0.981	0.970	0.501	0.653	1.172	1.213	1.362	1.417	2.681	1.780	1.403	1.828	0.681	2.035	1.712	1.686	0.756	0.945	0.014	0.029	0.056	0.090	0.014	0.026	0.056	0.074	0.756	1.161
P97536	Cullin-associated NEDD8-dissociated protein 1 OS=Rattus norvegicus GN=Cand1 PE=1 SV=1 - [CAND1_RAT]	136.3	0.935	0.964	0.521	0.996	0.923	1.641	0.619	0.677	0.930	0.794	1.261	1.452	1.698	2.097	0.964	2.043	0.740	1.750	1.470	0.743	0.605	0.955	0.022	0.042	0.354	0.349	0.056	0.066	0.259	0.212	0.756	1.289
P97675	Ectonucleotide pyrophosphatase/phosphodiesterase family member 3 OS=Rattus norvegicus GN=Enpp3 PE=1 SV=2 - [ENPP3_RAT]	99.0	0.596	0.986	0.415	0.694	0.675	1.296	0.429	0.369	1.127	0.899	1.328	1.125	2.308	1.879	1.125	1.795	0.918	2.126	1.582	0.726	0.605	1.090	0.014	0.037	0.036	0.086	0.085	0.088	0.127	0.125	0.605	1.403
P98158	Low-density lipoprotein receptor-related protein 2 OS=Rattus norvegicus OX=10116 GN=Lrp2 PE=1 SV=1 - [LRP2_RAT]	518.9	0.244	0.611	0.148	0.568	0.478	0.507	0.147	0.225	0.882	0.805	0.633	0.697	1.087	6.398	0.643	5.625	0.490	0.902	0.867	0.708	0.756	1.134	0.014	0.035	0.036	0.085	0.184	0.155	0.184	0.162	0.917	0.951
Q00438	Polypyrimidine tract-binding protein 1 OS=Rattus norvegicus GN=Ptbp1 PE=1 SV=1 - [PTBP1_RAT]	59.3	1.072	1.001	0.655	1.232	1.029	0.875	0.385	0.492	1.002	1.018	1.594	1.524	3.002	2.185	1.147	2.125	0.699	1.794	1.717	1.135	0.127	0.591	0.022	0.039	0.127	0.158	0.014	0.026	0.056	0.079	0.470	1.407
Q00729	Histone H2B type 1-A OS=Rattus norvegicus GN=Hist1h2ba PE=1 SV=2 - [H2B1A_RAT]	14.2	1.793	0.608	0.821	1.148	1.037	1.305	0.992	0.743	0.669	0.626	0.013	0.013	0.013	0.013	0.013	1.330	0.889	1.221	1.349	0.550	0.470	0.909	0.009	0.098	0.756	0.653	0.009	0.228	0.354	0.281	0.009	0.239
Q01205	Dihydrolipoyllysine-residue succinyltransferase component of 2-oxoglutarate dehydrogenase complex, mitochondrial OS=Rattus norvegicus GN=Dlst PE=1 SV=2 - [ODO2_RAT]	48.9	0.525	0.890	0.495	0.873	0.749	0.846	0.323	0.560	0.937	0.888	0.920	1.103	1.984	3.632	1.064	2.637	0.773	1.238	1.178	1.129	0.756	1.104	0.014	0.031	0.036	0.086	0.022	0.038	0.056	0.090	0.917	1.023
Q01986	Dual specificity mitogen-activated protein kinase kinase 1 OS=Rattus norvegicus GN=Map2k1 PE=1 SV=2 - [MP2K1_RAT]	43.4	0.644	0.939	0.490	1.050	0.917	1.235	0.487	0.812	0.879	0.764	1.582	1.551	2.557	2.828	1.311	2.240	0.538	1.939	1.408	0.965	0.756	1.020	0.014	0.028	0.127	0.164	0.014	0.025	0.127	0.125	0.259	1.268
Q02974	Ketohexokinase OS=Rattus norvegicus OX=10116 GN=Khk PE=1 SV=1 - [KHK_RAT]	32.7	0.390	0.459	0.381	0.495	0.531	0.668	0.358	0.509	0.478	0.502	0.699	0.727	1.036	5.720	0.607	4.977	0.453	0.901	0.811	0.803	0.470	1.140	0.014	0.037	0.056	0.120	0.022	0.039	0.085	0.103	0.917	0.985
Q03346	Mitochondrial-processing peptidase subunit beta OS=Rattus norvegicus OX=10116 GN=Pmpcb PE=1 SV=3 - [MPPB_RAT]	54.2	0.899	1.174	0.616	1.230	1.100	0.993	0.743	0.650	1.045	1.290	1.277	1.195	2.447	3.626	1.490	2.244	0.881	1.325	1.556	1.181	0.756	0.906	0.022	0.039	0.127	0.159	0.036	0.049	0.085	0.093	0.354	1.414
Q04462	Valine--tRNA ligase OS=Rattus norvegicus GN=Vars PE=2 SV=2 - [SYVC_RAT]	140.3	0.751	1.025	0.607	1.068	0.951	1.441	0.551	0.742	0.859	0.852	1.242	1.389	2.154	1.229	1.177	1.075	1.001	1.723	1.363	0.922	0.605	0.967	0.014	0.034	0.127	0.168	0.085	0.089	0.085	0.101	0.259	1.308
Q04631	Protein farnesyltransferase/geranylgeranyltransferase type-1 subunit alpha OS=Rattus norvegicus GN=Fnta PE=1 SV=1 - [FNTA_RAT]	44.0	0.688	1.182	0.452	1.119	1.028	1.271	0.553	0.583	0.978	1.169	1.250	1.258	2.188	1.428	1.339	1.631	0.744	1.737	1.660	1.589	0.917	0.941	0.014	0.032	0.056	0.091	0.036	0.051	0.056	0.075	0.470	1.455
Q06496	Sodium-dependent phosphate transport protein 2A OS=Rattus norvegicus OX=10116 GN=Slc34a1 PE=1 SV=1 - [NPT2A_RAT]	68.7	0.013	0.013	0.013	0.013	0.013	0.013	0.013	0.013	0.013	0.013	2.170	1.312	3.132	4.289	1.008	0.013	0.013	0.013	0.013	0.013	#DIV/0!	#DIV/0!	0.009	0.028	#DIV/0!	#DIV/0!	0.009	0.022	#DIV/0!	#DIV/0!	0.009	0.063
Q07936	Annexin A2 OS=Rattus norvegicus GN=Anxa2 PE=1 SV=2 - [ANXA2_RAT]	38.7	0.629	0.888	0.501	0.832	0.784	0.822	0.431	0.608	0.679	0.830	0.980	1.095	2.049	2.300	1.266	2.263	0.698	1.493	1.330	0.798	0.470	1.115	0.014	0.036	0.127	0.170	0.014	0.031	0.085	0.106	0.605	1.430
Q08163	Adenylyl cyclase-associated protein 1 OS=Rattus norvegicus GN=Cap1 PE=1 SV=3 - [CAP1_RAT]	51.6	0.651	0.929	0.592	0.808	0.803	0.968	0.460	0.714	0.880	0.901	0.972	0.986	2.131	1.868	1.117	1.777	0.877	1.266	1.141	1.177	0.605	1.080	0.014	0.038	0.022	0.061	0.014	0.031	0.056	0.097	0.917	1.159
Q09030	Trefoil factor 2 OS=Rattus norvegicus OX=10116 GN=Tff2 PE=2 SV=1 - [TFF2_RAT]	14.1	1.344	0.993	0.955	1.323	0.928	0.013	0.013	0.013	0.013	0.013	0.013	0.013	0.013	0.013	0.013	0.013	0.013	0.013	0.013	0.013	0.009	0.054	0.009	0.090	0.009	0.077	#DIV/0!	#DIV/0!	#DIV/0!	#DIV/0!	#DIV/0!	#DIV/0!
Q0ZHH6	Atlastin-3 OS=Rattus norvegicus OX=10116 GN=Atl3 PE=2 SV=2 - [ATLA3_RAT]	60.5	0.013	0.013	0.013	0.013	0.013	0.013	0.013	0.013	0.013	0.013	4.141	3.650	5.710	1.802	2.188	0.013	0.013	0.013	0.013	0.013	#DIV/0!	#DIV/0!	0.009	0.027	#DIV/0!	#DIV/0!	0.009	0.022	#DIV/0!	#DIV/0!	0.009	0.060
Q1JU68	Eukaryotic translation initiation factor 3 subunit A OS=Rattus norvegicus GN=Eif3a PE=2 SV=2 - [EIF3A_RAT]	163.1	0.757	1.048	0.566	1.135	0.937	1.759	0.471	0.673	0.576	0.678	1.364	1.450	2.427	1.553	0.993	1.483	0.885	2.096	1.329	0.780	0.354	0.959	0.036	0.056	0.184	0.214	0.085	0.088	0.085	0.0997	0.354	1.467
Q2A121	Alpha-ketoglutarate-dependent dioxygenase FTO OS=Rattus norvegicus GN=Fto PE=2 SV=1 - [FTO_RAT]	57.9	0.013	0.013	0.013	0.013	0.013	1.226	0.650	0.771	1.138	1.041	0.934	1.370	2.030	1.146	5.084	0.013	0.013	0.013	0.013	0.013	0.009	0.067	0.009	0.030	#DIV/0!	#DIV/0!	0.085	0.085	0.009	0.103	0.009	0.067
Q32Q06	AP-1 complex subunit mu-1 OS=Rattus norvegicus GN=Ap1m1 PE=1 SV=3 - [AP1M1_RAT]	48.5	0.013	0.013	0.013	0.013	0.013	0.013	0.013	0.013	0.013	0.013	1.046	1.160	2.442	2.160	1.091	0.013	0.013	0.013	0.013	0.013	#DIV/0!	#DIV/0!	0.009	0.047	#DIV/0!	#DIV/0!	0.009	0.038	#DIV/0!	#DIV/0!	0.009	0.086
Q3B7D1	Ubiquitin-conjugating enzyme E2 Z OS=Rattus norvegicus GN=Ube2z PE=2 SV=2 - [UBE2Z_RAT]	38.3	0.013	0.013	0.013	0.013	0.013	1.537	0.707	0.829	0.742	0.706	0.679	0.796	1.868	0.913	1.323	1.356	0.659	1.300	1.228	1.554	0.009	0.086	0.009	0.086	0.009	0.064	0.470	0.357	0.354	0.277	0.756	1.482
Q3B8Q2	Eukaryotic initiation factor 4A-III OS=Rattus norvegicus GN=Eif4a3 PE=1 SV=1 - [IF4A3_RAT]	46.8	0.013	0.013	0.013	0.013	0.013	0.013	0.013	0.013	0.013	0.013	1.396	1.215	2.139	2.198	1.095	0.013	0.013	0.013	0.013	0.013	#DIV/0!	#DIV/0!	0.009	0.045	#DIV/0!	#DIV/0!	0.009	0.036	#DIV/0!	#DIV/0!	0.009	0.084
Q3MIE0	Enoyl-CoA hydratase domain-containing protein 3, mitochondrial OS=Rattus norvegicus OX=10116 GN=Echdc3 PE=2 SV=1 - [ECHD3_RAT]	32.4	0.740	1.058	0.564	0.910	0.869	1.211	0.479	0.625	0.962	1.009	1.045	1.321	2.094	1.879	1.216	1.923	0.776	1.597	1.366	1.567	0.756	0.997	0.022	0.042	0.056	0.095	0.022	0.038	0.056	0.076	0.917	1.066
Q3T1K5	F-actin-capping protein subunit alpha-2 OS=Rattus norvegicus GN=Capza2 PE=1 SV=1 - [CAZA2_RAT]	32.9	0.565	1.077	0.459	0.942	0.875	0.935	0.402	0.589	1.014	1.151	1.059	1.140	2.113	2.390	1.190	2.166	0.680	1.411	1.249	1.374	0.756	1.044	0.022	0.042	0.056	0.106	0.036	0.051	0.056	0.083	0.917	1.068
Q3T1L0	Aldehyde dehydrogenase family 16 member A1 OS=Rattus norvegicus GN=Aldh16a1 PE=2 SV=1 - [A16A1_RAT]	85.4	0.013	0.013	0.013	0.013	0.013	0.013	0.013	0.013	0.013	0.013	0.742	0.931	1.580	1.793	0.871	1.393	0.798	0.887	1.009	0.599	#DIV/0!	#DIV/0!	0.009	0.077	0.009	0.142	0.009	0.067	0.009	0.116	0.470	1.778
Q497B0	Omega-amidase NIT2 OS=Rattus norvegicus GN=Nit2 PE=1 SV=1 - [NIT2_RAT]	30.7	0.511	0.792	0.455	0.794	0.757	0.802	0.373	0.556	0.861	0.940	0.904	0.988	1.877	3.942	0.848	4.466	0.512	1.362	1.275	1.143	0.354	1.072	0.014	0.033	0.056	0.091	0.056	0.066	0.085	0.092	0.756	1.142
Q4AEF8	Coatomer subunit gamma-1 OS=Rattus norvegicus GN=Copg1 PE=2 SV=1 - [COPG1_RAT]	97.6	0.853	1.062	0.609	1.103	0.950	1.434	0.461	0.428	0.873	0.853	1.724	1.948	2.742	1.651	1.037	1.962	0.506	2.847	1.846	0.928	0.470	1.007	0.036	0.054	0.354	0.342	0.022	0.037	0.085	0.093	0.917	0.970
Q4FZT0	Stomatin-like protein 2, mitochondrial OS=Rattus norvegicus OX=10116 GN=Stoml2 PE=1 SV=1 - [STML2_RAT]	38.4	0.013	0.013	0.013	0.013	0.013	0.013	0.013	0.013	0.013	0.013	0.972	0.977	2.541	3.338	1.194	0.013	0.013	0.013	0.013	0.013	#DIV/0!	#DIV/0!	0.009	0.036	#DIV/0!	#DIV/0!	0.009	0.029	#DIV/0!	#DIV/0!	0.009	0.074
Q4FZT2	Protein phosphatase methylesterase 1 OS=Rattus norvegicus GN=Ppme1 PE=1 SV=2 - [PPME1_RAT]	42.3	0.013	0.013	0.013	0.013	0.013	0.013	0.013	0.013	0.013	0.013	1.148	1.024	1.527	2.608	0.832	0.013	0.013	0.013	0.013	0.013	#DIV/0!	#DIV/0!	0.009	0.056	#DIV/0!	#DIV/0!	0.009	0.047	#DIV/0!	#DIV/0!	0.009	0.099
Q4FZX7	Signal recognition particle receptor subunit beta OS=Rattus norvegicus OX=10116 GN=Srprb PE=2 SV=1 - [SRPRB_RAT]	29.6	1.043	0.674	0.761	0.886	0.777	1.602	0.765	0.705	0.845	0.627	1.076	0.930	1.476	1.434	0.968	1.391	0.918	1.514	1.463	0.914	0.756	0.960	0.036	0.061	0.036	0.087	0.127	0.123	0.127	0.128	0.917	1.236
Q4KLN8	E3 ubiquitin-protein ligase RNF8 OS=Rattus norvegicus OX=10116 GN=Rnf8 PE=2 SV=1 - [RNF8_RAT]	55.6	1.009	1.245	1.049	1.686	1.153	0.013	0.013	0.013	0.013	0.013	0.013	0.013	0.013	0.013	0.013	0.013	0.013	0.013	0.013	0.013	0.009	0.052	0.009	0.071	0.009	0.063	#DIV/0!	#DIV/0!	#DIV/0!	#DIV/0!	#DIV/0!	#DIV/0!
Q4KM73	UMP-CMP kinase OS=Rattus norvegicus GN=Cmpk1 PE=1 SV=2 - [KCY_RAT]	22.2	0.816	1.014	0.638	1.192	0.958	1.144	0.563	0.690	0.919	0.944	1.127	1.212	2.260	1.699	1.109	1.419	0.892	1.549	1.425	1.201	0.470	0.971	0.036	0.057	0.056	0.0996	0.036	0.053	0.056	0.086	0.756	1.310
Q4KM74	Vesicle-trafficking protein SEC22b OS=Rattus norvegicus GN=Sec22b PE=1 SV=3 - [SC22B_RAT]	24.7	0.912	1.014	0.723	1.204	0.833	1.224	0.713	0.724	0.676	0.672	1.688	1.659	2.968	1.968	1.217	1.499	0.800	1.813	1.330	0.451	0.184	0.747	0.014	0.028	0.470	0.442	0.022	0.037	0.184	0.172	0.127	0.751
Q4QQT4	Serine/threonine-protein phosphatase 2A 65 kDa regulatory subunit A beta isoform OS=Rattus norvegicus GN=Ppp2r1b PE=2 SV=1 - [2AAB_RAT]	66.0	0.513	1.258	0.328	1.023	1.006	1.046	0.381	0.378	1.293	1.196	1.451	1.387	2.313	2.894	1.243	2.328	0.614	1.639	1.781	1.241	0.605	1.007	0.022	0.040	0.085	0.120	0.022	0.036	0.085	0.093	0.605	1.326
Q4QQW8	Putative phospholipase B-like 2 OS=Rattus norvegicus GN=Plbd2 PE=1 SV=2 - [PLBL2_RAT]	65.4	0.013	0.013	0.013	0.013	0.013	0.013	0.013	0.013	0.013	0.013	0.458	0.557	1.670	2.513	0.895	2.070	0.228	0.799	0.932	0.557	#DIV/0!	#DIV/0!	0.009	0.073	0.009	0.165	0.009	0.064	0.009	0.128	0.756	1.558
Q4V7B0	Cilia- and flagella-associated protein 157 OS=Rattus norvegicus OX=10116 GN=Cfap157 PE=1 SV=2 - [CF157_RAT]	62.8	0.013	0.013	0.013	0.013	0.013	0.013	0.013	0.013	0.013	0.013	0.629	1.670	1.318	0.905	1.084	0.013	0.013	0.013	0.013	0.013	#DIV/0!	#DIV/0!	0.009	0.085	#DIV/0!	#DIV/0!	0.009	0.076	#DIV/0!	#DIV/0!	0.009	0.190
Q4V7C6	GMP synthase [glutamine-hydrolyzing] OS=Rattus norvegicus GN=Gmps PE=1 SV=1 - [GUAA_RAT]	76.7	0.672	0.972	0.543	1.035	1.017	0.951	0.432	0.584	1.020	1.017	1.137	1.040	2.151	2.041	1.166	1.776	0.793	1.276	1.212	1.022	0.605	1.025	0.014	0.033	0.085	0.130	0.014	0.030	0.056	0.099	0.470	1.534
Q4V7C7	Actin-related protein 3 OS=Rattus norvegicus GN=Actr3 PE=1 SV=1 - [ARP3_RAT]	47.3	0.622	0.906	0.565	0.896	0.779	1.070	0.470	0.704	0.875	0.962	0.896	1.040	2.008	1.915	1.215	1.713	0.752	1.403	1.174	1.268	0.605	1.063	0.036	0.060	0.056	0.121	0.056	0.071	0.056	0.093	0.756	1.349
Q4V8F9	Hydroxysteroid dehydrogenase-like protein 2 OS=Rattus norvegicus OX=10116 GN=Hsdl2 PE=2 SV=1 - [HSDL2_RAT]	58.3	0.246	0.969	0.195	0.790	0.682	0.627	0.456	0.505	0.923	1.000	0.904	1.143	1.950	4.418	0.833	4.059	0.483	1.261	1.372	0.968	0.605	1.132	0.036	0.057	0.085	0.124	0.085	0.086	0.127	0.124	0.917	0.966
Q505J8	Phenylalanine--tRNA ligase alpha subunit OS=Rattus norvegicus GN=Farsa PE=1 SV=1 - [SYFA_RAT]	57.7	1.000	1.046	0.577	1.168	1.065	1.071	0.381	0.460	0.966	0.935	1.706	1.515	2.365	1.555	1.102	1.682	0.743	2.527	2.318	0.920	0.184	0.752	0.022	0.040	0.354	0.342	0.014	0.028	0.184	0.160	0.917	0.999
Q562C9	1,2-dihydroxy-3-keto-5-methylthiopentene dioxygenase OS=Rattus norvegicus OX=10116 GN=Adi1 PE=2 SV=1 - [MTND_RAT]	21.4	0.013	0.013	0.013	0.013	0.013	1.065	0.522	0.535	0.895	0.916	1.413	1.039	2.183	1.775	0.956	1.288	0.662	1.416	1.266	0.890	0.009	0.234	0.009	0.052	0.009	0.078	0.036	0.054	0.184	0.174	0.259	1.327
Q566Q8	UPF0696 protein C11orf68 homolog OS=Rattus norvegicus OX=10116 GN=Bles03 PE=1 SV=2 - [CK068_RAT]	31.4	0.583	1.048	0.552	1.144	1.036	0.962	0.382	0.725	0.762	0.815	1.176	1.096	2.106	2.544	1.094	2.148	0.635	1.498	1.198	0.951	0.354	1.009	0.036	0.056	0.184	0.216	0.014	0.029	0.127	0.132	0.605	1.426
Q568Z6	IST1 homolog OS=Rattus norvegicus GN=Ist1 PE=2 SV=1 - [IST1_RAT]	39.9	0.688	1.209	0.562	1.187	1.046	1.314	0.588	0.520	1.275	1.239	1.371	1.418	2.198	1.630	1.089	1.536	0.501	1.826	1.591	0.991	0.470	0.913	0.036	0.056	0.354	0.348	0.056	0.065	0.354	0.274	0.605	1.442
Q5BJQ0	Atypical kinase COQ8A, mitochondrial OS=Rattus norvegicus OX=10116 GN=Coq8a PE=2 SV=1 - [COQ8A_RAT]	72.2	0.013	0.013	0.013	0.013	0.013	0.013	0.013	0.013	0.013	0.013	1.289	1.234	2.789	2.435	1.151	0.013	0.013	0.013	0.013	0.013	#DIV/0!	#DIV/0!	0.009	0.038	#DIV/0!	#DIV/0!	0.009	0.030	#DIV/0!	#DIV/0!	0.009	0.075
Q5BJZ6	Carnosine N-methyltransferase OS=Rattus norvegicus OX=10116 GN=Carnmt1 PE=1 SV=1 - [CARME_RAT]	46.4	0.013	0.013	0.013	0.013	0.013	0.013	0.013	0.013	0.013	0.013	1.069	1.170	2.269	2.196	0.955	0.013	0.013	0.013	0.013	0.013	#DIV/0!	#DIV/0!	0.009	0.049	#DIV/0!	#DIV/0!	0.009	0.041	#DIV/0!	#DIV/0!	0.009	0.089
Q5BK81	Prostaglandin reductase 2 OS=Rattus norvegicus GN=Ptgr2 PE=2 SV=2 - [PTGR2_RAT]	38.1	0.265	1.061	0.152	0.835	0.749	0.571	0.387	0.336	1.013	1.193	1.134	1.159	2.200	4.891	1.204	4.905	0.466	1.655	1.855	1.541	0.605	1.125	0.014	0.028	0.056	0.088	0.036	0.050	0.056	0.073	0.756	1.103
Q5I0C3	Methylcrotonoyl-CoA carboxylase subunit alpha, mitochondrial OS=Rattus norvegicus OX=10116 GN=Mccc1 PE=1 SV=1 - [MCCA_RAT]	79.3	0.775	0.928	0.496	1.049	0.958	1.246	0.334	0.478	0.726	0.796	1.099	1.167	1.936	2.614	0.975	2.400	0.686	2.260	1.481	0.900	0.354	1.018	0.022	0.041	0.259	0.271	0.056	0.070	0.085	0.096	0.756	1.209
Q5I0E7	Transmembrane emp24 domain-containing protein 9 OS=Rattus norvegicus OX=10116 GN=Tmed9 PE=1 SV=1 - [TMED9_RAT]	27.0	1.600	0.730	0.769	1.380	0.967	1.190	0.837	0.766	1.054	0.563	0.013	0.013	0.013	0.013	0.013	0.013	0.013	0.013	0.013	0.013	0.470	0.904	0.009	0.095	0.009	0.082	0.009	0.194	0.009	0.158	#DIV/0!	#DIV/0!
Q5I0G4	Glycine--tRNA ligase OS=Rattus norvegicus OX=10116 GN=Gars1 PE=1 SV=2 - [GARS_RAT]	81.7	0.903	1.014	0.610	1.138	0.963	1.349	0.619	0.815	0.898	0.822	1.186	1.389	2.203	1.100	1.071	1.113	1.049	1.552	1.376	0.769	0.470	0.948	0.036	0.058	0.184	0.220	0.056	0.069	0.184	0.167	0.470	1.595
Q5I0L4	Cytosolic Fe-S cluster assembly factor NUBP1 OS=Rattus norvegicus GN=Nubp1 PE=1 SV=1 - [NUBP1_RAT]	34.0	0.965	0.867	0.729	0.936	1.097	1.519	0.816	0.958	0.905	0.832	1.152	0.995	2.117	1.404	1.082	1.828	0.690	1.243	1.304	0.931	0.917	0.925	0.036	0.059	0.354	0.355	0.085	0.088	0.470	0.351	0.605	1.544
Q5I0P2	Glycine cleavage system H protein, mitochondrial OS=Rattus norvegicus OX=10116 GN=Gcsh PE=2 SV=1 - [GCSH_RAT]	18.5	0.735	0.926	0.477	1.094	0.848	0.953	0.478	0.594	0.900	0.884	1.473	1.133	2.669	4.067	1.025	2.719	0.706	1.270	1.297	0.981	0.917	1.016	0.022	0.039	0.127	0.165	0.014	0.025	0.056	0.085	0.354	1.410
Q5M827	Pirin OS=Rattus norvegicus GN=Pir PE=1 SV=1 - [PIR_RAT]	32.2	0.013	0.013	0.013	0.013	0.013	0.013	0.013	0.013	0.013	0.013	0.942	1.252	2.578	4.943	1.344	0.013	0.013	0.013	0.013	0.013	#DIV/0!	#DIV/0!	0.009	0.029	#DIV/0!	#DIV/0!	0.009	0.023	#DIV/0!	#DIV/0!	0.009	0.065
Q5M886	Manganese-dependent ADP-ribose/CDP-alcohol diphosphatase OS=Rattus norvegicus OX=10116 GN=Adprm PE=1 SV=1 - [ADPRM_RAT]	38.7	0.013	0.013	0.013	0.013	0.013	0.013	0.013	0.013	0.013	0.013	1.169	1.261	2.718	1.953	1.578	0.013	0.013	0.013	0.013	0.013	#DIV/0!	#DIV/0!	0.009	0.040	#DIV/0!	#DIV/0!	0.009	0.031	#DIV/0!	#DIV/0!	0.009	0.077
Q5PPH0	Enolase-phosphatase E1 OS=Rattus norvegicus GN=Enoph1 PE=2 SV=1 - [ENOPH_RAT]	28.9	0.013	0.013	0.013	0.013	0.013	0.013	0.013	0.013	0.013	0.013	1.365	1.250	3.311	2.118	2.597	0.013	0.013	0.013	0.013	0.013	#DIV/0!	#DIV/0!	0.009	0.030	#DIV/0!	#DIV/0!	0.009	0.024	#DIV/0!	#DIV/0!	0.009	0.066
Q5PPJ4	Deoxyhypusine hydroxylase OS=Rattus norvegicus OX=10116 GN=Dohh PE=2 SV=1 - [DOHH_RAT]	33.1	0.013	0.013	0.013	0.013	0.013	1.216	0.503	0.855	0.657	0.761	0.693	0.699	1.651	1.804	0.759	0.013	0.013	0.013	0.013	0.013	0.009	0.207	0.009	0.084	#DIV/0!	#DIV/0!	0.470	0.360	0.009	0.368	0.009	0.187
Q5PPJ9	Endophilin-B2 OS=Rattus norvegicus GN=Sh3glb2 PE=2 SV=2 - [SHLB2_RAT]	44.8	0.013	0.013	0.013	0.013	0.013	0.013	0.013	0.013	0.013	0.013	1.068	1.170	2.274	1.680	1.113	0.013	0.013	0.013	0.013	0.013	#DIV/0!	#DIV/0!	0.009	0.053	#DIV/0!	#DIV/0!	0.009	0.045	#DIV/0!	#DIV/0!	0.009	0.096
Q5RJQ4	NAD-dependent protein deacetylase sirtuin-2 OS=Rattus norvegicus GN=Sirt2 PE=1 SV=1 - [SIR2_RAT]	39.3	0.966	0.695	0.677	0.992	0.642	1.491	0.698	0.890	0.729	0.833	1.318	1.093	2.292	1.278	0.794	0.013	0.013	0.013	0.013	0.013	0.354	0.957	0.036	0.061	0.009	0.603	0.184	0.157	0.009	0.119	0.009	0.112
Q5RK30	Ribosome maturation protein SBDS OS=Rattus norvegicus GN=Sbds PE=2 SV=1 - [SBDS_RAT]	28.7	0.013	0.013	0.013	0.013	0.013	0.013	0.013	0.013	0.013	0.013	1.221	0.881	2.002	3.173	0.757	1.123	1.310	0.673	0.635	0.484	#DIV/0!	#DIV/0!	0.009	0.045	0.009	0.342	0.009	0.037	0.009	0.226	0.127	0.777
Q5U1Y1	Ras-related protein Rab-34 OS=Rattus norvegicus OX=10116 GN=Rab34 PE=2 SV=1 - [RAB34_RAT]	29.1	0.013	0.013	0.013	0.013	0.013	0.013	0.013	0.013	0.013	0.013	0.975	0.825	1.757	1.946	1.628	0.013	0.013	0.013	0.013	0.013	#DIV/0!	#DIV/0!	0.009	0.056	#DIV/0!	#DIV/0!	0.009	0.047	#DIV/0!	#DIV/0!	0.009	0.099
Q5U2Q3	Ester hydrolase C11orf54 homolog OS=Rattus norvegicus PE=1 SV=1 - [CK054_RAT]	35.0	0.377	0.725	0.357	0.786	0.700	0.729	0.347	0.507	0.772	0.782	0.925	0.873	1.576	6.546	0.712	5.935	0.361	1.124	1.066	1.099	0.756	1.127	0.036	0.055	0.085	0.118	0.056	0.064	0.085	0.092	0.917	0.926
Q5U2R7	LRP chaperone MESD OS=Rattus norvegicus OX=10116 GN=Mesd PE=2 SV=1 - [MESD_RAT]	25.2	0.013	0.013	0.013	0.013	0.013	0.013	0.013	0.013	0.013	0.013	1.000	0.792	2.109	1.045	1.100	0.013	0.013	0.013	0.013	0.013	#DIV/0!	#DIV/0!	0.009	0.075	#DIV/0!	#DIV/0!	0.009	0.065	#DIV/0!	#DIV/0!	0.009	0.148
Q5U2U2	Crk-like protein OS=Rattus norvegicus GN=Crkl PE=1 SV=1 - [CRKL_RAT]	33.8	0.505	0.982	0.359	0.964	0.880	0.865	0.254	0.475	0.890	0.889	1.273	1.176	2.407	3.227	1.079	2.596	0.464	1.263	1.384	1.113	0.470	1.111	0.014	0.030	0.085	0.128	0.014	0.027	0.085	0.104	0.605	1.359
Q5U2X6	Coiled-coil domain-containing protein 47 OS=Rattus norvegicus OX=10116 GN=Ccdc47 PE=2 SV=1 - [CCD47_RAT]	55.7	0.013	0.013	0.013	0.013	0.013	0.013	0.013	0.013	0.013	0.013	2.110	2.377	6.507	3.068	1.203	0.013	0.013	0.013	0.013	0.013	#DIV/0!	#DIV/0!	0.009	0.027	#DIV/0!	#DIV/0!	0.009	0.022	#DIV/0!	#DIV/0!	0.009	0.061
Q5U300	Ubiquitin-like modifier-activating enzyme 1 OS=Rattus norvegicus GN=Uba1 PE=1 SV=1 - [UBA1_RAT]	117.7	0.555	1.089	0.517	1.014	1.014	1.070	0.425	0.648	1.183	1.166	1.090	1.266	2.258	1.452	1.344	1.341	0.790	1.480	1.331	1.394	0.470	0.998	0.014	0.034	0.056	0.112	0.036	0.052	0.056	0.084	0.756	1.325
Q5U3Z3	Isochorismatase domain-containing protein 2 OS=Rattus norvegicus OX=10116 GN=Isoc2 PE=2 SV=1 - [ISOC2_RAT]	23.1	0.528	1.137	0.536	0.921	0.911	0.692	0.286	0.238	1.820	1.438	1.409	1.473	2.846	2.171	1.321	1.953	0.595	1.388	1.488	1.352	0.917	0.972	0.014	0.029	0.056	0.105	0.127	0.114	0.354	0.274	0.354	1.426
Q5XFX0	Transgelin-2 OS=Rattus norvegicus GN=Tagln2 PE=2 SV=1 - [TAGL2_RAT]	22.4	0.640	0.915	0.531	1.073	0.857	0.866	0.368	0.545	0.951	0.858	1.221	0.994	2.262	1.521	0.958	1.894	0.647	1.385	1.302	1.284	0.756	1.083	0.036	0.060	0.056	0.112	0.014	0.032	0.056	0.098	0.917	1.149
Q5XHY5	Threonine--tRNA ligase, cytoplasmic OS=Rattus norvegicus GN=Tars PE=1 SV=1 - [SYTC_RAT]	80.5	0.914	0.932	0.771	1.057	0.909	1.545	0.631	0.762	0.806	0.636	1.147	1.285	1.787	1.231	0.961	1.222	0.869	1.717	1.292	0.771	0.184	0.728	0.022	0.043	0.354	0.358	0.085	0.093	0.127	0.131	0.605	1.588
Q5XI32	F-actin-capping protein subunit beta OS=Rattus norvegicus GN=Capzb PE=1 SV=1 - [CAPZB_RAT]	30.6	0.593	0.870	0.520	0.976	0.899	1.018	0.429	0.657	0.900	0.941	0.935	1.000	1.991	2.031	1.030	1.974	0.897	1.261	1.182	1.200	0.605	1.070	0.022	0.044	0.036	0.086	0.056	0.073	0.056	0.091	0.917	1.144
Q5XI78	2-oxoglutarate dehydrogenase, mitochondrial OS=Rattus norvegicus GN=Ogdh PE=1 SV=1 - [ODO1_RAT]	116.2	0.468	0.829	0.409	0.827	0.764	0.847	0.285	0.481	0.684	0.715	0.843	1.009	1.702	5.403	0.945	4.523	0.443	1.375	1.114	1.044	0.756	1.124	0.014	0.029	0.085	0.120	0.022	0.037	0.085	0.095	0.917	0.944
Q5XIA3	tRNA wybutosine-synthesizing protein 4 OS=Rattus norvegicus OX=10116 GN=Lcmt2 PE=2 SV=1 - [TYW4_RAT]	75.5	1.374	0.971	0.512	0.672	0.621	0.971	0.414	0.862	1.158	0.680	2.031	2.296	1.657	1.105	1.300	0.013	0.013	0.013	0.013	0.013	0.756	1.019	0.036	0.056	0.009	0.394	0.022	0.037	0.009	0.304	0.009	0.080
Q5XIB4	Ufm1-specific protease 2 OS=Rattus norvegicus OX=10116 GN=Ufsp2 PE=2 SV=1 - [UFSP2_RAT]	52.3	0.919	1.042	0.439	1.196	1.074	1.283	0.375	0.542	0.952	0.783	1.445	1.227	2.335	1.961	1.067	2.857	0.415	1.814	1.379	1.166	0.470	1.014	0.036	0.056	0.184	0.206	0.036	0.052	0.127	0.124	0.756	1.207
Q5XIE0	Acidic leucine-rich nuclear phosphoprotein 32 family member E OS=Rattus norvegicus OX=10116 GN=Anp32e PE=2 SV=1 - [AN32E_RAT]	29.4	0.574	1.002	0.289	1.228	0.968	1.015	0.283	0.461	1.075	1.061	1.308	1.072	2.715	1.872	1.203	1.887	0.804	1.586	1.398	1.154	0.917	1.007	0.036	0.057	0.085	0.125	0.022	0.038	0.056	0.086	0.756	1.232
Q5XIM9	T-complex protein 1 subunit beta OS=Rattus norvegicus GN=Cct2 PE=1 SV=3 - [TCPB_RAT]	57.4	0.736	1.167	0.575	1.204	1.081	1.395	0.510	0.743	1.042	0.851	1.312	1.380	2.295	2.470	1.283	2.243	0.745	1.698	1.827	1.138	0.756	0.924	0.014	0.029	0.127	0.158	0.056	0.064	0.085	0.093	0.470	1.425
Q5XIN6	Mitochondrial proton/calcium exchanger protein OS=Rattus norvegicus OX=10116 GN=Letm1 PE=1 SV=1 - [LETM1_RAT]	83.0	0.013	0.013	0.013	0.013	0.013	1.243	0.464	0.529	1.077	0.910	1.360	1.289	2.230	3.483	0.987	2.768	0.656	1.730	1.297	0.742	0.009	0.127	0.009	0.034	0.009	0.046	0.036	0.050	0.184	0.161	0.470	1.419
Q5XIP1	Protein pelota homolog OS=Rattus norvegicus GN=Pelo PE=1 SV=1 - [PELO_RAT]	43.4	1.888	0.978	1.578	1.082	1.014	0.013	0.013	0.013	0.013	0.013	0.013	0.013	0.013	0.013	0.013	0.013	0.013	0.013	0.013	0.013	0.009	0.051	0.009	0.064	0.009	0.057	#DIV/0!	#DIV/0!	#DIV/0!	#DIV/0!	#DIV/0!	#DIV/0!
Q5XIU5	Proteasome inhibitor PI31 subunit OS=Rattus norvegicus OX=10116 GN=Psmf1 PE=1 SV=1 - [PSMF1_RAT]	29.8	0.013	0.013	0.013	0.013	0.013	0.013	0.013	0.013	0.013	0.013	0.665	0.799	1.746	1.619	1.111	0.013	0.013	0.013	0.013	0.013	#DIV/0!	#DIV/0!	0.009	0.076	#DIV/0!	#DIV/0!	0.009	0.067	#DIV/0!	#DIV/0!	0.009	0.157
Q62639	GTP-binding protein Rheb OS=Rattus norvegicus OX=10116 GN=Rheb PE=1 SV=1 - [RHEB_RAT]	20.5	0.742	1.045	0.450	1.243	0.961	1.277	0.539	0.759	0.745	0.688	1.758	1.416	2.870	2.409	1.229	2.092	0.653	2.032	1.989	1.097	0.756	0.992	0.022	0.039	0.127	0.159	0.022	0.036	0.127	0.123	0.470	1.401
Q62725	Nuclear transcription factor Y subunit gamma OS=Rattus norvegicus OX=10116 GN=Nfyc PE=2 SV=1 - [NFYC_RAT]	37.2	0.013	0.013	0.013	0.013	0.013	0.013	0.013	0.013	0.013	0.013	1.228	1.089	2.066	1.735	1.211	0.013	0.013	0.013	0.013	0.013	#DIV/0!	#DIV/0!	0.009	0.052	#DIV/0!	#DIV/0!	0.009	0.044	#DIV/0!	#DIV/0!	0.009	0.094
Q62753	Syntaxin-binding protein 2 OS=Rattus norvegicus GN=Stxbp2 PE=2 SV=1 - [STXB2_RAT]	66.7	0.013	0.013	0.013	0.013	0.013	1.411	0.466	0.717	0.721	0.685	1.061	1.248	1.618	5.321	0.760	3.944	0.597	1.539	1.361	1.026	0.009	0.203	0.009	0.031	0.009	0.035	0.056	0.064	0.184	0.159	0.756	1.119
Q62818	Translation initiation factor eIF-2B subunit beta OS=Rattus norvegicus OX=10116 GN=Eif2b2 PE=2 SV=1 - [EI2BB_RAT]	38.9	0.013	0.013	0.013	0.013	0.013	0.013	0.013	0.013	0.013	0.013	1.230	1.043	1.922	1.622	0.931	0.013	0.013	0.013	0.013	0.013	#DIV/0!	#DIV/0!	0.009	0.061	#DIV/0!	#DIV/0!	0.009	0.053	#DIV/0!	#DIV/0!	0.009	0.113
Q62862	Dual specificity mitogen-activated protein kinase kinase 5 OS=Rattus norvegicus OX=10116 GN=Map2k5 PE=1 SV=1 - [MP2K5_RAT]	50.2	1.210	0.400	0.675	1.142	1.333	1.254	0.817	1.059	0.251	0.474	0.135	0.177	0.394	0.283	0.779	0.346	1.659	0.400	0.502	0.200	0.470	1.008	0.036	0.062	0.184	0.226	0.085	0.098	0.470	0.360	0.259	1.411
Q62930	Complement component C9 OS=Rattus norvegicus GN=C9 PE=1 SV=1 - [CO9_RAT]	62.2	0.599	0.857	0.403	0.954	0.867	0.781	0.434	0.529	1.055	1.013	1.121	1.015	2.175	3.347	1.142	4.113	0.497	1.404	1.219	1.390	0.756	1.085	0.014	0.031	0.085	0.118	0.022	0.037	0.085	0.092	0.756	1.141
Q62931	Golgi SNAP receptor complex member 1 OS=Rattus norvegicus GN=Gosr1 PE=1 SV=1 - [GOSR1_RAT]	28.5	0.013	0.013	0.013	0.013	0.013	0.013	0.013	0.013	0.013	0.013	1.189	1.175	2.145	1.490	1.004	0.013	0.013	0.013	0.013	0.013	#DIV/0!	#DIV/0!	0.009	0.058	#DIV/0!	#DIV/0!	0.009	0.049	#DIV/0!	#DIV/0!	0.009	0.105

Q62940	E3 ubiquitin-protein ligase NEDD4 OS=Rattus norvegicus GN=Nedd4 PE=1 SV=1 - [NEDD4_RAT]	102.3	0.852	0.735	0.677	1.098	0.857	1.025	0.486	0.747	0.928	1.171	1.081	1.564	2.091	3.369	1.161	2.276	0.855	1.267	1.172	1.165	0.605	0.993	0.022	0.040	0.036	0.084	0.036	0.050	0.085	0.098	0.605	1.357
Q62991	Sec1 family domain-containing protein 1 OS=Rattus norvegicus GN=Scfd1 PE=1 SV=1 - [SCFD1_RAT]	72.2	0.871	1.042	0.542	1.118	0.994	1.587	0.546	0.666	0.773	0.863	1.679	1.620	2.281	1.761	1.044	1.447	0.686	2.260	1.545	1.015	0.470	0.961	0.022	0.040	0.184	0.210	0.022	0.037	0.184	0.161	0.184	1.030
Q63041	Alpha-1-macroglobulin OS=Rattus norvegicus GN=A1m PE=1 SV=1 - [A1M_RAT]	167.0	0.481	0.552	0.443	0.586	0.741	0.639	0.354	0.657	0.895	0.717	0.726	0.770	1.748	2.026	0.842	3.489	0.564	1.274	0.840	1.195	0.354	1.088	0.022	0.045	0.036	0.088	0.056	0.076	0.127	0.129	0.917	1.148
Q63083	Nucleobindin-1 OS=Rattus norvegicus OX=10116 GN=Nucb1 PE=1 SV=1 - [NUCB1_RAT]	53.5	0.013	0.013	0.013	0.013	0.013	0.013	0.013	0.013	0.013	0.013	1.516	1.625	3.246	3.124	1.561	0.013	0.013	0.013	0.013	0.013	#DIV/0!	#DIV/0!	0.009	0.029	#DIV/0!	#DIV/0!	0.009	0.023	#DIV/0!	#DIV/0!	0.009	0.065
Q63228	Glia maturation factor beta OS=Rattus norvegicus GN=Gmfb PE=1 SV=2 - [GMFB_RAT]	16.7	0.013	0.013	0.013	0.013	0.013	1.232	0.525	0.707	0.726	0.516	0.870	0.727	1.456	1.819	0.682	0.013	0.013	0.013	0.013	0.013	0.009	0.337	0.009	0.089	#DIV/0!	#DIV/0!	0.127	0.126	0.009	0.630	0.009	0.199
Q63270	Cytoplasmic aconitate hydratase OS=Rattus norvegicus GN=Aco1 PE=1 SV=1 - [ACOC_RAT]	98.1	0.504	0.861	0.432	0.875	0.805	0.933	0.418	0.582	0.879	0.983	0.888	0.998	1.757	3.760	0.977	3.134	0.650	1.227	1.088	1.145	0.354	1.064	0.014	0.033	0.056	0.107	0.056	0.066	0.056	0.082	0.917	1.025
Q63413	Spliceosome RNA helicase Ddx39b OS=Rattus norvegicus GN=Ddx39b PE=1 SV=3 - [DX39B_RAT]	49.0	0.888	0.969	0.640	1.027	1.026	1.041	0.620	0.823	0.841	0.981	1.308	1.177	2.127	2.461	1.069	1.776	0.795	1.587	1.264	0.858	0.605	0.966	0.014	0.030	0.354	0.352	0.014	0.027	0.184	0.165	0.354	1.462
Q63486	Ras-related GTP-binding protein A OS=Rattus norvegicus GN=Rraga PE=1 SV=1 - [RRAGA_RAT]	36.5	0.515	1.490	0.436	1.087	1.093	0.954	0.572	0.520	1.353	1.248	1.517	1.638	2.703	2.081	1.288	1.766	0.780	1.961	1.898	1.720	0.756	0.926	0.022	0.039	0.056	0.089	0.022	0.036	0.056	0.073	0.917	0.967
Q63487	Ras-related GTP-binding protein B OS=Rattus norvegicus GN=RragB PE=1 SV=1 - [RRAGB_RAT]	43.2	0.013	0.013	0.013	0.013	0.013	0.013	0.013	0.013	0.013	0.013	1.738	1.858	3.212	1.369	1.575	0.013	0.013	0.013	0.013	0.013	#DIV/0!	#DIV/0!	0.009	0.032	#DIV/0!	#DIV/0!	0.009	0.025	#DIV/0!	#DIV/0!	0.009	0.071
Q63569	26S protease regulatory subunit 6A OS=Rattus norvegicus GN=Psmc3 PE=2 SV=1 - [PRS6A_RAT]	49.1	0.652	1.194	0.486	1.149	0.948	1.237	0.381	0.614	0.875	0.742	1.428	1.491	2.334	2.052	1.223	1.741	0.821	1.525	1.459	0.876	0.605	1.020	0.014	0.030	0.184	0.215	0.022	0.038	0.056	0.095	0.354	1.450
Q63570	26S protease regulatory subunit 6B OS=Rattus norvegicus GN=Psmc4 PE=1 SV=1 - [PRS6B_RAT]	47.4	0.374	1.369	0.277	1.066	0.903	0.726	0.397	0.416	1.132	0.756	1.361	1.463	2.492	2.355	1.310	2.073	0.441	2.423	1.834	0.870	0.917	1.044	0.036	0.055	0.184	0.209	0.014	0.027	0.085	0.098	0.605	1.337
Q63610	Tropomyosin alpha-3 chain OS=Rattus norvegicus GN=Tpm3 PE=1 SV=2 - [TPM3_RAT]	29.0	0.855	0.892	0.491	0.924	0.827	0.874	0.559	0.623	0.889	0.751	1.250	0.925	2.189	2.555	0.855	1.930	0.680	1.138	1.225	1.049	0.470	1.085	0.036	0.058	0.085	0.131	0.036	0.053	0.056	0.102	0.470	1.513
Q63639	Retinal dehydrogenase 2 OS=Rattus norvegicus GN=Aldh1a2 PE=1 SV=2 - [AL1A2_RAT]	56.6	0.508	1.006	0.398	1.031	0.903	0.773	0.432	0.552	1.133	1.067	1.128	1.208	2.335	1.617	1.288	1.320	0.680	1.491	1.305	1.580	0.605	1.069	0.014	0.036	0.056	0.119	0.022	0.039	0.056	0.094	0.917	1.113
Q63663	Guanylate-binding protein 1 OS=Rattus norvegicus GN=Gbp2 PE=1 SV=2 - [GBP2_RAT]	67.1	0.013	0.013	0.013	0.013	0.013	0.013	0.013	0.013	0.013	0.013	1.044	1.054	2.172	1.071	1.306	0.013	0.013	0.013	0.013	0.013	#DIV/0!	#DIV/0!	0.009	0.063	#DIV/0!	#DIV/0!	0.009	0.054	#DIV/0!	#DIV/0!	0.009	0.117
Q63678	Zinc-alpha-2-glycoprotein OS=Rattus norvegicus GN=Azgp1 PE=2 SV=1 - [ZA2G_RAT]	34.0	0.604	0.895	0.405	0.867	0.730	0.835	0.653	0.604	1.067	0.956	0.868	0.999	1.610	2.181	0.923	2.419	0.664	1.395	1.288	1.419	0.354	1.030	0.022	0.045	0.056	0.108	0.127	0.122	0.056	0.079	0.756	1.323
Q63692	Hsp90 co-chaperone Cdc37 OS=Rattus norvegicus GN=Cdc37 PE=1 SV=2 - [CDC37_RAT]	44.5	0.405	1.190	0.258	1.292	1.023	0.840	0.143	0.348	1.140	0.942	1.449	1.536	2.452	2.440	1.293	1.977	0.578	1.847	1.717	0.980	0.354	1.038	0.014	0.029	0.184	0.212	0.014	0.027	0.085	0.102	0.470	1.428
Q63768	Adapter molecule crk OS=Rattus norvegicus GN=Crk PE=1 SV=1 - [CRK_RAT]	33.8	0.688	1.211	0.448	1.280	1.264	1.039	0.543	0.582	1.219	1.265	1.451	1.454	2.918	1.447	1.528	1.374	0.652	1.684	2.052	1.602	0.756	0.911	0.014	0.028	0.085	0.118	0.014	0.025	0.056	0.075	0.917	1.006
Q63798	Proteasome activator complex subunit 2 OS=Rattus norvegicus GN=Psme2 PE=2 SV=3 - [PSME2_RAT]	26.8	0.707	1.104	0.553	1.016	0.938	1.168	0.597	0.753	1.103	0.848	1.173	1.331	2.577	2.151	1.400	1.932	0.649	1.242	1.832	1.306	0.756	0.949	0.014	0.030	0.085	0.123	0.014	0.026	0.085	0.096	0.354	1.430
Q64057	Alpha-aminoadipic semialdehyde dehydrogenase OS=Rattus norvegicus GN=Aldh7a1 PE=1 SV=2 - [AL7A1_RAT]	58.7	0.460	1.070	0.374	0.979	0.856	0.793	0.329	0.454	1.118	1.065	1.046	1.204	2.146	3.040	1.023	2.425	0.647	1.377	1.251	1.425	0.917	1.040	0.036	0.057	0.056	0.103	0.085	0.087	0.056	0.084	0.917	1.028
Q641Y8	ATP-dependent RNA helicase DDX1 OS=Rattus norvegicus GN=Ddx1 PE=2 SV=1 - [DDX1_RAT]	82.4	0.842	0.922	0.702	1.075	1.026	0.932	0.372	0.456	1.386	1.407	1.369	1.490	2.352	2.013	1.529	2.015	0.614	1.748	1.657	1.426	0.917	0.937	0.014	0.029	0.127	0.160	0.036	0.050	0.056	0.075	0.756	1.179
Q641Z7	Acid sphingomyelinase-like phosphodiesterase 3a OS=Rattus norvegicus OX=10116 GN=Smpdl3a PE=2 SV=1 - [ASM3A_RAT]	49.9	0.013	0.013	0.013	0.013	0.013	0.013	0.013	0.013	0.013	0.013	2.281	1.932	4.136	1.384	1.068	0.013	0.013	0.013	0.013	0.013	#DIV/0!	#DIV/0!	0.009	0.030	#DIV/0!	#DIV/0!	0.009	0.024	#DIV/0!	#DIV/0!	0.009	0.066
Q64232	Very-long-chain enoyl-CoA reductase OS=Rattus norvegicus OX=10116 GN=Tecr PE=1 SV=1 - [TECR_RAT]	36.1	0.013	0.013	0.013	0.013	0.013	0.013	0.013	0.013	0.013	0.013	0.980	2.499	1.257	1.716	0.761	0.013	0.013	0.013	0.013	0.013	#DIV/0!	#DIV/0!	0.009	0.054	#DIV/0!	#DIV/0!	0.009	0.046	#DIV/0!	#DIV/0!	0.009	0.098
Q642A4	UPF0598 protein C8orf82 homolog OS=Rattus norvegicus OX=10116 PE=2 SV=1 - [CH082_RAT]	24.4	0.678	0.732	0.673	0.856	0.733	0.013	0.013	0.013	0.013	0.013	1.023	0.938	1.845	3.713	1.126	0.013	0.013	0.013	0.013	0.013	0.009	0.372	0.014	0.031	0.009	0.732	0.009	0.032	#DIV/0!	#DIV/0!	0.009	0.078
Q64303	Serine/threonine-protein kinase PAK 2 OS=Rattus norvegicus GN=Pak2 PE=1 SV=1 - [PAK2_RAT]	57.9	0.932	0.798	0.743	1.020	1.075	1.323	0.600	0.828	0.938	0.974	1.218	1.081	2.523	1.454	1.382	1.376	0.753	1.607	1.311	1.150	0.917	0.931	0.014	0.032	0.085	0.126	0.036	0.051	0.184	0.164	0.470	1.500
Q64640	Adenosine kinase OS=Rattus norvegicus GN=Adk PE=1 SV=3 - [ADK_RAT]	40.1	0.596	0.992	0.510	1.074	1.020	1.053	0.421	0.741	1.055	1.102	1.057	1.119	2.220	1.344	1.201	1.175	0.781	1.315	1.349	1.403	0.605	0.995	0.022	0.043	0.056	0.119	0.022	0.039	0.056	0.093	0.917	1.189
Q66H59	N-acetylneuraminate lyase OS=Rattus norvegicus OX=10116 GN=Npl PE=2 SV=1 - [NPL_RAT]	35.1	0.013	0.013	0.013	0.013	0.013	0.013	0.013	0.013	0.013	0.013	1.111	1.315	1.522	3.636	0.566	0.013	0.013	0.013	0.013	0.013	#DIV/0!	#DIV/0!	0.009	0.043	#DIV/0!	#DIV/0!	0.009	0.035	#DIV/0!	#DIV/0!	0.009	0.082
Q66H71	Serine/threonine-protein phosphatase CPPED1 OS=Rattus norvegicus GN=Cpped1 PE=1 SV=1 - [CPPED_RAT]	35.2	0.580	0.974	0.418	1.042	1.076	0.977	0.417	0.614	0.958	1.046	0.976	1.136	2.033	1.828	1.156	1.836	0.665	1.338	1.253	0.997	0.756	1.030	0.036	0.059	0.127	0.170	0.036	0.054	0.085	0.105	0.756	1.364
Q66HA8	Heat shock protein 105 kDa OS=Rattus norvegicus GN=Hsph1 PE=2 SV=1 - [HS105_RAT]	96.4	0.778	0.907	0.625	0.992	0.999	1.164	0.513	0.673	0.956	0.902	0.939	1.114	1.927	1.647	1.259	1.300	0.972	1.256	1.117	1.049	0.756	0.985	0.036	0.059	0.036	0.089	0.056	0.072	0.056	0.101	0.470	1.622
Q66HF1	NADH-ubiquinone oxidoreductase 75 kDa subunit, mitochondrial OS=Rattus norvegicus GN=Ndufs1 PE=1 SV=1 - [NDUS1_RAT]	79.4	0.556	1.053	0.415	1.066	0.930	0.883	0.494	0.634	1.324	1.195	1.383	1.088	2.787	1.989	1.218	2.251	0.672	1.293	1.479	1.416	0.605	0.996	0.014	0.031	0.056	0.099	0.056	0.065	0.085	0.095	0.917	1.030
Q66HR2	Microtubule-associated protein RP/EB family member 1 OS=Rattus norvegicus GN=Mapre1 PE=1 SV=3 - [MARE1_RAT]	30.0	0.689	0.820	0.595	1.084	0.950	1.004	0.432	0.639	0.949	0.981	1.291	1.186	2.508	2.354	1.038	2.349	0.495	1.575	1.572	1.581	0.917	0.997	0.022	0.040	0.127	0.161	0.014	0.027	0.085	0.095	0.917	1.012
Q68FQ0	T-complex protein 1 subunit epsilon OS=Rattus norvegicus GN=Cct5 PE=1 SV=1 - [TCPE_RAT]	59.5	0.753	1.031	0.616	1.098	1.036	1.474	0.640	0.776	0.838	0.808	1.143	1.188	1.977	2.080	1.100	1.639	0.882	1.381	1.246	0.914	0.917	0.938	0.014	0.032	0.184	0.218	0.056	0.067	0.085	0.101	0.470	1.544
Q68FS2	COP9 signalosome complex subunit 4 OS=Rattus norvegicus GN=Cops4 PE=1 SV=1 - [CSN4_RAT]	46.3	0.650	1.073	0.479	1.005	0.914	0.964	0.375	0.493	1.052	0.972	0.949	1.147	1.952	1.689	1.136	1.914	0.818	1.613	1.278	0.925	0.756	1.056	0.036	0.060	0.127	0.167	0.056	0.074	0.184	0.167	0.605	1.501
Q68FS4	Cytosol aminopeptidase OS=Rattus norvegicus GN=Lap3 PE=1 SV=1 - [AMPL_RAT]	56.1	0.613	1.010	0.463	0.948	0.867	0.954	0.429	0.570	1.039	0.940	1.099	1.209	2.082	1.711	1.168	1.847	0.703	1.752	1.437	1.120	0.917	1.021	0.014	0.037	0.056	0.106	0.014	0.031	0.056	0.085	0.917	1.101
Q68FT9	Selenocysteine lyase OS=Rattus norvegicus GN=Scly PE=1 SV=1 - [SCLY_RAT]	47.2	0.431	1.145	0.229	0.995	1.093	0.834	0.345	0.391	1.236	1.137	1.160	1.159	2.410	2.714	1.089	2.788	0.577	1.428	1.381	1.392	0.917	1.020	0.036	0.056	0.056	0.095	0.085	0.086	0.056	0.077	0.756	1.183
Q6AXQ5	2',5'-phosphodiesterase 12 OS=Rattus norvegicus OX=10116 GN=Pde12 PE=2 SV=1 - [PDE12_RAT]	67.1	0.013	0.013	0.013	0.013	0.013	0.013	0.013	0.013	0.013	0.013	3.360	2.691	6.613	4.173	2.645	0.013	0.013	0.013	0.013	0.013	#DIV/0!	#DIV/0!	0.009	0.027	#DIV/0!	#DIV/0!	0.009	0.022	#DIV/0!	#DIV/0!	0.009	0.060
Q6AY09	Heterogeneous nuclear ribonucleoprotein H2 OS=Rattus norvegicus GN=Hnrnph2 PE=1 SV=1 - [HNRH2_RAT]	49.3	0.926	0.815	0.695	1.060	0.992	1.030	0.605	0.756	0.883	1.008	1.070	1.096	2.192	1.685	1.136	1.359	0.695	1.165	1.177	1.034	0.756	0.951	0.014	0.034	0.184	0.223	0.014	0.030	0.085	0.108	0.470	1.614
Q6AY30	Saccharopine dehydrogenase-like oxidoreductase OS=Rattus norvegicus OX=10116 GN=Sccpdh PE=1 SV=1 - [SCPDL_RAT]	47.1	1.278	0.839	0.313	0.832	0.627	2.435	0.603	0.441	1.102	0.960	1.578	1.151	1.892	3.315	0.921	2.287	0.577	1.747	1.328	0.896	0.605	0.936	0.036	0.056	0.127	0.167	0.184	0.153	0.605	0.440	0.470	1.434
Q6AYB4	Heat shock 70 kDa protein 14 OS=Rattus norvegicus OX=10116 GN=Hspa14 PE=2 SV=1 - [HSP7E_RAT]	54.4	0.013	0.013	0.013	0.013	0.013	0.013	0.013	0.013	0.013	0.013	2.112	2.426	3.985	5.039	1.201	0.013	0.013	0.013	0.013	0.013	#DIV/0!	#DIV/0!	0.009	0.027	#DIV/0!	#DIV/0!	0.009	0.022	#DIV/0!	#DIV/0!	0.009	0.061
Q6AYK8	Eukaryotic translation initiation factor 3 subunit D OS=Rattus norvegicus GN=Eif3d PE=1 SV=1 - [EIF3D_RAT]	63.9	0.965	0.873	0.608	0.924	0.952	1.390	0.424	0.733	0.556	0.786	1.069	1.127	2.014	1.485	0.946	1.092	0.813	1.675	1.184	0.760	0.259	0.917	0.036	0.060	0.354	0.363	0.056	0.075	0.127	0.135	0.470	1.665
Q6AYQ8	Acylpyruvase FAHD1, mitochondrial OS=Rattus norvegicus OX=10116 GN=Fahd1 PE=1 SV=1 - [FAHD1_RAT]	24.5	0.681	0.822	0.563	0.948	0.798	1.012	0.517	0.625	0.917	0.939	1.044	0.966	1.827	4.204	0.936	3.796	0.542	1.344	1.375	1.240	0.756	1.067	0.022	0.040	0.127	0.159	0.056	0.065	0.085	0.092	0.917	0.970
Q6AYS7	Aminoacylase-1A OS=Rattus norvegicus GN=Acy1a PE=1 SV=1 - [ACY1A_RAT]	45.8	0.452	0.629	0.309	0.734	0.631	0.702	0.349	0.443	0.678	0.735	0.762	0.826	1.380	6.060	0.716	4.833	0.614	1.083	0.994	0.934	0.605	1.144	0.022	0.040	0.056	0.098	0.022	0.037	0.056	0.078	0.917	0.946
Q6AYT0	Quinone oxidoreductase OS=Rattus norvegicus GN=Cryz PE=2 SV=1 - [QOR_RAT]	35.0	0.207	0.370	0.201	0.429	0.378	0.482	0.249	0.337	0.377	0.424	0.612	0.592	0.922	8.685	0.580	6.124	0.340	0.713	0.674	0.646	0.470	1.144	0.014	0.030	0.056	0.121	0.014	0.026	0.056	0.093	0.917	0.928
Q6AYT3	tRNA-splicing ligase RtcB homolog OS=Rattus norvegicus GN=Rtcb PE=2 SV=1 - [RTCB_RAT]	55.2	0.958	0.770	0.623	1.094	0.937	1.078	0.457	0.773	0.799	0.801	1.229	1.172	2.191	3.071	1.052	4.028	0.265	2.045	1.996	1.628	0.605	1.018	0.022	0.040	0.127	0.157	0.022	0.037	0.127	0.122	0.756	1.116
Q6B345	Protein S100-A11 OS=Rattus norvegicus GN=S100a11 PE=3 SV=1 - [S10AB_RAT]	11.1	0.370	1.181	0.303	1.012	0.835	0.455	0.269	0.323	1.028	0.914	1.687	1.017	3.709	2.650	1.343	2.995	0.383	1.642	1.791	1.666	0.605	1.120	0.022	0.039	0.056	0.090	0.022	0.037	0.056	0.077	0.756	1.112
Q6E0V2	Katanin p60 ATPase-containing subunit A1 OS=Rattus norvegicus OX=10116 GN=Katna1 PE=1 SV=1 - [KTNA1_RAT]	55.8	1.560	0.537	1.412	1.021	1.068	0.013	0.013	0.013	0.013	0.013	0.013	0.013	0.013	0.013	0.013	0.529	2.060	0.446	0.561	0.480	0.009	0.054	0.009	0.086	0.184	0.225	#DIV/0!	#DIV/0!	0.009	0.315	0.009	1.173
Q6EV70	GDP-fucose protein O-fucosyltransferase 1 OS=Rattus norvegicus GN=Pofut1 PE=2 SV=1 - [OFUT1_RAT]	45.0	0.013	0.013	0.013	0.013	0.013	0.814	0.261	0.434	0.978	1.135	1.320	1.325	2.463	2.089	1.489	0.013	0.013	0.013	0.013	0.013	0.009	0.399	0.009	0.039	#DIV/0!	#DIV/0!	0.014	0.027	0.009	0.802	0.009	0.077
Q6IRK9	Carboxypeptidase Q OS=Rattus norvegicus GN=Cpq PE=1 SV=1 - [CBPQ_RAT]	52.0	0.304	0.488	0.233	0.454	0.449	0.443	0.227	0.325	0.577	0.588	0.654	0.683	1.174	5.465	0.713	4.966	0.415	0.949	0.910	0.854	0.756	1.135	0.014	0.039	0.056	0.122	0.014	0.031	0.056	0.096	0.917	0.981
Q6MG61	Chloride intracellular channel protein 1 OS=Rattus norvegicus GN=Clic1 PE=1 SV=1 - [CLIC1_RAT]	27.0	0.542	0.733	0.401	0.868	0.770	0.858	0.382	0.582	0.753	0.806	0.847	0.841	1.403	3.529	0.934	3.355	0.736	1.109	1.141	1.097	0.917	1.067	0.036	0.060	0.036	0.084	0.036	0.055	0.056	0.084	0.917	1.053
Q6MGD0	Protein CutA OS=Rattus norvegicus GN=Cuta PE=1 SV=2 - [CUTA_RAT]	18.6	0.737	1.297	0.359	1.315	1.007	0.828	0.457	0.545	1.420	1.185	1.656	1.393	3.218	1.686	1.248	1.617	0.598	2.253	2.076	1.789	0.917	0.934	0.036	0.054	0.085	0.117	0.036	0.050	0.056	0.073	0.756	1.138
Q6P4Z9	COP9 signalosome complex subunit 8 OS=Rattus norvegicus GN=Cops8 PE=2 SV=1 - [CSN8_RAT]	23.2	0.013	0.013	0.013	0.013	0.013	1.381	0.770	0.866	0.705	0.687	0.757	0.803	1.581	1.419	0.973	1.415	1.134	1.255	1.068	0.633	0.009	0.102	0.009	0.091	0.009	0.079	0.184	0.163	0.354	0.281	0.917	1.288
Q6P502	T-complex protein 1 subunit gamma OS=Rattus norvegicus GN=Cct3 PE=1 SV=1 - [TCPG_RAT]	60.6	0.854	0.903	0.702	1.005	0.887	1.538	0.611	0.835	0.740	0.681	0.910	1.060	1.663	2.232	1.033	1.642	0.894	1.246	1.110	0.813	0.259	0.891	0.022	0.043	0.184	0.222	0.056	0.070	0.127	0.132	0.470	1.618
Q6P6R2	Dihydrolipoyl dehydrogenase, mitochondrial OS=Rattus norvegicus GN=Dld PE=1 SV=1 - [DLDH_RAT]	54.0	0.516	1.003	0.488	0.889	0.810	1.047	0.362	0.579	0.964	0.986	1.219	1.366	2.547	2.238	1.338	2.031	0.679	1.570	1.503	1.337	0.605	1.085	0.014	0.031	0.056	0.104	0.014	0.026	0.056	0.081	0.605	1.366
Q6P6S4	Nucleotide exchange factor SIL1 OS=Rattus norvegicus GN=Sil1 PE=2 SV=1 - [SIL1_RAT]	52.3	0.713	1.017	0.444	1.069	1.048	1.157	0.586	0.637	1.202	1.028	1.152	1.259	2.547	1.837	1.509	2.223	0.547	1.666	1.742	1.338	0.605	0.962	0.014	0.030	0.085	0.120	0.036	0.050	0.127	0.123	0.917	1.018
Q6P6V0	Glucose-6-phosphate isomerase OS=Rattus norvegicus GN=Gpi PE=1 SV=1 - [G6PI_RAT]	62.8	0.585	0.871	0.473	0.925	0.880	0.887	0.369	0.590	0.756	0.889	1.124	1.101	2.233	4.022	0.958	3.764	0.599	1.398	1.258	1.204	0.917	1.053	0.014	0.029	0.056	0.091	0.014	0.026	0.056	0.075	0.917	0.959
Q6P756	Adaptin ear-binding coat-associated protein 2 OS=Rattus norvegicus GN=Necap2 PE=1 SV=2 - [NECP2_RAT]	28.4	0.013	0.013	0.013	0.013	0.013	0.818	0.377	0.445	0.837	0.831	0.819	0.876	1.854	2.032	0.959	2.576	0.799	1.535	1.324	1.558	0.009	0.770	0.009	0.065	0.009	0.040	0.036	0.057	0.056	0.081	0.756	1.281
Q6P7P5	Basic leucine zipper and W2 domain-containing protein 1 OS=Rattus norvegicus GN=Bzw1 PE=2 SV=1 - [BZW1_RAT]	48.0	0.013	0.013	0.013	0.013	0.013	1.559	0.669	0.559	0.577	0.500	2.133	2.418	2.529	4.061	1.302	3.703	0.369	4.058	1.878	0.791	0.009	0.276	0.009	0.028	0.009	0.029	0.022	0.036	0.184	0.159	0.470	1.368
Q6P7Q4	Lactoylglutathione lyase OS=Rattus norvegicus GN=Glo1 PE=1 SV=3 - [LGUL_RAT]	20.8	0.595	1.128	0.386	1.200	0.936	0.871	0.216	0.426	0.954	0.954	1.499	1.239	2.546	1.215	1.201	1.093	0.763	1.469	1.418	1.391	0.470	1.090	0.014	0.032	0.127	0.169	0.014	0.031	0.056	0.105	0.470	1.507
Q6PDU7	ATP synthase subunit g, mitochondrial OS=Rattus norvegicus OX=10116 GN=Atp5mg PE=1 SV=2 - [ATP5L_RAT]	11.4	0.398	1.115	0.182	0.952	0.798	0.698	0.305	0.250	0.754	0.676	1.141	1.189	2.112	4.374	0.853	3.231	0.642	1.690	1.305	0.702	0.354	1.082	0.036	0.055	0.184	0.214	0.014	0.027	0.085	0.104	0.605	1.311
Q6PEC4	S-phase kinase-associated protein 1 OS=Rattus norvegicus GN=Skp1 PE=2 SV=3 - [SKP1_RAT]	18.7	0.765	0.838	0.547	1.047	0.835	0.981	0.411	0.588	0.995	0.933	1.697	1.047	2.329	3.494	1.125	3.070	0.506	1.831	1.828	1.439	0.917	1.009	0.014	0.028	0.127	0.158	0.014	0.025	0.085	0.092	0.917	0.946
Q6Q0N1	Cytosolic non-specific dipeptidase OS=Rattus norvegicus GN=Cndp2 PE=1 SV=1 - [CNDP2_RAT]	52.7	0.477	0.787	0.342	0.680	0.698	0.726	0.411	0.532	0.759	0.787	0.978	0.980	1.702	4.918	0.832	4.762	0.483	1.249	1.147	1.003	0.470	1.126	0.014	0.031	0.056	0.093	0.014	0.027	0.085	0.094	0.917	0.950
Q6QI44	N-alpha-acetyltransferase 25, NatB auxiliary subunit OS=Rattus norvegicus GN=Naa25 PE=2 SV=1 - [NAA25_RAT]	111.2	0.013	0.013	0.013	0.013	0.013	1.658	0.730	0.920	0.686	0.621	0.896	1.106	1.498	1.634	0.936	1.381	1.503	2.064	2.107	1.158	0.009	0.075	0.009	0.075	0.009	0.036	0.184	0.160	0.056	0.073	0.127	0.761
Q6SKG1	Acyl-coenzyme A synthetase ACSM3, mitochondrial OS=Rattus norvegicus OX=10116 GN=Acsm3 PE=2 SV=1 - [ACSM3_RAT]	65.7	0.013	0.013	0.013	0.013	0.013	1.238	0.616	0.730	0.777	0.666	0.928	1.004	1.594	4.776	0.848	5.045	0.471	1.232	1.089	0.820	0.009	0.189	0.009	0.036	0.009	0.033	0.056	0.064	0.354	0.272	0.756	1.131
Q6TEK4	Vitamin K epoxide reductase complex subunit 1 OS=Rattus norvegicus OX=10116 GN=Vkorc1 PE=1 SV=1 - [VKOR1_RAT]	17.8	0.013	0.013	0.013	0.013	0.013	0.013	0.013	0.013	0.013	0.013	1.005	0.904	1.769	1.041	0.932	0.013	0.013	0.013	0.013	0.013	#DIV/0!	#DIV/0!	0.009	0.082	#DIV/0!	#DIV/0!	0.009	0.073	#DIV/0!	#DIV/0!	0.009	0.182
Q711G3	Isoamyl acetate-hydrolyzing esterase 1 homolog OS=Rattus norvegicus GN=Iah1 PE=2 SV=2 - [IAH1_RAT]	28.0	0.312	0.694	0.252	0.663	0.648	0.540	0.230	0.398	0.618	0.655	0.729	0.826	1.253	6.365	0.691	4.818	0.476	0.953	0.853	0.852	0.470	1.133	0.022	0.041	0.056	0.113	0.014	0.028	0.056	0.093	0.917	0.955
Q792I0	Protein lin-7 homolog C OS=Rattus norvegicus GN=Lin7c PE=1 SV=1 - [LIN7C_RAT]	21.8	0.013	0.013	0.013	0.013	0.013	0.013	0.013	0.013	0.013	0.013	1.069	0.930	1.827	1.906	0.913	0.013	0.013	0.013	0.013	0.013	#DIV/0!	#DIV/0!	0.009	0.063	#DIV/0!	#DIV/0!	0.009	0.055	#DIV/0!	#DIV/0!	0.009	0.118
Q794F9	4F2 cell-surface antigen heavy chain OS=Rattus norvegicus GN=Slc3a2 PE=1 SV=1 - [4F2_RAT]	58.0	0.786	0.921	0.524	1.001	0.838	1.538	0.618	0.638	1.160	0.751	1.044	1.052	1.820	1.694	0.995	1.549	0.850	1.596	1.445	0.727	0.917	0.955	0.022	0.044	0.184	0.221	0.184	0.157	0.184	0.164	0.470	1.599
Q7TPB1	T-complex protein 1 subunit delta OS=Rattus norvegicus GN=Cct4 PE=1 SV=3 - [TCPD_RAT]	58.1	0.890	1.050	0.832	1.153	0.929	1.500	0.749	0.824	0.787	0.709	1.197	1.277	1.910	2.183	1.027	1.500	0.876	1.298	1.369	0.720	0.127	0.557	0.036	0.056	0.470	0.441	0.056	0.066	0.354	0.279	0.470	1.566
Q7TQ94	Deaminated glutathione amidase OS=Rattus norvegicus OX=10116 GN=Nit1 PE=2 SV=2 - [NIT1_RAT]	36.1	0.664	0.859	0.531	0.906	0.853	0.997	0.486	0.666	0.945	1.026	0.933	0.975	1.822	2.332	1.032	2.022	0.807	1.281	1.186	1.152	0.354	1.011	0.014	0.038	0.056	0.118	0.127	0.119	0.056	0.088	0.917	1.139
Q80U96	Exportin-1 OS=Rattus norvegicus GN=Xpo1 PE=1 SV=1 - [XPO1_RAT]	123.0	0.716	0.738	0.446	0.912	0.587	2.463	0.356	0.490	0.724	1.112	1.577	1.355	1.897	3.229	0.890	2.598	0.524	2.036	1.418	0.919	0.756	0.978	0.022	0.041	0.085	0.125	0.127	0.114	0.259	0.210	0.756	1.168
Q80Z70	Protein sel-1 homolog 1 OS=Rattus norvegicus OX=10116 GN=Sel1l PE=1 SV=2 - [SE1L1_RAT]	88.6	0.013	0.013	0.013	0.013	0.013	0.013	0.013	0.013	0.013	0.013	1.866	1.925	3.499	3.386	1.701	0.013	0.013	0.013	0.013	0.013	#DIV/0!	#DIV/0!	0.009	0.028	#DIV/0!	#DIV/0!	0.009	0.022	#DIV/0!	#DIV/0!	0.009	0.063
Q8CGV7	Thiamine-triphosphatase OS=Rattus norvegicus GN=Thtpa PE=2 SV=3 - [THTPA_RAT]	24.5	0.013	0.013	0.013	0.013	0.013	0.013	0.013	0.013	0.013	0.013	0.893	0.817	1.951	0.983	0.923	0.013	0.013	0.013	0.013	0.013	#DIV/0!	#DIV/0!	0.009	0.087	#DIV/0!	#DIV/0!	0.009	0.078	#DIV/0!	#DIV/0!	0.009	0.193
Q8CIN7	Inositol monophosphatase 2 OS=Rattus norvegicus OX=10116 GN=Impa2 PE=2 SV=1 - [IMPA2_RAT]	31.8	0.013	0.013	0.013	0.013	0.013	0.013	0.013	0.013	0.013	0.013	1.074	1.496	2.549	2.993	1.235	0.013	0.013	0.013	0.013	0.013	#DIV/0!	#DIV/0!	0.009	0.034	#DIV/0!	#DIV/0!	0.009	0.027	#DIV/0!	#DIV/0!	0.009	0.073
Q8K1Q0	Glycylpeptide N-tetradecanoyltransferase 1 OS=Rattus norvegicus GN=Nmt1 PE=1 SV=1 - [NMT1_RAT]	56.8	0.559	1.082	0.441	1.209	1.047	0.548	0.208	0.379	0.894	1.338	1.388	1.591	2.858	1.628	1.196	1.566	0.327	1.912	1.715	1.646	0.354	1.023	0.022	0.040	0.127	0.162	0.022	0.038	0.085	0.101	0.756	1.195
Q8K3E7	Protein dpy-30 homolog OS=Rattus norvegicus OX=10116 GN=Dpy30 PE=1 SV=1 - [DPY30_RAT]	11.2	0.013	0.013	0.013	0.013	0.013	0.013	0.013	0.013	0.013	0.013	2.500	1.864	4.595	2.161	1.383	0.013	0.013	0.013	0.013	0.013	#DIV/0!	#DIV/0!	0.009	0.028	#DIV/0!	#DIV/0!	0.009	0.022	#DIV/0!	#DIV/0!	0.009	0.062
Q8K3P7	Histidine triad nucleotide-binding protein 3 OS=Rattus norvegicus OX=10116 GN=Hint3 PE=2 SV=2 - [HINT3_RAT]	19.7	0.013	0.013	0.013	0.013	0.013	0.013	0.013	0.013	0.013	0.013	0.827	0.811	1.320	1.938	0.593	0.013	0.013	0.013	0.013	0.013	#DIV/0!	#DIV/0!	0.009	0.093	#DIV/0!	#DIV/0!	0.009	0.082	#DIV/0!	#DIV/0!	0.009	0.212
Q8K3X8	Heat shock factor-binding protein 1 OS=Rattus norvegicus OX=10116 GN=Hsbp1 PE=3 SV=1 - [HSBP1_RAT]	8.6	0.013	0.013	0.013	0.013	0.013	0.013	0.013	0.013	0.013	0.013	1.381	1.483	2.411	2.969	1.192	0.013	0.013	0.013	0.013	0.013	#DIV/0!	#DIV/0!	0.009	0.034	#DIV/0!	#DIV/0!	0.009	0.027	#DIV/0!	#DIV/0!	0.009	0.072
Q8K4M9	Oxysterol-binding protein-related protein 1 OS=Rattus norvegicus OX=10116 GN=Osbpl1a PE=1 SV=1 - [OSBL1_RAT]	107.7	0.424	1.518	0.351	0.956	0.943	0.923	0.446	0.435	1.278	1.288	1.524	1.663	2.973	2.300	1.341	1.944	0.859	1.763	1.795	1.506	0.756	0.975	0.022	0.039	0.085	0.119	0.014	0.025	0.056	0.074	0.605	1.309
Q8VHT6	Arsenite methyltransferase OS=Rattus norvegicus OX=10116 GN=As3mt PE=1 SV=1 - [AS3MT_RAT]	41.0	0.676	1.130	0.433	1.172	1.143	1.125	0.532	0.591	1.014	1.043	1.259	1.202	2.456	1.723	1.247	1.641	0.521	1.570	1.732	1.328	0.354	0.926	0.014	0.031	0.085	0.122	0.014	0.028	0.127	0.126	0.917	1.070
Q91ZW6	Trimethyllysine dioxygenase, mitochondrial OS=Rattus norvegicus OX=10116 GN=Tmlhe PE=1 SV=2 - [TMLH_RAT]	49.5	0.482	0.641	0.368	0.643	0.660	0.669	0.361	0.431	0.691	0.743	0.684	0.753	1.221	5.278	0.770	4.658	0.624	1.063	1.025	0.846	0.470	1.131	0.014	0.035	0.056	0.101	0.036	0.053	0.056	0.081	0.917	0.980
Q920A6	Retinoid-inducible serine carboxypeptidase OS=Rattus norvegicus GN=Scpep1 PE=2 SV=1 - [RISC_RAT]	51.1	0.382	0.692	0.242	0.731	0.716	0.589	0.286	0.410	0.783	0.841	0.833	0.932	1.548	7.354	0.754	4.473	0.478	1.119	1.240	1.164	0.605	1.142	0.014	0.028	0.056	0.097	0.056	0.064	0.056	0.077	0.917	0.928
Q99NA5	Isocitrate dehydrogenase [NAD] subunit alpha, mitochondrial OS=Rattus norvegicus GN=Idh3a PE=1 SV=1 - [IDH3A_RAT]	39.6	0.728	0.725	0.589	0.913	0.875	1.076	0.912	1.020	0.570	0.564	0.799	0.937	1.401	3.948	0.920	2.957	0.706	1.114	1.166	0.732	0.756	1.058	0.036	0.058	0.184	0.220	0.259	0.207	0.184	0.164	0.605	1.414
Q9EPF2	Cell surface glycoprotein MUC18 OS=Rattus norvegicus OX=10116 GN=Mcam PE=1 SV=2 - [MUC18_RAT]	71.3	0.013	0.013	0.013	0.013	0.013	0.900	0.225	0.510	1.176	1.125	1.036	1.155	2.105	2.461	1.122	1.781	0.634	1.518	1.247	1.186	0.009	0.229	0.009	0.048	0.009	0.059	0.127	0.117	0.056	0.095	0.917	1.094
Q9EQP5	Prolargin OS=Rattus norvegicus GN=Prelp PE=2 SV=1 - [PRELP_RAT]	43.2	0.433	0.856	0.401	1.182	0.925	0.608	0.331	0.457	0.809	0.778	1.176	1.217	2.452	2.861	1.287	3.556	0.473	1.914	1.746	1.374	0.354	1.074	0.022	0.040	0.056	0.089	0.014	0.029	0.056	0.074	0.756	1.126
Q9ES40	PRA1 family protein 3 OS=Rattus norvegicus OX=10116 GN=Arl6ip5 PE=1 SV=1 - [PRAF3_RAT]	21.5	0.013	0.013	0.013	0.013	0.013	1.752	0.658	0.556	0.803	0.733	1.508	1.564	2.569	1.332	1.023	1.665	0.564	1.871	1.721	0.790	0.009	0.089	0.009	0.046	0.009	0.056	0.085	0.086	0.354	0.276	0.917	1.075
Q9ESH6	Glutaredoxin-1 OS=Rattus norvegicus GN=Glrx PE=3 SV=3 - [GLRX1_RAT]	11.9	0.658	1.003	0.535	1.252	1.134	1.132	0.423	0.588	1.069	1.116	1.252	1.176	2.478	1.527	1.296	1.046	0.764	1.761	1.552	1.850	0.605	0.961	0.036	0.056	0.127	0.162	0.014	0.028	0.184	0.162	0.917	1.070
Q9ESN0	Protein Niban OS=Rattus norvegicus GN=Fam129a PE=1 SV=2 - [NIBAN_RAT]	103.4	1.036	1.089	0.716	0.982	0.845	1.934	0.612	0.702	0.841	0.678	1.186	1.208	1.907	1.013	1.140	1.121	0.872	1.935	1.421	0.669	0.184	0.709	0.036	0.059	0.354	0.354	0.127	0.119	0.259	0.214	0.605	1.569
Q9JHB5	Translin-associated protein X OS=Rattus norvegicus GN=Tsnax PE=1 SV=1 - [TSNAX_RAT]	33.0	0.013	0.013	0.013	0.013	0.013	1.304	0.917	0.775	0.948	1.008	0.744	0.889	1.677	1.012	1.032	1.338	0.875	1.362	0.958	1.516	0.009	0.063	0.009	0.0995	0.009	0.065	0.756	0.551	0.184	0.163	0.605	1.673
Q9JHW0	Proteasome subunit beta type-7 OS=Rattus norvegicus GN=Psmb7 PE=1 SV=1 - [PSB7_RAT]	29.9	0.692	0.866	0.722	0.844	0.795	1.110	0.508	0.714	1.324	1.130	0.992	1.068	1.774	1.507	1.232	1.562	1.110	1.191	1.336	1.084	0.470	0.994	0.014	0.039	0.014	0.040	0.259	0.208	0.259	0.213	0.917	1.197
Q9JID1	Programmed cell death protein 4 OS=Rattus norvegicus GN=Pdcd4 PE=1 SV=2 - [PDCD4_RAT]	51.7	0.805	0.949	0.593	0.973	1.049	0.765	0.282	0.427	1.048	1.131	2.793	1.605	2.741	1.886	0.983	1.522	0.607	3.005	2.623	1.007	0.605	1.044	0.022	0.039	0.127	0.157	0.036	0.050	0.127	0.122	0.605	1.301
Q9JIX3	Bis(5'-adenosyl)-triphosphatase OS=Rattus norvegicus OX=10116 GN=Fhit PE=1 SV=1 - [FHIT_RAT]	17.3	0.013	0.013	0.013	0.013	0.013	1.132	0.559	0.768	1.140	1.003	0.931	0.998	1.856	1.720	0.813	2.034	1.054	1.293	1.034	1.197	0.009	0.076	0.009	0.069	0.009	0.056	0.470	0.354	0.085	0.097	0.354	1.537
Q9JJ31	Cullin-5 OS=Rattus norvegicus GN=Cul5 PE=1 SV=3 - [CUL5_RAT]	90.8	0.013	0.013	0.013	0.013	0.013	1.379	0.633	0.742	1.091	0.705	2.256	2.549	2.759	2.633	2.102	1.556	0.747	1.903	1.885	0.982	0.009	0.084	0.009	0.028	0.009	0.047	0.014	0.025	0.085	0.095	0.014	0.091
Q9JJP9	Ubiquilin-1 OS=Rattus norvegicus GN=Ubqln1 PE=1 SV=1 - [UBQL1_RAT]	62.0	0.574	0.961	0.526	1.069	1.009	0.860	0.510	0.592	1.054	1.016	1.691	1.400	3.341	1.777	1.493	1.803	0.533	1.713	2.002	1.767	0.917	0.997	0.014	0.028	0.085	0.119	0.014	0.025	0.085	0.094	0.756	1.139
Q9JLA3	UDP-glucose:glycoprotein glucosyltransferase 1 OS=Rattus norvegicus GN=Uggt1 PE=1 SV=2 - [UGGG1_RAT]	176.3	0.701	1.065	0.579	1.032	1.007	1.231	0.455	0.643	1.038	1.033	1.092	1.203	2.242	1.016	1.388	0.973	0.922	1.541	1.319	1.287	0.756	0.950	0.036	0.059	0.184	0.220	0.127	0.118	0.184	0.167	0.605	1.520
Q9JLJ3	4-trimethylaminobutyraldehyde dehydrogenase OS=Rattus norvegicus GN=Aldh9a1 PE=1 SV=1 - [AL9A1_RAT]	53.6	0.618	0.846	0.559	0.896	0.843	1.072	0.520	0.733	0.819	0.918	0.875	0.981	1.783	3.037	0.974	2.583	0.840	1.153	1.043	0.995	0.756	1.066	0.022	0.043	0.056	0.116	0.085	0.088	0.085	0.10004	0.917	1.091
Q9JLZ1	Glutaredoxin-3 OS=Rattus norvegicus GN=Glrx3 PE=1 SV=2 - [GLRX3_RAT]	37.8	0.726	0.925	0.650	0.947	0.923	1.004	0.588	0.802	0.902	0.942	1.415	1.555	2.877	1.825	1.323	1.917	0.632	1.873	1.524	1.701	0.917	0.981	0.014	0.029	0.127	0.160	0.014	0.026	0.085	0.093	0.917	0.988
Q9JM53	Apoptosis-inducing factor 1, mitochondrial OS=Rattus norvegicus GN=Aifm1 PE=1 SV=1 - [AIFM1_RAT]	66.7	0.694	0.811	0.558	0.761	0.613	1.182	0.474	0.650	0.654	0.762	1.101	1.108	1.964	3.667	1.045	2.931	0.593	1.458	1.249	0.949	0.917	1.055	0.014	0.031	0.085	0.128	0.056	0.065	0.127	0.127	0.605	1.355
Q9QZK8	Deoxyribonuclease-2-alpha OS=Rattus norvegicus GN=Dnase2 PE=2 SV=1 - [DNS2A_RAT]	38.2	0.013	0.013	0.013	0.013	0.013	0.013	0.013	0.013	0.013	0.013	0.749	0.784	1.547	2.188	1.041	0.013	0.013	0.013	0.013	0.013	#DIV/0!	#DIV/0!	0.009	0.070	#DIV/0!	#DIV/0!	0.009	0.061	#DIV/0!	#DIV/0!	0.009	0.139
Q9QZT0	CUB and zona pellucida-like domain-containing protein 1 OS=Rattus norvegicus GN=Cuzd1 PE=2 SV=2 - [CUZD1_RAT]	68.6	0.665	0.966	0.549	0.887	0.753	1.071	0.462	0.548	1.237	0.975	1.016	0.991	1.890	0.892	1.302	1.060	0.950	1.543	0.999	0.873	0.605	1.033	0.022	0.045	0.056	0.128	0.259	0.212	0.605	0.445	0.605	1.664
Q9R085	Ubiquitin carboxyl-terminal hydrolase 15 OS=Rattus norvegicus GN=Usp15 PE=1 SV=1 - [UBP15_RAT]	109.2	0.013	0.013	0.013	0.013	0.013	1.255	0.741	0.757	0.768	0.778	2.043	1.659	3.940	2.598	1.893	1.864	0.792	1.248	1.211	1.180	0.009	0.113	0.009	0.028	0.009	0.060	0.014	0.025	0.085	0.101	0.022	0.146
Q9R0D6	Transcobalamin-2 OS=Rattus norvegicus GN=Tcn2 PE=2 SV=1 - [TCO2_RAT]	47.4	0.013	0.013	0.013	0.013	0.013	0.704	0.358	0.589	1.048	1.284	1.026	0.864	1.828	4.729	1.035	2.690	0.418	1.351	1.251	0.935	0.009	0.216	0.009	0.034	0.009	0.055	0.184	0.153	0.259	0.214	0.756	1.181
Q9R0J8	Legumain OS=Rattus norvegicus GN=Lgmn PE=1 SV=1 - [LGMN_RAT]	49.4	0.213	0.660	0.235	0.747	0.639	0.974	0.500	0.564	0.746	0.809	1.004	0.837	1.608	3.708	0.910	3.028	0.696	1.014	0.915	0.945	0.259	0.956	0.014	0.039	0.022	0.062	0.036	0.053	0.127	0.131	0.756	1.256
Q9WUC4	Copper transport protein ATOX1 OS=Rattus norvegicus GN=Atox1 PE=1 SV=1 - [ATOX1_RAT]	7.3	0.013	0.013	0.013	0.013	0.013	0.013	0.013	0.013	0.013	0.013	0.872	1.536	1.630	1.260	1.399	0.013	0.013	0.013	0.013	0.013	#DIV/0!	#DIV/0!	0.009	0.062	#DIV/0!	#DIV/0!	0.009	0.054	#DIV/0!	#DIV/0!	0.009	0.116
Q9WUW3	Complement factor I OS=Rattus norvegicus GN=Cfi PE=2 SV=1 - [CFAI_RAT]	67.3	0.013	0.013	0.013	0.013	0.013	0.978	0.620	0.963	0.878	0.821	0.824	0.835	1.670	1.812	0.934	1.628	0.930	1.043	0.939	0.774	0.009	0.120	0.009	0.074	0.009	0.087	0.354	0.278	0.354	0.282	0.756	1.511
Q9WV59	E3 ubiquitin-protein ligase TRIM17 OS=Rattus norvegicus GN=Trim17 PE=2 SV=1 - [TRI17_RAT]	54.9	2.344	0.596	1.581	1.056	0.803	0.013	0.013	0.013	0.013	0.013	0.013	0.013	0.013	0.013	0.013	0.013	0.013	0.013	0.013	0.013	0.009	0.052	0.009	0.066	0.009	0.059	#DIV/0!	#DIV/0!	#DIV/0!	#DIV/0!	#DIV/0!	#DIV/0!

Q9WVC0	Septin-7 OS=Rattus norvegicus GN=Sept7 PE=1 SV=1 - [SEPT7_RAT]	50.5	0.832	0.792	0.780	0.972	0.804	1.208	0.697	0.855	0.676	0.708	0.881	1.036	2.037	1.791	1.056	1.665	0.909	1.368	1.108	0.957	0.470	1.040	0.022	0.043	0.036	0.088	0.056	0.073	0.056	0.097	0.756	1.399
Q9WVK7	Hydroxyacyl-coenzyme A dehydrogenase, mitochondrial OS=Rattus norvegicus GN=Hadh PE=2 SV=1 - [HCDH_RAT]	34.4	0.551	0.705	0.547	0.785	0.727	0.859	0.430	0.575	0.809	0.902	0.997	1.123	2.129	4.094	0.926	3.045	0.658	1.303	1.234	1.257	0.354	1.069	0.014	0.030	0.056	0.105	0.014	0.026	0.056	0.081	0.917	0.983
Q9WVR7	Protein phosphatase 1F OS=Rattus norvegicus GN=Ppm1f PE=2 SV=1 - [PPM1F_RAT]	49.1	0.013	0.013	0.013	0.013	0.013	0.788	0.779	0.653	1.000	0.796	0.983	1.069	2.210	1.584	1.242	1.510	0.558	1.844	1.708	1.214	0.009	0.192	0.009	0.057	0.009	0.052	0.022	0.040	0.127	0.127	0.917	1.115
Q9Z0V6	Thioredoxin-dependent peroxide reductase, mitochondrial OS=Rattus norvegicus GN=Prdx3 PE=1 SV=2 - [PRDX3_RAT]	28.3	0.431	1.018	0.450	1.076	0.945	0.953	0.390	0.663	1.079	0.856	1.101	1.111	1.990	2.753	1.228	2.346	0.549	1.412	1.312	1.102	0.917	1.018	0.014	0.032	0.056	0.110	0.014	0.028	0.085	0.1002	0.756	1.241
Q9Z0W7	Chloride intracellular channel protein 4 OS=Rattus norvegicus GN=Clic4 PE=1 SV=3 - [CLIC4_RAT]	28.6	0.673	0.826	0.411	0.855	0.774	1.053	0.480	0.616	0.806	0.695	0.959	1.093	1.721	3.856	0.992	3.538	0.583	1.396	1.280	1.098	0.917	1.054	0.014	0.032	0.085	0.122	0.036	0.051	0.085	0.095	0.917	0.996
Q9Z1N4	3'(2'),5'-bisphosphate nucleotidase 1 OS=Rattus norvegicus GN=Bpnt1 PE=1 SV=1 - [BPNT1_RAT]	33.2	0.726	0.844	0.502	0.960	0.946	0.971	0.476	0.631	0.823	0.873	1.155	1.193	2.201	3.433	1.034	2.890	0.660	1.119	1.067	1.041	0.756	1.072	0.014	0.030	0.085	0.126	0.014	0.026	0.056	0.088	0.259	1.277
Q9Z1P2	Alpha-actinin-1 OS=Rattus norvegicus GN=Actn1 PE=1 SV=1 - [ACTN1_RAT]	102.9	0.506	0.901	0.486	0.797	0.787	1.002	0.443	0.588	0.952	1.009	0.961	1.111	2.200	1.301	1.358	1.412	0.939	1.421	1.264	1.296	0.354	1.040	0.014	0.039	0.014	0.041	0.036	0.055	0.056	0.094	0.917	1.164
Q9Z2G8	Nucleosome assembly protein 1-like 1 OS=Rattus norvegicus GN=Nap1l1 PE=2 SV=1 - [NP1L1_RAT]	45.3	0.378	1.178	0.347	1.248	0.885	0.937	0.361	0.481	0.955	1.126	1.503	1.213	2.860	1.855	1.347	2.051	0.295	1.873	1.892	1.559	0.917	1.014	0.022	0.040	0.127	0.161	0.014	0.027	0.127	0.124	0.605	1.343
Q9Z339	Glutathione S-transferase omega-1 OS=Rattus norvegicus GN=Gsto1 PE=1 SV=2 - [GSTO1_RAT]	27.7	0.567	0.938	0.501	1.027	0.922	1.079	0.444	0.600	1.080	1.101	0.987	1.104	2.207	2.388	1.086	2.111	0.700	1.407	1.158	1.087	0.354	0.990	0.022	0.042	0.056	0.115	0.085	0.087	0.085	0.099	0.756	1.287

Table S5. Extracted protein profile of b-III (Effect of FGR, only Poor nutrition, Male)

Accession	Description	MW [kDa]	Expression level																				p<0.05, q<0.10											
			FGR-Good-Male					FGR-Poor-Male					Sham-Good-Male					Sham-Poor-Male					Steel-Dwass & FDR											
			#21	#22	#23	#24	#25	#26	#27	#28	#29	#30	#31	#32	#33	#34	#35	#36	#37	#38	#39	#40	FGR-GN vs FGR-PN		FGR-GN vs Sham-GN		FGR-GN vs Sham-PN		FGR-PN vs Sham-GN		FGR-PN vs Sham-PN		Sham-GN vs Sham-PN	
Filter	Filter	Filter	Filter	Filter	Filter	Filter	Filter	Filter	Filter	Filter	Filter	Filter	Filter	Filter	Filter	Filter	Filter	Filter	Filter	Filter	Filter	Filter	p-value	q-value	p	q-value	p	q-value	p	q-value	p	q-value	p	q-value
A0JPM9	Eukaryotic translation initiation factor 3 subunit J OS=Rattus norvegicus OX=10116 GN=Eif3j PE=1 SV=1 - [EIF3J_RAT]	29.2	0.852	0.817	1.827	0.715	0.795	0.919	0.923	1.260	0.247	0.613	0.960	0.989	0.953	1.796	0.562	0.013	0.013	0.013	0.013	0.013	0.917	0.563	0.470	0.511	0.009	0.049	0.259	0.535	0.009	0.032	0.009	0.040
A0JPQ1	Uncharacterized protein C7orf57 homolog OS=Rattus norvegicus OX=10116 PE=2 SV=1 - [CG057_RAT]	32.7	0.013	0.013	0.013	0.013	0.013	0.013	0.013	0.013	0.013	0.013	0.013	0.013	0.013	0.013	0.013	0.591	1.285	1.592	1.210	0.821	#DIV/0!	#DIV/0!	#DIV/0!	#DIV/0!	0.009	0.045	#DIV/0!	#DIV/0!	0.009	0.016	0.009	0.037
A2VD13	Pentatricopeptide repeat-containing protein 1, mitochondrial OS=Rattus norvegicus OX=10116 GN=Ptcd1 PE=2 SV=1 - [PTCD1_RAT]	77.1	0.013	0.013	0.013	0.013	0.013	0.854	1.019	1.007	0.571	1.088	0.013	0.013	0.013	0.013	0.013	0.013	0.013	0.013	0.013	0.013	0.009	0.0319	#DIV/0!	#DIV/0!	#DIV/0!	#DIV/0!	0.009	0.061	0.009	0.022	#DIV/0!	#DIV/0!
B0BNF1	Septin-8 OS=Rattus norvegicus GN=Sept8 PE=1 SV=1 - [SEPT8_RAT]	51.2	0.013	0.013	0.013	0.013	0.013	0.818	1.266	1.312	0.179	1.303	0.013	0.013	0.013	0.013	0.013	0.013	0.013	0.013	0.013	0.013	0.009	0.0270	#DIV/0!	#DIV/0!	#DIV/0!	#DIV/0!	0.009	0.049	0.009	0.019	#DIV/0!	#DIV/0!
B0K019	BAG family molecular chaperone regulator 1 OS=Rattus norvegicus OX=10116 GN=Bag1 PE=2 SV=1 - [BAG1_RAT]	40.1	0.808	0.593	1.102	0.207	0.509	0.013	0.013	0.013	0.013	0.013	0.629	0.835	0.864	1.619	0.136	0.534	0.650	0.659	0.539	0.771	0.009	0.145	0.470	0.575	0.917	0.975	0.009	0.093	0.009	0.089	0.354	0.855
B0LPN4	Ryanodine receptor 2 OS=Rattus norvegicus GN=Ryr2 PE=1 SV=2 - [RYR2_RAT]	562.6	0.013	0.013	0.013	0.013	0.013	0.928	0.756	0.953	2.236	0.937	0.013	0.013	0.013	0.013	0.013	0.013	0.013	0.013	0.013	0.013	0.009	0.0225	#DIV/0!	#DIV/0!	#DIV/0!	#DIV/0!	0.009	0.039	0.009	0.016	#DIV/0!	#DIV/0!
B2GUY0	Endoplasmic reticulum mannosyl-oligosaccharide 1,2-alpha-mannosidase OS=Rattus norvegicus OX=10116 GN=Man1b1 PE=2 SV=2 - [MA1B1_RAT]	74.8	0.013	0.013	0.013	0.013	0.013	0.013	0.013	0.013	0.013	0.013	0.013	0.013	0.013	0.013	0.013	0.785	0.893	1.915	0.737	1.012	#DIV/0!	#DIV/0!	#DIV/0!	#DIV/0!	0.009	0.046	#DIV/0!	#DIV/0!	0.009	0.017	0.009	0.039
B2RYG6	Ubiquitin thioesterase OTUB1 OS=Rattus norvegicus GN=Otub1 PE=1 SV=1 - [OTUB1_RAT]	31.3	0.852	0.778	1.493	0.504	0.541	0.806	0.927	1.135	0.134	0.734	0.621	0.945	1.105	1.305	0.296	0.013	0.013	0.013	0.013	0.013	0.917	0.589	0.756	0.621	0.009	0.069	0.605	0.771	0.009	0.041	0.009	0.075
B5DF27	Lysyl oxidase homolog 2 OS=Rattus norvegicus GN=Loxl2 PE=2 SV=2 - [LOXL2_RAT]	87.1	0.013	0.013	0.013	0.013	0.013	0.013	0.013	0.013	0.013	0.013	0.013	0.013	0.013	0.013	0.013	0.704	1.688	1.327	1.029	1.295	#DIV/0!	#DIV/0!	#DIV/0!	#DIV/0!	0.009	0.042	#DIV/0!	#DIV/0!	0.009	0.016	0.009	0.035
B5DF91	ELAV-like protein 1 OS=Rattus norvegicus OX=10116 GN=Elavl1 PE=1 SV=1 - [ELAV1_RAT]	36.1	0.013	0.013	0.013	0.013	0.013	0.694	0.689	1.015	0.291	0.514	0.013	0.013	0.013	0.013	0.013	0.013	0.013	0.013	0.013	0.013	0.009	0.152	#DIV/0!	#DIV/0!	#DIV/0!	#DIV/0!	0.009	0.372	0.009	0.082	#DIV/0!	#DIV/0!
B5DFM7	Cation channel sperm-associated protein subunit delta OS=Rattus norvegicus OX=10116 GN=Catsperd PE=2 SV=1 - [CTSRD_RAT]	80.6	0.013	0.013	0.013	0.013	0.013	0.718	0.397	0.492	1.467	0.272	0.013	0.013	0.013	0.013	0.013	0.013	0.013	0.013	0.013	0.013	0.009	0.120	#DIV/0!	#DIV/0!	#DIV/0!	#DIV/0!	0.009	0.305	0.009	0.070	#DIV/0!	#DIV/0!
C9WPN6	Eukaryotic translation initiation factor 2 subunit 3, Y-linked OS=Rattus norvegicus OX=10116 GN=Eif2s3y PE=2 SV=2 - [IF2H_RAT]	51.1	0.013	0.013	0.013	0.013	0.013	0.953	0.896	1.790	0.050	0.799	0.013	0.013	0.013	0.013	0.013	0.013	0.013	0.013	0.013	0.013	0.009	0.0328	#DIV/0!	#DIV/0!	#DIV/0!	#DIV/0!	0.009	0.065	0.009	0.023	#DIV/0!	#DIV/0!
D3ZHV2	Microtubule-actin cross-linking factor 1 OS=Rattus norvegicus GN=Macf1 PE=1 SV=1 - [MACF1_RAT]	619.2	0.013	0.013	0.013	0.013	0.013	0.922	0.850	1.154	0.539	0.521	0.013	0.013	0.013	0.013	0.013	0.013	0.013	0.013	0.013	0.013	0.009	0.0500	#DIV/0!	#DIV/0!	#DIV/0!	#DIV/0!	0.009	0.103	0.009	0.032	#DIV/0!	#DIV/0!
D3ZQL7	Tubulin polymerization-promoting protein OS=Rattus norvegicus OX=10116 GN=Tppp PE=1 SV=1 - [TPPP_RAT]	23.5	0.635	0.506	1.362	0.500	0.508	0.866	0.896	1.162	0.233	0.848	0.594	0.595	0.823	1.539	0.240	0.013	0.013	0.013	0.013	0.013	0.470	0.489	0.605	0.637	0.009	0.117	0.470	0.742	0.009	0.031	0.009	0.138
D3ZVU1	SprT-like domain-containing protein Spartan OS=Rattus norvegicus OX=10116 GN=Sprtn PE=3 SV=1 - [SPRTN_RAT]	55.0	0.013	0.013	0.013	0.013	0.013	0.656	1.215	0.861	0.129	0.790	0.013	0.013	0.013	0.013	0.013	0.013	0.013	0.013	0.013	0.013	0.009	0.0701	#DIV/0!	#DIV/0!	#DIV/0!	#DIV/0!	0.009	0.168	0.009	0.046	#DIV/0!	#DIV/0!
D4A4T9	Cysteine and histidine-rich domain-containing protein 1 OS=Rattus norvegicus GN=Chordc1 PE=3 SV=1 - [CHRD1_RAT]	37.3	0.674	0.627	1.282	0.255	0.495	0.013	0.013	0.013	0.013	0.013	0.013	0.013	0.013	0.013	0.013	0.493	0.577	0.892	0.628	0.862	0.009	0.127	0.009	0.264	0.756	0.956	#DIV/0!	#DIV/0!	0.009	0.059	0.009	0.252
F1LNI5	Protein phosphatase 1G OS=Rattus norvegicus GN=Ppm1g PE=1 SV=2 - [PPM1G_RAT]	58.7	1.037	1.095	1.257	0.441	0.418	0.918	1.074	1.452	0.233	1.118	0.525	0.907	1.072	1.736	0.248	0.013	0.013	0.013	0.013	0.013	0.756	0.520	0.917	0.640	0.009	0.065	0.605	0.683	0.009	0.019	0.009	0.062
F1M775	Protein diaphanous homolog 1 OS=Rattus norvegicus OX=10116 GN=Diaph1 PE=1 SV=3 - [DIAP1_RAT]	140.3	0.013	0.013	0.013	0.013	0.013	0.610	0.957	1.206	0.223	0.770	0.601	0.943	0.811	1.134	0.237	0.013	0.013	0.013	0.013	0.013	0.009	0.0598	0.009	0.127	#DIV/0!	#DIV/0!	0.917	0.820	0.009	0.039	0.009	0.151
F1MA98	Nucleoprotein TPR OS=Rattus norvegicus GN=Tpr PE=1 SV=1 - [TPR_RAT]	267.1	0.374	0.474	1.815	0.605	0.588	0.714	0.777	0.762	0.151	0.556	0.841	0.960	1.024	1.332	0.555	0.817	0.953	1.599	0.945	1.183	0.756	0.575	0.259	0.428	0.127	0.420	0.085	0.289	0.014	0.022	0.756	0.722
O08701	Arginase-2, mitochondrial OS=Rattus norvegicus OX=10116 GN=Arg2 PE=1 SV=1 - [ARGI2_RAT]	38.6	0.013	0.013	0.013	0.013	0.013	0.836	1.011	1.228	0.482	0.909	0.013	0.013	0.013	0.013	0.013	0.275	0.407	0.589	0.438	0.504	0.009	0.0335	#DIV/0!	#DIV/0!	0.009	0.796	0.009	0.066	0.036	0.053	0.009	1.073
O08815	STE20-like serine/threonine-protein kinase OS=Rattus norvegicus GN=Slk PE=2 SV=1 - [SLK_RAT]	137.8	0.013	0.013	0.013	0.013	0.013	0.676	1.062	1.280	0.355	0.815	0.013	0.013	0.013	0.013	0.013	0.013	0.013	0.013	0.013	0.013	0.009	0.0401	#DIV/0!	#DIV/0!	#DIV/0!	#DIV/0!	0.009	0.085	0.009	0.027	#DIV/0!	#DIV/0!
O35331	Pyridoxal kinase OS=Rattus norvegicus GN=Pdxk PE=1 SV=1 - [PDXK_RAT]	34.9	0.911	0.602	0.820	0.729	0.451	0.781	1.097	1.116	0.650	0.949	0.502	0.705	0.739	1.162	0.273	0.293	0.597	0.628	0.517	0.629	0.127	0.229	0.756	0.669	0.184	0.585	0.259	0.597	0.014	0.022	0.470	0.959
O55012	Phosphatidylinositol-binding clathrin assembly protein OS=Rattus norvegicus GN=Picalm PE=1 SV=1 - [PICAL_RAT]	69.2	0.013	0.013	0.013	0.013	0.013	0.473	0.820	1.084	0.188	0.856	0.138	0.444	0.848	1.254	0.061	0.013	0.013	0.013	0.013	0.013	0.009	0.103	0.009	0.475	#DIV/0!	#DIV/0!	0.470	0.769	0.009	0.063	0.009	0.613
O55159	Epithelial cell adhesion molecule OS=Rattus norvegicus OX=10116 GN=Epcam PE=1 SV=1 - [EPCAM_RAT]	35.2	0.013	0.013	0.013	0.013	0.013	0.713	1.324	0.840	0.164	1.608	0.013	0.013	0.013	0.013	0.013	0.013	0.013	0.013	0.013	0.013	0.009	0.0303	#DIV/0!	#DIV/0!	#DIV/0!	#DIV/0!	0.009	0.057	0.009	0.021	#DIV/0!	#DIV/0!
O55165	Kinesin-like protein KIF3C OS=Rattus norvegicus OX=10116 GN=Kif3c PE=2 SV=1 - [KIF3C_RAT]	89.8	0.013	0.013	0.013	0.013	0.013	0.013	0.013	0.013	0.013	0.013	0.013	0.013	0.013	0.013	0.013	0.503	0.663	1.080	0.749	0.887	#DIV/0!	#DIV/0!	#DIV/0!	#DIV/0!	0.009	0.084	#DIV/0!	#DIV/0!	0.009	0.034	0.009	0.123
O70260	E3 SUMO-protein ligase PIAS3 OS=Rattus norvegicus OX=10116 GN=Pias3 PE=1 SV=2 - [PIAS3_RAT]	68.3	0.013	0.013	0.013	0.013	0.013	0.013	0.013	0.013	0.013	0.013	0.013	0.013	0.013	0.013	0.013	0.536	2.259	1.706	1.830	0.655	#DIV/0!	#DIV/0!	#DIV/0!	#DIV/0!	0.009	0.041	#DIV/0!	#DIV/0!	0.009	0.015	0.009	0.034
O88350	Putative hydrolase RBBP9 OS=Rattus norvegicus GN=Rbbp9 PE=1 SV=2 - [RBBP9_RAT]	21.0	0.794	0.569	1.496	0.402	0.504	1.193	1.307	1.539	0.834	1.147	0.279	0.634	0.896	1.390	0.125	0.397	0.630	0.646	0.505	0.760	0.085	0.167	0.756	0.666	0.756	0.958	0.127	0.378	0.014	0.021	0.917	0.855
O88794	Pyridoxine-5'-phosphate oxidase OS=Rattus norvegicus OX=10116 GN=Pnpo PE=1 SV=1 - [PNPO_RAT]	30.2	1.351	0.956	1.781	0.835	0.759	0.612	0.701	0.877	0.175	0.621	0.829	1.228	0.880	1.534	0.824	0.653	1.348	1.428	1.096	0.939	0.036	0.0782	0.756	0.581	0.917	0.838	0.036	0.132	0.036	0.051	0.756	0.718
O88941	Mannosyl-oligosaccharide glucosidase OS=Rattus norvegicus OX=10116 GN=Mogs PE=1 SV=1 - [MOGS_RAT]	91.8	0.013	0.013	0.013	0.013	0.013	0.946	1.589	1.421	0.214	1.445	0.517	0.835	1.030	1.277	0.190	0.013	0.013	0.013	0.013	0.013	0.009	0.0228	0.009	0.103	#DIV/0!	#DIV/0!	0.184	0.459	0.009	0.016	0.009	0.126
P00388	NADPH--cytochrome P450 reductase OS=Rattus norvegicus OX=10116 GN=Por PE=1 SV=3 - [NCPR_RAT]	76.9	0.013	0.013	0.013	0.013	0.013	0.935	1.038	1.137	0.444	0.823	0.013	0.013	0.013	0.013	0.013	0.013	0.013	0.013	0.013	0.013	0.009	0.0346	#DIV/0!	#DIV/0!	#DIV/0!	#DIV/0!	0.009	0.071	0.009	0.024	#DIV/0!	#DIV/0!
P00507	Aspartate aminotransferase, mitochondrial OS=Rattus norvegicus GN=Got2 PE=1 SV=2 - [AATM_RAT]	47.3	0.644	0.689	1.054	0.469	0.477	0.766	1.036	0.990	0.559	0.831	0.519	0.796	0.859	0.905	0.272	0.315	0.552	0.663	0.492	0.722	0.259	0.368	0.756	0.673	0.756	0.978	0.354	0.675	0.036	0.052	0.354	0.891
P00762	Anionic trypsin-1 OS=Rattus norvegicus GN=Prss1 PE=1 SV=1 - [TRY1_RAT]	25.9	0.930	0.620	0.914	0.371	0.405	0.948	1.175	1.029	0.947	1.111	0.597	0.928	0.944	1.462	0.186	0.473	0.746	0.701	0.463	0.877	0.014	0.0321	0.470	0.574	0.917	0.968	0.127	0.379	0.014	0.022	0.354	0.851
P00774	Chymotrypsin-like elastase family member 2A OS=Rattus norvegicus OX=10116 GN=Cela2a PE=2 SV=1 - [CEL2A_RAT]	28.9	1.066	0.767	0.746	0.427	0.416	1.121	1.257	1.239	0.973	1.305	0.736	0.849	0.774	1.400	0.244	0.499	0.919	0.895	0.549	0.912	0.022	0.0508	0.605	0.630	0.470	0.862	0.127	0.374	0.014	0.021	0.756	0.820
P01026	Complement C3 OS=Rattus norvegicus GN=C3 PE=1 SV=3 - [CO3_RAT]	186.3	1.282	0.839	1.735	0.877	0.549	0.968	1.212	0.937	1.243	1.229	0.491	0.876	0.814	0.723	0.358	0.322	1.090	0.824	0.792	0.796	0.605	0.467	0.085	0.199	0.184	0.524	0.014	0.054	0.036	0.051	0.605	0.858
P02564	Myosin-7 OS=Rattus norvegicus GN=Myh7 PE=1 SV=2 - [MYH7_RAT]	222.9	0.013	0.013	0.013	0.013	0.013	0.013	0.013	0.013	0.013	0.013	0.013	0.013	0.013	0.013	0.013	1.008	1.981	1.973	1.650	1.093	#DIV/0!	#DIV/0!	#DIV/0!	#DIV/0!	0.009	0.041	#DIV/0!	#DIV/0!	0.009	0.015	0.009	0.034
P02692	Fatty acid-binding protein, liver OS=Rattus norvegicus OX=10116 GN=Fabp1 PE=1 SV=1 - [FABPL_RAT]	14.3	0.013	0.013	0.013	0.013	0.013	0.727	1.155	0.916	3.192	0.872	0.013	0.013	0.013	0.013	0.013	0.013	0.013	0.013	0.013	0.013	0.009	0.0219	#DIV/0!	#DIV/0!	#DIV/0!	#DIV/0!	0.009	0.037	0.009	0.015	#DIV/0!	#DIV/0!
P02764	Alpha-1-acid glycoprotein OS=Rattus norvegicus GN=Orm1 PE=2 SV=1 - [A1AG_RAT]	23.6	0.013	0.013	0.013	0.013	0.013	0.963	1.049	1.079	1.126	0.943	0.013	0.013	0.013	0.013	0.013	0.013	0.013	0.013	0.013	0.013	0.009	0.0248	#DIV/0!	#DIV/0!	#DIV/0!	#DIV/0!	0.009	0.045	0.009	0.017	#DIV/0!	#DIV/0!
P02770	Serum albumin OS=Rattus norvegicus GN=Alb PE=1 SV=2 - [ALBU_RAT]	68.7	1.173	0.664	1.138	0.656	0.461	0.873	1.115	0.948	1.350	1.207	0.438	0.563	0.855	1.071	0.298	0.375	0.876	0.578	0.491	0.701	0.184	0.281	0.259	0.459	0.354	0.800	0.036	0.131	0.022	0.034	0.917	0.856
P04041	Glutathione peroxidase 1 OS=Rattus norvegicus GN=Gpx1 PE=1 SV=4 - [GPX1_RAT]	22.3	0.912	0.782	1.021	0.452	0.401	0.896	1.274	1.236	0.929	0.897	0.621	0.803	0.706	1.099	0.325	0.439	0.800	0.844	0.650	0.816	0.127	0.223	0.917	0.684	0.917	0.950	0.056	0.199	0.014	0.022	0.756	0.853
P04550	Parathymosin OS=Rattus norvegicus OX=10116 GN=Ptms PE=1 SV=2 - [PTMS_RAT]	11.6	0.013	0.013	0.013	0.013	0.013	1.174	1.123	1.559	0.097	0.776	0.013	0.013	0.013	0.013	0.013	0.013	0.013	0.013	0.013	0.013	0.009	0.0285	#DIV/0!	#DIV/0!	#DIV/0!	#DIV/0!	0.009	0.053	0.009	0.020	#DIV/0!	#DIV/0!
P04631	Protein S100-B OS=Rattus norvegicus GN=S100b PE=1 SV=2 - [S100B_RAT]	10.7	0.013	0.013	0.013	0.013	0.013	0.718	0.744	1.150	2.659	0.505	0.013	0.013	0.013	0.013	0.013	0.013	0.013	0.013	0.013	0.013	0.009	0.0226	#DIV/0!	#DIV/0!	#DIV/0!	#DIV/0!	0.009	0.039	0.009	0.016	#DIV/0!	#DIV/0!
P04916	Retinol-binding protein 4 OS=Rattus norvegicus GN=Rbp4 PE=1 SV=1 - [RET4_RAT]	23.2	0.398	0.288	0.417	0.210	0.186	0.356	0.612	0.483	0.295	0.576	0.182	0.303	0.349	0.441	0.186	0.127	0.228	0.267	0.221	0.221	0.085	0.173	0.917	0.703	0.354	0.860	0.085	0.296	0.014	0.023	0.354	0.925
P05545	Serine protease inhibitor A3K OS=Rattus norvegicus GN=Serpina3k PE=1 SV=3 - [SPA3K_RAT]	46.5	1.031	0.572	0.912	0.230	0.377	1.187	1.536	1.251	1.396	1.796	0.364	0.593	0.983	1.357	0.190	0.212	0.510	0.397	0.273	0.700	0.014	0.0318	0.917	0.692	0.354	0.851	0.036	0.129	0.014	0.021	0.470	0.976
P07154	Cathepsin L1 OS=Rattus norvegicus GN=Ctsl PE=1 SV=2 - [CATL1_RAT]	37.6	0.013	0.013	0.013	0.013	0.013	0.696	0.591	0.808	0.396	0.606	0.013	0.013	0.013	0.013	0.013	0.013	0.013	0.013	0.013	0.013	0.009	0.197	#DIV/0!	#DIV/0!	#DIV/0!	#DIV/0!	0.009	0.407	0.009	0.095	#DIV/0!	#DIV/0!
P07338	Chymotrypsinogen B OS=Rattus norvegicus OX=10116 GN=Ctrb1 PE=1 SV=1 - [CTRB1_RAT]	27.8	0.579	0.500	0.741	0.402	0.325	1.005	1.215	1.102	0.576	1.067	0.586	0.688	1.075	1.658	0.281	0.369	0.575	0.646	0.410	0.609	0.036	0.0792	0.259	0.476	0.756	0.994	0.605	0.684	0.036	0.052	0.259	0.773
P07687	Epoxide hydrolase 1 OS=Rattus norvegicus OX=10116 GN=Ephx1 PE=1 SV=1 - [HYEP_RAT]	52.5	0.013	0.013	0.013	0.013	0.013	0.641	0.837	0.816	0.436	0.745	0.013	0.013	0.013	0.013	0.013	0.013	0.013	0.013	0.013	0.013	0.009	0.0953	#DIV/0!	#DIV/0!	#DIV/0!	#DIV/0!	0.009	0.231	0.009	0.057	#DIV/0!	#DIV/0!
P07896	Peroxisomal bifunctional enzyme OS=Rattus norvegicus OX=10116 GN=Ehhadh PE=1 SV=2 - [ECHP_RAT]	78.6	0.531	0.463	1.236	0.619	0.500	0.614	0.817	0.985	0.186	0.589	0.633	0.774	0.758	1.007	0.323	0.013	0.013	0.013	0.013	0.013	0.756	0.577	0.470	0.582	0.009	0.133	0.605	0.810	0.009	0.085	0.009	0.232
P09006	Serine protease inhibitor A3N OS=Rattus norvegicus GN=Serpina3n PE=1 SV=3 - [SPA3N_RAT]	46.6	1.356	0.928	2.377	0.838	0.736	1.117	1.073	1.196	1.308	1.126	0.632	0.931	0.895	1.025	0.319	0.492	1.125	0.985	0.744	0.860	0.605	0.467	0.354	0.449	0.470	0.754	0.014	0.053	0.036	0.050	0.756	0.812
P09117	Fructose-bisphosphate aldolase C OS=Rattus norvegicus GN=Aldoc PE=1 SV=3 - [ALDOC_RAT]	39.3	0.013	0.013	0.013	0.013	0.013	0.958	1.172	1.220	0.324	1.000	0.436	0.716	0.986	1.222	0.231	0.013	0.013	0.013	0.013	0.013	0.009	0.0296	0.009	0.149	#DIV/0!	#DIV/0!	0.470	0.716	0.009	0.021	0.009	0.191
P09760	Tyrosine-protein kinase Fer OS=Rattus norvegicus GN=Fer PE=1 SV=2 - [FER_RAT]	94.3	0.013	0.013	0.013	0.013	0.013	1.012	0.624	1.047	0.496	0.468	0.013	0.013	0.013	0.013	0.013	0.013	0.013	0.013	0.013	0.013	0.009	0.0709	#DIV/0!	#DIV/0!	#DIV/0!	#DIV/0!	0.009	0.175	0.009	0.046	#DIV/0!	#DIV/0!
P10688	1-phosphatidylinositol 4,5-bisphosphate phosphodiesterase delta-1 OS=Rattus norvegicus GN=Plcd1 PE=1 SV=1 - [PLCD1_RAT]	85.9	0.807	0.797	1.619	0.304	0.592	0.932	1.431	1.540	0.190	0.890	0.847	1.292	1.717	1.860	0.266	0.013	0.013	0.013	0.013	0.013	0.470	0.435	0.259	0.416	0.009	0.070	0.605	0.672	0.009	0.018	0.009	0.035
P10959	Carboxylesterase 1C OS=Rattus norvegicus GN=Ces1c PE=1 SV=3 - [EST1C_RAT]	60.1	1.022	0.772	0.820	0.334	0.341	0.999	1.503	1.075	1.348	1.595	0.356	0.607	1.090	1.396	0.217	0.309	0.558	0.355	0.279	0.824	0.022	0.0506	0.756	0.669	0.354	0.843	0.127	0.372	0.014	0.021	0.354	0.896
P12346	Serotransferrin OS=Rattus norvegicus GN=Tf PE=1 SV=3 - [TRFE_RAT]	76.3	1.197	0.841	1.125	0.530	0.409	0.915	1.253	0.885	1.437	1.392	0.429	0.596	0.778	0.950	0.247	0.332	0.842	0.566	0.453	0.751	0.085	0.166	0.354	0.528	0.354	0.804	0.036	0.130	0.014	0.022	0.917	0.860
P12788	Trypsin-4 OS=Rattus norvegicus OX=10116 GN=Try4 PE=2 SV=1 - [TRY4_RAT]	26.6	0.918	0.472	0.941	0.336	0.391	0.683	1.149	1.172	0.625	1.018	0.179	0.803	1.166	1.197	0.115	0.013	0.013	0.013	0.013	0.013	0.085	0.170	0.917	0.693	0.009	0.199	0.756	0.790	0.009	0.021	0.009	0.245
P15083	Polymeric immunoglobulin receptor OS=Rattus norvegicus GN=Pigr PE=1 SV=1 - [PIGR_RAT]	84.7	0.862	1.306	1.503	1.074	0.652	0.645	0.654	0.827	0.421	0.739	0.938	1.125	1.324	1.333	0.590	0.686	0.977	1.350	0.895	1.258	0.056	0.114	0.917	0.618	0.917	0.841	0.085	0.282	0.036	0.051	0.917	0.741
P15429	Beta-enolase OS=Rattus norvegicus GN=Eno3 PE=1 SV=3 - [ENOB_RAT]	47.0	0.013	0.013	0.013	0.013	0.013	1.071	3.932	0.642	0.326	0.924	0.013	0.013	0.013	0.013	0.013	0.013	0.013	0.013	0.013	0.013	0.009	0.0218	#DIV/0!	#DIV/0!	#DIV/0!	#DIV/0!	0.009	0.037	0.009	0.015	#DIV/0!	#DIV/0!
P15684	Aminopeptidase N OS=Rattus norvegicus OX=10116 GN=Anpep PE=1 SV=2 - [AMPN_RAT]	109.4	0.013	0.013	0.013	0.013	0.013	0.663	1.151	1.180	0.436	1.198	0.013	0.013	0.013	0.013	0.013	0.013	0.013	0.013	0.013	0.013	0.009	0.0309	#DIV/0!	#DIV/0!	#DIV/0!	#DIV/0!	0.009	0.058	0.009	0.022	#DIV/0!	#DIV/0!
P16036	Phosphate carrier protein, mitochondrial OS=Rattus norvegicus OX=10116 GN=Slc25a3 PE=1 SV=1 - [MPCP_RAT]	39.4	0.013	0.013	0.013	0.013	0.013	0.013	0.013	0.013	0.013	0.013	0.013	0.013	0.013	0.013	0.013	0.728	0.699	1.199	0.684	1.080	#DIV/0!	#DIV/0!	#DIV/0!	#DIV/0!	0.009	0.060	#DIV/0!	#DIV/0!	0.009	0.024	0.009	0.067
P17046	Lysosome-associated membrane glycoprotein 2 OS=Rattus norvegicus OX=10116 GN=Lamp2 PE=1 SV=3 - [LAMP2_RAT]	45.1	0.745	0.517	1.741	0.830	0.651	0.896	1.734	1.247	0.471	1.675	1.089	1.175	0.934	1.418	0.501	0.013	0.013	0.013	0.013	0.013	0.470	0.422	0.470	0.518	0.009	0.056	0.605	0.671	0.009	0.016	0.009	0.042
P17164	Tissue alpha-L-fucosidase OS=Rattus norvegicus GN=Fuca1 PE=1 SV=1 - [FUCO_RAT]	53.5	0.013	0.013	0.013	0.013	0.013	0.614	0.826	0.921	0.224	0.817	0.013	0.013	0.013	0.013	0.013	0.013	0.013	0.013	0.013	0.013	0.009	0.107	#DIV/0!	#DIV/0!	#DIV/0!	#DIV/0!	0.009	0.276	0.009	0.066	#DIV/0!	#DIV/0!
P17702	60S ribosomal protein L28 OS=Rattus norvegicus GN=Rpl28 PE=1 SV=4 - [RL28_RAT]	15.8	0.013	0.013	0.013	0.013	0.013	0.692	0.975	0.968	0.260	0.935	0.013	0.013	0.013	0.013	0.013	0.013	0.013	0.013	0.013	0.013	0.009	0.0559	#DIV/0!	#DIV/0!	#DIV/0!	#DIV/0!	0.009	0.130	0.009	0.036	#DIV/0!	#DIV/0!
P17955	Nuclear pore glycoprotein p62 OS=Rattus norvegicus GN=Nup62 PE=1 SV=1 - [NUP62_RAT]	53.4	0.459	0.466	1.141	0.185	0.383	0.830	0.900	1.146	0.073	0.957	0.013	0.013	0.013	0.013	0.013	0.013	0.013	0.013	0.013	0.013	0.354	0.462	0.009	0.648	0.009	0.398	0.009	0.117	0.009	0.033	#DIV/0!	#DIV/0!
P20059	Hemopexin OS=Rattus norvegicus GN=Hpx PE=1 SV=3 - [HEMO_RAT]	51.3	1.256	0.910	1.584	0.634	0.608	1.006	1.223	1.091	1.299	1.203	0.522	0.728	0.872	1.031	0.274	0.442	1.094	0.794	0.620	0.854	0.470	0.421	0.259	0.432	0.354	0.735	0.022	0.085	0.036	0.050	0.756	0.849
P20759	Ig gamma-1 chain C region OS=Rattus norvegicus PE=1 SV=1 - [IGHG1_RAT]	35.9	1.210	0.576	1.126	0.912	0.532	1.036	1.377	0.843	1.575	1.131	0.807	0.684	0.825	0.929	0.469	0.404	0.930	0.811	0.712	0.906	0.184	0.279	0.470	0.555	0.470	0.816	0.022	0.085	0.036	0.050	0.756	0.835
P20760	Ig gamma-2A chain C region OS=Rattus norvegicus GN=Igg-2a PE=1 SV=1 - [IGG2A_RAT]	35.2	1.611	0.653	0.750	0.437	0.392	1.016	1.184	1.111	1.560	1.192	0.345	0.385	0.627	0.758	0.292	0.285	0.867	0.699	0.534	0.832	0.127	0.219	0.184	0.391	0.917	0.953	0.014	0.054	0.014	0.021	0.354	0.900
P20762	Ig gamma-2C chain C region OS=Rattus norvegicus PE=2 SV=1 - [IGG2C_RAT]	36.5	1.054	0.506	0.869	0.239	0.369	0.711	0.902	0.752	0.747	0.764	0.973	0.963	1.032	1.164	0.591	0.013	0.013	0.013	0.013	0.013	0.470	0.515	0.127	0.282	0.009	0.219	0.127	0.388	0.009	0.035	0.009	0.050
P20767	Ig lambda-2 chain C region OS=Rattus norvegicus PE=4 SV=1 - [LAC2_RAT]	11.3	0.983	0.590	0.840	0.361	0.392	1.055	1.273	1.490	1.147	1.246	0.260	0.367	0.809	1.022	0.111	0.285	0.702	0.531	0.467	0.645	0.014	0.0319	0.470	0.586	0.605	0.946	0.014	0.054	0.014	0.022	0.756	0.886
P21139	Alpha-mannosidase 2C1 OS=Rattus norvegicus GN=Man2c1 PE=1 SV=1 - [MA2C1_RAT]	115.9	0.013	0.013	0.013	0.013	0.013	0.830	1.477	1.202	0.194	1.849	0.013	0.013	0.013	0.013	0.013	0.013	0.013	0.013	0.013	0.013	0.009	0.0230	#DIV/0!	#DIV/0!	#DIV/0!	#DIV/0!	0.009	0.041	0.009	0.016	#DIV/0!	#DIV/0!
P21670	Proteasome subunit alpha type-4 OS=Rattus norvegicus GN=Psma4 PE=1 SV=1 - [PSA4_RAT]	29.5	0.825	0.924	0.883	0.422	0.401	0.880	1.725	1.194	0.701	1.531	0.404	0.614	0.927	1.102	0.290	0.297	0.649	0.622	0.492	0.738	0.127	0.220	0.917	0.688	0.354	0.833	0.085	0.277	0.022	0.034	0.917	0.858
P23680	Serum amyloid P-component OS=Rattus norvegicus GN=Apcs PE=2 SV=2 - [SAMP_RAT]	26.2	0.013	0.013	0.013	0.013	0.013	0.810	1.135	0.796	0.959	1.277	0.013	0.013	0.013	0.013	0.013	0.013	0.013	0.013	0.013	0.013	0.009	0.0259	#DIV/0!	#DIV/0!	#DIV/0!	#DIV/0!	0.009	0.048	0.009	0.018	#DIV/0!	#DIV/0!
P24049	60S ribosomal protein L17 OS=Rattus norvegicus OX=10116 GN=Rpl17 PE=2 SV=3 - [RL17_RAT]	21.4	0.013	0.013	0.013	0.013	0.013	0.741	0.735	1.051	0.147	0.803	0.013	0.013	0.013	0.013	0.013	0.013	0.013	0.013	0.013	0.013	0.009	0.0938	#DIV/0!	#DIV/0!	#DIV/0!	#DIV/0!	0.009	0.225	0.009	0.056	#DIV/0!	#DIV/0!
P24155	Thimet oligopeptidase OS=Rattus norvegicus GN=Thop1 PE=1 SV=4 - [THOP1_RAT]	78.3	1.010	0.872	1.393	0.610	0.569	0.976	1.151	1.197	0.754	0.941	0.540	0.911	0.955	1.203	0.255	0.376	0.749	0.760	0.683	0.846	0.470	0.428	0.605	0.591	0.354	0.776	0.354	0.613	0.036	0.051	0.470	0.904
P25031	Regenerating islet-derived protein 3-beta OS=Rattus norvegicus OX=10116 GN=Reg3b PE=1 SV=1 - [REG3B_RAT]	19.6	0.013	0.013	0.013	0.013	0.013	0.013	0.013	0.013	0.013	0.013	0.013	0.013	0.013	0.013	0.013	0.382	0.990	0.602	6.156	0.348	#DIV/0!	#DIV/0!	#DIV/0!	#DIV/0!	0.009	0.040	#DIV/0!	#DIV/0!	0.009	0.015	0.009	0.033
P27653	C-1-tetrahydrofolate synthase, cytoplasmic OS=Rattus norvegicus GN=Mthfd1 PE=1 SV=3 - [C1TC_RAT]	100.9	0.371	0.351	1.445	0.474	0.464	0.560	0.701	0.947	0.281	0.585	0.843	1.180	0.859	1.377	0.455	0.013	0.013	0.013	0.013	0.013	0.470	0.525	0.259	0.446	0.009	0.179	0.184	0.509	0.009	0.099	0.009	0.050
P28075	Proteasome subunit beta type-5 OS=Rattus norvegicus GN=Psmb5 PE=1 SV=3 - [PSB5_RAT]	28.6	0.732	0.746	1.429	0.459	0.636	1.039	1.524	1.292	0.523	1.106	0.848	0.857	1.049	1.104	0.313	0.013	0.013	0.013	0.013	0.013	0.259	0.350	0.470	0.550	0.009	0.075	0.184	0.457	0.009	0.017	0.009	0.082
P32038	Complement factor D OS=Rattus norvegicus GN=Cfd PE=1 SV=2 - [CFAD_RAT]	28.4	0.543	0.536	1.275	0.199	0.362	1.022	1.109	1.442	0.326	0.995	0.013	0.013	0.013	0.013	0.013	0.013	0.013	0.013	0.013	0.013	0.259	0.365	0.009	0.396	0.009	0.258	0.009	0.049	0.009	0.018	#DIV/0!	#DIV/0!
P32362	Uroporphyrinogen decarboxylase (Fragment) OS=Rattus norvegicus GN=Urod PE=1 SV=1 - [DCUP_RAT]	40.4	0.013	0.013	0.013	0.013	0.013	0.951	1.540	1.251	0.259	1.060	0.628	0.729	0.978	1.261	0.268	0.013	0.013	0.013	0.013	0.013	0.009	0.0253	0.009	0.1004	#DIV/0!	#DIV/0!	0.470	0.667	0.009	0.018	0.009	0.124
P32551	Cytochrome b-c1 complex subunit 2, mitochondrial OS=Rattus norvegicus OX=10116 GN=Uqcrc2 PE=1 SV=2 - [QCR2_RAT]	48.4	0.514	0.621	1.140	0.421	0.588	0.989	0.886	1.540	0.115	0.737	0.586	0.750	0.885	1.407	0.373	0.013	0.013	0.013	0.013	0.013	0.354	0.436	0.605	0.637	0.009	0.146	0.605	0.753	0.009	0.025	0.009	0.102
P35435	ATP synthase subunit gamma, mitochondrial OS=Rattus norvegicus GN=Atp5c1 PE=1 SV=2 - [ATPG_RAT]	30.2	0.013	0.013	0.013	0.013	0.013	0.013	0.013	0.013	0.013	0.013	0.339	0.637	0.879	1.217	0.121	0.352	0.720	0.849	0.545	0.848	#DIV/0!	#DIV/0!	0.009	0.357	0.009	0.139	0.009	0.389	0.009	0.075	0.917	0.851
P35704	Peroxiredoxin-2 OS=Rattus norvegicus GN=Prdx2 PE=1 SV=3 - [PRDX2_RAT]	21.8	0.791	0.554	0.948	0.183	0.369	0.942	1.458	1.308	0.494	1.087	0.268	0.702	0.854	1.209	0.134	0.269	0.411	0.389	0.275	0.696	0.085	0.169	0.917	0.696	0.470	0.924	0.127	0.391	0.022	0.034	0.605	0.899
P36876	Serine/threonine-protein phosphatase 2A 55 kDa regulatory subunit B alpha isoform OS=Rattus norvegicus GN=Ppp2r2a PE=2 SV=1 - [2ABA_RAT]	51.6	0.756	0.867	1.162	0.561	0.384	0.661	1.136	1.150	0.336	1.009	0.437	1.318	0.907	1.177	0.328	0.013	0.013	0.013	0.013	0.013	0.756	0.552	0.605	0.609	0.009	0.092	0.917	0.770	0.009	0.025	0.009	0.083
P37996	ADP-ribosylation factor-like protein 3 OS=Rattus norvegicus GN=Arl3 PE=1 SV=2 - [ARL3_RAT]	20.4	0.759	0.394	1.482	0.308	0.402	0.665	1.045	1.085	0.179	0.917	0.013	0.013	0.013	0.013	0.013	0.013	0.013	0.013	0.013	0.013	0.605	0.529	0.009	0.255	0.009	0.135	0.009	0.120	0.009	0.034	#DIV/0!	#DIV/0!
P40112	Proteasome subunit beta type-3 OS=Rattus norvegicus GN=Psmb3 PE=1 SV=1 - [PSB3_RAT]	22.9	0.987	1.130	0.737	0.287	0.408	1.044	1.390	1.251	0.697	1.170	0.517	0.904	1.012	1.076	0.266	0.301	0.595	0.507	0.402	0.838	0.085	0.168	0.917	0.681	0.470	0.913	0.085	0.278	0.022	0.034	0.259	0.777
P43245	Multidrug resistance protein 1 OS=Rattus norvegicus OX=10116 GN=Abcb1 PE=2 SV=1 - [MDR1_RAT]	141.3	0.013	0.013	0.013	0.013	0.013	0.013	0.013	0.013	0.013	0.013	0.013	0.013	0.013	0.013	0.013	0.629	0.946	1.176	0.985	1.139	#DIV/0!	#DIV/0!	#DIV/0!	#DIV/0!	0.009	0.051	#DIV/0!	#DIV/0!	0.009	0.019	0.009	0.047
P47245	Nardilysin OS=Rattus norvegicus GN=Nrd1 PE=1 SV=1 - [NRDC_RAT]	132.9	1.177	0.818	1.264	0.438	0.511	0.013	0.013	0.013	0.013	0.013	0.356	0.733	1.126	1.295	0.153	0.382	0.733	0.781	0.621	0.957	0.009	0.0393	0.605	0.611	0.470	0.837	0.009	0.161	0.009	0.057	0.917	0.839
P48199	C-reactive protein OS=Rattus norvegicus GN=Crp PE=1 SV=1 - [CRP_RAT]	25.5	0.594	0.406	1.038	0.205	0.211	0.942	1.486	0.951	1.134	1.823	0.368	0.487	0.757	0.883	0.228	0.174	0.457	0.427	0.276	0.409	0.036	0.0780	0.605	0.652	0.756	1.003	0.014	0.053	0.014	0.022	0.259	0.787
P50297	Arylamine N-acetyltransferase 1 OS=Rattus norvegicus OX=10116 GN=Nat1 PE=2 SV=1 - [ARY1_RAT]	33.4	1.234	0.907	1.258	0.434	0.425	0.013	0.013	0.013	0.013	0.013	0.438	0.726	0.979	1.288	0.248	0.513	1.309	0.720	0.645	1.458	0.009	0.0374	0.917	0.665	0.470	0.776	0.009	0.158	0.009	0.021	0.470	0.763
P52303	AP-1 complex subunit beta-1 OS=Rattus norvegicus GN=Ap1b1 PE=1 SV=1 - [AP1B1_RAT]	104.5	0.013	0.013	0.013	0.013	0.013	0.874	1.535	1.494	0.358	0.989	0.013	1.138	0.889	1.485	0.013	0.119	0.313	0.269	0.162	0.565	0.009	0.0240	0.063	0.151	0.009	1.751	0.353	0.703	0.022	0.034	0.604	0.906
P53812	Phosphatidylinositol transfer protein beta isoform OS=Rattus norvegicus GN=Pitpnb PE=1 SV=2 - [PIPNB_RAT]	31.4	0.013	0.013	0.013	0.013	0.013	0.710	0.823	1.093	0.332	0.796	0.013	0.013	0.013	0.013	0.013	0.013	0.013	0.013	0.013	0.013	0.009	0.0610	#DIV/0!	#DIV/0!	#DIV/0!	#DIV/0!	0.009	0.145	0.009	0.040	#DIV/0!	#DIV/0!
P55068	Brevican core protein OS=Rattus norvegicus GN=Bcan PE=1 SV=2 - [PGCB_RAT]	96.0	1.309	1.522	2.212	2.014	0.907	0.013	0.013	0.013	0.013	0.013	1.603	1.769	0.786	0.829	0.902	0.459	1.380	1.209	1.357	1.049	0.009	0.0214	0.184	0.347	0.184	0.512	0.009	0.039	0.009	0.017	0.917	0.739
P55159	Serum paraoxonase/arylesterase 1 OS=Rattus norvegicus GN=Pon1 PE=1 SV=3 - [PON1_RAT]	39.3	0.013	0.013	0.013	0.013	0.013	0.873	1.193	0.756	0.066	1.647	0.013	0.013	0.013	0.013	0.013	0.013	0.013	0.013	0.013	0.013	0.009	0.0323	#DIV/0!	#DIV/0!	#DIV/0!	#DIV/0!	0.009	0.062	0.009	0.022	#DIV/0!	#DIV/0!
P61314	60S ribosomal protein L15 OS=Rattus norvegicus GN=Rpl15 PE=1 SV=2 - [RL15_RAT]	24.1	0.013	0.013	0.013	0.013	0.013	0.804	1.102	1.186	0.225	1.268	0.013	0.013	0.013	0.013	0.013	0.013	0.013	0.013	0.013	0.013	0.009	0.0316	#DIV/0!	#DIV/0!	#DIV/0!	#DIV/0!	0.009	0.060	0.009	0.022	#DIV/0!	#DIV/0!
P61459	Pterin-4-alpha-carbinolamine dehydratase OS=Rattus norvegicus OX=10116 GN=Pcbd1 PE=1 SV=2 - [PHS_RAT]	12.0	0.013	0.013	0.013	0.013	0.013	0.901	0.762	0.800	0.467	0.908	0.561	0.742	0.551	1.107	0.394	0.013	0.013	0.013	0.013	0.013	0.009	0.0549	0.009	0.246	#DIV/0!	#DIV/0!	0.354	0.680	0.009	0.035	0.009	0.286
P62634	Cellular nucleic acid-binding protein OS=Rattus norvegicus GN=Cnbp PE=2 SV=1 - [CNBP_RAT]	19.4	0.844	0.886	1.518	0.448	0.684	0.673	1.053	1.091	0.142	0.809	0.745	0.979	1.133	1.399	0.402	0.013	0.013	0.013	0.013	0.013	0.756	0.548	0.756	0.595	0.009	0.061	0.470	0.699	0.009	0.039	0.009	0.054
P62718	60S ribosomal protein L18a OS=Rattus norvegicus OX=10116 GN=Rpl18a PE=2 SV=1 - [RL18A_RAT]	20.7	0.410	0.598	1.241	0.480	0.563	0.657	0.718	0.889	0.100	0.997	0.620	0.953	0.954	0.736	0.429	0.013	0.013	0.013	0.013	0.013	0.470	0.519	0.354	0.532	0.009	0.144	0.917	0.826	0.009	0.068	0.009	0.162
P62747	Rho-related GTP-binding protein RhoB OS=Rattus norvegicus GN=Rhob PE=1 SV=1 - [RHOB_RAT]	22.1	0.013	0.013	0.013	0.013	0.013	0.763	1.898	1.362	0.236	1.515	0.013	0.359	1.058	1.960	0.013	0.213	0.190	0.184	0.093	0.805	0.009	0.0227	0.063	0.152	0.009	1.459	0.353	0.701	0.036	0.052	0.755	0.897
P62902	60S ribosomal protein L31 OS=Rattus norvegicus OX=10116 GN=Rpl31 PE=2 SV=1 - [RL31_RAT]	14.5	0.013	0.013	0.013	0.013	0.013	0.774	0.987	1.233	0.212	0.949	0.257	0.443	1.089	1.535	0.228	0.013	0.013	0.013	0.013	0.013	0.009	0.0420	0.009	0.166	#DIV/0!	#DIV/0!	0.917	0.815	0.009	0.027	0.009	0.215
P62912	60S ribosomal protein L32 OS=Rattus norvegicus OX=10116 GN=Rpl32 PE=1 SV=2 - [RL32_RAT]	15.8	0.689	0.744	1.270	0.426	0.551	0.013	0.013	0.013	0.013	0.013	0.579	0.997	1.083	0.876	0.287	0.622	0.526	0.873	0.491	0.774	0.009	0.0685	0.756	0.653	0.917	0.959	0.009	0.134	0.009	0.076	0.354	0.859

P63086	Mitogen-activated protein kinase 1 OS=Rattus norvegicus GN=Mapk1 PE=1 SV=3 - [MK01_RAT]	41.2	0.572	0.622	1.235	0.325	0.401	0.857	1.188	1.242	0.233	0.852	0.641	0.901	0.868	1.071	0.353	0.013	0.013	0.013	0.013	0.013	0.354	0.438	0.354	0.531	0.009	0.175	0.756	0.781	0.009	0.024	0.009	0.130
P63095	Guanine nucleotide-binding protein G(s) subunit alpha isoforms short OS=Rattus norvegicus GN=Gnas PE=1 SV=1 - [GNAS2_RAT]	45.6	0.013	0.013	0.013	0.013	0.013	0.747	0.824	1.133	0.311	0.726	0.013	0.013	0.013	0.013	0.013	0.013	0.013	0.013	0.013	0.013	0.009	0.0629	#DIV/0!	#DIV/0!	#DIV/0!	#DIV/0!	0.009	0.150	0.009	0.041	#DIV/0!	#DIV/0!
P63100	Calcineurin subunit B type 1 OS=Rattus norvegicus GN=Ppp3r1 PE=1 SV=2 - [CANB1_RAT]	19.3	0.013	0.013	0.013	0.013	0.013	0.963	1.297	1.378	0.490	1.252	0.138	0.640	0.986	1.846	0.070	0.013	0.013	0.013	0.013	0.013	0.009	0.0235	0.009	0.140	#DIV/0!	#DIV/0!	0.354	0.607	0.009	0.017	0.009	0.165
P70531	Eukaryotic elongation factor 2 kinase OS=Rattus norvegicus GN=Eef2k PE=1 SV=1 - [EF2K_RAT]	81.4	0.013	0.013	0.013	0.013	0.013	0.013	0.013	0.013	0.013	0.013	0.013	0.013	0.013	0.013	0.013	0.612	0.723	0.914	0.517	1.011	#DIV/0!	#DIV/0!	#DIV/0!	#DIV/0!	0.009	0.090	#DIV/0!	#DIV/0!	0.009	0.038	0.009	0.145
P70569	Unconventional myosin-Vb OS=Rattus norvegicus OX=10116 GN=Myo5b PE=1 SV=1 - [MYO5B_RAT]	213.6	0.013	0.013	0.013	0.013	0.013	0.013	0.013	0.013	0.013	0.013	0.013	0.013	0.013	0.013	0.013	0.612	1.392	1.399	1.063	1.073	#DIV/0!	#DIV/0!	#DIV/0!	#DIV/0!	0.009	0.045	#DIV/0!	#DIV/0!	0.009	0.016	0.009	0.037
P70580	Membrane-associated progesterone receptor component 1 OS=Rattus norvegicus OX=10116 GN=Pgrmc1 PE=1 SV=3 - [PGRC1_RAT]	21.6	0.825	0.418	2.199	0.619	0.560	0.625	0.848	1.107	0.223	0.736	0.771	0.920	0.980	1.313	0.451	0.013	0.013	0.013	0.013	0.013	0.756	0.548	0.470	0.528	0.009	0.054	0.354	0.666	0.009	0.051	0.009	0.065
P70582	Nuclear pore complex protein Nup54 OS=Rattus norvegicus GN=Nup54 PE=1 SV=1 - [NUP54_RAT]	55.7	0.891	0.846	1.789	0.757	0.623	1.092	1.170	1.463	0.402	0.626	0.654	1.049	0.951	1.192	0.404	0.013	0.013	0.013	0.013	0.013	0.917	0.549	0.917	0.628	0.009	0.050	0.756	0.710	0.009	0.020	0.009	0.077
P81799	N-acetyl-D-glucosamine kinase OS=Rattus norvegicus GN=Nagk PE=1 SV=4 - [NAGK_RAT]	37.2	0.013	0.013	0.013	0.013	0.013	0.941	1.345	1.376	0.253	0.968	0.358	0.603	0.819	1.306	0.135	0.013	0.013	0.013	0.013	0.013	0.009	0.0267	0.009	0.340	#DIV/0!	#DIV/0!	0.184	0.499	0.009	0.019	0.009	0.330
P97571	Calpain-1 catalytic subunit OS=Rattus norvegicus GN=Capn1 PE=1 SV=1 - [CAN1_RAT]	82.1	0.857	0.795	1.834	0.995	0.751	0.013	0.013	0.013	0.013	0.013	0.722	1.036	0.929	1.242	0.603	0.622	0.733	1.165	0.741	1.025	0.009	0.0243	0.756	0.586	0.354	0.716	0.009	0.062	0.009	0.025	0.917	0.770
P97608	5-oxoprolinase OS=Rattus norvegicus GN=Oplah PE=1 SV=2 - [OPLA_RAT]	137.6	0.502	0.679	0.730	0.261	0.334	0.013	0.013	0.013	0.013	0.013	0.436	0.830	1.015	1.380	0.245	0.456	0.657	0.976	0.605	0.915	0.009	0.339	0.354	0.543	0.259	0.700	0.009	0.119	0.009	0.047	0.917	0.826
Q02356	AMP deaminase 2 OS=Rattus norvegicus GN=Ampd2 PE=2 SV=2 - [AMPD2_RAT]	94.7	0.013	0.013	0.013	0.013	0.013	0.744	0.896	1.021	0.299	0.786	0.013	0.013	0.013	0.013	0.013	0.013	0.013	0.013	0.013	0.013	0.009	0.0622	#DIV/0!	#DIV/0!	#DIV/0!	#DIV/0!	0.009	0.147	0.009	0.040	#DIV/0!	#DIV/0!
Q03386	Ral guanine nucleotide dissociation stimulator OS=Rattus norvegicus OX=10116 GN=Ralgds PE=1 SV=1 - [GNDS_RAT]	98.8	1.050	0.837	2.034	1.169	0.842	0.663	0.834	0.869	0.359	0.698	0.796	1.079	0.893	1.264	0.660	0.013	0.013	0.013	0.013	0.013	0.036	0.0777	0.470	0.510	0.009	0.043	0.184	0.496	0.009	0.062	0.009	0.052
Q09326	Alpha-1,6-mannosyl-glycoprotein 2-beta-N-acetylglucosaminyltransferase OS=Rattus norvegicus GN=Mgat2 PE=1 SV=1 - [MGAT2_RAT]	51.1	0.013	0.013	0.013	0.013	0.013	0.013	0.013	0.013	0.013	0.013	0.013	0.013	0.013	0.013	0.013	0.607	0.823	1.303	0.683	0.994	#DIV/0!	#DIV/0!	#DIV/0!	#DIV/0!	0.009	0.059	#DIV/0!	#DIV/0!	0.009	0.023	0.009	0.065
Q2LC84	Protein numb homolog OS=Rattus norvegicus GN=Numb PE=1 SV=2 - [NUMB_RAT]	70.6	1.755	1.613	1.723	3.079	1.149	0.498	0.493	0.618	0.302	0.279	1.766	1.571	0.594	0.611	1.427	0.704	2.009	1.749	2.459	1.262	0.014	0.0317	0.184	0.346	0.917	0.836	0.036	0.133	0.014	0.021	0.259	0.680
Q32PZ3	Protein unc-45 homolog A OS=Rattus norvegicus GN=Unc45a PE=2 SV=1 - [UN45A_RAT]	103.2	0.013	0.013	0.013	0.013	0.013	0.013	0.013	0.013	0.013	0.013	0.013	0.013	0.013	0.013	0.013	0.834	0.942	1.749	1.035	2.981	#DIV/0!	#DIV/0!	#DIV/0!	#DIV/0!	0.009	0.041	#DIV/0!	#DIV/0!	0.009	0.015	0.009	0.034
Q3MID3	ADP-ribosylation factor GTPase-activating protein 2 OS=Rattus norvegicus GN=Arfgap2 PE=1 SV=1 - [ARFG2_RAT]	56.5	0.743	0.728	1.865	0.632	0.677	0.013	0.013	0.013	0.013	0.013	0.816	1.079	0.732	1.157	0.505	0.745	0.761	1.277	0.692	1.056	0.009	0.0305	0.605	0.577	0.259	0.642	0.009	0.076	0.009	0.022	0.917	0.770
Q4G075	Leukocyte elastase inhibitor A OS=Rattus norvegicus GN=Serpinb1a PE=1 SV=1 - [ILEUA_RAT]	42.7	0.973	0.764	1.152	0.410	0.532	1.034	0.923	1.134	1.107	0.866	0.471	0.830	0.893	1.283	0.221	0.457	0.735	0.640	0.478	0.926	0.259	0.354	0.917	0.677	0.470	0.875	0.184	0.471	0.036	0.052	0.756	0.860
Q4KM35	Proteasome subunit beta type-10 OS=Rattus norvegicus GN=Psmb10 PE=2 SV=1 - [PSB10_RAT]	29.0	0.013	0.013	0.013	0.013	0.013	0.895	1.447	1.254	0.690	0.980	0.013	0.013	0.013	0.013	0.013	0.013	0.013	0.013	0.013	0.013	0.009	0.0239	#DIV/0!	#DIV/0!	#DIV/0!	#DIV/0!	0.009	0.043	0.009	0.017	#DIV/0!	#DIV/0!
Q4KM65	Cleavage and polyadenylation specificity factor subunit 5 OS=Rattus norvegicus GN=Nudt21 PE=2 SV=1 - [CPSF5_RAT]	26.2	0.013	0.013	0.013	0.013	0.013	0.732	0.743	1.017	0.439	0.648	0.013	0.013	0.013	0.013	0.013	0.013	0.013	0.013	0.013	0.013	0.009	0.0753	#DIV/0!	#DIV/0!	#DIV/0!	#DIV/0!	0.009	0.190	0.009	0.048	#DIV/0!	#DIV/0!
Q4QQS8	Nuclear pore complex protein Nup85 OS=Rattus norvegicus GN=Nup85 PE=2 SV=1 - [NUP85_RAT]	74.8	0.013	0.013	0.013	0.013	0.013	0.013	0.013	0.013	0.013	0.013	0.013	0.013	0.013	0.013	0.013	0.447	0.929	0.944	0.781	0.813	#DIV/0!	#DIV/0!	#DIV/0!	#DIV/0!	0.009	0.082	#DIV/0!	#DIV/0!	0.009	0.033	0.009	0.114
Q4V8B0	Oxidation resistance protein 1 OS=Rattus norvegicus GN=Oxr1 PE=2 SV=3 - [OXR1_RAT]	92.8	0.013	0.013	0.013	0.013	0.013	0.013	0.013	0.013	0.013	0.013	0.013	0.013	0.013	0.013	0.013	0.558	0.729	0.865	0.481	0.998	#DIV/0!	#DIV/0!	#DIV/0!	#DIV/0!	0.009	0.103	#DIV/0!	#DIV/0!	0.009	0.047	0.009	0.183
Q4V8F9	Hydroxysteroid dehydrogenase-like protein 2 OS=Rattus norvegicus OX=10116 GN=Hsdl2 PE=2 SV=1 - [HSDL2_RAT]	58.3	0.013	0.013	0.013	0.013	0.013	0.602	1.237	1.091	0.406	0.904	0.339	0.600	0.609	1.205	0.119	0.013	0.013	0.013	0.013	0.013	0.009	0.0379	0.009	0.419	#DIV/0!	#DIV/0!	0.259	0.625	0.009	0.026	0.009	0.429
Q58FK9	Kynurenine--oxoglutarate transaminase 3 OS=Rattus norvegicus GN=Ccbl2 PE=2 SV=1 - [KAT3_RAT]	51.0	0.857	0.800	1.168	0.443	0.492	0.970	1.207	1.207	0.658	0.971	0.360	0.816	0.837	1.137	0.207	0.368	0.652	0.700	0.487	0.811	0.127	0.224	0.605	0.641	0.354	0.815	0.127	0.393	0.036	0.052	0.605	0.890
Q5BJN8	Replication stress response regulator SDE2 OS=Rattus norvegicus OX=10116 GN=Sde2 PE=1 SV=2 - [SDE2_RAT]	48.1	0.013	0.013	0.013	0.013	0.013	0.013	0.013	0.013	0.013	0.013	0.013	0.013	0.013	0.013	0.013	0.684	2.667	2.439	2.312	1.078	#DIV/0!	#DIV/0!	#DIV/0!	#DIV/0!	0.009	0.040	#DIV/0!	#DIV/0!	0.009	0.015	0.009	0.033
Q5FVH2	Phospholipase D3 OS=Rattus norvegicus GN=Pld3 PE=2 SV=1 - [PLD3_RAT]	54.4	0.013	0.013	0.013	0.013	0.013	0.013	0.013	0.013	0.013	0.013	0.013	0.013	0.013	0.013	0.013	0.568	0.864	1.193	0.833	0.965	#DIV/0!	#DIV/0!	#DIV/0!	#DIV/0!	0.009	0.058	#DIV/0!	#DIV/0!	0.009	0.023	0.009	0.065
Q5FVI6	V-type proton ATPase subunit C 1 OS=Rattus norvegicus GN=Atp6v1c1 PE=2 SV=1 - [VATC1_RAT]	43.9	0.013	0.013	0.013	0.013	0.013	0.573	0.853	1.181	0.067	0.824	0.013	0.013	0.013	0.013	0.013	0.013	0.013	0.013	0.013	0.013	0.009	0.0897	#DIV/0!	#DIV/0!	#DIV/0!	#DIV/0!	0.009	0.209	0.009	0.055	#DIV/0!	#DIV/0!
Q5FVL2	ER membrane protein complex subunit 8 OS=Rattus norvegicus OX=10116 GN=Emc8 PE=2 SV=1 - [EMC8_RAT]	23.4	0.709	0.796	1.134	0.519	0.562	0.013	0.013	0.013	0.013	0.013	0.013	0.013	0.013	0.013	0.013	0.765	0.657	0.966	0.727	0.943	0.009	0.0649	0.009	0.130	0.470	0.834	#DIV/0!	#DIV/0!	0.009	0.029	0.009	0.094
Q5I0K3	Citramalyl-CoA lyase, mitochondrial OS=Rattus norvegicus OX=10116 GN=Clybl PE=2 SV=2 - [CLYBL_RAT]	37.3	0.013	0.013	0.013	0.013	0.013	0.776	0.999	0.987	0.316	0.731	0.452	0.509	0.672	0.752	0.372	0.013	0.013	0.013	0.013	0.013	0.009	0.0575	0.009	0.446	#DIV/0!	#DIV/0!	0.184	0.521	0.009	0.037	0.009	0.572
Q5M7T9	Threonine synthase-like 2 OS=Rattus norvegicus OX=10116 GN=Thnsl2 PE=2 SV=1 - [THNS2_RAT]	54.1	0.013	0.013	0.013	0.013	0.013	0.630	0.891	0.877	0.259	0.815	0.584	0.793	0.895	0.970	0.461	0.013	0.013	0.013	0.013	0.013	0.009	0.0968	0.009	0.135	#DIV/0!	#DIV/0!	0.756	0.824	0.009	0.058	0.009	0.159
Q5PPJ4	Deoxyhypusine hydroxylase OS=Rattus norvegicus OX=10116 GN=Dohh PE=2 SV=1 - [DOHH_RAT]	33.1	0.013	0.013	0.013	0.013	0.013	0.013	0.013	0.013	0.013	0.013	0.013	0.013	0.013	0.013	0.013	0.681	1.025	1.318	0.819	1.129	#DIV/0!	#DIV/0!	#DIV/0!	#DIV/0!	0.009	0.049	#DIV/0!	#DIV/0!	0.009	0.018	0.009	0.045
Q5PQN7	Protein LZIC OS=Rattus norvegicus OX=10116 GN=Lzic PE=2 SV=1 - [LZIC_RAT]	21.4	0.013	0.013	0.013	0.013	0.013	0.013	0.013	0.013	0.013	0.013	0.013	0.013	0.013	0.013	0.013	0.720	0.950	1.311	0.945	0.951	#DIV/0!	#DIV/0!	#DIV/0!	#DIV/0!	0.009	0.051	#DIV/0!	#DIV/0!	0.009	0.019	0.009	0.046
Q5PQR8	EKC/KEOPS complex subunit Tprkb OS=Rattus norvegicus OX=10116 GN=Tprkb PE=2 SV=1 - [TPRKB_RAT]	17.6	0.013	0.013	0.013	0.013	0.013	0.936	1.269	1.608	0.226	1.301	0.013	0.013	0.013	0.013	0.013	0.013	0.013	0.013	0.013	0.013	0.009	0.0237	#DIV/0!	#DIV/0!	#DIV/0!	#DIV/0!	0.009	0.042	0.009	0.017	#DIV/0!	#DIV/0!
Q5RJP0	Aldose reductase-related protein 1 OS=Rattus norvegicus GN=Akr1b7 PE=1 SV=1 - [ALD1_RAT]	36.1	0.013	0.013	0.013	0.013	0.013	0.517	0.723	0.795	0.362	1.085	0.013	0.013	0.013	0.013	0.013	0.013	0.013	0.013	0.013	0.013	0.009	0.0924	#DIV/0!	#DIV/0!	#DIV/0!	#DIV/0!	0.009	0.219	0.009	0.055	#DIV/0!	#DIV/0!
Q5U1Y4	1,5-anhydro-D-fructose reductase OS=Rattus norvegicus GN=Akr1e2 PE=2 SV=1 - [AKCL2_RAT]	34.5	0.013	0.013	0.013	0.013	0.013	1.027	1.195	1.136	1.103	1.146	0.013	0.013	0.013	0.013	0.013	0.013	0.013	0.013	0.013	0.013	0.009	0.0229	#DIV/0!	#DIV/0!	#DIV/0!	#DIV/0!	0.009	0.040	0.009	0.016	#DIV/0!	#DIV/0!
Q5U1Z2	Trafficking protein particle complex subunit 3 OS=Rattus norvegicus GN=Trappc3 PE=2 SV=1 - [TPPC3_RAT]	20.3	0.495	0.549	1.354	0.426	0.481	0.013	0.013	0.013	0.013	0.013	0.774	0.975	0.935	1.790	0.307	0.599	0.279	0.858	0.439	1.019	0.009	0.135	0.354	0.492	0.917	0.969	0.009	0.052	0.009	0.083	0.354	0.832
Q5U3Z3	Isochorismatase domain-containing protein 2 OS=Rattus norvegicus OX=10116 GN=Isoc2 PE=2 SV=1 - [ISOC2_RAT]	23.1	0.684	0.516	0.903	0.429	0.371	0.790	1.103	0.997	0.504	1.135	0.277	0.592	0.738	1.326	0.159	0.013	0.013	0.013	0.013	0.013	0.085	0.170	0.917	0.697	0.009	0.265	0.259	0.616	0.009	0.022	0.009	0.358
Q5XI22	Acetyl-CoA acetyltransferase, cytosolic OS=Rattus norvegicus GN=Acat2 PE=1 SV=1 - [THIC_RAT]	41.1	0.013	0.013	0.013	0.013	0.013	0.836	1.387	1.212	0.377	1.124	0.013	0.013	0.013	0.013	0.013	0.013	0.013	0.013	0.013	0.013	0.009	0.0262	#DIV/0!	#DIV/0!	#DIV/0!	#DIV/0!	0.009	0.048	0.009	0.018	#DIV/0!	#DIV/0!
Q5XI46	ATPase PAAT OS=Rattus norvegicus OX=10116 GN=Paat PE=2 SV=1 - [PAAT_RAT]	48.8	0.013	0.013	0.013	0.013	0.013	0.013	0.013	0.013	0.013	0.013	0.013	0.013	0.013	0.013	0.013	0.451	1.148	0.759	0.666	0.883	#DIV/0!	#DIV/0!	#DIV/0!	#DIV/0!	0.009	0.083	#DIV/0!	#DIV/0!	0.009	0.033	0.009	0.118
Q5XIG8	Serine-threonine kinase receptor-associated protein OS=Rattus norvegicus GN=Strap PE=1 SV=1 - [STRAP_RAT]	38.4	0.901	0.853	1.401	0.657	0.545	0.013	0.013	0.013	0.013	0.013	0.766	1.036	1.000	1.168	0.413	0.483	0.774	1.036	0.887	0.909	0.009	0.0348	0.756	0.606	0.917	0.891	0.009	0.071	0.009	0.028	0.756	0.769
Q5XIJ6	BRISC and BRCA1-A complex member 1 OS=Rattus norvegicus GN=Babam1 PE=1 SV=1 - [BABA1_RAT]	36.9	0.013	0.013	0.013	0.013	0.013	0.695	0.887	1.082	0.131	0.789	0.013	0.013	0.013	0.013	0.013	0.013	0.013	0.013	0.013	0.013	0.009	0.0744	#DIV/0!	#DIV/0!	#DIV/0!	#DIV/0!	0.009	0.186	0.009	0.048	#DIV/0!	#DIV/0!
Q62658	Peptidyl-prolyl cis-trans isomerase FKBP1A OS=Rattus norvegicus GN=Fkbp1a PE=1 SV=3 - [FKB1A_RAT]	11.9	0.013	0.013	0.013	0.013	0.013	0.790	0.785	1.083	0.148	0.639	0.013	0.013	0.013	0.013	0.013	0.013	0.013	0.013	0.013	0.013	0.009	0.0999	#DIV/0!	#DIV/0!	#DIV/0!	#DIV/0!	0.009	0.238	0.009	0.061	#DIV/0!	#DIV/0!
Q62745	CD81 antigen OS=Rattus norvegicus GN=Cd81 PE=1 SV=1 - [CD81_RAT]	25.9	0.013	0.013	0.013	0.013	0.013	0.625	1.004	0.808	0.299	0.963	0.013	0.013	0.013	0.013	0.013	0.013	0.013	0.013	0.013	0.013	0.009	0.0677	#DIV/0!	#DIV/0!	#DIV/0!	#DIV/0!	0.009	0.155	0.009	0.044	#DIV/0!	#DIV/0!
Q62751	Iron-responsive element-binding protein 2 OS=Rattus norvegicus GN=Ireb2 PE=1 SV=2 - [IREB2_RAT]	104.7	0.013	0.013	0.013	0.013	0.013	0.869	0.695	1.011	0.276	0.566	0.013	0.013	0.013	0.013	0.013	0.013	0.013	0.013	0.013	0.013	0.009	0.105	#DIV/0!	#DIV/0!	#DIV/0!	#DIV/0!	0.009	0.252	0.009	0.064	#DIV/0!	#DIV/0!
Q62940	E3 ubiquitin-protein ligase NEDD4 OS=Rattus norvegicus GN=Nedd4 PE=1 SV=1 - [NEDD4_RAT]	102.3	0.916	0.736	1.219	0.705	0.502	0.013	0.013	0.013	0.013	0.013	0.742	0.946	1.066	1.276	0.270	0.420	0.822	0.839	0.957	1.006	0.009	0.0448	0.470	0.546	0.756	0.898	0.009	0.074	0.009	0.030	0.756	0.783
Q62975	Protein Z-dependent protease inhibitor OS=Rattus norvegicus GN=Serpina10 PE=2 SV=2 - [ZPI_RAT]	50.2	0.013	0.013	0.013	0.013	0.013	0.971	1.083	1.344	0.707	0.994	0.013	0.013	0.013	0.013	0.013	0.013	0.013	0.013	0.013	0.013	0.009	0.0251	#DIV/0!	#DIV/0!	#DIV/0!	#DIV/0!	0.009	0.046	0.009	0.017	#DIV/0!	#DIV/0!
Q63009	Protein arginine N-methyltransferase 1 OS=Rattus norvegicus GN=Prmt1 PE=1 SV=1 - [ANM1_RAT]	40.5	0.524	0.611	1.188	0.313	0.525	0.013	0.013	0.013	0.013	0.013	0.748	0.955	1.232	1.392	0.337	0.471	0.554	0.876	0.442	0.932	0.009	0.174	0.184	0.370	0.917	0.973	0.009	0.056	0.009	0.077	0.259	0.719
Q63028	Alpha-adducin OS=Rattus norvegicus GN=Add1 PE=2 SV=2 - [ADDA_RAT]	80.3	0.013	0.013	0.013	0.013	0.013	0.846	0.934	1.036	0.396	0.848	0.534	0.761	0.626	1.010	0.514	0.013	0.013	0.013	0.013	0.013	0.009	0.0462	0.009	0.210	#DIV/0!	#DIV/0!	0.354	0.677	0.009	0.029	0.009	0.260
Q63041	Alpha-1-macroglobulin OS=Rattus norvegicus GN=A1m PE=1 SV=1 - [A1M_RAT]	167.0	1.461	0.697	2.293	0.987	0.593	0.935	1.253	0.932	1.322	1.344	0.489	0.909	0.874	0.830	0.331	0.319	1.145	0.708	0.799	0.777	0.917	0.541	0.184	0.354	0.470	0.759	0.014	0.053	0.036	0.050	0.756	0.851
Q63556	Serine protease inhibitor A3M (Fragment) OS=Rattus norvegicus GN=Serpina3m PE=2 SV=1 - [SPA3M_RAT]	46.1	1.030	0.696	1.397	0.775	0.606	0.932	1.050	1.050	1.231	1.405	0.865	1.053	0.987	1.285	0.487	0.458	0.945	0.925	0.691	0.920	0.127	0.218	0.756	0.592	0.605	0.857	0.354	0.588	0.022	0.034	0.259	0.695
Q63622	Disks large homolog 2 OS=Rattus norvegicus GN=Dlg2 PE=1 SV=1 - [DLG2_RAT]	94.9	0.013	0.013	0.013	0.013	0.013	0.815	1.403	1.316	0.230	1.087	0.013	0.013	0.013	0.013	0.013	0.013	0.013	0.013	0.013	0.013	0.009	0.0273	#DIV/0!	#DIV/0!	#DIV/0!	#DIV/0!	0.009	0.050	0.009	0.019	#DIV/0!	#DIV/0!
Q641Y8	ATP-dependent RNA helicase DDX1 OS=Rattus norvegicus GN=Ddx1 PE=2 SV=1 - [DDX1_RAT]	82.4	0.686	0.725	0.965	0.367	0.351	0.982	1.400	1.350	0.962	1.475	0.367	0.562	1.164	1.386	0.185	0.013	0.013	0.013	0.013	0.013	0.022	0.0510	0.917	0.690	0.009	0.182	0.127	0.375	0.009	0.016	0.009	0.168
Q641Z8	Peflin OS=Rattus norvegicus GN=Pef1 PE=2 SV=1 - [PEF1_RAT]	30.0	0.917	0.626	1.632	0.445	0.632	0.784	0.931	0.960	0.116	0.728	0.709	1.137	0.896	1.270	0.371	0.013	0.013	0.013	0.013	0.013	0.756	0.558	0.756	0.611	0.009	0.065	0.605	0.780	0.009	0.052	0.009	0.067
Q64240	Protein AMBP OS=Rattus norvegicus GN=Ambp PE=1 SV=1 - [AMBP_RAT]	38.8	0.903	0.561	0.695	0.314	0.354	0.853	1.073	0.936	0.718	1.185	0.308	0.566	0.795	1.111	0.163	0.374	0.618	0.653	0.394	0.673	0.036	0.0790	0.917	0.699	0.917	0.991	0.127	0.402	0.014	0.022	0.917	0.860
Q64268	Heparin cofactor 2 OS=Rattus norvegicus GN=Serpind1 PE=1 SV=1 - [HEP2_RAT]	54.5	0.013	0.013	0.013	0.013	0.013	0.911	1.047	1.195	0.485	1.256	0.434	0.614	0.678	0.795	0.181	0.013	0.013	0.013	0.013	0.013	0.009	0.0266	0.009	0.549	#DIV/0!	#DIV/0!	0.056	0.200	0.009	0.018	0.009	0.715
Q64270	Translation initiation factor eIF-2B subunit alpha OS=Rattus norvegicus GN=Eif2b1 PE=2 SV=1 - [EI2BA_RAT]	33.7	0.013	0.013	0.013	0.013	0.013	0.013	0.013	0.013	0.013	0.013	0.013	0.013	0.013	0.013	0.013	0.499	1.049	1.154	0.642	1.064	#DIV/0!	#DIV/0!	#DIV/0!	#DIV/0!	0.009	0.060	#DIV/0!	#DIV/0!	0.009	0.023	0.009	0.066
Q64319	Neutral and basic amino acid transport protein rBAT OS=Rattus norvegicus GN=Slc3a1 PE=1 SV=1 - [SLC31_RAT]	78.5	0.013	0.013	0.013	0.013	0.013	1.493	1.530	1.280	1.931	1.534	0.013	0.013	0.013	0.013	0.013	0.013	0.013	0.013	0.013	0.013	0.009	0.0215	#DIV/0!	#DIV/0!	#DIV/0!	#DIV/0!	0.009	0.036	0.009	0.015	#DIV/0!	#DIV/0!
Q64602	Kynurenine/alpha-aminoadipate aminotransferase, mitochondrial OS=Rattus norvegicus OX=10116 GN=Aadat PE=1 SV=1 - [AADAT_RAT]	47.8	0.013	0.013	0.013	0.013	0.013	0.841	0.896	0.848	0.377	0.742	0.013	0.013	0.013	0.013	0.013	0.013	0.013	0.013	0.013	0.013	0.009	0.0670	#DIV/0!	#DIV/0!	#DIV/0!	#DIV/0!	0.009	0.153	0.009	0.043	#DIV/0!	#DIV/0!
Q66H12	Alpha-N-acetylgalactosaminidase OS=Rattus norvegicus GN=Naga PE=2 SV=1 - [NAGAB_RAT]	46.8	1.310	0.886	1.545	0.560	0.526	0.853	0.878	1.066	0.339	0.703	0.415	0.629	0.775	0.852	0.279	0.013	0.013	0.013	0.013	0.013	0.470	0.445	0.184	0.372	0.009	0.052	0.184	0.520	0.009	0.035	0.009	0.390
Q66HB6	Cancer-associated gene 1 protein homolog OS=Rattus norvegicus GN=Cage1 PE=2 SV=1 - [CAGE1_RAT]	97.4	0.013	0.013	0.013	0.013	0.013	0.013	0.013	0.013	0.013	0.013	0.013	0.013	0.013	0.013	0.013	0.440	1.110	0.934	0.787	0.832	#DIV/0!	#DIV/0!	#DIV/0!	#DIV/0!	0.009	0.072	#DIV/0!	#DIV/0!	0.009	0.028	0.009	0.089
Q66HF1	NADH-ubiquinone oxidoreductase 75 kDa subunit, mitochondrial OS=Rattus norvegicus GN=Ndufs1 PE=1 SV=1 - [NDUS1_RAT]	79.4	0.668	0.696	0.884	0.377	0.429	0.773	0.955	1.203	0.126	0.735	0.469	0.627	0.834	1.490	0.224	0.013	0.013	0.013	0.013	0.013	0.259	0.380	0.917	0.691	0.009	0.204	0.756	0.819	0.009	0.038	0.009	0.179
Q66HG4	Aldose 1-epimerase OS=Rattus norvegicus OX=10116 GN=Galm PE=1 SV=1 - [GALM_RAT]	37.9	0.702	0.536	1.048	0.513	0.311	0.586	0.698	0.770	0.532	0.534	0.222	0.488	0.547	0.764	0.144	0.171	0.371	0.305	0.187	0.415	0.756	0.578	0.354	0.557	0.036	0.156	0.184	0.531	0.014	0.022	0.354	0.920
Q68FS2	COP9 signalosome complex subunit 4 OS=Rattus norvegicus GN=Cops4 PE=1 SV=1 - [CSN4_RAT]	46.3	0.395	0.576	1.062	0.193	0.431	0.982	1.166	1.334	1.247	1.071	0.542	0.932	1.001	1.350	0.321	0.343	0.516	0.712	0.388	0.912	0.022	0.0513	0.354	0.535	0.917	0.992	0.184	0.455	0.014	0.022	0.259	0.766
Q68FW9	COP9 signalosome complex subunit 3 OS=Rattus norvegicus GN=Cops3 PE=2 SV=1 - [CSN3_RAT]	47.8	0.013	0.013	0.013	0.013	0.013	0.754	1.274	1.187	0.440	1.027	0.383	0.774	0.804	1.084	0.176	0.013	0.013	0.013	0.013	0.013	0.009	0.0292	0.009	0.324	#DIV/0!	#DIV/0!	0.259	0.607	0.009	0.021	0.009	0.318
Q68FX1	Mannose-6-phosphate isomerase OS=Rattus norvegicus GN=Mpi PE=2 SV=1 - [MPI_RAT]	46.4	0.013	0.013	0.013	0.013	0.013	1.155	1.649	1.639	0.359	1.493	0.534	0.913	0.947	1.165	0.242	0.013	0.013	0.013	0.013	0.013	0.009	0.0221	0.009	0.117	#DIV/0!	#DIV/0!	0.127	0.373	0.009	0.015	0.009	0.136
Q6AXM8	Serum paraoxonase/arylesterase 2 OS=Rattus norvegicus OX=10116 GN=Pon2 PE=2 SV=1 - [PON2_RAT]	39.6	0.013	0.013	0.013	0.013	0.013	0.872	1.055	1.180	0.535	0.957	0.013	0.013	0.013	0.013	0.013	0.013	0.013	0.013	0.013	0.013	0.009	0.0313	#DIV/0!	#DIV/0!	#DIV/0!	#DIV/0!	0.009	0.059	0.009	0.022	#DIV/0!	#DIV/0!
Q6AXS5	Plasminogen activator inhibitor 1 RNA-binding protein OS=Rattus norvegicus OX=10116 GN=Serbp1 PE=1 SV=2 - [PAIRB_RAT]	44.7	0.868	0.648	1.527	0.170	0.539	0.013	0.013	0.013	0.013	0.013	0.985	0.780	1.242	1.727	0.404	0.830	0.941	1.571	0.729	0.968	0.009	0.0616	0.354	0.465	0.184	0.529	0.009	0.045	0.009	0.018	0.756	0.724
Q6AXY0	Glutathione S-transferase A6 OS=Rattus norvegicus GN=Gsta6 PE=1 SV=1 - [GSTA6_RAT]	25.8	1.049	0.754	1.442	0.691	0.819	0.816	1.056	1.139	0.295	0.981	0.565	0.921	0.915	1.362	0.351	0.013	0.013	0.013	0.013	0.013	0.917	0.560	0.605	0.581	0.009	0.052	0.756	0.760	0.009	0.025	0.009	0.088
Q6AY19	Atypical kinase COQ8B, mitochondrial OS=Rattus norvegicus OX=10116 GN=Coq8b PE=1 SV=1 - [COQ8B_RAT]	58.9	0.651	0.498	1.710	0.713	0.702	0.013	0.013	0.013	0.013	0.013	0.939	0.832	0.888	1.260	0.932	0.763	0.797	1.524	0.825	0.823	0.009	0.0365	0.127	0.278	0.127	0.425	0.009	0.050	0.009	0.020	0.127	0.428
Q6AY56	Tubulin alpha-8 chain OS=Rattus norvegicus GN=Tuba8 PE=2 SV=1 - [TBA8_RAT]	50.0	1.203	0.967	1.347	0.560	0.883	0.872	0.653	1.028	0.276	0.608	1.220	1.254	1.463	1.295	0.694	0.896	1.405	1.889	1.456	1.166	0.184	0.290	0.259	0.414	0.127	0.418	0.036	0.130	0.022	0.033	0.605	0.720
Q6AY80	Ribosyldihydronicotinamide dehydrogenase [quinone] OS=Rattus norvegicus OX=10116 GN=Nqo2 PE=1 SV=3 - [NQO2_RAT]	26.3	0.013	0.013	0.013	0.013	0.013	0.646	1.059	1.316	0.207	0.782	0.013	0.013	0.013	0.013	0.013	0.013	0.013	0.013	0.013	0.013	0.009	0.0484	#DIV/0!	#DIV/0!	#DIV/0!	#DIV/0!	0.009	0.101	0.009	0.031	#DIV/0!	#DIV/0!
Q6AYC8	SH2 domain-containing protein 4A OS=Rattus norvegicus OX=10116 GN=Sh2d4a PE=2 SV=1 - [SH24A_RAT]	48.5	0.013	0.013	0.013	0.013	0.013	0.881	0.831	1.375	0.053	0.792	0.013	0.013	0.013	0.013	0.013	0.013	0.013	0.013	0.013	0.013	0.009	0.0517	#DIV/0!	#DIV/0!	#DIV/0!	#DIV/0!	0.009	0.113	0.009	0.032	#DIV/0!	#DIV/0!
Q6EV70	GDP-fucose protein O-fucosyltransferase 1 OS=Rattus norvegicus GN=Pofut1 PE=2 SV=1 - [OFUT1_RAT]	45.0	0.488	0.671	0.925	0.174	0.333	0.851	1.081	1.282	0.270	0.858	0.013	0.013	0.013	0.013	0.013	0.013	0.013	0.013	0.013	0.013	0.184	0.305	0.009	0.713	0.009	0.417	0.009	0.072	0.009	0.024	#DIV/0!	#DIV/0!
Q6IFW6	Keratin, type I cytoskeletal 10 OS=Rattus norvegicus GN=Krt10 PE=3 SV=1 - [K1C10_RAT]	56.5	0.600	0.630	0.968	0.725	0.643	0.926	1.243	0.834	0.679	1.085	0.618	0.782	0.938	1.128	0.444	0.360	0.352	0.523	0.489	0.701	0.085	0.168	0.756	0.654	0.056	0.240	0.354	0.624	0.022	0.034	0.085	0.304
Q6IUU3	Sulfhydryl oxidase 1 OS=Rattus norvegicus GN=Qsox1 PE=1 SV=1 - [QSOX1_RAT]	82.4	0.803	0.569	1.072	0.452	0.605	0.013	0.013	0.013	0.013	0.013	0.013	0.013	0.013	0.013	0.013	0.435	0.677	0.695	0.483	0.861	0.009	0.0859	0.009	0.188	0.756	0.960	#DIV/0!	#DIV/0!	0.009	0.091	0.009	0.343
Q6P686	Osteoclast-stimulating factor 1 OS=Rattus norvegicus GN=Ostf1 PE=1 SV=1 - [OSTF1_RAT]	23.7	0.075	0.296	0.291	0.290	0.217	0.725	1.118	1.071	0.254	0.931	0.311	0.564	0.712	1.049	0.181	0.013	0.013	0.013	0.013	0.013	0.056	0.120	0.085	0.204	0.009	2.919	0.184	0.518	0.009	0.028	0.009	0.452
Q6P6R2	Dihydrolipoyl dehydrogenase, mitochondrial OS=Rattus norvegicus GN=Dld PE=1 SV=1 - [DLDH_RAT]	54.0	0.995	0.928	1.102	0.662	0.543	0.954	1.302	1.259	0.802	1.032	0.489	0.917	0.776	1.076	0.253	0.390	0.768	0.778	0.738	0.805	0.184	0.281	0.354	0.507	0.354	0.781	0.085	0.279	0.022	0.034	0.756	0.858
Q6P6S0	Synaptosomal-associated protein 47 OS=Rattus norvegicus OX=10116 GN=Snap47 PE=2 SV=1 - [SNP47_RAT]	47.0	0.013	0.013	0.013	0.013	0.013	1.008	1.170	1.224	0.915	1.064	0.013	0.013	0.013	0.013	0.013	0.013	0.013	0.013	0.013	0.013	0.009	0.0234	#DIV/0!	#DIV/0!	#DIV/0!	#DIV/0!	0.009	0.042	0.009	0.017	#DIV/0!	#DIV/0!
Q6P6S4	Nucleotide exchange factor SIL1 OS=Rattus norvegicus GN=Sil1 PE=2 SV=1 - [SIL1_RAT]	52.3	0.782	0.741	1.400	0.610	0.648	0.838	0.912	1.189	0.188	0.923	0.992	0.990	0.842	1.282	0.409	0.013	0.013	0.013	0.013	0.013	0.470	0.460	0.470	0.539	0.009	0.068	0.470	0.690	0.009	0.030	0.009	0.060
Q6PDU7	ATP synthase subunit g, mitochondrial OS=Rattus norvegicus OX=10116 GN=Atp5mg PE=1 SV=2 - [ATP5L_RAT]	11.4	0.013	0.013	0.013	0.013	0.013	0.556	0.966	0.815	0.137	0.717	0.211	0.780	0.998	1.185	0.064	0.013	0.013	0.013	0.013	0.013	0.009	0.156	0.009	0.310	#DIV/0!	#DIV/0!	0.756	0.829	0.009	0.087	0.009	0.306
Q6SKG1	Acyl-coenzyme A synthetase ACSM3, mitochondrial OS=Rattus norvegicus OX=10116 GN=Acsm3 PE=2 SV=1 - [ACSM3_RAT]	65.7	0.013	0.013	0.013	0.013	0.013	1.024	1.008	1.425	0.045	0.808	0.013	0.013	0.013	0.013	0.013	0.013	0.013	0.013	0.013	0.013	0.009	0.0357	#DIV/0!	#DIV/0!	#DIV/0!	#DIV/0!	0.009	0.073	0.009	0.024	#DIV/0!	#DIV/0!
Q7TP48	Adipocyte plasma membrane-associated protein OS=Rattus norvegicus OX=10116 GN=Apmap PE=2 SV=2 - [APMAP_RAT]	42.0	0.013	0.013	0.013	0.013	0.013	0.543	1.099	0.918	0.252	0.983	0.244	0.621	0.608	1.177	0.076	0.013	0.013	0.013	0.013	0.013	0.009	0.0581	0.009	0.509	#DIV/0!	#DIV/0!	0.470	0.766	0.009	0.037	0.009	0.660
Q8CGS5	Zinc phosphodiesterase ELAC protein 2 OS=Rattus norvegicus GN=Elac2 PE=1 SV=1 - [RNZ2_RAT]	92.3	0.013	0.013	0.013	0.013	0.013	1.180	1.070	1.343	0.348	0.888	0.622	0.533	0.871	1.289	0.216	0.013	0.013	0.013	0.013	0.013	0.009	0.0276	0.009	0.174	#DIV/0!	#DIV/0!	0.259	0.579	0.009	0.019	0.009	0.226
Q8K4D8	Aldehyde dehydrogenase family 1 member A3 OS=Rattus norvegicus GN=Aldh1a3 PE=2 SV=1 - [AL1A3_RAT]	56.1	0.013	0.013	0.013	0.013	0.013	1.024	1.256	1.278	0.495	1.171	0.503	0.872	0.993	1.192	0.222	0.013	0.013	0.013	0.013	0.013	0.009	0.0244	0.009	0.121	#DIV/0!	#DIV/0!	0.184	0.465	0.009	0.017	0.009	0.141
Q8K581	Thioredoxin domain-containing protein 9 OS=Rattus norvegicus GN=Txndc9 PE=2 SV=2 - [TXND9_RAT]	26.3	0.013	0.013	0.013	0.013	0.013	0.585	0.792	1.172	0.013	0.494	0.013	0.013	0.013	0.013	0.013	0.013	0.013	0.013	0.013	0.013	0.025	0.0561	#DIV/0!	#DIV/0!	#DIV/0!	#DIV/0!	0.025	0.093	0.025	0.037	#DIV/0!	#DIV/0!
Q8VD87	Ribonuclease pancreatic gamma-type OS=Rattus rattus OX=10117 PE=3 SV=1 - [RNS1G_RATRT]	16.5	0.013	0.013	0.013	0.013	0.013	0.574	0.677	0.958	0.780	0.785	0.013	0.013	0.013	0.013	0.013	0.013	0.013	0.013	0.013	0.013	0.009	0.0592	#DIV/0!	#DIV/0!	#DIV/0!	#DIV/0!	0.009	0.143	0.009	0.039	#DIV/0!	#DIV/0!
Q8VIF7	Selenium-binding protein 1 OS=Rattus norvegicus GN=Selenbp1 PE=1 SV=1 - [SBP1_RAT]	52.5	0.982	0.864	1.019	0.507	0.480	0.944	1.129	1.293	0.943	0.937	0.498	0.738	0.838	1.243	0.241	0.461	0.747	0.667	0.431	0.880	0.184	0.282	0.605	0.632	0.259	0.685	0.085	0.280	0.014	0.022	0.756	0.865
Q99ND9	RWD domain-containing protein 1 OS=Rattus norvegicus GN=Rwdd1 PE=2 SV=1 - [RWDD1_RAT]	27.8	1.008	0.911	1.855	0.968	0.844	0.959	0.955	1.185	0.295	0.687	0.919	1.154	0.906	1.140	0.623	0.013	0.013	0.013	0.013	0.013	0.354	0.406	0.756	0.583	0.009	0.044	0.917	0.741	0.009	0.028	0.009	0.049
Q9EQ10	PCNA-interacting partner OS=Rattus norvegicus OX=10116 GN=Parpbp PE=1 SV=2 - [PARI_RAT]	63.9	1.894	1.205	3.098	0.572	1.184	0.013	0.013	0.013	0.013	0.013	1.457	1.720	0.621	0.685	0.909	0.792	2.016	1.888	1.788	1.250	0.009	0.0215	0.470	0.509	0.756	0.830	0.009	0.042	0.009	0.015	0.127	0.422
Q9EST6	Acidic leucine-rich nuclear phosphoprotein 32 family member B OS=Rattus norvegicus GN=Anp32b PE=2 SV=1 - [AN32B_RAT]	31.0	1.052	0.819	1.601	0.819	0.735	0.013	0.013	0.013	0.013	0.013	0.905	0.886	1.352	1.990	0.606	1.040	0.900	1.520	0.696	0.839	0.009	0.0256	0.605	0.554	0.917	0.847	0.009	0.040	0.009	0.018	0.756	0.719
Q9HB97	Alpha-parvin OS=Rattus norvegicus GN=Parva PE=1 SV=2 - [PARVA_RAT]	42.3	0.013	0.013	0.013	0.013	0.013	0.847	1.128	1.151	0.609	0.950	0.013	0.013	0.013	0.013	0.013	0.013	0.013	0.013	0.013	0.013	0.009	0.0290	#DIV/0!	#DIV/0!	#DIV/0!	#DIV/0!	0.009	0.054	0.009	0.021	#DIV/0!	#DIV/0!
Q9JHU5	Arfaptin-1 OS=Rattus norvegicus GN=Arfip1 PE=1 SV=1 - [ARFP1_RAT]	40.8	1.313	1.231	1.939	1.161	1.049	0.984	0.595	1.104	0.138	0.421	1.100	1.620	1.071	1.041	0.712	0.872	1.221	1.551	1.229	1.259	0.022	0.0505	0.184	0.347	0.756	0.831	0.127	0.386	0.036	0.051	0.354	0.748
Q9JIX3	Bis(5'-adenosyl)-triphosphatase OS=Rattus norvegicus OX=10116 GN=Fhit PE=1 SV=1 - [FHIT_RAT]	17.3	0.013	0.013	0.013	0.013	0.013	0.669	1.186	0.947	0.171	1.048	0.599	0.806	0.813	1.138	0.356	0.013	0.013	0.013	0.013	0.013	0.009	0.0480	0.009	0.132	#DIV/0!	#DIV/0!	0.605	0.793	0.009	0.031	0.009	0.153
Q9JJ19	Na(+)/H(+) exchange regulatory cofactor NHE-RF1 OS=Rattus norvegicus OX=10116 GN=Slc9a3r1 PE=1 SV=3 - [NHRF1_RAT]	38.8	0.013	0.013	0.013	0.013	0.013	0.694	0.450	0.870	0.013	0.508	0.013	0.013	0.013	0.013	0.013	0.013	0.013	0.013	0.013	0.013	0.025	0.0563	#DIV/0!	#DIV/0!	#DIV/0!	#DIV/0!	0.025	0.094	0.025	0.037	#DIV/0!	#DIV/0!
Q9JJ22	Endoplasmic reticulum aminopeptidase 1 OS=Rattus norvegicus GN=Erap1 PE=2 SV=2 - [ERAP1_RAT]	106.4	1.018	0.740	1.510	0.690	0.698	0.013	0.013	0.013	0.013	0.013	0.448	0.781	0.984	1.097	0.314	0.390	0.697	0.763	0.611	0.854	0.009	0.0299	0.605	0.592	0.259	0.655	0.009	0.178	0.009	0.073	0.605	0.870
Q9JJ31	Cullin-5 OS=Rattus norvegicus GN=Cul5 PE=1 SV=3 - [CUL5_RAT]	90.8	0.013	0.013	0.013	0.013	0.013	0.751	1.162	1.088	0.222	0.995	0.013	0.013	0.013	0.013	0.013	0.013	0.013	0.013	0.013	0.013	0.009	0.0386	#DIV/0!	#DIV/0!	#DIV/0!	#DIV/0!	0.009	0.081	0.009	0.026	#DIV/0!	#DIV/0!
Q9JJP9	Ubiquilin-1 OS=Rattus norvegicus GN=Ubqln1 PE=1 SV=1 - [UBQL1_RAT]	62.0	1.143	0.665	1.064	0.384	0.563	0.860	1.518	1.280	0.594	2.039	0.013	0.013	0.013	0.013	0.013	0.013	0.013	0.013	0.013	0.013	0.127	0.218	0.009	0.115	0.009	0.088	0.009	0.038	0.009	0.016	#DIV/0!	#DIV/0!
Q9QZ81	Protein argonaute-2 OS=Rattus norvegicus GN=Ago2 PE=2 SV=2 - [AGO2_RAT]	97.3	0.013	0.013	0.013	0.013	0.013	1.140	1.200	1.095	0.334	1.087	0.013	0.013	0.013	0.013	0.013	0.013	0.013	0.013	0.013	0.013	0.009	0.0271	#DIV/0!	#DIV/0!	#DIV/0!	#DIV/0!	0.009	0.049	0.009	0.019	#DIV/0!	#DIV/0!
Q9QZK8	Deoxyribonuclease-2-alpha OS=Rattus norvegicus GN=Dnase2 PE=2 SV=1 - [DNS2A_RAT]	38.2	0.013	0.013	0.013	0.013	0.013	0.013	0.013	0.013	0.013	0.013	0.013	0.013	0.013	0.013	0.013	0.472	1.316	1.013	0.863	1.082	#DIV/0!	#DIV/0!	#DIV/0!	#DIV/0!	0.009	0.053	#DIV/0!	#DIV/0!	0.009	0.020	0.009	0.049
Q9R0D6	Transcobalamin-2 OS=Rattus norvegicus GN=Tcn2 PE=2 SV=1 - [TCO2_RAT]	47.4	0.667	0.473	0.669	0.329	0.332	0.772	0.880	0.969	1.006	1.007	0.013	0.013	0.013	0.013	0.013	0.388	0.656	0.693	0.403	0.666	0.014	0.0323	0.009	0.792	0.605	0.952	0.009	0.057	0.014	0.022	0.009	0.477
Q9WTQ2	Podocalyxin OS=Rattus norvegicus OX=10116 GN=Podxl PE=1 SV=2 - [PODXL_RAT]	51.6	0.013	0.013	0.013	0.013	0.013	0.440	1.122	0.853	0.095	0.819	0.249	0.386	0.609	0.964	0.086	0.013	0.013	0.013	0.013	0.013	0.009	0.133	0.009	1.188	#DIV/0!	#DIV/0!	0.354	0.694	0.009	0.072	0.009	0.953
Q9WU82	Catenin beta-1 OS=Rattus norvegicus GN=Ctnnb1 PE=1 SV=1 - [CTNB1_RAT]	85.4	0.714	0.687	1.249	0.427	0.384	0.749	1.414	1.107	0.185	1.247	0.613	0.974	1.021	1.285	0.266	0.013	0.013	0.013	0.013	0.013	0.354	0.422	0.605	0.622	0.009	0.122	0.605	0.702	0.009	0.020	0.009	0.083
Q9WVR7	Protein phosphatase 1F OS=Rattus norvegicus GN=Ppm1f PE=2 SV=1 - [PPM1F_RAT]	49.1	0.013	0.013	0.013	0.013	0.013	0.836	1.224	1.662	0.110	1.091	0.297	0.529	1.005	1.424	0.167	0.013	0.013	0.013	0.013	0.013	0.009	0.0265	0.009	0.216	#DIV/0!	#DIV/0!	0.470	0.708	0.009	0.018	0.009	0.268
Q9Z0V5	Peroxiredoxin-4 OS=Rattus norvegicus GN=Prdx4 PE=2 SV=1 - [PRDX4_RAT]	31.0	0.968	0.715	1.326	0.445	0.619	0.956	1.415	1.082	0.904	1.188	0.529	0.772	0.834	1.127	0.263	0.471	0.818	0.694	0.544	0.945	0.184	0.280	0.756	0.647	0.605	0.894	0.056	0.195	0.022	0.034	0.917	0.842

Q9Z1X1	Extended synaptotagmin-1 OS=Rattus norvegicus GN=Esyt1 PE=1 SV=1 - [ESYT1_RAT]	121.1	0.640	0.705	1.121	0.487	0.447	0.013	0.013	0.013	0.013	0.013	0.504	0.895	0.682	1.399	0.323	0.455	0.519	1.079	0.634	0.733	0.009	0.109	0.756	0.664	0.917	0.961	0.009	0.138	0.009	0.063	0.917	0.835
Q9Z2G8	Nucleosome assembly protein 1-like 1 OS=Rattus norvegicus GN=Nap1l1 PE=2 SV=1 - [NP1L1_RAT]	45.3	0.807	0.794	0.692	0.340	0.294	0.727	1.323	1.347	0.339	1.141	0.512	0.928	1.068	1.924	0.279	0.013	0.013	0.013	0.013	0.013	0.184	0.296	0.354	0.514	0.009	0.243	0.605	0.678	0.009	0.019	0.009	0.051
Q9Z2L0	Voltage-dependent anion-selective channel protein 1 OS=Rattus norvegicus OX=10116 GN=Vdac1 PE=1 SV=4 - [VDAC1_RAT]	30.7	0.013	0.013	0.013	0.013	0.013	0.879	0.871	1.112	0.702	0.630	0.013	0.013	0.013	0.013	0.013	0.013	0.013	0.013	0.013	0.013	0.009	0.0396	#DIV/0!	#DIV/0!	#DIV/0!	#DIV/0!	0.009	0.083	0.009	0.026	#DIV/0!	#DIV/0!
Q9Z2M4	Peroxisomal 2,4-dienoyl-CoA reductase OS=Rattus norvegicus OX=10116 GN=Decr2 PE=1 SV=1 - [DECR2_RAT]	31.3	0.013	0.013	0.013	0.013	0.013	0.013	0.013	0.013	0.013	0.013	0.013	0.013	0.013	0.013	0.013	0.532	0.758	0.884	0.575	0.979	#DIV/0!	#DIV/0!	#DIV/0!	#DIV/0!	0.009	0.093	#DIV/0!	#DIV/0!	0.009	0.041	0.009	0.148

Table S6. Extracted protein profile of d-III (Effect of FGR, only Poor nutrition, Female)

			Expression level																															
Accession	Description	MW [kDa]	FGR-Good-Female					FGR-Poor_Female					Sham-Good-Female					Sham-Poor-Female					Steel-Dwass & FDR											
			#01	#02	#03	#04	#05	#06	#07	#08	#09	#10	#11	#12	#13	#14	#15	#16	#17	#18	#19	#20	FGR-GN vs FGR-PN		FGR-GN vs Sham-GN		FGR-GN vs Sham-PN		FGR-PN vs Sham-GN		FGR-PN vs Sham-PN		Sham-GN vs Sham-PN	
Filter	Filter	Filter	Filter	Filter	Filter	Filter	Filter	Filter	Filter	Filter	Filter	Filter	Filter	Filter	Filter	Filter	Filter	Filter	Filter	Filter	Filter	Filter	p-value	q-value	p	q-value	p	q-value	p	q-value	p	q-value	p	q-value
A1L1L2	Transmembrane protein 214 OS=Rattus norvegicus GN=Tmem214 PE=2 SV=1 - [TM214_RAT]	76.5	0.013	0.013	0.013	0.013	0.013	3.403	0.821	0.685	0.733	0.637	1.101	1.069	1.453	0.648	0.905	0.013	0.013	0.013	0.013	0.013	0.009	0.052	0.009	0.111	#DIV/0!	#DIV/0!	0.354	0.275	0.009	0.049	0.009	0.275
A2VCX1	TIP41-like protein OS=Rattus norvegicus GN=Tiprl PE=1 SV=1 - [TIPRL_RAT]	31.2	0.821	0.697	0.829	1.151	1.626	1.339	1.284	1.384	1.020	2.694	0.524	0.687	0.969	0.597	2.900	0.013	0.013	0.013	0.013	0.013	0.127	0.545	0.354	0.345	0.009	0.101	0.127	0.115	0.009	0.031	0.009	0.174
B0BN85	Protein SGT1 homolog OS=Rattus norvegicus GN=Sugt1 PE=2 SV=1 - [SGT1_RAT]	38.1	0.628	0.940	0.504	1.110	1.085	0.951	0.483	0.577	1.139	1.045	0.984	1.070	2.335	1.349	1.195	1.298	0.999	1.498	1.676	1.495	0.917	0.977	0.085	0.112	0.036	0.083	0.056	0.072	0.036	0.070	0.470	1.497
B0BN93	26S proteasome non-ATPase regulatory subunit 13 OS=Rattus norvegicus GN=Psmd13 PE=1 SV=1 - [PSD13_RAT]	42.8	0.973	0.941	0.787	1.189	1.036	0.013	0.013	0.013	0.013	0.013	0.978	1.078	1.601	1.895	1.079	2.350	0.566	1.545	1.415	1.338	0.009	0.064	0.085	0.111	0.127	0.159	0.009	0.055	0.009	0.036	0.756	1.317
B0K020	CDGSH iron-sulfur domain-containing protein 1 OS=Rattus norvegicus OX=10116 GN=Cisd1 PE=3 SV=1 - [CISD1_RAT]	12.1	1.050	0.892	0.612	1.151	0.918	0.013	0.013	0.013	0.013	0.013	0.903	0.765	1.465	1.980	0.701	2.262	0.861	1.275	1.198	0.589	0.009	0.074	0.756	0.652	0.470	0.437	0.009	0.071	0.009	0.051	0.917	1.241
B2GV24	E3 UFM1-protein ligase 1 OS=Rattus norvegicus GN=Ufl1 PE=1 SV=1 - [UFL1_RAT]	89.5	1.374	0.775	0.949	1.239	1.009	0.013	0.013	0.013	0.013	0.013	0.741	0.791	1.258	1.016	0.880	1.128	0.960	1.378	1.083	0.393	0.009	0.056	0.470	0.440	0.917	0.768	0.009	0.144	0.009	0.099	0.470	1.826
B5DEN9	Vacuolar protein sorting-associated protein 28 homolog OS=Rattus norvegicus GN=Vps28 PE=2 SV=1 - [VPS28_RAT]	26.1	0.013	0.013	0.013	0.013	0.013	0.013	0.013	0.013	0.013	0.013	0.013	0.013	0.013	0.013	0.013	2.536	0.826	2.513	2.492	1.648	#DIV/0!	#DIV/0!	#DIV/0!	#DIV/0!	0.009	0.030	#DIV/0!	#DIV/0!	0.009	0.023	0.009	0.069
B5DF91	ELAV-like protein 1 OS=Rattus norvegicus OX=10116 GN=Elavl1 PE=1 SV=1 - [ELAV1_RAT]	36.1	0.930	0.720	0.693	1.228	1.019	0.013	0.013	0.013	0.013	0.013	0.878	0.989	1.759	1.726	0.906	1.096	0.960	0.962	1.133	0.840	0.009	0.078	0.354	0.345	0.470	0.450	0.009	0.062	0.009	0.095	0.605	1.683
B5DFC9	Nidogen-2 OS=Rattus norvegicus GN=Nid2 PE=2 SV=1 - [NID2_RAT]	152.9	0.013	0.013	0.013	0.013	0.013	0.013	0.013	0.013	0.013	0.013	0.533	0.596	1.146	1.441	0.776	2.202	0.651	0.761	1.441	0.628	#DIV/0!	#DIV/0!	0.009	0.255	0.009	0.075	0.009	0.178	0.009	0.062	0.470	1.788
D3ZF77	Aldo-keto reductase family 1 member C15 OS=Rattus norvegicus OX=10116 GN=Akr1c15 PE=1 SV=1 - [AK1CA_RAT]	37.2	0.736	1.090	0.735	1.198	0.994	0.013	0.013	0.013	0.013	0.013	0.741	0.485	2.207	0.840	1.356	1.169	0.955	1.314	1.322	1.635	0.009	0.070	0.756	0.653	0.085	0.123	0.009	0.075	0.009	0.048	0.470	1.682
D3ZVR7	Prostamide/prostaglandin F synthase OS=Rattus norvegicus OX=10116 GN=Prxl2b PE=3 SV=1 - [PXL2B_RAT]	21.6	0.013	0.013	0.013	0.013	0.013	0.013	0.013	0.013	0.013	0.013	0.672	0.608	1.171	1.651	0.807	1.618	1.239	1.007	0.927	0.517	#DIV/0!	#DIV/0!	0.009	0.154	0.009	0.088	0.009	0.123	0.009	0.074	0.756	1.579
D4ABX8	Leucine-rich repeat and fibronectin type-III domain-containing protein 4 OS=Rattus norvegicus OX=10116 GN=Lrfn4 PE=1 SV=1 - [LRFN4_RAT]	67.2	0.013	0.013	0.013	0.013	0.013	1.495	1.151	1.173	0.643	0.632	0.013	0.013	0.013	0.013	0.013	0.013	0.013	0.013	0.013	0.013	0.009	0.060	#DIV/0!	#DIV/0!	#DIV/0!	#DIV/0!	0.009	0.108	0.009	0.084	#DIV/0!	#DIV/0!
F1LNI5	Protein phosphatase 1G OS=Rattus norvegicus GN=Ppm1g PE=1 SV=2 - [PPM1G_RAT]	58.7	0.488	1.145	0.419	1.101	0.998	0.013	0.013	0.013	0.013	0.013	1.120	0.975	2.123	1.328	1.021	1.301	0.850	1.660	1.625	1.639	0.009	0.146	0.184	0.206	0.056	0.098	0.009	0.055	0.009	0.037	0.605	1.481
G3V6U9	Actin-histidine N-methyltransferase OS=Rattus norvegicus OX=10116 GN=Setd3 PE=1 SV=1 - [SETD3_RAT]	67.4	0.650	1.136	0.358	1.528	1.390	0.013	0.013	0.013	0.013	0.013	0.013	0.013	0.013	0.013	0.013	2.560	0.956	1.521	1.607	1.468	0.009	0.061	0.009	0.129	0.127	0.157	#DIV/0!	#DIV/0!	0.009	0.028	0.009	0.084
O08730	Glycogenin-1 OS=Rattus norvegicus GN=Gyg1 PE=2 SV=4 - [GLYG_RAT]	37.4	0.013	0.013	0.013	0.013	0.013	0.808	0.465	0.668	0.658	0.998	0.013	0.013	0.013	0.013	0.013	1.555	1.086	1.070	1.115	1.147	0.009	0.431	#DIV/0!	#DIV/0!	0.009	0.067	0.009	0.910	0.014	0.033	0.009	0.156
O35094	Mitochondrial import inner membrane translocase subunit TIM44 OS=Rattus norvegicus GN=Timm44 PE=1 SV=1 - [TIM44_RAT]	51.0	0.434	1.158	0.386	0.986	0.926	0.013	0.013	0.013	0.013	0.013	0.894	0.921	1.798	1.871	0.968	2.379	0.601	1.462	1.250	1.148	0.009	0.245	0.354	0.349	0.085	0.127	0.009	0.057	0.009	0.042	0.756	1.354
O35142	Coatomer subunit beta' OS=Rattus norvegicus GN=Copb2 PE=1 SV=3 - [COPB2_RAT]	102.5	1.294	0.877	0.920	1.026	0.979	1.469	0.312	0.603	0.694	0.756	0.928	0.937	2.064	1.627	0.902	1.850	0.910	2.389	1.598	1.295	0.127	0.573	0.605	0.533	0.085	0.117	0.056	0.075	0.036	0.069	0.470	1.471
O35828	Coronin-7 OS=Rattus norvegicus GN=Coro7 PE=1 SV=2 - [CORO7_RAT]	100.7	1.036	0.757	0.750	0.946	0.985	0.013	0.013	0.013	0.013	0.013	0.873	0.936	1.737	2.028	1.187	2.887	0.418	1.549	1.331	1.457	0.009	0.095	0.184	0.202	0.127	0.159	0.009	0.052	0.009	0.031	0.756	1.271
O70593	Small glutamine-rich tetratricopeptide repeat-containing protein alpha OS=Rattus norvegicus GN=Sgta PE=1 SV=1 - [SGTA_RAT]	34.1	1.040	0.820	0.698	1.283	0.998	0.013	0.013	0.013	0.013	0.013	0.813	0.792	1.685	2.154	0.832	1.816	0.952	1.029	0.980	0.872	0.009	0.067	0.756	0.646	0.756	0.655	0.009	0.061	0.009	0.063	0.470	1.694
O88794	Pyridoxine-5'-phosphate oxidase OS=Rattus norvegicus OX=10116 GN=Pnpo PE=1 SV=1 - [PNPO_RAT]	30.2	0.768	0.582	0.726	1.128	0.683	0.901	0.601	0.756	0.952	0.757	0.869	0.972	1.592	1.342	1.070	1.493	1.057	1.231	1.161	0.874	0.605	1.060	0.056	0.084	0.036	0.091	0.036	0.057	0.036	0.077	0.917	1.254
O88941	Mannosyl-oligosaccharide glucosidase OS=Rattus norvegicus OX=10116 GN=Mogs PE=1 SV=1 - [MOGS_RAT]	91.8	0.835	1.007	0.667	1.157	0.791	1.223	0.440	0.516	0.968	0.717	0.927	1.008	1.941	1.804	0.896	1.528	0.842	2.269	1.795	1.328	0.470	1.042	0.127	0.151	0.036	0.083	0.127	0.123	0.036	0.070	0.756	1.278
P02651	Apolipoprotein A-IV OS=Rattus norvegicus GN=Apoa4 PE=1 SV=2 - [APOA4_RAT]	44.4	0.754	0.907	0.518	1.216	1.030	1.037	0.504	0.697	0.768	0.753	0.924	0.904	1.939	2.524	0.911	2.635	0.816	1.334	1.197	0.778	0.354	0.997	0.259	0.265	0.259	0.275	0.056	0.073	0.036	0.072	0.756	1.306
P02696	Retinol-binding protein 1 OS=Rattus norvegicus GN=Rbp1 PE=1 SV=2 - [RET1_RAT]	15.8	0.332	1.077	0.272	0.750	0.796	0.013	0.013	0.013	0.013	0.013	0.606	0.673	1.274	4.527	0.772	3.299	0.619	0.987	0.826	0.762	0.009	0.980	0.354	0.343	0.259	0.285	0.009	0.038	0.009	0.046	0.917	1.087
P02803	Metallothionein-1 OS=Rattus norvegicus OX=10116 GN=Mt1 PE=1 SV=1 - [MT1_RAT]	6.0	0.013	0.013	0.013	0.013	0.013	0.013	0.013	0.013	0.013	0.013	0.013	0.013	0.013	0.013	0.013	4.654	0.905	0.550	0.500	0.451	#DIV/0!	#DIV/0!	#DIV/0!	#DIV/0!	0.009	0.048	#DIV/0!	#DIV/0!	0.009	0.038	0.009	0.102
P04166	Cytochrome b5 type B OS=Rattus norvegicus OX=10116 GN=Cyb5b PE=1 SV=2 - [CYB5B_RAT]	16.3	0.013	0.013	0.013	0.013	0.013	0.013	0.013	0.013	0.013	0.013	0.944	0.786	1.593	1.567	0.461	1.826	0.729	1.501	1.488	0.562	#DIV/0!	#DIV/0!	0.009	0.1005	0.009	0.063	0.009	0.089	0.009	0.053	0.917	1.273
P04631	Protein S100-B OS=Rattus norvegicus GN=S100b PE=1 SV=2 - [S100B_RAT]	10.7	0.013	0.013	0.013	0.013	0.013	0.398	0.109	0.013	1.323	1.298	0.013	0.013	0.013	0.013	0.013	0.013	0.013	0.013	0.013	0.013	0.025	0.136	#DIV/0!	#DIV/0!	#DIV/0!	#DIV/0!	0.025	0.040	0.025	0.056	#DIV/0!	#DIV/0!
P04897	Guanine nucleotide-binding protein G(i) subunit alpha-2 OS=Rattus norvegicus GN=Gnai2 PE=1 SV=3 - [GNAI2_RAT]	40.5	1.192	0.768	0.773	1.061	0.895	0.013	0.013	0.013	0.013	0.013	0.908	0.778	1.559	2.017	0.777	1.774	1.288	1.290	1.381	0.722	0.009	0.071	0.354	0.347	0.127	0.164	0.009	0.066	0.009	0.047	0.917	1.221
P05065	Fructose-bisphosphate aldolase A OS=Rattus norvegicus GN=Aldoa PE=1 SV=2 - [ALDOA_RAT]	39.3	0.619	0.763	0.541	0.876	0.819	0.912	0.467	0.705	0.739	0.855	0.700	0.733	1.542	2.502	0.860	2.380	0.780	1.023	0.954	0.990	0.917	1.050	0.259	0.272	0.036	0.091	0.354	0.279	0.036	0.077	0.605	1.571
P05544	Serine protease inhibitor A3L OS=Rattus norvegicus GN=Serpina3l PE=1 SV=3 - [SPA3L_RAT]	46.2	0.301	0.648	0.250	0.823	0.806	0.566	0.360	0.523	0.625	0.618	1.134	0.803	2.289	1.595	0.750	2.293	0.615	2.027	0.733	2.138	0.605	1.146	0.085	0.116	0.127	0.167	0.014	0.033	0.036	0.072	0.917	1.083
P07323	Gamma-enolase OS=Rattus norvegicus GN=Eno2 PE=1 SV=2 - [ENOG_RAT]	47.1	0.013	0.013	0.013	0.013	0.013	0.013	0.013	0.013	0.013	0.013	0.568	0.515	1.507	0.899	0.838	1.042	0.975	0.933	1.004	1.140	#DIV/0!	#DIV/0!	0.009	0.327	0.009	0.103	0.009	0.233	0.009	0.085	0.127	0.783
P07483	Fatty acid-binding protein, heart OS=Rattus norvegicus GN=Fabp3 PE=1 SV=2 - [FABPH_RAT]	14.8	0.737	0.710	0.498	0.962	0.938	0.013	0.013	0.013	0.013	0.013	0.347	0.460	0.683	5.137	0.579	5.126	0.537	0.706	0.656	0.521	0.009	0.284	0.259	0.268	0.470	0.434	0.009	0.046	0.009	0.032	0.605	1.410
P07896	Peroxisomal bifunctional enzyme OS=Rattus norvegicus OX=10116 GN=Ehhadh PE=1 SV=2 - [ECHP_RAT]	78.6	0.582	0.706	0.525	0.895	0.754	0.564	0.374	0.400	0.543	0.513	0.733	0.849	1.300	5.911	0.658	4.451	0.592	1.050	1.025	0.724	0.036	0.194	0.184	0.196	0.127	0.163	0.014	0.029	0.014	0.033	0.756	1.145
P09330	Ribose-phosphate pyrophosphokinase 2 OS=Rattus norvegicus GN=Prps2 PE=1 SV=3 - [PRPS2_RAT]	34.8	0.013	0.013	0.013	0.013	0.013	0.013	0.013	0.013	0.013	0.013	0.013	0.013	0.013	0.013	0.013	2.437	0.859	1.317	1.036	0.968	#DIV/0!	#DIV/0!	#DIV/0!	#DIV/0!	0.009	0.055	#DIV/0!	#DIV/0!	0.009	0.045	0.009	0.122
P0C1X8	AP2-associated protein kinase 1 OS=Rattus norvegicus GN=Aak1 PE=1 SV=1 - [AAK1_RAT]	103.7	0.013	0.013	0.013	0.013	0.013	0.013	0.013	0.013	0.013	0.013	0.013	0.013	0.013	0.013	0.013	4.021	0.283	2.371	2.330	1.784	#DIV/0!	#DIV/0!	#DIV/0!	#DIV/0!	0.009	0.029	#DIV/0!	#DIV/0!	0.009	0.023	0.009	0.066
P11466	Peroxisomal carnitine O-octanoyltransferase OS=Rattus norvegicus GN=Crot PE=1 SV=3 - [OCTC_RAT]	70.3	0.013	0.013	0.013	0.013	0.013	0.013	0.013	0.013	0.013	0.013	0.013	0.013	0.013	0.013	0.013	1.376	1.072	1.387	1.496	1.414	#DIV/0!	#DIV/0!	#DIV/0!	#DIV/0!	0.009	0.054	#DIV/0!	#DIV/0!	0.009	0.043	0.009	0.114
P11762	Galectin-1 OS=Rattus norvegicus GN=Lgals1 PE=1 SV=2 - [LEG1_RAT]	14.8	0.825	0.699	0.531	0.970	0.928	0.713	0.542	0.722	0.815	0.775	0.774	0.778	1.975	0.852	1.002	0.870	0.858	1.018	1.039	1.153	0.470	1.096	0.354	0.353	0.085	0.133	0.056	0.077	0.014	0.034	0.259	1.391
P11960	2-oxoisovalerate dehydrogenase subunit alpha, mitochondrial (Fragment) OS=Rattus norvegicus OX=10116 GN=Bckdha PE=1 SV=1 - [ODBA_RAT]	50.1	0.696	0.838	0.697	1.141	0.969	1.219	0.486	0.766	0.937	0.911	0.905	1.121	1.910	1.482	1.223	1.397	0.946	1.451	1.260	1.120	0.917	0.963	0.056	0.082	0.056	0.113	0.085	0.092	0.036	0.072	0.756	1.397
P12369	cAMP-dependent protein kinase type II-beta regulatory subunit OS=Rattus norvegicus GN=Prkar2b PE=1 SV=3 - [KAP3_RAT]	46.1	0.013	0.013	0.013	0.013	0.013	0.013	0.013	0.013	0.013	0.013	0.013	0.013	0.013	0.013	0.013	1.538	1.430	1.324	1.821	1.164	#DIV/0!	#DIV/0!	#DIV/0!	#DIV/0!	0.009	0.045	#DIV/0!	#DIV/0!	0.009	0.035	0.009	0.096
P12749	60S ribosomal protein L26 OS=Rattus norvegicus GN=Rpl26 PE=1 SV=1 - [RL26_RAT]	17.3	1.050	0.895	0.786	1.735	0.866	0.013	0.013	0.013	0.013	0.013	0.013	0.013	0.013	0.013	0.013	0.535	1.178	1.884	1.117	0.414	0.009	0.056	0.009	0.101	0.917	0.764	#DIV/0!	#DIV/0!	0.009	0.080	0.009	0.300
P12928	Pyruvate kinase PKLR OS=Rattus norvegicus GN=Pklr PE=2 SV=2 - [KPYR_RAT]	62.2	0.013	0.013	0.013	0.013	0.013	0.013	0.013	0.013	0.013	0.013	0.013	0.013	0.013	0.013	0.013	2.277	1.008	1.058	0.923	0.825	#DIV/0!	#DIV/0!	#DIV/0!	#DIV/0!	0.009	0.064	#DIV/0!	#DIV/0!	0.009	0.053	0.009	0.147
P13437	3-ketoacyl-CoA thiolase, mitochondrial OS=Rattus norvegicus OX=10116 GN=Acaa2 PE=2 SV=1 - [THIM_RAT]	41.8	0.926	0.711	0.769	0.932	0.785	0.013	0.013	0.013	0.013	0.013	0.730	0.817	1.300	5.683	0.784	3.444	0.501	1.122	1.096	0.686	0.009	0.161	0.470	0.423	0.605	0.539	0.009	0.027	0.009	0.041	0.605	1.349
P14630	Apolipoprotein M OS=Rattus norvegicus GN=Apom PE=1 SV=2 - [APOM_RAT]	21.5	0.013	0.013	0.013	0.013	0.013	0.013	0.013	0.013	0.013	0.013	0.013	0.013	0.013	0.013	0.013	6.231	0.060	0.984	0.882	0.013	#DIV/0!	#DIV/0!	#DIV/0!	#DIV/0!	0.025	0.065	#DIV/0!	#DIV/0!	0.025	0.055	0.025	0.162
P14669	Annexin A3 OS=Rattus norvegicus GN=Anxa3 PE=1 SV=4 - [ANXA3_RAT]	36.3	0.722	0.841	0.651	1.014	0.937	0.973	0.540	0.823	0.852	0.930	0.931	0.885	1.918	1.335	1.097	1.245	0.924	1.170	0.989	1.194	0.917	0.989	0.085	0.115	0.056	0.126	0.056	0.075	0.036	0.077	0.917	1.253
P15429	Beta-enolase OS=Rattus norvegicus GN=Eno3 PE=1 SV=3 - [ENOB_RAT]	47.0	0.013	0.013	0.013	0.013	0.013	0.013	0.013	0.013	0.013	0.013	0.013	0.013	0.013	0.013	0.013	1.407	0.567	1.594	1.622	0.820	#DIV/0!	#DIV/0!	#DIV/0!	#DIV/0!	0.009	0.066	#DIV/0!	#DIV/0!	0.009	0.054	0.009	0.150
P18163	Long-chain-fatty-acid--CoA ligase 1 OS=Rattus norvegicus OX=10116 GN=Acsl1 PE=1 SV=1 - [ACSL1_RAT]	78.1	0.901	0.773	0.668	0.951	0.828	0.013	0.013	0.013	0.013	0.013	1.175	1.118	1.601	2.180	0.811	2.103	0.737	1.799	1.500	0.934	0.009	0.163	0.056	0.082	0.127	0.164	0.009	0.051	0.009	0.037	0.917	1.112
P18265	Glycogen synthase kinase-3 alpha OS=Rattus norvegicus GN=Gsk3a PE=1 SV=1 - [GSK3A_RAT]	51.0	1.049	1.124	0.881	1.433	1.117	0.013	0.013	0.013	0.013	0.013	0.987	0.850	2.088	0.810	1.171	0.565	1.432	1.111	0.996	0.986	0.009	0.053	0.605	0.533	0.354	0.354	0.009	0.068	0.009	0.086	0.917	1.289
P18395	Cold shock domain-containing protein E1 OS=Rattus norvegicus OX=10116 GN=Csde1 PE=2 SV=1 - [CSDE1_RAT]	88.8	1.166	0.952	0.954	1.063	0.811	0.013	0.013	0.013	0.013	0.013	1.088	1.116	1.877	1.976	0.651	1.453	0.744	1.370	1.172	0.523	0.009	0.064	0.259	0.264	0.605	0.545	0.009	0.053	0.009	0.075	0.605	1.627
P18665	39S ribosomal protein L3, mitochondrial OS=Rattus norvegicus GN=Mrpl3 PE=2 SV=1 - [RM03_RAT]	38.2	0.013	0.013	0.013	0.013	0.013	0.013	0.013	0.013	0.013	0.013	0.013	0.013	0.013	0.013	0.013	2.653	0.770	1.285	1.018	0.509	#DIV/0!	#DIV/0!	#DIV/0!	#DIV/0!	0.009	0.061	#DIV/0!	#DIV/0!	0.009	0.050	0.009	0.143
P19132	Ferritin heavy chain OS=Rattus norvegicus GN=Fth1 PE=1 SV=3 - [FRIH_RAT]	21.1	0.752	0.615	0.507	0.815	0.603	0.857	0.523	0.652	0.521	0.535	0.829	0.731	1.583	2.226	0.726	2.979	0.702	1.009	0.984	0.898	0.756	1.122	0.085	0.116	0.036	0.090	0.056	0.077	0.022	0.051	0.756	1.412
P19939	Apolipoprotein C-I OS=Rattus norvegicus GN=Apoc1 PE=3 SV=1 - [APOC1_RAT]	9.9	0.013	0.013	0.013	0.013	0.013	0.013	0.013	0.013	0.013	0.013	0.013	0.013	0.013	0.013	0.013	4.088	0.783	1.414	1.589	0.731	#DIV/0!	#DIV/0!	#DIV/0!	#DIV/0!	0.009	0.034	#DIV/0!	#DIV/0!	0.009	0.026	0.009	0.079
P22057	Prostaglandin-H2 D-isomerase OS=Rattus norvegicus GN=Ptgds PE=1 SV=2 - [PTGDS_RAT]	21.3	0.013	0.013	0.013	0.013	0.013	0.013	0.013	0.013	0.013	0.013	0.013	0.013	0.013	0.013	0.013	3.443	0.910	1.212	2.318	0.847	#DIV/0!	#DIV/0!	#DIV/0!	#DIV/0!	0.009	0.033	#DIV/0!	#DIV/0!	0.009	0.025	0.009	0.076
P22062	Protein-L-isoaspartate(D-aspartate) O-methyltransferase OS=Rattus norvegicus GN=Pcmt1 PE=1 SV=2 - [PIMT_RAT]	24.6	0.247	1.006	0.672	0.873	0.800	0.013	0.013	0.013	0.013	0.013	1.066	0.865	2.054	1.807	0.876	1.174	0.965	0.944	0.985	0.921	0.009	0.415	0.056	0.084	0.085	0.134	0.009	0.054	0.009	0.096	0.756	1.486
P23562	Band 3 anion transport protein OS=Rattus norvegicus OX=10116 GN=Slc4a1 PE=1 SV=3 - [B3AT_RAT]	103.1	0.013	0.013	0.013	0.013	0.013	0.013	0.013	0.013	0.013	0.013	0.013	0.013	0.013	0.013	0.013	3.273	0.748	1.327	1.024	1.180	#DIV/0!	#DIV/0!	#DIV/0!	#DIV/0!	0.009	0.042	#DIV/0!	#DIV/0!	0.009	0.032	0.009	0.091
P23764	Glutathione peroxidase 3 OS=Rattus norvegicus GN=Gpx3 PE=2 SV=2 - [GPX3_RAT]	25.4	0.319	0.468	0.291	0.712	0.614	0.354	0.119	0.340	0.544	0.414	0.632	0.608	1.200	4.587	0.646	4.905	0.403	1.034	0.954	0.626	0.470	1.142	0.085	0.115	0.085	0.130	0.014	0.033	0.036	0.077	0.917	1.027
P27274	CD59 glycoprotein OS=Rattus norvegicus GN=Cd59 PE=1 SV=2 - [CD59_RAT]	13.8	0.013	0.013	0.013	0.013	0.013	0.013	0.013	0.013	0.013	0.013	0.910	0.503	1.582	1.731	0.361	2.625	0.619	1.200	1.186	0.328	#DIV/0!	#DIV/0!	0.009	0.124	0.009	0.067	0.009	0.111	0.009	0.055	0.917	1.287
P27605	Hypoxanthine-guanine phosphoribosyltransferase OS=Rattus norvegicus GN=Hprt1 PE=1 SV=1 - [HPRT_RAT]	24.5	0.651	1.008	0.511	1.131	1.066	1.109	0.474	0.684	0.924	1.049	1.035	1.070	2.119	1.404	1.185	1.341	0.948	1.270	1.321	1.232	0.756	0.968	0.056	0.081	0.056	0.113	0.056	0.073	0.036	0.073	0.917	1.193
P28648	CD63 antigen OS=Rattus norvegicus GN=Cd63 PE=1 SV=2 - [CD63_RAT]	25.7	0.013	0.013	0.013	0.013	0.013	0.013	0.013	0.013	0.013	0.013	0.013	0.013	0.013	0.013	0.013	1.638	0.785	1.337	1.176	0.521	#DIV/0!	#DIV/0!	#DIV/0!	#DIV/0!	0.009	0.081	#DIV/0!	#DIV/0!	0.009	0.066	0.009	0.223
P29147	D-beta-hydroxybutyrate dehydrogenase, mitochondrial OS=Rattus norvegicus OX=10116 GN=Bdh1 PE=1 SV=2 - [BDH_RAT]	38.2	0.013	0.013	0.013	0.013	0.013	0.013	0.013	0.013	0.013	0.013	0.013	0.013	0.013	0.013	0.013	3.104	0.987	1.537	2.010	0.888	#DIV/0!	#DIV/0!	#DIV/0!	#DIV/0!	0.009	0.034	#DIV/0!	#DIV/0!	0.009	0.026	0.009	0.079
P29975	Aquaporin-1 OS=Rattus norvegicus OX=10116 GN=Aqp1 PE=1 SV=4 - [AQP1_RAT]	28.8	0.013	0.013	0.013	0.013	0.013	0.013	0.013	0.013	0.013	0.013	0.622	0.570	1.048	1.236	0.773	2.660	0.867	0.796	0.799	0.413	#DIV/0!	#DIV/0!	0.009	0.373	0.009	0.078	0.009	0.294	0.009	0.065	0.756	1.595
P30919	N(4)-(Beta-N-acetylglucosaminyl)-L-asparaginase OS=Rattus norvegicus OX=10116 GN=Aga PE=1 SV=2 - [ASPG_RAT]	37.1	0.645	0.878	0.450	0.983	0.909	0.945	0.489	0.550	0.895	0.980	0.901	0.884	1.937	1.279	0.961	0.974	1.007	0.998	1.087	0.994	0.917	1.031	0.127	0.155	0.022	0.063	0.184	0.163	0.022	0.052	0.605	1.703
P31000	Vimentin OS=Rattus norvegicus GN=Vim PE=1 SV=2 - [VIME_RAT]	53.7	0.894	0.804	0.529	1.381	1.026	0.013	0.013	0.013	0.013	0.013	0.013	0.013	0.013	0.013	0.013	1.988	0.723	1.434	1.739	0.952	0.009	0.073	0.009	0.194	0.184	0.210	#DIV/0!	#DIV/0!	0.009	0.042	0.009	0.110
P31232	Transgelin OS=Rattus norvegicus GN=Tagln PE=1 SV=2 - [TAGL_RAT]	22.6	0.805	0.919	0.466	0.861	0.905	0.733	0.591	0.994	0.444	0.814	0.897	0.658	2.396	1.114	1.346	1.204	0.883	1.095	1.144	1.504	0.470	1.094	0.184	0.207	0.036	0.091	0.085	0.096	0.022	0.052	0.917	1.229
P35280	Ras-related protein Rab-8A OS=Rattus norvegicus GN=Rab8a PE=1 SV=2 - [RAB8A_RAT]	23.7	0.013	0.013	0.013	0.013	0.013	0.013	0.013	0.013	0.013	0.013	0.013	0.013	0.013	0.013	0.013	2.064	0.823	1.568	1.246	1.252	#DIV/0!	#DIV/0!	#DIV/0!	#DIV/0!	0.009	0.050	#DIV/0!	#DIV/0!	0.009	0.040	0.009	0.108
P37996	ADP-ribosylation factor-like protein 3 OS=Rattus norvegicus GN=Arl3 PE=1 SV=2 - [ARL3_RAT]	20.4	0.515	1.175	0.275	1.279	0.927	0.013	0.013	0.013	0.013	0.013	1.161	1.230	2.323	2.638	0.950	2.885	0.813	1.395	1.607	0.768	0.009	0.140	0.127	0.146	0.184	0.209	0.009	0.034	0.009	0.033	0.756	1.200
P41565	Isocitrate dehydrogenase [NAD] subunit gamma 1, mitochondrial OS=Rattus norvegicus GN=Idh3g PE=2 SV=2 - [IDHG1_RAT]	42.8	0.940	0.738	0.685	1.353	0.948	0.013	0.013	0.013	0.013	0.013	0.679	0.789	1.097	4.259	0.800	5.365	0.479	1.473	1.522	0.514	0.009	0.073	0.917	0.748	0.605	0.532	0.009	0.042	0.009	0.024	0.917	0.978
P47727	Carbonyl reductase [NADPH] 1 OS=Rattus norvegicus GN=Cbr1 PE=1 SV=2 - [CBR1_RAT]	30.6	0.871	0.742	0.553	0.912	0.968	1.000	0.554	0.844	0.710	0.826	0.797	0.863	1.797	1.356	1.074	1.407	0.875	1.243	1.302	1.312	0.756	1.055	0.184	0.209	0.036	0.088	0.085	0.097	0.022	0.051	0.605	1.624
P49911	Acidic leucine-rich nuclear phosphoprotein 32 family member A OS=Rattus norvegicus GN=Anp32a PE=2 SV=1 - [AN32A_RAT]	28.5	1.005	0.893	0.612	1.262	1.078	0.999	0.486	0.754	0.719	0.708	1.102	0.853	2.220	1.248	0.913	2.016	0.766	1.741	1.386	1.053	0.127	0.605	0.470	0.431	0.184	0.208	0.036	0.057	0.022	0.051	0.756	1.352
P50503	Hsc70-interacting protein OS=Rattus norvegicus GN=St13 PE=1 SV=1 - [F10A1_RAT]	41.3	0.785	0.788	0.557	1.025	0.833	0.962	0.534	0.550	0.643	0.679	1.033	0.786	1.905	1.134	0.773	1.047	0.692	1.079	0.995	0.887	0.184	0.776	0.259	0.273	0.184	0.226	0.036	0.057	0.036	0.079	0.605	1.740
P51583	Multifunctional protein ADE2 OS=Rattus norvegicus GN=Paics PE=2 SV=3 - [PUR6_RAT]	47.1	0.702	0.887	0.626	0.994	0.915	1.081	0.584	0.720	0.861	0.877	0.652	0.850	1.671	2.088	0.961	2.084	0.886	1.162	1.126	0.961	0.756	1.015	0.354	0.349	0.085	0.130	0.354	0.278	0.036	0.073	0.756	1.425
P51607	N-acylglucosamine 2-epimerase OS=Rattus norvegicus GN=Renbp PE=2 SV=2 - [RENBP_RAT]	49.6	0.013	0.013	0.013	0.013	0.013	0.013	0.013	0.013	0.013	0.013	0.013	0.013	0.013	0.013	0.013	1.605	0.967	1.263	1.262	1.244	#DIV/0!	#DIV/0!	#DIV/0!	#DIV/0!	0.009	0.059	#DIV/0!	#DIV/0!	0.009	0.048	0.009	0.133
P52632	Signal transducer and activator of transcription 5B OS=Rattus norvegicus GN=Stat5b PE=1 SV=1 - [STA5B_RAT]	90.2	0.762	1.133	0.764	1.201	1.273	0.013	0.013	0.013	0.013	0.013	1.128	1.069	2.029	1.448	1.198	1.251	1.024	1.572	1.033	0.790	0.009	0.059	0.354	0.339	0.605	0.541	0.009	0.052	0.009	0.062	0.259	1.336
P53534	Glycogen phosphorylase, brain form (Fragment) OS=Rattus norvegicus GN=Pygb PE=1 SV=3 - [PYGB_RAT]	96.1	0.844	0.802	0.570	1.036	0.831	1.235	0.493	0.552	0.627	0.558	0.981	1.078	1.778	1.918	0.810	1.901	0.761	1.616	1.426	0.795	0.184	0.773	0.085	0.114	0.354	0.355	0.056	0.076	0.036	0.076	0.605	1.512
P55314	Complement component C8 beta chain OS=Rattus norvegicus GN=C8b PE=2 SV=2 - [CO8B_RAT]	66.6	0.013	0.013	0.013	0.013	0.013	0.013	0.013	0.013	0.013	0.013	0.013	0.013	0.013	0.013	0.013	4.342	0.194	2.109	1.598	1.984	#DIV/0!	#DIV/0!	#DIV/0!	#DIV/0!	0.009	0.030	#DIV/0!	#DIV/0!	0.009	0.023	0.009	0.068
P62161	Calmodulin OS=Rattus norvegicus GN=Calm1 PE=1 SV=2 - [CALM_RAT]	16.8	0.358	1.272	0.278	1.216	1.363	0.013	0.013	0.013	0.013	0.013	1.148	1.036	2.934	1.523	0.990	1.287	0.310	1.289	1.190	1.217	0.009	0.092	0.470	0.425	0.756	0.661	0.009	0.041	0.009	0.074	0.917	1.194
P62260	14-3-3 protein epsilon OS=Rattus norvegicus GN=Ywhae PE=1 SV=1 - [1433E_RAT]	29.2	0.762	0.982	0.497	1.085	1.063	1.041	0.514	0.647	0.918	0.995	0.942	0.950	1.910	1.468	1.059	1.417	0.931	1.257	1.267	1.283	0.605	1.003	0.354	0.347	0.056	0.112	0.085	0.094	0.036	0.074	0.917	1.222
P62278	40S ribosomal protein S13 OS=Rattus norvegicus GN=Rps13 PE=1 SV=2 - [RS13_RAT]	17.2	0.713	0.878	0.857	1.228	1.031	1.019	0.332	0.559	0.748	0.598	1.178	1.468	3.071	0.753	1.031	1.271	0.893	2.057	1.285	0.869	0.085	0.434	0.184	0.199	0.127	0.165	0.022	0.040	0.036	0.078	0.917	1.118
P62836	Ras-related protein Rap-1A OS=Rattus norvegicus GN=Rap1a PE=1 SV=1 - [RAP1A_RAT]	21.0	0.013	0.013	0.013	0.013	0.013	0.013	0.013	0.013	0.013	0.013	0.013	0.013	0.013	0.013	0.013	3.329	0.509	1.626	1.507	0.887	#DIV/0!	#DIV/0!	#DIV/0!	#DIV/0!	0.009	0.039	#DIV/0!	#DIV/0!	0.009	0.030	0.009	0.087
P62870	Transcription elongation factor B polypeptide 2 OS=Rattus norvegicus GN=Tceb2 PE=1 SV=1 - [ELOB_RAT]	13.2	0.868	0.845	0.641	1.136	0.903	0.013	0.013	0.013	0.013	0.013	1.104	0.856	2.087	1.163	0.951	1.241	0.998	1.520	1.291	0.903	0.009	0.105	0.127	0.153	0.056	0.117	0.009	0.063	0.009	0.055	0.605	1.615
P62961	Nuclease-sensitive element-binding protein 1 OS=Rattus norvegicus OX=10116 GN=Ybx1 PE=2 SV=3 - [YBOX1_RAT]	35.7	0.869	0.844	0.666	1.264	1.106	0.721	0.419	0.510	0.520	0.649	1.272	1.049	2.204	1.023	0.965	1.107	0.770	1.194	1.094	0.904	0.022	0.123	0.184	0.202	0.605	0.548	0.014	0.033	0.014	0.034	0.470	1.716
P62994	Growth factor receptor-bound protein 2 OS=Rattus norvegicus GN=Grb2 PE=1 SV=1 - [GRB2_RAT]	25.2	0.013	0.013	0.013	0.013	0.013	0.013	0.013	0.013	0.013	0.013	0.013	0.013	0.013	0.013	0.013	1.257	1.049	1.014	1.155	0.724	#DIV/0!	#DIV/0!	#DIV/0!	#DIV/0!	0.009	0.092	#DIV/0!	#DIV/0!	0.009	0.077	0.009	0.269
P63045	Vesicle-associated membrane protein 2 OS=Rattus norvegicus GN=Vamp2 PE=1 SV=2 - [VAMP2_RAT]	12.7	1.012	0.986	0.560	1.052	0.909	0.013	0.013	0.013	0.013	0.013	0.013	0.013	0.013	0.013	0.013	1.038	0.887	1.770	1.969	0.969	0.009	0.088	0.009	0.238	0.354	0.348	#DIV/0!	#DIV/0!	0.009	0.044	0.009	0.120
P67779	Prohibitin OS=Rattus norvegicus OX=10116 GN=Phb PE=1 SV=1 - [PHB_RAT]	29.8	0.812	1.080	0.446	1.121	1.113	0.013	0.013	0.013	0.013	0.013	0.013	0.013	0.013	0.013	0.013	1.001	1.127	0.977	1.101	1.335	0.009	0.080	0.009	0.213	0.354	0.361	#DIV/0!	#DIV/0!	0.009	0.064	0.009	0.202
P69682	Adaptin ear-binding coat-associated protein 1 OS=Rattus norvegicus GN=Necap1 PE=1 SV=1 - [NECP1_RAT]	29.8	0.954	0.880	0.705	1.129	1.095	0.013	0.013	0.013	0.013	0.013	0.013	0.013	0.013	0.013	0.013	1.146	1.186	0.959	1.010	0.859	0.009	0.069	0.009	0.180	0.354	0.362	#DIV/0!	#DIV/0!	0.009	0.079	0.009	0.281
P82450	Sialate O-acetylesterase OS=Rattus norvegicus OX=10116 GN=Siae PE=1 SV=2 - [SIAE_RAT]	60.4	0.013	0.013	0.013	0.013	0.013	0.013	0.013	0.013	0.013	0.013	0.582	0.700	1.283	1.541	0.873	2.106	0.867	1.590	2.131	1.065	#DIV/0!	#DIV/0!	0.009	0.151	0.009	0.040	0.009	0.120	0.009	0.030	0.127	0.773
P83006	Platelet-activating factor acetylhydrolase 2, cytoplasmic OS=Rattus norvegicus GN=Pafah2 PE=1 SV=2 - [PAFA2_RAT]	43.5	0.013	0.013	0.013	0.013	0.013	0.013	0.013	0.013	0.013	0.013	0.013	0.013	0.013	0.013	0.013	1.824	0.995	1.225	1.019	0.792	#DIV/0!	#DIV/0!	#DIV/0!	#DIV/0!	0.009	0.069	#DIV/0!	#DIV/0!	0.009	0.057	0.009	0.161
P83941	Transcription elongation factor B polypeptide 1 OS=Rattus norvegicus GN=Tceb1 PE=1 SV=1 - [ELOC_RAT]	12.5	0.013	0.013	0.013	0.013	0.013	0.013	0.013	0.013	0.013	0.013	0.013	0.013	0.013	0.013	0.013	1.097	0.878	1.272	1.065	0.760	#DIV/0!	#DIV/0!	#DIV/0!	#DIV/0!	0.009	0.105	#DIV/0!	#DIV/0!	0.009	0.088	0.009	0.315
P85834	Elongation factor Tu, mitochondrial OS=Rattus norvegicus GN=Tufm PE=1 SV=1 - [EFTU_RAT]	49.5	1.577	0.414	1.192	0.728	0.589	0.013	0.013	0.013	0.013	0.013	0.480	0.598	0.901	3.496	0.469	3.106	0.591	1.254	1.031	0.402	0.009	0.090	0.917	0.763	0.756	0.651	0.009	0.066	0.009	0.048	0.756	1.430
P85973	Purine nucleoside phosphorylase OS=Rattus norvegicus GN=Pnp PE=1 SV=1 - [PNPH_RAT]	32.3	0.602	0.905	0.538	0.941	0.948	0.958	0.471	0.679	0.881	1.067	0.804	0.855	1.684	1.708	1.098	1.753	0.908	1.151	1.080	1.092	0.756	1.049	0.184	0.208	0.036	0.089	0.184	0.162	0.036	0.075	0.756	1.455
P97531	Cdc42-interacting protein 4 OS=Rattus norvegicus GN=Trip10 PE=1 SV=3 - [CIP4_RAT]	62.8	0.013	0.013	0.013	0.013	0.013	0.013	0.013	0.013	0.013	0.013	0.013	0.013	0.013	0.013	0.013	1.242	0.923	1.120	1.046	0.722	#DIV/0!	#DIV/0!	#DIV/0!	#DIV/0!	0.009	0.108	#DIV/0!	#DIV/0!	0.009	0.089	0.009	0.331
P97562	Peroxisomal acyl-coenzyme A oxidase 2 OS=Rattus norvegicus OX=10116 GN=Acox2 PE=1 SV=1 - [ACOX2_RAT]	76.7	0.013	0.013	0.013	0.013	0.013	0.013	0.013	0.013	0.013	0.013	0.013	0.013	0.013	0.013	0.013	9.919	0.724	2.362	2.187	2.287	#DIV/0!	#DIV/0!	#DIV/0!	#DIV/0!	0.009	0.028	#DIV/0!	#DIV/0!	0.009	0.022	0.009	0.060
P97571	Calpain-1 catalytic subunit OS=Rattus norvegicus GN=Capn1 PE=1 SV=1 - [CAN1_RAT]	82.1	0.863	0.754	0.659	0.949	0.965	1.140	0.515	0.660	0.831	0.870	0.856	0.878	1.878	2.423	1.023	2.082	0.948	1.205	1.173	1.012	0.756	1.022	0.127	0.150	0.036	0.086	0.085	0.091	0.036	0.073	0.756	1.344
P97608	5-oxoprolinase OS=Rattus norvegicus GN=Oplah PE=1 SV=2 - [OPLA_RAT]	137.6	0.950	0.543	0.632	0.957	1.092	0.013	0.013	0.013	0.013	0.013	0.599	0.732	1.340	1.772	0.835	2.068	0.878	1.187	1.071	0.874	0.009	0.138	0.605	0.543	0.259	0.283	0.009	0.092	0.009	0.053	0.354	1.645
Q01984	Histamine N-methyltransferase OS=Rattus norvegicus OX=10116 GN=Hnmt PE=1 SV=3 - [HNMT_RAT]	33.9	0.013	0.013	0.013	0.013	0.013	0.013	0.013	0.013	0.013	0.013	0.013	0.013	0.013	0.013	0.013	3.002	0.541	0.580	0.461	0.375	#DIV/0!	#DIV/0!	#DIV/0!	#DIV/0!	0.009	0.119	#DIV/0!	#DIV/0!	0.009	0.097	0.009	0.340
Q06645	ATP synthase F(0) complex subunit C1, mitochondrial OS=Rattus norvegicus OX=10116 GN=Atp5mc1 PE=1 SV=1 - [AT5G1_RAT]	14.2	1.785	0.571	1.050	0.910	0.789	1.767	1.061	0.867	0.696	0.555	0.841	0.708	0.982	3.413	0.598	0.013	0.013	0.013	0.013	0.013	0.756	0.902	0.917	0.748	0.009	0.102	0.917	0.655	0.009	0.098	0.009	0.125
Q07009	Calpain-2 catalytic subunit OS=Rattus norvegicus GN=Capn2 PE=1 SV=3 - [CAN2_RAT]	79.9	1.051	0.756	0.764	1.107	1.043	0.013	0.013	0.013	0.013	0.013	1.216	1.289	2.608	2.778	0.957	2.853	0.487	1.778	1.510	1.254	0.009	0.070	0.056	0.077	0.127	0.158	0.009	0.030	0.009	0.029	0.917	0.985
Q0D2L3	Agmatinase, mitochondrial OS=Rattus norvegicus OX=10116 GN=Agmat PE=2 SV=1 - [SPEB_RAT]	38.0	0.013	0.013	0.013	0.013	0.013	0.013	0.013	0.013	0.013	0.013	0.516	0.480	0.904	0.834	0.599	1.600	1.084	0.927	0.841	0.931	#DIV/0!	#DIV/0!	0.009	1.742	0.009	0.084	0.009	1.517	0.009	0.070	0.022	0.147
Q10758	Keratin, type II cytoskeletal 8 OS=Rattus norvegicus GN=Krt8 PE=1 SV=3 - [K2C8_RAT]	54.0	1.517	0.710	0.704	1.353	0.956	0.948	0.567	0.639	0.672	0.626	0.965	0.746	2.026	0.896	0.608	1.013	0.826	1.091	1.139	0.759	0.036	0.193	0.917	0.762	0.917	0.772	0.184	0.165	0.036	0.079	0.470	1.802
Q1HCL7	NAD kinase 2, mitochondrial OS=Rattus norvegicus OX=10116 GN=Nadk2 PE=1 SV=1 - [NAKD2_RAT]	48.1	0.644	1.110	0.534	1.069	1.055	1.132	0.892	0.818	0.885	0.849	1.025	0.850	2.058	1.015	1.122	1.816	1.014	1.655	2.025	0.984	0.917	0.943	0.470	0.437	0.184	0.208	0.184	0.160	0.036	0.069	0.756	1.336
Q2PQA9	Kinesin-1 heavy chain OS=Rattus norvegicus GN=Kif5b PE=1 SV=1 - [KINH_RAT]	109.5	1.043	1.004	0.724	1.240	0.936	0.980	0.543	0.508	0.700	0.659	1.169	1.153	2.000	2.178	0.994	3.046	0.836	1.806	2.084	1.250	0.036	0.194	0.127	0.146	0.085	0.117	0.014	0.031	0.022	0.050	0.605	1.341
Q3T1J1	Eukaryotic translation initiation factor 5A-1 OS=Rattus norvegicus GN=Eif5a PE=1 SV=3 - [IF5A1_RAT]	16.8	0.987	0.977	0.586	1.316	1.275	0.873	0.478	0.695	0.886	1.068	1.179	1.188	2.652	0.933	1.285	1.019	0.986	1.502	1.531	1.344	0.184	0.716	0.470	0.424	0.127	0.162	0.022	0.039	0.036	0.073	0.756	1.334
Q4G050	MAP kinase-interacting serine/threonine-protein kinase 1 OS=Rattus norvegicus OX=10116 GN=Mknk1 PE=2 SV=1 - [MKNK1_RAT]	46.2	0.013	0.013	0.013	0.013	0.013	0.013	0.013	0.013	0.013	0.013	0.013	0.013	0.013	0.013	0.013	0.802	0.552	1.690	1.349	0.925	#DIV/0!	#DIV/0!	#DIV/0!	#DIV/0!	0.009	0.086	#DIV/0!	#DIV/0!	0.009	0.072	0.009	0.244
Q4V8K2	Beta-catenin-like protein 1 OS=Rattus norvegicus GN=Ctnnbl1 PE=1 SV=1 - [CTBL1_RAT]	64.9	1.753	1.385	1.180	1.351	1.293	1.403	0.935	0.901	0.744	1.051	1.165	1.321	1.449	2.190	1.210	1.955	1.160	1.599	1.420	1.667	0.085	0.421	0.917	0.746	0.354	0.341	0.056	0.065	0.022	0.050	0.605	1.396
Q561S0	NADH dehydrogenase [ubiquinone] 1 alpha subcomplex subunit 10, mitochondrial OS=Rattus norvegicus GN=Ndufa10 PE=1 SV=1 - [NDUAA_RAT]	40.5	0.493	1.334	0.379	1.730	1.290	0.013	0.013	0.013	0.013	0.013	1.373	1.940	3.034	4.150	1.004	2.160	0.853	1.636	1.726	0.624	0.009	0.057	0.085	0.105	0.354	0.342	0.009	0.023	0.009	0.039	0.259	1.256
Q562C6	Leucine zipper transcription factor-like protein 1 OS=Rattus norvegicus OX=10116 GN=Lztfl1 PE=2 SV=1 - [LZTL1_RAT]	34.6	0.460	1.183	0.404	1.047	0.950	0.013	0.013	0.013	0.013	0.013	0.989	1.061	1.756	2.515	1.087	2.375	0.883	1.655	1.363	1.267	0.009	0.183	0.085	0.111	0.056	0.093	0.009	0.044	0.009	0.033	0.917	1.055

Q5BJY9	Keratin, type I cytoskeletal 18 OS=Rattus norvegicus GN=Krt18 PE=1 SV=3 - [K1C18_RAT]	47.7	1.401	0.731	0.750	1.460	0.972	0.968	0.495	0.702	0.716	0.696	1.117	0.747	1.923	1.661	0.649	1.383	0.752	1.184	1.413	0.781	0.036	0.192	0.756	0.646	0.756	0.652	0.127	0.126	0.036	0.078	0.917	1.259
Q5EGY4	Synaptobrevin homolog YKT6 OS=Rattus norvegicus GN=Ykt6 PE=1 SV=1 - [YKT6_RAT]	22.4	0.480	1.084	0.395	1.342	1.225	0.013	0.013	0.013	0.013	0.013	1.263	1.196	2.582	2.280	0.982	2.241	0.590	1.497	1.476	1.259	0.009	0.086	0.184	0.196	0.085	0.121	0.009	0.034	0.009	0.038	0.756	1.215
Q5I0J9	Putative L-aspartate dehydrogenase OS=Rattus norvegicus OX=10116 GN=Aspdh PE=2 SV=1 - [ASPD_RAT]	31.2	0.013	0.013	0.013	0.013	0.013	0.013	0.013	0.013	0.013	0.013	0.425	0.495	0.933	2.585	0.731	2.221	0.888	0.986	0.937	0.771	#DIV/0!	#DIV/0!	0.009	0.112	0.009	0.071	0.009	0.10002	0.009	0.060	0.259	1.385
Q5M7W5	Microtubule-associated protein 4 OS=Rattus norvegicus GN=Map4 PE=1 SV=1 - [MAP4_RAT]	110.2	0.013	0.013	0.013	0.013	0.013	1.394	1.142	1.036	0.753	0.711	0.013	0.013	0.013	0.013	0.013	0.013	0.013	0.013	0.013	0.013	0.009	0.062	#DIV/0!	#DIV/0!	#DIV/0!	#DIV/0!	0.009	0.115	0.009	0.090	#DIV/0!	#DIV/0!
Q5M819	Phosphoserine phosphatase OS=Rattus norvegicus GN=Psph PE=2 SV=1 - [SERB_RAT]	25.0	0.788	0.839	0.675	0.981	0.974	1.019	0.493	0.709	0.872	0.973	0.962	0.955	1.971	1.764	1.041	1.600	0.873	1.223	1.243	1.084	0.917	0.989	0.085	0.113	0.036	0.087	0.085	0.093	0.036	0.075	0.917	1.205
Q5PQL8	Protein FAM149B1 OS=Rattus norvegicus OX=10116 GN=Fam149b1 PE=2 SV=1 - [F149B_RAT]	53.2	1.449	0.547	1.164	0.869	0.861	1.466	0.871	2.054	0.238	0.494	0.013	0.013	0.013	0.013	0.013	0.013	0.013	0.013	0.013	0.013	0.917	0.919	0.009	0.161	0.009	0.125	0.009	0.105	0.009	0.082	#DIV/0!	#DIV/0!
Q5U1Y4	1,5-anhydro-D-fructose reductase OS=Rattus norvegicus GN=Akr1e2 PE=2 SV=1 - [AKCL2_RAT]	34.5	0.013	0.013	0.013	0.013	0.013	0.013	0.013	0.013	0.013	0.013	0.472	0.540	1.057	2.277	0.798	3.435	0.530	1.155	0.901	0.965	#DIV/0!	#DIV/0!	0.009	0.116	0.009	0.049	0.009	0.102	0.009	0.039	0.470	1.673
Q5U2U7	mRNA cap guanine-N7 methyltransferase OS=Rattus norvegicus GN=Rnmt PE=1 SV=1 - [MCES_RAT]	52.8	0.676	0.819	0.625	0.745	0.732	1.094	0.652	0.841	0.805	0.910	0.990	0.824	1.972	0.456	1.161	1.276	0.917	1.230	1.203	0.981	0.127	0.615	0.127	0.157	0.014	0.043	0.470	0.359	0.036	0.076	0.470	1.746
Q5U2V4	Phospholipase B-like 1 OS=Rattus norvegicus OX=10116 GN=Plbd1 PE=2 SV=1 - [PLBL1_RAT]	63.0	0.013	0.013	0.013	0.013	0.013	0.013	0.013	0.013	0.013	0.013	0.641	0.682	1.408	1.419	0.878	1.076	1.193	1.379	1.186	0.902	#DIV/0!	#DIV/0!	0.009	0.138	0.009	0.073	0.009	0.118	0.009	0.060	0.605	1.723
Q5U312	Ankycorbin OS=Rattus norvegicus OX=10116 GN=Rai14 PE=1 SV=2 - [RAI14_RAT]	109.1	0.013	0.013	0.013	0.013	0.013	1.399	2.571	1.106	0.361	0.409	0.013	0.013	0.013	0.013	0.013	0.013	0.013	0.013	0.013	0.013	0.009	0.053	#DIV/0!	#DIV/0!	#DIV/0!	#DIV/0!	0.009	0.070	0.009	0.058	#DIV/0!	#DIV/0!
Q5XID1	Anamorsin OS=Rattus norvegicus GN=Ciapin1 PE=2 SV=1 - [CPIN1_RAT]	33.0	0.723	1.243	0.401	1.305	1.049	0.976	0.413	0.547	1.145	1.119	1.033	0.948	1.949	1.377	1.023	1.555	0.980	1.317	1.179	1.158	0.605	0.960	0.470	0.433	0.259	0.277	0.259	0.211	0.036	0.073	0.756	1.421
Q5XIG6	N-acetylgalactosamine kinase OS=Rattus norvegicus OX=10116 GN=Galk2 PE=2 SV=1 - [GALK2_RAT]	50.2	0.013	0.013	0.013	0.013	0.013	0.013	0.013	0.013	0.013	0.013	0.013	0.013	0.013	0.013	0.013	2.856	0.663	1.431	1.250	0.782	#DIV/0!	#DIV/0!	#DIV/0!	#DIV/0!	0.009	0.050	#DIV/0!	#DIV/0!	0.009	0.040	0.009	0.106
Q5XIG8	Serine-threonine kinase receptor-associated protein OS=Rattus norvegicus GN=Strap PE=1 SV=1 - [STRAP_RAT]	38.4	0.740	0.773	0.796	0.932	0.913	0.904	0.352	0.583	0.770	0.709	0.718	0.914	1.593	1.204	1.111	1.483	0.868	1.310	1.098	0.989	0.085	0.437	0.184	0.209	0.036	0.089	0.036	0.057	0.022	0.052	0.917	1.281
Q5XIM5	Protein CDV3 homolog OS=Rattus norvegicus OX=10116 GN=Cdv3 PE=1 SV=1 - [CDV3_RAT]	24.3	0.013	0.013	0.013	0.013	0.013	0.013	0.013	0.013	0.013	0.013	0.013	0.013	0.013	0.013	0.013	1.714	0.754	1.459	1.618	1.298	#DIV/0!	#DIV/0!	#DIV/0!	#DIV/0!	0.009	0.051	#DIV/0!	#DIV/0!	0.009	0.041	0.009	0.109
Q62696	Disks large homolog 1 OS=Rattus norvegicus GN=Dlg1 PE=1 SV=1 - [DLG1_RAT]	100.5	0.751	0.705	0.699	0.957	0.904	0.013	0.013	0.013	0.013	0.013	0.609	0.771	1.572	1.348	0.757	1.579	0.972	1.252	1.061	0.966	0.009	0.196	0.470	0.445	0.014	0.041	0.009	0.114	0.009	0.058	0.354	1.667
Q62745	CD81 antigen OS=Rattus norvegicus GN=Cd81 PE=1 SV=1 - [CD81_RAT]	25.9	1.048	0.780	0.763	1.046	0.914	0.013	0.013	0.013	0.013	0.013	0.845	0.693	1.426	2.179	0.728	2.343	0.762	1.573	1.371	0.802	0.009	0.083	0.917	0.763	0.354	0.346	0.009	0.069	0.009	0.041	0.470	1.602
Q62975	Protein Z-dependent protease inhibitor OS=Rattus norvegicus GN=Serpina10 PE=2 SV=2 - [ZPI_RAT]	50.2	0.013	0.013	0.013	0.013	0.013	0.013	0.013	0.013	0.013	0.013	0.013	0.013	0.013	0.013	0.013	1.768	1.057	1.289	1.430	0.720	#DIV/0!	#DIV/0!	#DIV/0!	#DIV/0!	0.009	0.061	#DIV/0!	#DIV/0!	0.009	0.050	0.009	0.142
Q63065	[Pyruvate dehydrogenase (acetyl-transferring)] kinase isozyme 1, mitochondrial OS=Rattus norvegicus OX=10116 GN=Pdk1 PE=1 SV=1 - [PDK1_RAT]	49.0	0.868	1.145	0.670	1.124	1.087	0.013	0.013	0.013	0.013	0.013	0.013	0.013	0.013	0.013	0.013	1.505	0.941	1.546	1.388	1.139	0.009	0.066	0.009	0.158	0.085	0.122	#DIV/0!	#DIV/0!	0.009	0.045	0.009	0.127
Q63108	Carboxylesterase 1E OS=Rattus norvegicus GN=Ces1e PE=2 SV=1 - [EST1E_RAT]	61.7	0.013	0.013	0.013	0.013	0.013	0.013	0.013	0.013	0.013	0.013	0.013	0.013	0.013	0.013	0.013	3.851	0.559	0.996	0.942	0.743	#DIV/0!	#DIV/0!	#DIV/0!	#DIV/0!	0.009	0.047	#DIV/0!	#DIV/0!	0.009	0.037	0.009	0.102
Q63135	Complement component receptor 1-like protein OS=Rattus norvegicus GN=Cr1l PE=1 SV=1 - [CR1L_RAT]	61.6	0.825	0.926	0.584	0.934	0.939	0.013	0.013	0.013	0.013	0.013	0.013	0.013	0.013	0.013	0.013	2.240	0.680	1.389	1.237	0.773	0.009	0.128	0.009	0.435	0.354	0.357	#DIV/0!	#DIV/0!	0.009	0.049	0.009	0.136
Q63150	Dihydropyrimidinase OS=Rattus norvegicus OX=10116 GN=Dpys PE=1 SV=2 - [DPYS_RAT]	56.8	0.443	0.687	0.308	0.501	0.623	0.013	0.013	0.013	0.013	0.013	0.620	0.694	1.026	4.777	0.600	4.032	0.609	1.096	0.947	0.774	0.009	1.540	0.085	0.115	0.036	0.088	0.009	0.040	0.009	0.034	0.605	1.392
Q63377	Sodium/potassium-transporting ATPase subunit beta-3 OS=Rattus norvegicus GN=Atp1b3 PE=2 SV=1 - [AT1B3_RAT]	31.8	1.029	1.055	0.540	1.079	0.813	0.013	0.013	0.013	0.013	0.013	1.315	1.259	1.932	2.107	0.969	1.437	0.743	1.761	1.352	0.613	0.009	0.088	0.056	0.079	0.354	0.358	0.009	0.043	0.009	0.056	0.470	1.548
Q63416	Inter-alpha-trypsin inhibitor heavy chain H3 OS=Rattus norvegicus GN=Itih3 PE=2 SV=1 - [ITIH3_RAT]	99.0	0.691	0.597	0.589	0.811	0.806	0.646	0.480	0.800	0.658	0.743	0.776	0.740	1.539	1.671	0.925	2.335	0.822	1.143	0.912	0.970	0.605	1.114	0.085	0.116	0.014	0.042	0.056	0.077	0.014	0.033	0.605	1.645
Q63450	Calcium/calmodulin-dependent protein kinase type 1 OS=Rattus norvegicus GN=Camk1 PE=1 SV=2 - [KCC1A_RAT]	41.6	0.013	0.013	0.013	0.013	0.013	1.792	0.696	0.900	1.058	0.911	0.013	0.013	0.013	0.013	0.013	0.013	0.013	0.013	0.013	0.013	0.009	0.056	#DIV/0!	#DIV/0!	#DIV/0!	#DIV/0!	0.009	0.088	0.009	0.071	#DIV/0!	#DIV/0!
Q63544	Gamma-synuclein OS=Rattus norvegicus OX=10116 GN=Sncg PE=1 SV=2 - [SYUG_RAT]	13.0	0.783	0.736	0.664	1.056	1.167	1.023	0.625	0.918	0.899	0.878	0.922	0.885	2.676	2.036	1.110	1.142	0.971	1.026	1.015	1.109	0.917	0.956	0.127	0.148	0.354	0.364	0.085	0.088	0.036	0.078	0.756	1.390
Q63556	Serine protease inhibitor A3M (Fragment) OS=Rattus norvegicus GN=Serpina3m PE=2 SV=1 - [SPA3M_RAT]	46.1	0.711	0.737	0.460	0.842	0.854	0.806	0.487	0.607	0.863	0.854	0.678	0.744	1.378	1.280	0.728	1.858	0.814	1.325	1.090	1.112	0.756	1.098	0.470	0.445	0.036	0.090	0.470	0.362	0.036	0.076	0.259	1.380
Q64119	Myosin light polypeptide 6 OS=Rattus norvegicus GN=Myl6 PE=1 SV=3 - [MYL6_RAT]	17.0	0.634	1.173	0.411	1.325	1.133	0.013	0.013	0.013	0.013	0.013	1.872	1.122	2.870	2.466	0.858	2.382	0.606	1.455	2.096	1.169	0.009	0.072	0.184	0.195	0.184	0.205	0.009	0.028	0.009	0.031	0.605	1.322
Q64361	Latexin OS=Rattus norvegicus GN=Lxn PE=1 SV=1 - [LXN_RAT]	25.6	0.798	0.735	0.644	0.975	0.899	0.970	0.704	0.879	0.854	0.878	0.606	0.702	1.503	0.870	1.078	1.020	1.007	0.964	1.152	1.047	0.756	1.008	0.756	0.660	0.022	0.062	0.917	0.662	0.022	0.052	0.470	1.807
Q64537	Calpain small subunit 1 OS=Rattus norvegicus GN=Capns1 PE=1 SV=3 - [CPNS1_RAT]	28.6	0.935	0.626	0.722	1.007	0.940	0.013	0.013	0.013	0.013	0.013	0.878	0.941	1.946	1.509	1.166	1.495	1.042	1.263	1.285	1.178	0.009	0.125	0.085	0.114	0.014	0.039	0.009	0.058	0.009	0.050	0.756	1.406
Q64620	Serine/threonine-protein phosphatase 6 catalytic subunit OS=Rattus norvegicus GN=Ppp6c PE=2 SV=2 - [PPP6_RAT]	35.1	0.013	0.013	0.013	0.013	0.013	0.013	0.013	0.013	0.013	0.013	0.730	0.799	1.389	1.503	0.857	1.376	0.915	1.059	1.055	0.768	#DIV/0!	#DIV/0!	0.009	0.102	0.009	0.094	0.009	0.091	0.009	0.078	0.917	1.310
Q66H15	Regulator of microtubule dynamics protein 3 OS=Rattus norvegicus OX=10116 GN=Rmdn3 PE=1 SV=1 - [RMD3_RAT]	52.3	0.013	0.013	0.013	0.013	0.013	0.013	0.013	0.013	0.013	0.013	0.013	0.013	0.013	0.013	0.013	6.681	0.298	1.768	1.736	1.831	#DIV/0!	#DIV/0!	#DIV/0!	#DIV/0!	0.009	0.029	#DIV/0!	#DIV/0!	0.009	0.022	0.009	0.063
Q66HA6	ADP-ribosylation factor-like protein 8B OS=Rattus norvegicus GN=Arl8b PE=2 SV=1 - [ARL8B_RAT]	21.5	0.884	1.036	0.458	1.452	1.040	0.013	0.013	0.013	0.013	0.013	1.676	1.029	2.429	3.403	0.863	2.344	0.569	1.238	1.249	0.708	0.009	0.066	0.259	0.259	0.605	0.539	0.009	0.027	0.009	0.052	0.259	1.286
Q66HD3	Nuclear autoantigenic sperm protein OS=Rattus norvegicus GN=Nasp PE=1 SV=1 - [NASP_RAT]	84.1	0.013	0.013	0.013	0.013	0.013	0.013	0.013	0.013	0.013	0.013	0.013	0.013	0.013	0.013	0.013	1.480	0.474	1.237	1.778	1.344	#DIV/0!	#DIV/0!	#DIV/0!	#DIV/0!	0.009	0.060	#DIV/0!	#DIV/0!	0.009	0.049	0.009	0.137
Q68FT1	Ubiquinone biosynthesis protein COQ9, mitochondrial OS=Rattus norvegicus OX=10116 GN=Coq9 PE=1 SV=2 - [COQ9_RAT]	35.1	0.635	0.851	0.444	1.077	1.045	1.009	0.481	0.591	0.914	0.938	0.760	0.961	2.085	1.510	1.181	1.349	1.024	1.256	1.407	1.103	0.756	1.052	0.127	0.154	0.036	0.087	0.085	0.094	0.014	0.033	0.917	1.211
Q68FT5	S-methylmethionine--homocysteine S-methyltransferase BHMT2 OS=Rattus norvegicus OX=10116 GN=Bhmt2 PE=2 SV=1 - [BHMT2_RAT]	39.9	0.013	0.013	0.013	0.013	0.013	0.013	0.013	0.013	0.013	0.013	0.013	0.013	0.013	0.013	0.013	1.927	0.781	1.079	1.069	0.804	#DIV/0!	#DIV/0!	#DIV/0!	#DIV/0!	0.009	0.075	#DIV/0!	#DIV/0!	0.009	0.063	0.009	0.179
Q6AY20	Cation-dependent mannose-6-phosphate receptor OS=Rattus norvegicus OX=10116 GN=M6pr PE=1 SV=1 - [MPRD_RAT]	31.1	0.903	0.726	0.788	0.903	0.825	0.013	0.013	0.013	0.013	0.013	0.695	0.702	1.308	1.993	0.723	1.856	0.840	1.025	0.830	0.569	0.009	0.152	0.605	0.543	0.470	0.451	0.009	0.084	0.009	0.082	0.917	1.305
Q6AYC4	Macrophage-capping protein OS=Rattus norvegicus GN=Capg PE=1 SV=1 - [CAPG_RAT]	38.8	0.642	0.790	0.487	0.911	0.758	0.936	0.521	0.798	0.626	0.799	0.785	0.659	2.241	1.019	0.924	1.389	0.947	1.149	0.946	1.136	0.605	1.101	0.127	0.156	0.014	0.043	0.259	0.214	0.014	0.033	0.259	1.375
Q6IFU7	Keratin, type I cytoskeletal 42 OS=Rattus norvegicus GN=Krt42 PE=3 SV=1 - [K1C42_RAT]	50.2	0.013	0.013	0.013	0.013	0.013	0.926	0.744	0.652	1.008	2.273	0.013	0.013	0.013	0.013	0.013	0.013	0.013	0.013	0.013	0.013	0.009	0.053	#DIV/0!	#DIV/0!	#DIV/0!	#DIV/0!	0.009	0.076	0.009	0.064	#DIV/0!	#DIV/0!
Q6IFU8	Keratin, type I cytoskeletal 17 OS=Rattus norvegicus GN=Krt17 PE=2 SV=1 - [K1C17_RAT]	48.1	0.013	0.013	0.013	0.013	0.013	0.813	0.491	0.719	1.075	3.344	0.013	0.013	0.013	0.013	0.013	0.013	0.013	0.013	0.013	0.013	0.009	0.051	#DIV/0!	#DIV/0!	#DIV/0!	#DIV/0!	0.009	0.058	0.009	0.047	#DIV/0!	#DIV/0!
Q6IG04	Keratin, type II cytoskeletal 72 OS=Rattus norvegicus GN=Krt72 PE=3 SV=2 - [K2C72_RAT]	56.8	0.811	1.544	0.268	0.861	1.575	0.574	0.518	0.279	1.002	2.992	0.013	0.013	0.013	0.013	0.013	0.013	0.013	0.013	0.013	0.013	0.756	0.900	0.009	0.131	0.009	0.107	0.009	0.087	0.009	0.070	#DIV/0!	#DIV/0!
Q6IG12	Keratin, type II cytoskeletal 7 OS=Rattus norvegicus GN=Krt7 PE=3 SV=1 - [K2C7_RAT]	50.7	0.013	0.013	0.013	0.013	0.013	0.013	0.013	0.013	0.013	0.013	0.013	0.013	0.013	0.013	0.013	6.687	0.204	0.911	0.854	0.775	#DIV/0!	#DIV/0!	#DIV/0!	#DIV/0!	0.009	0.031	#DIV/0!	#DIV/0!	0.009	0.024	0.009	0.072
Q6IRE4	Tumor susceptibility gene 101 protein OS=Rattus norvegicus GN=Tsg101 PE=1 SV=1 - [TS101_RAT]	44.0	0.796	1.161	0.525	1.053	0.961	0.013	0.013	0.013	0.013	0.013	0.013	0.013	0.013	0.013	0.013	1.734	0.624	1.710	1.790	1.270	0.009	0.091	0.009	0.249	0.085	0.120	#DIV/0!	#DIV/0!	0.009	0.036	0.009	0.1001
Q6IUP3	Elongator complex protein 5 OS=Rattus norvegicus GN=Elp5 PE=2 SV=1 - [ELP5_RAT]	35.5	0.013	0.013	0.013	0.013	0.013	1.787	0.516	1.872	0.404	0.631	0.013	0.013	0.013	0.013	0.013	0.013	0.013	0.013	0.013	0.013	0.009	0.058	#DIV/0!	#DIV/0!	#DIV/0!	#DIV/0!	0.009	0.097	0.009	0.077	#DIV/0!	#DIV/0!
Q6JE36	Protein NDRG1 OS=Rattus norvegicus GN=Ndrg1 PE=1 SV=1 - [NDRG1_RAT]	42.9	0.695	0.763	0.503	1.119	1.138	1.006	0.465	0.762	0.851	0.875	0.867	0.826	1.615	3.440	1.156	2.681	0.977	1.311	1.354	1.135	0.756	1.024	0.085	0.108	0.056	0.092	0.127	0.117	0.022	0.050	0.756	1.217
Q6MG08	ATP-binding cassette sub-family F member 1 OS=Rattus norvegicus OX=10116 GN=Abcf1 PE=1 SV=1 - [ABCF1_RAT]	95.2	0.013	0.013	0.013	0.013	0.013	0.013	0.013	0.013	0.013	0.013	0.013	0.013	0.013	0.013	0.013	2.433	0.202	2.762	1.749	1.082	#DIV/0!	#DIV/0!	#DIV/0!	#DIV/0!	0.009	0.036	#DIV/0!	#DIV/0!	0.009	0.028	0.009	0.082
Q6MGB5	Estradiol 17-beta-dehydrogenase 8 OS=Rattus norvegicus OX=10116 GN=Hsd17b8 PE=1 SV=1 - [DHB8_RAT]	26.8	0.013	0.013	0.013	0.013	0.013	0.013	0.013	0.013	0.013	0.013	0.013	0.013	0.013	0.013	0.013	2.256	0.980	1.018	1.008	0.942	#DIV/0!	#DIV/0!	#DIV/0!	#DIV/0!	0.009	0.063	#DIV/0!	#DIV/0!	0.009	0.051	0.009	0.145
Q6P791	Ragulator complex protein LAMTOR1 OS=Rattus norvegicus GN=Lamtor1 PE=1 SV=1 - [LTOR1_RAT]	17.7	0.013	0.013	0.013	0.013	0.013	0.013	0.013	0.013	0.013	0.013	0.013	0.013	0.013	0.013	0.013	1.838	0.578	1.281	1.234	1.067	#DIV/0!	#DIV/0!	#DIV/0!	#DIV/0!	0.009	0.067	#DIV/0!	#DIV/0!	0.009	0.054	0.009	0.152
Q6TUG0	DnaJ homolog subfamily B member 11 OS=Rattus norvegicus GN=Dnajb11 PE=2 SV=1 - [DJB11_RAT]	40.5	1.320	0.754	1.288	0.925	0.849	0.013	0.013	0.013	0.013	0.013	0.687	0.755	1.651	1.462	0.952	1.403	1.102	1.402	0.800	0.771	0.009	0.059	0.756	0.651	0.605	0.542	0.009	0.081	0.009	0.066	0.917	1.292
Q704E8	ATP-binding cassette sub-family B member 7, mitochondrial OS=Rattus norvegicus OX=10116 GN=Abcb7 PE=1 SV=1 - [ABCB7_RAT]	82.5	0.013	0.013	0.013	0.013	0.013	1.731	0.925	0.863	0.941	0.957	0.013	0.013	0.013	0.013	0.013	0.013	0.013	0.013	0.013	0.013	0.009	0.055	#DIV/0!	#DIV/0!	#DIV/0!	#DIV/0!	0.009	0.085	0.009	0.068	#DIV/0!	#DIV/0!
Q75Q39	Mitochondrial import receptor subunit TOM70 OS=Rattus norvegicus GN=Tomm70a PE=1 SV=1 - [TOM70_RAT]	67.4	0.013	0.013	0.013	0.013	0.013	0.013	0.013	0.013	0.013	0.013	0.013	0.013	0.013	0.013	0.013	1.671	1.259	1.126	0.952	0.811	#DIV/0!	#DIV/0!	#DIV/0!	#DIV/0!	0.009	0.070	#DIV/0!	#DIV/0!	0.009	0.058	0.009	0.163
Q75WE7	von Willebrand factor A domain-containing protein 5A OS=Rattus norvegicus GN=Vwa5a PE=2 SV=1 - [VWA5A_RAT]	91.4	0.013	0.013	0.013	0.013	0.013	0.013	0.013	0.013	0.013	0.013	0.711	0.686	1.294	1.780	0.666	1.933	0.902	0.964	1.064	0.944	#DIV/0!	#DIV/0!	0.009	0.119	0.009	0.071	0.009	0.103	0.009	0.059	0.354	1.661
Q76IC5	Pyroglutamyl-peptidase 1 OS=Rattus norvegicus OX=10116 GN=Pgpep1 PE=1 SV=1 - [PGPI_RAT]	22.9	0.013	0.013	0.013	0.013	0.013	0.013	0.013	0.013	0.013	0.013	0.013	0.013	0.013	0.013	0.013	4.618	0.294	1.073	1.064	1.081	#DIV/0!	#DIV/0!	#DIV/0!	#DIV/0!	0.009	0.037	#DIV/0!	#DIV/0!	0.009	0.028	0.009	0.083
Q78P75	Dynein light chain 2, cytoplasmic OS=Rattus norvegicus GN=Dynll2 PE=1 SV=1 - [DYL2_RAT]	10.3	0.693	1.186	0.424	1.333	0.926	0.013	0.013	0.013	0.013	0.013	1.030	0.815	1.832	1.594	0.827	3.109	0.602	1.421	1.910	0.999	0.009	0.082	0.354	0.347	0.184	0.205	0.009	0.064	0.009	0.029	0.605	1.447
Q7M767	Ubiquitin-conjugating enzyme E2 variant 2 OS=Rattus norvegicus GN=Ube2v2 PE=1 SV=3 - [UB2V2_RAT]	16.3	0.814	0.878	0.541	1.057	1.023	1.163	0.594	0.696	0.900	0.876	0.960	0.917	1.949	1.137	0.985	1.204	0.942	1.206	1.209	1.076	0.917	0.970	0.184	0.207	0.036	0.089	0.085	0.095	0.036	0.076	0.470	1.711
Q7TP48	Adipocyte plasma membrane-associated protein OS=Rattus norvegicus OX=10116 GN=Apmap PE=2 SV=2 - [APMAP_RAT]	42.0	0.393	0.917	0.303	1.028	0.906	0.013	0.013	0.013	0.013	0.013	0.823	0.787	1.387	4.593	0.712	4.071	0.570	1.230	1.138	0.793	0.009	0.567	0.470	0.426	0.184	0.211	0.009	0.034	0.009	0.030	0.917	1.008
Q811M5	Complement component C6 OS=Rattus norvegicus GN=C6 PE=2 SV=1 - [CO6_RAT]	105.0	0.013	0.013	0.013	0.013	0.013	0.013	0.013	0.013	0.013	0.013	0.013	0.013	0.013	0.013	0.013	3.017	0.892	1.475	1.150	1.096	#DIV/0!	#DIV/0!	#DIV/0!	#DIV/0!	0.009	0.041	#DIV/0!	#DIV/0!	0.009	0.032	0.009	0.090
Q8K4V4	Sorting nexin-27 OS=Rattus norvegicus GN=Snx27 PE=1 SV=2 - [SNX27_RAT]	61.0	0.013	0.013	0.013	0.013	0.013	0.013	0.013	0.013	0.013	0.013	0.604	0.703	1.456	1.611	0.741	1.142	1.217	0.930	0.929	0.908	#DIV/0!	#DIV/0!	0.009	0.122	0.009	0.0995	0.009	0.106	0.009	0.081	0.605	1.743
Q8R491	EH domain-containing protein 3 OS=Rattus norvegicus GN=Ehd3 PE=1 SV=2 - [EHD3_RAT]	60.8	0.013	0.013	0.013	0.013	0.013	0.013	0.013	0.013	0.013	0.013	0.013	0.013	0.013	0.013	0.013	4.388	0.404	1.773	2.052	1.292	#DIV/0!	#DIV/0!	#DIV/0!	#DIV/0!	0.009	0.030	#DIV/0!	#DIV/0!	0.009	0.023	0.009	0.069
Q91ZY8	E3 ubiquitin-protein ligase TRIM9 OS=Rattus norvegicus GN=Trim9 PE=1 SV=1 - [TRIM9_RAT]	79.2	0.013	0.013	0.013	0.013	0.013	0.013	0.013	0.013	0.013	0.013	0.013	0.013	0.013	0.013	0.013	1.037	1.038	1.128	1.090	0.852	#DIV/0!	#DIV/0!	#DIV/0!	#DIV/0!	0.009	0.098	#DIV/0!	#DIV/0!	0.009	0.080	0.009	0.293
Q921A4	Cytoglobin OS=Rattus norvegicus OX=10116 GN=Cygb PE=1 SV=1 - [CYGB_RAT]	21.5	0.791	0.844	0.722	1.048	0.977	0.564	0.386	0.554	0.606	0.645	0.959	0.800	1.672	2.969	0.966	3.902	0.638	1.141	1.525	1.180	0.014	0.077	0.259	0.264	0.127	0.158	0.014	0.032	0.022	0.050	0.756	1.201
Q9EQ10	PCNA-interacting partner OS=Rattus norvegicus OX=10116 GN=Parpbp PE=1 SV=2 - [PARI_RAT]	63.9	1.302	0.332	1.087	0.903	0.813	1.960	0.991	1.382	0.314	0.428	0.013	0.013	0.013	0.013	0.013	0.013	0.013	0.013	0.013	0.013	0.756	0.914	0.009	0.282	0.009	0.214	0.009	0.112	0.009	0.087	#DIV/0!	#DIV/0!
Q9EQX9	Ubiquitin-conjugating enzyme E2 N OS=Rattus norvegicus GN=Ube2n PE=1 SV=1 - [UBE2N_RAT]	17.1	0.784	1.054	0.640	1.160	0.952	1.215	0.584	0.693	0.842	0.835	1.022	1.027	2.040	1.274	1.083	1.227	0.955	1.249	1.278	1.027	0.605	0.980	0.127	0.151	0.085	0.130	0.056	0.075	0.036	0.076	0.756	1.447
Q9HB97	Alpha-parvin OS=Rattus norvegicus GN=Parva PE=1 SV=2 - [PARVA_RAT]	42.3	0.633	0.893	0.373	1.033	0.883	0.013	0.013	0.013	0.013	0.013	0.915	0.845	1.537	3.039	0.748	3.198	0.596	1.251	1.239	0.770	0.009	0.308	0.259	0.269	0.259	0.277	0.009	0.048	0.009	0.038	0.917	1.101
Q9JJ19	Na(+)/H(+) exchange regulatory cofactor NHE-RF1 OS=Rattus norvegicus OX=10116 GN=Slc9a3r1 PE=1 SV=3 - [NHRF1_RAT]	38.8	0.687	0.756	0.483	1.021	0.957	0.013	0.013	0.013	0.013	0.013	0.844	0.663	1.335	2.924	0.707	2.206	0.805	1.060	1.111	0.740	0.009	0.240	0.470	0.438	0.127	0.171	0.009	0.057	0.009	0.056	0.756	1.426
Q9JJ22	Endoplasmic reticulum aminopeptidase 1 OS=Rattus norvegicus GN=Erap1 PE=2 SV=2 - [ERAP1_RAT]	106.4	0.851	0.801	0.697	0.990	0.855	0.013	0.013	0.013	0.013	0.013	0.013	0.013	0.013	0.013	0.013	1.433	1.040	1.306	1.280	1.441	0.009	0.135	0.009	0.454	0.014	0.039	#DIV/0!	#DIV/0!	0.009	0.046	0.009	0.128
Q9JLH7	CDK5 regulatory subunit-associated protein 3 OS=Rattus norvegicus OX=10116 GN=Cdk5rap3 PE=1 SV=1 - [CK5P3_RAT]	57.0	1.619	0.866	0.704	1.349	1.082	0.013	0.013	0.013	0.013	0.013	1.017	1.207	2.173	1.896	1.206	2.183	1.024	1.985	1.286	1.229	0.009	0.053	0.259	0.260	0.259	0.270	0.009	0.043	0.009	0.031	0.470	1.440
Q9QXQ0	Alpha-actinin-4 OS=Rattus norvegicus GN=Actn4 PE=1 SV=2 - [ACTN4_RAT]	104.8	0.645	0.779	0.601	0.809	0.840	1.002	0.497	0.667	0.841	0.947	0.763	0.928	1.686	2.383	1.069	2.093	0.875	1.155	1.008	1.088	0.354	1.028	0.056	0.083	0.014	0.041	0.127	0.122	0.036	0.074	0.917	1.182
Q9QYU2	Elongation factor Ts, mitochondrial OS=Rattus norvegicus OX=10116 GN=Tsfm PE=2 SV=1 - [EFTS_RAT]	35.2	0.013	0.013	0.013	0.013	0.013	0.013	0.013	0.013	0.013	0.013	0.013	0.013	0.013	0.013	0.013	1.800	0.917	1.559	1.124	1.408	#DIV/0!	#DIV/0!	#DIV/0!	#DIV/0!	0.009	0.053	#DIV/0!	#DIV/0!	0.009	0.043	0.009	0.111
Q9WUJ3	Myomegalin OS=Rattus norvegicus GN=Pde4dip PE=1 SV=1 - [MYOME_RAT]	261.9	0.013	0.013	0.013	0.013	0.013	0.013	0.013	0.013	0.013	0.013	0.013	0.013	0.013	0.013	0.013	1.798	0.970	0.973	0.775	0.835	#DIV/0!	#DIV/0!	#DIV/0!	#DIV/0!	0.009	0.085	#DIV/0!	#DIV/0!	0.009	0.071	0.009	0.235
Q9WV25	Poly(U)-binding-splicing factor PUF60 OS=Rattus norvegicus GN=Puf60 PE=2 SV=2 - [PUF60_RAT]	60.2	0.795	0.962	0.463	1.111	1.134	1.001	0.512	0.741	0.879	1.002	0.996	0.879	2.036	1.822	1.001	2.023	0.950	1.493	1.525	1.222	0.605	0.991	0.259	0.267	0.056	0.092	0.184	0.159	0.036	0.070	0.756	1.309
Q9Z2J4	Nexilin OS=Rattus norvegicus GN=Nexn PE=1 SV=1 - [NEXN_RAT]	78.3	0.013	0.013	0.013	0.013	0.013	1.134	0.680	0.658	1.273	1.361	0.013	0.013	0.013	0.013	0.013	0.013	0.013	0.013	0.013	0.013	0.009	0.059	#DIV/0!	#DIV/0!	#DIV/0!	#DIV/0!	0.009	0.107	0.009	0.083	#DIV/0!	#DIV/0!
Q9Z311	Enoyl-[acyl-carrier-protein] reductase, mitochondrial OS=Rattus norvegicus OX=10116 GN=Mecr PE=1 SV=1 - [MECR_RAT]	40.3	0.608	0.996	0.425	1.085	1.008	0.780	0.358	0.470	1.020	1.065	0.945	1.049	1.989	2.709	1.186	2.740	0.790	1.367	1.366	Filter	0.756	1.069	0.085	0.109	0.056	0.093	0.056	0.068	0.036	0.070	0.756	1.213

Table S7. Extracted protein profile of c-I (Effect of Nutrition, common, Male)

Accession	Description	MW [kDa]	Expression level																				p<0.05, q<0.10											
			FGR-Good-Male					FGR-Poor-Male					Sham-Good-Male					Sham-Poor-Male					Steel-Dwass & FDR											
			#21	#22	#23	#24	#25	#26	#27	#28	#29	#30	#31	#32	#33	#34	#35	#36	#37	#38	#39	#40	FGR-GN vs FGR-PN		FGR-GN vs Sham-GN		FGR-GN vs Sham-PN		FGR-PN vs Sham-GN		FGR-PN vs Sham-PN		Sham-GN vs Sham-PN	
Filter	Filter	Filter	Filter	Filter	Filter	Filter	Filter	Filter	Filter	Filter	Filter	Filter	Filter	Filter	Filter	Filter	Filter	Filter	Filter	Filter	Filter	Filter	p-value	q-value	p	q-value	p	q-value	p	q-value	p	q-value	p	q-value
P04157	Receptor-type tyrosine-protein phosphatase C OS=Rattus norvegicus OX=10116 GN=Ptprc PE=1 SV=2 - [PTPRC_RAT]	143.2	1.581	1.172	2.197	4.978	1.063	0.013	0.013	0.013	0.013	0.013	2.240	0.887	0.321	0.357	2.753	0.013	0.013	0.013	0.013	0.013	0.009	0.0212	0.354	0.447	0.009	0.040	0.009	0.037	#DIV/0!	#DIV/0!	0.009	0.034
Q5TKR9	Histone acetyltransferase KAT6A OS=Rattus norvegicus OX=10116 GN=Kat6a PE=1 SV=2 - [KAT6A_RAT]	223.2	0.013	0.013	0.013	0.013	0.013	0.724	0.850	0.934	0.841	4.188	0.755	0.977	0.906	1.388	2.835	0.013	0.013	0.013	0.013	0.013	0.009	0.0216	0.009	0.024	#DIV/0!	#DIV/0!	0.470	0.636	0.009	0.015	0.009	0.034
Q62951	Dihydropyrimidinase-related protein 4 (Fragment) OS=Rattus norvegicus GN=Dpysl4 PE=1 SV=1 - [DPYL4_RAT]	61.0	1.872	0.805	2.583	1.132	1.067	0.013	0.013	0.013	0.013	0.013	0.013	0.013	0.013	0.013	0.013	0.558	1.544	1.378	1.266	0.922	0.009	0.0217	0.009	0.024	0.605	0.810	#DIV/0!	#DIV/0!	0.009	0.016	0.009	0.036
Q9ERE4	Golgi phosphoprotein 3 OS=Rattus norvegicus GN=Golph3 PE=1 SV=1 - [GOLP3_RAT]	33.8	0.013	0.013	0.013	0.013	0.013	0.729	2.200	1.804	0.280	1.717	0.728	0.980	0.975	2.073	0.632	0.013	0.013	0.013	0.013	0.013	0.009	0.0219	0.009	0.027	#DIV/0!	#DIV/0!	0.605	0.670	0.009	0.015	0.009	0.038
P97779	Hyaluronan-mediated motility receptor OS=Rattus norvegicus OX=10116 GN=Hmmr PE=2 SV=1 - [HMMR_RAT]	57.8	1.202	1.338	1.605	0.773	0.915	0.013	0.013	0.013	0.013	0.013	1.013	1.645	0.907	1.131	0.443	0.013	0.013	0.013	0.013	0.013	0.009	0.0224	0.605	0.552	0.009	0.043	0.009	0.045	#DIV/0!	#DIV/0!	0.009	0.041
P25235	Dolichyl-diphosphooligosaccharide--protein glycosyltransferase subunit 2 OS=Rattus norvegicus OX=10116 GN=Rpn2 PE=2 SV=2 - [RPN2_RAT]	69.0	0.625	0.962	1.868	1.419	0.886	0.013	0.013	0.013	0.013	0.013	0.997	1.185	1.255	1.357	0.526	0.013	0.013	0.013	0.013	0.013	0.009	0.0227	0.917	0.617	0.009	0.044	0.009	0.043	#DIV/0!	#DIV/0!	0.009	0.039
D3ZC96	Tetratricopeptide repeat protein 39B OS=Rattus norvegicus GN=Ttc39b PE=3 SV=1 - [TT39B_RAT]	70.1	0.013	0.013	0.013	0.013	0.013	0.899	1.786	0.798	0.670	1.390	0.655	0.697	1.209	1.654	0.246	0.013	0.013	0.013	0.013	0.013	0.009	0.0231	0.009	0.046	#DIV/0!	#DIV/0!	0.354	0.590	0.009	0.016	0.009	0.063
P97675	Ectonucleotide pyrophosphatase/phosphodiesterase family member 3 OS=Rattus norvegicus GN=Enpp3 PE=1 SV=2 - [ENPP3_RAT]	99.0	0.013	0.013	0.013	0.013	0.013	0.712	1.810	1.112	0.562	1.235	1.051	1.186	1.113	1.740	0.817	0.013	0.013	0.013	0.013	0.013	0.009	0.0233	0.009	0.025	#DIV/0!	#DIV/0!	0.756	0.689	0.009	0.017	0.009	0.035
Q8CGV7	Thiamine-triphosphatase OS=Rattus norvegicus GN=Thtpa PE=2 SV=3 - [THTPA_RAT]	24.5	1.150	0.952	1.704	0.455	0.799	0.013	0.013	0.013	0.013	0.013	0.013	0.013	0.013	0.013	0.013	0.730	1.031	1.409	0.899	1.064	0.009	0.0254	0.009	0.031	0.917	0.844	#DIV/0!	#DIV/0!	0.009	0.017	0.009	0.042
O35828	Coronin-7 OS=Rattus norvegicus GN=Coro7 PE=1 SV=2 - [CORO7_RAT]	100.7	0.962	0.935	1.663	0.801	0.695	0.013	0.013	0.013	0.013	0.013	0.931	1.104	0.823	1.166	0.647	0.013	0.013	0.013	0.013	0.013	0.009	0.0255	0.917	0.624	0.009	0.048	0.009	0.054	#DIV/0!	#DIV/0!	0.009	0.053
Q2PQA9	Kinesin-1 heavy chain OS=Rattus norvegicus GN=Kif5b PE=1 SV=1 - [KINH_RAT]	109.5	0.013	0.013	0.013	0.013	0.013	0.810	1.625	0.822	0.631	1.125	0.601	0.829	1.067	1.307	0.372	0.013	0.013	0.013	0.013	0.013	0.009	0.0257	0.009	0.064	#DIV/0!	#DIV/0!	0.605	0.687	0.009	0.018	0.009	0.081
Q6AY23	Pyrroline-5-carboxylate reductase 2 OS=Rattus norvegicus OX=10116 GN=Pycr2 PE=2 SV=1 - [P5CR2_RAT]	33.7	0.013	0.013	0.013	0.013	0.013	0.673	1.638	1.008	0.168	1.508	0.702	1.098	1.317	1.768	0.256	0.013	0.013	0.013	0.013	0.013	0.009	0.0258	0.009	0.029	#DIV/0!	#DIV/0!	0.756	0.689	0.009	0.018	0.009	0.041
P27791	cAMP-dependent protein kinase catalytic subunit alpha OS=Rattus norvegicus GN=Prkaca PE=1 SV=2 - [KAPCA_RAT]	40.6	0.013	0.013	0.013	0.013	0.013	0.876	1.219	1.415	0.427	0.999	0.551	1.272	1.108	1.309	0.313	0.013	0.013	0.013	0.013	0.013	0.009	0.0263	0.009	0.041	#DIV/0!	#DIV/0!	0.917	0.723	0.009	0.018	0.009	0.059
Q8VID1	Dehydrogenase/reductase SDR family member 4 OS=Rattus norvegicus GN=Dhrs4 PE=2 SV=2 - [DHRS4_RAT]	29.8	0.944	0.667	1.552	0.930	0.790	0.013	0.013	0.013	0.013	0.013	0.726	0.999	0.800	1.006	0.597	0.013	0.013	0.013	0.013	0.013	0.009	0.0269	0.756	0.596	0.009	0.050	0.009	0.090	#DIV/0!	#DIV/0!	0.009	0.087
P54311	Guanine nucleotide-binding protein G(I)/G(S)/G(T) subunit beta-1 OS=Rattus norvegicus GN=Gnb1 PE=1 SV=4 - [GBB1_RAT]	37.4	0.013	0.013	0.013	0.013	0.013	0.700	1.523	1.122	0.292	1.217	0.851	1.300	1.122	1.729	0.334	0.013	0.013	0.013	0.013	0.013	0.009	0.0272	0.009	0.027	#DIV/0!	#DIV/0!	0.605	0.672	0.009	0.019	0.009	0.039
Q62764	Y-box-binding protein 3 OS=Rattus norvegicus OX=10116 GN=Ybx3 PE=1 SV=1 - [YBOX3_RAT]	38.8	0.013	0.013	0.013	0.013	0.013	1.270	1.058	1.457	0.443	0.566	1.834	1.612	1.070	1.409	0.664	0.013	0.013	0.013	0.013	0.013	0.009	0.0278	0.009	0.024	#DIV/0!	#DIV/0!	0.184	0.451	0.009	0.019	0.009	0.034
Q9EQP5	Prolargin OS=Rattus norvegicus GN=Prelp PE=2 SV=1 - [PRELP_RAT]	43.2	0.013	0.013	0.013	0.013	0.013	0.807	1.347	1.266	0.223	1.149	0.512	0.779	0.969	1.812	0.200	0.013	0.013	0.013	0.013	0.013	0.009	0.0280	0.009	0.056	#DIV/0!	#DIV/0!	0.470	0.659	0.009	0.020	0.009	0.076
Q99P74	Ras-related protein Rab-27B OS=Rattus norvegicus OX=10116 GN=Rab27b PE=2 SV=3 - [RB27B_RAT]	24.6	0.013	0.013	0.013	0.013	0.013	0.575	1.522	1.302	0.251	1.139	0.875	1.104	0.814	1.904	0.412	0.013	0.013	0.013	0.013	0.013	0.009	0.0281	0.009	0.030	#DIV/0!	#DIV/0!	0.917	0.718	0.009	0.020	0.009	0.043
P28492	Glutaminase liver isoform, mitochondrial OS=Rattus norvegicus GN=Gls2 PE=2 SV=3 - [GLSL_RAT]	66.2	0.013	0.013	0.013	0.013	0.013	0.813	1.167	1.486	0.309	0.978	0.730	0.907	0.958	1.239	0.444	0.013	0.013	0.013	0.013	0.013	0.009	0.0282	0.009	0.056	#DIV/0!	#DIV/0!	0.605	0.693	0.009	0.020	0.009	0.075
Q91ZN1	Coronin-1A OS=Rattus norvegicus GN=Coro1a PE=1 SV=3 - [COR1A_RAT]	51.0	0.013	0.013	0.013	0.013	0.013	0.713	1.578	1.572	0.226	0.647	0.178	0.625	0.855	1.526	0.013	0.013	0.013	0.013	0.013	0.013	0.009	0.0284	0.025	0.064	#DIV/0!	#DIV/0!	0.259	0.601	0.009	0.020	0.025	0.092
P68182	cAMP-dependent protein kinase catalytic subunit beta OS=Rattus norvegicus GN=Prkacb PE=1 SV=2 - [KAPCB_RAT]	40.7	0.013	0.013	0.013	0.013	0.013	0.862	1.141	1.387	0.235	1.063	0.806	0.854	0.835	1.082	0.554	0.013	0.013	0.013	0.013	0.013	0.009	0.0286	0.009	0.067	#DIV/0!	#DIV/0!	0.259	0.552	0.009	0.020	0.009	0.086
O35112	CD166 antigen OS=Rattus norvegicus GN=Alcam PE=1 SV=1 - [CD166_RAT]	65.0	0.013	0.013	0.013	0.013	0.013	0.717	1.390	1.199	0.290	1.090	0.841	1.054	0.955	1.881	0.376	0.013	0.013	0.013	0.013	0.013	0.009	0.0288	0.009	0.030	#DIV/0!	#DIV/0!	0.917	0.719	0.009	0.020	0.009	0.043
Q5EGY4	Synaptobrevin homolog YKT6 OS=Rattus norvegicus GN=Ykt6 PE=1 SV=1 - [YKT6_RAT]	22.4	0.013	0.013	0.013	0.013	0.013	0.842	1.228	1.265	0.285	1.054	0.539	0.996	0.851	1.319	0.331	0.013	0.013	0.013	0.013	0.013	0.009	0.0293	0.009	0.078	#DIV/0!	#DIV/0!	0.756	0.732	0.009	0.021	0.009	0.0998
P18645	UDP-glucose 4-epimerase OS=Rattus norvegicus GN=Gale PE=2 SV=1 - [GALE_RAT]	38.2	0.013	0.013	0.013	0.013	0.013	0.844	1.160	1.296	0.291	1.082	0.743	1.237	0.854	1.465	0.626	0.013	0.013	0.013	0.013	0.013	0.009	0.0295	0.009	0.033	#DIV/0!	#DIV/0!	0.917	0.722	0.009	0.021	0.009	0.046
Q10728	Protein phosphatase 1 regulatory subunit 12A OS=Rattus norvegicus GN=Ppp1r12a PE=1 SV=2 - [MYPT1_RAT]	115.2	1.135	0.784	1.478	0.642	0.613	0.013	0.013	0.013	0.013	0.013	0.013	0.013	0.013	0.013	0.013	0.630	0.977	1.072	0.735	0.933	0.009	0.0302	0.009	0.038	0.917	0.865	#DIV/0!	#DIV/0!	0.009	0.024	0.009	0.069
Q6IG02	Keratin, type II cytoskeletal 2 epidermal OS=Rattus norvegicus GN=Krt2 PE=3 SV=1 - [K22E_RAT]	69.1	0.013	0.013	0.013	0.013	0.013	1.056	1.117	0.802	0.560	1.108	0.932	0.795	1.322	1.623	0.756	0.013	0.013	0.013	0.013	0.013	0.009	0.0306	0.009	0.027	#DIV/0!	#DIV/0!	0.756	0.690	0.009	0.021	0.009	0.038
Q07984	Translocon-associated protein subunit delta OS=Rattus norvegicus OX=10116 GN=Ssr4 PE=2 SV=1 - [SSRD_RAT]	19.0	0.013	0.013	0.013	0.013	0.013	0.661	1.414	1.123	0.290	1.117	0.634	1.024	1.411	2.133	0.352	0.013	0.013	0.013	0.013	0.013	0.009	0.0311	0.009	0.026	#DIV/0!	#DIV/0!	0.917	0.717	0.009	0.022	0.009	0.037
A2VCX1	TIP41-like protein OS=Rattus norvegicus GN=Tiprl PE=1 SV=1 - [TIPRL_RAT]	31.2	1.224	0.887	1.513	1.397	1.102	0.661	0.605	0.835	0.231	0.601	0.865	0.883	0.928	1.365	0.601	0.013	0.013	0.013	0.013	0.013	0.014	0.0320	0.085	0.199	0.009	0.042	0.085	0.290	0.009	0.129	0.009	0.055
Q6B345	Protein S100-A11 OS=Rattus norvegicus GN=S100a11 PE=3 SV=1 - [S10AB_RAT]	11.1	0.013	0.013	0.013	0.013	0.013	0.806	1.205	1.133	0.207	1.156	0.515	0.563	1.105	1.926	0.264	0.013	0.013	0.013	0.013	0.013	0.009	0.0326	0.009	0.051	#DIV/0!	#DIV/0!	0.605	0.702	0.009	0.023	0.009	0.069
P85125	Polymerase I and transcript release factor OS=Rattus norvegicus GN=Ptrf PE=1 SV=1 - [PTRF_RAT]	43.9	0.013	0.013	0.013	0.013	0.013	0.787	1.209	1.116	0.294	1.041	0.672	0.913	0.999	1.278	0.430	0.013	0.013	0.013	0.013	0.013	0.009	0.0339	0.009	0.055	#DIV/0!	#DIV/0!	0.756	0.729	0.009	0.023	0.009	0.073
Q9Z336	Dynein light chain Tctex-type 1 OS=Rattus norvegicus GN=Dynlt1 PE=1 SV=1 - [DYLT1_RAT]	12.4	0.013	0.013	0.013	0.013	0.013	0.636	1.401	1.058	0.118	1.189	0.338	0.784	1.418	1.356	0.142	0.013	0.013	0.013	0.013	0.013	0.009	0.0341	0.009	0.078	#DIV/0!	#DIV/0!	0.917	0.771	0.009	0.023	0.009	0.099
Q62636	Ras-related protein Rap-1b OS=Rattus norvegicus GN=Rap1b PE=2 SV=2 - [RAP1B_RAT]	20.8	0.013	0.013	0.013	0.013	0.013	0.736	1.088	1.174	0.340	0.972	0.620	0.961	0.846	1.624	0.286	0.013	0.013	0.013	0.013	0.013	0.009	0.0354	0.009	0.052	#DIV/0!	#DIV/0!	0.605	0.716	0.009	0.024	0.009	0.070
Q6P7A9	Lysosomal alpha-glucosidase OS=Rattus norvegicus GN=Gaa PE=2 SV=1 - [LYAG_RAT]	106.1	0.891	0.653	1.429	0.692	0.601	0.013	0.013	0.013	0.013	0.013	0.013	0.013	0.013	0.013	0.013	0.716	0.996	1.276	0.856	1.220	0.009	0.0367	0.009	0.058	0.259	0.638	#DIV/0!	#DIV/0!	0.009	0.018	0.009	0.044
Q5PPH0	Enolase-phosphatase E1 OS=Rattus norvegicus GN=Enoph1 PE=2 SV=1 - [ENOPH_RAT]	28.9	1.087	0.830	1.205	0.503	0.637	0.013	0.013	0.013	0.013	0.013	0.677	0.956	1.047	1.231	0.291	0.013	0.013	0.013	0.013	0.013	0.009	0.0370	0.917	0.649	0.009	0.064	0.009	0.082	#DIV/0!	#DIV/0!	0.009	0.079
Q62848	ADP-ribosylation factor GTPase-activating protein 1 OS=Rattus norvegicus GN=Arfgap1 PE=1 SV=1 - [ARFG1_RAT]	45.4	0.013	0.013	0.013	0.013	0.013	0.819	1.041	1.060	0.391	0.869	0.013	0.013	0.013	0.013	0.013	0.777	1.160	1.107	1.072	1.373	0.009	0.0409	#DIV/0!	#DIV/0!	0.009	0.045	0.009	0.087	0.085	0.099	0.009	0.038
Q7TNY6	Golgi resident protein GCP60 OS=Rattus norvegicus OX=10116 GN=Acbd3 PE=1 SV=3 - [GCP60_RAT]	60.4	0.013	0.013	0.013	0.013	0.013	0.665	1.072	1.081	0.478	0.879	1.077	1.009	0.694	1.280	0.509	0.013	0.013	0.013	0.013	0.013	0.009	0.0415	0.009	0.040	#DIV/0!	#DIV/0!	0.605	0.710	0.009	0.027	0.009	0.058
Q66HA6	ADP-ribosylation factor-like protein 8B OS=Rattus norvegicus GN=Arl8b PE=2 SV=1 - [ARL8B_RAT]	21.5	0.013	0.013	0.013	0.013	0.013	0.579	1.067	0.946	0.251	1.297	0.887	1.060	0.629	1.533	0.532	0.013	0.013	0.013	0.013	0.013	0.009	0.0423	0.009	0.038	#DIV/0!	#DIV/0!	0.917	0.744	0.009	0.027	0.009	0.055
B2RZD7	LYR motif-containing protein 9 OS=Rattus norvegicus OX=10116 GN=Lyrm9 PE=3 SV=1 - [LYRM9_RAT]	9.4	0.721	0.696	1.452	0.709	0.545	0.013	0.013	0.013	0.013	0.013	0.013	0.013	0.013	0.013	0.013	0.486	0.793	1.130	0.989	0.895	0.009	0.0426	0.009	0.068	0.470	0.801	#DIV/0!	#DIV/0!	0.009	0.025	0.009	0.074
Q9JIL3	Interleukin enhancer-binding factor 3 OS=Rattus norvegicus GN=Ilf3 PE=1 SV=2 - [ILF3_RAT]	95.9	0.013	0.013	0.013	0.013	0.013	0.895	0.954	1.183	0.321	0.770	0.710	0.994	1.037	1.146	0.380	0.013	0.013	0.013	0.013	0.013	0.009	0.0429	0.009	0.057	#DIV/0!	#DIV/0!	0.756	0.762	0.009	0.027	0.009	0.077
Q6AY46	tRNA (adenine(58)-N(1))-methyltransferase catalytic subunit TRMT61A OS=Rattus norvegicus OX=10116 GN=Trmt61a PE=2 SV=1 - [TRM61_RAT]	31.6	0.013	0.013	0.013	0.013	0.013	0.798	0.702	1.008	1.023	0.581	0.883	0.904	1.020	1.393	0.284	0.013	0.013	0.013	0.013	0.013	0.009	0.0435	0.009	0.045	#DIV/0!	#DIV/0!	0.605	0.722	0.009	0.028	0.009	0.063
O88656	Actin-related protein 2/3 complex subunit 1B OS=Rattus norvegicus GN=Arpc1b PE=2 SV=3 - [ARC1B_RAT]	41.0	0.013	0.013	0.013	0.013	0.013	0.817	1.032	1.256	0.144	0.854	0.013	0.013	0.013	0.013	0.013	0.451	0.838	1.129	1.163	0.980	0.009	0.0442	#DIV/0!	#DIV/0!	0.009	0.055	0.009	0.092	0.756	0.336	0.009	0.059
O88637	Ethanolamine-phosphate cytidylyltransferase OS=Rattus norvegicus GN=Pcyt2 PE=1 SV=1 - [PCY2_RAT]	45.2	0.013	0.013	0.013	0.013	0.013	0.716	1.076	1.252	0.115	0.909	0.712	0.956	0.883	1.435	0.258	0.013	0.013	0.013	0.013	0.013	0.009	0.0455	0.009	0.059	#DIV/0!	#DIV/0!	0.917	0.783	0.009	0.029	0.009	0.078
P33124	Long-chain-fatty-acid--CoA ligase 6 OS=Rattus norvegicus OX=10116 GN=Acsl6 PE=1 SV=1 - [ACSL6_RAT]	78.1	0.013	0.013	0.013	0.013	0.013	0.520	1.405	0.981	0.069	1.087	1.206	1.277	1.040	1.703	0.703	0.013	0.013	0.013	0.013	0.013	0.009	0.0458	0.009	0.025	#DIV/0!	#DIV/0!	0.259	0.520	0.009	0.029	0.009	0.035
Q78P75	Dynein light chain 2, cytoplasmic OS=Rattus norvegicus GN=Dynll2 PE=1 SV=1 - [DYL2_RAT]	10.3	0.013	0.013	0.013	0.013	0.013	0.701	1.000	1.273	0.271	0.805	0.417	0.913	1.076	2.074	0.200	0.013	0.013	0.013	0.013	0.013	0.009	0.0469	0.009	0.037	#DIV/0!	#DIV/0!	0.917	0.747	0.009	0.030	0.009	0.053
Q9JJ50	Hepatocyte growth factor-regulated tyrosine kinase substrate OS=Rattus norvegicus GN=Hgs PE=1 SV=1 - [HGS_RAT]	86.2	0.013	0.013	0.013	0.013	0.013	0.620	0.985	1.068	0.216	1.151	0.980	1.159	0.819	1.540	0.565	0.013	0.013	0.013	0.013	0.013	0.009	0.0476	0.009	0.030	#DIV/0!	#DIV/0!	0.605	0.692	0.009	0.030	0.009	0.044
P50878	60S ribosomal protein L4 OS=Rattus norvegicus GN=Rpl4 PE=1 SV=3 - [RL4_RAT]	47.2	0.013	0.013	0.013	0.013	0.013	0.689	0.815	1.086	0.253	1.154	0.764	1.207	0.946	0.669	0.464	0.013	0.013	0.013	0.013	0.013	0.009	0.0492	0.009	0.076	#DIV/0!	#DIV/0!	0.917	0.796	0.009	0.031	0.009	0.096
B5DF89	Cullin-3 OS=Rattus norvegicus GN=Cul3 PE=1 SV=2 - [CUL3_RAT]	88.9	0.013	0.013	0.013	0.013	0.013	0.688	1.133	1.116	0.188	0.847	0.703	1.073	0.756	1.188	0.356	0.013	0.013	0.013	0.013	0.013	0.009	0.0504	0.009	0.071	#DIV/0!	#DIV/0!	0.917	0.796	0.009	0.032	0.009	0.090
P31000	Vimentin OS=Rattus norvegicus GN=Vim PE=1 SV=2 - [VIME_RAT]	53.7	0.649	0.588	1.566	0.651	0.487	0.013	0.013	0.013	0.013	0.013	0.601	0.794	1.487	1.541	0.204	0.013	0.013	0.013	0.013	0.013	0.009	0.0512	0.756	0.614	0.009	0.080	0.009	0.058	#DIV/0!	#DIV/0!	0.009	0.056
Q63798	Proteasome activator complex subunit 2 OS=Rattus norvegicus GN=Psme2 PE=2 SV=3 - [PSME2_RAT]	26.8	0.013	0.013	0.013	0.013	0.013	0.750	1.034	1.186	0.212	0.744	0.647	0.822	0.988	1.170	0.677	0.013	0.013	0.013	0.013	0.013	0.009	0.0521	0.009	0.054	#DIV/0!	#DIV/0!	0.917	0.789	0.009	0.033	0.009	0.071
Q9JMB5	Proteasomal ubiquitin receptor ADRM1 OS=Rattus norvegicus GN=Adrm1 PE=2 SV=2 - [ADRM1_RAT]	42.1	0.013	0.013	0.013	0.013	0.013	0.919	0.654	1.556	0.161	0.604	0.725	1.232	1.071	1.747	0.632	0.013	0.013	0.013	0.013	0.013	0.009	0.0530	0.009	0.027	#DIV/0!	#DIV/0!	0.259	0.530	0.009	0.034	0.009	0.038
P41565	Isocitrate dehydrogenase [NAD] subunit gamma 1, mitochondrial OS=Rattus norvegicus GN=Idh3g PE=2 SV=2 - [IDHG1_RAT]	42.8	0.013	0.013	0.013	0.013	0.013	0.552	1.243	0.920	0.027	1.106	0.534	0.851	1.075	1.369	0.236	0.013	0.013	0.013	0.013	0.013	0.009	0.0540	0.009	0.072	#DIV/0!	#DIV/0!	0.917	0.804	0.009	0.035	0.009	0.092
Q62698	Cytoplasmic dynein 1 light intermediate chain 2 OS=Rattus norvegicus GN=Dync1li2 PE=1 SV=1 - [DC1L2_RAT]	54.7	0.013	0.013	0.013	0.013	0.013	0.527	0.696	1.511	0.013	0.762	0.910	0.672	1.153	2.786	0.225	0.013	0.013	0.013	0.013	0.013	0.025	0.0557	0.009	0.026	#DIV/0!	#DIV/0!	0.470	0.647	0.025	0.037	0.009	0.036
Q9JK25	CAP-Gly domain-containing linker protein 1 OS=Rattus norvegicus OX=10116 GN=Clip1 PE=1 SV=2 - [CLIP1_RAT]	148.2	0.013	0.013	0.013	0.013	0.013	1.063	0.395	1.392	0.013	0.429	0.567	1.024	1.250	1.816	0.269	0.013	0.013	0.013	0.013	0.013	0.025	0.0559	0.009	0.033	#DIV/0!	#DIV/0!	0.470	0.720	0.025	0.037	0.009	0.046
Q5XIE0	Acidic leucine-rich nuclear phosphoprotein 32 family member E OS=Rattus norvegicus OX=10116 GN=Anp32e PE=2 SV=1 - [AN32E_RAT]	29.4	1.035	0.674	1.253	0.408	0.461	0.013	0.013	0.013	0.013	0.013	0.534	0.674	1.222	1.694	0.173	0.013	0.013	0.013	0.013	0.013	0.009	0.0565	0.917	0.660	0.009	0.088	0.009	0.075	#DIV/0!	#DIV/0!	0.009	0.073
Q62871	Cytoplasmic dynein 1 intermediate chain 2 OS=Rattus norvegicus GN=Dync1i2 PE=1 SV=1 - [DC1I2_RAT]	71.1	0.013	0.013	0.013	0.013	0.013	0.689	0.959	1.152	0.167	0.853	0.889	0.932	0.996	1.220	0.546	0.013	0.013	0.013	0.013	0.013	0.009	0.0570	0.009	0.040	#DIV/0!	#DIV/0!	0.470	0.703	0.009	0.037	0.009	0.058
Q64620	Serine/threonine-protein phosphatase 6 catalytic subunit OS=Rattus norvegicus GN=Ppp6c PE=2 SV=2 - [PPP6_RAT]	35.1	0.013	0.013	0.013	0.013	0.013	0.763	0.825	0.978	0.461	0.697	0.989	1.055	1.000	1.188	0.433	0.013	0.013	0.013	0.013	0.013	0.009	0.0642	0.009	0.038	#DIV/0!	#DIV/0!	0.127	0.392	0.009	0.042	0.009	0.054
Q5FVL2	ER membrane protein complex subunit 8 OS=Rattus norvegicus OX=10116 GN=Emc8 PE=2 SV=1 - [EMC8_RAT]	23.4	0.709	0.796	1.134	0.519	0.562	0.013	0.013	0.013	0.013	0.013	0.013	0.013	0.013	0.013	0.013	0.765	0.657	0.966	0.727	0.943	0.009	0.0649	0.009	0.130	0.470	0.834	#DIV/0!	#DIV/0!	0.009	0.029	0.009	0.094
Q6UPE1	Electron transfer flavoprotein-ubiquinone oxidoreductase, mitochondrial OS=Rattus norvegicus OX=10116 GN=Etfdh PE=1 SV=1 - [ETFD_RAT]	68.2	0.013	0.013	0.013	0.013	0.013	0.633	1.055	0.722	0.332	0.970	0.896	0.969	0.843	1.445	0.431	0.013	0.013	0.013	0.013	0.013	0.009	0.0663	0.009	0.039	#DIV/0!	#DIV/0!	0.605	0.751	0.009	0.042	0.009	0.058
P84817	Mitochondrial fission 1 protein OS=Rattus norvegicus GN=Fis1 PE=1 SV=1 - [FIS1_RAT]	17.0	0.013	0.013	0.013	0.013	0.013	0.593	1.319	0.875	0.117	0.771	0.742	0.881	0.830	1.139	0.458	0.013	0.013	0.013	0.013	0.013	0.009	0.0693	0.009	0.075	#DIV/0!	#DIV/0!	0.756	0.813	0.009	0.044	0.009	0.095
P83941	Transcription elongation factor B polypeptide 1 OS=Rattus norvegicus GN=Tceb1 PE=1 SV=1 - [ELOC_RAT]	12.5	0.013	0.013	0.013	0.013	0.013	0.799	0.786	1.218	0.109	0.657	0.775	1.162	1.060	1.297	0.420	0.013	0.013	0.013	0.013	0.013	0.009	0.0772	0.009	0.036	#DIV/0!	#DIV/0!	0.470	0.714	0.009	0.049	0.009	0.050
Q03386	Ral guanine nucleotide dissociation stimulator OS=Rattus norvegicus OX=10116 GN=Ralgds PE=1 SV=1 - [GNDS_RAT]	98.8	1.050	0.837	2.034	1.169	0.842	0.663	0.834	0.869	0.359	0.698	0.796	1.079	0.893	1.264	0.660	0.013	0.013	0.013	0.013	0.013	0.036	0.0777	0.470	0.510	0.009	0.043	0.184	0.496	0.009	0.062	0.009	0.052
Q9EPF2	Cell surface glycoprotein MUC18 OS=Rattus norvegicus OX=10116 GN=Mcam PE=1 SV=2 - [MUC18_RAT]	71.3	0.013	0.013	0.013	0.013	0.013	0.769	0.819	0.908	0.420	0.640	0.537	0.942	1.169	1.592	0.484	0.013	0.013	0.013	0.013	0.013	0.009	0.0792	0.009	0.036	#DIV/0!	#DIV/0!	0.354	0.647	0.009	0.050	0.009	0.050
P52632	Signal transducer and activator of transcription 5B OS=Rattus norvegicus GN=Stat5b PE=1 SV=1 - [STA5B_RAT]	90.2	0.546	0.601	1.546	0.428	0.418	0.013	0.013	0.013	0.013	0.013	0.815	0.884	0.935	1.018	0.397	0.013	0.013	0.013	0.013	0.013	0.009	0.0802	0.470	0.571	0.009	0.112	0.009	0.098	#DIV/0!	#DIV/0!	0.009	0.098
Q9QWN8	Spectrin beta chain, non-erythrocytic 2 OS=Rattus norvegicus GN=Sptbn2 PE=1 SV=2 - [SPTN2_RAT]	270.9	0.013	0.013	0.013	0.013	0.013	0.427	1.030	0.886	0.269	0.890	0.926	1.158	0.709	1.185	0.512	0.013	0.013	0.013	0.013	0.013	0.009	0.0835	0.009	0.043	#DIV/0!	#DIV/0!	0.259	0.595	0.009	0.053	0.009	0.061
Q6QI44	N-alpha-acetyltransferase 25, NatB auxiliary subunit OS=Rattus norvegicus GN=Naa25 PE=2 SV=1 - [NAA25_RAT]	111.2	0.013	0.013	0.013	0.013	0.013	0.608	0.967	1.119	0.065	0.740	1.551	0.918	0.705	1.161	0.873	0.013	0.013	0.013	0.013	0.013	0.009	0.0871	0.009	0.029	#DIV/0!	#DIV/0!	0.259	0.563	0.009	0.053	0.009	0.041
Q66HL2	Src substrate cortactin OS=Rattus norvegicus GN=Cttn PE=1 SV=1 - [SRC8_RAT]	56.9	0.013	0.013	0.013	0.013	0.013	0.722	0.762	1.152	0.192	0.670	0.925	1.083	0.938	1.400	0.788	0.013	0.013	0.013	0.013	0.013	0.009	0.0884	0.009	0.029	#DIV/0!	#DIV/0!	0.085	0.280	0.009	0.054	0.009	0.042
P07323	Gamma-enolase OS=Rattus norvegicus GN=Eno2 PE=1 SV=2 - [ENOG_RAT]	47.1	1.028	0.660	1.291	0.180	0.305	0.013	0.013	0.013	0.013	0.013	0.774	0.994	0.881	0.976	0.495	0.013	0.013	0.013	0.013	0.013	0.009	0.0983	0.756	0.649	0.009	0.120	0.009	0.091	#DIV/0!	#DIV/0!	0.009	0.088

Table S8. Extracted protein profile of e-I (Effect of nutrition, common, Female)

												Expression level																						
Accession	Description	MW [kDa]	FGR-Good-Female					FGR-Poor_Female					Sham-Good-Female					Sham-Poor-Female					Steel-Dwass & FDR											
			#01	#02	#03	#04	#05	#06	#07	#08	#09	#10	#11	#12	#13	#14	#15	#16	#17	#18	#19	#20	FGR-GN vs FGR-PN		FGR-GN vs Sham-GN		FGR-GN vs Sham-PN		FGR-PN vs Sham-GN		FGR-PN vs Sham-PN		Sham-GN vs Sham-PN	
Filter	Filter	Filter	Filter	Filter	Filter	Filter	Filter	Filter	Filter	Filter	Filter	Filter	Filter	Filter	Filter	Filter	Filter	Filter	Filter	Filter	Filter	Filter	p-value	q-value	p	q-value	p	q-value	p	q-value	p	q-value	p	q-value
B2RYD2	Epithelial splicing regulatory protein 1 OS=Rattus norvegicus OX=10116 GN=Esrp1 PE=2 SV=2 - [ESRP1_RAT]	75.0	0.013	0.013	0.013	0.013	0.013	1.607	0.430	0.729	1.389	1.272	1.411	1.655	2.491	1.256	1.454	0.013	0.013	0.013	0.013	0.013	0.009	0.055	0.009	0.042	#DIV/0!	#DIV/0!	0.127	0.115	0.009	0.067	0.009	0.081
D4A4T9	Cysteine and histidine-rich domain-containing protein 1 OS=Rattus norvegicus GN=Chordc1 PE=3 SV=1 - [CHRD1_RAT]	37.3	0.013	0.013	0.013	0.013	0.013	1.291	0.548	0.687	0.970	0.994	1.065	0.995	2.010	2.225	1.015	0.013	0.013	0.013	0.013	0.013	0.009	0.091	0.009	0.053	#DIV/0!	#DIV/0!	0.056	0.068	0.009	0.142	0.009	0.095
G3V6U9	Actin-histidine N-methyltransferase OS=Rattus norvegicus OX=10116 GN=Setd3 PE=1 SV=1 - [SETD3_RAT]	67.4	0.650	1.136	0.358	1.528	1.390	0.013	0.013	0.013	0.013	0.013	0.013	0.013	0.013	0.013	0.013	2.560	0.956	1.521	1.607	1.468	0.009	0.061	0.009	0.129	0.127	0.157	#DIV/0!	#DIV/0!	0.009	0.028	0.009	0.084
G3V9D0	Protein O-glucosyltransferase 1 OS=Rattus norvegicus GN=Poglut1 PE=3 SV=1 - [PGLT1_RAT]	46.5	0.501	1.319	0.302	1.352	1.103	0.013	0.013	0.013	0.013	0.013	1.280	1.235	2.869	1.620	1.109	0.013	0.013	0.013	0.013	0.013	0.009	0.079	0.184	0.197	0.009	0.168	0.009	0.035	#DIV/0!	#DIV/0!	0.009	0.083
P49186	Mitogen-activated protein kinase 9 OS=Rattus norvegicus GN=Mapk9 PE=1 SV=1 - [MK09_RAT]	48.0	0.924	1.113	0.991	1.119	1.335	0.013	0.013	0.013	0.013	0.013	1.178	1.580	3.210	1.604	1.201	0.013	0.013	0.013	0.013	0.013	0.009	0.054	0.036	0.054	0.009	0.079	0.009	0.031	#DIV/0!	#DIV/0!	0.009	0.076
Q2A121	Alpha-ketoglutarate-dependent dioxygenase FTO OS=Rattus norvegicus GN=Fto PE=2 SV=1 - [FTO_RAT]	57.9	0.013	0.013	0.013	0.013	0.013	1.226	0.650	0.771	1.138	1.041	0.934	1.370	2.030	1.146	5.084	0.013	0.013	0.013	0.013	0.013	0.009	0.067	0.009	0.030	#DIV/0!	#DIV/0!	0.085	0.085	0.009	0.103	0.009	0.067
Q9JJ31	Cullin-5 OS=Rattus norvegicus GN=Cul5 PE=1 SV=3 - [CUL5_RAT]	90.8	0.013	0.013	0.013	0.013	0.013	1.379	0.633	0.742	1.091	0.705	2.256	2.549	2.759	2.633	2.102	1.556	0.747	1.903	1.885	0.982	0.009	0.084	0.009	0.028	0.009	0.047	0.014	0.025	0.085	0.095	0.014	0.091
Q9QZ81	Protein argonaute-2 OS=Rattus norvegicus GN=Ago2 PE=2 SV=2 - [AGO2_RAT]	97.3	0.013	0.013	0.013	0.013	0.013	1.031	0.658	0.544	1.466	1.319	1.238	1.160	2.435	1.461	1.228	0.013	0.013	0.013	0.013	0.013	0.009	0.062	0.009	0.050	#DIV/0!	#DIV/0!	0.259	0.207	0.009	0.092	0.009	0.092

Table S9. Extracted protein profile of c-II (Effect of Nutrition, only FGR, Male)

Accession	Description	MW [kDa]	Expression level: FGR-Good-Male					FGR-Poor-Male					Sham-Good-Male					Sham-Poor-Male					p<0.05, q<0.10 Steel-Dwass & FDR: FGR-GN vs FGR-PN		FGR-GN vs Sham-GN		FGR-GN vs Sham-PN		FGR-PN vs Sham-GN		FGR-PN vs Sham-PN		Sham-GN vs Sham-PN	
			#21	#22	#23	#24	#25	#26	#27	#28	#29	#30	#31	#32	#33	#34	#35	#36	#37	#38	#39	#40	p-value	q-value	p	q-value	p	q-value	p	q-value	p	q-value	p	q-value
Filter	Filter	Filter	Filter	Filter	Filter	Filter	Filter	Filter	Filter	Filter	Filter	Filter	Filter	Filter	Filter	Filter	Filter	Filter	Filter	Filter	Filter	Filter												
A2VCW5	Sodium-coupled neutral amino acid transporter 5 OS=Rattus norvegicus OX=10116 GN=Slc38a5 PE=1 SV=1 - [S38A5_RAT]	52.4	0.013	0.013	0.013	0.013	0.013	0.952	1.223	1.227	0.360	1.163	0.508	0.969	0.917	1.827	0.127	0.422	0.339	0.558	0.349	1.011	0.009	0.0264	0.009	0.051	0.009	0.381	0.470	0.646	0.085	0.106	0.470	0.916
A2VD13	Pentatricopeptide repeat-containing protein 1, mitochondrial OS=Rattus norvegicus OX=10116 GN=Ptcd1 PE=2 SV=1 - [PTCD1_RAT]	77.1	0.013	0.013	0.013	0.013	0.013	0.854	1.019	1.007	0.571	1.088	0.013	0.013	0.013	0.013	0.013	0.013	0.013	0.013	0.013	0.013	0.009	0.0319	#DIV/0!	#DIV/0!	#DIV/0!	#DIV/0!	0.009	0.061	0.009	0.022	#DIV/0!	#DIV/0!
B0BN18	Prefoldin subunit 2 OS=Rattus norvegicus GN=Pfdn2 PE=2 SV=1 - [PFD2_RAT]	16.6	0.013	0.013	0.013	0.013	0.013	0.816	1.021	1.164	0.222	0.956	0.505	0.771	1.178	1.769	0.167	1.147	0.810	1.710	0.788	0.897	0.009	0.0412	0.009	0.049	0.009	0.046	0.917	0.761	0.917	0.369	0.470	0.713
B0BNF1	Septin-8 OS=Rattus norvegicus GN=Sept8 PE=1 SV=1 - [SEPT8_RAT]	51.2	0.013	0.013	0.013	0.013	0.013	0.818	1.266	1.312	0.179	1.303	0.013	0.013	0.013	0.013	0.013	0.013	0.013	0.013	0.013	0.013	0.009	0.0270	#DIV/0!	#DIV/0!	#DIV/0!	#DIV/0!	0.009	0.049	0.009	0.019	#DIV/0!	#DIV/0!
B0LPN4	Ryanodine receptor 2 OS=Rattus norvegicus GN=Ryr2 PE=1 SV=2 - [RYR2_RAT]	562.6	0.013	0.013	0.013	0.013	0.013	0.928	0.756	0.953	2.236	0.937	0.013	0.013	0.013	0.013	0.013	0.013	0.013	0.013	0.013	0.013	0.009	0.0225	#DIV/0!	#DIV/0!	#DIV/0!	#DIV/0!	0.009	0.039	0.009	0.016	#DIV/0!	#DIV/0!
B0LT89	Serine/threonine-protein kinase 24 OS=Rattus norvegicus GN=Stk24 PE=2 SV=1 - [STK24_RAT]	48.0	0.013	0.013	0.013	0.013	0.013	0.599	1.232	1.117	0.201	1.065	0.334	0.951	0.845	1.621	0.203	0.013	0.013	0.013	0.013	0.013	0.009	0.0391	0.009	0.089	#DIV/0!	#DIV/0!	0.756	0.785	0.009	0.026	0.009	0.110
B2RYJ4	L-aminoadipate-semialdehyde dehydrogenase-phosphopantetheinyl transferase OS=Rattus norvegicus GN=Aasdhppt PE=2 SV=1 - [ADPPT_RAT]	35.8	0.689	0.599	2.123	1.042	0.753	0.013	0.013	0.013	0.013	0.013	0.013	0.013	0.013	0.013	0.013	0.013	0.013	0.013	0.013	0.013	0.009	0.0246	0.009	0.029	0.009	0.047	#DIV/0!	#DIV/0!	#DIV/0!	#DIV/0!	#DIV/0!	#DIV/0!
C9WPN6	Eukaryotic translation initiation factor 2 subunit 3, Y-linked OS=Rattus norvegicus OX=10116 GN=Eif2s3y PE=2 SV=2 - [IF2H_RAT]	51.1	0.013	0.013	0.013	0.013	0.013	0.953	0.896	1.790	0.050	0.799	0.013	0.013	0.013	0.013	0.013	0.013	0.013	0.013	0.013	0.013	0.009	0.0328	#DIV/0!	#DIV/0!	#DIV/0!	#DIV/0!	0.009	0.065	0.009	0.023	#DIV/0!	#DIV/0!
D3ZHV2	Microtubule-actin cross-linking factor 1 OS=Rattus norvegicus GN=Macf1 PE=1 SV=1 - [MACF1_RAT]	619.2	0.013	0.013	0.013	0.013	0.013	0.922	0.850	1.154	0.539	0.521	0.013	0.013	0.013	0.013	0.013	0.013	0.013	0.013	0.013	0.013	0.009	0.0500	#DIV/0!	#DIV/0!	#DIV/0!	#DIV/0!	0.009	0.103	0.009	0.032	#DIV/0!	#DIV/0!
D3ZLZ7	Inosine-5'-monophosphate dehydrogenase 1 OS=Rattus norvegicus GN=Impdh1 PE=3 SV=1 - [IMDH1_RAT]	55.3	0.013	0.013	0.013	0.013	0.013	0.784	1.588	1.255	0.322	1.181	0.865	0.884	1.098	1.644	0.365	0.754	0.790	1.405	0.594	1.119	0.009	0.0250	0.009	0.034	0.009	0.054	0.917	0.718	0.605	0.296	0.756	0.730
D3ZVU1	SprT-like domain-containing protein Spartan OS=Rattus norvegicus OX=10116 GN=Sprtn PE=3 SV=1 - [SPRTN_RAT]	55.0	0.013	0.013	0.013	0.013	0.013	0.656	1.215	0.861	0.129	0.790	0.013	0.013	0.013	0.013	0.013	0.013	0.013	0.013	0.013	0.013	0.009	0.0701	#DIV/0!	#DIV/0!	#DIV/0!	#DIV/0!	0.009	0.168	0.009	0.046	#DIV/0!	#DIV/0!
F1LU71	Methylglutaconyl-CoA hydratase, mitochondrial OS=Rattus norvegicus OX=10116 GN=Auh PE=1 SV=2 - [AUHM_RAT]	33.3	0.013	0.013	0.013	0.013	0.013	0.866	1.251	1.113	0.172	0.983	0.635	1.083	1.086	1.077	0.502	0.487	0.806	1.123	0.852	1.064	0.009	0.0344	0.009	0.049	0.009	0.063	0.756	0.727	0.605	0.304	0.756	0.755
F1M775	Protein diaphanous homolog 1 OS=Rattus norvegicus OX=10116 GN=Diaph1 PE=1 SV=3 - [DIAP1_RAT]	140.3	0.013	0.013	0.013	0.013	0.013	0.610	0.957	1.206	0.223	0.770	0.601	0.943	0.811	1.134	0.237	0.013	0.013	0.013	0.013	0.013	0.009	0.0598	0.009	0.127	#DIV/0!	#DIV/0!	0.917	0.820	0.009	0.039	0.009	0.151
O08701	Arginase-2, mitochondrial OS=Rattus norvegicus OX=10116 GN=Arg2 PE=1 SV=1 - [ARGI2_RAT]	38.6	0.013	0.013	0.013	0.013	0.013	0.836	1.011	1.228	0.482	0.909	0.013	0.013	0.013	0.013	0.013	0.275	0.407	0.589	0.438	0.504	0.009	0.0335	#DIV/0!	#DIV/0!	0.009	0.796	0.009	0.066	0.036	0.053	0.009	1.073
O08815	STE20-like serine/threonine-protein kinase OS=Rattus norvegicus GN=Slk PE=2 SV=1 - [SLK_RAT]	137.8	0.013	0.013	0.013	0.013	0.013	0.676	1.062	1.280	0.355	0.815	0.013	0.013	0.013	0.013	0.013	0.013	0.013	0.013	0.013	0.013	0.009	0.0401	#DIV/0!	#DIV/0!	#DIV/0!	#DIV/0!	0.009	0.085	0.009	0.027	#DIV/0!	#DIV/0!
O35142	Coatomer subunit beta' OS=Rattus norvegicus GN=Copb2 PE=1 SV=3 - [COPB2_RAT]	102.5	0.013	0.013	0.013	0.013	0.013	0.667	0.779	0.930	0.774	0.697	0.903	0.653	1.206	1.115	0.228	0.456	0.481	1.029	0.652	1.060	0.009	0.0544	0.009	0.069	0.009	0.097	0.756	0.802	0.605	0.324	0.470	0.835
O35162	Heat shock 70 kDa protein 13 OS=Rattus norvegicus GN=Hspa13 PE=2 SV=2 - [HSP13_RAT]	51.8	0.013	0.013	0.013	0.013	0.013	0.857	1.016	0.877	0.276	1.022	0.540	0.978	0.803	1.506	0.214	0.567	0.540	0.845	0.470	0.984	0.009	0.0473	0.009	0.077	0.009	0.127	0.605	0.764	0.259	0.208	0.756	0.837
O55159	Epithelial cell adhesion molecule OS=Rattus norvegicus OX=10116 GN=Epcam PE=1 SV=1 - [EPCAM_RAT]	35.2	0.013	0.013	0.013	0.013	0.013	0.713	1.324	0.840	0.164	1.608	0.013	0.013	0.013	0.013	0.013	0.013	0.013	0.013	0.013	0.013	0.009	0.0303	#DIV/0!	#DIV/0!	#DIV/0!	#DIV/0!	0.009	0.057	0.009	0.021	#DIV/0!	#DIV/0!
O88775	Embigin OS=Rattus norvegicus GN=Emb PE=1 SV=1 - [EMB_RAT]	37.0	0.013	0.013	0.013	0.013	0.013	0.837	1.580	1.338	0.398	1.217	0.467	0.760	0.923	1.536	0.288	0.626	0.600	0.752	0.420	1.050	0.009	0.0236	0.009	0.084	0.009	0.125	0.354	0.600	0.184	0.158	0.756	0.840
O88794	Pyridoxine-5'-phosphate oxidase OS=Rattus norvegicus OX=10116 GN=Pnpo PE=1 SV=1 - [PNPO_RAT]	30.2	1.351	0.956	1.781	0.835	0.759	0.612	0.701	0.877	0.175	0.621	0.829	1.228	0.880	1.534	0.824	0.653	1.348	1.428	1.096	0.939	0.036	0.0782	0.756	0.581	0.917	0.838	0.036	0.132	0.036	0.051	0.756	0.718
O88941	Mannosyl-oligosaccharide glucosidase OS=Rattus norvegicus OX=10116 GN=Mogs PE=1 SV=1 - [MOGS_RAT]	91.8	0.013	0.013	0.013	0.013	0.013	0.946	1.589	1.421	0.214	1.445	0.517	0.835	1.030	1.277	0.190	0.013	0.013	0.013	0.013	0.013	0.009	0.0228	0.009	0.103	#DIV/0!	#DIV/0!	0.184	0.459	0.009	0.016	0.009	0.126
P00388	NADPH--cytochrome P450 reductase OS=Rattus norvegicus OX=10116 GN=Por PE=1 SV=3 - [NCPR_RAT]	76.9	0.013	0.013	0.013	0.013	0.013	0.935	1.038	1.137	0.444	0.823	0.013	0.013	0.013	0.013	0.013	0.013	0.013	0.013	0.013	0.013	0.009	0.0346	#DIV/0!	#DIV/0!	#DIV/0!	#DIV/0!	0.009	0.071	0.009	0.024	#DIV/0!	#DIV/0!
P00762	Anionic trypsin-1 OS=Rattus norvegicus GN=Prss1 PE=1 SV=1 - [TRY1_RAT]	25.9	0.930	0.620	0.914	0.371	0.405	0.948	1.175	1.029	0.947	1.111	0.597	0.928	0.944	1.462	0.186	0.473	0.746	0.701	0.463	0.877	0.014	0.0321	0.470	0.574	0.917	0.968	0.127	0.379	0.014	0.022	0.354	0.851
P00774	Chymotrypsin-like elastase family member 2A OS=Rattus norvegicus OX=10116 GN=Cela2a PE=2 SV=1 - [CEL2A_RAT]	28.9	1.066	0.767	0.746	0.427	0.416	1.121	1.257	1.239	0.973	1.305	0.736	0.849	0.774	1.400	0.244	0.499	0.919	0.895	0.549	0.912	0.022	0.0508	0.605	0.630	0.470	0.862	0.127	0.374	0.014	0.021	0.756	0.820
P01835	Ig kappa chain C region, B allele OS=Rattus norvegicus PE=1 SV=1 - [KACB_RAT]	11.6	1.215	0.129	0.353	0.161	0.151	1.089	1.698	1.477	0.862	1.401	0.371	0.373	0.857	1.066	0.464	0.293	1.546	1.024	0.882	0.579	0.036	0.0785	0.127	0.294	0.127	0.456	0.022	0.085	0.184	0.154	0.470	0.881
P02692	Fatty acid-binding protein, liver OS=Rattus norvegicus OX=10116 GN=Fabp1 PE=1 SV=1 - [FABPL_RAT]	14.3	0.013	0.013	0.013	0.013	0.013	0.727	1.155	0.916	3.192	0.872	0.013	0.013	0.013	0.013	0.013	0.013	0.013	0.013	0.013	0.013	0.009	0.0219	#DIV/0!	#DIV/0!	#DIV/0!	#DIV/0!	0.009	0.037	0.009	0.015	#DIV/0!	#DIV/0!
P02764	Alpha-1-acid glycoprotein OS=Rattus norvegicus GN=Orm1 PE=2 SV=1 - [A1AG_RAT]	23.6	0.013	0.013	0.013	0.013	0.013	0.963	1.049	1.079	1.126	0.943	0.013	0.013	0.013	0.013	0.013	0.013	0.013	0.013	0.013	0.013	0.009	0.0248	#DIV/0!	#DIV/0!	#DIV/0!	#DIV/0!	0.009	0.045	0.009	0.017	#DIV/0!	#DIV/0!
P04550	Parathymosin OS=Rattus norvegicus OX=10116 GN=Ptms PE=1 SV=2 - [PTMS_RAT]	11.6	0.013	0.013	0.013	0.013	0.013	1.174	1.123	1.559	0.097	0.776	0.013	0.013	0.013	0.013	0.013	0.013	0.013	0.013	0.013	0.013	0.009	0.0285	#DIV/0!	#DIV/0!	#DIV/0!	#DIV/0!	0.009	0.053	0.009	0.020	#DIV/0!	#DIV/0!
P04631	Protein S100-B OS=Rattus norvegicus GN=S100b PE=1 SV=2 - [S100B_RAT]	10.7	0.013	0.013	0.013	0.013	0.013	0.718	0.744	1.150	2.659	0.505	0.013	0.013	0.013	0.013	0.013	0.013	0.013	0.013	0.013	0.013	0.009	0.0226	#DIV/0!	#DIV/0!	#DIV/0!	#DIV/0!	0.009	0.039	0.009	0.016	#DIV/0!	#DIV/0!
P05545	Serine protease inhibitor A3K OS=Rattus norvegicus GN=Serpina3k PE=1 SV=3 - [SPA3K_RAT]	46.5	1.031	0.572	0.912	0.230	0.377	1.187	1.536	1.251	1.396	1.796	0.364	0.593	0.983	1.357	0.190	0.212	0.510	0.397	0.273	0.700	0.014	0.0318	0.917	0.692	0.354	0.851	0.036	0.129	0.014	0.021	0.470	0.976
P06238	Alpha-2-macroglobulin OS=Rattus norvegicus GN=A2m PE=2 SV=2 - [A2MG_RAT]	163.7	0.943	0.767	3.931	0.481	0.595	0.013	0.013	0.013	0.013	0.013	0.013	0.013	0.013	0.013	0.013	0.013	0.013	0.013	0.013	0.013	0.009	0.0220	0.009	0.024	0.009	0.041	#DIV/0!	#DIV/0!	#DIV/0!	#DIV/0!	#DIV/0!	#DIV/0!
P06757	Alcohol dehydrogenase 1 OS=Rattus norvegicus GN=Adh1 PE=1 SV=3 - [ADH1_RAT]	39.6	0.262	0.250	0.375	0.083	0.180	0.477	0.533	0.498	0.283	0.558	0.486	0.475	0.411	0.579	0.177	0.013	0.013	0.013	0.013	0.013	0.022	0.0517	0.085	0.205	0.009	4.378	0.470	0.774	0.009	0.387	0.009	1.226
P07338	Chymotrypsinogen B OS=Rattus norvegicus OX=10116 GN=Ctrb1 PE=1 SV=1 - [CTRB1_RAT]	27.8	0.579	0.500	0.741	0.402	0.325	1.005	1.215	1.102	0.576	1.067	0.586	0.688	1.075	1.658	0.281	0.369	0.575	0.646	0.410	0.609	0.036	0.0792	0.259	0.476	0.756	0.994	0.605	0.684	0.036	0.052	0.259	0.773
P07687	Epoxide hydrolase 1 OS=Rattus norvegicus OX=10116 GN=Ephx1 PE=1 SV=1 - [HYEP_RAT]	52.5	0.013	0.013	0.013	0.013	0.013	0.641	0.837	0.816	0.436	0.745	0.013	0.013	0.013	0.013	0.013	0.013	0.013	0.013	0.013	0.013	0.009	0.0953	#DIV/0!	#DIV/0!	#DIV/0!	#DIV/0!	0.009	0.231	0.009	0.057	#DIV/0!	#DIV/0!
P07872	Peroxisomal acyl-coenzyme A oxidase 1 OS=Rattus norvegicus GN=Acox1 PE=1 SV=1 - [ACOX1_RAT]	74.6	1.344	0.657	2.259	1.865	1.018	0.639	0.791	0.984	0.144	0.654	1.150	1.578	0.633	0.654	0.576	0.471	1.283	1.256	1.249	0.818	0.036	0.0775	0.127	0.274	0.259	0.630	0.605	0.787	0.127	0.128	0.605	0.729
P09117	Fructose-bisphosphate aldolase C OS=Rattus norvegicus GN=Aldoc PE=1 SV=3 - [ALDOC_RAT]	39.3	0.013	0.013	0.013	0.013	0.013	0.958	1.172	1.220	0.324	1.000	0.436	0.716	0.986	1.222	0.231	0.013	0.013	0.013	0.013	0.013	0.009	0.0296	0.009	0.149	#DIV/0!	#DIV/0!	0.470	0.716	0.009	0.021	0.009	0.191
P09760	Tyrosine-protein kinase Fer OS=Rattus norvegicus GN=Fer PE=1 SV=2 - [FER_RAT]	94.3	0.013	0.013	0.013	0.013	0.013	1.012	0.624	1.047	0.496	0.468	0.013	0.013	0.013	0.013	0.013	0.013	0.013	0.013	0.013	0.013	0.009	0.0709	#DIV/0!	#DIV/0!	#DIV/0!	#DIV/0!	0.009	0.175	0.009	0.046	#DIV/0!	#DIV/0!
P10959	Carboxylesterase 1C OS=Rattus norvegicus GN=Ces1c PE=1 SV=3 - [EST1C_RAT]	60.1	1.022	0.772	0.820	0.334	0.341	0.999	1.503	1.075	1.348	1.595	0.356	0.607	1.090	1.396	0.217	0.309	0.558	0.355	0.279	0.824	0.022	0.0506	0.756	0.669	0.354	0.843	0.127	0.372	0.014	0.021	0.354	0.896
P14272	Plasma kallikrein OS=Rattus norvegicus GN=Klkb1 PE=1 SV=1 - [KLKB1_RAT]	71.2	0.013	0.013	0.013	0.013	0.013	1.219	1.414	1.292	0.613	1.578	0.583	0.963	1.113	1.108	0.246	0.013	0.013	0.013	0.013	0.013	0.009	0.0223	0.009	0.079	#DIV/0!	#DIV/0!	0.056	0.194	0.009	0.016	0.009	0.101
P14562	Lysosome-associated membrane glycoprotein 1 OS=Rattus norvegicus GN=Lamp1 PE=1 SV=1 - [LAMP1_RAT]	43.9	0.013	0.013	0.013	0.013	0.013	0.629	0.918	0.874	0.351	0.759	0.816	0.960	0.817	1.294	0.498	0.490	0.625	0.871	0.543	1.040	0.009	0.0813	0.009	0.049	0.009	0.111	0.354	0.667	0.756	0.366	0.470	0.807
P15429	Beta-enolase OS=Rattus norvegicus GN=Eno3 PE=1 SV=3 - [ENOB_RAT]	47.0	0.013	0.013	0.013	0.013	0.013	1.071	3.932	0.642	0.326	0.924	0.013	0.013	0.013	0.013	0.013	0.013	0.013	0.013	0.013	0.013	0.009	0.0218	#DIV/0!	#DIV/0!	#DIV/0!	#DIV/0!	0.009	0.037	0.009	0.015	#DIV/0!	#DIV/0!
P15684	Aminopeptidase N OS=Rattus norvegicus OX=10116 GN=Anpep PE=1 SV=2 - [AMPN_RAT]	109.4	0.013	0.013	0.013	0.013	0.013	0.663	1.151	1.180	0.436	1.198	0.013	0.013	0.013	0.013	0.013	0.013	0.013	0.013	0.013	0.013	0.009	0.0309	#DIV/0!	#DIV/0!	#DIV/0!	#DIV/0!	0.009	0.058	0.009	0.022	#DIV/0!	#DIV/0!
P15800	Laminin subunit beta-2 OS=Rattus norvegicus GN=Lamb2 PE=2 SV=1 - [LAMB2_RAT]	196.3	1.161	0.740	1.715	0.778	0.688	0.013	0.013	0.013	0.013	0.013	0.013	0.013	0.013	0.013	0.013	0.013	0.013	0.013	0.013	0.013	0.009	0.0252	0.009	0.030	0.009	0.048	#DIV/0!	#DIV/0!	#DIV/0!	#DIV/0!	#DIV/0!	#DIV/0!
P17702	60S ribosomal protein L28 OS=Rattus norvegicus GN=Rpl28 PE=1 SV=4 - [RL28_RAT]	15.8	0.013	0.013	0.013	0.013	0.013	0.692	0.975	0.968	0.260	0.935	0.013	0.013	0.013	0.013	0.013	0.013	0.013	0.013	0.013	0.013	0.009	0.0559	#DIV/0!	#DIV/0!	#DIV/0!	#DIV/0!	0.009	0.130	0.009	0.036	#DIV/0!	#DIV/0!
P18163	Long-chain-fatty-acid--CoA ligase 1 OS=Rattus norvegicus OX=10116 GN=Acsl1 PE=1 SV=1 - [ACSL1_RAT]	78.1	0.013	0.013	0.013	0.013	0.013	0.570	1.230	1.050	0.242	1.095	0.805	0.940	1.002	1.396	0.367	0.596	0.761	1.048	0.626	0.943	0.009	0.0404	0.009	0.043	0.009	0.079	0.917	0.750	0.605	0.314	0.605	0.769
P20767	Ig lambda-2 chain C region OS=Rattus norvegicus PE=4 SV=1 - [LAC2_RAT]	11.3	0.983	0.590	0.840	0.361	0.392	1.055	1.273	1.490	1.147	1.246	0.260	0.367	0.809	1.022	0.111	0.285	0.702	0.531	0.467	0.645	0.014	0.0319	0.470	0.586	0.605	0.946	0.014	0.054	0.014	0.022	0.756	0.886
P21139	Alpha-mannosidase 2C1 OS=Rattus norvegicus GN=Man2c1 PE=1 SV=1 - [MA2C1_RAT]	115.9	0.013	0.013	0.013	0.013	0.013	0.830	1.477	1.202	0.194	1.849	0.013	0.013	0.013	0.013	0.013	0.013	0.013	0.013	0.013	0.013	0.009	0.0230	#DIV/0!	#DIV/0!	#DIV/0!	#DIV/0!	0.009	0.041	0.009	0.016	#DIV/0!	#DIV/0!
P22062	Protein-L-isoaspartate(D-aspartate) O-methyltransferase OS=Rattus norvegicus GN=Pcmt1 PE=1 SV=2 - [PIMT_RAT]	24.6	0.013	0.013	0.013	0.013	0.013	1.046	1.443	1.474	0.169	1.014	0.823	0.890	0.833	1.310	0.626	0.585	0.845	1.225	0.844	0.844	0.009	0.0249	0.009	0.045	0.009	0.063	0.259	0.524	0.259	0.184	0.917	0.769
P23680	Serum amyloid P-component OS=Rattus norvegicus GN=Apcs PE=2 SV=2 - [SAMP_RAT]	26.2	0.013	0.013	0.013	0.013	0.013	0.810	1.135	0.796	0.959	1.277	0.013	0.013	0.013	0.013	0.013	0.013	0.013	0.013	0.013	0.013	0.009	0.0259	#DIV/0!	#DIV/0!	#DIV/0!	#DIV/0!	0.009	0.048	0.009	0.018	#DIV/0!	#DIV/0!
P24049	60S ribosomal protein L17 OS=Rattus norvegicus OX=10116 GN=Rpl17 PE=2 SV=3 - [RL17_RAT]	21.4	0.013	0.013	0.013	0.013	0.013	0.741	0.735	1.051	0.147	0.803	0.013	0.013	0.013	0.013	0.013	0.013	0.013	0.013	0.013	0.013	0.009	0.0938	#DIV/0!	#DIV/0!	#DIV/0!	#DIV/0!	0.009	0.225	0.009	0.056	#DIV/0!	#DIV/0!
P24051	40S ribosomal protein S27-like OS=Rattus norvegicus GN=Rps27l PE=1 SV=3 - [RS27L_RAT]	9.5	0.013	0.013	0.013	0.013	0.013	0.770	0.965	1.446	0.268	1.021	0.936	1.139	1.117	1.019	0.427	0.592	0.713	1.228	0.744	1.097	0.009	0.0333	0.009	0.039	0.009	0.062	0.756	0.705	0.756	0.333	0.756	0.742
P25304	Agrin OS=Rattus norvegicus GN=Agrn PE=1 SV=2 - [AGRIN_RAT]	208.5	1.342	0.668	2.019	1.230	0.720	0.013	0.013	0.013	0.013	0.013	0.013	0.013	0.013	0.013	0.013	0.013	0.013	0.013	0.013	0.013	0.009	0.0223	0.009	0.025	0.009	0.043	#DIV/0!	#DIV/0!	#DIV/0!	#DIV/0!	#DIV/0!	#DIV/0!
P25886	60S ribosomal protein L29 OS=Rattus norvegicus OX=10116 GN=Rpl29 PE=1 SV=3 - [RL29_RAT]	17.3	0.013	0.013	0.013	0.013	0.013	0.772	0.722	1.192	0.285	0.653	1.127	0.942	0.840	1.470	0.517	0.889	0.857	1.419	0.757	1.217	0.009	0.0717	0.009	0.033	0.009	0.048	0.259	0.573	0.085	0.101	0.917	0.748
P26644	Beta-2-glycoprotein 1 OS=Rattus norvegicus GN=Apoh PE=2 SV=2 - [APOH_RAT]	33.2	0.013	0.013	0.013	0.013	0.013	0.834	1.209	1.002	0.306	1.185	0.768	1.000	0.858	1.276	0.500	0.641	0.885	1.059	0.541	1.198	0.009	0.0321	0.009	0.048	0.009	0.063	0.756	0.714	0.756	0.333	0.917	0.774
P28023	Dynactin subunit 1 OS=Rattus norvegicus GN=Dctn1 PE=1 SV=2 - [DCTN1_RAT]	141.8	0.013	0.013	0.013	0.013	0.013	0.728	1.106	1.074	0.259	1.001	0.643	0.841	0.918	1.415	0.262	0.552	0.660	0.817	0.514	0.971	0.009	0.0418	0.009	0.070	0.009	0.115	0.756	0.780	0.259	0.205	0.605	0.837
P32362	Uroporphyrinogen decarboxylase (Fragment) OS=Rattus norvegicus GN=Urod PE=1 SV=1 - [DCUP_RAT]	40.4	0.013	0.013	0.013	0.013	0.013	0.951	1.540	1.251	0.259	1.060	0.628	0.729	0.978	1.261	0.268	0.013	0.013	0.013	0.013	0.013	0.009	0.0253	0.009	0.1004	#DIV/0!	#DIV/0!	0.470	0.667	0.009	0.018	0.009	0.124
P38656	Lupus La protein homolog OS=Rattus norvegicus GN=Ssb PE=1 SV=1 - [LA_RAT]	47.7	0.013	0.013	0.013	0.013	0.013	0.679	1.101	1.157	0.211	1.099	0.479	0.628	0.965	1.574	0.181	0.510	0.493	0.612	0.340	0.946	0.009	0.0376	0.009	0.111	0.009	0.282	0.470	0.727	0.184	0.172	0.605	0.874
P47245	Nardilysin OS=Rattus norvegicus GN=Nrd1 PE=1 SV=1 - [NRDC_RAT]	132.9	1.177	0.818	1.264	0.438	0.511	0.013	0.013	0.013	0.013	0.013	0.356	0.733	1.126	1.295	0.153	0.382	0.733	0.781	0.621	0.957	0.009	0.0393	0.605	0.611	0.470	0.837	0.009	0.161	0.009	0.057	0.917	0.839
P48199	C-reactive protein OS=Rattus norvegicus GN=Crp PE=1 SV=1 - [CRP_RAT]	25.5	0.594	0.406	1.038	0.205	0.211	0.942	1.486	0.951	1.134	1.823	0.368	0.487	0.757	0.883	0.228	0.174	0.457	0.427	0.276	0.409	0.036	0.0780	0.605	0.652	0.756	1.003	0.014	0.053	0.014	0.022	0.259	0.787
P49242	40S ribosomal protein S3a OS=Rattus norvegicus GN=Rps3a PE=1 SV=2 - [RS3A_RAT]	29.9	0.013	0.013	0.013	0.013	0.013	0.664	0.839	1.217	0.177	0.896	0.841	1.221	1.139	0.945	0.457	0.629	0.578	1.136	0.584	1.098	0.009	0.0586	0.009	0.039	0.009	0.074	0.354	0.643	0.756	0.353	0.470	0.745
P50297	Arylamine N-acetyltransferase 1 OS=Rattus norvegicus OX=10116 GN=Nat1 PE=2 SV=1 - [ARY1_RAT]	33.4	1.234	0.907	1.258	0.434	0.425	0.013	0.013	0.013	0.013	0.013	0.438	0.726	0.979	1.288	0.248	0.513	1.309	0.720	0.645	1.458	0.009	0.0374	0.917	0.665	0.470	0.776	0.009	0.158	0.009	0.021	0.470	0.763
P52303	AP-1 complex subunit beta-1 OS=Rattus norvegicus GN=Ap1b1 PE=1 SV=1 - [AP1B1_RAT]	104.5	0.013	0.013	0.013	0.013	0.013	0.874	1.535	1.494	0.358	0.989	0.013	1.138	0.889	1.485	0.013	0.119	0.313	0.269	0.162	0.565	0.009	0.0240	0.063	0.151	0.009	1.751	0.353	0.703	0.022	0.034	0.604	0.906
P53534	Glycogen phosphorylase, brain form (Fragment) OS=Rattus norvegicus GN=Pygb PE=1 SV=3 - [PYGB_RAT]	96.1	0.013	0.013	0.013	0.013	0.013	0.599	1.263	1.223	0.192	1.287	0.801	0.942	0.931	1.388	0.416	0.561	0.639	0.952	0.528	1.092	0.009	0.0318	0.009	0.045	0.009	0.091	0.917	0.733	0.354	0.230	0.756	0.792
P53812	Phosphatidylinositol transfer protein beta isoform OS=Rattus norvegicus GN=Pitpnb PE=1 SV=2 - [PIPNB_RAT]	31.4	0.013	0.013	0.013	0.013	0.013	0.710	0.823	1.093	0.332	0.796	0.013	0.013	0.013	0.013	0.013	0.013	0.013	0.013	0.013	0.013	0.009	0.0610	#DIV/0!	#DIV/0!	#DIV/0!	#DIV/0!	0.009	0.145	0.009	0.040	#DIV/0!	#DIV/0!
P55068	Brevican core protein OS=Rattus norvegicus GN=Bcan PE=1 SV=2 - [PGCB_RAT]	96.0	1.309	1.522	2.212	2.014	0.907	0.013	0.013	0.013	0.013	0.013	1.603	1.769	0.786	0.829	0.902	0.459	1.380	1.209	1.357	1.049	0.009	0.0214	0.184	0.347	0.184	0.512	0.009	0.039	0.009	0.017	0.917	0.739
P55159	Serum paraoxonase/arylesterase 1 OS=Rattus norvegicus GN=Pon1 PE=1 SV=3 - [PON1_RAT]	39.3	0.013	0.013	0.013	0.013	0.013	0.873	1.193	0.756	0.066	1.647	0.013	0.013	0.013	0.013	0.013	0.013	0.013	0.013	0.013	0.013	0.009	0.0323	#DIV/0!	#DIV/0!	#DIV/0!	#DIV/0!	0.009	0.062	0.009	0.022	#DIV/0!	#DIV/0!
P57113	Maleylacetoacetate isomerase OS=Rattus norvegicus GN=Gstz1 PE=1 SV=2 - [MAAI_RAT]	23.9	0.013	0.013	0.013	0.013	0.013	0.653	1.189	0.962	0.193	1.296	0.402	0.664	0.733	1.480	0.165	0.241	0.353	0.386	0.197	0.574	0.009	0.0359	0.009	0.204	0.009	0.973	0.605	0.791	0.127	0.144	0.184	0.601
P60892	Ribose-phosphate pyrophosphokinase 1 OS=Rattus norvegicus GN=Prps1 PE=1 SV=2 - [PRPS1_RAT]	34.8	0.013	0.013	0.013	0.013	0.013	0.631	1.414	1.018	0.214	1.378	0.562	0.809	1.305	1.296	0.297	0.461	0.455	0.547	0.332	1.042	0.009	0.0300	0.009	0.057	0.009	0.292	0.605	0.701	0.259	0.208	0.259	0.761
P61314	60S ribosomal protein L15 OS=Rattus norvegicus GN=Rpl15 PE=1 SV=2 - [RL15_RAT]	24.1	0.013	0.013	0.013	0.013	0.013	0.804	1.102	1.186	0.225	1.268	0.013	0.013	0.013	0.013	0.013	0.013	0.013	0.013	0.013	0.013	0.009	0.0316	#DIV/0!	#DIV/0!	#DIV/0!	#DIV/0!	0.009	0.060	0.009	0.022	#DIV/0!	#DIV/0!
P61459	Pterin-4-alpha-carbinolamine dehydratase OS=Rattus norvegicus OX=10116 GN=Pcbd1 PE=1 SV=2 - [PHS_RAT]	12.0	0.013	0.013	0.013	0.013	0.013	0.901	0.762	0.800	0.467	0.908	0.561	0.742	0.551	1.107	0.394	0.013	0.013	0.013	0.013	0.013	0.009	0.0549	0.009	0.246	#DIV/0!	#DIV/0!	0.354	0.680	0.009	0.035	0.009	0.286
P62161	Calmodulin OS=Rattus norvegicus GN=Calm1 PE=1 SV=2 - [CALM_RAT]	16.8	0.013	0.013	0.013	0.013	0.013	1.476	1.676	1.764	0.077	1.143	0.223	0.589	1.465	2.235	0.028	0.353	0.592	0.291	0.156	0.937	0.009	0.0222	0.009	0.041	0.009	0.674	0.470	0.636	0.127	0.126	0.756	0.862
P62198	26S protease regulatory subunit 8 OS=Rattus norvegicus GN=Psmc5 PE=1 SV=1 - [PRS8_RAT]	45.6	0.013	0.013	0.013	0.013	0.013	0.803	1.189	1.217	0.487	0.898	0.013	0.013	0.013	0.013	0.013	0.517	0.638	1.155	0.699	0.990	0.009	0.0314	#DIV/0!	#DIV/0!	0.009	0.076	0.009	0.059	0.470	0.264	0.009	0.103
P62268	40S ribosomal protein S23 OS=Rattus norvegicus GN=Rps23 PE=1 SV=3 - [RS23_RAT]	15.8	0.013	0.013	0.013	0.013	0.013	0.732	0.964	1.147	0.109	1.036	0.967	1.209	1.067	1.031	0.664	0.765	0.859	1.343	0.873	0.995	0.009	0.0496	0.009	0.032	0.009	0.052	0.470	0.666	0.756	0.334	0.605	0.727
P62332	ADP-ribosylation factor 6 OS=Rattus norvegicus GN=Arf6 PE=1 SV=2 - [ARF6_RAT]	20.1	0.013	0.013	0.013	0.013	0.013	0.681	1.150	1.117	0.253	1.088	0.732	0.942	0.801	2.061	0.312	0.536	0.406	0.761	0.397	0.946	0.009	0.0361	0.009	0.034	0.009	0.208	0.917	0.729	0.259	0.209	0.470	0.811
P62747	Rho-related GTP-binding protein RhoB OS=Rattus norvegicus GN=Rhob PE=1 SV=1 - [RHOB_RAT]	22.1	0.013	0.013	0.013	0.013	0.013	0.763	1.898	1.362	0.236	1.515	0.013	0.359	1.058	1.960	0.013	0.213	0.190	0.184	0.093	0.805	0.009	0.0227	0.063	0.152	0.009	1.459	0.353	0.701	0.036	0.052	0.755	0.897
P62864	40S ribosomal protein S30 OS=Rattus norvegicus OX=10116 GN=Fau PE=1 SV=1 - [RS30_RAT]	6.6	0.013	0.013	0.013	0.013	0.013	1.232	0.830	1.338	0.160	0.768	0.824	1.080	1.794	1.064	0.466	0.599	1.023	1.576	0.719	1.233	0.009	0.0350	0.009	0.028	0.009	0.047	0.756	0.694	0.756	0.329	0.917	0.743
P62902	60S ribosomal protein L31 OS=Rattus norvegicus OX=10116 GN=Rpl31 PE=2 SV=1 - [RL31_RAT]	14.5	0.013	0.013	0.013	0.013	0.013	0.774	0.987	1.233	0.212	0.949	0.257	0.443	1.089	1.535	0.228	0.013	0.013	0.013	0.013	0.013	0.009	0.0420	0.009	0.166	#DIV/0!	#DIV/0!	0.917	0.815	0.009	0.027	0.009	0.215
P62912	60S ribosomal protein L32 OS=Rattus norvegicus OX=10116 GN=Rpl32 PE=1 SV=2 - [RL32_RAT]	15.8	0.689	0.744	1.270	0.426	0.551	0.013	0.013	0.013	0.013	0.013	0.579	0.997	1.083	0.876	0.287	0.622	0.526	0.873	0.491	0.774	0.009	0.0685	0.756	0.653	0.917	0.959	0.009	0.134	0.009	0.076	0.354	0.859
P63095	Guanine nucleotide-binding protein G(s) subunit alpha isoforms short OS=Rattus norvegicus GN=Gnas PE=1 SV=1 - [GNAS2_RAT]	45.6	0.013	0.013	0.013	0.013	0.013	0.747	0.824	1.133	0.311	0.726	0.013	0.013	0.013	0.013	0.013	0.013	0.013	0.013	0.013	0.013	0.009	0.0629	#DIV/0!	#DIV/0!	#DIV/0!	#DIV/0!	0.009	0.150	0.009	0.041	#DIV/0!	#DIV/0!
P63100	Calcineurin subunit B type 1 OS=Rattus norvegicus GN=Ppp3r1 PE=1 SV=2 - [CANB1_RAT]	19.3	0.013	0.013	0.013	0.013	0.013	0.963	1.297	1.378	0.490	1.252	0.138	0.640	0.986	1.846	0.070	0.013	0.013	0.013	0.013	0.013	0.009	0.0235	0.009	0.140	#DIV/0!	#DIV/0!	0.354	0.607	0.009	0.017	0.009	0.165
P63326	40S ribosomal protein S10 OS=Rattus norvegicus GN=Rps10 PE=2 SV=1 - [RS10_RAT]	18.9	0.013	0.013	0.013	0.013	0.013	1.011	0.875	1.312	0.139	0.922	0.858	0.929	1.531	1.127	0.571	0.684	0.738	1.223	0.580	1.181	0.009	0.0372	0.009	0.031	0.009	0.060	0.756	0.701	0.756	0.336	0.756	0.731
P81718	Tyrosine-protein phosphatase non-receptor type 6 OS=Rattus norvegicus OX=10116 GN=Ptpn6 PE=1 SV=1 - [PTN6_RAT]	69.5	0.013	0.013	0.013	0.013	0.013	0.580	0.994	0.898	0.136	0.904	0.761	1.054	1.513	1.647	0.227	0.546	0.444	1.080	0.427	1.158	0.009	0.0824	0.009	0.029	0.009	0.101	0.259	0.562	0.917	0.395	0.470	0.735
P81799	N-acetyl-D-glucosamine kinase OS=Rattus norvegicus GN=Nagk PE=1 SV=4 - [NAGK_RAT]	37.2	0.013	0.013	0.013	0.013	0.013	0.941	1.345	1.376	0.253	0.968	0.358	0.603	0.819	1.306	0.135	0.013	0.013	0.013	0.013	0.013	0.009	0.0267	0.009	0.340	#DIV/0!	#DIV/0!	0.184	0.499	0.009	0.019	0.009	0.330
P82450	Sialate O-acetylesterase OS=Rattus norvegicus OX=10116 GN=Siae PE=1 SV=2 - [SIAE_RAT]	60.4	0.943	1.098	1.091	1.077	0.842	0.734	0.697	0.886	0.452	0.640	0.013	0.013	0.013	0.013	0.013	0.013	0.013	0.013	0.013	0.013	0.022	0.0511	0.009	0.031	0.009	0.048	0.009	0.267	0.009	0.065	#DIV/0!	#DIV/0!
P83953	Importin subunit alpha-5 OS=Rattus norvegicus GN=Kpna1 PE=1 SV=1 - [IMA5_RAT]	60.1	0.871	0.805	1.516	0.541	0.492	0.013	0.013	0.013	0.013	0.013	0.013	0.013	0.013	0.013	0.013	0.013	0.013	0.013	0.013	0.013	0.009	0.0383	0.009	0.060	0.009	0.067	#DIV/0!	#DIV/0!	#DIV/0!	#DIV/0!	#DIV/0!	#DIV/0!
P84850	D-2-hydroxyglutarate dehydrogenase, mitochondrial OS=Rattus norvegicus OX=10116 GN=D2hgdh PE=3 SV=1 - [D2HDH_RAT]	58.8	0.013	0.013	0.013	0.013	0.013	0.648	1.074	1.198	0.344	0.840	0.400	0.538	0.917	1.506	0.195	0.437	0.675	0.616	0.409	0.788	0.009	0.0439	0.009	0.162	0.009	0.250	0.605	0.795	0.259	0.211	0.917	0.852
P97526	Neurofibromin OS=Rattus norvegicus OX=10116 GN=Nf1 PE=1 SV=1 - [NF1_RAT]	316.9	1.389	0.936	1.600	0.947	0.558	0.013	0.013	0.013	0.013	0.013	0.013	0.013	0.013	0.013	0.013	0.367	0.914	0.831	0.703	0.890	0.009	0.0234	0.009	0.027	0.085	0.326	#DIV/0!	#DIV/0!	0.009	0.043	0.009	0.156
P97571	Calpain-1 catalytic subunit OS=Rattus norvegicus GN=Capn1 PE=1 SV=1 - [CAN1_RAT]	82.1	0.857	0.795	1.834	0.995	0.751	0.013	0.013	0.013	0.013	0.013	0.722	1.036	0.929	1.242	0.603	0.622	0.733	1.165	0.741	1.025	0.009	0.0243	0.756	0.586	0.354	0.716	0.009	0.062	0.009	0.025	0.917	0.770
Q00715	Histone H2B type 1 OS=Rattus norvegicus PE=1 SV=2 - [H2B1_RAT]	14.0	0.013	0.013	0.013	0.013	0.013	0.748	0.893	1.024	0.319	0.914	0.583	0.773	0.963	1.528	0.266	0.349	0.915	0.944	0.782	1.033	0.009	0.0526	0.009	0.069	0.009	0.074	0.917	0.799	0.470	0.275	0.756	0.798
Q00729	Histone H2B type 1-A OS=Rattus norvegicus GN=Hist1h2ba PE=1 SV=2 - [H2B1A_RAT]	14.2	0.013	0.013	0.013	0.013	0.013	0.662	0.752	1.045	0.160	1.097	1.015	0.902	1.089	2.584	0.709	0.986	0.697	1.303	0.634	1.048	0.009	0.0656	0.009	0.024	0.009	0.053	0.354	0.589	0.605	0.309	0.470	0.707
Q01986	Dual specificity mitogen-activated protein kinase kinase 1 OS=Rattus norvegicus GN=Map2k1 PE=1 SV=2 - [MP2K1_RAT]	43.4	0.013	0.013	0.013	0.013	0.013	0.675	1.281	0.950	0.330	1.404	0.575	1.020	0.848	1.435	0.276	0.160	0.273	0.296	0.487	0.492	0.009	0.0308	0.009	0.065	0.009	1.095	0.756	0.725	0.036	0.053	0.056	0.203
Q02356	AMP deaminase 2 OS=Rattus norvegicus GN=Ampd2 PE=2 SV=2 - [AMPD2_RAT]	94.7	0.013	0.013	0.013	0.013	0.013	0.744	0.896	1.021	0.299	0.786	0.013	0.013	0.013	0.013	0.013	0.013	0.013	0.013	0.013	0.013	0.009	0.0622	#DIV/0!	#DIV/0!	#DIV/0!	#DIV/0!	0.009	0.147	0.009	0.040	#DIV/0!	#DIV/0!
Q02589	[Protein ADP-ribosylarginine] hydrolase OS=Rattus norvegicus GN=Adprh PE=1 SV=2 - [ADPRH_RAT]	39.9	0.891	0.823	1.503	0.362	0.602	0.013	0.013	0.013	0.013	0.013	0.013	0.013	0.013	0.013	0.013	0.013	0.013	0.013	0.013	0.013	0.009	0.0406	0.009	0.063	0.009	0.068	#DIV/0!	#DIV/0!	#DIV/0!	#DIV/0!	#DIV/0!	#DIV/0!
Q2A121	Alpha-ketoglutarate-dependent dioxygenase FTO OS=Rattus norvegicus GN=Fto PE=2 SV=1 - [FTO_RAT]	57.9	0.013	0.013	0.013	0.013	0.013	0.955	1.172	0.997	0.196	0.997	0.629	0.920	1.067	1.284	0.346	0.449	0.892	0.877	0.651	1.005	0.009	0.0352	0.009	0.059	0.009	0.085	0.917	0.761	0.354	0.232	0.605	0.794
Q2LAP6	Testin OS=Rattus norvegicus GN=Tes PE=1 SV=1 - [TES_RAT]	47.6	0.013	0.013	0.013	0.013	0.013	0.487	1.296	0.917	0.098	1.260	1.225	1.066	1.398	2.496	0.409	0.749	0.279	1.225	0.320	1.039	0.009	0.0465	0.009	0.024	0.009	0.107	0.354	0.587	0.605	0.322	0.127	0.425
Q2LC84	Protein numb homolog OS=Rattus norvegicus GN=Numb PE=1 SV=2 - [NUMB_RAT]	70.6	1.755	1.613	1.723	3.079	1.149	0.498	0.493	0.618	0.302	0.279	1.766	1.571	0.594	0.611	1.427	0.704	2.009	1.749	2.459	1.262	0.014	0.0317	0.184	0.346	0.917	0.836	0.036	0.133	0.014	0.021	0.259	0.680
Q3MID3	ADP-ribosylation factor GTPase-activating protein 2 OS=Rattus norvegicus GN=Arfgap2 PE=1 SV=1 - [ARFG2_RAT]	56.5	0.743	0.728	1.865	0.632	0.677	0.013	0.013	0.013	0.013	0.013	0.816	1.079	0.732	1.157	0.505	0.745	0.761	1.277	0.692	1.056	0.009	0.0305	0.605	0.577	0.259	0.642	0.009	0.076	0.009	0.022	0.917	0.770
Q498R7	UPF0587 protein C1orf123 homolog OS=Rattus norvegicus PE=2 SV=1 - [CA123_RAT]	18.1	0.013	0.013	0.013	0.013	0.013	0.823	1.302	1.263	0.383	1.171	0.448	0.586	0.982	1.410	0.269	0.592	0.964	1.205	0.804	0.975	0.009	0.0261	0.009	0.137	0.009	0.056	0.470	0.685	0.470	0.260	0.470	0.774
Q4AEF8	Coatomer subunit gamma-1 OS=Rattus norvegicus GN=Copg1 PE=2 SV=1 - [COPG1_RAT]	97.6	0.013	0.013	0.013	0.013	0.013	0.698	1.036	1.110	0.356	1.085	0.667	0.988	1.078	1.243	0.346	0.425	0.532	0.802	0.485	1.093	0.009	0.0363	0.009	0.054	0.009	0.137	0.756	0.742	0.470	0.279	0.470	0.864
Q4FZU6	Annexin A8 OS=Rattus norvegicus GN=Anxa8 PE=1 SV=1 - [ANXA8_RAT]	36.7	1.061	1.084	1.606	0.926	0.756	0.013	0.013	0.013	0.013	0.013	0.013	0.013	0.013	0.013	0.013	0.013	0.013	0.013	0.013	0.013	0.009	0.0232	0.009	0.027	0.009	0.045	#DIV/0!	#DIV/0!	#DIV/0!	#DIV/0!	#DIV/0!	#DIV/0!
Q4KLF8	Actin-related protein 2/3 complex subunit 5 OS=Rattus norvegicus GN=Arpc5 PE=1 SV=3 - [ARPC5_RAT]	16.3	0.013	0.013	0.013	0.013	0.013	0.809	0.901	1.180	0.132	0.812	1.007	1.043	1.019	1.453	0.614	0.787	0.886	1.386	0.923	0.946	0.009	0.0554	0.009	0.029	0.009	0.049	0.259	0.544	0.354	0.224	0.354	0.757

Q4KLL0	Transcription elongation factor A protein 1 OS=Rattus norvegicus GN=Tcea1 PE=1 SV=1 - [TCEA1_RAT]	33.9	0.013	0.013	0.013	0.013	0.013	0.825	1.131	1.222	0.095	0.797	0.474	0.992	1.043	2.035	0.444	0.671	0.739	1.138	0.606	0.923	0.009	0.0452	0.009	0.032	0.009	0.072	0.917	0.732	0.605	0.315	0.917	0.764
Q4KM35	Proteasome subunit beta type-10 OS=Rattus norvegicus GN=Psmb10 PE=2 SV=1 - [PSB10_RAT]	29.0	0.013	0.013	0.013	0.013	0.013	0.895	1.447	1.254	0.690	0.980	0.013	0.013	0.013	0.013	0.013	0.013	0.013	0.013	0.013	0.013	0.009	0.0239	#DIV/0!	#DIV/0!	#DIV/0!	#DIV/0!	0.009	0.043	0.009	0.017	#DIV/0!	#DIV/0!
Q4KM65	Cleavage and polyadenylation specificity factor subunit 5 OS=Rattus norvegicus GN=Nudt21 PE=2 SV=1 - [CPSF5_RAT]	26.2	0.013	0.013	0.013	0.013	0.013	0.732	0.743	1.017	0.439	0.648	0.013	0.013	0.013	0.013	0.013	0.013	0.013	0.013	0.013	0.013	0.009	0.0753	#DIV/0!	#DIV/0!	#DIV/0!	#DIV/0!	0.009	0.190	0.009	0.048	#DIV/0!	#DIV/0!
Q4V8F9	Hydroxysteroid dehydrogenase-like protein 2 OS=Rattus norvegicus OX=10116 GN=Hsdl2 PE=2 SV=1 - [HSDL2_RAT]	58.3	0.013	0.013	0.013	0.013	0.013	0.602	1.237	1.091	0.406	0.904	0.339	0.600	0.609	1.205	0.119	0.013	0.013	0.013	0.013	0.013	0.009	0.0379	0.009	0.419	#DIV/0!	#DIV/0!	0.259	0.625	0.009	0.026	0.009	0.429
Q561R9	Beta-lactamase-like protein 2 OS=Rattus norvegicus GN=Lactb2 PE=2 SV=1 - [LACB2_RAT]	32.5	0.013	0.013	0.013	0.013	0.013	0.717	1.025	1.091	0.387	0.973	0.631	0.728	0.782	1.137	0.356	0.452	0.816	0.917	0.530	0.894	0.009	0.0398	0.009	0.146	0.009	0.108	0.605	0.785	0.354	0.239	0.756	0.848
Q562C9	1,2-dihydroxy-3-keto-5-methylthiopentene dioxygenase OS=Rattus norvegicus OX=10116 GN=Adi1 PE=2 SV=1 - [MTND_RAT]	21.4	0.013	0.013	0.013	0.013	0.013	0.932	1.084	1.328	0.254	0.858	0.532	0.809	1.092	1.539	0.212	0.731	0.820	1.253	0.761	1.025	0.009	0.0337	0.009	0.063	0.009	0.055	0.756	0.740	0.605	0.300	0.756	0.752
Q5FVI6	V-type proton ATPase subunit C 1 OS=Rattus norvegicus GN=Atp6v1c1 PE=2 SV=1 - [VATC1_RAT]	43.9	0.013	0.013	0.013	0.013	0.013	0.573	0.853	1.181	0.067	0.824	0.013	0.013	0.013	0.013	0.013	0.013	0.013	0.013	0.013	0.013	0.009	0.0897	#DIV/0!	#DIV/0!	#DIV/0!	#DIV/0!	0.009	0.209	0.009	0.055	#DIV/0!	#DIV/0!
Q5I0K3	Citramalyl-CoA lyase, mitochondrial OS=Rattus norvegicus OX=10116 GN=Clybl PE=2 SV=2 - [CLYBL_RAT]	37.3	0.013	0.013	0.013	0.013	0.013	0.776	0.999	0.987	0.316	0.731	0.452	0.509	0.672	0.752	0.372	0.013	0.013	0.013	0.013	0.013	0.009	0.0575	0.009	0.446	#DIV/0!	#DIV/0!	0.184	0.521	0.009	0.037	0.009	0.572
Q5M7T9	Threonine synthase-like 2 OS=Rattus norvegicus OX=10116 GN=Thnsl2 PE=2 SV=1 - [THNS2_RAT]	54.1	0.013	0.013	0.013	0.013	0.013	0.630	0.891	0.877	0.259	0.815	0.584	0.793	0.895	0.970	0.461	0.013	0.013	0.013	0.013	0.013	0.009	0.0968	0.009	0.135	#DIV/0!	#DIV/0!	0.756	0.824	0.009	0.058	0.009	0.159
Q5PQR8	EKC/KEOPS complex subunit Tprkb OS=Rattus norvegicus OX=10116 GN=Tprkb PE=2 SV=1 - [TPRKB_RAT]	17.6	0.013	0.013	0.013	0.013	0.013	0.936	1.269	1.608	0.226	1.301	0.013	0.013	0.013	0.013	0.013	0.013	0.013	0.013	0.013	0.013	0.009	0.0237	#DIV/0!	#DIV/0!	#DIV/0!	#DIV/0!	0.009	0.042	0.009	0.017	#DIV/0!	#DIV/0!
Q5QE80	Aldehyde oxidase 3 OS=Rattus norvegicus GN=Aox3 PE=1 SV=1 - [AOXC_RAT]	146.7	1.486	0.879	1.790	0.721	0.339	0.013	0.013	0.013	0.013	0.013	0.013	0.013	0.013	0.013	0.013	0.013	0.013	0.013	0.013	0.013	0.009	0.0245	0.009	0.028	0.009	0.047	#DIV/0!	#DIV/0!	#DIV/0!	#DIV/0!	#DIV/0!	#DIV/0!
Q5RJP0	Aldose reductase-related protein 1 OS=Rattus norvegicus GN=Akr1b7 PE=1 SV=1 - [ALD1_RAT]	36.1	0.013	0.013	0.013	0.013	0.013	0.517	0.723	0.795	0.362	1.085	0.013	0.013	0.013	0.013	0.013	0.013	0.013	0.013	0.013	0.013	0.009	0.0924	#DIV/0!	#DIV/0!	#DIV/0!	#DIV/0!	0.009	0.219	0.009	0.055	#DIV/0!	#DIV/0!
Q5RJR2	Twinfilin-1 OS=Rattus norvegicus GN=Twf1 PE=2 SV=1 - [TWF1_RAT]	40.1	0.013	0.013	0.013	0.013	0.013	0.790	0.984	1.212	0.137	0.849	0.509	1.012	1.022	1.340	0.280	0.550	0.664	1.018	0.640	0.995	0.009	0.0508	0.009	0.065	0.009	0.086	0.605	0.760	0.756	0.353	0.756	0.808
Q5RK09	Eukaryotic translation initiation factor 3 subunit G OS=Rattus norvegicus GN=Eif3g PE=1 SV=1 - [EIF3G_RAT]	35.6	0.013	0.013	0.013	0.013	0.013	0.696	1.057	1.308	0.192	0.865	0.735	0.910	0.937	1.707	0.237	0.769	0.424	1.065	0.360	1.372	0.009	0.0432	0.009	0.041	0.009	0.077	0.756	0.739	0.917	0.382	0.917	0.779
Q5U1Y4	1,5-anhydro-D-fructose reductase OS=Rattus norvegicus GN=Akr1e2 PE=2 SV=1 - [AKCL2_RAT]	34.5	0.013	0.013	0.013	0.013	0.013	1.027	1.195	1.136	1.103	1.146	0.013	0.013	0.013	0.013	0.013	0.013	0.013	0.013	0.013	0.013	0.009	0.0229	#DIV/0!	#DIV/0!	#DIV/0!	#DIV/0!	0.009	0.040	0.009	0.016	#DIV/0!	#DIV/0!
Q5U209	Ribonuclease H2 subunit A OS=Rattus norvegicus OX=10116 GN=Rnaseh2a PE=2 SV=1 - [RNH2A_RAT]	33.3	1.253	0.748	1.614	0.853	0.692	0.013	0.013	0.013	0.013	0.013	0.013	0.013	0.013	0.013	0.013	0.013	0.013	0.013	0.013	0.013	0.009	0.0247	0.009	0.029	0.009	0.047	#DIV/0!	#DIV/0!	#DIV/0!	#DIV/0!	#DIV/0!	#DIV/0!
Q5U2Z3	Nucleosome assembly protein 1-like 4 OS=Rattus norvegicus GN=Nap1l4 PE=1 SV=1 - [NP1L4_RAT]	43.9	0.013	0.013	0.013	0.013	0.013	0.852	1.202	0.963	0.447	0.765	0.554	0.728	0.779	1.564	0.291	0.013	0.013	0.013	0.013	0.013	0.009	0.0381	0.009	0.093	#DIV/0!	#DIV/0!	0.470	0.724	0.009	0.026	0.009	0.113
Q5XHZ0	Heat shock protein 75 kDa, mitochondrial OS=Rattus norvegicus GN=Trap1 PE=1 SV=1 - [TRAP1_RAT]	80.4	0.013	0.013	0.013	0.013	0.013	0.692	1.475	0.836	0.256	1.978	0.870	0.969	0.875	1.325	0.482	0.314	0.630	0.747	0.546	0.798	0.009	0.0241	0.009	0.042	0.009	0.225	0.917	0.720	0.259	0.195	0.085	0.301
Q5XI22	Acetyl-CoA acetyltransferase, cytosolic OS=Rattus norvegicus GN=Acat2 PE=1 SV=1 - [THIC_RAT]	41.1	0.013	0.013	0.013	0.013	0.013	0.836	1.387	1.212	0.377	1.124	0.013	0.013	0.013	0.013	0.013	0.013	0.013	0.013	0.013	0.013	0.009	0.0262	#DIV/0!	#DIV/0!	#DIV/0!	#DIV/0!	0.009	0.048	0.009	0.018	#DIV/0!	#DIV/0!
Q5XIG8	Serine-threonine kinase receptor-associated protein OS=Rattus norvegicus GN=Strap PE=1 SV=1 - [STRAP_RAT]	38.4	0.901	0.853	1.401	0.657	0.545	0.013	0.013	0.013	0.013	0.013	0.766	1.036	1.000	1.168	0.413	0.483	0.774	1.036	0.887	0.909	0.009	0.0348	0.756	0.606	0.917	0.891	0.009	0.071	0.009	0.028	0.756	0.769
Q5XIJ6	BRISC and BRCA1-A complex member 1 OS=Rattus norvegicus GN=Babam1 PE=1 SV=1 - [BABA1_RAT]	36.9	0.013	0.013	0.013	0.013	0.013	0.695	0.887	1.082	0.131	0.789	0.013	0.013	0.013	0.013	0.013	0.013	0.013	0.013	0.013	0.013	0.009	0.0744	#DIV/0!	#DIV/0!	#DIV/0!	#DIV/0!	0.009	0.186	0.009	0.048	#DIV/0!	#DIV/0!
Q60587	Trifunctional enzyme subunit beta, mitochondrial OS=Rattus norvegicus OX=10116 GN=Hadhb PE=1 SV=1 - [ECHB_RAT]	51.4	0.013	0.013	0.013	0.013	0.013	0.503	0.973	0.961	0.183	0.881	0.513	0.856	0.762	1.360	0.281	0.359	0.355	0.456	0.315	0.859	0.009	0.0847	0.009	0.125	0.009	0.625	0.917	0.823	0.184	0.174	0.354	0.889
Q62658	Peptidyl-prolyl cis-trans isomerase FKBP1A OS=Rattus norvegicus GN=Fkbp1a PE=1 SV=3 - [FKB1A_RAT]	11.9	0.013	0.013	0.013	0.013	0.013	0.790	0.785	1.083	0.148	0.639	0.013	0.013	0.013	0.013	0.013	0.013	0.013	0.013	0.013	0.013	0.009	0.0999	#DIV/0!	#DIV/0!	#DIV/0!	#DIV/0!	0.009	0.238	0.009	0.061	#DIV/0!	#DIV/0!
Q62745	CD81 antigen OS=Rattus norvegicus GN=Cd81 PE=1 SV=1 - [CD81_RAT]	25.9	0.013	0.013	0.013	0.013	0.013	0.625	1.004	0.808	0.299	0.963	0.013	0.013	0.013	0.013	0.013	0.013	0.013	0.013	0.013	0.013	0.009	0.0677	#DIV/0!	#DIV/0!	#DIV/0!	#DIV/0!	0.009	0.155	0.009	0.044	#DIV/0!	#DIV/0!
Q62940	E3 ubiquitin-protein ligase NEDD4 OS=Rattus norvegicus GN=Nedd4 PE=1 SV=1 - [NEDD4_RAT]	102.3	0.916	0.736	1.219	0.705	0.502	0.013	0.013	0.013	0.013	0.013	0.742	0.946	1.066	1.276	0.270	0.420	0.822	0.839	0.957	1.006	0.009	0.0448	0.470	0.546	0.756	0.898	0.009	0.074	0.009	0.030	0.756	0.783
Q62975	Protein Z-dependent protease inhibitor OS=Rattus norvegicus GN=Serpina10 PE=2 SV=2 - [ZPI_RAT]	50.2	0.013	0.013	0.013	0.013	0.013	0.971	1.083	1.344	0.707	0.994	0.013	0.013	0.013	0.013	0.013	0.013	0.013	0.013	0.013	0.013	0.009	0.0251	#DIV/0!	#DIV/0!	#DIV/0!	#DIV/0!	0.009	0.046	0.009	0.017	#DIV/0!	#DIV/0!
Q63010	Liver carboxylesterase B-1 OS=Rattus norvegicus PE=1 SV=1 - [EST5_RAT]	62.5	0.226	0.404	0.354	0.236	0.183	0.644	0.820	0.752	0.418	0.701	0.266	0.330	0.418	0.792	0.199	0.322	0.514	0.369	0.697	0.667	0.014	0.0324	0.354	0.563	0.056	0.244	0.127	0.406	0.127	0.144	0.354	0.912
Q63028	Alpha-adducin OS=Rattus norvegicus GN=Add1 PE=2 SV=2 - [ADDA_RAT]	80.3	0.013	0.013	0.013	0.013	0.013	0.846	0.934	1.036	0.396	0.848	0.534	0.761	0.626	1.010	0.514	0.013	0.013	0.013	0.013	0.013	0.009	0.0462	0.009	0.210	#DIV/0!	#DIV/0!	0.354	0.677	0.009	0.029	0.009	0.260
Q63065	[Pyruvate dehydrogenase (acetyl-transferring)] kinase isozyme 1, mitochondrial OS=Rattus norvegicus OX=10116 GN=Pdk1 PE=1 SV=1 - [PDK1_RAT]	49.0	0.013	0.013	0.013	0.013	0.013	0.719	1.294	1.185	0.296	1.177	0.013	0.013	0.013	0.013	0.013	0.458	0.763	0.989	0.749	0.934	0.009	0.0297	#DIV/0!	#DIV/0!	0.009	0.083	0.009	0.055	0.470	0.265	0.009	0.119
Q63347	26S protease regulatory subunit 7 OS=Rattus norvegicus GN=Psmc2 PE=1 SV=3 - [PRS7_RAT]	48.5	0.013	0.013	0.013	0.013	0.013	0.762	0.932	1.284	0.261	0.860	0.901	1.122	0.948	1.249	0.450	0.544	0.497	1.091	0.558	0.982	0.009	0.0445	0.009	0.037	0.009	0.098	0.470	0.678	0.756	0.355	0.470	0.760
Q63362	NADH dehydrogenase [ubiquinone] 1 alpha subcomplex subunit 5 OS=Rattus norvegicus OX=10116 GN=Ndufa5 PE=1 SV=3 - [NDUA5_RAT]	13.4	0.013	0.013	0.013	0.013	0.013	0.648	0.846	1.219	0.180	0.724	0.793	0.830	1.057	1.801	0.405	0.783	0.328	1.473	0.314	1.103	0.009	0.0726	0.009	0.033	0.009	0.075	0.470	0.694	0.756	0.358	0.470	0.733
Q63610	Tropomyosin alpha-3 chain OS=Rattus norvegicus GN=Tpm3 PE=1 SV=2 - [TPM3_RAT]	29.0	0.013	0.013	0.013	0.013	0.013	0.758	1.154	1.234	0.134	0.718	1.091	1.291	1.273	1.838	0.589	1.130	1.361	1.707	1.010	1.153	0.009	0.0488	0.009	0.025	0.009	0.042	0.184	0.454	0.184	0.155	0.917	0.738
Q63622	Disks large homolog 2 OS=Rattus norvegicus GN=Dlg2 PE=1 SV=1 - [DLG2_RAT]	94.9	0.013	0.013	0.013	0.013	0.013	0.815	1.403	1.316	0.230	1.087	0.013	0.013	0.013	0.013	0.013	0.013	0.013	0.013	0.013	0.013	0.009	0.0273	#DIV/0!	#DIV/0!	#DIV/0!	#DIV/0!	0.009	0.050	0.009	0.019	#DIV/0!	#DIV/0!
Q63644	Rho-associated protein kinase 1 OS=Rattus norvegicus GN=Rock1 PE=1 SV=1 - [ROCK1_RAT]	159.5	0.719	0.517	1.961	1.268	0.840	0.013	0.013	0.013	0.013	0.013	0.013	0.013	0.013	0.013	0.013	0.013	0.013	0.013	0.013	0.013	0.009	0.0238	0.009	0.027	0.009	0.046	#DIV/0!	#DIV/0!	#DIV/0!	#DIV/0!	#DIV/0!	#DIV/0!
Q63688	25-hydroxycholesterol 7-alpha-hydroxylase (Fragment) OS=Rattus norvegicus OX=10116 GN=Cyp7b1 PE=1 SV=1 - [CP7B1_RAT]	48.2	0.424	0.474	1.026	0.421	0.431	0.823	1.421	1.106	0.536	1.537	0.881	1.125	1.134	2.028	0.228	0.697	0.714	1.059	0.508	1.585	0.036	0.0787	0.184	0.360	0.056	0.238	0.917	0.714	0.470	0.259	0.470	0.711
Q641Y8	ATP-dependent RNA helicase DDX1 OS=Rattus norvegicus GN=Ddx1 PE=2 SV=1 - [DDX1_RAT]	82.4	0.686	0.725	0.965	0.367	0.351	0.982	1.400	1.350	0.962	1.475	0.367	0.562	1.164	1.386	0.185	0.013	0.013	0.013	0.013	0.013	0.022	0.0510	0.917	0.690	0.009	0.182	0.127	0.375	0.009	0.016	0.009	0.168
Q64240	Protein AMBP OS=Rattus norvegicus GN=Ambp PE=1 SV=1 - [AMBP_RAT]	38.8	0.903	0.561	0.695	0.314	0.354	0.853	1.073	0.936	0.718	1.185	0.308	0.566	0.795	1.111	0.163	0.374	0.618	0.653	0.394	0.673	0.036	0.0790	0.917	0.699	0.917	0.991	0.127	0.402	0.014	0.022	0.917	0.860
Q64268	Heparin cofactor 2 OS=Rattus norvegicus GN=Serpind1 PE=1 SV=1 - [HEP2_RAT]	54.5	0.013	0.013	0.013	0.013	0.013	0.911	1.047	1.195	0.485	1.256	0.434	0.614	0.678	0.795	0.181	0.013	0.013	0.013	0.013	0.013	0.009	0.0266	0.009	0.549	#DIV/0!	#DIV/0!	0.056	0.200	0.009	0.018	0.009	0.715
Q64319	Neutral and basic amino acid transport protein rBAT OS=Rattus norvegicus GN=Slc3a1 PE=1 SV=1 - [SLC31_RAT]	78.5	0.013	0.013	0.013	0.013	0.013	1.493	1.530	1.280	1.931	1.534	0.013	0.013	0.013	0.013	0.013	0.013	0.013	0.013	0.013	0.013	0.009	0.0215	#DIV/0!	#DIV/0!	#DIV/0!	#DIV/0!	0.009	0.036	0.009	0.015	#DIV/0!	#DIV/0!
Q64560	Tripeptidyl-peptidase 2 OS=Rattus norvegicus GN=Tpp2 PE=2 SV=3 - [TPP2_RAT]	138.2	0.740	0.879	1.259	0.401	0.612	0.013	0.013	0.013	0.013	0.013	0.013	0.013	0.013	0.013	0.013	0.442	0.649	0.884	0.629	0.967	0.009	0.0535	0.009	0.095	0.917	0.935	#DIV/0!	#DIV/0!	0.009	0.049	0.009	0.204
Q64602	Kynurenine/alpha-aminoadipate aminotransferase, mitochondrial OS=Rattus norvegicus OX=10116 GN=Aadat PE=1 SV=1 - [AADAT_RAT]	47.8	0.013	0.013	0.013	0.013	0.013	0.841	0.896	0.848	0.377	0.742	0.013	0.013	0.013	0.013	0.013	0.013	0.013	0.013	0.013	0.013	0.009	0.0670	#DIV/0!	#DIV/0!	#DIV/0!	#DIV/0!	0.009	0.153	0.009	0.043	#DIV/0!	#DIV/0!
Q66H61	Glutamine--tRNA ligase OS=Rattus norvegicus OX=10116 GN=Qars PE=1 SV=1 - [SYQ_RAT]	87.6	0.013	0.013	0.013	0.013	0.013	0.504	1.141	0.855	0.206	1.048	0.489	0.694	1.119	1.387	0.098	0.394	0.416	0.013	0.013	0.776	0.009	0.0604	0.009	0.119	0.063	0.262	0.917	0.819	0.084	0.110	0.126	0.439
Q68FS2	COP9 signalosome complex subunit 4 OS=Rattus norvegicus GN=Cops4 PE=1 SV=1 - [CSN4_RAT]	46.3	0.395	0.576	1.062	0.193	0.431	0.982	1.166	1.334	1.247	1.071	0.542	0.932	1.001	1.350	0.321	0.343	0.516	0.712	0.388	0.912	0.022	0.0513	0.354	0.535	0.917	0.992	0.184	0.455	0.014	0.022	0.259	0.766
Q68FU1	Pleckstrin homology domain-containing family F member 1 OS=Rattus norvegicus OX=10116 GN=Plekhf1 PE=2 SV=1 - [PKHF1_RAT]	31.4	1.554	0.896	2.552	2.218	1.053	0.013	0.013	0.013	0.013	0.013	0.013	0.013	0.013	0.013	0.013	0.013	0.013	0.013	0.013	0.013	0.009	0.0213	0.009	0.024	0.009	0.041	#DIV/0!	#DIV/0!	#DIV/0!	#DIV/0!	#DIV/0!	#DIV/0!
Q68FW9	COP9 signalosome complex subunit 3 OS=Rattus norvegicus GN=Cops3 PE=2 SV=1 - [CSN3_RAT]	47.8	0.013	0.013	0.013	0.013	0.013	0.754	1.274	1.187	0.440	1.027	0.383	0.774	0.804	1.084	0.176	0.013	0.013	0.013	0.013	0.013	0.009	0.0292	0.009	0.324	#DIV/0!	#DIV/0!	0.259	0.607	0.009	0.021	0.009	0.318
Q68FX1	Mannose-6-phosphate isomerase OS=Rattus norvegicus GN=Mpi PE=2 SV=1 - [MPI_RAT]	46.4	0.013	0.013	0.013	0.013	0.013	1.155	1.649	1.639	0.359	1.493	0.534	0.913	0.947	1.165	0.242	0.013	0.013	0.013	0.013	0.013	0.009	0.0221	0.009	0.117	#DIV/0!	#DIV/0!	0.127	0.373	0.009	0.015	0.009	0.136
Q6AXM8	Serum paraoxonase/arylesterase 2 OS=Rattus norvegicus OX=10116 GN=Pon2 PE=2 SV=1 - [PON2_RAT]	39.6	0.013	0.013	0.013	0.013	0.013	0.872	1.055	1.180	0.535	0.957	0.013	0.013	0.013	0.013	0.013	0.013	0.013	0.013	0.013	0.013	0.009	0.0313	#DIV/0!	#DIV/0!	#DIV/0!	#DIV/0!	0.009	0.059	0.009	0.022	#DIV/0!	#DIV/0!
Q6AXS5	Plasminogen activator inhibitor 1 RNA-binding protein OS=Rattus norvegicus OX=10116 GN=Serbp1 PE=1 SV=2 - [PAIRB_RAT]	44.7	0.868	0.648	1.527	0.170	0.539	0.013	0.013	0.013	0.013	0.013	0.985	0.780	1.242	1.727	0.404	0.830	0.941	1.571	0.729	0.968	0.009	0.0616	0.354	0.465	0.184	0.529	0.009	0.045	0.009	0.018	0.756	0.724
Q6AY19	Atypical kinase COQ8B, mitochondrial OS=Rattus norvegicus OX=10116 GN=Coq8b PE=1 SV=1 - [COQ8B_RAT]	58.9	0.651	0.498	1.710	0.713	0.702	0.013	0.013	0.013	0.013	0.013	0.939	0.832	0.888	1.260	0.932	0.763	0.797	1.524	0.825	0.823	0.009	0.0365	0.127	0.278	0.127	0.425	0.009	0.050	0.009	0.020	0.127	0.428
Q6AY20	Cation-dependent mannose-6-phosphate receptor OS=Rattus norvegicus OX=10116 GN=M6pr PE=1 SV=1 - [MPRD_RAT]	31.1	0.013	0.013	0.013	0.013	0.013	0.644	0.789	0.914	0.575	0.671	1.019	0.998	0.736	0.934	0.639	0.550	0.772	0.870	0.602	0.924	0.009	0.0735	0.009	0.053	0.009	0.094	0.184	0.506	0.917	0.394	0.184	0.581
Q6AY30	Saccharopine dehydrogenase-like oxidoreductase OS=Rattus norvegicus OX=10116 GN=Sccpdh PE=1 SV=1 - [SCPDL_RAT]	47.1	0.013	0.013	0.013	0.013	0.013	0.587	1.215	0.854	0.452	1.110	0.643	1.208	0.568	1.491	0.266	0.538	0.344	0.674	0.313	1.637	0.009	0.0388	0.009	0.064	0.009	0.118	0.917	0.775	0.354	0.240	0.756	0.828
Q6AY80	Ribosyldihydronicotinamide dehydrogenase [quinone] OS=Rattus norvegicus OX=10116 GN=Nqo2 PE=1 SV=3 - [NQO2_RAT]	26.3	0.013	0.013	0.013	0.013	0.013	0.646	1.059	1.316	0.207	0.782	0.013	0.013	0.013	0.013	0.013	0.013	0.013	0.013	0.013	0.013	0.009	0.0484	#DIV/0!	#DIV/0!	#DIV/0!	#DIV/0!	0.009	0.101	0.009	0.031	#DIV/0!	#DIV/0!
Q6AYC8	SH2 domain-containing protein 4A OS=Rattus norvegicus OX=10116 GN=Sh2d4a PE=2 SV=1 - [SH24A_RAT]	48.5	0.013	0.013	0.013	0.013	0.013	0.881	0.831	1.375	0.053	0.792	0.013	0.013	0.013	0.013	0.013	0.013	0.013	0.013	0.013	0.013	0.009	0.0517	#DIV/0!	#DIV/0!	#DIV/0!	#DIV/0!	0.009	0.113	0.009	0.032	#DIV/0!	#DIV/0!
Q6AYG3	Protein prune homolog OS=Rattus norvegicus GN=Prune PE=2 SV=1 - [PRUNE_RAT]	50.0	0.013	0.013	0.013	0.013	0.013	0.774	1.232	1.373	0.014	0.773	0.630	0.991	0.871	1.416	0.297	0.548	0.583	0.929	0.719	0.839	0.009	0.0173	0.009	0.061	0.009	0.105	0.917	0.777	0.470	0.277	0.470	0.826
Q6AYQ4	Transmembrane protein 109 OS=Rattus norvegicus OX=10116 GN=Tmem109 PE=2 SV=1 - [TM109_RAT]	26.2	0.013	0.013	0.013	0.013	0.013	0.668	1.740	0.990	0.254	1.032	1.166	1.403	0.643	1.756	0.489	0.880	0.541	1.060	0.499	1.453	0.009	0.0289	0.009	0.026	0.009	0.058	0.605	0.673	0.917	0.370	0.605	0.726
Q6AZ50	Ubiquitin-like-conjugating enzyme ATG3 OS=Rattus norvegicus GN=Atg3 PE=2 SV=1 - [ATG3_RAT]	35.8	0.013	0.013	0.013	0.013	0.013	0.681	1.148	1.458	0.148	1.035	0.574	0.997	1.100	1.950	0.301	0.614	0.426	0.726	0.413	0.913	0.009	0.0331	0.009	0.033	0.009	0.186	0.917	0.724	0.259	0.207	0.354	0.826
Q6IG12	Keratin, type II cytoskeletal 7 OS=Rattus norvegicus GN=Krt7 PE=3 SV=1 - [K2C7_RAT]	50.7	0.726	0.641	0.922	0.814	0.469	0.013	0.013	0.013	0.013	0.013	0.013	0.013	0.013	0.013	0.013	0.013	0.013	0.013	0.013	0.013	0.009	0.0762	0.009	0.155	0.009	0.109	#DIV/0!	#DIV/0!	#DIV/0!	#DIV/0!	#DIV/0!	#DIV/0!
Q6IUU3	Sulfhydryl oxidase 1 OS=Rattus norvegicus GN=Qsox1 PE=1 SV=1 - [QSOX1_RAT]	82.4	0.803	0.569	1.072	0.452	0.605	0.013	0.013	0.013	0.013	0.013	0.013	0.013	0.013	0.013	0.013	0.435	0.677	0.695	0.483	0.861	0.009	0.0859	0.009	0.188	0.756	0.960	#DIV/0!	#DIV/0!	0.009	0.091	0.009	0.343
Q6P6S0	Synaptosomal-associated protein 47 OS=Rattus norvegicus OX=10116 GN=Snap47 PE=2 SV=1 - [SNP47_RAT]	47.0	0.013	0.013	0.013	0.013	0.013	1.008	1.170	1.224	0.915	1.064	0.013	0.013	0.013	0.013	0.013	0.013	0.013	0.013	0.013	0.013	0.009	0.0234	#DIV/0!	#DIV/0!	#DIV/0!	#DIV/0!	0.009	0.042	0.009	0.017	#DIV/0!	#DIV/0!
Q6P7S1	Acid ceramidase OS=Rattus norvegicus GN=Asah1 PE=2 SV=1 - [ASAH1_RAT]	44.4	0.013	0.013	0.013	0.013	0.013	0.808	1.241	1.053	0.300	0.992	0.628	0.795	0.789	1.286	0.339	0.512	0.689	0.741	0.402	0.831	0.009	0.0343	0.009	0.106	0.009	0.172	0.470	0.717	0.184	0.169	0.605	0.863
Q6PCT3	Tumor protein D54 OS=Rattus norvegicus GN=Tpd52l2 PE=2 SV=1 - [TPD54_RAT]	24.0	0.013	0.013	0.013	0.013	0.013	0.554	0.783	1.003	0.551	0.594	1.084	1.137	0.755	1.941	0.381	0.803	0.565	1.123	0.502	1.122	0.009	0.0910	0.009	0.027	0.009	0.071	0.259	0.553	0.470	0.279	0.605	0.734
Q6SKG1	Acyl-coenzyme A synthetase ACSM3, mitochondrial OS=Rattus norvegicus OX=10116 GN=Acsm3 PE=2 SV=1 - [ACSM3_RAT]	65.7	0.013	0.013	0.013	0.013	0.013	1.024	1.008	1.425	0.045	0.808	0.013	0.013	0.013	0.013	0.013	0.013	0.013	0.013	0.013	0.013	0.009	0.0357	#DIV/0!	#DIV/0!	#DIV/0!	#DIV/0!	0.009	0.073	0.009	0.024	#DIV/0!	#DIV/0!
Q75WE7	von Willebrand factor A domain-containing protein 5A OS=Rattus norvegicus GN=Vwa5a PE=2 SV=1 - [VWA5A_RAT]	91.4	0.860	0.635	1.688	0.887	0.759	0.013	0.013	0.013	0.013	0.013	0.013	0.013	0.013	0.013	0.013	0.347	0.612	0.765	0.464	0.723	0.009	0.0275	0.009	0.034	0.056	0.236	#DIV/0!	#DIV/0!	0.009	0.137	0.009	0.409
Q7TP48	Adipocyte plasma membrane-associated protein OS=Rattus norvegicus OX=10116 GN=Apmap PE=2 SV=2 - [APMAP_RAT]	42.0	0.013	0.013	0.013	0.013	0.013	0.543	1.099	0.918	0.252	0.983	0.244	0.621	0.608	1.177	0.076	0.013	0.013	0.013	0.013	0.013	0.009	0.0581	0.009	0.509	#DIV/0!	#DIV/0!	0.470	0.766	0.009	0.037	0.009	0.660
Q8CGS5	Zinc phosphodiesterase ELAC protein 2 OS=Rattus norvegicus GN=Elac2 PE=1 SV=1 - [RNZ2_RAT]	92.3	0.013	0.013	0.013	0.013	0.013	1.180	1.070	1.343	0.348	0.888	0.622	0.533	0.871	1.289	0.216	0.013	0.013	0.013	0.013	0.013	0.009	0.0276	0.009	0.174	#DIV/0!	#DIV/0!	0.259	0.579	0.009	0.019	0.009	0.226
Q8K1Q0	Glycylpeptide N-tetradecanoyltransferase 1 OS=Rattus norvegicus GN=Nmt1 PE=1 SV=1 - [NMT1_RAT]	56.8	0.679	0.540	0.409	0.259	0.144	0.867	0.936	1.164	0.623	0.809	0.698	0.917	1.201	1.496	0.333	0.696	0.926	1.229	0.898	1.172	0.022	0.0515	0.056	0.138	0.014	0.061	0.756	0.708	0.354	0.222	0.917	0.753
Q8K4D8	Aldehyde dehydrogenase family 1 member A3 OS=Rattus norvegicus GN=Aldh1a3 PE=2 SV=1 - [AL1A3_RAT]	56.1	0.013	0.013	0.013	0.013	0.013	1.024	1.256	1.278	0.495	1.171	0.503	0.872	0.993	1.192	0.222	0.013	0.013	0.013	0.013	0.013	0.009	0.0244	0.009	0.121	#DIV/0!	#DIV/0!	0.184	0.465	0.009	0.017	0.009	0.141
Q8K4G6	ADP-ribose glycohydrolase MACROD1 (Fragment) OS=Rattus norvegicus OX=10116 GN=Macrod1 PE=2 SV=2 - [MACD1_RAT]	28.6	0.013	0.013	0.013	0.013	0.013	0.792	0.819	1.164	0.114	0.678	0.707	1.006	0.935	1.371	0.424	0.707	1.089	1.371	0.567	1.085	0.009	0.0782	0.009	0.047	0.009	0.052	0.470	0.736	0.470	0.267	0.470	0.723
Q8K4M9	Oxysterol-binding protein-related protein 1 OS=Rattus norvegicus OX=10116 GN=Osbpl1a PE=1 SV=1 - [OSBL1_RAT]	107.7	0.013	0.013	0.013	0.013	0.013	0.909	1.380	1.166	0.605	1.174	0.621	0.838	0.898	1.292	0.179	0.365	0.745	0.707	0.551	0.849	0.009	0.0242	0.009	0.110	0.009	0.159	0.259	0.540	0.056	0.075	0.470	0.915
Q8K581	Thioredoxin domain-containing protein 9 OS=Rattus norvegicus GN=Txndc9 PE=2 SV=2 - [TXND9_RAT]	26.3	0.013	0.013	0.013	0.013	0.013	0.585	0.792	1.172	0.013	0.494	0.013	0.013	0.013	0.013	0.013	0.013	0.013	0.013	0.013	0.013	0.025	0.0561	#DIV/0!	#DIV/0!	#DIV/0!	#DIV/0!	0.025	0.093	0.025	0.037	#DIV/0!	#DIV/0!
Q8VD87	Ribonuclease pancreatic gamma-type OS=Rattus rattus OX=10117 PE=3 SV=1 - [RNS1G_RATRT]	16.5	0.013	0.013	0.013	0.013	0.013	0.574	0.677	0.958	0.780	0.785	0.013	0.013	0.013	0.013	0.013	0.013	0.013	0.013	0.013	0.013	0.009	0.0592	#DIV/0!	#DIV/0!	#DIV/0!	#DIV/0!	0.009	0.143	0.009	0.039	#DIV/0!	#DIV/0!
Q99ML5	Prenylcysteine oxidase OS=Rattus norvegicus OX=10116 GN=Pcyox1 PE=1 SV=1 - [PCYOX_RAT]	56.3	0.250	0.348	0.763	0.343	0.462	0.794	1.396	1.426	0.356	1.154	0.497	0.906	0.791	1.859	0.384	0.623	0.496	0.661	0.322	1.315	0.036	0.0795	0.056	0.138	0.259	0.706	0.605	0.679	0.184	0.161	0.470	0.822
Q9EQ10	PCNA-interacting partner OS=Rattus norvegicus OX=10116 GN=Parpbp PE=1 SV=2 - [PARI_RAT]	63.9	1.894	1.205	3.098	0.572	1.184	0.013	0.013	0.013	0.013	0.013	1.457	1.720	0.621	0.685	0.909	0.792	2.016	1.888	1.788	1.250	0.009	0.0215	0.470	0.509	0.756	0.830	0.009	0.042	0.009	0.015	0.127	0.422
Q9EST6	Acidic leucine-rich nuclear phosphoprotein 32 family member B OS=Rattus norvegicus GN=Anp32b PE=2 SV=1 - [AN32B_RAT]	31.0	1.052	0.819	1.601	0.819	0.735	0.013	0.013	0.013	0.013	0.013	0.905	0.886	1.352	1.990	0.606	1.040	0.900	1.520	0.696	0.839	0.009	0.0256	0.605	0.554	0.917	0.847	0.009	0.040	0.009	0.018	0.756	0.719
Q9HB97	Alpha-parvin OS=Rattus norvegicus GN=Parva PE=1 SV=2 - [PARVA_RAT]	42.3	0.013	0.013	0.013	0.013	0.013	0.847	1.128	1.151	0.609	0.950	0.013	0.013	0.013	0.013	0.013	0.013	0.013	0.013	0.013	0.013	0.009	0.0290	#DIV/0!	#DIV/0!	#DIV/0!	#DIV/0!	0.009	0.054	0.009	0.021	#DIV/0!	#DIV/0!
Q9JHU5	Arfaptin-1 OS=Rattus norvegicus GN=Arfip1 PE=1 SV=1 - [ARFP1_RAT]	40.8	1.313	1.231	1.939	1.161	1.049	0.984	0.595	1.104	0.138	0.421	1.100	1.620	1.071	1.041	0.712	0.872	1.221	1.551	1.229	1.259	0.022	0.0505	0.184	0.347	0.756	0.831	0.127	0.386	0.036	0.051	0.354	0.748
Q9JIX3	Bis(5'-adenosyl)-triphosphatase OS=Rattus norvegicus OX=10116 GN=Fhit PE=1 SV=1 - [FHIT_RAT]	17.3	0.013	0.013	0.013	0.013	0.013	0.669	1.186	0.947	0.171	1.048	0.599	0.806	0.813	1.138	0.356	0.013	0.013	0.013	0.013	0.013	0.009	0.0480	0.009	0.132	#DIV/0!	#DIV/0!	0.605	0.793	0.009	0.031	0.009	0.153
Q9JJ19	Na(+)/H(+) exchange regulatory cofactor NHE-RF1 OS=Rattus norvegicus OX=10116 GN=Slc9a3r1 PE=1 SV=3 - [NHRF1_RAT]	38.8	0.013	0.013	0.013	0.013	0.013	0.694	0.450	0.870	0.013	0.508	0.013	0.013	0.013	0.013	0.013	0.013	0.013	0.013	0.013	0.013	0.025	0.0563	#DIV/0!	#DIV/0!	#DIV/0!	#DIV/0!	0.025	0.094	0.025	0.037	#DIV/0!	#DIV/0!
Q9JJ22	Endoplasmic reticulum aminopeptidase 1 OS=Rattus norvegicus GN=Erap1 PE=2 SV=2 - [ERAP1_RAT]	106.4	1.018	0.740	1.510	0.690	0.698	0.013	0.013	0.013	0.013	0.013	0.448	0.781	0.984	1.097	0.314	0.390	0.697	0.763	0.611	0.854	0.009	0.0299	0.605	0.592	0.259	0.655	0.009	0.178	0.009	0.073	0.605	0.870
Q9JJ31	Cullin-5 OS=Rattus norvegicus GN=Cul5 PE=1 SV=3 - [CUL5_RAT]	90.8	0.013	0.013	0.013	0.013	0.013	0.751	1.162	1.088	0.222	0.995	0.013	0.013	0.013	0.013	0.013	0.013	0.013	0.013	0.013	0.013	0.009	0.0386	#DIV/0!	#DIV/0!	#DIV/0!	#DIV/0!	0.009	0.081	0.009	0.026	#DIV/0!	#DIV/0!
Q9JLD2	Neuroserpin OS=Rattus norvegicus GN=Serpini1 PE=1 SV=1 - [NEUS_RAT]	46.2	0.013	0.013	0.013	0.013	0.013	1.083	1.105	1.233	0.127	0.975	0.805	1.102	0.788	1.121	0.382	0.582	1.119	1.082	0.822	1.069	0.009	0.0324	0.009	0.061	0.009	0.053	0.605	0.711	0.605	0.299	0.756	0.747
Q9JMJ4	Pre-mRNA-processing factor 19 OS=Rattus norvegicus GN=Prpf19 PE=1 SV=2 - [PRP19_RAT]	55.2	0.013	0.013	0.013	0.013	0.013	0.759	1.147	1.112	0.835	0.945	0.013	0.013	0.013	0.013	0.013	0.405	0.523	1.044	0.621	1.028	0.009	0.0277	#DIV/0!	#DIV/0!	0.009	0.104	0.009	0.051	0.184	0.162	0.009	0.187
Q9QZ81	Protein argonaute-2 OS=Rattus norvegicus GN=Ago2 PE=2 SV=2 - [AGO2_RAT]	97.3	0.013	0.013	0.013	0.013	0.013	1.140	1.200	1.095	0.334	1.087	0.013	0.013	0.013	0.013	0.013	0.013	0.013	0.013	0.013	0.013	0.009	0.0271	#DIV/0!	#DIV/0!	#DIV/0!	#DIV/0!	0.009	0.049	0.009	0.019	#DIV/0!	#DIV/0!
Q9R0D6	Transcobalamin-2 OS=Rattus norvegicus GN=Tcn2 PE=2 SV=1 - [TCO2_RAT]	47.4	0.667	0.473	0.669	0.329	0.332	0.772	0.880	0.969	1.006	1.007	0.013	0.013	0.013	0.013	0.013	0.388	0.656	0.693	0.403	0.666	0.014	0.0323	0.009	0.792	0.605	0.952	0.009	0.057	0.014	0.022	0.009	0.477
Q9WVR7	Protein phosphatase 1F OS=Rattus norvegicus GN=Ppm1f PE=2 SV=1 - [PPM1F_RAT]	49.1	0.013	0.013	0.013	0.013	0.013	0.836	1.224	1.662	0.110	1.091	0.297	0.529	1.005	1.424	0.167	0.013	0.013	0.013	0.013	0.013	0.009	0.0265	0.009	0.216	#DIV/0!	#DIV/0!	0.470	0.708	0.009	0.018	0.009	0.268
Q9Z252	Protein lin-7 homolog B OS=Rattus norvegicus GN=Lin7b PE=1 SV=1 - [LIN7B_RAT]	22.9	0.450	0.736	2.065	0.732	0.502	0.013	0.013	0.013	0.013	0.013	0.013	0.013	0.013	0.013	0.013	0.013	0.013	0.013	0.013	0.013	0.009	0.0330	0.009	0.044	0.009	0.056	#DIV/0!	#DIV/0!	#DIV/0!	#DIV/0!	#DIV/0!	#DIV/0!
Q9Z270	Vesicle-associated membrane protein-associated protein A OS=Rattus norvegicus OX=10116 GN=Vapa PE=1 SV=3 - [VAPA_RAT]	27.8	0.013	0.013	0.013	0.013	0.013	0.693	1.046	1.061	0.162	0.777	0.531	0.749	0.907	1.480	0.223	0.582	0.550	0.737	0.534	0.843	0.009	0.0635	0.009	0.096	0.009	0.154	0.917	0.818	0.354	0.246	0.756	0.852
Q9Z2L0	Voltage-dependent anion-selective channel protein 1 OS=Rattus norvegicus OX=10116 GN=Vdac1 PE=1 SV=4 - [VDAC1_RAT]	30.7	0.013	0.013	0.013	0.013	0.013	0.879	0.871	1.112	0.702	0.630	0.013	0.013	0.013	0.013	0.013	0.013	0.013	0.013	0.013	0.013	0.009	0.0396	#DIV/0!	#DIV/0!	#DIV/0!	#DIV/0!	0.009	0.083	0.009	0.026	#DIV/0!	#DIV/0!

Table S10. Extracted protein profile of e-II (Effect of nutrition, only FGR, Female)

Accession	Description	MW [kDa]	Expression level																				Steel-Dwass & FDR											
			FGR-Good-Female					FGR-Poor_Female					Sham-Good-Female					Sham-Poor-Female					FGR-GN vs FGR-PN		FGR-GN vs Sham-GN		FGR-GN vs Sham-PN		FGR-PN vs Sham-GN		FGR-PN vs Sham-PN		Sham-GN vs Sham-PN	
			#01	#02	#03	#04	#05	#06	#07	#08	#09	#10	#11	#12	#13	#14	#15	#16	#17	#18	#19	#20												
Filter	Filter	Filter	Filter	Filter	Filter	Filter	Filter	Filter	Filter	Filter	Filter	Filter	Filter	Filter	Filter	Filter	Filter	Filter	Filter	Filter	Filter	Filter	p-value	q-value	p	q-value	p	q-value	p	q-value	p	q-value	p	q-value
A1L1L2	Transmembrane protein 214 OS=Rattus norvegicus GN=Tmem214 PE=2 SV=1 - [TM214_RAT]	76.5	0.013	0.013	0.013	0.013	0.013	3.403	0.821	0.685	0.733	0.637	1.101	1.069	1.453	0.648	0.905	0.013	0.013	0.013	0.013	0.013	0.009	0.052	0.009	0.111	#DIV/0!	#DIV/0!	0.354	0.275	0.009	0.049	0.009	0.275
A4L9P8	Centrosomal protein of 295 kDa OS=Rattus norvegicus OX=10116 GN=Cep295 PE=2 SV=2 - [CE295_RAT]	269.8	1.746	0.315	0.878	0.713	0.807	0.013	0.013	0.013	0.013	0.013	0.013	0.013	0.013	0.013	0.013	0.013	0.013	0.013	0.013	0.013	0.009	0.097	0.009	0.268	0.009	0.205	#DIV/0!	#DIV/0!	#DIV/0!	#DIV/0!	#DIV/0!	#DIV/0!
B0BN93	26S proteasome non-ATPase regulatory subunit 13 OS=Rattus norvegicus GN=Psmd13 PE=1 SV=1 - [PSD13_RAT]	42.8	0.973	0.941	0.787	1.189	1.036	0.013	0.013	0.013	0.013	0.013	0.978	1.078	1.601	1.895	1.079	2.350	0.566	1.545	1.415	1.338	0.009	0.064	0.085	0.111	0.127	0.159	0.009	0.055	0.009	0.036	0.756	1.317
B0K019	BAG family molecular chaperone regulator 1 OS=Rattus norvegicus OX=10116 GN=Bag1 PE=2 SV=1 - [BAG1_RAT]	40.1	0.013	0.013	0.013	0.013	0.013	0.893	0.339	0.647	1.309	1.381	0.013	0.013	0.013	0.013	0.013	0.013	0.013	0.013	0.013	0.013	0.009	0.080	#DIV/0!	#DIV/0!	#DIV/0!	#DIV/0!	0.009	0.160	0.009	0.130	#DIV/0!	#DIV/0!
B0K020	CDGSH iron-sulfur domain-containing protein 1 OS=Rattus norvegicus OX=10116 GN=Cisd1 PE=3 SV=1 - [CISD1_RAT]	12.1	1.050	0.892	0.612	1.151	0.918	0.013	0.013	0.013	0.013	0.013	0.903	0.765	1.465	1.980	0.701	2.262	0.861	1.275	1.198	0.589	0.009	0.074	0.756	0.652	0.470	0.437	0.009	0.071	0.009	0.051	0.917	1.241
B2GV24	E3 UFM1-protein ligase 1 OS=Rattus norvegicus GN=Ufl1 PE=1 SV=1 - [UFL1_RAT]	89.5	1.374	0.775	0.949	1.239	1.009	0.013	0.013	0.013	0.013	0.013	0.741	0.791	1.258	1.016	0.880	1.128	0.960	1.378	1.083	0.393	0.009	0.056	0.470	0.440	0.917	0.768	0.009	0.144	0.009	0.099	0.470	1.826
B5DF91	ELAV-like protein 1 OS=Rattus norvegicus OX=10116 GN=Elavl1 PE=1 SV=1 - [ELAV1_RAT]	36.1	0.930	0.720	0.693	1.228	1.019	0.013	0.013	0.013	0.013	0.013	0.878	0.989	1.759	1.726	0.906	1.096	0.960	0.962	1.133	0.840	0.009	0.078	0.354	0.345	0.470	0.450	0.009	0.062	0.009	0.095	0.605	1.683
D2XV59	GTP-binding protein 1 OS=Rattus norvegicus GN=Gtpbp1 PE=1 SV=1 - [GTPB1_RAT]	72.4	0.776	0.907	0.816	1.046	1.047	0.013	0.013	0.013	0.013	0.013	0.013	0.013	0.013	0.013	0.013	0.013	0.013	0.013	0.013	0.013	0.009	0.077	0.009	0.209	0.009	0.163	#DIV/0!	#DIV/0!	#DIV/0!	#DIV/0!	#DIV/0!	#DIV/0!
D3ZA12	Chromodomain-helicase-DNA-binding protein 6 OS=Rattus norvegicus GN=Chd6 PE=1 SV=2 - [CHD6_RAT]	303.3	0.013	0.013	0.013	0.013	0.013	1.062	0.894	0.796	1.108	0.613	0.401	0.422	0.931	0.334	0.648	0.013	0.013	0.013	0.013	0.013	0.009	0.096	0.009	5.225	#DIV/0!	#DIV/0!	0.085	0.098	0.009	0.147	0.009	3.227
D3ZF77	Aldo-keto reductase family 1 member C15 OS=Rattus norvegicus OX=10116 GN=Akr1c15 PE=1 SV=1 - [AK1CA_RAT]	37.2	0.736	1.090	0.735	1.198	0.994	0.013	0.013	0.013	0.013	0.013	0.741	0.485	2.207	0.840	1.356	1.169	0.955	1.314	1.322	1.635	0.009	0.070	0.756	0.653	0.085	0.123	0.009	0.075	0.009	0.048	0.470	1.682
D3ZLZ7	Inosine-5'-monophosphate dehydrogenase 1 OS=Rattus norvegicus GN=Impdh1 PE=3 SV=1 - [IMDH1_RAT]	55.3	0.898	1.134	0.925	1.194	1.279	0.013	0.013	0.013	0.013	0.013	1.372	1.295	2.358	1.234	1.212	0.840	0.583	1.802	1.350	0.834	0.009	0.055	0.036	0.055	0.605	0.540	0.009	0.044	0.009	0.069	0.259	1.331
D4ABX8	Leucine-rich repeat and fibronectin type-III domain-containing protein 4 OS=Rattus norvegicus OX=10116 GN=Lrfn4 PE=1 SV=1 - [LRFN4_RAT]	67.2	0.013	0.013	0.013	0.013	0.013	1.495	1.151	1.173	0.643	0.632	0.013	0.013	0.013	0.013	0.013	0.013	0.013	0.013	0.013	0.013	0.009	0.060	#DIV/0!	#DIV/0!	#DIV/0!	#DIV/0!	0.009	0.108	0.009	0.084	#DIV/0!	#DIV/0!
O35112	CD166 antigen OS=Rattus norvegicus GN=Alcam PE=1 SV=1 - [CD166_RAT]	65.0	0.013	0.013	0.013	0.013	0.013	1.512	0.735	0.648	0.930	0.761	1.075	0.926	1.999	1.225	0.752	0.013	0.013	0.013	0.013	0.013	0.009	0.079	0.009	0.076	#DIV/0!	#DIV/0!	0.259	0.211	0.009	0.126	0.009	0.154
O35394	Prenylated Rab acceptor protein 1 OS=Rattus norvegicus OX=10116 GN=Rabac1 PE=1 SV=1 - [PRAF1_RAT]	20.6	0.749	1.057	0.485	1.546	0.966	0.013	0.013	0.013	0.013	0.013	0.013	0.013	0.013	0.013	0.013	0.013	0.013	0.013	0.013	0.013	0.009	0.067	0.009	0.171	0.009	0.135	#DIV/0!	#DIV/0!	#DIV/0!	#DIV/0!	#DIV/0!	#DIV/0!
O35828	Coronin-7 OS=Rattus norvegicus GN=Coro7 PE=1 SV=2 - [CORO7_RAT]	100.7	1.036	0.757	0.750	0.946	0.985	0.013	0.013	0.013	0.013	0.013	0.873	0.936	1.737	2.028	1.187	2.887	0.418	1.549	1.331	1.457	0.009	0.095	0.184	0.202	0.127	0.159	0.009	0.052	0.009	0.031	0.756	1.271
O70260	E3 SUMO-protein ligase PIAS3 OS=Rattus norvegicus OX=10116 GN=Pias3 PE=1 SV=2 - [PIAS3_RAT]	68.3	0.013	0.013	0.013	0.013	0.013	1.275	1.686	1.309	0.188	0.296	0.013	0.013	0.013	0.013	0.013	0.013	0.013	0.013	0.013	0.013	0.009	0.069	#DIV/0!	#DIV/0!	#DIV/0!	#DIV/0!	0.009	0.138	0.009	0.112	#DIV/0!	#DIV/0!
O70593	Small glutamine-rich tetratricopeptide repeat-containing protein alpha OS=Rattus norvegicus GN=Sgta PE=1 SV=1 - [SGTA_RAT]	34.1	1.040	0.820	0.698	1.283	0.998	0.013	0.013	0.013	0.013	0.013	0.813	0.792	1.685	2.154	0.832	1.816	0.952	1.029	0.980	0.872	0.009	0.067	0.756	0.646	0.756	0.655	0.009	0.061	0.009	0.063	0.470	1.694
P00564	Creatine kinase M-type OS=Rattus norvegicus GN=Ckm PE=1 SV=2 - [KCRM_RAT]	43.0	0.013	0.013	0.013	0.013	0.013	0.712	0.405	0.538	0.757	2.981	0.887	1.119	1.966	0.835	1.513	1.571	0.653	1.477	1.225	0.843	0.009	0.056	0.009	0.068	0.009	0.073	0.127	0.117	0.259	0.212	0.756	1.461
P01835	Ig kappa chain C region, B allele OS=Rattus norvegicus PE=1 SV=1 - [KACB_RAT]	11.6	0.424	0.170	0.416	0.765	0.530	1.212	1.002	1.062	1.051	0.996	1.118	0.843	2.165	0.545	1.038	3.251	0.458	2.181	1.206	1.328	0.014	0.077	0.022	0.045	0.036	0.084	0.756	0.548	0.184	0.159	0.259	1.295
P04897	Guanine nucleotide-binding protein G(i) subunit alpha-2 OS=Rattus norvegicus GN=Gnai2 PE=1 SV=3 - [GNAI2_RAT]	40.5	1.192	0.768	0.773	1.061	0.895	0.013	0.013	0.013	0.013	0.013	0.908	0.778	1.559	2.017	0.777	1.774	1.288	1.290	1.381	0.722	0.009	0.071	0.354	0.347	0.127	0.164	0.009	0.066	0.009	0.047	0.917	1.221
P05964	Protein S100-A6 OS=Rattus norvegicus GN=S100a6 PE=1 SV=3 - [S10A6_RAT]	10.0	1.121	0.809	0.742	1.157	0.872	0.013	0.013	0.013	0.013	0.013	0.729	0.653	1.638	1.189	0.735	0.013	0.013	0.013	0.013	0.013	0.009	0.070	0.605	0.542	0.009	0.140	0.009	0.121	#DIV/0!	#DIV/0!	0.009	0.349
P06302	Prothymosin alpha OS=Rattus norvegicus OX=10116 GN=Ptma PE=1 SV=2 - [PTMA_RAT]	12.4	0.013	0.013	0.013	0.013	0.013	0.920	0.654	0.986	1.008	0.860	0.013	0.013	0.013	0.013	0.013	0.013	0.013	0.013	0.013	0.013	0.009	0.0998	#DIV/0!	#DIV/0!	#DIV/0!	#DIV/0!	0.009	0.186	0.009	0.152	#DIV/0!	#DIV/0!
P07153	Dolichyl-diphosphooligosaccharide--protein glycosyltransferase subunit 1 OS=Rattus norvegicus GN=Rpn1 PE=2 SV=1 - [RPN1_RAT]	68.3	0.982	0.887	0.672	1.215	1.144	0.013	0.013	0.013	0.013	0.013	1.346	1.083	2.825	1.319	1.332	1.429	0.962	1.209	1.412	0.851	0.009	0.065	0.036	0.056	0.354	0.353	0.009	0.038	0.009	0.057	0.470	1.517
P11240	Cytochrome c oxidase subunit 5A, mitochondrial OS=Rattus norvegicus OX=10116 GN=Cox5a PE=1 SV=1 - [COX5A_RAT]	16.1	1.129	0.739	0.749	1.343	1.104	0.013	0.013	0.013	0.013	0.013	0.013	0.013	0.013	0.013	0.013	0.013	0.013	0.013	0.013	0.013	0.009	0.061	0.009	0.127	0.009	0.106	#DIV/0!	#DIV/0!	#DIV/0!	#DIV/0!	#DIV/0!	#DIV/0!
P12749	60S ribosomal protein L26 OS=Rattus norvegicus GN=Rpl26 PE=1 SV=1 - [RL26_RAT]	17.3	1.050	0.895	0.786	1.735	0.866	0.013	0.013	0.013	0.013	0.013	0.013	0.013	0.013	0.013	0.013	0.535	1.178	1.884	1.117	0.414	0.009	0.056	0.009	0.101	0.917	0.764	#DIV/0!	#DIV/0!	0.009	0.080	0.009	0.300
P15205	Microtubule-associated protein 1B OS=Rattus norvegicus OX=10116 GN=Map1b PE=1 SV=3 - [MAP1B_RAT]	269.5	0.013	0.013	0.013	0.013	0.013	2.193	1.508	1.114	0.135	0.276	0.013	0.013	0.013	0.013	0.013	0.101	2.592	0.154	0.322	0.214	0.009	0.057	#DIV/0!	#DIV/0!	0.009	1.139	0.009	0.096	0.470	0.360	0.009	1.613
P18265	Glycogen synthase kinase-3 alpha OS=Rattus norvegicus GN=Gsk3a PE=1 SV=1 - [GSK3A_RAT]	51.0	1.049	1.124	0.881	1.433	1.117	0.013	0.013	0.013	0.013	0.013	0.987	0.850	2.088	0.810	1.171	0.565	1.432	1.111	0.996	0.986	0.009	0.053	0.605	0.533	0.354	0.354	0.009	0.068	0.009	0.086	0.917	1.289
P18395	Cold shock domain-containing protein E1 OS=Rattus norvegicus OX=10116 GN=Csde1 PE=2 SV=1 - [CSDE1_RAT]	88.8	1.166	0.952	0.954	1.063	0.811	0.013	0.013	0.013	0.013	0.013	1.088	1.116	1.877	1.976	0.651	1.453	0.744	1.370	1.172	0.523	0.009	0.064	0.259	0.264	0.605	0.545	0.009	0.053	0.009	0.075	0.605	1.627
P21708	Mitogen-activated protein kinase 3 OS=Rattus norvegicus GN=Mapk3 PE=1 SV=5 - [MK03_RAT]	43.1	0.013	0.013	0.013	0.013	0.013	1.223	0.620	0.891	0.770	0.973	0.793	0.711	1.682	1.330	0.922	1.300	1.023	0.944	1.009	0.893	0.009	0.095	0.009	0.096	0.009	0.095	0.470	0.358	0.184	0.173	0.756	1.565
P25235	Dolichyl-diphosphooligosaccharide--protein glycosyltransferase subunit 2 OS=Rattus norvegicus OX=10116 GN=Rpn2 PE=2 SV=2 - [RPN2_RAT]	69.0	2.018	1.854	1.501	1.606	1.954	1.035	0.821	0.786	0.867	0.769	1.160	1.069	1.271	1.651	1.002	0.889	1.191	1.068	1.379	0.908	0.014	0.076	0.036	0.054	0.014	0.038	0.022	0.040	0.036	0.077	0.354	1.630
P27615	Lysosome membrane protein 2 OS=Rattus norvegicus OX=10116 GN=Scarb2 PE=1 SV=2 - [SCRB2_RAT]	54.1	1.391	0.824	0.736	1.186	0.900	0.013	0.013	0.013	0.013	0.013	0.013	0.013	0.013	0.013	0.013	0.013	0.013	0.013	0.013	0.013	0.009	0.061	0.009	0.134	0.009	0.109	#DIV/0!	#DIV/0!	#DIV/0!	#DIV/0!	#DIV/0!	#DIV/0!
P31000	Vimentin OS=Rattus norvegicus GN=Vim PE=1 SV=2 - [VIME_RAT]	53.7	0.894	0.804	0.529	1.381	1.026	0.013	0.013	0.013	0.013	0.013	0.013	0.013	0.013	0.013	0.013	1.988	0.723	1.434	1.739	0.952	0.009	0.073	0.009	0.194	0.184	0.210	#DIV/0!	#DIV/0!	0.009	0.042	0.009	0.110
P41565	Isocitrate dehydrogenase [NAD] subunit gamma 1, mitochondrial OS=Rattus norvegicus GN=Idh3g PE=2 SV=2 - [IDHG1_RAT]	42.8	0.940	0.738	0.685	1.353	0.948	0.013	0.013	0.013	0.013	0.013	0.679	0.789	1.097	4.259	0.800	5.365	0.479	1.473	1.522	0.514	0.009	0.073	0.917	0.748	0.605	0.532	0.009	0.042	0.009	0.024	0.917	0.978
P43424	Galactose-1-phosphate uridylyltransferase OS=Rattus norvegicus GN=Galt PE=2 SV=1 - [GALT_RAT]	43.3	0.013	0.013	0.013	0.013	0.013	1.082	0.865	1.086	0.700	0.855	0.013	0.013	0.013	0.013	0.013	0.013	0.013	0.013	0.013	0.013	0.009	0.078	#DIV/0!	#DIV/0!	#DIV/0!	#DIV/0!	0.009	0.157	0.009	0.124	#DIV/0!	#DIV/0!
P52632	Signal transducer and activator of transcription 5B OS=Rattus norvegicus GN=Stat5b PE=1 SV=1 - [STA5B_RAT]	90.2	0.762	1.133	0.764	1.201	1.273	0.013	0.013	0.013	0.013	0.013	1.128	1.069	2.029	1.448	1.198	1.251	1.024	1.572	1.033	0.790	0.009	0.059	0.354	0.339	0.605	0.541	0.009	0.052	0.009	0.062	0.259	1.336
P62161	Calmodulin OS=Rattus norvegicus GN=Calm1 PE=1 SV=2 - [CALM_RAT]	16.8	0.358	1.272	0.278	1.216	1.363	0.013	0.013	0.013	0.013	0.013	1.148	1.036	2.934	1.523	0.990	1.287	0.310	1.289	1.190	1.217	0.009	0.092	0.470	0.425	0.756	0.661	0.009	0.041	0.009	0.074	0.917	1.194
P62255	Ubiquitin-conjugating enzyme E2 G1 OS=Rattus norvegicus GN=Ube2g1 PE=2 SV=3 - [UB2G1_RAT]	19.5	0.859	0.964	0.619	0.993	1.047	0.013	0.013	0.013	0.013	0.013	0.013	0.013	0.013	0.013	0.013	0.013	0.013	0.013	0.013	0.013	0.009	0.093	0.009	0.261	0.009	0.197	#DIV/0!	#DIV/0!	#DIV/0!	#DIV/0!	#DIV/0!	#DIV/0!
P63045	Vesicle-associated membrane protein 2 OS=Rattus norvegicus GN=Vamp2 PE=1 SV=2 - [VAMP2_RAT]	12.7	1.012	0.986	0.560	1.052	0.909	0.013	0.013	0.013	0.013	0.013	0.013	0.013	0.013	0.013	0.013	1.038	0.887	1.770	1.969	0.969	0.009	0.088	0.009	0.238	0.354	0.348	#DIV/0!	#DIV/0!	0.009	0.044	0.009	0.120
P67779	Prohibitin OS=Rattus norvegicus OX=10116 GN=Phb PE=1 SV=1 - [PHB_RAT]	29.8	0.812	1.080	0.446	1.121	1.113	0.013	0.013	0.013	0.013	0.013	0.013	0.013	0.013	0.013	0.013	1.001	1.127	0.977	1.101	1.335	0.009	0.080	0.009	0.213	0.354	0.361	#DIV/0!	#DIV/0!	0.009	0.064	0.009	0.202
P69682	Adaptin ear-binding coat-associated protein 1 OS=Rattus norvegicus GN=Necap1 PE=1 SV=1 - [NECP1_RAT]	29.8	0.954	0.880	0.705	1.129	1.095	0.013	0.013	0.013	0.013	0.013	0.013	0.013	0.013	0.013	0.013	1.146	1.186	0.959	1.010	0.859	0.009	0.069	0.009	0.180	0.354	0.362	#DIV/0!	#DIV/0!	0.009	0.079	0.009	0.281
P70531	Eukaryotic elongation factor 2 kinase OS=Rattus norvegicus GN=Eef2k PE=1 SV=1 - [EF2K_RAT]	81.4	0.712	0.986	0.536	1.180	1.115	0.013	0.013	0.013	0.013	0.013	0.013	0.013	0.013	0.013	0.013	0.013	0.013	0.013	0.013	0.013	0.009	0.085	0.009	0.232	0.009	0.180	#DIV/0!	#DIV/0!	#DIV/0!	#DIV/0!	#DIV/0!	#DIV/0!
P70541	Translation initiation factor eIF-2B subunit gamma OS=Rattus norvegicus GN=Eif2b3 PE=2 SV=2 - [EI2BG_RAT]	50.4	0.013	0.013	0.013	0.013	0.013	1.795	0.763	0.930	1.050	0.928	1.157	1.140	1.788	0.862	0.852	0.688	1.016	1.325	1.287	0.453	0.009	0.054	0.009	0.080	0.009	0.137	0.756	0.547	0.756	0.544	0.605	1.733
P85834	Elongation factor Tu, mitochondrial OS=Rattus norvegicus GN=Tufm PE=1 SV=1 - [EFTU_RAT]	49.5	1.577	0.414	1.192	0.728	0.589	0.013	0.013	0.013	0.013	0.013	0.480	0.598	0.901	3.496	0.469	3.106	0.591	1.254	1.031	0.402	0.009	0.090	0.917	0.763	0.756	0.651	0.009	0.066	0.009	0.048	0.756	1.430
P97592	Mast cell protease 4 OS=Rattus norvegicus OX=10116 GN=Mcpt4 PE=1 SV=1 - [MCPT4_RAT]	27.0	1.358	0.793	1.100	0.818	0.705	0.013	0.013	0.013	0.013	0.013	0.607	0.628	1.133	0.244	0.599	0.263	1.069	0.580	0.780	0.515	0.009	0.068	0.085	0.116	0.085	0.134	0.009	1.820	0.009	1.261	0.756	1.611
P97779	Hyaluronan-mediated motility receptor OS=Rattus norvegicus OX=10116 GN=Hmmr PE=2 SV=1 - [HMMR_RAT]	57.8	1.235	0.549	1.213	1.156	0.988	0.013	0.013	0.013	0.013	0.013	0.013	0.013	0.013	0.013	0.013	0.013	0.013	0.013	0.013	0.013	0.009	0.058	0.009	0.117	0.009	0.099	#DIV/0!	#DIV/0!	#DIV/0!	#DIV/0!	#DIV/0!	#DIV/0!
Q07009	Calpain-2 catalytic subunit OS=Rattus norvegicus GN=Capn2 PE=1 SV=3 - [CAN2_RAT]	79.9	1.051	0.756	0.764	1.107	1.043	0.013	0.013	0.013	0.013	0.013	1.216	1.289	2.608	2.778	0.957	2.853	0.487	1.778	1.510	1.254	0.009	0.070	0.056	0.077	0.127	0.158	0.009	0.030	0.009	0.029	0.917	0.985
Q08013	Translocon-associated protein subunit gamma OS=Rattus norvegicus OX=10116 GN=Ssr3 PE=1 SV=2 - [SSRG_RAT]	21.1	0.792	1.284	0.608	1.239	1.092	0.013	0.013	0.013	0.013	0.013	0.013	0.013	0.013	0.013	0.013	0.013	0.013	0.013	0.013	0.013	0.009	0.062	0.009	0.141	0.009	0.113	#DIV/0!	#DIV/0!	#DIV/0!	#DIV/0!	#DIV/0!	#DIV/0!
Q09030	Trefoil factor 2 OS=Rattus norvegicus OX=10116 GN=Tff2 PE=2 SV=1 - [TFF2_RAT]	14.1	1.344	0.993	0.955	1.323	0.928	0.013	0.013	0.013	0.013	0.013	0.013	0.013	0.013	0.013	0.013	0.013	0.013	0.013	0.013	0.013	0.009	0.054	0.009	0.090	0.009	0.077	#DIV/0!	#DIV/0!	#DIV/0!	#DIV/0!	#DIV/0!	#DIV/0!
Q09326	Alpha-1,6-mannosyl-glycoprotein 2-beta-N-acetylglucosaminyltransferase OS=Rattus norvegicus GN=Mgat2 PE=1 SV=1 - [MGAT2_RAT]	51.1	1.143	0.890	0.733	1.231	1.004	0.013	0.013	0.013	0.013	0.013	0.013	0.013	0.013	0.013	0.013	0.013	0.013	0.013	0.013	0.013	0.009	0.063	0.009	0.145	0.009	0.114	#DIV/0!	#DIV/0!	#DIV/0!	#DIV/0!	#DIV/0!	#DIV/0!
Q3B7D1	Ubiquitin-conjugating enzyme E2 Z OS=Rattus norvegicus GN=Ube2z PE=2 SV=2 - [UBE2Z_RAT]	38.3	0.013	0.013	0.013	0.013	0.013	1.537	0.707	0.829	0.742	0.706	0.679	0.796	1.868	0.913	1.323	1.356	0.659	1.300	1.228	1.554	0.009	0.086	0.009	0.086	0.009	0.064	0.470	0.357	0.354	0.277	0.756	1.482
Q4KLF8	Actin-related protein 2/3 complex subunit 5 OS=Rattus norvegicus GN=Arpc5 PE=1 SV=3 - [ARPC5_RAT]	16.3	0.889	0.856	0.687	1.084	0.936	0.013	0.013	0.013	0.013	0.013	0.867	0.853	1.795	1.172	0.957	0.013	0.013	0.013	0.013	0.013	0.009	0.098	0.354	0.352	0.009	0.209	0.009	0.074	#DIV/0!	#DIV/0!	0.009	0.184
Q4KLN8	E3 ubiquitin-protein ligase RNF8 OS=Rattus norvegicus OX=10116 GN=Rnf8 PE=2 SV=1 - [RNF8_RAT]	55.6	1.009	1.245	1.049	1.686	1.153	0.013	0.013	0.013	0.013	0.013	0.013	0.013	0.013	0.013	0.013	0.013	0.013	0.013	0.013	0.013	0.009	0.052	0.009	0.071	0.009	0.063	#DIV/0!	#DIV/0!	#DIV/0!	#DIV/0!	#DIV/0!	#DIV/0!
Q561S0	NADH dehydrogenase [ubiquinone] 1 alpha subcomplex subunit 10, mitochondrial OS=Rattus norvegicus GN=Ndufa10 PE=1 SV=1 - [NDUAA_RAT]	40.5	0.493	1.334	0.379	1.730	1.290	0.013	0.013	0.013	0.013	0.013	1.373	1.940	3.034	4.150	1.004	2.160	0.853	1.636	1.726	0.624	0.009	0.057	0.085	0.105	0.354	0.342	0.009	0.023	0.009	0.039	0.259	1.256
Q5EGY4	Synaptobrevin homolog YKT6 OS=Rattus norvegicus GN=Ykt6 PE=1 SV=1 - [YKT6_RAT]	22.4	0.480	1.084	0.395	1.342	1.225	0.013	0.013	0.013	0.013	0.013	1.263	1.196	2.582	2.280	0.982	2.241	0.590	1.497	1.476	1.259	0.009	0.086	0.184	0.196	0.085	0.121	0.009	0.034	0.009	0.038	0.756	1.215
Q5HZE4	Methylthioribose-1-phosphate isomerase OS=Rattus norvegicus GN=Mri1 PE=2 SV=1 - [MTNA_RAT]	39.6	1.181	0.889	0.570	0.927	0.881	0.013	0.013	0.013	0.013	0.013	1.364	1.586	1.996	1.578	1.121	1.708	0.780	2.314	1.386	0.937	0.009	0.099	0.022	0.041	0.127	0.162	0.009	0.041	0.009	0.036	0.756	1.245
Q5M7W5	Microtubule-associated protein 4 OS=Rattus norvegicus GN=Map4 PE=1 SV=1 - [MAP4_RAT]	110.2	0.013	0.013	0.013	0.013	0.013	1.394	1.142	1.036	0.753	0.711	0.013	0.013	0.013	0.013	0.013	0.013	0.013	0.013	0.013	0.013	0.009	0.062	#DIV/0!	#DIV/0!	#DIV/0!	#DIV/0!	0.009	0.115	0.009	0.090	#DIV/0!	#DIV/0!
Q5U312	Ankycorbin OS=Rattus norvegicus OX=10116 GN=Rai14 PE=1 SV=2 - [RAI14_RAT]	109.1	0.013	0.013	0.013	0.013	0.013	1.399	2.571	1.106	0.361	0.409	0.013	0.013	0.013	0.013	0.013	0.013	0.013	0.013	0.013	0.013	0.009	0.053	#DIV/0!	#DIV/0!	#DIV/0!	#DIV/0!	0.009	0.070	0.009	0.058	#DIV/0!	#DIV/0!
Q5U316	Ras-related protein Rab-35 OS=Rattus norvegicus GN=Rab35 PE=1 SV=1 - [RAB35_RAT]	23.0	1.259	0.771	0.775	1.064	1.056	0.013	0.013	0.013	0.013	0.013	1.528	1.332	2.297	2.742	1.092	2.585	0.851	1.867	1.899	1.774	0.009	0.065	0.022	0.039	0.056	0.088	0.009	0.029	0.009	0.025	0.917	0.952
Q5U3Y7	Sigma intracellular receptor 2 OS=Rattus norvegicus OX=10116 GN=Tmem97 PE=2 SV=1 - [SGMR2_RAT]	20.9	0.013	0.013	0.013	0.013	0.013	1.333	0.436	0.749	1.572	0.997	1.070	0.940	2.397	0.160	1.236	0.013	0.013	0.013	0.013	0.013	0.009	0.060	0.009	0.079	#DIV/0!	#DIV/0!	0.917	0.656	0.009	0.087	0.009	0.165
Q5XIH7	Prohibitin-2 OS=Rattus norvegicus OX=10116 GN=Phb2 PE=1 SV=1 - [PHB2_RAT]	33.3	0.636	1.028	0.537	1.349	1.045	0.013	0.013	0.013	0.013	0.013	0.013	0.013	0.013	0.013	0.013	0.013	0.013	0.013	0.013	0.013	0.009	0.076	0.009	0.205	0.009	0.160	#DIV/0!	#DIV/0!	#DIV/0!	#DIV/0!	#DIV/0!	#DIV/0!
Q5XIP1	Protein pelota homolog OS=Rattus norvegicus GN=Pelo PE=1 SV=1 - [PELO_RAT]	43.4	1.888	0.978	1.578	1.082	1.014	0.013	0.013	0.013	0.013	0.013	0.013	0.013	0.013	0.013	0.013	0.013	0.013	0.013	0.013	0.013	0.009	0.051	0.009	0.064	0.009	0.057	#DIV/0!	#DIV/0!	#DIV/0!	#DIV/0!	#DIV/0!	#DIV/0!
Q5XIS9	Serine/threonine-protein kinase D2 OS=Rattus norvegicus OX=10116 GN=Prkd2 PE=1 SV=1 - [KPCD2_RAT]	96.4	0.013	0.013	0.013	0.013	0.013	1.058	1.886	0.755	0.717	1.985	0.013	0.013	0.013	0.013	0.013	1.459	0.864	0.926	0.704	0.927	0.009	0.051	#DIV/0!	#DIV/0!	0.009	0.128	0.009	0.059	0.470	0.351	0.009	0.359
Q5XIU9	Membrane-associated progesterone receptor component 2 OS=Rattus norvegicus OX=10116 GN=Pgrmc2 PE=1 SV=1 - [PGRC2_RAT]	23.4	1.434	0.708	0.769	1.016	0.758	0.013	0.013	0.013	0.013	0.013	0.940	0.780	1.603	1.419	0.626	0.998	0.655	1.579	1.148	0.329	0.009	0.072	0.605	0.541	0.917	0.779	0.009	0.086	0.009	0.115	0.756	1.585
Q62745	CD81 antigen OS=Rattus norvegicus GN=Cd81 PE=1 SV=1 - [CD81_RAT]	25.9	1.048	0.780	0.763	1.046	0.914	0.013	0.013	0.013	0.013	0.013	0.845	0.693	1.426	2.179	0.728	2.343	0.762	1.573	1.371	0.802	0.009	0.083	0.917	0.763	0.354	0.346	0.009	0.069	0.009	0.041	0.470	1.602
Q63065	[Pyruvate dehydrogenase (acetyl-transferring)] kinase isozyme 1, mitochondrial OS=Rattus norvegicus OX=10116 GN=Pdk1 PE=1 SV=1 - [PDK1_RAT]	49.0	0.868	1.145	0.670	1.124	1.087	0.013	0.013	0.013	0.013	0.013	0.013	0.013	0.013	0.013	0.013	1.505	0.941	1.546	1.388	1.139	0.009	0.066	0.009	0.158	0.085	0.122	#DIV/0!	#DIV/0!	0.009	0.045	0.009	0.127
Q63377	Sodium/potassium-transporting ATPase subunit beta-3 OS=Rattus norvegicus GN=Atp1b3 PE=2 SV=1 - [AT1B3_RAT]	31.8	1.029	1.055	0.540	1.079	0.813	0.013	0.013	0.013	0.013	0.013	1.315	1.259	1.932	2.107	0.969	1.437	0.743	1.761	1.352	0.613	0.009	0.088	0.056	0.079	0.354	0.358	0.009	0.043	0.009	0.056	0.470	1.548
Q63450	Calcium/calmodulin-dependent protein kinase type 1 OS=Rattus norvegicus GN=Camk1 PE=1 SV=2 - [KCC1A_RAT]	41.6	0.013	0.013	0.013	0.013	0.013	1.792	0.696	0.900	1.058	0.911	0.013	0.013	0.013	0.013	0.013	0.013	0.013	0.013	0.013	0.013	0.009	0.056	#DIV/0!	#DIV/0!	#DIV/0!	#DIV/0!	0.009	0.088	0.009	0.071	#DIV/0!	#DIV/0!
Q64119	Myosin light polypeptide 6 OS=Rattus norvegicus GN=Myl6 PE=1 SV=3 - [MYL6_RAT]	17.0	0.634	1.173	0.411	1.325	1.133	0.013	0.013	0.013	0.013	0.013	1.872	1.122	2.870	2.466	0.858	2.382	0.606	1.455	2.096	1.169	0.009	0.072	0.184	0.195	0.184	0.205	0.009	0.028	0.009	0.031	0.605	1.322
Q66HA6	ADP-ribosylation factor-like protein 8B OS=Rattus norvegicus GN=Arl8b PE=2 SV=1 - [ARL8B_RAT]	21.5	0.884	1.036	0.458	1.452	1.040	0.013	0.013	0.013	0.013	0.013	1.676	1.029	2.429	3.403	0.863	2.344	0.569	1.238	1.249	0.708	0.009	0.066	0.259	0.259	0.605	0.539	0.009	0.027	0.009	0.052	0.259	1.286
Q66HG9	Mitochondrial antiviral-signaling protein OS=Rattus norvegicus OX=10116 GN=Mavs PE=1 SV=1 - [MAVS_RAT]	53.8	1.006	0.768	0.619	1.192	0.974	0.013	0.013	0.013	0.013	0.013	1.339	1.150	2.668	2.302	1.398	1.591	0.636	2.036	2.044	1.410	0.009	0.082	0.022	0.040	0.085	0.118	0.009	0.030	0.009	0.031	0.917	0.991
Q6AXM7	HBS1-like protein OS=Rattus norvegicus GN=Hbs1l PE=2 SV=1 - [HBS1L_RAT]	74.7	0.680	1.166	0.452	1.222	0.960	0.013	0.013	0.013	0.013	0.013	1.577	1.829	2.959	3.536	1.077	1.600	0.715	2.109	1.816	1.046	0.009	0.094	0.036	0.054	0.127	0.160	0.009	0.023	0.009	0.035	0.259	1.260
Q6AY43	Cytoplasmic dynein 2 light intermediate chain 1 OS=Rattus norvegicus OX=10116 GN=Dync2li1 PE=1 SV=2 - [DC2L1_RAT]	39.6	0.013	0.013	0.013	0.013	0.013	1.418	0.673	1.104	0.555	0.783	0.013	0.013	0.013	0.013	0.013	0.013	0.013	0.013	0.013	0.013	0.009	0.084	#DIV/0!	#DIV/0!	#DIV/0!	#DIV/0!	0.009	0.172	0.009	0.134	#DIV/0!	#DIV/0!
Q6AY56	Tubulin alpha-8 chain OS=Rattus norvegicus GN=Tuba8 PE=2 SV=1 - [TBA8_RAT]	50.0	0.013	0.013	0.013	0.013	0.013	1.247	0.731	1.272	0.594	0.768	0.013	0.013	0.013	0.013	0.013	0.162	1.189	1.167	1.028	0.951	0.009	0.075	#DIV/0!	#DIV/0!	0.009	0.190	0.009	0.152	0.917	0.654	0.009	0.561
Q6E0V2	Katanin p60 ATPase-containing subunit A1 OS=Rattus norvegicus OX=10116 GN=Katna1 PE=1 SV=1 - [KTNA1_RAT]	55.8	1.560	0.537	1.412	1.021	1.068	0.013	0.013	0.013	0.013	0.013	0.013	0.013	0.013	0.013	0.013	0.529	2.060	0.446	0.561	0.480	0.009	0.054	0.009	0.086	0.184	0.225	#DIV/0!	#DIV/0!	0.009	0.315	0.009	1.173
Q6IFU7	Keratin, type I cytoskeletal 42 OS=Rattus norvegicus GN=Krt42 PE=3 SV=1 - [K1C42_RAT]	50.2	0.013	0.013	0.013	0.013	0.013	0.926	0.744	0.652	1.008	2.273	0.013	0.013	0.013	0.013	0.013	0.013	0.013	0.013	0.013	0.013	0.009	0.053	#DIV/0!	#DIV/0!	#DIV/0!	#DIV/0!	0.009	0.076	0.009	0.064	#DIV/0!	#DIV/0!
Q6IFU8	Keratin, type I cytoskeletal 17 OS=Rattus norvegicus GN=Krt17 PE=2 SV=1 - [K1C17_RAT]	48.1	0.013	0.013	0.013	0.013	0.013	0.813	0.491	0.719	1.075	3.344	0.013	0.013	0.013	0.013	0.013	0.013	0.013	0.013	0.013	0.013	0.009	0.051	#DIV/0!	#DIV/0!	#DIV/0!	#DIV/0!	0.009	0.058	0.009	0.047	#DIV/0!	#DIV/0!
Q6IG02	Keratin, type II cytoskeletal 2 epidermal OS=Rattus norvegicus GN=Krt2 PE=3 SV=1 - [K22E_RAT]	69.1	1.345	0.696	0.726	1.125	1.301	0.013	0.013	0.013	0.013	0.013	0.974	1.049	1.783	0.935	0.920	0.013	0.013	0.013	0.013	0.013	0.009	0.058	0.917	0.757	0.009	0.092	0.009	0.073	#DIV/0!	#DIV/0!	0.009	0.177
Q6IRE4	Tumor susceptibility gene 101 protein OS=Rattus norvegicus GN=Tsg101 PE=1 SV=1 - [TS101_RAT]	44.0	0.796	1.161	0.525	1.053	0.961	0.013	0.013	0.013	0.013	0.013	0.013	0.013	0.013	0.013	0.013	1.734	0.624	1.710	1.790	1.270	0.009	0.091	0.009	0.249	0.085	0.120	#DIV/0!	#DIV/0!	0.009	0.036	0.009	0.1001
Q6IUP3	Elongator complex protein 5 OS=Rattus norvegicus GN=Elp5 PE=2 SV=1 - [ELP5_RAT]	35.5	0.013	0.013	0.013	0.013	0.013	1.787	0.516	1.872	0.404	0.631	0.013	0.013	0.013	0.013	0.013	0.013	0.013	0.013	0.013	0.013	0.009	0.058	#DIV/0!	#DIV/0!	#DIV/0!	#DIV/0!	0.009	0.097	0.009	0.077	#DIV/0!	#DIV/0!
Q6P6Q2	Keratin, type II cytoskeletal 5 OS=Rattus norvegicus GN=Krt5 PE=1 SV=1 - [K2C5_RAT]	61.8	0.013	0.013	0.013	0.013	0.013	1.225	0.512	0.662	0.887	1.494	0.013	0.013	0.013	0.013	0.013	0.013	0.013	0.013	0.013	0.013	0.009	0.068	#DIV/0!	#DIV/0!	#DIV/0!	#DIV/0!	0.009	0.132	0.009	0.108	#DIV/0!	#DIV/0!
Q6P9T8	Tubulin beta-4B chain OS=Rattus norvegicus GN=Tubb4b PE=1 SV=1 - [TBB4B_RAT]	49.8	0.619	1.130	0.591	1.288	1.066	0.013	0.013	0.013	0.013	0.013	1.395	1.411	2.321	2.824	1.110	2.074	0.580	1.888	1.301	0.863	0.009	0.071	0.036	0.054	0.354	0.346	0.009	0.028	0.009	0.044	0.259	1.281
Q6QI44	N-alpha-acetyltransferase 25, NatB auxiliary subunit OS=Rattus norvegicus GN=Naa25 PE=2 SV=1 - [NAA25_RAT]	111.2	0.013	0.013	0.013	0.013	0.013	1.658	0.730	0.920	0.686	0.621	0.896	1.106	1.498	1.634	0.936	1.381	1.503	2.064	2.107	1.158	0.009	0.075	0.009	0.075	0.009	0.036	0.184	0.160	0.056	0.073	0.127	0.761
Q6TUG0	DnaJ homolog subfamily B member 11 OS=Rattus norvegicus GN=Dnajb11 PE=2 SV=1 - [DJB11_RAT]	40.5	1.320	0.754	1.288	0.925	0.849	0.013	0.013	0.013	0.013	0.013	0.687	0.755	1.651	1.462	0.952	1.403	1.102	1.402	0.800	0.771	0.009	0.059	0.756	0.651	0.605	0.542	0.009	0.081	0.009	0.066	0.917	1.292
Q6VV72	Eukaryotic translation initiation factor 1A OS=Rattus norvegicus OX=10116 GN=Eif1a PE=2 SV=3 - [IF1A_RAT]	16.5	1.425	0.439	0.926	0.915	0.815	0.013	0.013	0.013	0.013	0.013	0.437	0.445	0.916	0.721	0.618	0.013	0.013	0.013	0.013	0.013	0.009	0.087	0.184	0.210	0.009	0.183	0.009	3.034	#DIV/0!	#DIV/0!	0.009	1.844
Q704E8	ATP-binding cassette sub-family B member 7, mitochondrial OS=Rattus norvegicus OX=10116 GN=Abcb7 PE=1 SV=1 - [ABCB7_RAT]	82.5	0.013	0.013	0.013	0.013	0.013	1.731	0.925	0.863	0.941	0.957	0.013	0.013	0.013	0.013	0.013	0.013	0.013	0.013	0.013	0.013	0.009	0.055	#DIV/0!	#DIV/0!	#DIV/0!	#DIV/0!	0.009	0.085	0.009	0.068	#DIV/0!	#DIV/0!
Q71UE8	NEDD8 OS=Rattus norvegicus OX=10116 GN=Nedd8 PE=1 SV=1 - [NEDD8_RAT]	9.0	1.094	0.770	0.885	1.436	0.900	0.013	0.013	0.013	0.013	0.013	0.013	0.013	0.013	0.013	0.013	0.013	0.013	0.013	0.013	0.013	0.009	0.060	0.009	0.126	0.009	0.104	#DIV/0!	#DIV/0!	#DIV/0!	#DIV/0!	#DIV/0!	#DIV/0!
Q78P75	Dynein light chain 2, cytoplasmic OS=Rattus norvegicus GN=Dynll2 PE=1 SV=1 - [DYL2_RAT]	10.3	0.693	1.186	0.424	1.333	0.926	0.013	0.013	0.013	0.013	0.013	1.030	0.815	1.832	1.594	0.827	3.109	0.602	1.421	1.910	0.999	0.009	0.082	0.354	0.347	0.184	0.205	0.009	0.064	0.009	0.029	0.605	1.447
Q7TT51	Lengsin OS=Rattus norvegicus OX=10116 GN=Lgsn PE=2 SV=1 - [LGSN_RAT]	62.2	1.174	0.949	0.644	1.201	1.262	0.013	0.013	0.013	0.013	0.013	0.013	0.013	0.013	0.013	0.013	0.013	0.013	0.013	0.013	0.013	0.009	0.057	0.009	0.107	0.009	0.091	#DIV/0!	#DIV/0!	#DIV/0!	#DIV/0!	#DIV/0!	#DIV/0!
Q8CGS5	Zinc phosphodiesterase ELAC protein 2 OS=Rattus norvegicus GN=Elac2 PE=1 SV=1 - [RNZ2_RAT]	92.3	1.479	1.591	0.583	2.344	1.907	0.013	0.013	0.013	0.013	0.013	1.067	0.897	1.957	1.534	1.040	0.013	0.013	0.013	0.013	0.013	0.009	0.051	0.470	0.423	0.009	0.038	0.009	0.057	#DIV/0!	#DIV/0!	0.009	0.129
Q921A4	Cytoglobin OS=Rattus norvegicus OX=10116 GN=Cygb PE=1 SV=1 - [CYGB_RAT]	21.5	0.791	0.844	0.722	1.048	0.977	0.564	0.386	0.554	0.606	0.645	0.959	0.800	1.672	2.969	0.966	3.902	0.638	1.141	1.525	1.180	0.014	0.077	0.259	0.264	0.127	0.158	0.014	0.032	0.022	0.050	0.756	1.201
Q924S5	Lon protease homolog, mitochondrial OS=Rattus norvegicus GN=Lonp1 PE=2 SV=1 - [LONM_RAT]	105.7	1.080	0.813	0.821	0.981	0.927	0.013	0.013	0.013	0.013	0.013	1.273	1.200	1.823	2.337	0.973	3.089	0.656	2.394	1.674	0.902	0.009	0.074	0.036	0.057	0.354	0.341	0.009	0.042	0.009	0.025	0.917	1.002
Q9EQH5	C-terminal-binding protein 2 OS=Rattus norvegicus GN=Ctbp2 PE=1 SV=2 - [CTBP2_RAT]	49.0	1.093	0.781	0.787	1.096	1.156	0.013	0.013	0.013	0.013	0.013	0.609	0.573	1.153	1.278	0.588	0.013	0.013	0.013	0.013	0.013	0.009	0.065	0.470	0.444	0.009	0.121	0.009	0.325	#DIV/0!	#DIV/0!	0.009	1.076
Q9ES40	PRA1 family protein 3 OS=Rattus norvegicus OX=10116 GN=Arl6ip5 PE=1 SV=1 - [PRAF3_RAT]	21.5	0.013	0.013	0.013	0.013	0.013	1.752	0.658	0.556	0.803	0.733	1.508	1.564	2.569	1.332	1.023	1.665	0.564	1.871	1.721	0.790	0.009	0.089	0.009	0.046	0.009	0.056	0.085	0.086	0.354	0.276	0.917	1.075
Q9JHB5	Translin-associated protein X OS=Rattus norvegicus GN=Tsnax PE=1 SV=1 - [TSNAX_RAT]	33.0	0.013	0.013	0.013	0.013	0.013	1.304	0.917	0.775	0.948	1.008	0.744	0.889	1.677	1.012	1.032	1.338	0.875	1.362	0.958	1.516	0.009	0.063	0.009	0.0995	0.009	0.065	0.756	0.551	0.184	0.163	0.605	1.673
Q9JIX3	Bis(5'-adenosyl)-triphosphatase OS=Rattus norvegicus OX=10116 GN=Fhit PE=1 SV=1 - [FHIT_RAT]	17.3	0.013	0.013	0.013	0.013	0.013	1.132	0.559	0.768	1.140	1.003	0.931	0.998	1.856	1.720	0.813	2.034	1.054	1.293	1.034	1.197	0.009	0.076	0.009	0.069	0.009	0.056	0.470	0.354	0.085	0.097	0.354	1.537
Q9JLH7	CDK5 regulatory subunit-associated protein 3 OS=Rattus norvegicus OX=10116 GN=Cdk5rap3 PE=1 SV=1 - [CK5P3_RAT]	57.0	1.619	0.866	0.704	1.349	1.082	0.013	0.013	0.013	0.013	0.013	1.017	1.207	2.173	1.896	1.206	2.183	1.024	1.985	1.286	1.229	0.009	0.053	0.259	0.260	0.259	0.270	0.009	0.043	0.009	0.031	0.470	1.440
Q9QUL6	Vesicle-fusing ATPase OS=Rattus norvegicus GN=Nsf PE=1 SV=1 - [NSF_RAT]	82.6	1.606	0.939	1.002	1.312	1.048	0.013	0.013	0.013	0.013	0.013	0.013	0.013	0.013	0.013	0.013	2.238	0.013	3.671	1.577	1.981	0.009	0.052	0.009	0.077	0.184	0.204	#DIV/0!	#DIV/0!	0.025	0.055	0.025	0.161
Q9QXT4	Probable N-acetyltransferase CML1 OS=Rattus norvegicus GN=Cml1 PE=2 SV=1 - [CMLO1_RAT]	24.8	0.701	1.271	0.267	1.399	1.356	0.013	0.013	0.013	0.013	0.013	0.013	0.013	0.013	0.013	0.013	0.013	0.013	0.013	0.013	0.013	0.009	0.063	0.009	0.147	0.009	0.117	#DIV/0!	#DIV/0!	#DIV/0!	#DIV/0!	#DIV/0!	#DIV/0!
Q9WV59	E3 ubiquitin-protein ligase TRIM17 OS=Rattus norvegicus GN=Trim17 PE=2 SV=1 - [TRI17_RAT]	54.9	2.344	0.596	1.581	1.056	0.803	0.013	0.013	0.013	0.013	0.013	0.013	0.013	0.013	0.013	0.013	0.013	0.013	0.013	0.013	0.013	0.009	0.052	0.009	0.066	0.009	0.059	#DIV/0!	#DIV/0!	#DIV/0!	#DIV/0!	#DIV/0!	#DIV/0!
Q9Z1X1	Extended synaptotagmin-1 OS=Rattus norvegicus GN=Esyt1 PE=1 SV=1 - [ESYT1_RAT]	121.1	0.013	0.013	0.013	0.013	0.013	1.072	0.546	0.620	1.197	1.132	0.013	0.013	0.013	0.013	0.013	0.013	0.013	0.013	0.013	0.013	0.009	0.081	#DIV/0!	#DIV/0!	#DIV/0!	#DIV/0!	0.009	0.165	0.009	0.132	#DIV/0!	#DIV/0!
Q9Z2J4	Nexilin OS=Rattus norvegicus GN=Nexn PE=1 SV=1 - [NEXN_RAT]	78.3	0.013	0.013	0.013	0.013	0.013	1.134	0.680	0.658	1.273	1.361	0.013	0.013	0.013	0.013	0.013	0.013	0.013	0.013	0.013	0.013	0.009	0.059	#DIV/0!	#DIV/0!	#DIV/0!	#DIV/0!	0.009	0.107	0.009	0.083	#DIV/0!	#DIV/0!

Table S11. Extracted protein profile of c-III (Effect of Nutrition, only Sham, Male)

Accession	Description	MW [kDa]	Expression level																				p<0.05, q<0.10											
			FGR-Good-Male					FGR-Poor-Male					Sham-Good-Male					Sham-Poor-Male					Steel-Dwass & FDR											
			#21	#22	#23	#24	#25	#26	#27	#28	#29	#30	#31	#32	#33	#34	#35	#36	#37	#38	#39	#40	FGR-GN vs FGR-PN		FGR-GN vs Sham-GN		FGR-GN vs Sham-PN		FGR-PN vs Sham-GN		FGR-PN vs Sham-PN		Sham-GN vs Sham-PN	
Filter	Filter	Filter	Filter	Filter	Filter	Filter	Filter	Filter	Filter	Filter	Filter	Filter	Filter	Filter	Filter	Filter	Filter	Filter	Filter	Filter	Filter	Filter	p-value	q-value	p	q-value	p	q-value	p	q-value	p	q-value	p	q-value
A0JPM9	Eukaryotic translation initiation factor 3 subunit J OS=Rattus norvegicus OX=10116 GN=Eif3j PE=1 SV=1 - [EIF3J_RAT]	29.2	0.852	0.817	1.827	0.715	0.795	0.919	0.923	1.260	0.247	0.613	0.960	0.989	0.953	1.796	0.562	0.013	0.013	0.013	0.013	0.013	0.917	0.563	0.470	0.511	0.009	0.049	0.259	0.535	0.009	0.032	0.009	0.040
A0JPQ1	Uncharacterized protein C7orf57 homolog OS=Rattus norvegicus OX=10116 PE=2 SV=1 - [CG057_RAT]	32.7	0.013	0.013	0.013	0.013	0.013	0.013	0.013	0.013	0.013	0.013	0.013	0.013	0.013	0.013	0.013	0.591	1.285	1.592	1.210	0.821	#DIV/0!	#DIV/0!	#DIV/0!	#DIV/0!	0.009	0.045	#DIV/0!	#DIV/0!	0.009	0.016	0.009	0.037
A0MZ67	Shootin-1 OS=Rattus norvegicus OX=10116 GN=Shtn1 PE=1 SV=1 - [SHOT1_RAT]	71.4	0.013	0.013	0.013	0.013	0.013	0.013	0.013	0.013	0.013	0.013	0.979	1.099	0.989	1.394	0.628	0.013	0.013	0.013	0.013	0.013	#DIV/0!	#DIV/0!	0.009	0.030	#DIV/0!	#DIV/0!	0.009	0.047	#DIV/0!	#DIV/0!	0.009	0.043
B2GUY0	Endoplasmic reticulum mannosyl-oligosaccharide 1,2-alpha-mannosidase OS=Rattus norvegicus OX=10116 GN=Man1b1 PE=2 SV=2 - [MA1B1_RAT]	74.8	0.013	0.013	0.013	0.013	0.013	0.013	0.013	0.013	0.013	0.013	0.013	0.013	0.013	0.013	0.013	0.785	0.893	1.915	0.737	1.012	#DIV/0!	#DIV/0!	#DIV/0!	#DIV/0!	0.009	0.046	#DIV/0!	#DIV/0!	0.009	0.017	0.009	0.039
B2GV24	E3 UFM1-protein ligase 1 OS=Rattus norvegicus GN=Ufl1 PE=1 SV=1 - [UFL1_RAT]	89.5	0.013	0.013	0.013	0.013	0.013	0.013	0.013	0.013	0.013	0.013	1.311	1.013	0.901	1.072	0.808	0.013	0.013	0.013	0.013	0.013	#DIV/0!	#DIV/0!	0.009	0.030	#DIV/0!	#DIV/0!	0.009	0.046	#DIV/0!	#DIV/0!	0.009	0.043
B2RYG6	Ubiquitin thioesterase OTUB1 OS=Rattus norvegicus GN=Otub1 PE=1 SV=1 - [OTUB1_RAT]	31.3	0.852	0.778	1.493	0.504	0.541	0.806	0.927	1.135	0.134	0.734	0.621	0.945	1.105	1.305	0.296	0.013	0.013	0.013	0.013	0.013	0.917	0.589	0.756	0.621	0.009	0.069	0.605	0.771	0.009	0.041	0.009	0.075
B5DF27	Lysyl oxidase homolog 2 OS=Rattus norvegicus GN=Loxl2 PE=2 SV=2 - [LOXL2_RAT]	87.1	0.013	0.013	0.013	0.013	0.013	0.013	0.013	0.013	0.013	0.013	0.013	0.013	0.013	0.013	0.013	0.704	1.688	1.327	1.029	1.295	#DIV/0!	#DIV/0!	#DIV/0!	#DIV/0!	0.009	0.042	#DIV/0!	#DIV/0!	0.009	0.016	0.009	0.035
F1LNI5	Protein phosphatase 1G OS=Rattus norvegicus GN=Ppm1g PE=1 SV=2 - [PPM1G_RAT]	58.7	1.037	1.095	1.257	0.441	0.418	0.918	1.074	1.452	0.233	1.118	0.525	0.907	1.072	1.736	0.248	0.013	0.013	0.013	0.013	0.013	0.756	0.520	0.917	0.640	0.009	0.065	0.605	0.683	0.009	0.019	0.009	0.062
O55156	CAP-Gly domain-containing linker protein 2 OS=Rattus norvegicus OX=10116 GN=Clip2 PE=1 SV=1 - [CLIP2_RAT]	115.4	0.622	0.614	1.217	0.358	0.433	0.013	0.013	0.013	0.013	0.013	0.948	1.065	0.756	0.986	0.692	0.013	0.013	0.013	0.013	0.013	0.009	0.142	0.127	0.283	0.009	0.156	0.009	0.067	#DIV/0!	#DIV/0!	0.009	0.064
O70257	Syntaxin-7 OS=Rattus norvegicus GN=Stx7 PE=1 SV=4 - [STX7_RAT]	29.8	0.013	0.013	0.013	0.013	0.013	0.013	0.013	0.013	0.013	0.013	0.394	0.583	1.030	1.935	0.196	0.013	0.013	0.013	0.013	0.013	#DIV/0!	#DIV/0!	0.009	0.067	#DIV/0!	#DIV/0!	0.009	0.089	#DIV/0!	#DIV/0!	0.009	0.085
O70260	E3 SUMO-protein ligase PIAS3 OS=Rattus norvegicus OX=10116 GN=Pias3 PE=1 SV=2 - [PIAS3_RAT]	68.3	0.013	0.013	0.013	0.013	0.013	0.013	0.013	0.013	0.013	0.013	0.013	0.013	0.013	0.013	0.013	0.536	2.259	1.706	1.830	0.655	#DIV/0!	#DIV/0!	#DIV/0!	#DIV/0!	0.009	0.041	#DIV/0!	#DIV/0!	0.009	0.015	0.009	0.034
O70593	Small glutamine-rich tetratricopeptide repeat-containing protein alpha OS=Rattus norvegicus GN=Sgta PE=1 SV=1 - [SGTA_RAT]	34.1	0.724	0.831	1.617	0.490	0.674	0.735	0.790	1.129	0.085	0.611	0.013	0.013	0.013	0.013	0.013	0.657	1.015	1.419	1.063	1.025	0.756	0.560	0.009	0.053	0.354	0.718	0.009	0.295	0.184	0.161	0.009	0.041
O88807	Protein-arginine deiminase type-4 OS=Rattus norvegicus OX=10116 GN=Padi4 PE=2 SV=1 - [PADI4_RAT]	74.4	2.373	1.590	1.096	0.343	0.757	0.920	0.500	0.972	0.559	0.389	0.013	0.013	0.013	0.013	0.013	0.895	1.797	1.127	0.767	1.683	0.259	0.349	0.009	0.025	0.605	0.811	0.009	0.317	0.085	0.099	0.009	0.035
P02564	Myosin-7 OS=Rattus norvegicus GN=Myh7 PE=1 SV=2 - [MYH7_RAT]	222.9	0.013	0.013	0.013	0.013	0.013	0.013	0.013	0.013	0.013	0.013	0.013	0.013	0.013	0.013	0.013	1.008	1.981	1.973	1.650	1.093	#DIV/0!	#DIV/0!	#DIV/0!	#DIV/0!	0.009	0.041	#DIV/0!	#DIV/0!	0.009	0.015	0.009	0.034
P05982	NAD(P)H dehydrogenase [quinone] 1 OS=Rattus norvegicus GN=Nqo1 PE=1 SV=4 - [NQO1_RAT]	30.9	0.013	0.013	0.013	0.013	0.013	0.013	0.013	0.013	0.013	0.013	0.929	1.020	1.150	1.430	0.489	0.013	0.013	0.013	0.013	0.013	#DIV/0!	#DIV/0!	0.009	0.031	#DIV/0!	#DIV/0!	0.009	0.047	#DIV/0!	#DIV/0!	0.009	0.044
P0C5W1	Microtubule-associated protein 1S OS=Rattus norvegicus GN=Map1s PE=1 SV=1 - [MAP1S_RAT]	102.7	0.013	0.013	0.013	0.013	0.013	0.013	0.013	0.013	0.013	0.013	1.640	1.252	0.603	0.787	1.017	0.013	0.013	0.013	0.013	0.013	#DIV/0!	#DIV/0!	0.009	0.028	#DIV/0!	#DIV/0!	0.009	0.043	#DIV/0!	#DIV/0!	0.009	0.039
P10688	1-phosphatidylinositol 4,5-bisphosphate phosphodiesterase delta-1 OS=Rattus norvegicus GN=Plcd1 PE=1 SV=1 - [PLCD1_RAT]	85.9	0.807	0.797	1.619	0.304	0.592	0.932	1.431	1.540	0.190	0.890	0.847	1.292	1.717	1.860	0.266	0.013	0.013	0.013	0.013	0.013	0.470	0.435	0.259	0.416	0.009	0.070	0.605	0.672	0.009	0.018	0.009	0.035
P13264	Glutaminase kidney isoform, mitochondrial OS=Rattus norvegicus GN=Gls PE=1 SV=2 - [GLSK_RAT]	74.0	0.746	0.599	1.740	0.728	0.731	0.543	0.735	0.764	0.116	0.539	0.930	1.302	0.816	1.017	0.997	0.013	0.013	0.013	0.013	0.013	0.259	0.374	0.127	0.277	0.009	0.055	0.014	0.054	0.009	0.203	0.009	0.044
P13437	3-ketoacyl-CoA thiolase, mitochondrial OS=Rattus norvegicus OX=10116 GN=Acaa2 PE=2 SV=1 - [THIM_RAT]	41.8	0.013	0.013	0.013	0.013	0.013	0.456	0.922	0.769	0.190	0.811	0.887	0.919	0.941	1.120	0.508	0.013	0.013	0.013	0.013	0.013	0.009	0.179	0.009	0.051	#DIV/0!	#DIV/0!	0.127	0.404	0.009	0.092	0.009	0.068
P16036	Phosphate carrier protein, mitochondrial OS=Rattus norvegicus OX=10116 GN=Slc25a3 PE=1 SV=1 - [MPCP_RAT]	39.4	0.013	0.013	0.013	0.013	0.013	0.013	0.013	0.013	0.013	0.013	0.013	0.013	0.013	0.013	0.013	0.728	0.699	1.199	0.684	1.080	#DIV/0!	#DIV/0!	#DIV/0!	#DIV/0!	0.009	0.060	#DIV/0!	#DIV/0!	0.009	0.024	0.009	0.067
P16290	Phosphoglycerate mutase 2 OS=Rattus norvegicus GN=Pgam2 PE=2 SV=2 - [PGAM2_RAT]	28.7	1.462	0.967	1.225	0.451	0.400	1.018	1.524	0.664	0.196	0.882	0.013	0.013	0.013	0.013	0.013	0.766	1.280	0.973	0.604	1.190	0.917	0.568	0.009	0.043	0.756	0.853	0.009	0.077	0.756	0.331	0.009	0.047
P17046	Lysosome-associated membrane glycoprotein 2 OS=Rattus norvegicus OX=10116 GN=Lamp2 PE=1 SV=3 - [LAMP2_RAT]	45.1	0.745	0.517	1.741	0.830	0.651	0.896	1.734	1.247	0.471	1.675	1.089	1.175	0.934	1.418	0.501	0.013	0.013	0.013	0.013	0.013	0.470	0.422	0.470	0.518	0.009	0.056	0.605	0.671	0.009	0.016	0.009	0.042
P17136	Small nuclear ribonucleoprotein-associated protein B OS=Rattus norvegicus OX=10116 GN=Snrpb PE=2 SV=2 - [RSMB_RAT]	23.6	0.013	0.013	0.013	0.013	0.013	0.013	0.013	0.013	0.013	0.013	0.836	1.149	1.445	2.290	0.416	0.013	0.013	0.013	0.013	0.013	#DIV/0!	#DIV/0!	0.009	0.025	#DIV/0!	#DIV/0!	0.009	0.038	#DIV/0!	#DIV/0!	0.009	0.035
P18614	Integrin alpha-1 OS=Rattus norvegicus GN=Itga1 PE=1 SV=1 - [ITA1_RAT]	130.7	0.013	0.013	0.013	0.013	0.013	0.013	0.013	0.013	0.013	0.013	0.922	1.328	1.117	1.710	0.415	0.013	0.013	0.013	0.013	0.013	#DIV/0!	#DIV/0!	0.009	0.026	#DIV/0!	#DIV/0!	0.009	0.041	#DIV/0!	#DIV/0!	0.009	0.037
P20650	Protein phosphatase 1A OS=Rattus norvegicus GN=Ppm1a PE=1 SV=1 - [PPM1A_RAT]	42.4	0.013	0.013	0.013	0.013	0.013	0.013	0.013	0.013	0.013	0.013	0.860	1.046	1.007	1.224	0.645	0.013	0.013	0.013	0.013	0.013	#DIV/0!	#DIV/0!	0.009	0.035	#DIV/0!	#DIV/0!	0.009	0.052	#DIV/0!	#DIV/0!	0.009	0.048
P20762	Ig gamma-2C chain C region OS=Rattus norvegicus PE=2 SV=1 - [IGG2C_RAT]	36.5	1.054	0.506	0.869	0.239	0.369	0.711	0.902	0.752	0.747	0.764	0.973	0.963	1.032	1.164	0.591	0.013	0.013	0.013	0.013	0.013	0.470	0.515	0.127	0.282	0.009	0.219	0.127	0.388	0.009	0.035	0.009	0.050
P21396	Amine oxidase [flavin-containing] A OS=Rattus norvegicus GN=Maoa PE=1 SV=1 - [AOFA_RAT]	59.5	0.013	0.013	0.013	0.013	0.013	0.013	0.013	0.013	0.013	0.013	0.627	0.868	0.830	1.420	0.319	0.013	0.013	0.013	0.013	0.013	#DIV/0!	#DIV/0!	0.009	0.073	#DIV/0!	#DIV/0!	0.009	0.096	#DIV/0!	#DIV/0!	0.009	0.093
P25031	Regenerating islet-derived protein 3-beta OS=Rattus norvegicus OX=10116 GN=Reg3b PE=1 SV=1 - [REG3B_RAT]	19.6	0.013	0.013	0.013	0.013	0.013	0.013	0.013	0.013	0.013	0.013	0.013	0.013	0.013	0.013	0.013	0.382	0.990	0.602	6.156	0.348	#DIV/0!	#DIV/0!	#DIV/0!	#DIV/0!	0.009	0.040	#DIV/0!	#DIV/0!	0.009	0.015	0.009	0.033
P27008	Poly [ADP-ribose] polymerase 1 OS=Rattus norvegicus GN=Parp1 PE=1 SV=4 - [PARP1_RAT]	112.6	0.013	0.013	0.013	0.013	0.013	0.013	0.013	0.013	0.013	0.013	1.025	1.766	0.967	1.031	0.570	0.013	0.013	0.013	0.013	0.013	#DIV/0!	#DIV/0!	0.009	0.027	#DIV/0!	#DIV/0!	0.009	0.042	#DIV/0!	#DIV/0!	0.009	0.038
P27653	C-1-tetrahydrofolate synthase, cytoplasmic OS=Rattus norvegicus GN=Mthfd1 PE=1 SV=3 - [C1TC_RAT]	100.9	0.371	0.351	1.445	0.474	0.464	0.560	0.701	0.947	0.281	0.585	0.843	1.180	0.859	1.377	0.455	0.013	0.013	0.013	0.013	0.013	0.470	0.525	0.259	0.446	0.009	0.179	0.184	0.509	0.009	0.099	0.009	0.050
P28075	Proteasome subunit beta type-5 OS=Rattus norvegicus GN=Psmb5 PE=1 SV=3 - [PSB5_RAT]	28.6	0.732	0.746	1.429	0.459	0.636	1.039	1.524	1.292	0.523	1.106	0.848	0.857	1.049	1.104	0.313	0.013	0.013	0.013	0.013	0.013	0.259	0.350	0.470	0.550	0.009	0.075	0.184	0.457	0.009	0.017	0.009	0.082
P28648	CD63 antigen OS=Rattus norvegicus GN=Cd63 PE=1 SV=2 - [CD63_RAT]	25.7	0.013	0.013	0.013	0.013	0.013	0.013	0.013	0.013	0.013	0.013	1.433	1.144	0.863	1.941	0.367	0.013	0.013	0.013	0.013	0.013	#DIV/0!	#DIV/0!	0.009	0.026	#DIV/0!	#DIV/0!	0.009	0.039	#DIV/0!	#DIV/0!	0.009	0.036
P29418	ATP synthase subunit epsilon, mitochondrial OS=Rattus norvegicus OX=10116 GN=Atp5f1e PE=1 SV=2 - [ATP5E_RAT]	5.8	0.013	0.013	0.013	0.013	0.013	0.013	0.013	0.013	0.013	0.013	0.758	0.744	0.791	1.839	0.363	0.013	0.013	0.013	0.013	0.013	#DIV/0!	#DIV/0!	0.009	0.043	#DIV/0!	#DIV/0!	0.009	0.064	#DIV/0!	#DIV/0!	0.009	0.061
P29457	Serpin H1 OS=Rattus norvegicus GN=Serpinh1 PE=1 SV=1 - [SERPH_RAT]	46.5	0.013	0.013	0.013	0.013	0.013	0.013	0.013	0.013	0.013	0.013	0.941	1.047	2.508	1.349	0.462	0.013	0.013	0.013	0.013	0.013	#DIV/0!	#DIV/0!	0.009	0.024	#DIV/0!	#DIV/0!	0.009	0.037	#DIV/0!	#DIV/0!	0.009	0.034
P36876	Serine/threonine-protein phosphatase 2A 55 kDa regulatory subunit B alpha isoform OS=Rattus norvegicus GN=Ppp2r2a PE=2 SV=1 - [2ABA_RAT]	51.6	0.756	0.867	1.162	0.561	0.384	0.661	1.136	1.150	0.336	1.009	0.437	1.318	0.907	1.177	0.328	0.013	0.013	0.013	0.013	0.013	0.756	0.552	0.605	0.609	0.009	0.092	0.917	0.770	0.009	0.025	0.009	0.083
P40329	Arginine--tRNA ligase, cytoplasmic OS=Rattus norvegicus GN=Rars PE=1 SV=2 - [SYRC_RAT]	75.8	0.013	0.013	0.013	0.013	0.013	0.013	0.013	0.013	0.013	0.013	1.077	0.983	1.187	1.469	0.503	0.013	0.013	0.013	0.013	0.013	#DIV/0!	#DIV/0!	0.009	0.028	#DIV/0!	#DIV/0!	0.009	0.044	#DIV/0!	#DIV/0!	0.009	0.040
P43245	Multidrug resistance protein 1 OS=Rattus norvegicus GN=Abcb1 PE=2 SV=1 - [MDR1_RAT]	141.3	0.013	0.013	0.013	0.013	0.013	0.013	0.013	0.013	0.013	0.013	0.013	0.013	0.013	0.013	0.013	0.629	0.946	1.176	0.985	1.139	#DIV/0!	#DIV/0!	#DIV/0!	#DIV/0!	0.009	0.051	#DIV/0!	#DIV/0!	0.009	0.019	0.009	0.047
P43424	Galactose-1-phosphate uridylyltransferase OS=Rattus norvegicus OX=10116 GN=Galt PE=2 SV=1 - [GALT_RAT]	43.3	0.013	0.013	0.013	0.013	0.013	0.013	0.013	0.013	0.013	0.013	0.963	0.865	0.868	0.894	1.094	0.013	0.013	0.013	0.013	0.013	#DIV/0!	#DIV/0!	0.009	0.037	#DIV/0!	#DIV/0!	0.009	0.054	#DIV/0!	#DIV/0!	0.009	0.052
P52873	Pyruvate carboxylase, mitochondrial OS=Rattus norvegicus GN=Pc PE=1 SV=2 - [PYC_RAT]	129.7	0.013	0.013	0.013	0.013	0.013	0.435	0.652	0.757	0.126	0.769	0.806	0.869	1.047	1.322	0.638	0.013	0.013	0.013	0.013	0.013	0.009	0.290	0.009	0.037	#DIV/0!	#DIV/0!	0.056	0.201	0.009	0.185	0.009	0.052
P52944	PDZ and LIM domain protein 1 OS=Rattus norvegicus GN=Pdlim1 PE=1 SV=4 - [PDLI1_RAT]	35.6	0.013	0.013	0.013	0.013	0.013	0.013	0.013	0.013	0.013	0.013	1.108	1.128	1.111	1.764	0.440	0.013	0.013	0.013	0.013	0.013	#DIV/0!	#DIV/0!	0.009	0.026	#DIV/0!	#DIV/0!	0.009	0.041	#DIV/0!	#DIV/0!	0.009	0.037
P54319	Phospholipase A-2-activating protein OS=Rattus norvegicus GN=Plaa PE=2 SV=3 - [PLAP_RAT]	87.0	0.126	0.734	1.063	0.300	0.512	0.013	0.013	0.013	0.013	0.013	0.568	1.240	0.792	1.095	0.489	0.013	0.013	0.013	0.013	0.013	0.009	0.305	0.184	0.387	0.009	0.365	0.009	0.086	#DIV/0!	#DIV/0!	0.009	0.080
P54921	Alpha-soluble NSF attachment protein OS=Rattus norvegicus GN=Napa PE=1 SV=2 - [SNAA_RAT]	33.2	0.013	0.013	0.013	0.013	0.013	0.013	0.013	0.013	0.013	0.013	0.748	1.114	0.967	1.502	0.310	0.013	0.013	0.013	0.013	0.013	#DIV/0!	#DIV/0!	0.009	0.038	#DIV/0!	#DIV/0!	0.009	0.057	#DIV/0!	#DIV/0!	0.009	0.055
P58775	Tropomyosin beta chain OS=Rattus norvegicus GN=Tpm2 PE=3 SV=1 - [TPM2_RAT]	32.8	0.013	0.013	0.013	0.013	0.013	0.013	0.013	0.013	0.013	0.013	0.962	1.172	0.908	1.302	0.900	0.013	0.013	0.013	0.013	0.013	#DIV/0!	#DIV/0!	0.009	0.028	#DIV/0!	#DIV/0!	0.009	0.044	#DIV/0!	#DIV/0!	0.009	0.040
P61515	Putative 60S ribosomal protein L37a OS=Rattus norvegicus OX=10116 GN=Rpl37a-ps1 PE=5 SV=2 - [RL37P_RAT]	10.3	0.470	0.768	0.727	0.390	0.398	0.709	0.865	1.161	0.165	0.928	0.914	1.159	1.078	0.826	0.542	0.013	0.013	0.013	0.013	0.013	0.259	0.384	0.036	0.090	0.009	0.337	0.756	0.766	0.009	0.036	0.009	0.060
P62634	Cellular nucleic acid-binding protein OS=Rattus norvegicus GN=Cnbp PE=2 SV=1 - [CNBP_RAT]	19.4	0.844	0.886	1.518	0.448	0.684	0.673	1.053	1.091	0.142	0.809	0.745	0.979	1.133	1.399	0.402	0.013	0.013	0.013	0.013	0.013	0.756	0.548	0.756	0.595	0.009	0.061	0.470	0.699	0.009	0.039	0.009	0.054
P62870	Transcription elongation factor B polypeptide 2 OS=Rattus norvegicus GN=Tceb2 PE=1 SV=1 - [ELOB_RAT]	13.2	0.013	0.013	0.013	0.013	0.013	0.013	0.013	0.013	0.013	0.013	0.871	1.010	0.969	1.541	0.410	0.013	0.013	0.013	0.013	0.013	#DIV/0!	#DIV/0!	0.009	0.035	#DIV/0!	#DIV/0!	0.009	0.051	#DIV/0!	#DIV/0!	0.009	0.048
P70541	Translation initiation factor eIF-2B subunit gamma OS=Rattus norvegicus GN=Eif2b3 PE=2 SV=2 - [EI2BG_RAT]	50.4	0.013	0.013	0.013	0.013	0.013	0.013	0.013	0.013	0.013	0.013	0.979	1.283	0.801	1.581	0.404	0.013	0.013	0.013	0.013	0.013	#DIV/0!	#DIV/0!	0.009	0.031	#DIV/0!	#DIV/0!	0.009	0.047	#DIV/0!	#DIV/0!	0.009	0.044
P70569	Unconventional myosin-Vb OS=Rattus norvegicus OX=10116 GN=Myo5b PE=1 SV=1 - [MYO5B_RAT]	213.6	0.013	0.013	0.013	0.013	0.013	0.013	0.013	0.013	0.013	0.013	0.013	0.013	0.013	0.013	0.013	0.612	1.392	1.399	1.063	1.073	#DIV/0!	#DIV/0!	#DIV/0!	#DIV/0!	0.009	0.045	#DIV/0!	#DIV/0!	0.009	0.016	0.009	0.037
P70580	Membrane-associated progesterone receptor component 1 OS=Rattus norvegicus GN=Pgrmc1 PE=1 SV=3 - [PGRC1_RAT]	21.6	0.825	0.418	2.199	0.619	0.560	0.625	0.848	1.107	0.223	0.736	0.771	0.920	0.980	1.313	0.451	0.013	0.013	0.013	0.013	0.013	0.756	0.548	0.470	0.528	0.009	0.054	0.354	0.666	0.009	0.051	0.009	0.065
P70582	Nuclear pore complex protein Nup54 OS=Rattus norvegicus GN=Nup54 PE=1 SV=1 - [NUP54_RAT]	55.7	0.891	0.846	1.789	0.757	0.623	1.092	1.170	1.463	0.402	0.626	0.654	1.049	0.951	1.192	0.404	0.013	0.013	0.013	0.013	0.013	0.917	0.549	0.917	0.628	0.009	0.050	0.756	0.710	0.009	0.020	0.009	0.077
P81795	Eukaryotic translation initiation factor 2 subunit 3 OS=Rattus norvegicus GN=Eif2s3 PE=1 SV=2 - [IF2G_RAT]	51.0	0.013	0.013	0.013	0.013	0.013	0.628	0.831	0.967	0.084	0.709	1.075	1.109	0.856	1.246	0.626	0.013	0.013	0.013	0.013	0.013	0.009	0.149	0.009	0.033	#DIV/0!	#DIV/0!	0.127	0.401	0.009	0.079	0.009	0.046
P85845	Fascin OS=Rattus norvegicus GN=Fscn1 PE=1 SV=1 - [FSCN1_RAT]	54.5	0.013	0.013	0.013	0.013	0.013	0.013	0.013	0.013	0.013	0.013	0.765	1.022	1.158	1.406	0.426	0.013	0.013	0.013	0.013	0.013	#DIV/0!	#DIV/0!	0.009	0.036	#DIV/0!	#DIV/0!	0.009	0.052	#DIV/0!	#DIV/0!	0.009	0.049
P97523	Hepatocyte growth factor receptor OS=Rattus norvegicus GN=Met PE=1 SV=1 - [MET_RAT]	153.8	0.373	0.416	2.708	0.243	0.730	0.719	0.585	0.924	0.053	0.457	0.816	1.136	0.682	1.030	0.636	0.013	0.013	0.013	0.013	0.013	0.917	0.599	0.259	0.425	0.009	0.057	0.127	0.405	0.009	0.177	0.009	0.072
Q07116	Sulfite oxidase, mitochondrial OS=Rattus norvegicus GN=Suox PE=1 SV=2 - [SUOX_RAT]	60.8	0.824	0.624	1.632	0.992	0.713	0.645	0.601	0.792	0.205	0.532	0.013	0.013	0.013	0.013	0.013	0.574	0.959	1.330	0.898	0.830	0.056	0.117	0.009	0.035	0.917	0.856	0.009	0.658	0.056	0.077	0.009	0.057
Q09326	Alpha-1,6-mannosyl-glycoprotein 2-beta-N-acetylglucosaminyltransferase OS=Rattus norvegicus GN=Mgat2 PE=1 SV=1 - [MGAT2_RAT]	51.1	0.013	0.013	0.013	0.013	0.013	0.013	0.013	0.013	0.013	0.013	0.013	0.013	0.013	0.013	0.013	0.607	0.823	1.303	0.683	0.994	#DIV/0!	#DIV/0!	#DIV/0!	#DIV/0!	0.009	0.059	#DIV/0!	#DIV/0!	0.009	0.023	0.009	0.065
Q32PZ3	Protein unc-45 homolog A OS=Rattus norvegicus GN=Unc45a PE=2 SV=1 - [UN45A_RAT]	103.2	0.013	0.013	0.013	0.013	0.013	0.013	0.013	0.013	0.013	0.013	0.013	0.013	0.013	0.013	0.013	0.834	0.942	1.749	1.035	2.981	#DIV/0!	#DIV/0!	#DIV/0!	#DIV/0!	0.009	0.041	#DIV/0!	#DIV/0!	0.009	0.015	0.009	0.034
Q3B8Q2	Eukaryotic initiation factor 4A-III OS=Rattus norvegicus GN=Eif4a3 PE=1 SV=1 - [IF4A3_RAT]	46.8	0.013	0.013	0.013	0.013	0.013	0.013	0.013	0.013	0.013	0.013	0.681	0.927	1.065	1.469	0.308	0.013	0.013	0.013	0.013	0.013	#DIV/0!	#DIV/0!	0.009	0.046	#DIV/0!	#DIV/0!	0.009	0.067	#DIV/0!	#DIV/0!	0.009	0.064
Q3KRD8	Eukaryotic translation initiation factor 6 OS=Rattus norvegicus GN=Eif6 PE=1 SV=1 - [IF6_RAT]	26.6	0.747	0.670	0.979	0.531	0.375	0.557	0.485	0.615	0.309	0.451	0.517	0.583	1.025	1.766	0.013	0.013	0.013	0.013	0.013	0.013	0.184	0.309	0.917	0.683	0.009	0.141	0.354	0.690	0.009	0.327	0.025	0.092
Q4V8H8	EH domain-containing protein 2 OS=Rattus norvegicus GN=Ehd2 PE=1 SV=1 - [EHD2_RAT]	61.2	0.013	0.013	0.013	0.013	0.013	0.013	0.013	0.013	0.013	0.013	0.418	0.557	1.371	1.497	0.225	0.013	0.013	0.013	0.013	0.013	#DIV/0!	#DIV/0!	0.009	0.071	#DIV/0!	#DIV/0!	0.009	0.095	#DIV/0!	#DIV/0!	0.009	0.091
Q562C6	Leucine zipper transcription factor-like protein 1 OS=Rattus norvegicus GN=Lztfl1 PE=2 SV=1 - [LZTL1_RAT]	34.6	0.013	0.013	0.013	0.013	0.013	0.013	0.013	0.013	0.013	0.013	0.442	0.848	1.206	1.485	0.160	0.013	0.013	0.013	0.013	0.013	#DIV/0!	#DIV/0!	0.009	0.066	#DIV/0!	#DIV/0!	0.009	0.088	#DIV/0!	#DIV/0!	0.009	0.084
Q5BJN8	Replication stress response regulator SDE2 OS=Rattus norvegicus OX=10116 GN=Sde2 PE=1 SV=2 - [SDE2_RAT]	48.1	0.013	0.013	0.013	0.013	0.013	0.013	0.013	0.013	0.013	0.013	0.013	0.013	0.013	0.013	0.013	0.684	2.667	2.439	2.312	1.078	#DIV/0!	#DIV/0!	#DIV/0!	#DIV/0!	0.009	0.040	#DIV/0!	#DIV/0!	0.009	0.015	0.009	0.033
Q5FVH2	Phospholipase D3 OS=Rattus norvegicus GN=Pld3 PE=2 SV=1 - [PLD3_RAT]	54.4	0.013	0.013	0.013	0.013	0.013	0.013	0.013	0.013	0.013	0.013	0.013	0.013	0.013	0.013	0.013	0.568	0.864	1.193	0.833	0.965	#DIV/0!	#DIV/0!	#DIV/0!	#DIV/0!	0.009	0.058	#DIV/0!	#DIV/0!	0.009	0.023	0.009	0.065
Q5HZE4	Methylthioribose-1-phosphate isomerase OS=Rattus norvegicus GN=Mri1 PE=2 SV=1 - [MTNA_RAT]	39.6	0.013	0.013	0.013	0.013	0.013	0.013	0.013	0.013	0.013	0.013	1.030	1.137	0.993	1.562	0.560	0.013	0.013	0.013	0.013	0.013	#DIV/0!	#DIV/0!	0.009	0.028	#DIV/0!	#DIV/0!	0.009	0.043	#DIV/0!	#DIV/0!	0.009	0.040
Q5PPJ4	Deoxyhypusine hydroxylase OS=Rattus norvegicus OX=10116 GN=Dohh PE=2 SV=1 - [DOHH_RAT]	33.1	0.013	0.013	0.013	0.013	0.013	0.013	0.013	0.013	0.013	0.013	0.013	0.013	0.013	0.013	0.013	0.681	1.025	1.318	0.819	1.129	#DIV/0!	#DIV/0!	#DIV/0!	#DIV/0!	0.009	0.049	#DIV/0!	#DIV/0!	0.009	0.018	0.009	0.045
Q5PQL8	Protein FAM149B1 OS=Rattus norvegicus OX=10116 GN=Fam149b1 PE=2 SV=1 - [F149B_RAT]	53.2	1.349	1.207	1.115	0.659	0.710	0.983	0.411	0.967	0.289	0.311	0.013	0.013	0.013	0.013	0.013	0.657	1.305	1.297	0.926	1.262	0.085	0.169	0.009	0.031	0.756	0.834	0.009	0.535	0.085	0.102	0.009	0.038
Q5PQN7	Protein LZIC OS=Rattus norvegicus OX=10116 GN=Lzic PE=2 SV=1 - [LZIC_RAT]	21.4	0.013	0.013	0.013	0.013	0.013	0.013	0.013	0.013	0.013	0.013	0.013	0.013	0.013	0.013	0.013	0.720	0.950	1.311	0.945	0.951	#DIV/0!	#DIV/0!	#DIV/0!	#DIV/0!	0.009	0.051	#DIV/0!	#DIV/0!	0.009	0.019	0.009	0.046
Q5XIA3	tRNA wybutosine-synthesizing protein 4 OS=Rattus norvegicus OX=10116 GN=Lcmt2 PE=2 SV=1 - [TYW4_RAT]	75.5	0.415	0.564	0.771	0.547	0.889	0.013	0.013	0.013	0.013	0.013	0.974	1.098	1.259	1.335	0.442	0.013	0.013	0.013	0.013	0.013	0.009	0.165	0.085	0.200	0.009	0.168	0.009	0.046	#DIV/0!	#DIV/0!	0.009	0.042
Q5XIP1	Protein pelota homolog OS=Rattus norvegicus GN=Pelo PE=1 SV=1 - [PELO_RAT]	43.4	0.013	0.013	0.013	0.013	0.013	0.013	0.013	0.013	0.013	0.013	1.217	1.442	2.141	1.498	0.496	0.013	0.013	0.013	0.013	0.013	#DIV/0!	#DIV/0!	0.009	0.024	#DIV/0!	#DIV/0!	0.009	0.037	#DIV/0!	#DIV/0!	0.009	0.034
Q5XIU9	Membrane-associated progesterone receptor component 2 OS=Rattus norvegicus OX=10116 GN=Pgrmc2 PE=1 SV=1 - [PGRC2_RAT]	23.4	0.013	0.013	0.013	0.013	0.013	0.013	0.013	0.013	0.013	0.013	1.330	1.191	0.879	1.754	0.653	0.013	0.013	0.013	0.013	0.013	#DIV/0!	#DIV/0!	0.009	0.025	#DIV/0!	#DIV/0!	0.009	0.039	#DIV/0!	#DIV/0!	0.009	0.036
Q62896	BET1 homolog OS=Rattus norvegicus GN=Bet1 PE=1 SV=1 - [BET1_RAT]	13.2	0.013	0.013	0.013	0.013	0.013	0.013	0.013	0.013	0.013	0.013	0.673	0.995	0.954	1.847	0.198	0.013	0.013	0.013	0.013	0.013	#DIV/0!	#DIV/0!	0.009	0.037	#DIV/0!	#DIV/0!	0.009	0.055	#DIV/0!	#DIV/0!	0.009	0.053
Q63092	CaM kinase-like vesicle-associated protein OS=Rattus norvegicus GN=Camkv PE=1 SV=1 - [CAMKV_RAT]	54.1	0.013	0.013	0.013	0.013	0.013	0.013	0.013	0.013	0.013	0.013	2.334	0.810	0.498	0.719	1.944	0.013	0.013	0.013	0.013	0.013	#DIV/0!	#DIV/0!	0.009	0.024	#DIV/0!	#DIV/0!	0.009	0.038	#DIV/0!	#DIV/0!	0.009	0.035
Q63135	Complement component receptor 1-like protein OS=Rattus norvegicus GN=Cr1l PE=1 SV=1 - [CR1L_RAT]	61.6	0.013	0.013	0.013	0.013	0.013	0.013	0.013	0.013	0.013	0.013	0.750	1.003	0.859	1.491	0.384	0.013	0.013	0.013	0.013	0.013	#DIV/0!	#DIV/0!	0.009	0.044	#DIV/0!	#DIV/0!	0.009	0.065	#DIV/0!	#DIV/0!	0.009	0.062
Q63159	Ubiquinone biosynthesis O-methyltransferase, mitochondrial OS=Rattus norvegicus OX=10116 GN=Coq3 PE=2 SV=2 - [COQ3_RAT]	38.7	0.013	0.013	0.013	0.013	0.013	0.013	0.013	0.013	0.013	0.013	0.698	1.092	0.897	1.013	0.645	0.013	0.013	0.013	0.013	0.013	#DIV/0!	#DIV/0!	0.009	0.052	#DIV/0!	#DIV/0!	0.009	0.072	#DIV/0!	#DIV/0!	0.009	0.070
Q63663	Guanylate-binding protein 2 OS=Rattus norvegicus GN=Gbp2 PE=1 SV=2 - [GBP2_RAT]	67.1	0.013	0.013	0.013	0.013	0.013	0.013	0.013	0.013	0.013	0.013	0.612	1.032	1.408	1.631	0.247	0.013	0.013	0.013	0.013	0.013	#DIV/0!	#DIV/0!	0.009	0.032	#DIV/0!	#DIV/0!	0.009	0.048	#DIV/0!	#DIV/0!	0.009	0.045
Q63862	Myosin-11 (Fragments) OS=Rattus norvegicus GN=Myh11 PE=1 SV=3 - [MYH11_RAT]	152.4	0.013	0.013	0.013	0.013	0.013	0.013	0.013	0.013	0.013	0.013	0.983	1.190	1.034	1.270	0.714	0.013	0.013	0.013	0.013	0.013	#DIV/0!	#DIV/0!	0.009	0.029	#DIV/0!	#DIV/0!	0.009	0.045	#DIV/0!	#DIV/0!	0.009	0.041
Q64119	Myosin light polypeptide 6 OS=Rattus norvegicus GN=Myl6 PE=1 SV=3 - [MYL6_RAT]	17.0	0.013	0.013	0.013	0.013	0.013	0.013	0.013	0.013	0.013	0.013	0.283	0.623	1.007	2.306	0.491	0.013	0.013	0.013	0.013	0.013	#DIV/0!	#DIV/0!	0.009	0.036	#DIV/0!	#DIV/0!	0.009	0.053	#DIV/0!	#DIV/0!	0.009	0.051
Q641Z8	Peflin OS=Rattus norvegicus GN=Pef1 PE=2 SV=1 - [PEF1_RAT]	30.0	0.917	0.626	1.632	0.445	0.632	0.784	0.931	0.960	0.116	0.728	0.709	1.137	0.896	1.270	0.371	0.013	0.013	0.013	0.013	0.013	0.756	0.558	0.756	0.611	0.009	0.065	0.605	0.780	0.009	0.052	0.009	0.067
Q64270	Translation initiation factor eIF-2B subunit alpha OS=Rattus norvegicus GN=Eif2b1 PE=2 SV=1 - [EI2BA_RAT]	33.7	0.013	0.013	0.013	0.013	0.013	0.013	0.013	0.013	0.013	0.013	0.013	0.013	0.013	0.013	0.013	0.499	1.049	1.154	0.642	1.064	#DIV/0!	#DIV/0!	#DIV/0!	#DIV/0!	0.009	0.060	#DIV/0!	#DIV/0!	0.009	0.023	0.009	0.066
Q64537	Calpain small subunit 1 OS=Rattus norvegicus GN=Capns1 PE=1 SV=3 - [CPNS1_RAT]	28.6	0.013	0.013	0.013	0.013	0.013	0.013	0.013	0.013	0.013	0.013	0.680	1.061	1.035	1.291	0.453	0.013	0.013	0.013	0.013	0.013	#DIV/0!	#DIV/0!	0.009	0.042	#DIV/0!	#DIV/0!	0.009	0.063	#DIV/0!	#DIV/0!	0.009	0.060
Q66H68	RNA-binding protein 47 OS=Rattus norvegicus GN=Rbm47 PE=2 SV=1 - [RBM47_RAT]	64.1	0.013	0.013	0.013	0.013	0.013	0.013	0.013	0.013	0.013	0.013	0.759	0.981	0.868	1.270	0.505	0.013	0.013	0.013	0.013	0.013	#DIV/0!	#DIV/0!	0.009	0.050	#DIV/0!	#DIV/0!	0.009	0.070	#DIV/0!	#DIV/0!	0.009	0.068
Q66H74	Golgi phosphoprotein 3-like OS=Rattus norvegicus GN=Golph3l PE=2 SV=1 - [GLP3L_RAT]	32.9	0.013	0.013	0.013	0.013	0.013	0.013	0.013	0.013	0.013	0.013	0.904	1.306	1.080	1.327	0.593	0.013	0.013	0.013	0.013	0.013	#DIV/0!	#DIV/0!	0.009	0.028	#DIV/0!	#DIV/0!	0.009	0.044	#DIV/0!	#DIV/0!	0.009	0.040
Q66HB6	Cancer-associated gene 1 protein homolog OS=Rattus norvegicus GN=Cage1 PE=2 SV=1 - [CAGE1_RAT]	97.4	0.013	0.013	0.013	0.013	0.013	0.013	0.013	0.013	0.013	0.013	0.013	0.013	0.013	0.013	0.013	0.440	1.110	0.934	0.787	0.832	#DIV/0!	#DIV/0!	#DIV/0!	#DIV/0!	0.009	0.072	#DIV/0!	#DIV/0!	0.009	0.028	0.009	0.089
Q6AXY0	Glutathione S-transferase A6 OS=Rattus norvegicus GN=Gsta6 PE=1 SV=1 - [GSTA6_RAT]	25.8	1.049	0.754	1.442	0.691	0.819	0.816	1.056	1.139	0.295	0.981	0.565	0.921	0.915	1.362	0.351	0.013	0.013	0.013	0.013	0.013	0.917	0.560	0.605	0.581	0.009	0.052	0.756	0.760	0.009	0.025	0.009	0.088
Q6AY65	Arfaptin-2 OS=Rattus norvegicus GN=Arfip2 PE=2 SV=1 - [ARFP2_RAT]	37.7	0.013	0.013	0.013	0.013	0.013	0.013	0.013	0.013	0.013	0.013	0.916	1.111	0.926	1.339	0.574	0.013	0.013	0.013	0.013	0.013	#DIV/0!	#DIV/0!	0.009	0.034	#DIV/0!	#DIV/0!	0.009	0.049	#DIV/0!	#DIV/0!	0.009	0.047
Q6GX84	Fidgetin-like protein 1 OS=Rattus norvegicus GN=Fignl1 PE=2 SV=1 - [FIGL1_RAT]	74.2	0.013	0.013	0.013	0.013	0.013	0.013	0.013	0.013	0.013	0.013	0.973	1.154	0.806	1.242	0.438	0.013	0.013	0.013	0.013	0.013	#DIV/0!	#DIV/0!	0.009	0.039	#DIV/0!	#DIV/0!	0.009	0.059	#DIV/0!	#DIV/0!	0.009	0.056
Q6IFU7	Keratin, type I cytoskeletal 42 OS=Rattus norvegicus GN=Krt42 PE=3 SV=1 - [K1C42_RAT]	50.2	0.013	0.013	0.013	0.013	0.013	0.013	0.013	0.013	0.013	0.013	0.770	0.818	5.742	0.882	0.581	0.013	0.013	0.013	0.013	0.013	#DIV/0!	#DIV/0!	0.009	0.023	#DIV/0!	#DIV/0!	0.009	0.036	#DIV/0!	#DIV/0!	0.009	0.033
Q6IFU8	Keratin, type I cytoskeletal 17 OS=Rattus norvegicus GN=Krt17 PE=2 SV=1 - [K1C17_RAT]	48.1	0.013	0.013	0.013	0.013	0.013	0.013	0.013	0.013	0.013	0.013	0.678	0.535	8.010	0.595	0.541	0.013	0.013	0.013	0.013	0.013	#DIV/0!	#DIV/0!	0.009	0.023	#DIV/0!	#DIV/0!	0.009	0.036	#DIV/0!	#DIV/0!	0.009	0.033
Q6P6S4	Nucleotide exchange factor SIL1 OS=Rattus norvegicus GN=Sil1 PE=2 SV=1 - [SIL1_RAT]	52.3	0.782	0.741	1.400	0.610	0.648	0.838	0.912	1.189	0.188	0.923	0.992	0.990	0.842	1.282	0.409	0.013	0.013	0.013	0.013	0.013	0.470	0.460	0.470	0.539	0.009	0.068	0.470	0.690	0.009	0.030	0.009	0.060
Q6P7P5	Basic leucine zipper and W2 domain-containing protein 1 OS=Rattus norvegicus GN=Bzw1 PE=2 SV=1 - [BZW1_RAT]	48.0	0.013	0.013	0.013	0.013	0.013	0.013	0.013	0.013	0.013	0.013	0.455	1.025	1.702	2.016	0.399	0.013	0.013	0.013	0.013	0.013	#DIV/0!	#DIV/0!	0.009	0.026	#DIV/0!	#DIV/0!	0.009	0.040	#DIV/0!	#DIV/0!	0.009	0.037
Q6PDU1	Serine/arginine-rich splicing factor 2 OS=Rattus norvegicus GN=Srsf2 PE=1 SV=3 - [SRSF2_RAT]	25.5	0.013	0.013	0.013	0.013	0.013	0.013	0.013	0.013	0.013	0.013	0.993	1.013	1.526	1.775	0.548	0.013	0.013	0.013	0.013	0.013	#DIV/0!	#DIV/0!	0.009	0.025	#DIV/0!	#DIV/0!	0.009	0.039	#DIV/0!	#DIV/0!	0.009	0.036
Q71UF4	Histone-binding protein RBBP7 OS=Rattus norvegicus GN=Rbbp7 PE=2 SV=1 - [RBBP7_RAT]	47.8	0.749	0.847	1.636	0.630	0.665	0.654	0.741	1.031	0.333	0.684	0.013	0.013	0.013	0.013	0.013	0.624	0.952	1.516	1.149	1.062	0.470	0.465	0.009	0.041	0.470	0.757	0.009	0.244	0.127	0.124	0.009	0.039
Q7TT51	Lengsin OS=Rattus norvegicus OX=10116 GN=Lgsn PE=2 SV=1 - [LGSN_RAT]	62.2	0.013	0.013	0.013	0.013	0.013	0.013	0.013	0.013	0.013	0.013	0.728	1.356	1.242	1.193	0.176	0.013	0.013	0.013	0.013	0.013	#DIV/0!	#DIV/0!	0.009	0.036	#DIV/0!	#DIV/0!	0.009	0.053	#DIV/0!	#DIV/0!	0.009	0.051
Q91ZV2	Discoidin, CUB and LCCL domain-containing protein 2 OS=Rattus norvegicus GN=Dcbld2 PE=2 SV=1 - [DCBD2_RAT]	83.9	0.013	0.013	0.013	0.013	0.013	0.013	0.013	0.013	0.013	0.013	1.106	1.171	1.023	1.581	0.692	0.013	0.013	0.013	0.013	0.013	#DIV/0!	#DIV/0!	0.009	0.026	#DIV/0!	#DIV/0!	0.009	0.041	#DIV/0!	#DIV/0!	0.009	0.037
Q99ND9	RWD domain-containing protein 1 OS=Rattus norvegicus GN=Rwdd1 PE=2 SV=1 - [RWDD1_RAT]	27.8	1.008	0.911	1.855	0.968	0.844	0.959	0.955	1.185	0.295	0.687	0.919	1.154	0.906	1.140	0.623	0.013	0.013	0.013	0.013	0.013	0.354	0.406	0.756	0.583	0.009	0.044	0.917	0.741	0.009	0.028	0.009	0.049
Q9ES21	Phosphatidylinositide phosphatase SAC1 OS=Rattus norvegicus GN=Sacm1l PE=1 SV=1 - [SAC1_RAT]	67.0	0.013	0.013	0.013	0.013	0.013	0.013	0.013	0.013	0.013	0.013	0.930	0.979	0.919	1.323	0.509	0.013	0.013	0.013	0.013	0.013	#DIV/0!	#DIV/0!	0.009	0.038	#DIV/0!	#DIV/0!	0.009	0.056	#DIV/0!	#DIV/0!	0.009	0.054
Q9ES40	PRA1 family protein 3 OS=Rattus norvegicus GN=Arl6ip5 PE=1 SV=1 - [PRAF3_RAT]	21.5	0.013	0.013	0.013	0.013	0.013	0.013	0.013	0.013	0.013	0.013	0.965	1.250	0.753	1.517	0.312	0.013	0.013	0.013	0.013	0.013	#DIV/0!	#DIV/0!	0.009	0.035	#DIV/0!	#DIV/0!	0.009	0.052	#DIV/0!	#DIV/0!	0.009	0.048
Q9ES54	Nuclear protein localization protein 4 homolog OS=Rattus norvegicus GN=Nploc4 PE=1 SV=3 - [NPL4_RAT]	68.0	0.013	0.013	0.013	0.013	0.013	0.013	0.013	0.013	0.013	0.013	0.580	0.929	0.992	1.648	0.151	0.013	0.013	0.013	0.013	0.013	#DIV/0!	#DIV/0!	0.009	0.054	#DIV/0!	#DIV/0!	0.009	0.074	#DIV/0!	#DIV/0!	0.009	0.072
Q9ESS6	Basal cell adhesion molecule OS=Rattus norvegicus OX=10116 GN=Bcam PE=2 SV=1 - [BCAM_RAT]	67.5	0.013	0.013	0.013	0.013	0.013	0.544	1.033	0.880	0.301	0.587	0.888	1.059	0.926	1.297	0.421	0.013	0.013	0.013	0.013	0.013	0.009	0.122	0.009	0.039	#DIV/0!	#DIV/0!	0.184	0.505	0.009	0.071	0.009	0.057
Q9JIY6	Probable N-acetyltransferase CML6 OS=Rattus norvegicus GN=Cml6 PE=2 SV=1 - [CMLO6_RAT]	24.8	0.013	0.013	0.013	0.013	0.013	0.013	0.013	0.013	0.013	0.013	0.815	0.955	0.819	1.691	0.528	0.013	0.013	0.013	0.013	0.013	#DIV/0!	#DIV/0!	0.009	0.035	#DIV/0!	#DIV/0!	0.009	0.051	#DIV/0!	#DIV/0!	0.009	0.047
Q9JJW3	Up-regulated during skeletal muscle growth protein 5 OS=Rattus norvegicus OX=10116 GN=Atp5md PE=1 SV=1 - [USMG5_RAT]	6.4	0.013	0.013	0.013	0.013	0.013	0.535	0.731	0.857	0.197	0.624	0.773	1.245	0.768	1.783	0.443	0.013	0.013	0.013	0.013	0.013	0.009	0.226	0.009	0.032	#DIV/0!	#DIV/0!	0.184	0.502	0.009	0.125	0.009	0.045
Q9JK72	Copper chaperone for superoxide dismutase OS=Rattus norvegicus GN=Ccs PE=1 SV=1 - [CCS_RAT]	28.9	1.350	1.024	2.106	1.079	0.883	0.837	0.628	1.133	0.449	0.614	0.013	0.013	0.013	0.013	0.013	0.733	0.893	1.238	0.923	0.999	0.056	0.114	0.009	0.024	0.184	0.517	0.009	0.164	0.127	0.126	0.009	0.048

Q9JKU3	Intraflagellar transport protein 172 homolog OS=Rattus norvegicus GN=Ift172 PE=1 SV=1 - [IF172_RAT]	197.5	0.013	0.013	0.013	0.013	0.013	0.013	0.013	0.013	0.013	0.013	1.000	1.118	0.928	1.172	0.797	0.013	0.013	0.013	0.013	0.013	#DIV/0!	#DIV/0!	0.009	0.032	#DIV/0!	#DIV/0!	0.009	0.048	#DIV/0!	#DIV/0!	0.009	0.045
Q9JLH7	CDK5 regulatory subunit-associated protein 3 OS=Rattus norvegicus OX=10116 GN=Cdk5rap3 PE=1 SV=1 - [CK5P3_RAT]	57.0	0.013	0.013	0.013	0.013	0.013	0.013	0.013	0.013	0.013	0.013	1.001	0.937	1.269	1.518	0.389	0.013	0.013	0.013	0.013	0.013	#DIV/0!	#DIV/0!	0.009	0.030	#DIV/0!	#DIV/0!	0.009	0.045	#DIV/0!	#DIV/0!	0.009	0.042
Q9JLT0	Myosin-10 OS=Rattus norvegicus GN=Myh10 PE=1 SV=1 - [MYH10_RAT]	228.8	0.013	0.013	0.013	0.013	0.013	0.013	0.013	0.013	0.013	0.013	2.177	1.271	0.603	0.590	1.074	0.013	0.013	0.013	0.013	0.013	#DIV/0!	#DIV/0!	0.009	0.026	#DIV/0!	#DIV/0!	0.009	0.040	#DIV/0!	#DIV/0!	0.009	0.036
Q9QUL6	Vesicle-fusing ATPase OS=Rattus norvegicus GN=Nsf PE=1 SV=1 - [NSF_RAT]	82.6	0.013	0.013	0.013	0.013	0.013	0.013	0.013	0.013	0.013	0.013	1.009	1.079	0.949	1.473	0.742	0.013	0.013	0.013	0.013	0.013	#DIV/0!	#DIV/0!	0.009	0.028	#DIV/0!	#DIV/0!	0.009	0.043	#DIV/0!	#DIV/0!	0.009	0.040
Q9QYL8	Acyl-protein thioesterase 2 OS=Rattus norvegicus GN=Lypla2 PE=1 SV=1 - [LYPA2_RAT]	24.8	0.982	0.763	1.651	0.761	0.789	0.815	0.845	1.091	0.346	0.715	0.013	0.013	0.013	0.013	0.013	0.579	1.073	0.993	0.733	0.808	0.605	0.487	0.009	0.032	0.756	0.860	0.009	0.136	0.917	0.385	0.009	0.079
Q9QZK8	Deoxyribonuclease-2-alpha OS=Rattus norvegicus GN=Dnase2 PE=2 SV=1 - [DNS2A_RAT]	38.2	0.013	0.013	0.013	0.013	0.013	0.013	0.013	0.013	0.013	0.013	0.013	0.013	0.013	0.013	0.013	0.472	1.316	1.013	0.863	1.082	#DIV/0!	#DIV/0!	#DIV/0!	#DIV/0!	0.009	0.053	#DIV/0!	#DIV/0!	0.009	0.020	0.009	0.049
Q9WU82	Catenin beta-1 OS=Rattus norvegicus GN=Ctnnb1 PE=1 SV=1 - [CTNB1_RAT]	85.4	0.714	0.687	1.249	0.427	0.384	0.749	1.414	1.107	0.185	1.247	0.613	0.974	1.021	1.285	0.266	0.013	0.013	0.013	0.013	0.013	0.354	0.422	0.605	0.622	0.009	0.122	0.605	0.702	0.009	0.020	0.009	0.083
Q9WUW3	Complement factor I OS=Rattus norvegicus GN=Cfi PE=2 SV=1 - [CFAI_RAT]	67.3	1.195	0.744	1.862	0.753	0.656	0.876	1.002	1.065	0.141	1.097	0.013	0.013	0.013	0.013	0.013	0.569	1.006	1.249	0.801	0.996	0.917	0.551	0.009	0.029	0.917	0.850	0.009	0.086	0.917	0.372	0.009	0.056
Q9Z2G8	Nucleosome assembly protein 1-like 1 OS=Rattus norvegicus GN=Nap1l1 PE=2 SV=1 - [NP1L1_RAT]	45.3	0.807	0.794	0.692	0.340	0.294	0.727	1.323	1.347	0.339	1.141	0.512	0.928	1.068	1.924	0.279	0.013	0.013	0.013	0.013	0.013	0.184	0.296	0.354	0.514	0.009	0.243	0.605	0.678	0.009	0.019	0.009	0.051

Table S12. Extracted protein profile of e-III (Effect of nutrition, only Sham, Female)

Accession	Description	MW [kDa]	Expression level																				Steel-Dwass & FDR											
			FGR-Good-Female					FGR-Poor_Female					Sham-Good-Female					Sham-Poor-Female					FGR-GN vs FGR-PN		FGR-GN vs Sham-GN		FGR-GN vs Sham-PN		FGR-PN vs Sham-GN		FGR-PN vs Sham-PN		Sham-GN vs Sham-PN	
			#01	#02	#03	#04	#05	#06	#07	#08	#09	#10	#11	#12	#13	#14	#15	#16	#17	#18	#19	#20												
Filter	Filter	Filter	Filter	Filter	Filter	Filter	Filter	Filter	Filter	Filter	Filter	Filter	Filter	Filter	Filter	Filter	Filter	Filter	Filter	Filter	Filter	Filter	p-value	q-value	p	q-value	p	q-value	p	q-value	p	q-value	p	q-value
B4F779	DCC-interacting protein 13-beta OS=Rattus norvegicus OX=10116 GN=Appl2 PE=2 SV=1 - [DP13B_RAT]	74.1	0.013	0.013	0.013	0.013	0.013	0.013	0.013	0.013	0.013	0.013	1.229	2.055	4.003	0.577	3.482	0.013	0.013	0.013	0.013	0.013	#DIV/0!	#DIV/0!	0.009	0.029	#DIV/0!	#DIV/0!	0.009	0.023	#DIV/0!	#DIV/0!	0.009	0.064
B5DEN9	Vacuolar protein sorting-associated protein 28 homolog OS=Rattus norvegicus GN=Vps28 PE=2 SV=1 - [VPS28_RAT]	26.1	0.013	0.013	0.013	0.013	0.013	0.013	0.013	0.013	0.013	0.013	0.013	0.013	0.013	0.013	0.013	2.536	0.826	2.513	2.492	1.648	#DIV/0!	#DIV/0!	#DIV/0!	#DIV/0!	0.009	0.030	#DIV/0!	#DIV/0!	0.009	0.023	0.009	0.069
D3ZKF5	Serine protease HTR4 OS=Rattus norvegicus OX=10116 GN=Htra4 PE=3 SV=1 - [HTRA4_RAT]	52.2	0.013	0.013	0.013	0.013	0.013	0.013	0.013	0.013	0.013	0.013	0.953	1.017	1.750	2.570	1.093	0.013	0.013	0.013	0.013	0.013	#DIV/0!	#DIV/0!	0.009	0.051	#DIV/0!	#DIV/0!	0.009	0.044	#DIV/0!	#DIV/0!	0.009	0.094
D4A2Z8	ATP-dependent DNA/RNA helicase DHX36 OS=Rattus norvegicus OX=10116 GN=Dhx36 PE=1 SV=1 - [DHX36_RAT]	113.8	0.013	0.013	0.013	0.013	0.013	0.013	0.013	0.013	0.013	0.013	1.565	1.492	3.145	1.829	1.429	0.013	0.013	0.013	0.013	0.013	#DIV/0!	#DIV/0!	0.009	0.034	#DIV/0!	#DIV/0!	0.009	0.026	#DIV/0!	#DIV/0!	0.009	0.071
O35263	Platelet-activating factor acetylhydrolase IB subunit gamma OS=Rattus norvegicus GN=Pafah1b3 PE=2 SV=1 - [PA1B3_RAT]	25.8	0.013	0.013	0.013	0.013	0.013	0.013	0.013	0.013	0.013	0.013	1.776	1.636	3.759	3.312	1.896	1.172	0.999	0.989	1.007	0.572	#DIV/0!	#DIV/0!	0.009	0.028	0.009	0.139	0.009	0.022	0.009	0.113	0.014	0.091
O55004	Ribonuclease 4 OS=Rattus norvegicus GN=Rnase4 PE=1 SV=1 - [RNAS4_RAT]	16.9	0.013	0.013	0.013	0.013	0.013	0.013	0.013	0.013	0.013	0.013	1.402	1.242	2.737	2.984	1.494	0.013	0.013	0.013	0.013	0.013	#DIV/0!	#DIV/0!	0.009	0.032	#DIV/0!	#DIV/0!	0.009	0.025	#DIV/0!	#DIV/0!	0.009	0.070
O70437	Mothers against decapentaplegic homolog 4 OS=Rattus norvegicus GN=Smad4 PE=1 SV=1 - [SMAD4_RAT]	60.4	0.013	0.013	0.013	0.013	0.013	0.013	0.013	0.013	0.013	0.013	1.348	1.258	1.979	2.168	1.271	0.013	0.013	0.013	0.013	0.013	#DIV/0!	#DIV/0!	0.009	0.045	#DIV/0!	#DIV/0!	0.009	0.037	#DIV/0!	#DIV/0!	0.009	0.085
O70511	Ankyrin-3 OS=Rattus norvegicus GN=Ank3 PE=1 SV=3 - [ANK3_RAT]	284.3	0.013	0.013	0.013	0.013	0.013	0.013	0.013	0.013	0.013	0.013	1.297	1.190	1.500	7.790	1.036	0.013	0.013	0.013	0.013	0.013	#DIV/0!	#DIV/0!	0.009	0.027	#DIV/0!	#DIV/0!	0.009	0.022	#DIV/0!	#DIV/0!	0.009	0.062
P02764	Alpha-1-acid glycoprotein OS=Rattus norvegicus GN=Orm1 PE=2 SV=1 - [A1AG_RAT]	23.6	0.013	0.013	0.013	0.013	0.013	0.013	0.013	0.013	0.013	0.013	0.942	1.120	2.396	2.691	1.527	0.013	0.013	0.013	0.013	0.013	#DIV/0!	#DIV/0!	0.009	0.040	#DIV/0!	#DIV/0!	0.009	0.031	#DIV/0!	#DIV/0!	0.009	0.078
P09117	Fructose-bisphosphate aldolase C OS=Rattus norvegicus GN=Aldoc PE=1 SV=3 - [ALDOC_RAT]	39.3	0.013	0.013	0.013	0.013	0.013	1.050	0.503	0.969	1.015	0.862	0.013	0.013	0.013	0.013	0.013	3.280	0.549	1.717	1.748	1.539	0.009	0.103	#DIV/0!	#DIV/0!	0.009	0.033	0.009	0.198	0.085	0.092	0.009	0.075
P0C1X8	AP2-associated protein kinase 1 OS=Rattus norvegicus GN=Aak1 PE=1 SV=1 - [AAK1_RAT]	103.7	0.013	0.013	0.013	0.013	0.013	0.013	0.013	0.013	0.013	0.013	0.013	0.013	0.013	0.013	0.013	4.021	0.283	2.371	2.330	1.784	#DIV/0!	#DIV/0!	#DIV/0!	#DIV/0!	0.009	0.029	#DIV/0!	#DIV/0!	0.009	0.023	0.009	0.066
P12369	cAMP-dependent protein kinase type II-beta regulatory subunit OS=Rattus norvegicus GN=Prkar2b PE=1 SV=3 - [KAP3_RAT]	46.1	0.013	0.013	0.013	0.013	0.013	0.013	0.013	0.013	0.013	0.013	0.013	0.013	0.013	0.013	0.013	1.538	1.430	1.324	1.821	1.164	#DIV/0!	#DIV/0!	#DIV/0!	#DIV/0!	0.009	0.045	#DIV/0!	#DIV/0!	0.009	0.035	0.009	0.096
P12788	Trypsin-4 OS=Rattus norvegicus OX=10116 GN=Try4 PE=2 SV=1 - [TRY4_RAT]	26.6	0.391	0.900	0.330	0.880	0.681	0.847	0.365	0.405	1.094	0.779	1.726	2.861	2.911	1.201	1.022	0.013	0.013	0.013	0.013	0.013	0.756	1.117	0.014	0.030	0.009	2.051	0.022	0.037	0.009	0.883	0.009	0.071
P16290	Phosphoglycerate mutase 2 OS=Rattus norvegicus GN=Pgam2 PE=2 SV=2 - [PGAM2_RAT]	28.7	0.013	0.013	0.013	0.013	0.013	0.013	0.013	0.013	0.013	0.013	1.501	1.667	2.363	2.585	0.916	0.013	0.013	0.013	0.013	0.013	#DIV/0!	#DIV/0!	0.009	0.036	#DIV/0!	#DIV/0!	0.009	0.028	#DIV/0!	#DIV/0!	0.009	0.073
P18266	Glycogen synthase kinase-3 beta OS=Rattus norvegicus GN=Gsk3b PE=1 SV=1 - [GSK3B_RAT]	46.7	0.013	0.013	0.013	0.013	0.013	0.013	0.013	0.013	0.013	0.013	2.114	1.772	3.349	4.342	1.332	0.013	0.013	0.013	0.013	0.013	#DIV/0!	#DIV/0!	0.009	0.027	#DIV/0!	#DIV/0!	0.009	0.022	#DIV/0!	#DIV/0!	0.009	0.062
P18614	Integrin alpha-1 OS=Rattus norvegicus GN=Itga1 PE=1 SV=1 - [ITA1_RAT]	130.7	0.013	0.013	0.013	0.013	0.013	0.013	0.013	0.013	0.013	0.013	1.096	0.737	1.959	4.101	1.236	0.013	0.013	0.013	0.013	0.013	#DIV/0!	#DIV/0!	0.009	0.036	#DIV/0!	#DIV/0!	0.009	0.028	#DIV/0!	#DIV/0!	0.009	0.073
P19939	Apolipoprotein C-I OS=Rattus norvegicus GN=Apoc1 PE=3 SV=1 - [APOC1_RAT]	9.9	0.013	0.013	0.013	0.013	0.013	0.013	0.013	0.013	0.013	0.013	0.013	0.013	0.013	0.013	0.013	4.088	0.783	1.414	1.589	0.731	#DIV/0!	#DIV/0!	#DIV/0!	#DIV/0!	0.009	0.034	#DIV/0!	#DIV/0!	0.009	0.026	0.009	0.079
P22057	Prostaglandin-H2 D-isomerase OS=Rattus norvegicus GN=Ptgds PE=1 SV=2 - [PTGDS_RAT]	21.3	0.013	0.013	0.013	0.013	0.013	0.013	0.013	0.013	0.013	0.013	0.013	0.013	0.013	0.013	0.013	3.443	0.910	1.212	2.318	0.847	#DIV/0!	#DIV/0!	#DIV/0!	#DIV/0!	0.009	0.033	#DIV/0!	#DIV/0!	0.009	0.025	0.009	0.076
P23562	Band 3 anion transport protein OS=Rattus norvegicus OX=10116 GN=Slc4a1 PE=1 SV=3 - [B3AT_RAT]	103.1	0.013	0.013	0.013	0.013	0.013	0.013	0.013	0.013	0.013	0.013	0.013	0.013	0.013	0.013	0.013	3.273	0.748	1.327	1.024	1.180	#DIV/0!	#DIV/0!	#DIV/0!	#DIV/0!	0.009	0.042	#DIV/0!	#DIV/0!	0.009	0.032	0.009	0.091
P24464	Cytochrome P450 4A12 OS=Rattus norvegicus OX=10116 GN=Cyp4a12 PE=2 SV=2 - [CP4AC_RAT]	58.5	0.013	0.013	0.013	0.013	0.013	0.013	0.013	0.013	0.013	0.013	0.524	0.538	0.929	7.509	0.440	0.013	0.013	0.013	0.013	0.013	#DIV/0!	#DIV/0!	0.009	0.032	#DIV/0!	#DIV/0!	0.009	0.025	#DIV/0!	#DIV/0!	0.009	0.069
P25031	Regenerating islet-derived protein 3-beta OS=Rattus norvegicus OX=10116 GN=Reg3b PE=1 SV=1 - [REG3B_RAT]	19.6	0.013	0.013	0.013	0.013	0.013	0.013	0.013	0.013	0.013	0.013	1.107	0.789	2.940	7.594	0.886	0.013	0.013	0.013	0.013	0.013	#DIV/0!	#DIV/0!	0.009	0.027	#DIV/0!	#DIV/0!	0.009	0.022	#DIV/0!	#DIV/0!	0.009	0.061
P27008	Poly [ADP-ribose] polymerase 1 OS=Rattus norvegicus GN=Parp1 PE=1 SV=4 - [PARP1_RAT]	112.6	0.013	0.013	0.013	0.013	0.013	0.013	0.013	0.013	0.013	0.013	1.161	1.419	2.534	1.866	0.979	0.013	0.013	0.013	0.013	0.013	#DIV/0!	#DIV/0!	0.009	0.047	#DIV/0!	#DIV/0!	0.009	0.037	#DIV/0!	#DIV/0!	0.009	0.085
P29147	D-beta-hydroxybutyrate dehydrogenase, mitochondrial OS=Rattus norvegicus OX=10116 GN=Bdh1 PE=1 SV=2 - [BDH_RAT]	38.2	0.013	0.013	0.013	0.013	0.013	0.013	0.013	0.013	0.013	0.013	0.013	0.013	0.013	0.013	0.013	3.104	0.987	1.537	2.010	0.888	#DIV/0!	#DIV/0!	#DIV/0!	#DIV/0!	0.009	0.034	#DIV/0!	#DIV/0!	0.009	0.026	0.009	0.079
P42854	Regenerating islet-derived protein 3-gamma OS=Rattus norvegicus OX=10116 GN=Reg3g PE=1 SV=1 - [REG3G_RAT]	19.1	0.013	0.013	0.013	0.013	0.013	0.013	0.013	0.013	0.013	0.013	0.839	0.617	1.529	6.039	0.785	0.013	0.013	0.013	0.013	0.013	#DIV/0!	#DIV/0!	0.009	0.032	#DIV/0!	#DIV/0!	0.009	0.025	#DIV/0!	#DIV/0!	0.009	0.070
P51869	Cytochrome P450 4F4 OS=Rattus norvegicus OX=10116 GN=Cyp4f4 PE=2 SV=1 - [CP4F4_RAT]	60.0	0.013	0.013	0.013	0.013	0.013	0.013	0.013	0.013	0.013	0.013	1.174	1.174	1.592	5.101	1.322	0.013	0.013	0.013	0.013	0.013	#DIV/0!	#DIV/0!	0.009	0.031	#DIV/0!	#DIV/0!	0.009	0.024	#DIV/0!	#DIV/0!	0.009	0.067
P55314	Complement component C8 beta chain OS=Rattus norvegicus GN=C8b PE=2 SV=2 - [CO8B_RAT]	66.6	0.013	0.013	0.013	0.013	0.013	0.013	0.013	0.013	0.013	0.013	0.013	0.013	0.013	0.013	0.013	4.342	0.194	2.109	1.598	1.984	#DIV/0!	#DIV/0!	#DIV/0!	#DIV/0!	0.009	0.030	#DIV/0!	#DIV/0!	0.009	0.023	0.009	0.068
P60825	Cold-inducible RNA-binding protein OS=Rattus norvegicus OX=10116 GN=Cirbp PE=2 SV=1 - [CIRBP_RAT]	18.6	0.013	0.013	0.013	0.013	0.013	0.013	0.013	0.013	0.013	0.013	1.663	1.611	3.606	2.534	2.041	0.013	0.013	0.013	0.013	0.013	#DIV/0!	#DIV/0!	0.009	0.029	#DIV/0!	#DIV/0!	0.009	0.023	#DIV/0!	#DIV/0!	0.009	0.064
P60905	DnaJ homolog subfamily C member 5 OS=Rattus norvegicus OX=10116 GN=Dnajc5 PE=1 SV=1 - [DNJC5_RAT]	22.1	0.013	0.013	0.013	0.013	0.013	0.013	0.013	0.013	0.013	0.013	1.165	1.011	2.177	1.869	1.252	0.013	0.013	0.013	0.013	0.013	#DIV/0!	#DIV/0!	0.009	0.051	#DIV/0!	#DIV/0!	0.009	0.043	#DIV/0!	#DIV/0!	0.009	0.093
P61206	ADP-ribosylation factor 3 OS=Rattus norvegicus GN=Arf3 PE=1 SV=2 - [ARF3_RAT]	20.6	0.013	0.013	0.013	0.013	0.013	0.013	0.013	0.013	0.013	0.013	2.140	1.707	4.424	6.705	2.021	0.013	0.013	0.013	0.013	0.013	#DIV/0!	#DIV/0!	0.009	0.027	#DIV/0!	#DIV/0!	0.009	0.022	#DIV/0!	#DIV/0!	0.009	0.061
P61354	60S ribosomal protein L27 OS=Rattus norvegicus OX=10116 GN=Rpl27 PE=2 SV=2 - [RL27_RAT]	15.8	0.542	1.047	0.760	1.610	1.203	0.843	0.330	0.810	1.085	1.006	1.004	1.216	2.084	1.490	1.478	0.013	0.013	0.013	0.013	0.013	0.470	0.938	0.184	0.198	0.009	0.097	0.036	0.054	0.009	0.327	0.009	0.097
P61621	Protein transport protein Sec61 subunit alpha isoform 1 OS=Rattus norvegicus OX=10116 GN=Sec61a1 PE=2 SV=2 - [S61A1_RAT]	52.2	0.013	0.013	0.013	0.013	0.013	0.013	0.013	0.013	0.013	0.013	1.224	1.106	2.583	1.445	1.389	0.013	0.013	0.013	0.013	0.013	#DIV/0!	#DIV/0!	0.009	0.049	#DIV/0!	#DIV/0!	0.009	0.040	#DIV/0!	#DIV/0!	0.009	0.088
P62494	Ras-related protein Rab-11A OS=Rattus norvegicus GN=Rab11a PE=1 SV=3 - [RB11A_RAT]	24.4	0.013	0.013	0.013	0.013	0.013	0.013	0.013	0.013	0.013	0.013	1.760	1.894	2.920	2.222	2.133	0.013	0.013	0.013	0.013	0.013	#DIV/0!	#DIV/0!	0.009	0.029	#DIV/0!	#DIV/0!	0.009	0.024	#DIV/0!	#DIV/0!	0.009	0.065
P62836	Ras-related protein Rap-1A OS=Rattus norvegicus GN=Rap1a PE=1 SV=1 - [RAP1A_RAT]	21.0	0.013	0.013	0.013	0.013	0.013	0.013	0.013	0.013	0.013	0.013	0.013	0.013	0.013	0.013	0.013	3.329	0.509	1.626	1.507	0.887	#DIV/0!	#DIV/0!	#DIV/0!	#DIV/0!	0.009	0.039	#DIV/0!	#DIV/0!	0.009	0.030	0.009	0.087
P62845	40S ribosomal protein S15 OS=Rattus norvegicus GN=Rps15 PE=1 SV=2 - [RS15_RAT]	17.0	0.013	0.013	0.013	0.013	0.013	0.696	0.193	0.516	0.871	0.927	1.462	2.259	3.751	1.666	1.095	0.013	0.013	0.013	0.013	0.013	0.009	1.078	0.009	0.031	#DIV/0!	#DIV/0!	0.014	0.025	0.009	1.471	0.009	0.068
P62853	40S ribosomal protein S25 OS=Rattus norvegicus GN=Rps25 PE=2 SV=1 - [RS25_RAT]	13.7	0.901	0.929	0.336	1.494	0.907	1.739	0.254	0.344	0.931	0.692	2.171	0.935	2.946	1.549	0.815	0.013	0.013	0.013	0.013	0.013	0.756	0.982	0.127	0.145	0.009	0.171	0.085	0.087	0.009	0.384	0.009	0.080
P63095	Guanine nucleotide-binding protein G(s) subunit alpha isoforms short OS=Rattus norvegicus GN=Gnas PE=1 SV=1 - [GNAS2_RAT]	45.6	0.013	0.013	0.013	0.013	0.013	0.013	0.013	0.013	0.013	0.013	1.266	1.386	2.135	3.059	1.185	0.013	0.013	0.013	0.013	0.013	#DIV/0!	#DIV/0!	0.009	0.036	#DIV/0!	#DIV/0!	0.009	0.028	#DIV/0!	#DIV/0!	0.009	0.074
P63100	Calcineurin subunit B type 1 OS=Rattus norvegicus GN=Ppp3r1 PE=1 SV=2 - [CANB1_RAT]	19.3	0.564	1.037	0.254	1.155	1.275	0.753	0.185	0.271	1.448	1.398	2.034	1.648	4.260	1.014	1.436	0.013	0.013	0.013	0.013	0.013	0.917	0.987	0.056	0.076	0.009	0.270	0.056	0.064	0.009	0.353	0.009	0.067
P63140	Nuclear transcription factor Y subunit beta OS=Rattus norvegicus OX=10116 GN=Nfyb PE=1 SV=1 - [NFYB_RAT]	22.8	0.013	0.013	0.013	0.013	0.013	0.013	0.013	0.013	0.013	0.013	1.350	1.315	3.053	3.606	1.023	0.013	0.013	0.013	0.013	0.013	#DIV/0!	#DIV/0!	0.009	0.031	#DIV/0!	#DIV/0!	0.009	0.024	#DIV/0!	#DIV/0!	0.009	0.068
P70712	Kynureninase OS=Rattus norvegicus OX=10116 GN=Kynu PE=1 SV=2 - [KYNU_RAT]	52.4	0.013	0.013	0.013	0.013	0.013	0.013	0.013	0.013	0.013	0.013	0.767	0.850	1.678	3.527	0.827	0.013	0.013	0.013	0.013	0.013	#DIV/0!	#DIV/0!	0.009	0.050	#DIV/0!	#DIV/0!	0.009	0.041	#DIV/0!	#DIV/0!	0.009	0.090
P83953	Importin subunit alpha-5 OS=Rattus norvegicus GN=Kpna1 PE=1 SV=1 - [IMA5_RAT]	60.1	0.013	0.013	0.013	0.013	0.013	0.013	0.013	0.013	0.013	0.013	1.410	1.237	2.304	1.933	1.356	0.013	0.013	0.013	0.013	0.013	#DIV/0!	#DIV/0!	0.009	0.043	#DIV/0!	#DIV/0!	0.009	0.034	#DIV/0!	#DIV/0!	0.009	0.081
P84245	Histone H3.3 OS=Rattus norvegicus GN=H3f3b PE=1 SV=2 - [H33_RAT]	15.3	0.013	0.013	0.013	0.013	0.013	0.013	0.013	0.013	0.013	0.013	1.450	2.033	3.107	3.203	1.830	0.013	0.013	0.013	0.013	0.013	#DIV/0!	#DIV/0!	0.009	0.028	#DIV/0!	#DIV/0!	0.009	0.023	#DIV/0!	#DIV/0!	0.009	0.064
P97562	Peroxisomal acyl-coenzyme A oxidase 2 OS=Rattus norvegicus OX=10116 GN=Acox2 PE=1 SV=1 - [ACOX2_RAT]	76.7	0.013	0.013	0.013	0.013	0.013	0.013	0.013	0.013	0.013	0.013	0.013	0.013	0.013	0.013	0.013	9.919	0.724	2.362	2.187	2.287	#DIV/0!	#DIV/0!	#DIV/0!	#DIV/0!	0.009	0.028	#DIV/0!	#DIV/0!	0.009	0.022	0.009	0.060
Q06496	Sodium-dependent phosphate transport protein 2A OS=Rattus norvegicus OX=10116 GN=Slc34a1 PE=1 SV=1 - [NPT2A_RAT]	68.7	0.013	0.013	0.013	0.013	0.013	0.013	0.013	0.013	0.013	0.013	2.170	1.312	3.132	4.289	1.008	0.013	0.013	0.013	0.013	0.013	#DIV/0!	#DIV/0!	0.009	0.028	#DIV/0!	#DIV/0!	0.009	0.022	#DIV/0!	#DIV/0!	0.009	0.063
Q0ZHH6	Atlastin-3 OS=Rattus norvegicus OX=10116 GN=Atl3 PE=2 SV=2 - [ATLA3_RAT]	60.5	0.013	0.013	0.013	0.013	0.013	0.013	0.013	0.013	0.013	0.013	4.141	3.650	5.710	1.802	2.188	0.013	0.013	0.013	0.013	0.013	#DIV/0!	#DIV/0!	0.009	0.027	#DIV/0!	#DIV/0!	0.009	0.022	#DIV/0!	#DIV/0!	0.009	0.060
Q32Q06	AP-1 complex subunit mu-1 OS=Rattus norvegicus GN=Ap1m1 PE=1 SV=3 - [AP1M1_RAT]	48.5	0.013	0.013	0.013	0.013	0.013	0.013	0.013	0.013	0.013	0.013	1.046	1.160	2.442	2.160	1.091	0.013	0.013	0.013	0.013	0.013	#DIV/0!	#DIV/0!	0.009	0.047	#DIV/0!	#DIV/0!	0.009	0.038	#DIV/0!	#DIV/0!	0.009	0.086
Q3B8Q2	Eukaryotic initiation factor 4A-III OS=Rattus norvegicus GN=Eif4a3 PE=1 SV=1 - [IF4A3_RAT]	46.8	0.013	0.013	0.013	0.013	0.013	0.013	0.013	0.013	0.013	0.013	1.396	1.215	2.139	2.198	1.095	0.013	0.013	0.013	0.013	0.013	#DIV/0!	#DIV/0!	0.009	0.045	#DIV/0!	#DIV/0!	0.009	0.036	#DIV/0!	#DIV/0!	0.009	0.084
Q4FZT0	Stomatin-like protein 2, mitochondrial OS=Rattus norvegicus OX=10116 GN=Stoml2 PE=1 SV=1 - [STML2_RAT]	38.4	0.013	0.013	0.013	0.013	0.013	0.013	0.013	0.013	0.013	0.013	0.972	0.977	2.541	3.338	1.194	0.013	0.013	0.013	0.013	0.013	#DIV/0!	#DIV/0!	0.009	0.036	#DIV/0!	#DIV/0!	0.009	0.029	#DIV/0!	#DIV/0!	0.009	0.074
Q4FZT2	Protein phosphatase methylesterase 1 OS=Rattus norvegicus GN=Ppme1 PE=1 SV=2 - [PPME1_RAT]	42.3	0.013	0.013	0.013	0.013	0.013	0.013	0.013	0.013	0.013	0.013	1.148	1.024	1.527	2.608	0.832	0.013	0.013	0.013	0.013	0.013	#DIV/0!	#DIV/0!	0.009	0.056	#DIV/0!	#DIV/0!	0.009	0.047	#DIV/0!	#DIV/0!	0.009	0.099
Q5BJQ0	Atypical kinase COQ8A, mitochondrial OS=Rattus norvegicus OX=10116 GN=Coq8a PE=2 SV=1 - [COQ8A_RAT]	72.2	0.013	0.013	0.013	0.013	0.013	0.013	0.013	0.013	0.013	0.013	1.289	1.234	2.789	2.435	1.151	0.013	0.013	0.013	0.013	0.013	#DIV/0!	#DIV/0!	0.009	0.038	#DIV/0!	#DIV/0!	0.009	0.030	#DIV/0!	#DIV/0!	0.009	0.075
Q5BJZ6	Carnosine N-methyltransferase OS=Rattus norvegicus OX=10116 GN=Carnmt1 PE=1 SV=1 - [CARME_RAT]	46.4	0.013	0.013	0.013	0.013	0.013	0.013	0.013	0.013	0.013	0.013	1.069	1.170	2.269	2.196	0.955	0.013	0.013	0.013	0.013	0.013	#DIV/0!	#DIV/0!	0.009	0.049	#DIV/0!	#DIV/0!	0.009	0.041	#DIV/0!	#DIV/0!	0.009	0.089
Q5FVL2	ER membrane protein complex subunit 8 OS=Rattus norvegicus OX=10116 GN=Emc8 PE=2 SV=1 - [EMC8_RAT]	23.4	0.673	1.130	0.497	1.340	1.215	1.351	1.235	0.881	0.562	0.769	1.118	1.056	2.100	2.346	1.229	0.013	0.013	0.013	0.013	0.013	0.756	0.908	0.259	0.261	0.009	0.131	0.184	0.154	0.009	0.105	0.009	0.087
Q5M827	Pirin OS=Rattus norvegicus GN=Pir PE=1 SV=1 - [PIR_RAT]	32.2	0.013	0.013	0.013	0.013	0.013	0.013	0.013	0.013	0.013	0.013	0.942	1.252	2.578	4.943	1.344	0.013	0.013	0.013	0.013	0.013	#DIV/0!	#DIV/0!	0.009	0.029	#DIV/0!	#DIV/0!	0.009	0.023	#DIV/0!	#DIV/0!	0.009	0.065
Q5M886	Manganese-dependent ADP-ribose/CDP-alcohol diphosphatase OS=Rattus norvegicus OX=10116 GN=Adprm PE=1 SV=1 - [ADPRM_RAT]	38.7	0.013	0.013	0.013	0.013	0.013	0.013	0.013	0.013	0.013	0.013	1.169	1.261	2.718	1.953	1.578	0.013	0.013	0.013	0.013	0.013	#DIV/0!	#DIV/0!	0.009	0.040	#DIV/0!	#DIV/0!	0.009	0.031	#DIV/0!	#DIV/0!	0.009	0.077
Q5PPH0	Enolase-phosphatase E1 OS=Rattus norvegicus GN=Enoph1 PE=2 SV=1 - [ENOPH_RAT]	28.9	0.013	0.013	0.013	0.013	0.013	0.013	0.013	0.013	0.013	0.013	1.365	1.250	3.311	2.118	2.597	0.013	0.013	0.013	0.013	0.013	#DIV/0!	#DIV/0!	0.009	0.030	#DIV/0!	#DIV/0!	0.009	0.024	#DIV/0!	#DIV/0!	0.009	0.066
Q5PPJ9	Endophilin-B2 OS=Rattus norvegicus GN=Sh3glb2 PE=2 SV=2 - [SHLB2_RAT]	44.8	0.013	0.013	0.013	0.013	0.013	0.013	0.013	0.013	0.013	0.013	1.068	1.170	2.274	1.680	1.113	0.013	0.013	0.013	0.013	0.013	#DIV/0!	#DIV/0!	0.009	0.053	#DIV/0!	#DIV/0!	0.009	0.045	#DIV/0!	#DIV/0!	0.009	0.096
Q5U1Y1	Ras-related protein Rab-34 OS=Rattus norvegicus OX=10116 GN=Rab34 PE=2 SV=1 - [RAB34_RAT]	29.1	0.013	0.013	0.013	0.013	0.013	0.013	0.013	0.013	0.013	0.013	0.975	0.825	1.757	1.946	1.628	0.013	0.013	0.013	0.013	0.013	#DIV/0!	#DIV/0!	0.009	0.056	#DIV/0!	#DIV/0!	0.009	0.047	#DIV/0!	#DIV/0!	0.009	0.099
Q5U2X6	Coiled-coil domain-containing protein 47 OS=Rattus norvegicus OX=10116 GN=Ccdc47 PE=2 SV=1 - [CCD47_RAT]	55.7	0.013	0.013	0.013	0.013	0.013	0.013	0.013	0.013	0.013	0.013	2.110	2.377	6.507	3.068	1.203	0.013	0.013	0.013	0.013	0.013	#DIV/0!	#DIV/0!	0.009	0.027	#DIV/0!	#DIV/0!	0.009	0.022	#DIV/0!	#DIV/0!	0.009	0.061
Q5XIA3	tRNA wybutosine-synthesizing protein 4 OS=Rattus norvegicus OX=10116 GN=Lcmt2 PE=2 SV=1 - [TYW4_RAT]	75.5	1.374	0.971	0.512	0.672	0.621	0.971	0.414	0.862	1.158	0.680	2.031	2.296	1.657	1.105	1.300	0.013	0.013	0.013	0.013	0.013	0.756	1.019	0.036	0.056	0.009	0.394	0.022	0.037	0.009	0.304	0.009	0.080
Q62725	Nuclear transcription factor Y subunit gamma OS=Rattus norvegicus OX=10116 GN=Nfyc PE=2 SV=1 - [NFYC_RAT]	37.2	0.013	0.013	0.013	0.013	0.013	0.013	0.013	0.013	0.013	0.013	1.228	1.089	2.066	1.735	1.211	0.013	0.013	0.013	0.013	0.013	#DIV/0!	#DIV/0!	0.009	0.052	#DIV/0!	#DIV/0!	0.009	0.044	#DIV/0!	#DIV/0!	0.009	0.094
Q63083	Nucleobindin-1 OS=Rattus norvegicus OX=10116 GN=Nucb1 PE=1 SV=1 - [NUCB1_RAT]	53.5	0.013	0.013	0.013	0.013	0.013	0.013	0.013	0.013	0.013	0.013	1.516	1.625	3.246	3.124	1.561	0.013	0.013	0.013	0.013	0.013	#DIV/0!	#DIV/0!	0.009	0.029	#DIV/0!	#DIV/0!	0.009	0.023	#DIV/0!	#DIV/0!	0.009	0.065
Q63487	Ras-related GTP-binding protein B OS=Rattus norvegicus GN=RragB PE=1 SV=1 - [RRAGB_RAT]	43.2	0.013	0.013	0.013	0.013	0.013	0.013	0.013	0.013	0.013	0.013	1.738	1.858	3.212	1.369	1.575	0.013	0.013	0.013	0.013	0.013	#DIV/0!	#DIV/0!	0.009	0.032	#DIV/0!	#DIV/0!	0.009	0.025	#DIV/0!	#DIV/0!	0.009	0.071
Q641Z7	Acid sphingomyelinase-like phosphodiesterase 3a OS=Rattus norvegicus OX=10116 GN=Smpdl3a PE=2 SV=1 - [ASM3A_RAT]	49.9	0.013	0.013	0.013	0.013	0.013	0.013	0.013	0.013	0.013	0.013	2.281	1.932	4.136	1.384	1.068	0.013	0.013	0.013	0.013	0.013	#DIV/0!	#DIV/0!	0.009	0.030	#DIV/0!	#DIV/0!	0.009	0.024	#DIV/0!	#DIV/0!	0.009	0.066
Q64232	Very-long-chain enoyl-CoA reductase OS=Rattus norvegicus OX=10116 GN=Tecr PE=1 SV=1 - [TECR_RAT]	36.1	0.013	0.013	0.013	0.013	0.013	0.013	0.013	0.013	0.013	0.013	0.980	2.499	1.257	1.716	0.761	0.013	0.013	0.013	0.013	0.013	#DIV/0!	#DIV/0!	0.009	0.054	#DIV/0!	#DIV/0!	0.009	0.046	#DIV/0!	#DIV/0!	0.009	0.098
Q642A4	UPF0598 protein C8orf82 homolog OS=Rattus norvegicus OX=10116 PE=2 SV=1 - [CH082_RAT]	24.4	0.678	0.732	0.673	0.856	0.733	0.013	0.013	0.013	0.013	0.013	1.023	0.938	1.845	3.713	1.126	0.013	0.013	0.013	0.013	0.013	0.009	0.372	0.014	0.031	0.009	0.732	0.009	0.032	#DIV/0!	#DIV/0!	0.009	0.078
Q66H15	Regulator of microtubule dynamics protein 3 OS=Rattus norvegicus OX=10116 GN=Rmdn3 PE=1 SV=1 - [RMD3_RAT]	52.3	0.013	0.013	0.013	0.013	0.013	0.013	0.013	0.013	0.013	0.013	0.013	0.013	0.013	0.013	0.013	6.681	0.298	1.768	1.736	1.831	#DIV/0!	#DIV/0!	#DIV/0!	#DIV/0!	0.009	0.029	#DIV/0!	#DIV/0!	0.009	0.022	0.009	0.063
Q66H59	N-acetylneuraminate lyase OS=Rattus norvegicus OX=10116 GN=Npl PE=2 SV=1 - [NPL_RAT]	35.1	0.013	0.013	0.013	0.013	0.013	0.013	0.013	0.013	0.013	0.013	1.111	1.315	1.522	3.636	0.566	0.013	0.013	0.013	0.013	0.013	#DIV/0!	#DIV/0!	0.009	0.043	#DIV/0!	#DIV/0!	0.009	0.035	#DIV/0!	#DIV/0!	0.009	0.082
Q6AXQ5	2',5'-phosphodiesterase 12 OS=Rattus norvegicus OX=10116 GN=Pde12 PE=2 SV=1 - [PDE12_RAT]	67.1	0.013	0.013	0.013	0.013	0.013	0.013	0.013	0.013	0.013	0.013	3.360	2.691	6.613	4.173	2.645	0.013	0.013	0.013	0.013	0.013	#DIV/0!	#DIV/0!	0.009	0.027	#DIV/0!	#DIV/0!	0.009	0.022	#DIV/0!	#DIV/0!	0.009	0.060
Q6AYB4	Heat shock 70 kDa protein 14 OS=Rattus norvegicus OX=10116 GN=Hspa14 PE=2 SV=1 - [HSP7E_RAT]	54.4	0.013	0.013	0.013	0.013	0.013	0.013	0.013	0.013	0.013	0.013	2.112	2.426	3.985	5.039	1.201	0.013	0.013	0.013	0.013	0.013	#DIV/0!	#DIV/0!	0.009	0.027	#DIV/0!	#DIV/0!	0.009	0.022	#DIV/0!	#DIV/0!	0.009	0.061
Q6EV70	GDP-fucose protein O-fucosyltransferase 1 OS=Rattus norvegicus GN=Pofut1 PE=2 SV=1 - [OFUT1_RAT]	45.0	0.013	0.013	0.013	0.013	0.013	0.814	0.261	0.434	0.978	1.135	1.320	1.325	2.463	2.089	1.489	0.013	0.013	0.013	0.013	0.013	0.009	0.399	0.009	0.039	#DIV/0!	#DIV/0!	0.014	0.027	0.009	0.802	0.009	0.077
Q6IG12	Keratin, type II cytoskeletal 7 OS=Rattus norvegicus GN=Krt7 PE=3 SV=1 - [K2C7_RAT]	50.7	0.013	0.013	0.013	0.013	0.013	0.013	0.013	0.013	0.013	0.013	0.013	0.013	0.013	0.013	0.013	6.687	0.204	0.911	0.854	0.775	#DIV/0!	#DIV/0!	#DIV/0!	#DIV/0!	0.009	0.031	#DIV/0!	#DIV/0!	0.009	0.024	0.009	0.072
Q6MG08	ATP-binding cassette sub-family F member 1 OS=Rattus norvegicus OX=10116 GN=Abcf1 PE=1 SV=1 - [ABCF1_RAT]	95.2	0.013	0.013	0.013	0.013	0.013	0.013	0.013	0.013	0.013	0.013	0.013	0.013	0.013	0.013	0.013	2.433	0.202	2.762	1.749	1.082	#DIV/0!	#DIV/0!	#DIV/0!	#DIV/0!	0.009	0.036	#DIV/0!	#DIV/0!	0.009	0.028	0.009	0.082
Q76IC5	Pyroglutamyl-peptidase 1 OS=Rattus norvegicus OX=10116 GN=Pgpep1 PE=1 SV=1 - [PGPI_RAT]	22.9	0.013	0.013	0.013	0.013	0.013	0.013	0.013	0.013	0.013	0.013	0.013	0.013	0.013	0.013	0.013	4.618	0.294	1.073	1.064	1.081	#DIV/0!	#DIV/0!	#DIV/0!	#DIV/0!	0.009	0.037	#DIV/0!	#DIV/0!	0.009	0.028	0.009	0.083
Q80Z70	Protein sel-1 homolog 1 OS=Rattus norvegicus OX=10116 GN=Sel1l PE=1 SV=2 - [SE1L1_RAT]	88.6	0.013	0.013	0.013	0.013	0.013	0.013	0.013	0.013	0.013	0.013	1.866	1.925	3.499	3.386	1.701	0.013	0.013	0.013	0.013	0.013	#DIV/0!	#DIV/0!	0.009	0.028	#DIV/0!	#DIV/0!	0.009	0.022	#DIV/0!	#DIV/0!	0.009	0.063
Q810F4	Protein FAM3C OS=Rattus norvegicus GN=Fam3c PE=2 SV=1 - [FAM3C_RAT]	24.7	0.751	0.785	0.554	1.055	0.912	1.200	0.676	0.800	1.076	1.037	1.284	1.029	2.490	1.878	0.827	0.013	0.013	0.013	0.013	0.013	0.259	0.883	0.056	0.080	0.009	0.446	0.184	0.154	0.009	0.106	0.009	0.092
Q811M5	Complement component C6 OS=Rattus norvegicus GN=C6 PE=2 SV=1 - [CO6_RAT]	105.0	0.013	0.013	0.013	0.013	0.013	0.013	0.013	0.013	0.013	0.013	0.013	0.013	0.013	0.013	0.013	3.017	0.892	1.475	1.150	1.096	#DIV/0!	#DIV/0!	#DIV/0!	#DIV/0!	0.009	0.041	#DIV/0!	#DIV/0!	0.009	0.032	0.009	0.090
Q8CIN7	Inositol monophosphatase 2 OS=Rattus norvegicus OX=10116 GN=Impa2 PE=2 SV=1 - [IMPA2_RAT]	31.8	0.013	0.013	0.013	0.013	0.013	0.013	0.013	0.013	0.013	0.013	1.074	1.496	2.549	2.993	1.235	0.013	0.013	0.013	0.013	0.013	#DIV/0!	#DIV/0!	0.009	0.034	#DIV/0!	#DIV/0!	0.009	0.027	#DIV/0!	#DIV/0!	0.009	0.073
Q8K3E7	Protein dpy-30 homolog OS=Rattus norvegicus OX=10116 GN=Dpy30 PE=1 SV=1 - [DPY30_RAT]	11.2	0.013	0.013	0.013	0.013	0.013	0.013	0.013	0.013	0.013	0.013	2.500	1.864	4.595	2.161	1.383	0.013	0.013	0.013	0.013	0.013	#DIV/0!	#DIV/0!	0.009	0.028	#DIV/0!	#DIV/0!	0.009	0.022	#DIV/0!	#DIV/0!	0.009	0.062
Q8K3X8	Heat shock factor-binding protein 1 OS=Rattus norvegicus OX=10116 GN=Hsbp1 PE=3 SV=1 - [HSBP1_RAT]	8.6	0.013	0.013	0.013	0.013	0.013	0.013	0.013	0.013	0.013	0.013	1.381	1.483	2.411	2.969	1.192	0.013	0.013	0.013	0.013	0.013	#DIV/0!	#DIV/0!	0.009	0.034	#DIV/0!	#DIV/0!	0.009	0.027	#DIV/0!	#DIV/0!	0.009	0.072
Q8R491	EH domain-containing protein 3 OS=Rattus norvegicus GN=Ehd3 PE=1 SV=2 - [EHD3_RAT]	60.8	0.013	0.013	0.013	0.013	0.013	0.013	0.013	0.013	0.013	0.013	0.013	0.013	0.013	0.013	0.013	4.388	0.404	1.773	2.052	1.292	#DIV/0!	#DIV/0!	#DIV/0!	#DIV/0!	0.009	0.030	#DIV/0!	#DIV/0!	0.009	0.023	0.009	0.069
Q9JK11	Reticulon-4 OS=Rattus norvegicus GN=Rtn4 PE=1 SV=1 - [RTN4_RAT]	126.3	1.071	0.811	0.762	0.973	0.781	1.680	0.711	0.705	0.864	0.558	1.094	1.067	1.887	3.941	0.830	0.013	0.013	0.013	0.013	0.013	0.354	0.920	0.056	0.077	0.009	0.233	0.085	0.085	0.009	0.136	0.009	0.075
Q9WVA1	Mitochondrial import inner membrane translocase subunit Tim8 A OS=Rattus norvegicus GN=Timm8a PE=1 SV=1 - [TIM8A_RAT]	11.0	0.280	1.197	0.198	1.031	0.856	0.013	0.013	0.013	0.013	0.013	1.200	1.001	2.342	2.231	0.982	0.013	0.013	0.013	0.013	0.013	0.009	0.469	0.085	0.112	0.009	0.789	0.009	0.040	#DIV/0!	#DIV/0!	0.009	0.088
