# Supplementary material for: Fetal growth restriction followed by early catch-up growth impairs pancreatic islet morphology in male rats
Source: Sci Rep. 2023 Feb 15;13:2732. doi: 10.1038/s41598-023-28584-2 (PMC9932152; doi:10.1038/s41598-023-28584-2)
Supplement: Supplementary file 3 — Supplementary Information 3. [file 41598_2023_28584_MOESM3_ESM.pdf]

Table S13: B-I Male_Effect of FGR (common)

Rank	Score	Category	Term ID	Term name	Count	%	PValue	List Total	Pop Hits	Pop Total	Fold Enrichment	Bonferroni	Benjamini	FDR
Cluster 1	6.03	Cellular component	GO:0031988	membrane-bounded vesicle	45	47.9	0.0000	86	3321	13535	2.13	0.0000	0.0000	0.0001
		Cellular component	GO:0070062	extracellular exosome	39	41.5	0.0000	86	2646	13535	2.32	0.0000	0.0000	0.0002
		Cellular component	GO:1903561	extracellular vesicle	39	41.5	0.0000	86	2661	13535	2.31	0.0000	0.0000	0.0002
		Cellular component	GO:0043230	extracellular organelle	39	41.5	0.0000	86	2666	13535	2.30	0.0001	0.0000	0.0002
		Cellular component	GO:0044421	extracellular region part	42	44.7	0.0000	86	3710	13535	1.78	0.0123	0.0021	0.0457
		Cellular component	GO:0005576	extracellular region	43	45.7	0.0002	86	4113	13535	1.65	0.0640	0.0073	0.2445
Cluster 2	3.46	Cellular component	GO:0099512	supramolecular fiber	16	17.0	0.0000	86	581	13535	4.33	0.0011	0.0002	0.0040
		Cellular component	GO:0099513	polymeric cytoskeletal fiber	16	17.0	0.0000	86	581	13535	4.33	0.0011	0.0002	0.0040
		Cellular component	GO:0005874	microtubule	11	11.7	0.0000	86	332	13535	5.21	0.0156	0.0022	0.0582
		Cellular component	GO:0015630	microtubule cytoskeleton	17	18.1	0.0007	86	1051	13535	2.55	0.2424	0.0249	1.0224
		Biological process	GO:0007017	microtubule-based process	10	10.6	0.0045	89	629	17375	3.10	1.0000	0.1846	7.6175
		Cellular component	GO:0005815	microtubule organizing center	10	10.6	0.0150	86	616	13535	2.55	0.9964	0.2016	18.8051
		Cellular component	GO:0005813	centrosome	8	8.5	0.0372	86	499	13535	2.52	1.0000	0.3173	40.7077
Cluster 3	2.26	Cellular component	GO:0005912	adherens junction	14	14.9	0.0002	86	643	13535	3.43	0.0632	0.0081	0.2412
		Cellular component	GO:0070161	anchoring junction	14	14.9	0.0002	86	658	13535	3.35	0.0786	0.0081	0.3024
		Cellular component	GO:0030054	cell junction	19	20.2	0.0010	86	1293	13535	2.31	0.2991	0.0292	1.3071
		Cellular component	GO:0005925	focal adhesion	9	9.6	0.0034	86	396	13535	3.58	0.7169	0.0807	4.5634
		Cellular component	GO:0005924	cell-substrate adherens junction	9	9.6	0.0036	86	401	13535	3.53	0.7438	0.0816	4.9156
		Cellular component	GO:0030055	cell-substrate junction	9	9.6	0.0039	86	406	13535	3.49	0.7695	0.0783	5.2876
		Molecular function	GO:0098641	cadherin binding involved in cell-cell adhesion	6	6.4	0.0062	88	220	16438	5.09	0.8859	0.1269	8.1911
		Biological process	GO:0007155	cell adhesion	16	17.0	0.0066	89	1468	17375	2.13	1.0000	0.2240	10.9374
		Biological process	GO:0022610	biological adhesion	16	17.0	0.0070	89	1478	17375	2.11	1.0000	0.2297	11.5865
		Molecular function	GO:0098632	protein binding involved in cell-cell adhesion	6	6.4	0.0071	88	227	16438	4.94	0.9156	0.1283	9.2724
		Molecular function	GO:0098631	protein binding involved in cell adhesion	6	6.4	0.0078	88	232	16438	4.83	0.9331	0.1327	10.0979
		Molecular function	GO:0045296	cadherin binding	6	6.4	0.0094	88	243	16438	4.61	0.9619	0.1508	12.0721
		Cellular component	GO:0005913	cell-cell adherens junction	6	6.4	0.0271	86	269	13535	3.51	1.0000	0.2972	31.5207
		Cellular component	GO:0005911	cell-cell junction	9	9.6	0.0371	86	609	13535	2.33	1.0000	0.3243	40.6871
		Biological process	GO:0098609	cell-cell adhesion	11	11.7	0.0415	89	1060	17375	2.03	1.0000	0.5239	52.2913
		Molecular function	GO:0050839	cell adhesion molecule binding	6	6.4	0.0574	88	393	16438	2.85	1.0000	0.3726	55.4012
Cluster 4	2.22	Biological process	GO:0051641	cellular localization	31	33.0	0.0000	89	2323	17375	2.61	0.0013	0.0013	0.0010
		Biological process	GO:0046907	intracellular transport	21	22.3	0.0000	89	1303	17375	3.15	0.0139	0.0046	0.0106
		Biological process	GO:0051649	establishment of localization in cell	24	25.5	0.0000	89	1724	17375	2.72	0.0245	0.0062	0.0189
		Biological process	GO:0051049	regulation of transport	24	25.5	0.0000	89	1860	17375	2.52	0.0810	0.0140	0.0643
		Biological process	GO:0032386	regulation of intracellular transport	11	11.7	0.0002	89	483	17375	4.45	0.3118	0.0456	0.2841
		Biological process	GO:0060341	regulation of cellular localization	14	14.9	0.0004	89	873	17375	3.13	0.6298	0.0602	0.7536
		Biological process	GO:0006886	intracellular protein transport	14	14.9	0.0006	89	901	17375	3.03	0.7379	0.0590	1.0140
		Biological process	GO:0034613	cellular protein localization	18	19.1	0.0013	89	1505	17375	2.33	0.9497	0.0949	2.2506
		Biological process	GO:0070727	cellular macromolecule localization	18	19.1	0.0014	89	1517	17375	2.32	0.9617	0.0969	2.4518
		Biological process	GO:0015031	protein transport	18	19.1	0.0025	89	1602	17375	2.19	0.9971	0.1426	4.3532
		Biological process	GO:0033157	regulation of intracellular protein transport	8	8.5	0.0032	89	381	17375	4.10	0.9994	0.1605	5.4391
		Biological process	GO:0046822	regulation of nucleocytoplasmic transport	6	6.4	0.0055	89	223	17375	5.25	1.0000	0.2058	9.1947
		Biological process	GO:0051223	regulation of protein transport	11	11.7	0.0059	89	778	17375	2.76	1.0000	0.2151	9.8114
		Biological process	GO:0033365	protein localization to organelle	11	11.7	0.0066	89	791	17375	2.71	1.0000	0.2271	10.9247
		Biological process	GO:0045184	establishment of protein localization	18	19.1	0.0070	89	1770	17375	1.99	1.0000	0.2324	11.5569
		Biological process	GO:1903827	regulation of cellular protein localization	9	9.6	0.0080	89	567	17375	3.10	1.0000	0.2536	13.0848
		Biological process	GO:0070201	regulation of establishment of protein localization	11	11.7	0.0094	89	834	17375	2.57	1.0000	0.2667	15.2376
		Biological process	GO:0046825	regulation of protein export from nucleus	3	3.2	0.0121	89	33	17375	17.75	1.0000	0.2916	19.1485
		Biological process	GO:0051222	positive regulation of protein transport	8	8.5	0.0126	89	494	17375	3.16	1.0000	0.2964	19.9193
		Biological process	GO:0051050	positive regulation of transport	12	12.8	0.0132	89	1014	17375	2.31	1.0000	0.3048	20.7403
		Biological process	GO:1904951	positive regulation of establishment of protein localization	8	8.5	0.0179	89	530	17375	2.95	1.0000	0.3634	27.1093
		Biological process	GO:0032880	regulation of protein localization	11	11.7	0.0287	89	995	17375	2.16	1.0000	0.4519	39.8373
		Biological process	GO:0006611	protein export from nucleus	3	3.2	0.0296	89	53	17375	11.05	1.0000	0.4594	40.8102
		Biological process	GO:0033036	macromolecule localization	21	22.3	0.0308	89	2573	17375	1.59	1.0000	0.4646	42.1250
		Biological process	GO:0008104	protein localization	19	20.2	0.0322	89	2255	17375	1.64	1.0000	0.4706	43.5215
		Biological process	GO:1903829	positive regulation of cellular protein localization	6	6.4	0.0360	89	360	17375	3.25	1.0000	0.4929	47.3200
		Biological process	GO:0090316	positive regulation of intracellular protein transport	5	5.3	0.0451	89	264	17375	3.70	1.0000	0.5412	55.3573
		Biological process	GO:0032388	positive regulation of intracellular transport	5	5.3	0.0653	89	299	17375	3.26	1.0000	0.6206	69.2807
		Biological process	GO:0072594	establishment of protein localization to organelle	6	6.4	0.1411	89	543	17375	2.16	1.0000	0.7851	92.9843
		Biological process	GO:0051168	nuclear export	3	3.2	0.1512	89	136	17375	4.31	1.0000	0.7997	94.2965
		Biological process	GO:0006913	nucleocytoplasmic transport	5	5.3	0.1525	89	408	17375	2.39	1.0000	0.8013	94.4471
		Biological process	GO:0051169	nuclear transport	5	5.3	0.1573	89	413	17375	2.36	1.0000	0.8066	94.9672
		Biological process	GO:0006605	protein targeting	6	6.4	0.1923	89	605	17375	1.94	1.0000	0.8471	97.6037
		Biological process	GO:1903533	regulation of protein targeting	4	4.3	0.2037	89	307	17375	2.54	1.0000	0.8537	98.1307
		Biological process	GO:0034504	protein localization to nucleus	4	4.3	0.2846	89	367	17375	2.13	1.0000	0.9047	99.7124
Cluster 5	2.21	Biological process	GO:0051640	organelle localization	13	13.8	0.0000	89	446	17375	5.69	0.0057	0.0028	0.0043
		Biological process	GO:0051656	establishment of organelle localization	10	10.6	0.0001	89	368	17375	5.31	0.2084	0.0328	0.1777
		Biological process	GO:0051650	establishment of vesicle localization	7	7.4	0.0004	89	185	17375	7.39	0.5556	0.0563	0.6154
		Biological process	GO:0051648	vesicle localization	7	7.4	0.0005	89	198	17375	6.90	0.6875	0.0661	0.8814
		Biological process	GO:0016192	vesicle-mediated transport	15	16.0	0.0022	89	1171	17375	2.50	0.9932	0.1331	3.7328
		Biological process	GO:0060627	regulation of vesicle-mediated transport	8	8.5	0.0091	89	463	17375	3.37	1.0000	0.2681	14.7142
		Biological process	GO:0032940	secretion by cell	11	11.7	0.0198	89	936	17375	2.29	1.0000	0.3889	29.4337
		Biological process	GO:0006887	exocytosis	6	6.4	0.0204	89	309	17375	3.79	1.0000	0.3916	30.1842
		Biological process	GO:1903530	regulation of secretion by cell	9	9.6	0.0271	89	709	17375	2.48	1.0000	0.4425	38.1407
		Cellular component	GO:0016023	cytoplasmic, membrane-bounded vesicle	12	12.8	0.0350	86	950	13535	1.99	1.0000	0.3313	38.8290
		Biological process	GO:0051046	regulation of secretion	9	9.6	0.0388	89	761	17375	2.31	1.0000	0.5110	49.9254
		Biological process	GO:0046903	secretion	11	11.7	0.0506	89	1098	17375	1.96	1.0000	0.5602	59.6117
		Cellular component	GO:0005768	endosome	9	9.6	0.0578	86	667	13535	2.12	1.0000	0.3764	56.0257
		Biological process	GO:0017157	regulation of exocytosis	4	4.3	0.0694	89	187	17375	4.18	1.0000	0.6344	71.5200
		Biological process	GO:0045055	regulated exocytosis	3	3.2	0.2578	89	194	17375	3.02	1.0000	0.8899	99.4523
Cluster 6	2.08	Molecular function	GO:0016462	pyrophosphatase activity	15	16.0	0.0000	88	749	16438	3.74	0.0125	0.0063	0.0494
		Molecular function	GO:0016818	hydrolase activity, acting on acid anhydrides, in phosphorus-containing anhydrides	15	16.0	0.0000	88	752	16438	3.73	0.0130	0.0044	0.0516
		Molecular function	GO:0016817	hydrolase activity, acting on acid anhydrides	15	16.0	0.0000	88	758	16438	3.70	0.0142	0.0036	0.0563
		Molecular function	GO:0017111	nucleoside-triphosphatase activity	14	14.9	0.0001	88	703	16438	3.72	0.0270	0.0046	0.1079
		Molecular function	GO:0003924	GTPase activity	7	7.4	0.0006	88	198	16438	6.60	0.1982	0.0272	0.8657
		Molecular function	GO:0019003	GDP binding	4	4.3	0.0039	88	60	16438	12.45	0.7461	0.1079	5.2525
		Molecular function	GO:0035639	purine ribonucleoside triphosphate binding	18	19.1	0.0097	88	1753	16438	1.92	0.9661	0.1488	12.4701
		Molecular function	GO:0032550	purine ribonucleoside binding	18	19.1	0.0102	88	1763	16438	1.91	0.9719	0.1499	13.1189
		Molecular function	GO:0001883	purine nucleoside binding	18	19.1	0.0104	88	1766	16438	1.90	0.9735	0.1460	13.3182
		Molecular function	GO:0032549	ribonucleoside binding	18	19.1	0.0104	88	1766	16438	1.90	0.9735	0.1460	13.3182
		Molecular function	GO:0001882	nucleoside binding	18	19.1	0.0108	88	1773	16438	1.90	0.9769	0.1454	13.7916
		Molecular function	GO:0032555	purine ribonucleotide binding	18	19.1	0.0123	88	1798	16438	1.87	0.9865	0.1525	15.5803
		Molecular function	GO:0017076	purine nucleotide binding	18	19.1	0.0130	88	1809	16438	1.86	0.9895	0.1502	16.4163
		Molecular function	GO:0032553	ribonucleotide binding	18	19.1	0.0134	88	1815	16438	1.85	0.9909	0.1496	16.8850
		Molecular function	GO:0005525	GTP binding	7	7.4	0.0154	88	380	16438	3.44	0.9955	0.1598	19.1377
		Molecular function	GO:0000166	nucleotide binding	21	22.3	0.0162	88	2307	16438	1.70	0.9965	0.1622	19.9826
		Molecular function	GO:1901265	nucleoside phosphate binding	21	22.3	0.0162	88	2307	16438	1.70	0.9965	0.1622	19.9826
		Molecular function	GO:0036094	small molecule binding	22	23.4	0.0195	88	2506	16438	1.64	0.9989	0.1867	23.5481
		Molecular function	GO:0032561	guanyl ribonucleotide binding	7	7.4	0.0198	88	402	16438	3.25	0.9990	0.1845	23.8905
		Molecular function	GO:0019001	guanyl nucleotide binding	7	7.4	0.0200	88	403	16438	3.24	0.9991	0.1815	24.1193
		Molecular function	GO:0097367	carbohydrate derivative binding	19	20.2	0.0313	88	2152	16438	1.65	1.0000	0.2409	35.2084
		Molecular function	GO:1901363	heterocyclic compound binding	36	38.3	0.1478	88	5645	16438	1.19	1.0000	0.6632	88.7514
		Molecular function	GO:0097159	organic cyclic compound binding	36	38.3	0.1717	88	5726	16438	1.17	1.0000	0.7086	92.3656
		Molecular function	GO:0005524	ATP binding	11	11.7	0.2065	88	1403	16438	1.46	1.0000	0.7554	95.7545

Molecular function	GO:0032559	adenyl ribonucleotide binding	11	11.7	0.2295	88	1440	16438	1.43	1.0000	0.7843	97.1616
Molecular function	GO:0030554	adenyl nucleotide binding	11	11.7	0.2353	88	1449	16438	1.42	1.0000	0.7881	97.4381

Table S14: D-I Female_Effect of FGR (common)

Rank	Score	Category	Term ID	Term name	Count	%	PValue	List Total	Pop Hits	Pop Total	Fold Enrichment	Bonferroni	Benjamini	FDR
Cluster 1	34.16	Cellular component	GO:0070062	extracellular exosome	141	56.0	0.0000	242	2646	13535	2.98	0.0000	0.0000	0.0000
		Cellular component	GO:1903561	extracellular vesicle	141	56.0	0.0000	242	2661	13535	2.96	0.0000	0.0000	0.0000
		Cellular component	GO:0043230	extracellular organelle	141	56.0	0.0000	242	2666	13535	2.96	0.0000	0.0000	0.0000
		Cellular component	GO:0031988	membrane-bounded vesicle	149	59.1	0.0000	242	3321	13535	2.51	0.0000	0.0000	0.0000
		Cellular component	GO:0044421	extracellular region part	149	59.1	0.0000	242	3710	13535	2.25	0.0000	0.0000	0.0000
		Cellular component	GO:0005576	extracellular region	151	59.9	0.0000	242	4113	13535	2.05	0.0000	0.0000	0.0000
Cluster 2	24.54	Biological process	GO:0019752	carboxylic acid metabolic process	60	23.8	0.0000	236	787	17375	5.61	0.0000	0.0000	0.0000
		Biological process	GO:0043436	oxoacid metabolic process	60	23.8	0.0000	236	791	17375	5.58	0.0000	0.0000	0.0000
		Biological process	GO:0006082	organic acid metabolic process	62	24.6	0.0000	236	875	17375	5.22	0.0000	0.0000	0.0000
		Biological process	GO:0032787	monocarboxylic acid metabolic process	40	15.9	0.0000	236	535	17375	5.50	0.0000	0.0000	0.0000
Cluster 3	10.86	Biological process	GO:0055114	oxidation-reduction process	46	18.3	0.0000	236	976	17375	3.47	0.0000	0.0000	0.0000
		Molecular function	GO:0048037	cofactor binding	26	10.3	0.0000	241	308	16438	5.76	0.0000	0.0000	0.0000
		Molecular function	GO:0050662	coenzyme binding	19	7.5	0.0000	241	212	16438	6.11	0.0000	0.0000	0.0000
Cluster 4	10.65	Cellular component	GO:0000323	lytic vacuole	33	13.1	0.0000	242	449	13535	4.11	0.0000	0.0000	0.0000
		Cellular component	GO:0005764	lysosome	33	13.1	0.0000	242	449	13535	4.11	0.0000	0.0000	0.0000
		Cellular component	GO:0005773	vacuole	51	20.2	0.0000	242	1018	13535	2.80	0.0000	0.0000	0.0000
Cluster 5	9.74	Biological process	GO:0032787	monocarboxylic acid metabolic process	40	15.9	0.0000	236	535	17375	5.50	0.0000	0.0000	0.0000
		Biological process	GO:0006629	lipid metabolic process	43	17.1	0.0000	236	1174	17375	2.70	0.0000	0.0000	0.0000
		Biological process	GO:0006631	fatty acid metabolic process	21	8.3	0.0000	236	354	17375	4.37	0.0003	0.0000	0.0001
		Biological process	GO:0044255	cellular lipid metabolic process	33	13.1	0.0000	236	896	17375	2.71	0.0020	0.0001	0.0009
Cluster 6	8.56	Molecular function	GO:0042802	identical protein binding	53	21.0	0.0000	241	1325	16438	2.73	0.0000	0.0000	0.0000
		Molecular function	GO:0042803	protein homodimerization activity	35	13.9	0.0000	241	805	16438	2.97	0.0000	0.0000	0.0000
		Molecular function	GO:0046983	protein dimerization activity	46	18.3	0.0000	241	1283	16438	2.45	0.0000	0.0000	0.0000
Cluster 7	7.84	Biological process	GO:0044283	small molecule biosynthetic process	30	11.9	0.0000	236	453	17375	4.88	0.0000	0.0000	0.0000
		Biological process	GO:0016053	organic acid biosynthetic process	21	8.3	0.0000	236	258	17375	5.99	0.0000	0.0000	0.0000
		Biological process	GO:0046394	carboxylic acid biosynthetic process	19	7.5	0.0000	236	242	17375	5.78	0.0000	0.0000	0.0000
		Biological process	GO:1901607	alpha-amino acid biosynthetic process	9	3.6	0.0000	236	73	17375	9.08	0.0249	0.0006	0.0117
		Biological process	GO:0008652	cellular amino acid biosynthetic process	8	3.2	0.0000	236	59	17375	9.98	0.0554	0.0011	0.0264
Cluster 8	7.73	Biological process	GO:0016052	carbohydrate catabolic process	16	6.3	0.0000	236	129	17375	9.13	0.0000	0.0000	0.0000
		Biological process	GO:0044724	single-organism carbohydrate catabolic process	15	6.0	0.0000	236	111	17375	9.95	0.0000	0.0000	0.0000
		Biological process	GO:0005975	carbohydrate metabolic process	34	13.5	0.0000	236	705	17375	3.55	0.0000	0.0000	0.0000
		Biological process	GO:0044723	single-organism carbohydrate metabolic process	26	10.3	0.0000	236	594	17375	3.22	0.0021	0.0001	0.0010
		Biological process	GO:0019318	hexose metabolic process	15	6.0	0.0000	236	212	17375	5.21	0.0048	0.0001	0.0022
		Biological process	GO:0005996	monosaccharide metabolic process	15	6.0	0.0000	236	222	17375	4.97	0.0082	0.0002	0.0038
Cluster 9	7.55	Biological process	GO:1901565	organonitrogen compound catabolic process	25	9.9	0.0000	236	272	17375	6.77	0.0000	0.0000	0.0000
		Biological process	GO:0044282	small molecule catabolic process	23	9.1	0.0000	236	270	17375	6.27	0.0000	0.0000	0.0000
		Biological process	GO:1901605	alpha-amino acid metabolic process	19	7.5	0.0000	236	194	17375	7.21	0.0000	0.0000	0.0000
		Biological process	GO:0016054	organic acid catabolic process	18	7.1	0.0000	236	192	17375	6.90	0.0000	0.0000	0.0000
		Biological process	GO:0006520	cellular amino acid metabolic process	17	6.7	0.0000	236	215	17375	5.82	0.0002	0.0000	0.0001
		Biological process	GO:0046395	carboxylic acid catabolic process	15	6.0	0.0000	236	169	17375	6.53	0.0003	0.0000	0.0001
		Biological process	GO:1901606	alpha-amino acid catabolic process	9	3.6	0.0000	236	62	17375	10.69	0.0072	0.0002	0.0033
		Biological process	GO:0009063	cellular amino acid catabolic process	9	3.6	0.0000	236	66	17375	10.04	0.0116	0.0003	0.0054
		Biological process	GO:0009064	glutamine family amino acid metabolic process	9	3.6	0.0000	236	72	17375	9.20	0.0224	0.0005	0.0105
		Biological process	GO:0009065	glutamine family amino acid catabolic process	4	1.6	0.0040	236	24	17375	12.27	1.0000	0.0937	7.1946
Cluster 10	6.74	Molecular function	GO:0008238	exopeptidase activity	17	6.7	0.0000	241	97	16438	11.95	0.0000	0.0000	0.0000
		Molecular function	GO:0070011	peptidase activity, acting on L-amino acid peptides	27	10.7	0.0000	241	651	16438	2.83	0.0029	0.0003	0.0052
		Molecular function	GO:0008233	peptidase activity	27	10.7	0.0000	241	688	16438	2.68	0.0078	0.0006	0.0141
		Biological process	GO:0006508	proteolysis	43	17.1	0.0001	236	1661	17375	1.91	0.1879	0.0032	0.0963
Cluster 11	5.59	Molecular function	GO:0016614	oxidoreductase activity, acting on CH-OH group of donors	14	5.6	0.0000	241	141	16438	6.77	0.0001	0.0000	0.0002
		Molecular function	GO:0016616	oxidoreductase activity, acting on the CH-OH group of donors, NAD or NADP as acceptor	12	4.8	0.0000	241	119	16438	6.88	0.0012	0.0001	0.0021
		Molecular function	GO:0051287	NAD binding	8	3.2	0.0001	241	72	16438	7.58	0.0704	0.0035	0.1318
Cluster 12	5.12	Biological process	GO:0043648	dicarboxylic acid metabolic process	13	5.2	0.0000	236	92	17375	10.40	0.0000	0.0000	0.0000
		Biological process	GO:0009064	glutamine family amino acid metabolic process	9	3.6	0.0000	236	72	17375	9.20	0.0224	0.0005	0.0105
		Biological process	GO:0006536	glutamate metabolic process	6	2.4	0.0000	236	30	17375	14.72	0.1717	0.0031	0.0871
		Biological process	GO:0009084	glutamine family amino acid biosynthetic process	4	1.6	0.0031	236	22	17375	13.39	1.0000	0.0780	5.6279
Cluster 13	3.52	Biological process	GO:0006461	protein complex assembly	38	15.1	0.0000	236	1268	17375	2.21	0.0289	0.0007	0.0136
		Biological process	GO:0070271	protein complex biogenesis	38	15.1	0.0000	236	1269	17375	2.20	0.0294	0.0006	0.0138
		Biological process	GO:0071822	protein complex subunit organization	40	15.9	0.0000	236	1407	17375	2.09	0.0517	0.0010	0.0246
		Biological process	GO:0065003	macromolecular complex assembly	42	16.7	0.0000	236	1563	17375	1.98	0.1066	0.0019	0.0522
		Biological process	GO:0022607	cellular component assembly	53	21.0	0.0006	236	2456	17375	1.59	0.8972	0.0214	1.0475
		Biological process	GO:0044085	cellular component biogenesis	54	21.4	0.0029	236	2705	17375	1.47	1.0000	0.0745	5.2363
		Biological process	GO:0043933	macromolecular complex subunit organization	45	17.9	0.0054	236	2213	17375	1.50	1.0000	0.1171	9.4957
		Biological process	GO:0051259	protein oligomerization	16	6.3	0.0055	236	527	17375	2.24	1.0000	0.1194	9.7340
		Biological process	GO:0034622	cellular macromolecular complex assembly	21	8.3	0.0217	236	906	17375	1.71	1.0000	0.3143	33.4319
Cluster 14	3.48	Biological process	GO:0051186	cofactor metabolic process	29	11.5	0.0000	236	373	17375	5.72	0.0000	0.0000	0.0000
		Biological process	GO:0006732	coenzyme metabolic process	26	10.3	0.0000	236	307	17375	6.24	0.0000	0.0000	0.0000
		Biological process	GO:0016052	carbohydrate catabolic process	16	6.3	0.0000	236	129	17375	9.13	0.0000	0.0000	0.0000
		Biological process	GO:0044724	single-organism carbohydrate catabolic process	15	6.0	0.0000	236	111	17375	9.95	0.0000	0.0000	0.0000
		Biological process	GO:0072524	pyridine-containing compound metabolic process	16	6.3	0.0000	236	135	17375	8.73	0.0000	0.0000	0.0000
		Biological process	GO:0046496	nicotinamide nucleotide metabolic process	15	6.0	0.0000	236	129	17375	8.56	0.0000	0.0000	0.0000
		Biological process	GO:0019362	pyridine nucleotide metabolic process	15	6.0	0.0000	236	129	17375	8.56	0.0000	0.0000	0.0000
		Biological process	GO:0006733	oxidoreduction coenzyme metabolic process	15	6.0	0.0000	236	145	17375	7.62	0.0000	0.0000	0.0000
		Biological process	GO:0055086	nucleobase-containing small molecule metabolic process	28	11.1	0.0000	236	672	17375	3.07	0.0018	0.0001	0.0008
		Biological process	GO:0019637	organophosphate metabolic process	33	13.1	0.0000	236	961	17375	2.53	0.0090	0.0002	0.0042
		Biological process	GO:0009117	nucleotide metabolic process	25	9.9	0.0000	236	613	17375	3.00	0.0128	0.0003	0.0059
		Biological process	GO:0006165	nucleoside diphosphate phosphorylation	10	4.0	0.0000	236	90	17375	8.18	0.0141	0.0004	0.0066
		Biological process	GO:0006753	nucleoside phosphate metabolic process	25	9.9	0.0000	236	620	17375	2.97	0.0154	0.0004	0.0072
		Biological process	GO:0046939	nucleotide phosphorylation	10	4.0	0.0000	236	97	17375	7.59	0.0262	0.0006	0.0123
		Biological process	GO:0006090	pyruvate metabolic process	10	4.0	0.0000	236	100	17375	7.36	0.0335	0.0007	0.0158
		Biological process	GO:1901657	glycosyl compound metabolic process	18	7.1	0.0000	236	380	17375	3.49	0.0672	0.0013	0.0322
		Biological process	GO:0009132	nucleoside diphosphate metabolic process	10	4.0	0.0000	236	115	17375	6.40	0.1004	0.0018	0.0490
		Biological process	GO:1901135	carbohydrate derivative metabolic process	32	12.7	0.0001	236	1072	17375	2.20	0.1882	0.0032	0.0965
		Biological process	GO:0046031	ADP metabolic process	8	3.2	0.0001	236	80	17375	7.36	0.3414	0.0059	0.1931
		Biological process	GO:0009205	purine ribonucleoside triphosphate metabolic process	13	5.2	0.0001	236	242	17375	3.95	0.3755	0.0063	0.2177
		Biological process	GO:0009199	ribonucleoside triphosphate metabolic process	13	5.2	0.0001	236	248	17375	3.86	0.4470	0.0078	0.2739
		Biological process	GO:0009144	purine nucleoside triphosphate metabolic process	13	5.2	0.0002	236	250	17375	3.83	0.4702	0.0082	0.2937
		Biological process	GO:0009135	purine nucleoside diphosphate metabolic process	8	3.2	0.0002	236	90	17375	6.54	0.5837	0.0108	0.4048
		Biological process	GO:0009179	purine ribonucleoside diphosphate metabolic process	8	3.2	0.0002	236	90	17375	6.54	0.5837	0.0108	0.4048
		Biological process	GO:0009185	ribonucleoside diphosphate metabolic process	8	3.2	0.0003	236	93	17375	6.33	0.6583	0.0124	0.4958
		Biological process	GO:0009141	nucleoside triphosphate metabolic process	13	5.2	0.0003	236	271	17375	3.53	0.7378	0.0148	0.6177
		Biological process	GO:0046128	purine ribonucleoside metabolic process	14	5.6	0.0004	236	314	17375	3.28	0.7672	0.0155	0.6724
		Biological process	GO:0009116	nucleoside metabolic process	15	6.0	0.0004	236	357	17375	3.09	0.7788	0.0159	0.6960
		Biological process	GO:0006096	glycolytic process	7	2.8	0.0004	236	71	17375	7.26	0.7953	0.0161	0.7317
		Biological process	GO:0042278	purine nucleoside metabolic process	14	5.6	0.0004	236	318	17375	3.24	0.8068	0.0165	0.7582
		Biological process	GO:0006757	ATP generation from ADP	7	2.8	0.0004	236	72	17375	7.16	0.8194	0.0170	0.7891
		Biological process	GO:0009119	ribonucleoside metabolic process	14	5.6	0.0007	236	338	17375	3.05	0.9453	0.0258	1.3363
		Biological process	GO:0090407	organophosphate biosynthetic process	16	6.3	0.0028	236	490	17375	2.40	1.0000	0.0737	5.1407
		Biological process	GO:0009167	purine ribonucleoside monophosphate metabolic process	11	4.4	0.0030	236	261	17375	3.10	1.0000	0.0768	5.4301
		Biological process	GO:0009126	purine nucleoside monophosphate metabolic process	11	4.4	0.0031	236	262	17375	3.09	1.0000	0.0780	5.5886
		Biological process	GO:0046034	ATP metabolic process	10	4.0	0.0034	236	223	17375	3.30	1.0000	0.0831	6.1485
		Biological process	GO:0046039	GTP metabolic process	4	1.6	0.0035	236	23	17375	12.80	1.0000	0.0857	6.3840
		Biological process	GO:0009161	ribonucleoside monophosphate metabolic process	11	4.4	0.0039	236	271	17375	2.99	1.0000	0.0928	7.0409
		Biological process	GO:0009123	nucleoside monophosphate metabolic process	11	4.4	0.0056	236	285	17375	2.84	1.0000	0.1201	9.8493
		Biological process	GO:1901068	guanosine-containing compound metabolic process	4	1.6	0.0145	236	38	17375	7.75	1.0000	0.2424	23.8468
		Biological process	GO:0072521	purine-containing compound metabolic process	15	6.0	0.0153	236	540	17375	2.05	1.0000	0.2516	24.9539
		Biological process	GO:0019693	ribose phosphate metabolic process	14	5.6	0.0191	236	501	17375	2.06	1.0000	0.2905	30.1691
		Biological process	GO:0009142	nucleoside triphosphate biosynthetic process	5	2.0	0.0232	236	80	17375	4.60	1.0000	0.3281	35.3560
		Biological process	GO:0009165	nucleotide biosynthetic process	10	4.0	0.0279	236	315	17375	2.34	1.0000	0.3534	40.9480
		Biological process	GO:1901293	nucleoside phosphate biosynthetic process	10	4.0	0.0288	236	317	17375	2.32	1.0000	0.3600	41.9196
		Biological process	GO:0009150	purine ribonucleotide metabolic process	13	5.2	0.0290	236	476	17375	2.01	1.0000	0.3606	42.1084

		Biological process	GO:0009259	ribonucleotide metabolic process	13	5.2	0.0350	236	490	17375	1.95	1.0000	0.3981	48.4543
		Biological process	GO:0006091	generation of precursor metabolites and energy	10	4.0	0.0359	236	330	17375	2.23	1.0000	0.4019	49.3687
		Biological process	GO:0046129	purine ribonucleoside biosynthetic process	5	2.0	0.0387	236	94	17375	3.92	1.0000	0.4212	52.0056
		Biological process	GO:0042451	purine nucleoside biosynthetic process	5	2.0	0.0387	236	94	17375	3.92	1.0000	0.4212	52.0056
		Biological process	GO:0006163	purine nucleotide metabolic process	13	5.2	0.0394	236	499	17375	1.92	1.0000	0.4222	52.5974
		Biological process	GO:0009206	purine ribonucleoside triphosphate biosynthetic process	4	1.6	0.0516	236	62	17375	4.75	1.0000	0.4836	62.6574
		Biological process	GO:0009145	purine nucleoside triphosphate biosynthetic process	4	1.6	0.0537	236	63	17375	4.67	1.0000	0.4942	64.1363
		Biological process	GO:0009201	ribonucleoside triphosphate biosynthetic process	4	1.6	0.0645	236	68	17375	4.33	1.0000	0.5463	71.0704
		Biological process	GO:0042455	ribonucleoside biosynthetic process	5	2.0	0.0673	236	113	17375	3.26	1.0000	0.5568	72.6368
		Biological process	GO:0009163	nucleoside biosynthetic process	5	2.0	0.0673	236	113	17375	3.26	1.0000	0.5568	72.6368
		Biological process	GO:1901659	glycosyl compound biosynthetic process	5	2.0	0.0708	236	115	17375	3.20	1.0000	0.5728	74.4896
		Biological process	GO:0009127	purine nucleoside monophosphate biosynthetic process	4	1.6	0.0890	236	78	17375	3.78	1.0000	0.6257	82.3286
		Biological process	GO:0009168	purine ribonucleoside monophosphate biosynthetic process	4	1.6	0.0890	236	78	17375	3.78	1.0000	0.6257	82.3286
		Biological process	GO:0009156	ribonucleoside monophosphate biosynthetic process	4	1.6	0.1168	236	88	17375	3.35	1.0000	0.6889	90.0550
		Biological process	GO:0009124	nucleoside monophosphate biosynthetic process	4	1.6	0.1441	236	97	17375	3.04	1.0000	0.7376	94.4577
		Biological process	GO:0046390	ribose phosphate biosynthetic process	6	2.4	0.2758	236	260	17375	1.70	1.0000	0.8892	99.7519
		Biological process	GO:0009152	purine ribonucleotide biosynthetic process	5	2.0	0.4204	236	245	17375	1.50	1.0000	0.9545	99.9961
		Biological process	GO:0006164	purine nucleotide biosynthetic process	5	2.0	0.4576	236	255	17375	1.44	1.0000	0.9635	99.9989
		Biological process	GO:0009260	ribonucleotide biosynthetic process	5	2.0	0.4599	236	257	17375	1.43	1.0000	0.9637	99.9989
		Biological process	GO:0072522	purine-containing compound biosynthetic process	5	2.0	0.4713	236	259	17375	1.42	1.0000	0.9663	99.9993
Cluster 15	3.45	Biological process	GO:0009991	response to extracellular stimulus	23	9.1	0.0000	236	630	17375	2.69	0.1748	0.0030	0.0889
		Biological process	GO:0031667	response to nutrient levels	22	8.7	0.0001	236	603	17375	2.69	0.2579	0.0043	0.1380
		Biological process	GO:0007584	response to nutrient	14	5.6	0.0003	236	304	17375	3.39	0.6535	0.0125	0.4895
		Biological process	GO:0009605	response to external stimulus	44	17.5	0.0172	236	2306	17375	1.40	1.0000	0.2714	27.5678
Cluster 16	3.36	Molecular function	GO:0004553	hydrolase activity, hydrolyzing O-glycosyl compounds	9	3.6	0.0001	241	100	16438	6.14	0.0882	0.0042	0.1667
		Molecular function	GO:0016798	hydrolase activity, acting on glycosyl bonds	9	3.6	0.0005	241	126	16438	4.87	0.3627	0.0149	0.8110
		Biological process	GO:1901136	carbohydrate derivative catabolic process	8	3.2	0.0015	236	124	17375	4.75	0.9975	0.0444	2.7399
Cluster 17	3.34	Biological process	GO:0019318	hexose metabolic process	15	6.0	0.0000	236	212	17375	5.21	0.0048	0.0001	0.0022
		Biological process	GO:0005996	monosaccharide metabolic process	15	6.0	0.0000	236	222	17375	4.97	0.0082	0.0002	0.0038
		Biological process	GO:0006006	glucose metabolic process	10	4.0	0.0009	236	183	17375	4.02	0.9711	0.0293	1.6270
		Biological process	GO:0006094	gluconeogenesis	6	2.4	0.0024	236	69	17375	6.40	0.9999	0.0665	4.3913
		Biological process	GO:0019319	hexose biosynthetic process	6	2.4	0.0027	236	71	17375	6.22	1.0000	0.0726	4.9638
		Biological process	GO:0046364	monosaccharide biosynthetic process	6	2.4	0.0031	236	73	17375	6.05	1.0000	0.0784	5.5860
		Biological process	GO:0016051	carbohydrate biosynthetic process	6	2.4	0.0994	236	181	17375	2.44	1.0000	0.6542	85.7237
Cluster 18	3.17	Biological process	GO:0019637	organophosphate metabolic process	33	13.1	0.0000	236	961	17375	2.53	0.0090	0.0002	0.0042
		Biological process	GO:0009117	nucleotide metabolic process	25	9.9	0.0000	236	613	17375	3.00	0.0128	0.0003	0.0059
		Biological process	GO:0006753	nucleoside phosphate metabolic process	25	9.9	0.0000	236	620	17375	2.97	0.0154	0.0004	0.0072
		Biological process	GO:0006796	phosphate-containing compound metabolic process	62	24.6	0.0003	236	2931	17375	1.56	0.6477	0.0125	0.4818
		Biological process	GO:0006793	phosphorus metabolic process	62	24.6	0.0003	236	2933	17375	1.56	0.6546	0.0124	0.4909
		Biological process	GO:0016310	phosphorylation	35	13.9	0.1672	236	2122	17375	1.21	1.0000	0.7761	96.6682
		Biological process	GO:0019220	regulation of phosphate metabolic process	25	9.9	0.3768	236	1643	17375	1.12	1.0000	0.9400	99.9848
		Biological process	GO:0051174	regulation of phosphorus metabolic process	25	9.9	0.3780	236	1644	17375	1.12	1.0000	0.9399	99.9853
Cluster 19	3.13	Biological process	GO:0060249	anatomical structure homeostasis	15	6.0	0.0003	236	343	17375	3.22	0.6354	0.0122	0.4660
		Biological process	GO:0001894	tissue homeostasis	11	4.4	0.0008	236	220	17375	3.68	0.9669	0.0289	1.5650
		Biological process	GO:0048871	multicellular organismal homeostasis	13	5.2	0.0019	236	332	17375	2.88	0.9996	0.0562	3.5532
Cluster 20	3.04	Biological process	GO:0010033	response to organic substance	74	29.4	0.0000	236	3067	17375	1.78	0.0013	0.0000	0.0006
		Biological process	GO:0009725	response to hormone	34	13.5	0.0000	236	1069	17375	2.34	0.0304	0.0007	0.0143
		Biological process	GO:1901700	response to oxygen-containing compound	48	19.0	0.0000	236	1872	17375	1.89	0.0780	0.0014	0.0376
		Biological process	GO:0009719	response to endogenous stimulus	47	18.7	0.0000	236	1849	17375	1.87	0.1197	0.0021	0.0590
		Biological process	GO:0014070	response to organic cyclic compound	36	14.3	0.0001	236	1302	17375	2.04	0.2527	0.0043	0.1348
		Biological process	GO:1901698	response to nitrogen compound	33	13.1	0.0001	236	1171	17375	2.07	0.3649	0.0063	0.2099
		Biological process	GO:0070887	cellular response to chemical stimulus	58	23.0	0.0001	236	2604	17375	1.64	0.3741	0.0064	0.2167
		Biological process	GO:0032870	cellular response to hormone stimulus	22	8.7	0.0003	236	671	17375	2.41	0.7242	0.0144	0.5945
		Biological process	GO:0033993	response to lipid	32	12.7	0.0004	236	1199	17375	1.96	0.7861	0.0161	0.7114
		Biological process	GO:0071407	cellular response to organic cyclic compound	21	8.3	0.0004	236	631	17375	2.45	0.7868	0.0160	0.7128
		Biological process	GO:0043434	response to peptide hormone	18	7.1	0.0005	236	501	17375	2.65	0.8598	0.0189	0.9055
		Biological process	GO:0071310	cellular response to organic substance	48	19.0	0.0005	236	2143	17375	1.65	0.8740	0.0197	0.9543
		Biological process	GO:1901652	response to peptide	19	7.5	0.0006	236	555	17375	2.52	0.8992	0.0214	1.0565
		Biological process	GO:0010243	response to organonitrogen compound	28	11.1	0.0013	236	1067	17375	1.93	0.9941	0.0393	2.3506
		Biological process	GO:0071495	cellular response to endogenous stimulus	31	12.3	0.0022	236	1277	17375	1.79	0.9998	0.0618	3.9663
		Biological process	GO:1901699	cellular response to nitrogen compound	19	7.5	0.0034	236	652	17375	2.15	1.0000	0.0831	6.1086
		Biological process	GO:0071375	cellular response to peptide hormone stimulus	12	4.8	0.0038	236	314	17375	2.81	1.0000	0.0902	6.7621
		Biological process	GO:0071396	cellular response to lipid	18	7.1	0.0043	236	614	17375	2.16	1.0000	0.0982	7.6330
		Biological process	GO:1901701	cellular response to oxygen-containing compound	26	10.3	0.0067	236	1088	17375	1.76	1.0000	0.1392	11.7409
		Biological process	GO:1901653	cellular response to peptide	12	4.8	0.0069	236	341	17375	2.59	1.0000	0.1423	12.0705
		Biological process	GO:0071417	cellular response to organonitrogen compound	15	6.0	0.0234	236	569	17375	1.94	1.0000	0.3294	35.6123
		Biological process	GO:0032868	response to insulin	9	3.6	0.0478	236	295	17375	2.25	1.0000	0.4664	59.7568
		Biological process	GO:0032869	cellular response to insulin stimulus	7	2.8	0.0722	236	215	17375	2.40	1.0000	0.5780	75.1903
		Biological process	GO:0071383	cellular response to steroid hormone stimulus	8	3.2	0.0740	236	270	17375	2.18	1.0000	0.5791	76.0763
		Biological process	GO:0048545	response to steroid hormone	11	4.4	0.1724	236	523	17375	1.55	1.0000	0.7839	97.0322
Cluster 21	2.80	Biological process	GO:0007040	lysosome organization	7	2.8	0.0001	236	54	17375	9.54	0.2926	0.0049	0.1602
		Biological process	GO:0080171	lytic vacuole organization	7	2.8	0.0001	236	54	17375	9.54	0.2926	0.0049	0.1602
		Biological process	GO:0007033	vacuole organization	10	4.0	0.0013	236	193	17375	3.81	0.9942	0.0391	2.3540
		Biological process	GO:0050885	neuromuscular process controlling balance	5	2.0	0.0122	236	66	17375	5.58	1.0000	0.2150	20.4354
		Biological process	GO:0050905	neuromuscular process	5	2.0	0.0819	236	121	17375	3.04	1.0000	0.6086	79.5898
Cluster 22	2.70	Biological process	GO:1901361	organic cyclic compound catabolic process	14	5.6	0.0004	236	314	17375	3.28	0.7672	0.0155	0.6724
		Biological process	GO:0019439	aromatic compound catabolic process	13	5.2	0.0006	236	290	17375	3.30	0.9150	0.0226	1.1346
		Biological process	GO:0044270	cellular nitrogen compound catabolic process	11	4.4	0.0051	236	281	17375	2.88	1.0000	0.1123	9.0025
		Biological process	GO:0046700	heterocycle catabolic process	10	4.0	0.0142	236	280	17375	2.63	1.0000	0.2397	23.3870
Cluster 23	2.66	Biological process	GO:0006575	cellular modified amino acid metabolic process	12	4.8	0.0000	236	145	17375	6.09	0.0180	0.0004	0.0084
		Biological process	GO:0042398	cellular modified amino acid biosynthetic process	6	2.4	0.0002	236	39	17375	11.33	0.4980	0.0088	0.3186
		Biological process	GO:0044272	sulfur compound biosynthetic process	6	2.4	0.0098	236	96	17375	4.60	1.0000	0.1835	16.7196
		Biological process	GO:0006749	glutathione metabolic process	5	2.0	0.0099	236	62	17375	5.94	1.0000	0.1838	16.8271
		Biological process	GO:0006750	glutathione biosynthetic process	3	1.2	0.0110	236	12	17375	18.41	1.0000	0.1991	18.5795
		Biological process	GO:0019184	nonribosomal peptide biosynthetic process	3	1.2	0.0149	236	14	17375	15.78	1.0000	0.2464	24.3431
		Biological process	GO:0006790	sulfur compound metabolic process	10	4.0	0.0205	236	298	17375	2.47	1.0000	0.3030	32.0192
Cluster 24	2.61	Biological process	GO:0019674	NAD metabolic process	7	2.8	0.0000	236	40	17375	12.88	0.0585	0.0011	0.0279
		Molecular function	GO:0004514	nicotinate-nucleotide diphosphorylase (carboxylating) activity	3	1.2	0.0006	241	3	16438	68.21	0.4177	0.0168	0.9725
		Biological process	GO:0009108	coenzyme biosynthetic process	8	3.2	0.0007	236	110	17375	5.35	0.9488	0.0260	1.3668
		Biological process	GO:0009435	NAD biosynthetic process	4	1.6	0.0008	236	14	17375	21.04	0.9592	0.0277	1.4702
		Biological process	GO:0019359	nicotinamide nucleotide biosynthetic process	4	1.6	0.0017	236	18	17375	16.36	0.9990	0.0502	3.1414
		Biological process	GO:0019363	pyridine nucleotide biosynthetic process	4	1.6	0.0017	236	18	17375	16.36	0.9990	0.0502	3.1414
		Biological process	GO:0072525	pyridine-containing compound biosynthetic process	4	1.6	0.0020	236	19	17375	15.50	0.9997	0.0579	3.6836
		Biological process	GO:0051188	cofactor biosynthetic process	8	3.2	0.0043	236	150	17375	3.93	1.0000	0.0995	7.7815
		Biological process	GO:0009165	nucleotide biosynthetic process	10	4.0	0.0279	236	315	17375	2.34	1.0000	0.3534	40.9480
		Biological process	GO:1901293	nucleoside phosphate biosynthetic process	10	4.0	0.0288	236	317	17375	2.32	1.0000	0.3600	41.9196
		Molecular function	GO:0016763	transferase activity, transferring pentosyl groups	3	1.2	0.1863	241	54	16438	3.79	1.0000	0.7596	95.8998
Cluster 25	2.60	Cellular component	GO:0098589	membrane region	21	8.3	0.0000	242	404	13535	2.91	0.0177	0.0015	0.0530
		Cellular component	GO:0045121	membrane raft	13	5.2	0.0209	242	345	13535	2.11	1.0000	0.2194	26.0999
		Cellular component	GO:0098857	membrane microdomain	13	5.2	0.0209	242	345	13535	2.11	1.0000	0.2194	26.0999
Cluster 26	2.54	Biological process	GO:0072001	renal system development	13	5.2	0.0023	236	339	17375	2.82	0.9999	0.0641	4.2050
		Biological process	GO:0001655	urogenital system development	14	5.6	0.0025	236	388	17375	2.66	1.0000	0.0675	4.5186
		Biological process	GO:0001822	kidney development	12	4.8	0.0042	236	319	17375	2.77	1.0000	0.0981	7.5806
Cluster 27	2.51	Cellular component	GO:0005925	focal adhesion	19	7.5	0.0003	242	396	13535	2.68	0.1187	0.0090	0.3754
		Cellular component	GO:0005924	cell-substrate adherens junction	19	7.5	0.0003	242	401	13535	2.65	0.1368	0.0092	0.4371
		Cellular component	GO:0030055	cell-substrate junction	19	7.5	0.0004	242	406	13535	2.62	0.1575	0.0100	0.5089
		Cellular component	GO:0005912	adherens junction	24	9.5	0.0012	242	643	13535	2.09	0.4275	0.0262	1.6470
		Cellular component	GO:0070161	anchoring junction	24	9.5	0.0016	242	658	13535	2.04	0.5294	0.0309	2.2193
		Cellular component	GO:0030054	cell junction	30	11.9	0.1150	242	1293	13535	1.30	1.0000	0.5733	82.6183
		Biological process	GO:0007010	cytoskeleton organization	17	6.7	0.4663	236	1133	17375	1.10	1.0000	0.9650	99.9992
Cluster 28	2.51	Biological process	GO:0015031	protein transport	39	15.5	0.0004	236	1602	17375	1.79	0.8347	0.0177	0.8298
		Biological process	GO:0045184	establishment of protein localization	41	16.3	0.0008	236	1770	17375	1.71	0.9620	0.0280	1.5026

		Biological process	GO:0006886	intracellular protein transport	25	9.9	0.0012	236	901	17375	2.04	0.9923	0.0378	2.2249
		Biological process	GO:0046907	intracellular transport	32	12.7	0.0015	236	1303	17375	1.81	0.9976	0.0444	2.7576
		Biological process	GO:0033036	macromolecule localization	52	20.6	0.0028	236	2573	17375	1.49	1.0000	0.0726	5.0312
		Biological process	GO:0051649	establishment of localization in cell	38	15.1	0.0032	236	1724	17375	1.62	1.0000	0.0790	5.7685
		Biological process	GO:0008104	protein localization	46	18.3	0.0045	236	2255	17375	1.50	1.0000	0.1026	8.0756
		Biological process	GO:0034613	cellular protein localization	33	13.1	0.0070	236	1505	17375	1.61	1.0000	0.1428	12.2366
		Biological process	GO:0051641	cellular localization	46	18.3	0.0077	236	2323	17375	1.46	1.0000	0.1550	13.4316
		Biological process	GO:0070727	cellular macromolecule localization	33	13.1	0.0078	236	1517	17375	1.60	1.0000	0.1561	13.5974
		Biological process	GO:1902580	single-organism cellular localization	22	8.7	0.0240	236	975	17375	1.66	1.0000	0.3312	36.2814
Cluster 29	2.34	Biological process	GO:0019395	fatty acid oxidation	8	3.2	0.0003	236	97	17375	6.07	0.7512	0.0150	0.6420
		Biological process	GO:0034440	lipid oxidation	8	3.2	0.0004	236	99	17375	5.95	0.7932	0.0161	0.7269
		Biological process	GO:0044242	cellular lipid catabolic process	10	4.0	0.0009	236	183	17375	4.02	0.9711	0.0293	1.6270
		Biological process	GO:0030258	lipid modification	9	3.6	0.0053	236	196	17375	3.38	1.0000	0.1164	9.3823
		Biological process	GO:0072329	monocarboxylic acid catabolic process	6	2.4	0.0098	236	96	17375	4.60	1.0000	0.1835	16.7196
		Biological process	GO:0006635	fatty acid beta-oxidation	4	1.6	0.0668	236	69	17375	4.27	1.0000	0.5561	72.3578
		Biological process	GO:0009062	fatty acid catabolic process	4	1.6	0.1081	236	85	17375	3.46	1.0000	0.6704	88.0832
Cluster 30	2.22	Biological process	GO:0009410	response to xenobiotic stimulus	8	3.2	0.0001	236	73	17375	8.07	0.2071	0.0035	0.1074
		Biological process	GO:0006805	xenobiotic metabolic process	7	2.8	0.0001	236	59	17375	8.73	0.4350	0.0076	0.2640
		Biological process	GO:0071466	cellular response to xenobiotic stimulus	7	2.8	0.0002	236	63	17375	8.18	0.5610	0.0102	0.3804
		Biological process	GO:0042178	xenobiotic catabolic process	4	1.6	0.0012	236	16	17375	18.41	0.9920	0.0379	2.2092
		Molecular function	GO:1900750	oligopeptide binding	4	1.6	0.0021	241	18	16438	15.16	0.8395	0.0470	3.2526
		Molecular function	GO:0004364	glutathione transferase activity	5	2.0	0.0035	241	43	16438	7.93	0.9504	0.0707	5.2852
		Molecular function	GO:0016765	transferase activity, transferring alkyl or aryl (other than methyl) groups	5	2.0	0.0220	241	73	16438	4.67	1.0000	0.2578	29.1644
		Molecular function	GO:0043295	glutathione binding	3	1.2	0.0250	241	17	16438	12.04	1.0000	0.2766	32.4312
		Molecular function	GO:0072341	modified amino acid binding	5	2.0	0.0296	241	80	16438	4.26	1.0000	0.3151	37.1996
		Cellular component	GO:0005640	nuclear outer membrane	3	1.2	0.0725	242	25	13535	6.71	1.0000	0.4268	65.9462
		Molecular function	GO:1901681	sulfur compound binding	6	2.4	0.2471	241	229	16438	1.79	1.0000	0.8218	98.7672
		Cellular component	GO:0031965	nuclear membrane	6	2.4	0.4669	242	252	13535	1.33	1.0000	0.9264	99.9878
Cluster 31	2.18	Biological process	GO:0009072	aromatic amino acid family metabolic process	5	2.0	0.0002	236	22	17375	16.73	0.5467	0.0100	0.3656
		Biological process	GO:0009074	aromatic amino acid family catabolic process	4	1.6	0.0006	236	13	17375	22.65	0.9210	0.0230	1.1685
		Biological process	GO:0042402	cellular biogenic amine catabolic process	4	1.6	0.0017	236	18	17375	16.36	0.9990	0.0502	3.1414
		Biological process	GO:0009310	amine catabolic process	4	1.6	0.0020	236	19	17375	15.50	0.9997	0.0579	3.6836
		Biological process	GO:0044270	cellular nitrogen compound catabolic process	11	4.4	0.0051	236	281	17375	2.88	1.0000	0.1123	9.0025
		Biological process	GO:0042436	indole-containing compound catabolic process	3	1.2	0.0062	236	9	17375	24.54	1.0000	0.1294	10.8517
		Biological process	GO:0046218	indolalkylamine catabolic process	3	1.2	0.0062	236	9	17375	24.54	1.0000	0.1294	10.8517
		Biological process	GO:0006569	tryptophan catabolic process	3	1.2	0.0062	236	9	17375	24.54	1.0000	0.1294	10.8517
		Biological process	GO:0006568	tryptophan metabolic process	3	1.2	0.0110	236	12	17375	18.41	1.0000	0.1991	18.5795
		Biological process	GO:0006586	indolalkylamine metabolic process	3	1.2	0.0110	236	12	17375	18.41	1.0000	0.1991	18.5795
		Biological process	GO:0006576	cellular biogenic amine metabolic process	4	1.6	0.0156	236	39	17375	7.55	1.0000	0.2546	25.3525
		Biological process	GO:0042430	indole-containing compound metabolic process	3	1.2	0.0512	236	27	17375	8.18	1.0000	0.4821	62.3847
		Biological process	GO:0044106	cellular amine metabolic process	4	1.6	0.0645	236	68	17375	4.33	1.0000	0.5463	71.0704
		Biological process	GO:0009308	amine metabolic process	4	1.6	0.0890	236	78	17375	3.78	1.0000	0.6257	82.3286
Cluster 32	2.12	Biological process	GO:0001101	response to acid chemical	16	6.3	0.0007	236	427	17375	2.76	0.9482	0.0261	1.3615
		Biological process	GO:0071229	cellular response to acid chemical	9	3.6	0.0088	236	214	17375	3.10	1.0000	0.1713	15.1629
		Biological process	GO:0043200	response to amino acid	6	2.4	0.0668	236	160	17375	2.76	1.0000	0.5568	72.3225
Cluster 33	2.12	Molecular function	GO:0004033	aldo-keto reductase (NADP) activity	4	1.6	0.0021	241	18	16438	15.16	0.8395	0.0470	3.2526
		Molecular function	GO:0004032	alditol:NADP+ 1-oxidoreductase activity	3	1.2	0.0107	241	11	16438	18.60	0.9999	0.1597	15.3540
		Molecular function	GO:0008106	alcohol dehydrogenase (NADP+) activity	3	1.2	0.0197	241	15	16438	13.64	1.0000	0.2435	26.4836
Cluster 34	2.11	Biological process	GO:0060627	regulation of vesicle-mediated transport	17	6.7	0.0006	236	463	17375	2.70	0.9045	0.0217	1.0814
		Biological process	GO:0006897	endocytosis	18	7.1	0.0009	236	528	17375	2.51	0.9702	0.0293	1.6127
		Biological process	GO:0045807	positive regulation of endocytosis	7	2.8	0.0095	236	133	17375	3.87	1.0000	0.1799	16.2360
		Biological process	GO:0030100	regulation of endocytosis	9	3.6	0.0098	236	218	17375	3.04	1.0000	0.1840	16.6884
		Biological process	GO:0006898	receptor-mediated endocytosis	8	3.2	0.0468	236	243	17375	2.42	1.0000	0.4639	59.0131
		Biological process	GO:0048259	regulation of receptor-mediated endocytosis	4	1.6	0.1025	236	83	17375	3.55	1.0000	0.6596	86.6094
Cluster 35	2.10	Cellular component	GO:0031968	organelle outer membrane	10	4.0	0.0030	242	167	13535	3.35	0.7695	0.0570	4.2756
		Cellular component	GO:0019867	outer membrane	10	4.0	0.0038	242	173	13535	3.23	0.8432	0.0619	5.3669
		Cellular component	GO:0005741	mitochondrial outer membrane	7	2.8	0.0415	242	142	13535	2.76	1.0000	0.3196	45.5397
Cluster 36	2.09	Molecular function	GO:0098641	cadherin binding involved in cell-cell adhesion	12	4.8	0.0004	241	220	16438	3.72	0.2917	0.0132	0.6213
		Molecular function	GO:0098632	protein binding involved in cell-cell adhesion	12	4.8	0.0005	241	227	16438	3.61	0.3618	0.0154	0.8084
		Molecular function	GO:0098631	protein binding involved in cell adhesion	12	4.8	0.0006	241	232	16438	3.53	0.4164	0.0172	0.9687
		Molecular function	GO:0045296	cadherin binding	12	4.8	0.0009	241	243	16438	3.37	0.5430	0.0228	1.4051
		Cellular component	GO:0005912	adherens junction	24	9.5	0.0012	242	643	13535	2.09	0.4275	0.0262	1.6470
		Cellular component	GO:0070161	anchoring junction	24	9.5	0.0016	242	658	13535	2.04	0.5294	0.0309	2.2193
		Molecular function	GO:0050839	cell adhesion molecule binding	14	5.6	0.0052	241	393	16438	2.43	0.9884	0.1007	7.7400
		Cellular component	GO:0005913	cell-cell adherens junction	11	4.4	0.0225	242	269	13535	2.29	1.0000	0.2205	27.8483
		Biological process	GO:0098609	cell-cell adhesion	21	8.3	0.0830	236	1060	17375	1.46	1.0000	0.6104	80.0152
		Cellular component	GO:0030054	cell junction	30	11.9	0.1150	242	1293	13535	1.30	1.0000	0.5733	82.6183
		Cellular component	GO:0005911	cell-cell junction	16	6.3	0.1278	242	609	13535	1.47	1.0000	0.5989	85.8893
		Biological process	GO:0007155	cell adhesion	26	10.3	0.1373	236	1468	17375	1.30	1.0000	0.7268	93.5768
		Biological process	GO:0022610	biological adhesion	26	10.3	0.1447	236	1478	17375	1.30	1.0000	0.7384	94.5264
Cluster 37	2.05	Biological process	GO:0001889	liver development	14	5.6	0.0000	236	222	17375	4.64	0.0421	0.0009	0.0199
		Biological process	GO:0061008	hepaticobiliary system development	14	5.6	0.0000	236	225	17375	4.58	0.0485	0.0010	0.0230
		Biological process	GO:0048732	gland development	16	6.3	0.0134	236	584	17375	2.02	1.0000	0.2313	22.1898
		Biological process	GO:0071384	cellular response to corticosteroid stimulus	6	2.4	0.0157	236	108	17375	4.09	1.0000	0.2548	25.4800
		Biological process	GO:0071385	cellular response to glucocorticoid stimulus	5	2.0	0.0483	236	101	17375	3.64	1.0000	0.4677	60.1243
		Biological process	GO:0071383	cellular response to steroid hormone stimulus	8	3.2	0.0740	236	270	17375	2.18	1.0000	0.5791	76.0763
		Biological process	GO:0031960	response to corticosteroid	8	3.2	0.1178	236	304	17375	1.94	1.0000	0.6898	90.2695
		Biological process	GO:0048545	response to steroid hormone	11	4.4	0.1724	236	523	17375	1.55	1.0000	0.7839	97.0322
		Biological process	GO:0051384	response to glucocorticoid	7	2.8	0.1800	236	280	17375	1.84	1.0000	0.7942	97.5000
Cluster 38	2.01	Biological process	GO:0071372	cellular response to follicle-stimulating hormone stimulus	4	1.6	0.0056	236	27	17375	10.91	1.0000	0.1207	9.9516
		Biological process	GO:0032354	response to follicle-stimulating hormone	4	1.6	0.0099	236	33	17375	8.92	1.0000	0.1833	16.8608
		Biological process	GO:0034698	response to gonadotropin	5	2.0	0.0116	236	65	17375	5.66	1.0000	0.2070	19.5010
		Biological process	GO:0071371	cellular response to gonadotropin stimulus	4	1.6	0.0145	236	38	17375	7.75	1.0000	0.2424	23.8468
Cluster 39	2.00	Molecular function	GO:0043167	ion binding	78	31.0	0.0005	241	3722	16438	1.43	0.3206	0.0142	0.6962
		Molecular function	GO:0043169	cation binding	67	26.6	0.0168	241	3545	16438	1.29	1.0000	0.2212	23.0545
		Molecular function	GO:0046872	metal ion binding	66	26.2	0.0187	241	3501	16438	1.29	1.0000	0.2369	25.4076
		Molecular function	GO:0046914	transition metal ion binding	28	11.1	0.0691	241	1373	16438	1.39	1.0000	0.4905	67.0165

Table S15: B-II Male_Effect of FGR (only Good)

Rank	Score	Category	Term ID	Term name	Count	%	PValue	List Total	Pop Hits	Pop Total	Fold Enrichment	Bonferroni	Benjamini	FDR
Cluster 1	7.83	Cellular component	GO:0043230	extracellular organelle	64	41.3	0.0000	145	2666	13535	2.24	0.0000	0.0000	0.0000
		Cellular component	GO:1903561	extracellular vesicle	63	40.6	0.0000	145	2661	13535	2.21	0.0000	0.0000	0.0000
		Cellular component	GO:0070062	extracellular exosome	62	40.0	0.0000	145	2646	13535	2.19	0.0000	0.0000	0.0000
		Cellular component	GO:0031988	membrane-bounded vesicle	66	42.6	0.0000	145	3321	13535	1.86	0.0000	0.0000	0.0001
		Cellular component	GO:0044421	extracellular region part	66	42.6	0.0000	145	3710	13535	1.66	0.0016	0.0002	0.0050
		Cellular component	GO:0005576	extracellular region	67	43.2	0.0001	145	4113	13535	1.52	0.0278	0.0031	0.0895
Cluster 2	3.41	Cellular component	GO:0005912	adherens junction	19	12.3	0.0002	145	643	13535	2.76	0.0694	0.0072	0.2281
		Cellular component	GO:0070161	anchoring junction	19	12.3	0.0002	145	658	13535	2.70	0.0911	0.0086	0.3031
		Cellular component	GO:0005925	focal adhesion	14	9.0	0.0003	145	396	13535	3.30	0.1294	0.0115	0.4391
		Cellular component	GO:0005924	cell-substrate adherens junction	14	9.0	0.0004	145	401	13535	3.26	0.1446	0.0119	0.4951
		Cellular component	GO:0030055	cell-substrate junction	14	9.0	0.0004	145	406	13535	3.22	0.1613	0.0125	0.5571
		Cellular component	GO:0030054	cell junction	26	16.8	0.0024	145	1293	13535	1.88	0.6499	0.0566	3.2791
Cluster 3	3.06	Molecular function	GO:0044822	poly(A) RNA binding	30	19.4	0.0000	147	1219	16438	2.75	0.0005	0.0002	0.0013
		Biological process	GO:1901566	organonitrogen compound biosynthetic process	31	20.0	0.0000	146	1412	17375	2.61	0.0045	0.0045	0.0028
		Cellular component	GO:0022627	cytosolic small ribosomal subunit	10	6.5	0.0000	145	107	13535	8.72	0.0009	0.0001	0.0029
		Molecular function	GO:0005198	structural molecule activity	23	14.8	0.0000	147	848	16438	3.03	0.0032	0.0008	0.0084
		Molecular function	GO:0003723	RNA binding	34	21.9	0.0000	147	1673	16438	2.27	0.0046	0.0009	0.0122
		Biological process	GO:0006412	translation	21	13.5	0.0000	146	804	17375	3.11	0.0337	0.0170	0.0213
		Cellular component	GO:0015935	small ribosomal subunit	10	6.5	0.0000	145	135	13535	6.91	0.0061	0.0008	0.0193
		Biological process	GO:0043043	peptide biosynthetic process	21	13.5	0.0000	146	824	17375	3.03	0.0478	0.0162	0.0304
		Biological process	GO:0006518	peptide metabolic process	22	14.2	0.0000	146	956	17375	2.74	0.1211	0.0318	0.0803
		Biological process	GO:0043603	cellular amide metabolic process	24	15.5	0.0000	146	1111	17375	2.57	0.1267	0.0267	0.0842
		Biological process	GO:0043604	amide biosynthetic process	21	13.5	0.0001	146	893	17375	2.80	0.1416	0.0251	0.0950
		Biological process	GO:0034248	regulation of cellular amide metabolic process	11	7.1	0.0009	146	359	17375	3.65	0.9197	0.0892	1.5568
		Cellular component	GO:0022626	cytosolic ribosome	11	7.1	0.0010	145	287	13535	3.58	0.3576	0.0273	1.3963
		Cellular component	GO:0044445	cytosolic part	13	8.4	0.0013	145	404	13535	3.00	0.4321	0.0327	1.7817
		Biological process	GO:0006417	regulation of translation	10	6.5	0.0019	146	333	17375	3.57	0.9963	0.1480	3.4258
		Cellular component	GO:0044391	ribosomal subunit	11	7.1	0.0042	145	350	13535	2.93	0.8485	0.0822	5.8192
		Molecular function	GO:0003735	structural constituent of ribosome	11	7.1	0.0044	147	421	16438	2.92	0.9132	0.0865	6.2318
		Biological process	GO:0071826	ribonucleoprotein complex subunit organization	8	5.2	0.0054	146	253	17375	3.76	1.0000	0.2504	9.2270
		Biological process	GO:0010608	posttranscriptional regulation of gene expression	10	6.5	0.0060	146	395	17375	3.01	1.0000	0.2571	10.1658
		Biological process	GO:0000028	ribosomal small subunit assembly	4	2.6	0.0066	146	46	17375	10.35	1.0000	0.2595	11.2703
		Biological process	GO:0042274	ribosomal small subunit biogenesis	5	3.2	0.0112	146	104	17375	5.72	1.0000	0.3486	18.3441
		Biological process	GO:0045727	positive regulation of translation	5	3.2	0.0144	146	112	17375	5.31	1.0000	0.4001	22.9411
		Cellular component	GO:0030529	intracellular ribonucleoprotein complex	19	12.3	0.0158	145	978	13535	1.81	0.9992	0.2391	20.2057
		Cellular component	GO:1990904	ribonucleoprotein complex	19	12.3	0.0160	145	979	13535	1.81	0.9992	0.2333	20.3781
		Biological process	GO:0022618	ribonucleoprotein complex assembly	7	4.5	0.0161	146	242	17375	3.44	1.0000	0.4188	25.1941
		Biological process	GO:0034250	positive regulation of cellular amide metabolic process	5	3.2	0.0207	146	125	17375	4.76	1.0000	0.4670	31.3212
		Cellular component	GO:0005840	ribosome	11	7.1	0.0238	145	456	13535	2.25	1.0000	0.2466	28.9589
		Biological process	GO:0022613	ribonucleoprotein complex biogenesis	9	5.8	0.0392	146	460	17375	2.33	1.0000	0.5882	51.1957
		Biological process	GO:0042255	ribosome assembly	4	2.6	0.0417	146	92	17375	5.17	1.0000	0.5949	53.4408
		Biological process	GO:0042254	ribosome biogenesis	6	3.9	0.1240	146	315	17375	2.27	1.0000	0.8291	90.6880
		Biological process	GO:0034645	cellular macromolecule biosynthetic process	39	25.2	0.4688	146	4454	17375	1.04	1.0000	0.9844	99.9988
Cluster 4	2.89	Molecular function	GO:0003723	RNA binding	34	21.9	0.0000	147	1673	16438	2.27	0.0046	0.0009	0.0122
		Molecular function	GO:1901265	nucleoside phosphate binding	39	25.2	0.0001	147	2307	16438	1.89	0.0481	0.0070	0.1298
		Molecular function	GO:0000166	nucleotide binding	39	25.2	0.0001	147	2307	16438	1.89	0.0481	0.0070	0.1298
		Molecular function	GO:0036094	small molecule binding	41	26.5	0.0001	147	2506	16438	1.83	0.0590	0.0076	0.1600
		Molecular function	GO:0016462	pyrophosphatase activity	18	11.6	0.0004	147	749	16438	2.69	0.1790	0.0217	0.5179
		Molecular function	GO:0016818	hydrolase activity, acting on acid anhydrides, in phosphorus-containing anhydrides	18	11.6	0.0004	147	752	16438	2.68	0.1866	0.0204	0.5422
		Molecular function	GO:0016817	hydrolase activity, acting on acid anhydrides	18	11.6	0.0004	147	758	16438	2.66	0.2024	0.0204	0.5938
		Molecular function	GO:0017111	nucleoside-triphosphatase activity	17	11.0	0.0005	147	703	16438	2.70	0.2500	0.0219	0.7544
		Molecular function	GO:0035639	purine ribonucleoside triphosphate binding	30	19.4	0.0007	147	1753	16438	1.91	0.3192	0.0271	1.0072
		Molecular function	GO:0032550	purine ribonucleoside binding	30	19.4	0.0008	147	1763	16438	1.90	0.3438	0.0277	1.1030
		Molecular function	GO:0032549	ribonucleoside binding	30	19.4	0.0008	147	1766	16438	1.90	0.3514	0.0267	1.1332
		Molecular function	GO:0001883	purine nucleoside binding	30	19.4	0.0008	147	1766	16438	1.90	0.3514	0.0267	1.1332
		Molecular function	GO:0001882	nucleoside binding	30	19.4	0.0008	147	1773	16438	1.89	0.3694	0.0268	1.2066
		Molecular function	GO:0032555	purine ribonucleotide binding	30	19.4	0.0010	147	1798	16438	1.87	0.4376	0.0298	1.5038
		Molecular function	GO:0017076	purine nucleotide binding	30	19.4	0.0011	147	1809	16438	1.85	0.4692	0.0312	1.6536
		Molecular function	GO:0032553	ribonucleotide binding	30	19.4	0.0012	147	1815	16438	1.85	0.4867	0.0313	1.7406
		Molecular function	GO:0097367	carbohydrate derivative binding	33	21.3	0.0021	147	2152	16438	1.71	0.6909	0.0498	3.0437
		Molecular function	GO:1901363	heterocyclic compound binding	68	43.9	0.0024	147	5645	16438	1.35	0.7432	0.0551	3.5158
		Molecular function	GO:0097159	organic cyclic compound binding	68	43.9	0.0036	147	5726	16438	1.33	0.8665	0.0745	5.1630
		Molecular function	GO:0005524	ATP binding	22	14.2	0.0124	147	1403	16438	1.75	0.9990	0.1794	16.6565
		Molecular function	GO:0032559	adenyl ribonucleotide binding	22	14.2	0.0162	147	1440	16438	1.71	0.9999	0.2124	21.2494
		Molecular function	GO:0030554	adenyl nucleotide binding	22	14.2	0.0173	147	1449	16438	1.70	0.9999	0.2148	22.4788
		Molecular function	GO:0042623	ATPase activity, coupled	8	5.2	0.0225	147	315	16438	2.84	1.0000	0.2449	28.3044
		Molecular function	GO:0016887	ATPase activity	9	5.8	0.0396	147	433	16438	2.32	1.0000	0.3554	44.5479
		Molecular function	GO:0003676	nucleic acid binding	41	26.5	0.1162	147	3764	16438	1.22	1.0000	0.6691	83.5561
Cluster 5	2.52	Biological process	GO:0034622	cellular macromolecular complex assembly	20	12.9	0.0002	146	906	17375	2.63	0.4289	0.0545	0.3479
		Biological process	GO:0022607	cellular component assembly	37	23.9	0.0004	146	2456	17375	1.79	0.7027	0.0777	0.7516
		Biological process	GO:0065003	macromolecular complex assembly	27	17.4	0.0005	146	1563	17375	2.06	0.7646	0.0772	0.8956
		Biological process	GO:0043933	macromolecular complex subunit organization	34	21.9	0.0006	146	2213	17375	1.83	0.8101	0.0837	1.0279
		Biological process	GO:0044085	cellular component biogenesis	39	25.2	0.0006	146	2705	17375	1.72	0.8445	0.0889	1.1510
		Biological process	GO:0043254	regulation of protein complex assembly	11	7.1	0.0010	146	365	17375	3.59	0.9427	0.0971	1.7634
		Biological process	GO:0044087	regulation of cellular component biogenesis	16	10.3	0.0034	146	816	17375	2.33	0.9999	0.1926	5.9392
		Biological process	GO:0051493	regulation of cytoskeleton organization	11	7.1	0.0036	146	436	17375	3.00	1.0000	0.1894	6.3208
		Biological process	GO:0071822	protein complex subunit organization	22	14.2	0.0065	146	1407	17375	1.86	1.0000	0.2586	11.0644
		Biological process	GO:0044089	positive regulation of cellular component biogenesis	10	6.5	0.0128	146	447	17375	2.66	1.0000	0.3745	20.5930
		Biological process	GO:0051258	protein polymerization	7	4.5	0.0146	146	237	17375	3.51	1.0000	0.4006	23.2227
		Biological process	GO:0006461	protein complex assembly	19	12.3	0.0186	146	1268	17375	1.78	1.0000	0.4518	28.5765
		Biological process	GO:0070271	protein complex biogenesis	19	12.3	0.0187	146	1269	17375	1.78	1.0000	0.4506	28.7506
		Biological process	GO:0043623	cellular protein complex assembly	9	5.8	0.0607	146	504	17375	2.13	1.0000	0.6720	67.4780
Cluster 6	2.39	Biological process	GO:0061024	membrane organization	20	12.9	0.0001	146	846	17375	2.81	0.2057	0.0324	0.1431
		Biological process	GO:0010256	endomembrane system organization	15	9.7	0.0001	146	511	17375	3.49	0.2448	0.0345	0.1745
		Biological process	GO:0007009	plasma membrane organization	10	6.5	0.0007	146	288	17375	4.13	0.8702	0.0886	1.2621
		Biological process	GO:1990778	protein localization to cell periphery	9	5.8	0.0008	146	233	17375	4.60	0.8897	0.0844	1.3618
		Biological process	GO:1902580	single-organism cellular localization	18	11.6	0.0032	146	975	17375	2.20	0.9999	0.1960	5.5387
		Biological process	GO:0044802	single-organism membrane organization	15	9.7	0.0035	146	738	17375	2.42	1.0000	0.1873	6.1261
		Biological process	GO:0072657	protein localization to membrane	9	5.8	0.0189	146	399	17375	2.68	1.0000	0.4498	28.9601
		Biological process	GO:0072659	protein localization to plasma membrane	6	3.9	0.0338	146	214	17375	3.34	1.0000	0.5588	45.9842
		Biological process	GO:0090002	establishment of protein localization to plasma membrane	4	2.6	0.1187	146	144	17375	3.31	1.0000	0.8191	89.6226
		Biological process	GO:0090150	establishment of protein localization to membrane	4	2.6	0.3742	146	262	17375	1.82	1.0000	0.9717	99.9776
Cluster 7	2.35	Biological process	GO:0061024	membrane organization	20	12.9	0.0001	146	846	17375	2.81	0.2057	0.0324	0.1431
		Biological process	GO:0034613	cellular protein localization	26	16.8	0.0007	146	1505	17375	2.06	0.8537	0.0875	1.1883
		Biological process	GO:0016192	vesicle-mediated transport	22	14.2	0.0007	146	1171	17375	2.24	0.8743	0.0862	1.2815
		Biological process	GO:0070727	cellular macromolecule localization	26	16.8	0.0007	146	1517	17375	2.04	0.8843	0.0859	1.3325
		Biological process	GO:0033036	macromolecule localization	36	23.2	0.0020	146	2573	17375	1.67	0.9967	0.1466	3.4871
		Biological process	GO:0051641	cellular localization	33	21.3	0.0026	146	2323	17375	1.69	0.9995	0.1697	4.6306
		Biological process	GO:1902580	single-organism cellular localization	18	11.6	0.0032	146	975	17375	2.20	0.9999	0.1960	5.5387
		Biological process	GO:0008104	protein localization	32	20.6	0.0032	146	2255	17375	1.69	0.9999	0.1943	5.6136
		Biological process	GO:0044802	single-organism membrane organization	15	9.7	0.0035	146	738	17375	2.42	1.0000	0.1873	6.1261
		Biological process	GO:0045184	establishment of protein localization	24	15.5	0.0212	146	1770	17375	1.61	1.0000	0.4643	31.9133
		Biological process	GO:0015031	protein transport	22	14.2	0.0252	146	1602	17375	1.63	1.0000	0.5033	36.6834

		Biological process	GO:0051649	establishment of localization in cell	22	14.2	0.0496	146	1724	17375	1.52	1.0000	0.6288	59.8416
		Biological process	GO:0046907	intracellular transport	17	11.0	0.0770	146	1303	17375	1.55	1.0000	0.7329	76.2341
		Biological process	GO:0006886	intracellular protein transport	12	7.7	0.1327	146	901	17375	1.58	1.0000	0.8426	92.2157
Cluster 8	2.34	Biological process	GO:0006082	organic acid metabolic process	20	12.9	0.0001	146	875	17375	2.72	0.3009	0.0390	0.2224
		Biological process	GO:1901605	alpha-amino acid metabolic process	9	5.8	0.0002	146	194	17375	5.52	0.4788	0.0575	0.4044
		Biological process	GO:1901607	alpha-amino acid biosynthetic process	6	3.9	0.0004	146	73	17375	9.78	0.6454	0.0714	0.6427
		Biological process	GO:0008652	cellular amino acid biosynthetic process	5	3.2	0.0015	146	59	17375	10.09	0.9861	0.1289	2.6263
		Biological process	GO:0006520	cellular amino acid metabolic process	8	5.2	0.0022	146	215	17375	4.43	0.9983	0.1504	3.8765
		Biological process	GO:0043436	oxoacid metabolic process	16	10.3	0.0025	146	791	17375	2.41	0.9994	0.1677	4.4634
		Biological process	GO:0019752	carboxylic acid metabolic process	15	9.7	0.0062	146	787	17375	2.27	1.0000	0.2606	10.4871
		Biological process	GO:0044283	small molecule biosynthetic process	10	6.5	0.0138	146	453	17375	2.63	1.0000	0.3909	22.1036
		Biological process	GO:0009067	aspartate family amino acid biosynthetic process	3	1.9	0.0212	146	27	17375	13.22	1.0000	0.4672	31.8711
		Biological process	GO:0009066	aspartate family amino acid metabolic process	3	1.9	0.0608	146	48	17375	7.44	1.0000	0.6702	67.5188
		Biological process	GO:0016053	organic acid biosynthetic process	6	3.9	0.0653	146	258	17375	2.77	1.0000	0.6859	70.1852
		Biological process	GO:0046394	carboxylic acid biosynthetic process	5	3.2	0.1444	146	242	17375	2.46	1.0000	0.8583	93.8928

Table S16: D-II Female_Effect of FGR (only Good Nutrition)

Rank	Score	Category	Term ID	Term name	Count	%	PValue	List Total	Pop Hits	Pop Total	Fold Enrichment	Bonferroni	Benjamini	FDR
Cluster 1	59.04	Cellular component	GO:0043230	extracellular organelle	235	56.1	0.0000	397	2666	13535	3.01	0.0000	0.0000	0.0000
		Cellular component	GO:0070062	extracellular exosome	234	55.8	0.0000	397	2646	13535	3.02	0.0000	0.0000	0.0000
		Cellular component	GO:1903561	extracellular vesicle	234	55.8	0.0000	397	2661	13535	3.00	0.0000	0.0000	0.0000
		Cellular component	GO:0031988	membrane-bounded vesicle	245	58.5	0.0000	397	3321	13535	2.52	0.0000	0.0000	0.0000
		Cellular component	GO:0044421	extracellular region part	251	59.9	0.0000	397	3710	13535	2.31	0.0000	0.0000	0.0000
		Cellular component	GO:0005576	extracellular region	254	60.6	0.0000	397	4113	13535	2.11	0.0000	0.0000	0.0000
Cluster 2	23.72	Biological process	GO:0006082	organic acid metabolic process	87	20.8	0.0000	408	875	17375	4.23	0.0000	0.0000	0.0000
		Biological process	GO:0019752	carboxylic acid metabolic process	79	18.9	0.0000	408	787	17375	4.27	0.0000	0.0000	0.0000
		Biological process	GO:0043436	oxoacid metabolic process	79	18.9	0.0000	408	791	17375	4.25	0.0000	0.0000	0.0000
		Biological process	GO:0032787	monocarboxylic acid metabolic process	41	9.8	0.0000	408	535	17375	3.26	0.0000	0.0000	0.0000
Cluster 3	13.94	Molecular function	GO:0048037	cofactor binding	40	9.5	0.0000	398	308	16438	5.36	0.0000	0.0000	0.0000
		Molecular function	GO:0050662	coenzyme binding	32	7.6	0.0000	398	212	16438	6.23	0.0000	0.0000	0.0000
		Molecular function	GO:0051287	NAD binding	16	3.8	0.0000	398	72	16438	9.18	0.0000	0.0000	0.0000
Cluster 4	13.07	Biological process	GO:0044712	single-organism catabolic process	69	16.5	0.0000	408	701	17375	4.19	0.0000	0.0000	0.0000
		Biological process	GO:0044282	small molecule catabolic process	38	9.1	0.0000	408	270	17375	5.99	0.0000	0.0000	0.0000
		Biological process	GO:0016054	organic acid catabolic process	29	6.9	0.0000	408	192	17375	6.43	0.0000	0.0000	0.0000
		Biological process	GO:0046395	carboxylic acid catabolic process	26	6.2	0.0000	408	169	17375	6.55	0.0000	0.0000	0.0000
		Biological process	GO:1901565	organonitrogen compound catabolic process	28	6.7	0.0000	408	272	17375	4.38	0.0000	0.0000	0.0000
		Biological process	GO:0009063	cellular amino acid catabolic process	15	3.6	0.0000	408	66	17375	9.68	0.0000	0.0000	0.0000
		Biological process	GO:1901606	alpha-amino acid catabolic process	10	2.4	0.0000	408	62	17375	6.87	0.0660	0.0006	0.0251
Cluster 5	11.47	Biological process	GO:0010035	response to inorganic substance	55	13.1	0.0000	408	677	17375	3.46	0.0000	0.0000	0.0000
		Biological process	GO:0010038	response to metal ion	40	9.5	0.0000	408	448	17375	3.80	0.0000	0.0000	0.0000
		Biological process	GO:1990267	response to transition metal nanoparticle	22	5.3	0.0000	408	199	17375	4.71	0.0000	0.0000	0.0000
Cluster 6	8.90	Molecular function	GO:0036094	small molecule binding	132	31.5	0.0000	398	2506	16438	2.18	0.0000	0.0000	0.0000
		Molecular function	GO:0000166	nucleotide binding	120	28.6	0.0000	398	2307	16438	2.15	0.0000	0.0000	0.0000
		Molecular function	GO:1901265	nucleoside phosphate binding	120	28.6	0.0000	398	2307	16438	2.15	0.0000	0.0000	0.0000
		Molecular function	GO:0032550	purine ribonucleoside binding	83	19.8	0.0000	398	1763	16438	1.94	0.0000	0.0000	0.0000
		Molecular function	GO:0001883	purine nucleoside binding	83	19.8	0.0000	398	1766	16438	1.94	0.0000	0.0000	0.0000
		Molecular function	GO:0032549	ribonucleoside binding	83	19.8	0.0000	398	1766	16438	1.94	0.0000	0.0000	0.0000
		Molecular function	GO:0001882	nucleoside binding	83	19.8	0.0000	398	1773	16438	1.93	0.0000	0.0000	0.0000
		Molecular function	GO:0017076	purine nucleotide binding	84	20.0	0.0000	398	1809	16438	1.92	0.0000	0.0000	0.0000
		Molecular function	GO:0032555	purine ribonucleotide binding	83	19.8	0.0000	398	1798	16438	1.91	0.0000	0.0000	0.0000
		Molecular function	GO:0032553	ribonucleotide binding	83	19.8	0.0000	398	1815	16438	1.89	0.0000	0.0000	0.0000
		Molecular function	GO:0035639	purine ribonucleoside triphosphate binding	81	19.3	0.0000	398	1753	16438	1.91	0.0000	0.0000	0.0000
		Molecular function	GO:0097367	carbohydrate derivative binding	91	21.7	0.0000	398	2152	16438	1.75	0.0001	0.0000	0.0001
		Molecular function	GO:0030554	adenyl nucleotide binding	67	16.0	0.0000	398	1449	16438	1.91	0.0004	0.0000	0.0005
		Molecular function	GO:0032559	adenyl ribonucleotide binding	66	15.8	0.0000	398	1440	16438	1.89	0.0006	0.0000	0.0009
		Molecular function	GO:0005524	ATP binding	64	15.3	0.0000	398	1403	16438	1.88	0.0012	0.0000	0.0017
		Molecular function	GO:0097159	organic cyclic compound binding	177	42.2	0.0000	398	5726	16438	1.28	0.0510	0.0012	0.0750
		Molecular function	GO:1901363	heterocyclic compound binding	174	41.5	0.0001	398	5645	16438	1.27	0.0751	0.0018	0.1119
Cluster 7	8.85	Biological process	GO:0006790	sulfur compound metabolic process	35	8.4	0.0000	408	298	17375	5.00	0.0000	0.0000	0.0000
		Biological process	GO:0006575	cellular modified amino acid metabolic process	23	5.5	0.0000	408	145	17375	6.75	0.0000	0.0000	0.0000
		Biological process	GO:0006749	glutathione metabolic process	15	3.6	0.0000	408	62	17375	10.30	0.0000	0.0000	0.0000
		Molecular function	GO:0016765	transferase activity, transferring alkyl or aryl (other than methyl) groups	11	2.6	0.0000	398	73	16438	6.22	0.0107	0.0003	0.0155
		Molecular function	GO:0004364	glutathione transferase activity	8	1.9	0.0001	398	43	16438	7.68	0.0743	0.0018	0.1107
Cluster 8	8.51	Biological process	GO:0044283	small molecule biosynthetic process	39	9.3	0.0000	408	453	17375	3.67	0.0000	0.0000	0.0000
		Biological process	GO:0016053	organic acid biosynthetic process	24	5.7	0.0000	408	258	17375	3.96	0.0002	0.0000	0.0001
		Biological process	GO:0046394	carboxylic acid biosynthetic process	23	5.5	0.0000	408	242	17375	4.05	0.0003	0.0000	0.0001
Cluster 9	7.87	Biological process	GO:0044248	cellular catabolic process	107	25.5	0.0000	408	1392	17375	3.27	0.0000	0.0000	0.0000
		Biological process	GO:0006508	proteolysis	90	21.5	0.0000	408	1661	17375	2.31	0.0000	0.0000	0.0000
		Cellular component	GO:0000502	proteasome complex	17	4.1	0.0000	397	72	13535	8.05	0.0000	0.0000	0.0000
		Biological process	GO:0030163	protein catabolic process	46	11.0	0.0000	408	770	17375	2.54	0.0001	0.0000	0.0000
		Biological process	GO:0044257	cellular protein catabolic process	41	9.8	0.0000	408	649	17375	2.69	0.0001	0.0000	0.0000
		Biological process	GO:0051603	proteolysis involved in cellular protein catabolic process	39	9.3	0.0000	408	619	17375	2.68	0.0003	0.0000	0.0001
		Biological process	GO:0044265	cellular macromolecule catabolic process	46	11.0	0.0000	408	825	17375	2.37	0.0006	0.0000	0.0002
		Biological process	GO:0009057	macromolecule catabolic process	51	12.2	0.0000	408	1006	17375	2.16	0.0020	0.0000	0.0007
		Biological process	GO:0019941	modification-dependent protein catabolic process	30	7.2	0.0000	408	520	17375	2.46	0.0771	0.0007	0.0295
		Biological process	GO:0043632	modification-dependent macromolecule catabolic process	30	7.2	0.0000	408	528	17375	2.42	0.1020	0.0009	0.0396
		Biological process	GO:0006511	ubiquitin-dependent protein catabolic process	29	6.9	0.0000	408	512	17375	2.41	0.1490	0.0013	0.0594
		Biological process	GO:0042176	regulation of protein catabolic process	20	4.8	0.0002	408	317	17375	2.69	0.6168	0.0056	0.3523
		Biological process	GO:0043161	proteasome-mediated ubiquitin-dependent protein catabolic process	18	4.3	0.0016	408	321	17375	2.39	0.9997	0.0315	2.9440
		Biological process	GO:0010498	proteasomal protein catabolic process	19	4.5	0.0017	408	352	17375	2.30	0.9999	0.0331	3.2068
Cluster 10	7.83	Molecular function	GO:0016903	oxidoreductase activity, acting on the aldehyde or oxo group of donors	15	3.6	0.0000	398	60	16438	10.33	0.0000	0.0000	0.0000
		Molecular function	GO:0016620	oxidoreductase activity, acting on the aldehyde or oxo group of donors, NAD or NADP as acceptor	12	2.9	0.0000	398	49	16438	10.11	0.0000	0.0000	0.0000
		Molecular function	GO:0004029	aldehyde dehydrogenase (NAD) activity	8	1.9	0.0000	398	25	16438	13.22	0.0017	0.0001	0.0024
Cluster 11	7.27	Biological process	GO:0032787	monocarboxylic acid metabolic process	41	9.8	0.0000	408	535	17375	3.26	0.0000	0.0000	0.0000
		Biological process	GO:0006629	lipid metabolic process	59	14.1	0.0000	408	1174	17375	2.14	0.0003	0.0000	0.0001
		Biological process	GO:0044255	cellular lipid metabolic process	47	11.2	0.0000	408	896	17375	2.23	0.0025	0.0000	0.0009
		Biological process	GO:0006631	fatty acid metabolic process	25	6.0	0.0000	408	354	17375	3.01	0.0178	0.0002	0.0066
Cluster 12	6.95	Cellular component	GO:0000502	proteasome complex	17	4.1	0.0000	397	72	13535	8.05	0.0000	0.0000	0.0000
		Molecular function	GO:0070003	threonine-type peptidase activity	9	2.1	0.0000	398	24	16438	15.49	0.0001	0.0000	0.0001
		Molecular function	GO:0004298	threonine-type endopeptidase activity	9	2.1	0.0000	398	24	16438	15.49	0.0001	0.0000	0.0001
		Cellular component	GO:0005839	proteasome core complex	9	2.1	0.0000	397	23	13535	13.34	0.0001	0.0000	0.0002
		Molecular function	GO:0004175	endopeptidase activity	26	6.2	0.0002	398	466	16438	2.30	0.1783	0.0043	0.2814
Cluster 13	6.82	Cellular component	GO:0070161	anchoring junction	51	12.2	0.0000	397	658	13535	2.64	0.0000	0.0000	0.0000
		Cellular component	GO:0005912	adherens junction	50	11.9	0.0000	397	643	13535	2.65	0.0000	0.0000	0.0000
		Cellular component	GO:0005925	focal adhesion	32	7.6	0.0000	397	396	13535	2.76	0.0004	0.0000	0.0009
		Cellular component	GO:0005924	cell-substrate adherens junction	32	7.6	0.0000	397	401	13535	2.72	0.0005	0.0000	0.0012
		Cellular component	GO:0030055	cell-substrate junction	32	7.6	0.0000	397	406	13535	2.69	0.0006	0.0000	0.0016
		Cellular component	GO:0030054	cell junction	63	15.0	0.0001	397	1293	13535	1.66	0.0374	0.0017	0.0931
Cluster 14	6.66	Biological process	GO:0006508	proteolysis	90	21.5	0.0000	408	1661	17375	2.31	0.0000	0.0000	0.0000
		Molecular function	GO:0008233	peptidase activity	37	8.8	0.0000	398	688	16438	2.22	0.0128	0.0003	0.0185
		Molecular function	GO:0070011	peptidase activity, acting on L-amino acid peptides	35	8.4	0.0000	398	651	16438	2.22	0.0234	0.0006	0.0339
		Molecular function	GO:0004175	endopeptidase activity	26	6.2	0.0002	398	466	16438	2.30	0.1783	0.0043	0.2814
Cluster 15	6.11	Biological process	GO:0031667	response to nutrient levels	38	9.1	0.0000	408	603	17375	2.68	0.0005	0.0000	0.0002
		Biological process	GO:0009605	response to external stimulus	92	22.0	0.0000	408	2306	17375	1.70	0.0013	0.0000	0.0005
		Biological process	GO:0009991	response to extracellular stimulus	38	9.1	0.0000	408	630	17375	2.57	0.0015	0.0000	0.0006
		Biological process	GO:0007584	response to nutrient	24	5.7	0.0000	408	304	17375	3.36	0.0045	0.0001	0.0017
		Biological process	GO:0033273	response to vitamin	15	3.6	0.0000	408	169	17375	3.78	0.2104	0.0017	0.0869
Cluster 16	5.54	Biological process	GO:0009636	response to toxic substance	26	6.2	0.0000	408	257	17375	4.31	0.0000	0.0000	0.0000
		Molecular function	GO:0016209	antioxidant activity	14	3.3	0.0000	398	79	16438	7.32	0.0001	0.0000	0.0001
		Biological process	GO:0098869	cellular oxidant detoxification	13	3.1	0.0000	408	83	17375	6.67	0.0026	0.0000	0.0010
		Biological process	GO:1990748	cellular detoxification	13	3.1	0.0000	408	86	17375	6.44	0.0039	0.0000	0.0014
		Molecular function	GO:0016684	oxidoreductase activity, acting on peroxide as acceptor	10	2.4	0.0000	398	45	16438	9.18	0.0012	0.0000	0.0017
		Biological process	GO:0098754	detoxification	13	3.1	0.0000	408	90	17375	6.15	0.0063	0.0001	0.0023
		Biological process	GO:0072593	reactive oxygen species metabolic process	20	4.8	0.0000	408	242	17375	3.52	0.0234	0.0002	0.0087
		Biological process	GO:0042744	hydrogen peroxide catabolic process	7	1.7	0.0000	408	21	17375	14.20	0.0327	0.0003	0.0122
		Molecular function	GO:0004601	peroxidase activity	9	2.1	0.0000	398	43	16438	8.64	0.0083	0.0002	0.0120
		Biological process	GO:0042743	hydrogen peroxide metabolic process	8	1.9	0.0000	408	40	17375	8.52	0.1668	0.0014	0.0671
		Molecular function	GO:0051920	peroxiredoxin activity	4	1.0	0.0007	398	8	16438	20.65	0.5504	0.0147	1.1400
		Molecular function	GO:0004602	glutathione peroxidase activity	5	1.2	0.0014	398	21	16438	9.83	0.8018	0.0266	2.2946
Cluster 17	5.46	Molecular function	GO:0042802	identical protein binding	67	16.0	0.0000	398	1325	16438	2.09	0.0000	0.0000	0.0000
		Molecular function	GO:0042803	protein homodimerization activity	41	9.8	0.0000	398	805	16438	2.10	0.0137	0.0004	0.0198
		Molecular function	GO:0046983	protein dimerization activity	52	12.4	0.0003	398	1283	16438	1.67	0.2753	0.0065	0.4608
Cluster 18	5.40	Molecular function	GO:0043167	ion binding	132	31.5	0.0000	398	3722	16438	1.46	0.0013	0.0000	0.0018
		Molecular function	GO:0046872	metal ion binding	123	29.4	0.0000	398	3501	16438	1.45	0.0061	0.0002	0.0087
		Molecular function	GO:0043169	cation binding	123	29.4	0.0000	398	3545	16438	1.43	0.0113	0.0003	0.0163
Cluster 19	5.13	Biological process	GO:0035383	thioester metabolic process	12	2.9	0.0000	408	79	17375	6.47	0.0114	0.0001	0.0042
		Biological process	GO:0006637	acyl-CoA metabolic process	12	2.9	0.0000	408	79	17375	6.47	0.0114	0.0001	0.0042
		Biological process	GO:0006084	acetyl-CoA metabolic process	7	1.7	0.0001	408	32	17375	9.32	0.3604	0.0030	0.1644

Cluster 20	5.07	Cellular component	GO:0070161	anchoring junction	51	12.2	0.0000	397	658	13535	2.64	0.0000	0.0000	0.0000
		Cellular component	GO:0005912	adherens junction	50	11.9	0.0000	397	643	13535	2.65	0.0000	0.0000	0.0000
		Molecular function	GO:0098631	protein binding involved in cell adhesion	21	5.0	0.0000	398	232	16438	3.74	0.0011	0.0000	0.0015
		Molecular function	GO:0098641	cadherin binding involved in cell-cell adhesion	20	4.8	0.0000	398	220	16438	3.75	0.0019	0.0001	0.0027
		Molecular function	GO:0045296	cadherin binding	21	5.0	0.0000	398	243	16438	3.57	0.0022	0.0001	0.0031
		Molecular function	GO:0098632	protein binding involved in cell-cell adhesion	20	4.8	0.0000	398	227	16438	3.64	0.0031	0.0001	0.0044
		Molecular function	GO:0050839	cell adhesion molecule binding	27	6.4	0.0000	398	393	16438	2.84	0.0041	0.0001	0.0058
		Cellular component	GO:0005913	cell-cell adherens junction	21	5.0	0.0001	397	269	13535	2.66	0.0751	0.0031	0.1906
		Cellular component	GO:0005911	cell-cell junction	34	8.1	0.0005	397	609	13535	1.90	0.2562	0.0105	0.7207
		Biological process	GO:0022610	biological adhesion	51	12.2	0.0053	408	1478	17375	1.47	1.0000	0.0848	9.7291
		Biological process	GO:0098609	cell-cell adhesion	39	9.3	0.0060	408	1060	17375	1.57	1.0000	0.0925	10.8697
		Biological process	GO:0007155	cell adhesion	50	11.9	0.0074	408	1468	17375	1.45	1.0000	0.1084	13.2604
Cluster 21	4.81	Biological process	GO:0065003	macromolecular complex assembly	71	16.9	0.0000	408	1563	17375	1.93	0.0004	0.0000	0.0002
		Biological process	GO:0006461	protein complex assembly	60	14.3	0.0000	408	1268	17375	2.02	0.0015	0.0000	0.0006
		Biological process	GO:0070271	protein complex biogenesis	60	14.3	0.0000	408	1269	17375	2.01	0.0016	0.0000	0.0006
		Biological process	GO:0071822	protein complex subunit organization	63	15.0	0.0000	408	1407	17375	1.91	0.0048	0.0001	0.0018
		Biological process	GO:0043933	macromolecular complex subunit organization	81	19.3	0.0000	408	2213	17375	1.56	0.2002	0.0017	0.0822
		Biological process	GO:0022607	cellular component assembly	85	20.3	0.0002	408	2456	17375	1.47	0.6155	0.0056	0.3511
		Biological process	GO:0034622	cellular macromolecular complex assembly	39	9.3	0.0004	408	906	17375	1.83	0.8581	0.0101	0.7159
		Biological process	GO:0051259	protein oligomerization	25	6.0	0.0016	408	527	17375	2.02	0.9997	0.0313	2.9443
		Biological process	GO:0044085	cellular component biogenesis	87	20.8	0.0016	408	2705	17375	1.37	0.9997	0.0314	2.9601
Cluster 22	4.64	Biological process	GO:0010033	response to organic substance	130	31.0	0.0000	408	3067	17375	1.81	0.0000	0.0000	0.0000
		Biological process	GO:1901698	response to nitrogen compound	67	16.0	0.0000	408	1171	17375	2.44	0.0000	0.0000	0.0000
		Biological process	GO:0009719	response to endogenous stimulus	89	21.2	0.0000	408	1849	17375	2.05	0.0000	0.0000	0.0000
		Biological process	GO:0014070	response to organic cyclic compound	70	16.7	0.0000	408	1302	17375	2.29	0.0000	0.0000	0.0000
		Biological process	GO:0010243	response to organonitrogen compound	61	14.6	0.0000	408	1067	17375	2.43	0.0000	0.0000	0.0000
		Biological process	GO:0009725	response to hormone	60	14.3	0.0000	408	1069	17375	2.39	0.0000	0.0000	0.0000
		Biological process	GO:1901700	response to oxygen-containing compound	86	20.5	0.0000	408	1872	17375	1.96	0.0000	0.0000	0.0000
		Biological process	GO:0070887	cellular response to chemical stimulus	95	22.7	0.0000	408	2604	17375	1.55	0.0394	0.0004	0.0148
		Biological process	GO:0033993	response to lipid	53	12.6	0.0000	408	1199	17375	1.88	0.0614	0.0006	0.0233
		Biological process	GO:1901652	response to peptide	31	7.4	0.0000	408	555	17375	2.38	0.0992	0.0009	0.0384
		Biological process	GO:0043434	response to peptide hormone	29	6.9	0.0000	408	501	17375	2.47	0.1034	0.0009	0.0401
		Biological process	GO:0048545	response to steroid hormone	27	6.4	0.0003	408	523	17375	2.20	0.7582	0.0078	0.5211
		Biological process	GO:0071310	cellular response to organic substance	72	17.2	0.0015	408	2143	17375	1.43	0.9996	0.0310	2.8710
		Biological process	GO:1901701	cellular response to oxygen-containing compound	42	10.0	0.0018	408	1088	17375	1.64	0.9999	0.0348	3.3782
		Biological process	GO:1901699	cellular response to nitrogen compound	28	6.7	0.0031	408	652	17375	1.83	1.0000	0.0547	5.7693
		Biological process	GO:0071417	cellular response to organonitrogen compound	25	6.0	0.0042	408	569	17375	1.87	1.0000	0.0697	7.6469
		Biological process	GO:0071407	cellular response to organic cyclic compound	26	6.2	0.0077	408	631	17375	1.75	1.0000	0.1117	13.7361
		Biological process	GO:0071495	cellular response to endogenous stimulus	43	10.3	0.0156	408	1277	17375	1.43	1.0000	0.1810	26.0153
		Biological process	GO:0071396	cellular response to lipid	24	5.7	0.0187	408	614	17375	1.66	1.0000	0.2028	30.2569
		Biological process	GO:0032868	response to insulin	14	3.3	0.0223	408	295	17375	2.02	1.0000	0.2323	34.9457
		Biological process	GO:0071383	cellular response to steroid hormone stimulus	13	3.1	0.0258	408	270	17375	2.05	1.0000	0.2543	39.3403
		Biological process	GO:0032870	cellular response to hormone stimulus	25	6.0	0.0270	408	671	17375	1.59	1.0000	0.2606	40.6752
		Biological process	GO:1901653	cellular response to peptide	13	3.1	0.1063	408	341	17375	1.62	1.0000	0.5858	88.3219
		Biological process	GO:0071375	cellular response to peptide hormone stimulus	11	2.6	0.2032	408	314	17375	1.49	1.0000	0.7640	98.6978
		Biological process	GO:0032869	cellular response to insulin stimulus	6	1.4	0.5691	408	215	17375	1.19	1.0000	0.9776	100.0000
Cluster 23	4.60	Biological process	GO:0055086	nucleobase-containing small molecule metabolic process	50	11.9	0.0000	408	672	17375	3.17	0.0000	0.0000	0.0000
		Biological process	GO:0006753	nucleoside phosphate metabolic process	46	11.0	0.0000	408	620	17375	3.16	0.0000	0.0000	0.0000
		Biological process	GO:1901657	glycosyl compound metabolic process	34	8.1	0.0000	408	380	17375	3.81	0.0000	0.0000	0.0000
		Biological process	GO:0009117	nucleotide metabolic process	44	10.5	0.0000	408	613	17375	3.06	0.0000	0.0000	0.0000
		Biological process	GO:0009116	nucleoside metabolic process	31	7.4	0.0000	408	357	17375	3.70	0.0000	0.0000	0.0000
		Biological process	GO:0019637	organophosphate metabolic process	55	13.1	0.0000	408	961	17375	2.44	0.0000	0.0000	0.0000
		Biological process	GO:0009141	nucleoside triphosphate metabolic process	26	6.2	0.0000	408	271	17375	4.09	0.0000	0.0000	0.0000
		Biological process	GO:0009123	nucleoside monophosphate metabolic process	26	6.2	0.0000	408	285	17375	3.89	0.0001	0.0000	0.0000
		Biological process	GO:0046128	purine ribonucleoside metabolic process	27	6.4	0.0000	408	314	17375	3.66	0.0001	0.0000	0.0001
		Biological process	GO:0009119	ribonucleoside metabolic process	28	6.7	0.0000	408	338	17375	3.53	0.0002	0.0000	0.0001
		Biological process	GO:0042278	purine nucleoside metabolic process	27	6.4	0.0000	408	318	17375	3.62	0.0002	0.0000	0.0001
		Biological process	GO:0009167	purine ribonucleoside monophosphate metabolic process	24	5.7	0.0000	408	261	17375	3.92	0.0003	0.0000	0.0001
		Biological process	GO:0009126	purine nucleoside monophosphate metabolic process	24	5.7	0.0000	408	262	17375	3.90	0.0003	0.0000	0.0001
		Biological process	GO:0019693	ribose phosphate metabolic process	34	8.1	0.0000	408	501	17375	2.89	0.0005	0.0000	0.0002
		Biological process	GO:0009161	ribonucleoside monophosphate metabolic process	24	5.7	0.0000	408	271	17375	3.77	0.0006	0.0000	0.0002
		Biological process	GO:0009144	purine nucleoside triphosphate metabolic process	23	5.5	0.0000	408	250	17375	3.92	0.0006	0.0000	0.0002
		Biological process	GO:0072521	purine-containing compound metabolic process	35	8.4	0.0000	408	540	17375	2.76	0.0009	0.0000	0.0003
		Biological process	GO:0009205	purine ribonucleoside triphosphate metabolic process	22	5.3	0.0000	408	242	17375	3.87	0.0014	0.0000	0.0005
		Biological process	GO:0009199	ribonucleoside triphosphate metabolic process	22	5.3	0.0000	408	248	17375	3.78	0.0022	0.0000	0.0008
		Biological process	GO:0006163	purine nucleotide metabolic process	31	7.4	0.0000	408	499	17375	2.65	0.0130	0.0001	0.0048
		Biological process	GO:0009259	ribonucleotide metabolic process	30	7.2	0.0000	408	490	17375	2.61	0.0259	0.0003	0.0097
		Biological process	GO:0046034	ATP metabolic process	19	4.5	0.0000	408	223	17375	3.63	0.0282	0.0003	0.0105
		Biological process	GO:0009150	purine ribonucleotide metabolic process	29	6.9	0.0000	408	476	17375	2.59	0.0418	0.0004	0.0157
		Biological process	GO:1901135	carbohydrate derivative metabolic process	47	11.2	0.0001	408	1072	17375	1.87	0.2418	0.0020	0.1018
		Biological process	GO:1901659	glycosyl compound biosynthetic process	12	2.9	0.0001	408	115	17375	4.44	0.3456	0.0029	0.1559
		Biological process	GO:0009127	purine nucleoside monophosphate biosynthetic process	10	2.4	0.0001	408	78	17375	5.46	0.3568	0.0030	0.1623
		Biological process	GO:0009168	purine ribonucleoside monophosphate biosynthetic process	10	2.4	0.0001	408	78	17375	5.46	0.3568	0.0030	0.1623
		Biological process	GO:0009156	ribonucleoside monophosphate biosynthetic process	10	2.4	0.0002	408	88	17375	4.84	0.6757	0.0063	0.4135
		Biological process	GO:0042455	ribonucleoside biosynthetic process	11	2.6	0.0003	408	113	17375	4.15	0.8137	0.0089	0.6164
		Biological process	GO:0009163	nucleoside biosynthetic process	11	2.6	0.0003	408	113	17375	4.15	0.8137	0.0089	0.6164
		Biological process	GO:0042451	purine nucleoside biosynthetic process	10	2.4	0.0004	408	94	17375	4.53	0.8433	0.0097	0.6798
		Biological process	GO:0046129	purine ribonucleoside biosynthetic process	10	2.4	0.0004	408	94	17375	4.53	0.8433	0.0097	0.6798
		Biological process	GO:0009124	nucleoside monophosphate biosynthetic process	10	2.4	0.0005	408	97	17375	4.39	0.9039	0.0115	0.8582
		Biological process	GO:0046390	ribose phosphate biosynthetic process	16	3.8	0.0013	408	260	17375	2.62	0.9985	0.0273	2.3631
		Biological process	GO:0009165	nucleotide biosynthetic process	18	4.3	0.0013	408	315	17375	2.43	0.9987	0.0276	2.4183
		Biological process	GO:1901293	nucleoside phosphate biosynthetic process	18	4.3	0.0014	408	317	17375	2.42	0.9992	0.0289	2.5886
		Biological process	GO:0090407	organophosphate biosynthetic process	23	5.5	0.0029	408	490	17375	2.00	1.0000	0.0515	5.3608
		Biological process	GO:0046033	AMP metabolic process	4	1.0	0.0057	408	16	17375	10.65	1.0000	0.0890	10.3360
		Biological process	GO:0009260	ribonucleotide biosynthetic process	14	3.3	0.0078	408	257	17375	2.32	1.0000	0.1109	13.8210
		Biological process	GO:0009142	nucleoside triphosphate biosynthetic process	7	1.7	0.0112	408	80	17375	3.73	1.0000	0.1446	19.2965
		Biological process	GO:0009152	purine ribonucleotide biosynthetic process	13	3.1	0.0131	408	245	17375	2.26	1.0000	0.1606	22.2733
		Biological process	GO:0009145	purine nucleoside triphosphate biosynthetic process	6	1.4	0.0159	408	63	17375	4.06	1.0000	0.1828	26.4154
		Biological process	GO:0006164	purine nucleotide biosynthetic process	13	3.1	0.0174	408	255	17375	2.17	1.0000	0.1953	28.5306
		Biological process	GO:0072522	purine-containing compound biosynthetic process	13	3.1	0.0194	408	259	17375	2.14	1.0000	0.2084	31.2667
		Biological process	GO:0009206	purine ribonucleoside triphosphate biosynthetic process	5	1.2	0.0573	408	62	17375	3.43	1.0000	0.4167	67.5949
		Biological process	GO:0009201	ribonucleoside triphosphate biosynthetic process	5	1.2	0.0752	408	68	17375	3.13	1.0000	0.4908	77.5253
		Biological process	GO:0006754	ATP biosynthetic process	4	1.0	0.1120	408	50	17375	3.41	1.0000	0.5988	89.6544
		Biological process	GO:1901137	carbohydrate derivative biosynthetic process	21	5.0	0.1195	408	642	17375	1.39	1.0000	0.6172	91.2035
		Biological process	GO:0015985	energy coupled proton transport, down electrochemical gradient	3	0.7	0.1311	408	27	17375	4.73	1.0000	0.6406	93.1776
		Biological process	GO:0015986	ATP synthesis coupled proton transport	3	0.7	0.1311	408	27	17375	4.73	1.0000	0.6406	93.1776
		Biological process	GO:0015992	proton transport	5	1.2	0.4681	408	150	17375	1.42	1.0000	0.9527	99.9994
		Biological process	GO:0006818	hydrogen transport	5	1.2	0.4732	408	151	17375	1.41	1.0000	0.9542	99.9995
		Biological process	GO:1902600	hydrogen ion transmembrane transport	4	1.0	0.5190	408	117	17375	1.46	1.0000	0.9670	99.9999
Cluster 24	4.04	Biological process	GO:0015980	energy derivation by oxidation of organic compounds	22	5.3	0.0000	408	231	17375	4.06	0.0007	0.0000	0.0002
		Biological process	GO:0045333	cellular respiration	14	3.3	0.0000	408	146	17375	4.08	0.1878	0.0016	0.0765
		Biological process	GO:0006099	tricarboxylic acid cycle	7	1.7	0.0000	408	29	17375	10.28	0.2201	0.0018	0.0915
		Biological process	GO:0043648	dicarboxylic acid metabolic process	11	2.6	0.0001	408	92	17375	5.09	0.2602	0.0021	0.1108
		Biological process	GO:0009060	aerobic respiration	9	2.1	0.0001	408	59	17375	6.50	0.2901	0.0024	0.1260
		Biological process	GO:0006734	NADH metabolic process	6	1.4	0.0001	408	21	17375	12.17	0.4134	0.0034	0.1961
		Biological process	GO:0019674	NAD metabolic process	7	1.7	0.0003	408	40	17375	7.45	0.8015	0.0087	0.5932
		Cellular component	GO:1990204	oxidoreductase complex	11	2.6	0.0021	397	116	13535	3.23	0.7241	0.0372	3.0984
		Biological process	GO:0006103	2-oxoglutarate metabolic process	4	1.0	0.0068	408	17	17375	10.02	1.0000	0.1023	12.2147
Cluster 25	3.69	Biological process	GO:0001889	liver development	17	4.1	0.0001	408	222	17375	3.26	0.3125	0.0026	0.1378
		Biological process	GO:0061008	hepaticobiliary system development	17	4.1	0.0001	408	225	17375	3.22	0.3554	0.0030	0.1615
		Biological process	GO:0048732	gland development	27	6.4	0.0014	408	584	17375	1.97	0.9993	0.0292	2.6217
Cluster 26	3.61	Biological process	GO:0051186	cofactor metabolic process	43	10.3	0.0000	408	373	17375	4.91	0.0000	0.0000	0.0000

		Biological process	GO:0006732	coenzyme metabolic process	35	8.4	0.0000	408	307	17375	4.86	0.0000	0.0000	0.0000
		Biological process	GO:0072524	pyridine-containing compound metabolic process	18	4.3	0.0000	408	135	17375	5.68	0.0001	0.0000	0.0000
		Biological process	GO:0006733	oxidoreduction coenzyme metabolic process	18	4.3	0.0000	408	145	17375	5.29	0.0003	0.0000	0.0001
		Biological process	GO:0046496	nicotinamide nucleotide metabolic process	17	4.1	0.0000	408	129	17375	5.61	0.0003	0.0000	0.0001
		Biological process	GO:0019362	pyridine nucleotide metabolic process	17	4.1	0.0000	408	129	17375	5.61	0.0003	0.0000	0.0001
		Biological process	GO:0009205	purine ribonucleoside triphosphate metabolic process	22	5.3	0.0000	408	242	17375	3.87	0.0014	0.0000	0.0005
		Biological process	GO:0009199	ribonucleoside triphosphate metabolic process	22	5.3	0.0000	408	248	17375	3.78	0.0022	0.0000	0.0008
		Biological process	GO:0046034	ATP metabolic process	19	4.5	0.0000	408	223	17375	3.63	0.0282	0.0003	0.0105
		Biological process	GO:0006734	NADH metabolic process	6	1.4	0.0001	408	21	17375	12.17	0.4134	0.0034	0.1961
		Biological process	GO:0019674	NAD metabolic process	7	1.7	0.0003	408	40	17375	7.45	0.8015	0.0087	0.5932
		Biological process	GO:0006090	pyruvate metabolic process	10	2.4	0.0006	408	100	17375	4.26	0.9467	0.0142	1.0731
		Biological process	GO:0016052	carbohydrate catabolic process	11	2.6	0.0009	408	129	17375	3.63	0.9916	0.0216	1.7456
		Biological process	GO:0009185	ribonucleoside diphosphate metabolic process	9	2.1	0.0015	408	93	17375	4.12	0.9996	0.0311	2.8648
		Biological process	GO:0009132	nucleoside diphosphate metabolic process	10	2.4	0.0015	408	115	17375	3.70	0.9997	0.0312	2.8944
		Biological process	GO:0046364	monosaccharide biosynthetic process	8	1.9	0.0016	408	73	17375	4.67	0.9997	0.0315	2.9849
		Biological process	GO:0046939	nucleotide phosphorylation	9	2.1	0.0020	408	97	17375	3.95	1.0000	0.0376	3.7343
		Biological process	GO:0046031	ADP metabolic process	8	1.9	0.0027	408	80	17375	4.26	1.0000	0.0487	5.0128
		Biological process	GO:0043471	regulation of cellular carbohydrate catabolic process	4	1.0	0.0047	408	15	17375	11.36	1.0000	0.0767	8.6207
		Biological process	GO:0009135	purine nucleoside diphosphate metabolic process	8	1.9	0.0052	408	90	17375	3.79	1.0000	0.0831	9.4544
		Biological process	GO:0006165	nucleoside diphosphate phosphorylation	8	1.9	0.0052	408	90	17375	3.79	1.0000	0.0831	9.4544
		Biological process	GO:0009179	purine ribonucleoside diphosphate metabolic process	8	1.9	0.0052	408	90	17375	3.79	1.0000	0.0831	9.4544
		Biological process	GO:0044275	cellular carbohydrate catabolic process	5	1.2	0.0071	408	33	17375	6.45	1.0000	0.1054	12.7197
		Biological process	GO:0010675	regulation of cellular carbohydrate metabolic process	10	2.4	0.0072	408	145	17375	2.94	1.0000	0.1065	12.8950
		Biological process	GO:0044724	single-organism carbohydrate catabolic process	8	1.9	0.0156	408	111	17375	3.07	1.0000	0.1807	25.9205
		Biological process	GO:0006096	glycolytic process	6	1.4	0.0254	408	71	17375	3.60	1.0000	0.2515	38.8197
		Biological process	GO:0019319	hexose biosynthetic process	6	1.4	0.0254	408	71	17375	3.60	1.0000	0.2515	38.8197
		Biological process	GO:0043470	regulation of carbohydrate catabolic process	5	1.2	0.0257	408	48	17375	4.44	1.0000	0.2535	39.1713
		Biological process	GO:0006757	ATP generation from ADP	6	1.4	0.0268	408	72	17375	3.55	1.0000	0.2600	40.4644
		Biological process	GO:0006094	gluconeogenesis	5	1.2	0.0784	408	69	17375	3.09	1.0000	0.5014	78.9763
		Molecular function	GO:0016866	intramolecular transferase activity	3	0.7	0.1377	398	27	16438	4.59	1.0000	0.6493	90.6955
		Biological process	GO:0051196	regulation of coenzyme metabolic process	3	0.7	0.3018	408	47	17375	2.72	1.0000	0.8637	99.8955
		Biological process	GO:0051193	regulation of cofactor metabolic process	3	0.7	0.3105	408	48	17375	2.66	1.0000	0.8692	99.9177
		Cellular component	GO:0036126	sperm flagellum	3	0.7	0.7446	397	90	13535	1.14	1.0000	0.9852	100.0000
		Cellular component	GO:0031514	motile cilium	4	1.0	0.7935	397	143	13535	0.95	1.0000	0.9898	100.0000
		Cellular component	GO:0044441	ciliary part	7	1.7	0.8981	397	312	13535	0.76	1.0000	0.9977	100.0000
Cluster 27	3.60	Biological process	GO:0046394	carboxylic acid biosynthetic process	23	5.5	0.0000	408	242	17375	4.05	0.0003	0.0000	0.0001
		Biological process	GO:0072330	monocarboxylic acid biosynthetic process	11	2.6	0.0069	408	170	17375	2.76	1.0000	0.1029	12.3203
		Biological process	GO:0006633	fatty acid biosynthetic process	8	1.9	0.0371	408	133	17375	2.56	1.0000	0.3213	51.4808
Cluster 28	3.44	Biological process	GO:0006091	generation of precursor metabolites and energy	30	7.2	0.0000	408	330	17375	3.87	0.0000	0.0000	0.0000
		Biological process	GO:0005996	monosaccharide metabolic process	20	4.8	0.0000	408	222	17375	3.84	0.0066	0.0001	0.0024
		Biological process	GO:0016051	carbohydrate biosynthetic process	17	4.1	0.0000	408	181	17375	4.00	0.0296	0.0003	0.0111
		Biological process	GO:0019318	hexose metabolic process	18	4.3	0.0000	408	212	17375	3.62	0.0552	0.0005	0.0209
		Biological process	GO:0005975	carbohydrate metabolic process	36	8.6	0.0000	408	705	17375	2.17	0.1199	0.0010	0.0470
		Biological process	GO:0006006	glucose metabolic process	16	3.8	0.0000	408	183	17375	3.72	0.1342	0.0011	0.0530
		Biological process	GO:0044262	cellular carbohydrate metabolic process	18	4.3	0.0001	408	260	17375	2.95	0.5216	0.0045	0.2710
		Biological process	GO:0044723	single-organism carbohydrate metabolic process	29	6.9	0.0004	408	594	17375	2.08	0.8612	0.0102	0.7241
		Biological process	GO:0016052	carbohydrate catabolic process	11	2.6	0.0009	408	129	17375	3.63	0.9916	0.0216	1.7456
		Biological process	GO:0043471	regulation of cellular carbohydrate catabolic process	4	1.0	0.0047	408	15	17375	11.36	1.0000	0.0767	8.6207
		Biological process	GO:0043255	regulation of carbohydrate biosynthetic process	8	1.9	0.0066	408	94	17375	3.62	1.0000	0.0998	11.8210
		Biological process	GO:0044275	cellular carbohydrate catabolic process	5	1.2	0.0071	408	33	17375	6.45	1.0000	0.1054	12.7197
		Biological process	GO:0010675	regulation of cellular carbohydrate metabolic process	10	2.4	0.0072	408	145	17375	2.94	1.0000	0.1065	12.8950
		Biological process	GO:0006109	regulation of carbohydrate metabolic process	11	2.6	0.0116	408	184	17375	2.55	1.0000	0.1497	20.0096
		Biological process	GO:0010906	regulation of glucose metabolic process	8	1.9	0.0178	408	114	17375	2.99	1.0000	0.1974	29.0440
		Biological process	GO:0006111	regulation of gluconeogenesis	5	1.2	0.0208	408	45	17375	4.73	1.0000	0.2202	33.0263
		Biological process	GO:0043470	regulation of carbohydrate catabolic process	5	1.2	0.0257	408	48	17375	4.44	1.0000	0.2535	39.1713
		Biological process	GO:0043467	regulation of generation of precursor metabolites and energy	7	1.7	0.0265	408	97	17375	3.07	1.0000	0.2577	40.0824
Cluster 29	3.41	Biological process	GO:0008219	cell death	78	18.6	0.0000	408	1903	17375	1.75	0.0055	0.0001	0.0020
		Biological process	GO:0010941	regulation of cell death	66	15.8	0.0000	408	1590	17375	1.77	0.0318	0.0003	0.0119
		Biological process	GO:0012501	programmed cell death	71	16.9	0.0000	408	1761	17375	1.72	0.0344	0.0003	0.0129
		Biological process	GO:0006915	apoptotic process	66	15.8	0.0000	408	1665	17375	1.69	0.1308	0.0011	0.0516
		Biological process	GO:0043067	regulation of programmed cell death	58	13.8	0.0001	408	1460	17375	1.69	0.3731	0.0031	0.1717
		Biological process	GO:0042981	regulation of apoptotic process	57	13.6	0.0001	408	1443	17375	1.68	0.4693	0.0040	0.2328
		Biological process	GO:0060548	negative regulation of cell death	38	9.1	0.0033	408	988	17375	1.64	1.0000	0.0568	6.1091
		Biological process	GO:0010942	positive regulation of cell death	27	6.4	0.0058	408	650	17375	1.77	1.0000	0.0897	10.4423
		Biological process	GO:0043069	negative regulation of programmed cell death	34	8.1	0.0066	408	893	17375	1.62	1.0000	0.1004	11.9232
		Biological process	GO:0043066	negative regulation of apoptotic process	33	7.9	0.0091	408	879	17375	1.60	1.0000	0.1249	15.9950
		Biological process	GO:0043065	positive regulation of apoptotic process	23	5.5	0.0261	408	600	17375	1.63	1.0000	0.2562	39.6739
		Biological process	GO:0043068	positive regulation of programmed cell death	23	5.5	0.0285	408	606	17375	1.62	1.0000	0.2690	42.4326
Cluster 30	3.26	Molecular function	GO:0044822	poly(A) RNA binding	57	13.6	0.0000	398	1219	16438	1.93	0.0026	0.0001	0.0037
		Molecular function	GO:0003723	RNA binding	66	15.8	0.0001	398	1673	16438	1.63	0.0806	0.0019	0.1205
		Molecular function	GO:0003676	nucleic acid binding	76	18.1	0.9782	398	3764	16438	0.83	1.0000	1.0000	100.0000
Cluster 31	3.11	Biological process	GO:0033036	macromolecule localization	91	21.7	0.0000	408	2573	17375	1.51	0.2051	0.0017	0.0844
		Biological process	GO:0008104	protein localization	81	19.3	0.0001	408	2255	17375	1.53	0.3519	0.0030	0.1595
		Biological process	GO:0045184	establishment of protein localization	67	16.0	0.0001	408	1770	17375	1.61	0.3867	0.0032	0.1798
		Biological process	GO:0015031	protein transport	62	14.8	0.0001	408	1602	17375	1.65	0.4082	0.0034	0.1929
		Biological process	GO:0033365	protein localization to organelle	37	8.8	0.0001	408	791	17375	1.99	0.4453	0.0037	0.2166
		Biological process	GO:0034613	cellular protein localization	58	13.8	0.0002	408	1505	17375	1.64	0.6378	0.0058	0.3730
		Biological process	GO:0070727	cellular macromolecule localization	58	13.8	0.0002	408	1517	17375	1.63	0.7201	0.0070	0.4674
		Biological process	GO:0070201	regulation of establishment of protein localization	37	8.8	0.0003	408	834	17375	1.89	0.8043	0.0087	0.5984
		Biological process	GO:0046907	intracellular transport	50	11.9	0.0007	408	1303	17375	1.63	0.9704	0.0166	1.2866
		Biological process	GO:0072594	establishment of protein localization to organelle	26	6.2	0.0011	408	543	17375	2.04	0.9961	0.0241	2.0191
		Biological process	GO:0032880	regulation of protein localization	40	9.5	0.0012	408	995	17375	1.71	0.9976	0.0256	2.1885
		Biological process	GO:1903827	regulation of cellular protein localization	26	6.2	0.0019	408	567	17375	1.95	1.0000	0.0367	3.6245
		Biological process	GO:0006886	intracellular protein transport	36	8.6	0.0024	408	901	17375	1.70	1.0000	0.0439	4.4395
		Biological process	GO:0060341	regulation of cellular localization	35	8.4	0.0026	408	873	17375	1.71	1.0000	0.0477	4.8903
		Biological process	GO:0051641	cellular localization	75	17.9	0.0033	408	2323	17375	1.37	1.0000	0.0570	6.1528
		Biological process	GO:0051649	establishment of localization in cell	57	13.6	0.0074	408	1724	17375	1.41	1.0000	0.1083	13.2157
		Biological process	GO:0006605	protein targeting	24	5.7	0.0159	408	605	17375	1.69	1.0000	0.1827	26.3494
		Biological process	GO:1902580	single-organism cellular localization	32	7.6	0.0527	408	975	17375	1.40	1.0000	0.3963	64.4564
Cluster 32	3.02	Biological process	GO:0006979	response to oxidative stress	35	8.4	0.0000	408	479	17375	3.11	0.0001	0.0000	0.0000
		Biological process	GO:0000302	response to reactive oxygen species	23	5.5	0.0000	408	265	17375	3.70	0.0016	0.0000	0.0006
		Biological process	GO:0034599	cellular response to oxidative stress	21	5.0	0.0000	408	281	17375	3.18	0.0563	0.0005	0.0213
		Biological process	GO:0042542	response to hydrogen peroxide	16	3.8	0.0000	408	175	17375	3.89	0.0814	0.0007	0.0312
		Biological process	GO:0034614	cellular response to reactive oxygen species	14	3.3	0.0001	408	165	17375	3.61	0.5180	0.0045	0.2682
		Biological process	GO:1903206	negative regulation of hydrogen peroxide-induced cell death	6	1.4	0.0002	408	23	17375	11.11	0.5720	0.0051	0.3118
		Biological process	GO:1901032	negative regulation of response to reactive oxygen species	6	1.4	0.0002	408	23	17375	11.11	0.5720	0.0051	0.3118
		Biological process	GO:0036473	cell death in response to oxidative stress	10	2.4	0.0002	408	86	17375	4.95	0.6110	0.0055	0.3469
		Biological process	GO:1900407	regulation of cellular response to oxidative stress	9	2.1	0.0006	408	80	17375	4.79	0.9462	0.0142	1.0699
		Biological process	GO:1902883	negative regulation of response to oxidative stress	7	1.7	0.0009	408	48	17375	6.21	0.9879	0.0203	1.6123
		Biological process	GO:1900408	negative regulation of cellular response to oxidative stress	7	1.7	0.0009	408	48	17375	6.21	0.9879	0.0203	1.6123
		Biological process	GO:1902882	regulation of response to oxidative stress	9	2.1	0.0009	408	86	17375	4.46	0.9913	0.0215	1.7289
		Biological process	GO:1903205	regulation of hydrogen peroxide-induced cell death	6	1.4	0.0010	408	33	17375	7.74	0.9928	0.0221	1.8013
		Biological process	GO:0036474	cell death in response to hydrogen peroxide	6	1.4	0.0010	408	33	17375	7.74	0.9928	0.0221	1.8013
		Biological process	GO:0070301	cellular response to hydrogen peroxide	10	2.4	0.0010	408	108	17375	3.94	0.9940	0.0225	1.8664
		Biological process	GO:1903201	regulation of oxidative stress-induced cell death	8	1.9	0.0012	408	70	17375	4.87	0.9984	0.0271	2.3388
		Biological process	GO:1901031	regulation of response to reactive oxygen species	6	1.4	0.0039	408	45	17375	5.68	1.0000	0.0657	7.1800
		Biological process	GO:1903202	negative regulation of oxidative stress-induced cell death	6	1.4	0.0047	408	47	17375	5.44	1.0000	0.0769	8.6151
		Biological process	GO:0008631	intrinsic apoptotic signaling pathway in response to oxidative stress	5	1.2	0.0139	408	40	17375	5.32	1.0000	0.1689	23.5338
		Biological process	GO:1901299	negative regulation of hydrogen peroxide-mediated programmed cell death	3	0.7	0.0140	408	8	17375	15.97	1.0000	0.1687	23.5528
		Biological process	GO:1902175	regulation of oxidative stress-induced intrinsic apoptotic signaling pathway	4	1.0	0.0246	408	27	17375	6.31	1.0000	0.2456	37.8778
		Biological process	GO:1901298	regulation of hydrogen peroxide-mediated programmed cell death	3	0.7	0.0414	408	14	17375	9.13	1.0000	0.3441	55.3829
		Biological process	GO:0010421	hydrogen peroxide-mediated programmed cell death	3	0.7	0.0529	408	16	17375	7.98	1.0000	0.3964	64.5994

		Biological process	GO:0097468	programmed cell death in response to reactive oxygen species	3	0.7	0.0529	408	16	17375	7.98	1.0000	0.3964	64.5994
		Biological process	GO:0097193	intrinsic apoptotic signaling pathway	10	2.4	0.2033	408	277	17375	1.54	1.0000	0.7637	98.7000
		Biological process	GO:2001242	regulation of intrinsic apoptotic signaling pathway	4	1.0	0.7073	408	155	17375	1.10	1.0000	0.9936	100.0000
Cluster 33	2.88	Biological process	GO:0045454	cell redox homeostasis	10	2.4	0.0000	408	64	17375	6.65	0.0851	0.0008	0.0327
		Molecular function	GO:0016667	oxidoreductase activity, acting on a sulfur group of donors	7	1.7	0.0014	398	51	16438	5.67	0.7847	0.0261	2.1789
		Biological process	GO:0002931	response to ischemia	4	1.0	0.0974	408	47	17375	3.62	1.0000	0.5604	85.8707
Cluster 34	2.83	Biological process	GO:0016485	protein processing	15	3.6	0.0011	408	230	17375	2.78	0.9966	0.0245	2.0678
		Biological process	GO:0051604	protein maturation	16	3.8	0.0015	408	264	17375	2.58	0.9995	0.0302	2.7603
		Biological process	GO:0031638	zymogen activation	6	1.4	0.0021	408	39	17375	6.55	1.0000	0.0387	3.8570
Cluster 35	2.75	Biological process	GO:1901566	organonitrogen compound biosynthetic process	64	15.3	0.0000	408	1412	17375	1.93	0.0026	0.0000	0.0009
		Biological process	GO:0043603	cellular amide metabolic process	53	12.6	0.0000	408	1111	17375	2.03	0.0072	0.0001	0.0027
		Biological process	GO:0006518	peptide metabolic process	46	11.0	0.0000	408	956	17375	2.05	0.0336	0.0003	0.0126
		Biological process	GO:0006413	translational initiation	9	2.1	0.0043	408	110	17375	3.48	1.0000	0.0723	7.9794
		Biological process	GO:0034248	regulation of cellular amide metabolic process	18	4.3	0.0049	408	359	17375	2.14	1.0000	0.0799	9.0028
		Biological process	GO:0006417	regulation of translation	16	3.8	0.0123	408	333	17375	2.05	1.0000	0.1556	21.0083
		Biological process	GO:0010608	posttranscriptional regulation of gene expression	17	4.1	0.0244	408	395	17375	1.83	1.0000	0.2445	37.6588
		Biological process	GO:0006412	translation	24	5.7	0.1868	408	804	17375	1.27	1.0000	0.7410	98.0780
		Biological process	GO:0043043	peptide biosynthetic process	24	5.7	0.2273	408	824	17375	1.24	1.0000	0.7949	99.2750
		Biological process	GO:0043604	amide biosynthetic process	25	6.0	0.2718	408	893	17375	1.19	1.0000	0.8393	99.7667
Cluster 36	2.62	Cellular component	GO:0045239	tricarboxylic acid cycle enzyme complex	5	1.2	0.0006	397	14	13535	12.18	0.2927	0.0115	0.8428
		Cellular component	GO:0045240	dihydrolipoyl dehydrogenase complex	4	1.0	0.0012	397	8	13535	17.05	0.5301	0.0226	1.8287
		Cellular component	GO:1990204	oxidoreductase complex	11	2.6	0.0021	397	116	13535	3.23	0.7241	0.0372	3.0984
		Biological process	GO:0006103	2-oxoglutarate metabolic process	4	1.0	0.0068	408	17	17375	10.02	1.0000	0.1023	12.2147
		Cellular component	GO:0045252	oxoglutarate dehydrogenase complex	3	0.7	0.0081	397	5	13535	20.46	0.9925	0.1125	11.2678
Cluster 37	2.45	Molecular function	GO:0051082	unfolded protein binding	12	2.9	0.0000	398	76	16438	6.52	0.0022	0.0001	0.0032
		Biological process	GO:0006457	protein folding	17	4.1	0.0000	408	173	17375	4.18	0.0166	0.0002	0.0062
		Biological process	GO:1903405	protein localization to nuclear body	5	1.2	0.0000	408	8	17375	26.62	0.0952	0.0008	0.0368
		Biological process	GO:1904867	protein localization to Cajal body	5	1.2	0.0000	408	8	17375	26.62	0.0952	0.0008	0.0368
		Biological process	GO:1904871	positive regulation of protein localization to Cajal body	5	1.2	0.0000	408	8	17375	26.62	0.0952	0.0008	0.0368
		Biological process	GO:1904869	regulation of protein localization to Cajal body	5	1.2	0.0000	408	8	17375	26.62	0.0952	0.0008	0.0368
		Biological process	GO:1990173	protein localization to nucleoplasm	5	1.2	0.0000	408	8	17375	26.62	0.0952	0.0008	0.0368
		Cellular component	GO:0005832	chaperonin-containing T-complex	5	1.2	0.0001	397	9	13535	18.94	0.0478	0.0021	0.1196
		Biological process	GO:1904872	regulation of telomerase RNA localization to Cajal body	5	1.2	0.0003	408	15	17375	14.20	0.8197	0.0091	0.6284
		Biological process	GO:1904874	positive regulation of telomerase RNA localization to Cajal body	5	1.2	0.0003	408	15	17375	14.20	0.8197	0.0091	0.6284
		Biological process	GO:0090670	RNA localization to Cajal body	5	1.2	0.0004	408	16	17375	13.31	0.8938	0.0112	0.8217
		Biological process	GO:0090671	telomerase RNA localization to Cajal body	5	1.2	0.0004	408	16	17375	13.31	0.8938	0.0112	0.8217
		Biological process	GO:0090672	telomerase RNA localization	5	1.2	0.0004	408	16	17375	13.31	0.8938	0.0112	0.8217
		Cellular component	GO:0002199	zona pellucida receptor complex	5	1.2	0.0006	397	14	13535	12.18	0.2927	0.0115	0.8428
		Biological process	GO:2000573	positive regulation of DNA biosynthetic process	8	1.9	0.0007	408	64	17375	5.32	0.9766	0.0176	1.3727
		Biological process	GO:0032206	positive regulation of telomere maintenance	7	1.7	0.0009	408	48	17375	6.21	0.9879	0.0203	1.6123
		Biological process	GO:0032212	positive regulation of telomere maintenance via telomerase	6	1.4	0.0010	408	33	17375	7.74	0.9928	0.0221	1.8013
		Biological process	GO:1904851	positive regulation of establishment of protein localization to telomere	4	1.0	0.0010	408	9	17375	18.93	0.9933	0.0222	1.8273
		Biological process	GO:1904358	positive regulation of telomere maintenance via telomere lengthening	6	1.4	0.0011	408	34	17375	7.52	0.9966	0.0244	2.0691
		Biological process	GO:0032204	regulation of telomere maintenance	8	1.9	0.0011	408	69	17375	4.94	0.9973	0.0252	2.1493
		Biological process	GO:1904356	regulation of telomere maintenance via telomere lengthening	7	1.7	0.0012	408	51	17375	5.85	0.9978	0.0259	2.2242
		Biological process	GO:0070203	regulation of establishment of protein localization to telomere	4	1.0	0.0014	408	10	17375	17.03	0.9991	0.0287	2.5563
		Biological process	GO:0070202	regulation of establishment of protein localization to chromosome	4	1.0	0.0018	408	11	17375	15.49	0.9999	0.0350	3.4393
		Biological process	GO:1901998	toxin transport	6	1.4	0.0023	408	40	17375	6.39	1.0000	0.0428	4.3149
		Biological process	GO:1904816	positive regulation of protein localization to chromosome, telomeric region	4	1.0	0.0024	408	12	17375	14.20	1.0000	0.0442	4.4834
		Biological process	GO:0010833	telomere maintenance via telomere lengthening	7	1.7	0.0027	408	60	17375	4.97	1.0000	0.0495	5.1115
		Biological process	GO:0090666	scaRNA localization to Cajal body	3	0.7	0.0032	408	4	17375	31.94	1.0000	0.0557	5.9096
		Biological process	GO:1904814	regulation of protein localization to chromosome, telomeric region	4	1.0	0.0038	408	14	17375	12.17	1.0000	0.0651	7.0726
		Biological process	GO:0032210	regulation of telomere maintenance via telomerase	6	1.4	0.0039	408	45	17375	5.68	1.0000	0.0657	7.1800
		Biological process	GO:0051054	positive regulation of DNA metabolic process	13	3.1	0.0044	408	212	17375	2.61	1.0000	0.0725	8.0234
		Biological process	GO:0070200	establishment of protein localization to telomere	4	1.0	0.0047	408	15	17375	11.36	1.0000	0.0767	8.6207
		Biological process	GO:0034504	protein localization to nucleus	18	4.3	0.0060	408	367	17375	2.09	1.0000	0.0925	10.8995
		Biological process	GO:0007004	telomere maintenance via telomerase	6	1.4	0.0072	408	52	17375	4.91	1.0000	0.1066	12.9767
		Biological process	GO:0006278	RNA-dependent DNA biosynthetic process	6	1.4	0.0079	408	53	17375	4.82	1.0000	0.1119	13.9841
		Biological process	GO:0000723	telomere maintenance	8	1.9	0.0096	408	101	17375	3.37	1.0000	0.1303	16.8393
		Biological process	GO:2000278	regulation of DNA biosynthetic process	8	1.9	0.0096	408	101	17375	3.37	1.0000	0.1303	16.8393
		Biological process	GO:0032200	telomere organization	8	1.9	0.0096	408	101	17375	3.37	1.0000	0.1303	16.8393
		Biological process	GO:0007339	binding of sperm to zona pellucida	5	1.2	0.0117	408	38	17375	5.60	1.0000	0.1501	20.1087
		Biological process	GO:0070199	establishment of protein localization to chromosome	4	1.0	0.0124	408	21	17375	8.11	1.0000	0.1564	21.2165
		Biological process	GO:1900182	positive regulation of protein localization to nucleus	9	2.1	0.0126	408	132	17375	2.90	1.0000	0.1568	21.4707
		Biological process	GO:0035036	sperm-egg recognition	5	1.2	0.0152	408	41	17375	5.19	1.0000	0.1784	25.3344
		Biological process	GO:0007338	single fertilization	8	1.9	0.0178	408	114	17375	2.99	1.0000	0.1974	29.0440
		Biological process	GO:0006403	RNA localization	9	2.1	0.0210	408	145	17375	2.64	1.0000	0.2219	33.3264
		Biological process	GO:0070198	protein localization to chromosome, telomeric region	4	1.0	0.0246	408	27	17375	6.31	1.0000	0.2456	37.8778
		Biological process	GO:0071897	DNA biosynthetic process	9	2.1	0.0269	408	152	17375	2.52	1.0000	0.2605	40.6023
		Biological process	GO:0051973	positive regulation of telomerase activity	4	1.0	0.0297	408	29	17375	5.87	1.0000	0.2764	43.7999
		Biological process	GO:2001252	positive regulation of chromosome organization	9	2.1	0.0307	408	156	17375	2.46	1.0000	0.2836	44.9313
		Biological process	GO:0009566	fertilization	9	2.1	0.0328	408	158	17375	2.43	1.0000	0.2967	47.1229
		Biological process	GO:0050821	protein stabilization	8	1.9	0.0334	408	130	17375	2.62	1.0000	0.2995	47.7830
		Biological process	GO:0008037	cell recognition	8	1.9	0.0359	408	132	17375	2.58	1.0000	0.3152	50.2484
		Biological process	GO:1900180	regulation of protein localization to nucleus	11	2.6	0.0387	408	224	17375	2.09	1.0000	0.3300	52.9782
		Biological process	GO:0009988	cell-cell recognition	5	1.2	0.0397	408	55	17375	3.87	1.0000	0.3352	53.8692
		Biological process	GO:0032846	positive regulation of homeostatic process	11	2.6	0.0543	408	238	17375	1.97	1.0000	0.4023	65.5755
		Biological process	GO:0051052	regulation of DNA metabolic process	15	3.6	0.0582	408	374	17375	1.71	1.0000	0.4201	68.1760
		Biological process	GO:0060249	anatomical structure homeostasis	14	3.3	0.0606	408	343	17375	1.74	1.0000	0.4295	69.6950
		Biological process	GO:0032844	regulation of homeostatic process	18	4.3	0.0685	408	488	17375	1.57	1.0000	0.4651	74.2383
		Biological process	GO:0031647	regulation of protein stability	10	2.4	0.0799	408	223	17375	1.91	1.0000	0.5055	79.6313
		Biological process	GO:0051972	regulation of telomerase activity	4	1.0	0.0927	408	46	17375	3.70	1.0000	0.5462	84.4021
		Biological process	GO:0033044	regulation of chromosome organization	11	2.6	0.1566	408	295	17375	1.59	1.0000	0.6934	96.1374
		Biological process	GO:0034502	protein localization to chromosome	4	1.0	0.1658	408	60	17375	2.84	1.0000	0.7069	96.8706
		Biological process	GO:0006259	DNA metabolic process	24	5.7	0.2343	408	832	17375	1.23	1.0000	0.8013	99.3913
		Biological process	GO:0045935	positive regulation of nucleobase-containing compound metabolic process	33	7.9	0.8968	408	1657	17375	0.85	1.0000	0.9997	100.0000
Cluster 38	2.45	Biological process	GO:0006578	amino-acid betaine biosynthetic process	4	1.0	0.0002	408	6	17375	28.39	0.7155	0.0070	0.4616
		Biological process	GO:0045329	carnitine biosynthetic process	3	0.7	0.0032	408	4	17375	31.94	1.0000	0.0557	5.9096
		Biological process	GO:0006577	amino-acid betaine metabolic process	4	1.0	0.0068	408	17	17375	10.02	1.0000	0.1023	12.2147
		Biological process	GO:0009437	carnitine metabolic process	3	0.7	0.0309	408	12	17375	10.65	1.0000	0.2847	45.1371
Cluster 39	2.43	Biological process	GO:0051084	'de novo' posttranslational protein folding	5	1.2	0.0011	408	20	17375	10.65	0.9961	0.0240	2.0195
		Biological process	GO:0006458	'de novo' protein folding	5	1.2	0.0011	408	20	17375	10.65	0.9961	0.0240	2.0195
		Biological process	GO:0061077	chaperone-mediated protein folding	6	1.4	0.0047	408	47	17375	5.44	1.0000	0.0769	8.6151
		Biological process	GO:0051085	chaperone mediated protein folding requiring cofactor	3	0.7	0.0360	408	13	17375	9.83	1.0000	0.3156	50.3661
Cluster 40	2.39	Biological process	GO:0006541	glutamine metabolic process	6	1.4	0.0003	408	26	17375	9.83	0.7911	0.0085	0.5745
		Biological process	GO:0009064	glutamine family amino acid metabolic process	8	1.9	0.0015	408	72	17375	4.73	0.9995	0.0303	2.7561
		Biological process	GO:0006536	glutamate metabolic process	3	0.7	0.1554	408	30	17375	4.26	1.0000	0.6913	96.0320
Cluster 41	2.34	Biological process	GO:0006144	purine nucleobase metabolic process	5	1.2	0.0039	408	28	17375	7.60	1.0000	0.0659	7.1786
		Biological process	GO:0009112	nucleobase metabolic process	6	1.4	0.0043	408	46	17375	5.55	1.0000	0.0716	7.8760
		Biological process	GO:0046033	AMP metabolic process	4	1.0	0.0057	408	16	17375	10.65	1.0000	0.0890	10.3360
Cluster 42	2.34	Molecular function	GO:0032561	guanyl ribonucleotide binding	21	5.0	0.0019	398	402	16438	2.16	0.8824	0.0319	3.0236
		Molecular function	GO:0019001	guanyl nucleotide binding	21	5.0	0.0020	398	403	16438	2.15	0.8895	0.0323	3.1102
		Molecular function	GO:0005525	GTP binding	20	4.8	0.0023	398	380	16438	2.17	0.9256	0.0369	3.6576
		Molecular function	GO:0003924	GTPase activity	10	2.4	0.0514	398	198	16438	2.09	1.0000	0.3856	57.0650
Cluster 43	2.33	Biological process	GO:0006739	NADP metabolic process	7	1.7	0.0002	408	36	17375	8.28	0.5884	0.0052	0.3261
		Biological process	GO:0051156	glucose 6-phosphate metabolic process	5	1.2	0.0016	408	22	17375	9.68	0.9997	0.0314	2.9255
		Biological process	GO:0009051	pentose-phosphate shunt, oxidative branch	3	0.7	0.0016	408	3	17375	42.59	0.9998	0.0318	3.0440
		Biological process	GO:0006098	pentose-phosphate shunt	4	1.0	0.0038	408	14	17375	12.17	1.0000	0.0651	7.0726
		Biological process	GO:0019682	glyceraldehyde-3-phosphate metabolic process	4	1.0	0.0038	408	14	17375	12.17	1.0000	0.0651	7.0726
		Molecular function	GO:0048029	monosaccharide binding	5	1.2	0.0627	398	62	16438	3.33	1.0000	0.4289	64.5358
		Molecular function	GO:0030246	carbohydrate binding	10	2.4	0.1212	398	237	16438	1.74	1.0000	0.6083	87.3862
Cluster 44	2.32	Cellular component	GO:0044429	mitochondrial part	49	11.7	0.0000	397	820	13535	2.04	0.0019	0.0001	0.0047

		Cellular component	GO:0005740	mitochondrial envelope	32	7.6	0.0032	397	629	13535	1.73	0.8548	0.0536	4.6059
		Cellular component	GO:0031967	organelle envelope	44	10.5	0.0088	397	1014	13535	1.48	0.9952	0.1193	12.2280
		Cellular component	GO:0031975	envelope	44	10.5	0.0099	397	1022	13535	1.47	0.9976	0.1278	13.6725
		Cellular component	GO:0031966	mitochondrial membrane	27	6.4	0.0222	397	584	13535	1.58	1.0000	0.2193	28.3006
		Cellular component	GO:0005743	mitochondrial inner membrane	20	4.8	0.0343	397	412	13535	1.66	1.0000	0.2813	40.3475
		Cellular component	GO:0019866	organelle inner membrane	20	4.8	0.0844	397	461	13535	1.48	1.0000	0.4740	72.8343
Cluster 45	2.32	Biological process	GO:0016042	lipid catabolic process	21	5.0	0.0000	408	275	17375	3.25	0.0415	0.0004	0.0156
		Biological process	GO:0044242	cellular lipid catabolic process	16	3.8	0.0000	408	183	17375	3.72	0.1342	0.0011	0.0530
		Biological process	GO:0072329	monocarboxylic acid catabolic process	11	2.6	0.0001	408	96	17375	4.88	0.3520	0.0029	0.1596
		Biological process	GO:0009062	fatty acid catabolic process	10	2.4	0.0002	408	85	17375	5.01	0.5781	0.0051	0.3171
		Molecular function	GO:0016627	oxidoreductase activity, acting on the CH-CH group of donors	8	1.9	0.0005	398	58	16438	5.70	0.4105	0.0099	0.7552
		Molecular function	GO:0050660	flavin adenine dinucleotide binding	8	1.9	0.0045	398	85	16438	3.89	0.9932	0.0612	6.9106
		Biological process	GO:0006635	fatty acid beta-oxidation	7	1.7	0.0055	408	69	17375	4.32	1.0000	0.0868	10.0183
		Molecular function	GO:0000062	fatty-acyl-CoA binding	5	1.2	0.0129	398	38	16438	5.43	1.0000	0.1393	18.8400
		Molecular function	GO:0009055	electron carrier activity	7	1.7	0.0207	398	89	16438	3.25	1.0000	0.2031	28.4968
		Biological process	GO:0019395	fatty acid oxidation	7	1.7	0.0265	408	97	17375	3.07	1.0000	0.2577	40.0824
		Biological process	GO:0034440	lipid oxidation	7	1.7	0.0289	408	99	17375	3.01	1.0000	0.2716	42.8606
		Biological process	GO:0030258	lipid modification	10	2.4	0.0418	408	196	17375	2.17	1.0000	0.3448	55.7498
		Molecular function	GO:0052890	oxidoreductase activity, acting on the CH-CH group of donors, with a flavin as acceptor	3	0.7	0.0623	398	17	16438	7.29	1.0000	0.4297	64.3353
		Molecular function	GO:0003995	acyl-CoA dehydrogenase activity	3	0.7	0.0623	398	17	16438	7.29	1.0000	0.4297	64.3353
		Biological process	GO:0033539	fatty acid beta-oxidation using acyl-CoA dehydrogenase	3	0.7	0.0788	408	20	17375	6.39	1.0000	0.5023	79.1686
		Biological process	GO:0055088	lipid homeostasis	3	0.7	0.8017	408	127	17375	1.01	1.0000	0.9982	100.0000
Cluster 46	2.31	Biological process	GO:0009894	regulation of catabolic process	28	6.7	0.0000	408	465	17375	2.56	0.0749	0.0007	0.0286
		Biological process	GO:0031329	regulation of cellular catabolic process	22	5.3	0.0000	408	317	17375	2.96	0.0988	0.0009	0.0383
		Biological process	GO:0042176	regulation of protein catabolic process	20	4.8	0.0002	408	317	17375	2.69	0.6168	0.0056	0.3523
		Biological process	GO:1903362	regulation of cellular protein catabolic process	14	3.3	0.0004	408	184	17375	3.24	0.8807	0.0107	0.7794
		Biological process	GO:1903050	regulation of proteolysis involved in cellular protein catabolic process	12	2.9	0.0017	408	164	17375	3.12	0.9999	0.0332	3.2039
		Biological process	GO:0061136	regulation of proteasomal protein catabolic process	11	2.6	0.0018	408	141	17375	3.32	0.9999	0.0348	3.3971
		Biological process	GO:0034976	response to endoplasmic reticulum stress	13	3.1	0.0054	408	218	17375	2.54	1.0000	0.0860	9.8984
		Biological process	GO:0045732	positive regulation of protein catabolic process	11	2.6	0.0116	408	184	17375	2.55	1.0000	0.1497	20.0096
		Biological process	GO:0036503	ERAD pathway	7	1.7	0.0147	408	85	17375	3.51	1.0000	0.1758	24.7095
		Biological process	GO:1903364	positive regulation of cellular protein catabolic process	8	1.9	0.0163	408	112	17375	3.04	1.0000	0.1850	26.9430
		Biological process	GO:0030433	ER-associated ubiquitin-dependent protein catabolic process	6	1.4	0.0228	408	69	17375	3.70	1.0000	0.2350	35.5777
		Biological process	GO:1903052	positive regulation of proteolysis involved in cellular protein catabolic process	7	1.7	0.0276	408	98	17375	3.04	1.0000	0.2634	41.4671
		Biological process	GO:0032434	regulation of proteasomal ubiquitin-dependent protein catabolic process	7	1.7	0.0370	408	105	17375	2.84	1.0000	0.3206	51.3136
		Biological process	GO:0009896	positive regulation of catabolic process	12	2.9	0.0443	408	261	17375	1.96	1.0000	0.3577	57.8916
		Biological process	GO:0031331	positive regulation of cellular catabolic process	10	2.4	0.0452	408	199	17375	2.14	1.0000	0.3626	58.6597
		Biological process	GO:1901800	positive regulation of proteasomal protein catabolic process	6	1.4	0.0473	408	84	17375	3.04	1.0000	0.3732	60.3438
		Biological process	GO:0032436	positive regulation of proteasomal ubiquitin-dependent protein catabolic process	4	1.0	0.2194	408	69	17375	2.47	1.0000	0.7853	99.1205
Cluster 47	2.31	Biological process	GO:0010638	positive regulation of organelle organization	28	6.7	0.0013	408	612	17375	1.95	0.9988	0.0276	2.4317
		Biological process	GO:0051130	positive regulation of cellular component organization	46	11.0	0.0033	408	1264	17375	1.55	1.0000	0.0565	6.0426
		Biological process	GO:0033043	regulation of organelle organization	41	9.8	0.0078	408	1148	17375	1.52	1.0000	0.1111	13.8172
		Biological process	GO:0051128	regulation of cellular component organization	72	17.2	0.0175	408	2376	17375	1.29	1.0000	0.1961	28.6936
Cluster 48	2.31	Biological process	GO:0051259	protein oligomerization	25	6.0	0.0016	408	527	17375	2.02	0.9997	0.0313	2.9443
		Biological process	GO:0051262	protein tetramerization	11	2.6	0.0033	408	153	17375	3.06	1.0000	0.0568	6.0889
		Biological process	GO:0051289	protein homotetramerization	7	1.7	0.0099	408	78	17375	3.82	1.0000	0.1335	17.3338
		Biological process	GO:0051260	protein homooligomerization	15	3.6	0.0118	408	301	17375	2.12	1.0000	0.1509	20.3008
Cluster 49	2.23	Biological process	GO:0009066	aspartate family amino acid metabolic process	8	1.9	0.0001	408	48	17375	7.10	0.4585	0.0039	0.2255
		Biological process	GO:0008652	cellular amino acid biosynthetic process	8	1.9	0.0004	408	59	17375	5.77	0.8977	0.0113	0.8353
		Biological process	GO:0043094	cellular metabolic compound salvage	6	1.4	0.0005	408	29	17375	8.81	0.9306	0.0131	0.9771
		Biological process	GO:0006555	methionine metabolic process	4	1.0	0.0080	408	18	17375	9.46	1.0000	0.1137	14.2508
		Biological process	GO:0019509	L-methionine biosynthetic process from methylthioadenosine	3	0.7	0.0140	408	8	17375	15.97	1.0000	0.1687	23.5528
		Biological process	GO:0071267	L-methionine salvage	3	0.7	0.0218	408	10	17375	12.78	1.0000	0.2281	34.3048
		Biological process	GO:0043102	amino acid salvage	3	0.7	0.0218	408	10	17375	12.78	1.0000	0.2281	34.3048
		Biological process	GO:0009067	aspartate family amino acid biosynthetic process	4	1.0	0.0246	408	27	17375	6.31	1.0000	0.2456	37.8778
		Biological process	GO:0071265	L-methionine biosynthetic process	3	0.7	0.0262	408	11	17375	11.61	1.0000	0.2559	39.7578
		Biological process	GO:0009086	methionine biosynthetic process	3	0.7	0.0529	408	16	17375	7.98	1.0000	0.3964	64.5994
Cluster 50	2.14	Molecular function	GO:0016667	oxidoreductase activity, acting on a sulfur group of donors	7	1.7	0.0014	398	51	16438	5.67	0.7847	0.0261	2.1789
		Molecular function	GO:0015038	glutathione disulfide oxidoreductase activity	3	0.7	0.0055	398	5	16438	24.78	0.9980	0.0747	8.5222
		Molecular function	GO:0015037	peptide disulfide oxidoreductase activity	3	0.7	0.0113	398	7	16438	17.70	1.0000	0.1286	16.6126
		Molecular function	GO:0015036	disulfide oxidoreductase activity	4	1.0	0.0321	398	29	16438	5.70	1.0000	0.2865	40.7221
Cluster 51	2.09	Biological process	GO:0008104	protein localization	81	19.3	0.0001	408	2255	17375	1.53	0.3519	0.0030	0.1595
		Biological process	GO:0045184	establishment of protein localization	67	16.0	0.0001	408	1770	17375	1.61	0.3867	0.0032	0.1798
		Biological process	GO:0033365	protein localization to organelle	37	8.8	0.0001	408	791	17375	1.99	0.4453	0.0037	0.2166
		Biological process	GO:0070201	regulation of establishment of protein localization	37	8.8	0.0003	408	834	17375	1.89	0.8043	0.0087	0.5984
		Biological process	GO:1903829	positive regulation of cellular protein localization	20	4.8	0.0009	408	360	17375	2.37	0.9900	0.0210	1.6788
		Biological process	GO:0032880	regulation of protein localization	40	9.5	0.0012	408	995	17375	1.71	0.9976	0.0256	2.1885
		Biological process	GO:1903827	regulation of cellular protein localization	26	6.2	0.0019	408	567	17375	1.95	1.0000	0.0367	3.6245
		Biological process	GO:0060341	regulation of cellular localization	35	8.4	0.0026	408	873	17375	1.71	1.0000	0.0477	4.8903
		Biological process	GO:1904951	positive regulation of establishment of protein localization	24	5.7	0.0035	408	530	17375	1.93	1.0000	0.0595	6.4469
		Biological process	GO:0032386	regulation of intracellular transport	22	5.3	0.0050	408	483	17375	1.94	1.0000	0.0812	9.2074
		Biological process	GO:0051223	regulation of protein transport	31	7.4	0.0053	408	778	17375	1.70	1.0000	0.0846	9.6760
		Biological process	GO:0051222	positive regulation of protein transport	19	4.5	0.0442	408	494	17375	1.64	1.0000	0.3579	57.8488
		Biological process	GO:0032388	positive regulation of intracellular transport	13	3.1	0.0500	408	299	17375	1.85	1.0000	0.3862	62.4883
		Biological process	GO:0051049	regulation of transport	55	13.1	0.0572	408	1860	17375	1.26	1.0000	0.4168	67.5395
		Biological process	GO:1903533	regulation of protein targeting	13	3.1	0.0591	408	307	17375	1.80	1.0000	0.4239	68.8032
		Biological process	GO:0033157	regulation of intracellular protein transport	15	3.6	0.0660	408	381	17375	1.68	1.0000	0.4552	72.8854
		Biological process	GO:0050708	regulation of protein secretion	15	3.6	0.1114	408	416	17375	1.54	1.0000	0.5984	89.5252
		Biological process	GO:0051050	positive regulation of transport	31	7.4	0.1126	408	1014	17375	1.30	1.0000	0.6000	89.7972
		Biological process	GO:0090316	positive regulation of intracellular protein transport	10	2.4	0.1675	408	264	17375	1.61	1.0000	0.7084	96.9846
		Biological process	GO:0009306	protein secretion	16	3.8	0.1814	408	492	17375	1.38	1.0000	0.7325	97.8144
		Biological process	GO:1903530	regulation of secretion by cell	17	4.1	0.5972	408	709	17375	1.02	1.0000	0.9821	100.0000
Cluster 52	2.01	Molecular function	GO:0016462	pyrophosphatase activity	33	7.9	0.0013	398	749	16438	1.82	0.7655	0.0251	2.0586
		Molecular function	GO:0016818	hydrolase activity, acting on acid anhydrides, in phosphorus-containing anhydrides	33	7.9	0.0014	398	752	16438	1.81	0.7931	0.0263	2.2344
		Molecular function	GO:0016817	hydrolase activity, acting on acid anhydrides	33	7.9	0.0016	398	758	16438	1.80	0.8320	0.0284	2.5262
		Molecular function	GO:0017111	nucleoside-triphosphatase activity	27	6.4	0.0209	398	703	16438	1.59	1.0000	0.2032	28.7361
		Molecular function	GO:0016887	ATPase activity	16	3.8	0.1037	398	433	16438	1.53	1.0000	0.5621	82.6794
		Molecular function	GO:0042623	ATPase activity, coupled	12	2.9	0.1421	398	315	16438	1.57	1.0000	0.6571	91.4266

Table S17: B-III Male_Effect of FGR (only Poor)

Rank	Score	Category	Term ID	Term name	Count	%	PValue	List Total	Pop Hits	Pop Total	Fold Enrichment	Bonferroni	Benjamini	FDR
Cluster 1	14.85	Cellular component	GO:1903561	extracellular vesicle	89	43.8	0.0000	190	2661	13535	2.38	0.0000	0.0000	0.0000
		Cellular component	GO:0043230	extracellular organelle	89	43.8	0.0000	190	2666	13535	2.38	0.0000	0.0000	0.0000
		Cellular component	GO:0070062	extracellular exosome	88	43.3	0.0000	190	2646	13535	2.37	0.0000	0.0000	0.0000
		Cellular component	GO:0044421	extracellular region part	104	51.2	0.0000	190	3710	13535	2.00	0.0000	0.0000	0.0000
		Cellular component	GO:0031988	membrane-bounded vesicle	95	46.8	0.0000	190	3321	13535	2.04	0.0000	0.0000	0.0000
		Cellular component	GO:0005576	extracellular region	105	51.7	0.0000	190	4113	13535	1.82	0.0000	0.0000	0.0000
Cluster 2	4.05	Biological process	GO:0010035	response to inorganic substance	23	11.3	0.0000	192	677	17375	3.07	0.0205	0.0069	0.0102
		Biological process	GO:0010038	response to metal ion	17	8.4	0.0000	192	448	17375	3.43	0.1282	0.0136	0.0679
		Biological process	GO:0070482	response to oxygen levels	13	6.4	0.0034	192	438	17375	2.69	1.0000	0.1734	6.1192
Cluster 3	4.04	Biological process	GO:0006508	proteolysis	42	20.7	0.0000	192	1661	17375	2.29	0.0022	0.0022	0.0011
		Molecular function	GO:0004175	endopeptidase activity	16	7.9	0.0003	188	466	16438	3.00	0.1876	0.0292	0.4407
		Molecular function	GO:0070011	peptidase activity, acting on L-amino acid peptides	19	9.4	0.0005	188	651	16438	2.55	0.2812	0.0360	0.6993
		Molecular function	GO:0008233	peptidase activity	19	9.4	0.0009	188	688	16438	2.41	0.4658	0.0509	1.3238
Cluster 4	3.07	Biological process	GO:0032269	negative regulation of cellular protein metabolic process	29	14.3	0.0000	192	984	17375	2.67	0.0130	0.0065	0.0065
		Biological process	GO:0051248	negative regulation of protein metabolic process	29	14.3	0.0000	192	1043	17375	2.52	0.0384	0.0097	0.0194
		Biological process	GO:0051346	negative regulation of hydrolase activity	17	8.4	0.0000	192	429	17375	3.59	0.0784	0.0135	0.0404
		Biological process	GO:0010951	negative regulation of endopeptidase activity	13	6.4	0.0000	192	264	17375	4.46	0.1277	0.0151	0.0676
		Biological process	GO:0052548	regulation of endopeptidase activity	16	7.9	0.0001	192	411	17375	3.52	0.1738	0.0172	0.0944
		Biological process	GO:0010466	negative regulation of peptidase activity	13	6.4	0.0001	192	279	17375	4.22	0.2088	0.0179	0.1158
		Biological process	GO:0044092	negative regulation of molecular function	27	13.3	0.0001	192	1056	17375	2.31	0.2983	0.0219	0.1751
		Biological process	GO:0052547	regulation of peptidase activity	16	7.9	0.0001	192	436	17375	3.32	0.3078	0.0214	0.1819
		Biological process	GO:0032268	regulation of cellular protein metabolic process	46	22.7	0.0001	192	2350	17375	1.77	0.3445	0.0220	0.2088
		Biological process	GO:0043086	negative regulation of catalytic activity	22	10.8	0.0001	192	781	17375	2.55	0.4135	0.0251	0.2637
		Biological process	GO:0045861	negative regulation of proteolysis	14	6.9	0.0002	192	355	17375	3.57	0.4421	0.0262	0.2883
		Molecular function	GO:0004867	serine-type endopeptidase inhibitor activity	8	3.9	0.0002	188	105	16438	6.66	0.1302	0.0674	0.2960
		Biological process	GO:0051246	regulation of protein metabolic process	47	23.2	0.0003	192	2514	17375	1.69	0.6353	0.0354	0.4979
		Biological process	GO:0051336	regulation of hydrolase activity	26	12.8	0.0015	192	1198	17375	1.96	0.9961	0.1138	2.7117
		Biological process	GO:0030162	regulation of proteolysis	18	8.9	0.0017	192	693	17375	2.35	0.9983	0.1217	3.0963
		Molecular function	GO:0004866	endopeptidase inhibitor activity	9	4.4	0.0018	188	195	16438	4.04	0.7189	0.0930	2.6614
		Molecular function	GO:0061135	endopeptidase regulator activity	9	4.4	0.0022	188	202	16438	3.90	0.7937	0.0939	3.2992
		Molecular function	GO:0030414	peptidase inhibitor activity	9	4.4	0.0026	188	207	16438	3.80	0.8406	0.0921	3.8274
		Molecular function	GO:0061134	peptidase regulator activity	9	4.4	0.0057	188	236	16438	3.33	0.9827	0.1618	8.2655
		Biological process	GO:0050790	regulation of catalytic activity	36	17.7	0.0067	192	2082	17375	1.56	1.0000	0.2413	11.6901
		Biological process	GO:0031324	negative regulation of cellular metabolic process	39	19.2	0.0073	192	2323	17375	1.52	1.0000	0.2478	12.6522
		Biological process	GO:0065009	regulation of molecular function	42	20.7	0.0108	192	2616	17375	1.45	1.0000	0.3144	18.1170
		Biological process	GO:0009892	negative regulation of metabolic process	40	19.7	0.0122	192	2478	17375	1.46	1.0000	0.3343	20.1922
		Molecular function	GO:0004857	enzyme inhibitor activity	10	4.9	0.0310	188	382	16438	2.29	1.0000	0.4137	37.9136
		Biological process	GO:0010605	negative regulation of macromolecule metabolic process	34	16.7	0.0537	192	2263	17375	1.36	1.0000	0.6228	63.8694
		Molecular function	GO:0098772	molecular function regulator	18	8.9	0.1949	188	1188	16438	1.32	1.0000	0.8025	96.2202
		Molecular function	GO:0030234	enzyme regulator activity	13	6.4	0.3006	188	880	16438	1.29	1.0000	0.8819	99.5498
Cluster 5	2.76	Biological process	GO:0007586	digestion	9	4.4	0.0001	192	135	17375	6.03	0.3704	0.0229	0.2287
		Biological process	GO:0022600	digestive system process	6	3.0	0.0061	192	105	17375	5.17	1.0000	0.2341	10.6111
		Biological process	GO:0050892	intestinal absorption	4	2.0	0.0072	192	36	17375	10.05	1.0000	0.2454	12.3978
Cluster 6	2.65	Molecular function	GO:0042802	identical protein binding	31	15.3	0.0002	188	1325	16438	2.05	0.1412	0.0495	0.3231
		Molecular function	GO:0042803	protein homodimerization activity	19	9.4	0.0048	188	805	16438	2.06	0.9680	0.1512	7.0536
		Molecular function	GO:0046983	protein dimerization activity	25	12.3	0.0109	188	1283	16438	1.70	0.9996	0.2499	15.2140
Cluster 7	2.55	Biological process	GO:0051186	cofactor metabolic process	13	6.4	0.0009	192	373	17375	3.15	0.9638	0.0858	1.6286
		Biological process	GO:0051188	cofactor biosynthetic process	8	3.9	0.0014	192	150	17375	4.83	0.9938	0.1092	2.4853
		Biological process	GO:0006732	coenzyme metabolic process	10	4.9	0.0070	192	307	17375	2.95	1.0000	0.2432	12.1567
		Biological process	GO:0009108	coenzyme biosynthetic process	6	3.0	0.0074	192	110	17375	4.94	1.0000	0.2470	12.7319
Cluster 8	2.37	Biological process	GO:0031667	response to nutrient levels	17	8.4	0.0010	192	603	17375	2.55	0.9796	0.0973	1.9069
		Biological process	GO:0009991	response to extracellular stimulus	17	8.4	0.0016	192	630	17375	2.44	0.9978	0.1219	2.9789
		Biological process	GO:0007584	response to nutrient	11	5.4	0.0020	192	304	17375	3.27	0.9994	0.1312	3.6220
		Biological process	GO:0009605	response to external stimulus	33	16.3	0.0963	192	2306	17375	1.30	1.0000	0.7314	84.5406
Cluster 9	2.35	Biological process	GO:1901698	response to nitrogen compound	31	15.3	0.0000	192	1171	17375	2.40	0.0443	0.0090	0.0224
		Biological process	GO:0014070	response to organic cyclic compound	31	15.3	0.0001	192	1302	17375	2.15	0.2786	0.0231	0.1615
		Biological process	GO:0010033	response to organic substance	56	27.6	0.0001	192	3067	17375	1.65	0.2942	0.0230	0.1723
		Biological process	GO:0010243	response to organonitrogen compound	27	13.3	0.0001	192	1067	17375	2.29	0.3422	0.0230	0.2071
		Biological process	GO:0009719	response to endogenous stimulus	38	18.7	0.0002	192	1849	17375	1.86	0.5670	0.0317	0.4134
		Biological process	GO:0009725	response to hormone	26	12.8	0.0003	192	1069	17375	2.20	0.6586	0.0364	0.5304
		Biological process	GO:1901700	response to oxygen-containing compound	37	18.2	0.0006	192	1872	17375	1.79	0.8888	0.0684	1.0810
		Biological process	GO:0070887	cellular response to chemical stimulus	44	21.7	0.0035	192	2604	17375	1.53	1.0000	0.1755	6.2876
		Biological process	GO:1901701	cellular response to oxygen-containing compound	23	11.3	0.0042	192	1088	17375	1.91	1.0000	0.1911	7.4722
		Biological process	GO:0033993	response to lipid	24	11.8	0.0065	192	1199	17375	1.81	1.0000	0.2424	11.2634
		Biological process	GO:1901699	cellular response to nitrogen compound	15	7.4	0.0131	192	652	17375	2.08	1.0000	0.3482	21.6152
		Biological process	GO:0032870	cellular response to hormone stimulus	15	7.4	0.0164	192	671	17375	2.02	1.0000	0.3926	26.3514
		Biological process	GO:1901652	response to peptide	13	6.4	0.0199	192	555	17375	2.12	1.0000	0.4265	31.0210
		Biological process	GO:0071407	cellular response to organic cyclic compound	14	6.9	0.0224	192	631	17375	2.01	1.0000	0.4482	34.1446
		Biological process	GO:0043434	response to peptide hormone	12	5.9	0.0229	192	501	17375	2.17	1.0000	0.4537	34.8057
		Biological process	GO:0071417	cellular response to organonitrogen compound	13	6.4	0.0236	192	569	17375	2.07	1.0000	0.4571	35.6786
		Biological process	GO:0071310	cellular response to organic substance	33	16.3	0.0437	192	2143	17375	1.39	1.0000	0.5798	56.1198
		Biological process	GO:0048545	response to steroid hormone	11	5.4	0.0637	192	523	17375	1.90	1.0000	0.6542	70.2889
		Biological process	GO:0071495	cellular response to endogenous stimulus	20	9.9	0.1097	192	1277	17375	1.42	1.0000	0.7550	88.2772
		Biological process	GO:0071375	cellular response to peptide hormone stimulus	6	3.0	0.2641	192	314	17375	1.73	1.0000	0.8876	99.6505
		Biological process	GO:1901653	cellular response to peptide	6	3.0	0.3205	192	341	17375	1.59	1.0000	0.9133	99.9197
Cluster 10	2.34	Molecular function	GO:0004298	threonine-type endopeptidase activity	4	2.0	0.0025	188	24	16438	14.57	0.8263	0.0978	3.6524
		Molecular function	GO:0070003	threonine-type peptidase activity	4	2.0	0.0025	188	24	16438	14.57	0.8263	0.0978	3.6524
		Cellular component	GO:0005839	proteasome core complex	4	2.0	0.0039	190	23	13535	12.39	0.8100	0.1292	5.3078
		Cellular component	GO:0000502	proteasome complex	5	2.5	0.0181	190	72	13535	4.95	0.9996	0.4075	22.7312
Cluster 11	2.34	Biological process	GO:0044712	single-organism catabolic process	22	10.8	0.0000	192	701	17375	2.84	0.1087	0.0143	0.0569
		Biological process	GO:0019752	carboxylic acid metabolic process	22	10.8	0.0002	192	787	17375	2.53	0.4472	0.0254	0.2929
		Biological process	GO:0043436	oxoacid metabolic process	22	10.8	0.0002	192	791	17375	2.52	0.4703	0.0261	0.3140
		Biological process	GO:0006082	organic acid metabolic process	23	11.3	0.0003	192	875	17375	2.38	0.6138	0.0346	0.4696
		Biological process	GO:0016054	organic acid catabolic process	9	4.4	0.0013	192	192	17375	4.24	0.9923	0.1094	2.3785
		Biological process	GO:0046395	carboxylic acid catabolic process	8	3.9	0.0027	192	169	17375	4.28	1.0000	0.1586	4.8359
		Biological process	GO:0044282	small molecule catabolic process	10	4.9	0.0030	192	270	17375	3.35	1.0000	0.1699	5.4664
		Biological process	GO:0032787	monocarboxylic acid metabolic process	12	5.9	0.0346	192	535	17375	2.03	1.0000	0.5405	47.8080
		Biological process	GO:0006631	fatty acid metabolic process	9	4.4	0.0423	192	354	17375	2.30	1.0000	0.5732	54.8986
		Biological process	GO:0044255	cellular lipid metabolic process	14	6.9	0.1880	192	896	17375	1.41	1.0000	0.8327	97.8508
		Biological process	GO:0006629	lipid metabolic process	17	8.4	0.2205	192	1174	17375	1.31	1.0000	0.8578	98.9894
		Biological process	GO:0008610	lipid biosynthetic process	6	3.0	0.6836	192	522	17375	1.04	1.0000	0.9933	100.0000
Cluster 12	2.27	Biological process	GO:0001525	angiogenesis	13	6.4	0.0016	192	401	17375	2.93	0.9979	0.1201	2.9944
		Biological process	GO:0048514	blood vessel morphogenesis	14	6.9	0.0032	192	492	17375	2.58	1.0000	0.1687	5.6848
		Biological process	GO:0001944	vasculature development	16	7.9	0.0044	192	633	17375	2.29	1.0000	0.1951	7.7370
		Biological process	GO:0072358	cardiovascular system development	22	10.8	0.0047	192	1030	17375	1.93	1.0000	0.2010	8.2950

		Biological process	GO:0072359	circulatory system development	22	10.8	0.0047	192	1030	17375	1.93	1.0000	0.2010	8.2950
		Biological process	GO:0001568	blood vessel development	15	7.4	0.0061	192	594	17375	2.29	1.0000	0.2325	10.6510
		Biological process	GO:0048646	anatomical structure formation involved in morphogenesis	21	10.3	0.0394	192	1194	17375	1.59	1.0000	0.5593	52.3870
Cluster 13	2.26	Molecular function	GO:0043167	ion binding	61	30.0	0.0018	188	3722	16438	1.43	0.7228	0.0876	2.6902
		Molecular function	GO:0046872	metal ion binding	55	27.1	0.0086	188	3501	16438	1.37	0.9978	0.2167	12.1722
		Molecular function	GO:0043169	cation binding	55	27.1	0.0110	188	3545	16438	1.36	0.9996	0.2444	15.3596
Cluster 14	2.21	Molecular function	GO:0030170	pyridoxal phosphate binding	6	3.0	0.0005	188	58	16438	9.05	0.3031	0.0355	0.7647
		Molecular function	GO:0016212	kynurenine-oxoglutarate transaminase activity	3	1.5	0.0008	188	4	16438	65.58	0.4179	0.0480	1.1436
		Molecular function	GO:0036137	kynurenine aminotransferase activity	3	1.5	0.0008	188	4	16438	65.58	0.4179	0.0480	1.1436
		Biological process	GO:0006103	2-oxoglutarate metabolic process	4	2.0	0.0008	192	17	17375	21.29	0.9481	0.0858	1.4537
		Biological process	GO:0097052	L-kynurenine metabolic process	3	1.5	0.0012	192	5	17375	54.30	0.9876	0.1039	2.1470
		Molecular function	GO:0048037	cofactor binding	11	5.4	0.0028	188	308	16438	3.12	0.8659	0.0956	4.1799
		Biological process	GO:0070189	kynurenine metabolic process	3	1.5	0.0051	192	10	17375	27.15	1.0000	0.2145	9.0062
		Biological process	GO:1901605	alpha-amino acid metabolic process	8	3.9	0.0057	192	194	17375	3.73	1.0000	0.2243	10.0171
		Biological process	GO:0006520	cellular amino acid metabolic process	8	3.9	0.0099	192	215	17375	3.37	1.0000	0.2988	16.6972
		Molecular function	GO:0008483	transaminase activity	3	1.5	0.0302	188	24	16438	10.93	1.0000	0.4119	37.0400
		Molecular function	GO:0016769	transferase activity, transferring nitrogenous groups	3	1.5	0.0350	188	26	16438	10.09	1.0000	0.4376	41.6184
		Biological process	GO:0042537	benzene-containing compound metabolic process	3	1.5	0.0454	192	31	17375	8.76	1.0000	0.5865	57.5353
		Biological process	GO:0043648	dicarboxylic acid metabolic process	4	2.0	0.0809	192	92	17375	3.93	1.0000	0.7043	78.8909
		Biological process	GO:0042180	cellular ketone metabolic process	5	2.5	0.1370	192	180	17375	2.51	1.0000	0.7798	93.3989
Cluster 15	2.08	Biological process	GO:0048871	multicellular organismal homeostasis	11	5.4	0.0038	192	332	17375	3.00	1.0000	0.1784	6.7611
		Biological process	GO:0001894	tissue homeostasis	8	3.9	0.0110	192	220	17375	3.29	1.0000	0.3184	18.5225
		Biological process	GO:0060249	anatomical structure homeostasis	10	4.9	0.0138	192	343	17375	2.64	1.0000	0.3586	22.6737

Table S18: D-III Female_Effect of FGR (only Poor)

Rank	Score	Category	Term ID	Term name	Count	%	PValue	List Total	Pop Hits	Pop Total	Fold Enrichment	Bonferroni	Benjamini	FDR
Cluster 1	18.89	Cellular component	GO:0070062	extracellular exosome	90	51.1	0.0000	166	2646	13535	2.77	0.0000	0.0000	0.0000
		Cellular component	GO:1903561	extracellular vesicle	90	51.1	0.0000	166	2661	13535	2.76	0.0000	0.0000	0.0000
		Cellular component	GO:0043230	extracellular organelle	90	51.1	0.0000	166	2666	13535	2.75	0.0000	0.0000	0.0000
		Cellular component	GO:0031988	membrane-bounded vesicle	96	54.5	0.0000	166	3321	13535	2.36	0.0000	0.0000	0.0000
		Cellular component	GO:0044421	extracellular region part	95	54.0	0.0000	166	3710	13535	2.09	0.0000	0.0000	0.0000
		Cellular component	GO:0005576	extracellular region	96	54.5	0.0000	166	4113	13535	1.90	0.0000	0.0000	0.0000
Cluster 2	5.00	Biological process	GO:0006082	organic acid metabolic process	30	17.0	0.0000	167	875	17375	3.57	0.0000	0.0000	0.0000
		Biological process	GO:0019752	carboxylic acid metabolic process	26	14.8	0.0000	167	787	17375	3.44	0.0004	0.0002	0.0002
		Biological process	GO:0043436	oxoacid metabolic process	26	14.8	0.0000	167	791	17375	3.42	0.0004	0.0001	0.0002
		Biological process	GO:0032787	monocarboxylic acid metabolic process	21	11.9	0.0000	167	535	17375	4.08	0.0006	0.0002	0.0004
		Biological process	GO:0006631	fatty acid metabolic process	16	9.1	0.0000	167	354	17375	4.70	0.0050	0.0007	0.0029
		Biological process	GO:0006629	lipid metabolic process	22	12.5	0.0041	167	1174	17375	1.95	1.0000	0.1596	7.0950
		Biological process	GO:0044255	cellular lipid metabolic process	18	10.2	0.0054	167	896	17375	2.09	1.0000	0.1857	9.3973
		Biological process	GO:0008610	lipid biosynthetic process	8	4.5	0.2329	167	522	17375	1.59	1.0000	0.8556	99.1800
Cluster 3	3.25	Biological process	GO:0019752	carboxylic acid metabolic process	26	14.8	0.0000	167	787	17375	3.44	0.0004	0.0002	0.0002
		Biological process	GO:0043436	oxoacid metabolic process	26	14.8	0.0000	167	791	17375	3.42	0.0004	0.0001	0.0002
		Biological process	GO:0044283	small molecule biosynthetic process	14	8.0	0.0004	167	453	17375	3.22	0.7357	0.0373	0.7582
		Biological process	GO:0046394	carboxylic acid biosynthetic process	10	5.7	0.0005	167	242	17375	4.30	0.8197	0.0430	0.9752
		Biological process	GO:0016053	organic acid biosynthetic process	10	5.7	0.0009	167	258	17375	4.03	0.9331	0.0571	1.5348
		Biological process	GO:0072330	monocarboxylic acid biosynthetic process	8	4.5	0.0012	167	170	17375	4.90	0.9808	0.0732	2.2353
		Biological process	GO:0006633	fatty acid biosynthetic process	6	3.4	0.0090	167	133	17375	4.69	1.0000	0.2477	15.1637
		Biological process	GO:0008610	lipid biosynthetic process	8	4.5	0.2329	167	522	17375	1.59	1.0000	0.8556	99.1800
		Biological process	GO:0006066	alcohol metabolic process	3	1.7	0.6777	167	243	17375	1.28	1.0000	0.9919	100.0000
Cluster 4	2.55	Biological process	GO:0006732	coenzyme metabolic process	16	9.1	0.0000	167	307	17375	5.42	0.0008	0.0002	0.0005
		Biological process	GO:0051186	cofactor metabolic process	17	9.7	0.0000	167	373	17375	4.74	0.0019	0.0003	0.0011
		Biological process	GO:0055086	nucleobase-containing small molecule metabolic process	21	11.9	0.0000	167	672	17375	3.25	0.0210	0.0018	0.0121
		Biological process	GO:0009117	nucleotide metabolic process	19	10.8	0.0000	167	613	17375	3.22	0.0727	0.0058	0.0432
		Biological process	GO:0006753	nucleoside phosphate metabolic process	19	10.8	0.0000	167	620	17375	3.19	0.0840	0.0062	0.0501
		Biological process	GO:0072524	pyridine-containing compound metabolic process	9	5.1	0.0000	167	135	17375	6.94	0.1368	0.0098	0.0841
		Biological process	GO:0046128	purine ribonucleoside metabolic process	13	7.4	0.0001	167	314	17375	4.31	0.1466	0.0099	0.0907
		Biological process	GO:0042278	purine nucleoside metabolic process	13	7.4	0.0001	167	318	17375	4.25	0.1640	0.0099	0.1024
		Biological process	GO:0072521	purine-containing compound metabolic process	17	9.7	0.0001	167	540	17375	3.28	0.1788	0.0103	0.1126
		Biological process	GO:0006733	oxidoreduction coenzyme metabolic process	9	5.1	0.0001	167	145	17375	6.46	0.2163	0.0121	0.1393
		Biological process	GO:0009119	ribonucleoside metabolic process	13	7.4	0.0001	167	338	17375	4.00	0.2739	0.0151	0.1829
		Biological process	GO:0009116	nucleoside metabolic process	13	7.4	0.0002	167	357	17375	3.79	0.4135	0.0240	0.3048
		Biological process	GO:0009150	purine ribonucleotide metabolic process	15	8.5	0.0002	167	476	17375	3.28	0.4624	0.0266	0.3544
		Biological process	GO:0009167	purine ribonucleoside monophosphate metabolic process	11	6.3	0.0002	167	261	17375	4.38	0.4858	0.0273	0.3797
		Biological process	GO:0009126	purine nucleoside monophosphate metabolic process	11	6.3	0.0002	167	262	17375	4.37	0.4968	0.0271	0.3920
		Biological process	GO:0009165	nucleotide biosynthetic process	12	6.8	0.0002	167	315	17375	3.96	0.5138	0.0274	0.4116
		Biological process	GO:1901293	nucleoside phosphate biosynthetic process	12	6.8	0.0002	167	317	17375	3.94	0.5330	0.0278	0.4346
		Biological process	GO:0019362	pyridine nucleotide metabolic process	8	4.5	0.0002	167	129	17375	6.45	0.5339	0.0269	0.4357
		Biological process	GO:0046496	nicotinamide nucleotide metabolic process	8	4.5	0.0002	167	129	17375	6.45	0.5339	0.0269	0.4357
		Biological process	GO:0009259	ribonucleotide metabolic process	15	8.5	0.0003	167	490	17375	3.18	0.5660	0.0284	0.4763
		Biological process	GO:0009161	ribonucleoside monophosphate metabolic process	11	6.3	0.0003	167	271	17375	4.22	0.5931	0.0295	0.5130
		Biological process	GO:1901657	glycosyl compound metabolic process	13	7.4	0.0003	167	380	17375	3.56	0.6116	0.0300	0.5395
		Biological process	GO:0006163	purine nucleotide metabolic process	15	8.5	0.0003	167	499	17375	3.13	0.6334	0.0309	0.5724
		Biological process	GO:0019693	ribose phosphate metabolic process	15	8.5	0.0003	167	501	17375	3.12	0.6482	0.0312	0.5958
		Biological process	GO:0006091	generation of precursor metabolites and energy	12	6.8	0.0003	167	330	17375	3.78	0.6580	0.0311	0.6118
		Biological process	GO:0009123	nucleoside monophosphate metabolic process	11	6.3	0.0004	167	285	17375	4.02	0.7381	0.0365	0.7634
		Biological process	GO:0072525	pyridine-containing compound biosynthetic process	4	2.3	0.0007	167	19	17375	21.90	0.9046	0.0520	1.3349
		Biological process	GO:0072522	purine-containing compound biosynthetic process	10	5.7	0.0009	167	259	17375	4.02	0.9388	0.0565	1.5850
		Biological process	GO:0009127	purine nucleoside monophosphate biosynthetic process	6	3.4	0.0009	167	78	17375	8.00	0.9426	0.0566	1.6209
		Biological process	GO:0009168	purine ribonucleoside monophosphate biosynthetic process	6	3.4	0.0009	167	78	17375	8.00	0.9426	0.0566	1.6209
		Biological process	GO:0009156	ribonucleoside monophosphate biosynthetic process	6	3.4	0.0016	167	88	17375	7.09	0.9927	0.0840	2.7713
		Biological process	GO:1901566	organonitrogen compound biosynthetic process	26	14.8	0.0020	167	1412	17375	1.92	0.9980	0.0997	3.4830
		Biological process	GO:0019637	organophosphate metabolic process	20	11.4	0.0021	167	961	17375	2.17	0.9987	0.1053	3.7461
		Biological process	GO:0009124	nucleoside monophosphate biosynthetic process	6	3.4	0.0024	167	97	17375	6.44	0.9995	0.1113	4.2266
		Biological process	GO:0009152	purine ribonucleotide biosynthetic process	9	5.1	0.0025	167	245	17375	3.82	0.9996	0.1144	4.4178
		Biological process	GO:0006164	purine nucleotide biosynthetic process	9	5.1	0.0032	167	255	17375	3.67	1.0000	0.1378	5.6052
		Biological process	GO:0051188	cofactor biosynthetic process	7	4.0	0.0032	167	150	17375	4.86	1.0000	0.1366	5.6307
		Biological process	GO:0009260	ribonucleotide biosynthetic process	9	5.1	0.0033	167	257	17375	3.64	1.0000	0.1387	5.8828
		Biological process	GO:0046390	ribose phosphate biosynthetic process	9	5.1	0.0036	167	260	17375	3.60	1.0000	0.1457	6.2776
		Biological process	GO:0009108	coenzyme biosynthetic process	6	3.4	0.0041	167	110	17375	5.68	1.0000	0.1591	7.1660
		Biological process	GO:0019674	NAD metabolic process	4	2.3	0.0065	167	40	17375	10.40	1.0000	0.2100	11.1908
		Biological process	GO:1901135	carbohydrate derivative metabolic process	20	11.4	0.0068	167	1072	17375	1.94	1.0000	0.2127	11.7035
		Biological process	GO:0090407	organophosphate biosynthetic process	12	6.8	0.0075	167	490	17375	2.55	1.0000	0.2227	12.7964
		Biological process	GO:0009135	purine nucleoside diphosphate metabolic process	5	2.8	0.0109	167	90	17375	5.78	1.0000	0.2742	17.9599
		Biological process	GO:0009179	purine ribonucleoside diphosphate metabolic process	5	2.8	0.0109	167	90	17375	5.78	1.0000	0.2742	17.9599
		Biological process	GO:0009185	ribonucleoside diphosphate metabolic process	5	2.8	0.0121	167	93	17375	5.59	1.0000	0.2945	19.8643
		Biological process	GO:0019363	pyridine nucleotide biosynthetic process	3	1.7	0.0126	167	18	17375	17.34	1.0000	0.2982	20.4584
		Biological process	GO:0019359	nicotinamide nucleotide biosynthetic process	3	1.7	0.0126	167	18	17375	17.34	1.0000	0.2982	20.4584
		Biological process	GO:0046129	purine ribonucleoside biosynthetic process	5	2.8	0.0126	167	94	17375	5.53	1.0000	0.2947	20.5198
		Biological process	GO:0042451	purine nucleoside biosynthetic process	5	2.8	0.0126	167	94	17375	5.53	1.0000	0.2947	20.5198
		Molecular function	GO:0000287	magnesium ion binding	7	4.0	0.0168	160	211	16438	3.41	1.0000	0.3319	22.2488
		Biological process	GO:0046034	ATP metabolic process	7	4.0	0.0204	167	223	17375	3.27	1.0000	0.3942	31.1147
		Biological process	GO:0044724	single-organism carbohydrate catabolic process	5	2.8	0.0219	167	111	17375	4.69	1.0000	0.4069	32.9994
		Biological process	GO:0042455	ribonucleoside biosynthetic process	5	2.8	0.0232	167	113	17375	4.60	1.0000	0.4138	34.5986
		Biological process	GO:0009163	nucleoside biosynthetic process	5	2.8	0.0232	167	113	17375	4.60	1.0000	0.4138	34.5986
		Biological process	GO:0009132	nucleoside diphosphate metabolic process	5	2.8	0.0245	167	115	17375	4.52	1.0000	0.4229	36.2166
		Biological process	GO:1901659	glycosyl compound biosynthetic process	5	2.8	0.0245	167	115	17375	4.52	1.0000	0.4229	36.2166
		Biological process	GO:0009205	purine ribonucleoside triphosphate metabolic process	7	4.0	0.0289	167	242	17375	3.01	1.0000	0.4509	41.2250
		Biological process	GO:0006096	glycolytic process	4	2.3	0.0305	167	71	17375	5.86	1.0000	0.4668	42.9472
		Biological process	GO:0006757	ATP generation from ADP	4	2.3	0.0316	167	72	17375	5.78	1.0000	0.4663	44.1181
		Biological process	GO:0009199	ribonucleoside triphosphate metabolic process	7	4.0	0.0320	167	248	17375	2.94	1.0000	0.4679	44.4704
		Biological process	GO:0009144	purine nucleoside triphosphate metabolic process	7	4.0	0.0331	167	250	17375	2.91	1.0000	0.4740	45.6670
		Biological process	GO:0016052	carbohydrate catabolic process	5	2.8	0.0353	167	129	17375	4.03	1.0000	0.4839	47.8378
		Biological process	GO:0046031	ADP metabolic process	4	2.3	0.0412	167	80	17375	5.20	1.0000	0.5118	53.3655
		Biological process	GO:0009141	nucleoside triphosphate metabolic process	7	4.0	0.0460	167	271	17375	2.69	1.0000	0.5345	57.3802
		Biological process	GO:0006165	nucleoside diphosphate phosphorylation	4	2.3	0.0551	167	90	17375	4.62	1.0000	0.5626	64.1735

		Biological process	GO:0046939	nucleotide phosphorylation	4	2.3	0.0659	167	97	17375	4.29	1.0000	0.5845	70.9372
		Biological process	GO:0006090	pyruvate metabolic process	4	2.3	0.0709	167	100	17375	4.16	1.0000	0.5958	73.5930
		Biological process	GO:0006754	ATP biosynthetic process	3	1.7	0.0825	167	50	17375	6.24	1.0000	0.6239	78.9992
		Biological process	GO:0044723	single-organism carbohydrate metabolic process	10	5.7	0.1163	167	594	17375	1.75	1.0000	0.7105	89.3592
		Biological process	GO:0009206	purine ribonucleoside triphosphate biosynthetic process	3	1.7	0.1185	167	62	17375	5.03	1.0000	0.7130	89.8206
		Biological process	GO:0009145	purine nucleoside triphosphate biosynthetic process	3	1.7	0.1216	167	63	17375	4.95	1.0000	0.7197	90.4600
		Biological process	GO:0009201	ribonucleoside triphosphate biosynthetic process	3	1.7	0.1377	167	68	17375	4.59	1.0000	0.7443	93.1693
		Biological process	GO:0005975	carbohydrate metabolic process	11	6.3	0.1383	167	705	17375	1.62	1.0000	0.7449	93.2531
		Biological process	GO:1901137	carbohydrate derivative biosynthetic process	10	5.7	0.1621	167	642	17375	1.62	1.0000	0.7834	95.9354
		Biological process	GO:0009142	nucleoside triphosphate biosynthetic process	3	1.7	0.1778	167	80	17375	3.90	1.0000	0.8036	97.1194
		Biological process	GO:0019318	hexose metabolic process	4	2.3	0.3291	167	212	17375	1.96	1.0000	0.9154	99.9277
		Biological process	GO:0005996	monosaccharide metabolic process	4	2.3	0.3562	167	222	17375	1.87	1.0000	0.9277	99.9657
Cluster 5	2.54	Molecular function	GO:0042802	identical protein binding	26	14.8	0.0009	160	1325	16438	2.02	0.4395	0.1093	1.3794
		Molecular function	GO:0042803	protein homodimerization activity	18	10.2	0.0021	160	805	16438	2.30	0.7198	0.1662	3.0066
		Molecular function	GO:0046983	protein dimerization activity	22	12.5	0.0121	160	1283	16438	1.76	0.9995	0.2905	16.5680
Cluster 6	2.33	Cellular component	GO:0044429	mitochondrial part	23	13.1	0.0004	166	820	13535	2.29	0.1592	0.0191	0.5635
		Cellular component	GO:0005739	mitochondrion	37	21.0	0.0013	166	1777	13535	1.70	0.4342	0.0463	1.8383
		Cellular component	GO:0005740	mitochondrial envelope	18	10.2	0.0017	166	629	13535	2.33	0.5302	0.0565	2.4313
		Cellular component	GO:0031966	mitochondrial membrane	16	9.1	0.0052	166	584	13535	2.23	0.8948	0.1176	7.0750
		Cellular component	GO:0031967	organelle envelope	22	12.5	0.0117	166	1014	13535	1.77	0.9938	0.1983	15.2660
		Cellular component	GO:0005743	mitochondrial inner membrane	12	6.8	0.0122	166	412	13535	2.37	0.9951	0.1915	15.8998
		Cellular component	GO:0031975	envelope	22	12.5	0.0127	166	1022	13535	1.76	0.9960	0.1914	16.4685
		Cellular component	GO:0019866	organelle inner membrane	12	6.8	0.0257	166	461	13535	2.12	1.0000	0.2627	30.7437
Cluster 7	2.20	Biological process	GO:0044712	single-organism catabolic process	22	12.5	0.0000	167	701	17375	3.27	0.0110	0.0011	0.0063
		Biological process	GO:0016042	lipid catabolic process	10	5.7	0.0013	167	275	17375	3.78	0.9854	0.0753	2.3890
		Biological process	GO:0019395	fatty acid oxidation	6	3.4	0.0024	167	97	17375	6.44	0.9995	0.1113	4.2266
		Biological process	GO:0034440	lipid oxidation	6	3.4	0.0026	167	99	17375	6.31	0.9997	0.1159	4.6112
		Biological process	GO:0006635	fatty acid beta-oxidation	5	2.8	0.0043	167	69	17375	7.54	1.0000	0.1620	7.4905
		Biological process	GO:0009062	fatty acid catabolic process	5	2.8	0.0089	167	85	17375	6.12	1.0000	0.2495	15.0025
		Biological process	GO:0016054	organic acid catabolic process	7	4.0	0.0104	167	192	17375	3.79	1.0000	0.2752	17.2754
		Biological process	GO:0030258	lipid modification	7	4.0	0.0114	167	196	17375	3.72	1.0000	0.2821	18.8243
		Biological process	GO:0072329	monocarboxylic acid catabolic process	5	2.8	0.0135	167	96	17375	5.42	1.0000	0.3061	21.8606
		Biological process	GO:0044282	small molecule catabolic process	8	4.5	0.0151	167	270	17375	3.08	1.0000	0.3311	24.1238
		Biological process	GO:0046395	carboxylic acid catabolic process	6	3.4	0.0232	167	169	17375	3.69	1.0000	0.4121	34.6502
		Biological process	GO:0044242	cellular lipid catabolic process	6	3.4	0.0312	167	183	17375	3.41	1.0000	0.4682	43.6965
		Cellular component	GO:0005777	peroxisome	5	2.8	0.0938	166	141	13535	2.89	1.0000	0.5030	75.0882
		Cellular component	GO:0042579	microbody	5	2.8	0.0938	166	141	13535	2.89	1.0000	0.5030	75.0882
Cluster 8	2.17	Biological process	GO:0001666	response to hypoxia	12	6.8	0.0014	167	393	17375	3.18	0.9890	0.0787	2.5463
		Biological process	GO:0036293	response to decreased oxygen levels	12	6.8	0.0018	167	404	17375	3.09	0.9963	0.0937	3.1570
		Biological process	GO:0070482	response to oxygen levels	12	6.8	0.0033	167	438	17375	2.85	1.0000	0.1388	5.8098
		Biological process	GO:0009628	response to abiotic stimulus	22	12.5	0.0112	167	1288	17375	1.78	1.0000	0.2801	18.5273
		Biological process	GO:0071456	cellular response to hypoxia	6	3.4	0.0154	167	152	17375	4.11	1.0000	0.3329	24.4391
		Biological process	GO:0036294	cellular response to decreased oxygen levels	6	3.4	0.0187	167	160	17375	3.90	1.0000	0.3715	29.0127
		Biological process	GO:0071453	cellular response to oxygen levels	6	3.4	0.0248	167	172	17375	3.63	1.0000	0.4223	36.5621

Table S19: C-I Male_Effect of Nutrition (common)

Rank	Score	Category	Term ID	Term name	Count	%	PValue	List Total	Pop Hits	Pop Total	Fold Enrichment	Bonferroni	Benjamini	FDR
Cluster 1	4.06	Cellular component	GO:0031988	membrane-bounded vesicle	32	47.8	0.0000	61	3321	13535	2.14	0.0017	0.0017	0.0071
		Cellular component	GO:0070062	extracellular exosome	27	40.3	0.0000	61	2646	13535	2.26	0.0069	0.0034	0.0292
		Cellular component	GO:1903561	extracellular vesicle	27	40.3	0.0000	61	2661	13535	2.25	0.0076	0.0025	0.0324
		Cellular component	GO:0043230	extracellular organelle	27	40.3	0.0000	61	2666	13535	2.25	0.0079	0.0020	0.0335
		Cellular component	GO:0044421	extracellular region part	29	43.3	0.0011	61	3710	13535	1.73	0.2928	0.0378	1.4583
		Cellular component	GO:0005576	extracellular region	29	43.3	0.0057	61	4113	13535	1.56	0.8381	0.0962	7.4297
Cluster 2	2.39	Cellular component	GO:0099513	polymeric cytoskeletal fiber	11	16.4	0.0002	61	581	13535	4.20	0.0655	0.0135	0.2870
		Cellular component	GO:0099512	supramolecular fiber	11	16.4	0.0002	61	581	13535	4.20	0.0655	0.0135	0.2870
		Cellular component	GO:0030286	dynein complex	4	6.0	0.0009	61	43	13535	20.64	0.2492	0.0352	1.2078
		Molecular function	GO:0003774	motor activity	5	7.5	0.0010	60	125	16438	10.96	0.2695	0.1453	1.3932
		Molecular function	GO:0016462	pyrophosphatase activity	10	14.9	0.0013	60	749	16438	3.66	0.3245	0.1226	1.7373
		Molecular function	GO:0016818	hydrolase activity, acting on acid anhydrides, in phosphorus-containing anhydrides	10	14.9	0.0013	60	752	16438	3.64	0.3320	0.0959	1.7859
		Molecular function	GO:0016817	hydrolase activity, acting on acid anhydrides	10	14.9	0.0014	60	758	16438	3.61	0.3471	0.0817	1.8864
		Cellular component	GO:0005868	cytoplasmic dynein complex	3	4.5	0.0028	61	18	13535	36.98	0.5932	0.0622	3.7418
		Cellular component	GO:0005875	microtubule associated complex	5	7.5	0.0031	61	136	13535	8.16	0.6247	0.0633	4.0702
		Cellular component	GO:0005874	microtubule	7	10.4	0.0034	61	332	13535	4.68	0.6638	0.0659	4.5160
		Molecular function	GO:0017111	nucleoside-triphosphatase activity	9	13.4	0.0035	60	703	16438	3.51	0.6459	0.1378	4.5312
		Cellular component	GO:0015630	microtubule cytoskeleton	12	17.9	0.0059	61	1051	13535	2.53	0.8481	0.0944	7.6796
		Cellular component	GO:0005815	microtubule organizing center	8	11.9	0.0187	61	616	13535	2.88	0.9975	0.2209	22.4317
		Cellular component	GO:0005813	centrosome	7	10.4	0.0227	61	499	13535	3.11	0.9993	0.2534	26.6379
		Biological process	GO:0007017	microtubule-based process	7	10.4	0.0243	63	629	17375	3.07	1.0000	0.6134	34.4099
		Biological process	GO:0007018	microtubule-based movement	3	4.5	0.1813	63	217	17375	3.81	1.0000	0.8478	96.7474
		Biological process	GO:0000226	microtubule cytoskeleton organization	4	6.0	0.2119	63	445	17375	2.48	1.0000	0.8655	98.3046
Cluster 3	2.278	Cellular component	GO:0005912	adherens junction	11	16.4	0.0005	61	643	13535	3.80	0.1412	0.0250	0.6430
		Cellular component	GO:0070161	anchoring junction	11	16.4	0.0006	61	658	13535	3.71	0.1666	0.0257	0.7699
		Cellular component	GO:0005925	focal adhesion	8	11.9	0.0018	61	396	13535	4.48	0.4302	0.0547	2.3565
		Cellular component	GO:0005924	cell-substrate adherens junction	8	11.9	0.0019	61	401	13535	4.43	0.4534	0.0534	2.5286
		Cellular component	GO:0030055	cell-substrate junction	8	11.9	0.0020	61	406	13535	4.37	0.4770	0.0526	2.7102
		Cellular component	GO:0030054	cell junction	14	20.9	0.0038	61	1293	13535	2.40	0.6997	0.0683	4.9725
		Biological process	GO:0007155	cell adhesion	11	16.4	0.0340	63	1468	17375	2.07	1.0000	0.6479	44.7268
		Biological process	GO:0022610	biological adhesion	11	16.4	0.0354	63	1478	17375	2.05	1.0000	0.6516	46.0755
		Cellular component	GO:0009897	external side of plasma membrane	3	4.5	0.3662	61	288	13535	2.31	1.0000	0.8962	99.7860

Table S20: E-I Female_Effect of Nutrition (common)														
Rank	Score	Category	Term ID	Term name	Count	%	PValue	List Total	Pop Hits	Pop Total	Fold Enrichment	Bonferroni	Benjamini	FDR
none	none	none	none	none	none	none	none	none	none	none	none	none	none	none

Table S21: C-II Male_Effect of Nutrition (only FGR)

Rank	Score	Category	Term ID	Term name	Count	%	PValue	List Total	Pop Hits	Pop Total	Fold Enrichment	Bonferroni	Benjamini	FDR
Cluster 1	9.42	Cellular component	GO:0070062	extracellular exosome	76	40.6	0.0000	179	2646	13535	2.17	0.0000	0.0000	0.0000
		Cellular component	GO:1903561	extracellular vesicle	76	40.6	0.0000	179	2661	13535	2.16	0.0000	0.0000	0.0000
		Cellular component	GO:0043230	extracellular organelle	76	40.6	0.0000	179	2666	13535	2.16	0.0000	0.0000	0.0000
		Cellular component	GO:0031988	membrane-bounded vesicle	80	42.8	0.0000	179	3321	13535	1.82	0.0000	0.0000	0.0000
		Cellular component	GO:0044421	extracellular region part	85	45.5	0.0000	179	3710	13535	1.73	0.0000	0.0000	0.0000
		Cellular component	GO:0005576	extracellular region	86	46.0	0.0000	179	4113	13535	1.58	0.0003	0.0001	0.0011
Cluster 2	3.12	Biological process	GO:0006082	organic acid metabolic process	28	15.0	0.0000	177	875	17375	3.14	0.0007	0.0007	0.0004
		Biological process	GO:0019752	carboxylic acid metabolic process	26	13.9	0.0000	177	787	17375	3.24	0.0013	0.0006	0.0007
		Biological process	GO:0043436	oxoacid metabolic process	26	13.9	0.0000	177	791	17375	3.23	0.0014	0.0005	0.0008
		Biological process	GO:0044712	single-organism catabolic process	22	11.8	0.0000	177	701	17375	3.08	0.0290	0.0049	0.0161
		Biological process	GO:0016054	organic acid catabolic process	11	5.9	0.0000	177	192	17375	5.62	0.0849	0.0126	0.0485
		Biological process	GO:0046395	carboxylic acid catabolic process	10	5.3	0.0001	177	169	17375	5.81	0.1731	0.0235	0.1039
		Biological process	GO:0044282	small molecule catabolic process	12	6.4	0.0001	177	270	17375	4.36	0.2805	0.0295	0.1798
		Biological process	GO:0032787	monocarboxylic acid metabolic process	16	8.6	0.0004	177	535	17375	2.94	0.7055	0.0593	0.6661
		Biological process	GO:0019395	fatty acid oxidation	6	3.2	0.0031	177	97	17375	6.07	1.0000	0.2665	5.4381
		Biological process	GO:0034440	lipid oxidation	6	3.2	0.0033	177	99	17375	5.95	1.0000	0.2800	5.9242
		Biological process	GO:0006635	fatty acid beta-oxidation	5	2.7	0.0053	177	69	17375	7.11	1.0000	0.3239	9.1812
		Biological process	GO:0006631	fatty acid metabolic process	10	5.3	0.0102	177	354	17375	2.77	1.0000	0.3689	16.9897
		Biological process	GO:0009062	fatty acid catabolic process	5	2.7	0.0109	177	85	17375	5.77	1.0000	0.3740	18.1058
		Biological process	GO:0072329	monocarboxylic acid catabolic process	5	2.7	0.0164	177	96	17375	5.11	1.0000	0.4059	26.0521
		Biological process	GO:0044242	cellular lipid catabolic process	6	3.2	0.0387	177	183	17375	3.22	1.0000	0.5451	51.2882
		Biological process	GO:0030258	lipid modification	6	3.2	0.0494	177	196	17375	3.01	1.0000	0.5833	60.2994
		Biological process	GO:0006629	lipid metabolic process	18	9.6	0.0870	177	1174	17375	1.51	1.0000	0.6814	80.9316
		Biological process	GO:0044255	cellular lipid metabolic process	14	7.5	0.1229	177	896	17375	1.53	1.0000	0.7524	90.8266
		Biological process	GO:0016042	lipid catabolic process	6	3.2	0.1463	177	275	17375	2.14	1.0000	0.7897	94.3965
Cluster 3	3.10	Molecular function	GO:0045296	cadherin binding	12	6.4	0.0001	180	243	16438	4.51	0.0498	0.0498	0.1107
		Cellular component	GO:0005912	adherens junction	22	11.8	0.0001	179	643	13535	2.59	0.0433	0.0055	0.1533
		Cellular component	GO:0030054	cell junction	34	18.2	0.0001	179	1293	13535	1.99	0.0586	0.0067	0.2090
		Cellular component	GO:0070161	anchoring junction	22	11.8	0.0002	179	658	13535	2.53	0.0592	0.0061	0.2112
		Molecular function	GO:0098641	cadherin binding involved in cell-cell adhesion	11	5.9	0.0002	180	220	16438	4.57	0.0999	0.0513	0.2280
		Molecular function	GO:0098632	protein binding involved in cell-cell adhesion	11	5.9	0.0002	180	227	16438	4.43	0.1268	0.0442	0.2936
		Molecular function	GO:0098631	protein binding involved in cell adhesion	11	5.9	0.0002	180	232	16438	4.33	0.1496	0.0397	0.3506
		Molecular function	GO:0050839	cell adhesion molecule binding	14	7.5	0.0004	180	393	16438	3.25	0.2319	0.0430	0.5703
		Cellular component	GO:0005925	focal adhesion	15	8.0	0.0008	179	396	13535	2.86	0.2613	0.0272	1.0437
		Cellular component	GO:0005924	cell-substrate adherens junction	15	8.0	0.0008	179	401	13535	2.83	0.2897	0.0281	1.1781
		Cellular component	GO:0030055	cell-substrate junction	15	8.0	0.0010	179	406	13535	2.79	0.3198	0.0272	1.3266
		Biological process	GO:0007155	cell adhesion	27	14.4	0.0036	177	1468	17375	1.81	1.0000	0.2771	6.3543
		Biological process	GO:0022610	biological adhesion	27	14.4	0.0039	177	1478	17375	1.79	1.0000	0.2742	6.9333
		Cellular component	GO:0005913	cell-cell adherens junction	10	5.3	0.0093	179	269	13535	2.81	0.9766	0.1712	12.2040
		Biological process	GO:0098609	cell-cell adhesion	20	10.7	0.0112	177	1060	17375	1.85	1.0000	0.3734	18.4929
		Cellular component	GO:0005911	cell-cell junction	14	7.5	0.0586	179	609	13535	1.74	1.0000	0.5438	56.9536
Cluster 4	2.64	Molecular function	GO:0043167	ion binding	61	32.6	0.0005	180	3722	16438	1.50	0.3121	0.0520	0.8078
		Molecular function	GO:0043169	cation binding	55	29.4	0.0042	180	3545	16438	1.42	0.9481	0.2185	6.2125
		Molecular function	GO:0046872	metal ion binding	54	28.9	0.0053	180	3501	16438	1.41	0.9757	0.2196	7.7446
Cluster 5	2.52	Biological process	GO:0034622	cellular macromolecular complex assembly	23	12.3	0.0001	177	906	17375	2.49	0.3459	0.0279	0.2318
		Biological process	GO:0065003	macromolecular complex assembly	31	16.6	0.0005	177	1563	17375	1.95	0.7953	0.0666	0.8636
		Biological process	GO:0006461	protein complex assembly	25	13.4	0.0022	177	1268	17375	1.94	0.9994	0.2256	3.9716
		Biological process	GO:0070271	protein complex biogenesis	25	13.4	0.0022	177	1269	17375	1.93	0.9994	0.2209	4.0120
		Biological process	GO:0071822	protein complex subunit organization	26	13.9	0.0041	177	1407	17375	1.81	1.0000	0.2802	7.2715
		Biological process	GO:0022607	cellular component assembly	38	20.3	0.0079	177	2456	17375	1.52	1.0000	0.3459	13.4004
		Biological process	GO:0043933	macromolecular complex subunit organization	35	18.7	0.0082	177	2213	17375	1.55	1.0000	0.3452	13.9698
		Biological process	GO:0043623	cellular protein complex assembly	12	6.4	0.0138	177	504	17375	2.34	1.0000	0.3895	22.4024
		Biological process	GO:0044085	cellular component biogenesis	39	20.9	0.0206	177	2705	17375	1.42	1.0000	0.4331	31.5195
Cluster 6	2.03	Molecular function	GO:0042803	protein homodimerization activity	18	9.6	0.0069	180	805	16438	2.04	0.9919	0.2469	9.9235
		Molecular function	GO:0042802	identical protein binding	25	13.4	0.0094	180	1325	16438	1.72	0.9986	0.2921	13.2623
		Molecular function	GO:0046983	protein dimerization activity	24	12.8	0.0123	180	1283	16438	1.71	0.9998	0.3495	17.0070
Cluster 7	2.00	Biological process	GO:0001889	liver development	8	4.3	0.0076	177	222	17375	3.54	1.0000	0.3542	12.9491
		Biological process	GO:0061008	hepaticobiliary system development	8	4.3	0.0081	177	225	17375	3.49	1.0000	0.3502	13.7974
		Biological process	GO:0048732	gland development	13	7.0	0.0160	177	584	17375	2.19	1.0000	0.4091	25.4260

Table S22: E-II Female_Effect of Nutrition (only FGR)														
Rank	Score	Category	Term ID	Term name	Count	%	PValue	List Total	Pop Hits	Pop Total	Fold Enrichment	Bonferroni	Benjamini	FDR
Cluster 1	3.37	Cellular component	GO:0070062	extracellular exosome	36	35.6	0.0000	95	2646	13535	1.94	0.0142	0.0071	0.0640
		Cellular component	GO:1903561	extracellular vesicle	36	35.6	0.0001	95	2661	13535	1.93	0.0160	0.0054	0.0723
		Cellular component	GO:0043230	extracellular organelle	36	35.6	0.0001	95	2666	13535	1.92	0.0166	0.0042	0.0753
		Cellular component	GO:0031988	membrane-bounded vesicle	40	39.6	0.0002	95	3321	13535	1.72	0.0610	0.0104	0.2824
		Cellular component	GO:0044421	extracellular region part	38	37.6	0.0078	95	3710	13535	1.46	0.9015	0.2515	9.8876
		Cellular component	GO:0005576	extracellular region	39	38.6	0.0242	95	4113	13535	1.35	0.9993	0.2923	27.8343
Cluster 2	3.20	Molecular function	GO:0016462	pyrophosphatase activity	17	16.8	0.0000	91	749	16438	4.10	0.0010	0.0010	0.0037
		Molecular function	GO:0016818	hydrolase activity, acting on acid anhydrides, in phosphorus-containing anhydrides	17	16.8	0.0000	91	752	16438	4.08	0.0010	0.0005	0.0039
		Molecular function	GO:0016817	hydrolase activity, acting on acid anhydrides	17	16.8	0.0000	91	758	16438	4.05	0.0012	0.0004	0.0043
		Molecular function	GO:0017111	nucleoside-triphosphatase activity	16	15.8	0.0000	91	703	16438	4.11	0.0022	0.0005	0.0080
		Molecular function	GO:0035639	purine ribonucleoside triphosphate binding	23	22.8	0.0001	91	1753	16438	2.37	0.0535	0.0109	0.2050
		Molecular function	GO:0097367	carbohydrate derivative binding	26	25.7	0.0002	91	2152	16438	2.18	0.0577	0.0099	0.2217
		Molecular function	GO:0032550	purine ribonucleoside binding	23	22.8	0.0002	91	1763	16438	2.36	0.0579	0.0085	0.2227
		Molecular function	GO:0032549	ribonucleoside binding	23	22.8	0.0002	91	1766	16438	2.35	0.0593	0.0068	0.2283
		Molecular function	GO:0001883	purine nucleoside binding	23	22.8	0.0002	91	1766	16438	2.35	0.0593	0.0068	0.2283
		Molecular function	GO:0001882	nucleoside binding	23	22.8	0.0002	91	1773	16438	2.34	0.0627	0.0065	0.2417
		Molecular function	GO:0008135	translation factor activity, RNA binding	6	5.9	0.0002	91	96	16438	11.29	0.0648	0.0061	0.2501
		Molecular function	GO:1901265	nucleoside phosphate binding	27	26.7	0.0002	91	2307	16438	2.11	0.0668	0.0057	0.2578
		Molecular function	GO:0000166	nucleotide binding	27	26.7	0.0002	91	2307	16438	2.11	0.0668	0.0057	0.2578
		Molecular function	GO:0032555	purine ribonucleotide binding	23	22.8	0.0002	91	1798	16438	2.31	0.0762	0.0061	0.2956
		Molecular function	GO:0017076	purine nucleotide binding	23	22.8	0.0002	91	1809	16438	2.30	0.0829	0.0062	0.3226
		Molecular function	GO:0032553	ribonucleotide binding	23	22.8	0.0002	91	1815	16438	2.29	0.0867	0.0060	0.3382
		Molecular function	GO:0036094	small molecule binding	28	27.7	0.0003	91	2506	16438	2.02	0.1000	0.0066	0.3927
		Molecular function	GO:0003746	translation elongation factor activity	4	4.0	0.0003	91	24	16438	30.11	0.1033	0.0064	0.4066
		Molecular function	GO:0003924	GTPase activity	7	6.9	0.0008	91	198	16438	6.39	0.2451	0.0147	1.0447
		Molecular function	GO:0005525	GTP binding	8	7.9	0.0048	91	380	16438	3.80	0.8320	0.0746	6.4456
		Molecular function	GO:0032561	guanyl ribonucleotide binding	8	7.9	0.0065	91	402	16438	3.59	0.9103	0.0920	8.6143
		Molecular function	GO:0019001	guanyl nucleotide binding	8	7.9	0.0066	91	403	16438	3.59	0.9131	0.0897	8.7235
		Molecular function	GO:0042623	ATPase activity, coupled	7	6.9	0.0077	91	315	16438	4.01	0.9412	0.0931	10.0462
		Molecular function	GO:0005524	ATP binding	15	14.9	0.0200	91	1403	16438	1.93	0.9994	0.2074	24.2569
		Molecular function	GO:0032559	adenyl ribonucleotide binding	15	14.9	0.0244	91	1440	16438	1.88	0.9999	0.2350	28.8346
		Molecular function	GO:0030554	adenyl nucleotide binding	15	14.9	0.0256	91	1449	16438	1.87	0.9999	0.2389	30.0116
		Molecular function	GO:0016887	ATPase activity	7	6.9	0.0315	91	433	16438	2.92	1.0000	0.2733	35.6728
		Molecular function	GO:1901363	heterocyclic compound binding	40	39.6	0.0472	91	5645	16438	1.28	1.0000	0.3598	48.6462
		Biological process	GO:0006414	translational elongation	3	3.0	0.0573	97	70	17375	7.68	1.0000	0.7540	64.4954
		Molecular function	GO:0097159	organic cyclic compound binding	40	39.6	0.0579	91	5726	16438	1.26	1.0000	0.4156	56.0817
Cluster 3	2.83	Cellular component	GO:0099512	supramolecular fiber	16	15.8	0.0000	95	581	13535	3.92	0.0031	0.0031	0.0138
		Cellular component	GO:0099513	polymeric cytoskeletal fiber	16	15.8	0.0000	95	581	13535	3.92	0.0031	0.0031	0.0138
		Cellular component	GO:0005874	microtubule	11	10.9	0.0001	95	332	13535	4.72	0.0294	0.0059	0.1338
		Cellular component	GO:0015630	microtubule cytoskeleton	15	14.9	0.0134	95	1051	13535	2.03	0.9819	0.3595	16.4824
		Cellular component	GO:0005815	microtubule organizing center	7	6.9	0.2564	95	616	13535	1.62	1.0000	0.7922	98.0799
		Cellular component	GO:0005813	centrosome	6	5.9	0.2658	95	499	13535	1.71	1.0000	0.8000	98.3783
Cluster 4	2.64	Biological process	GO:0009057	macromolecule catabolic process	18	17.8	0.0000	97	1006	17375	3.20	0.0760	0.0760	0.0578
		Biological process	GO:0044265	cellular macromolecule catabolic process	16	15.8	0.0000	97	825	17375	3.47	0.1013	0.0520	0.0780
		Biological process	GO:0030163	protein catabolic process	15	14.9	0.0001	97	770	17375	3.49	0.1788	0.0636	0.1439
		Biological process	GO:0044257	cellular protein catabolic process	13	12.9	0.0002	97	649	17375	3.59	0.4333	0.1324	0.4143
		Biological process	GO:0051603	proteolysis involved in cellular protein catabolic process	12	11.9	0.0006	97	619	17375	3.47	0.7680	0.2534	1.0626
		Biological process	GO:0019941	modification-dependent protein catabolic process	10	9.9	0.0023	97	520	17375	3.44	0.9962	0.5022	3.9972
		Biological process	GO:0043632	modification-dependent macromolecule catabolic process	10	9.9	0.0026	97	528	17375	3.39	0.9979	0.4969	4.4191
		Biological process	GO:0044248	cellular catabolic process	17	16.8	0.0038	97	1392	17375	2.19	0.9999	0.5691	6.5468
		Molecular function	GO:0044389	ubiquitin-like protein ligase binding	7	6.9	0.0058	91	297	16438	4.26	0.8828	0.0855	7.6964
		Biological process	GO:0006511	ubiquitin-dependent protein catabolic process	9	8.9	0.0074	97	512	17375	3.15	1.0000	0.6725	12.2408
		Biological process	GO:0006508	proteolysis	17	16.8	0.0194	97	1661	17375	1.83	1.0000	0.7100	29.1021
		Molecular function	GO:0031625	ubiquitin protein ligase binding	6	5.9	0.0224	91	293	16438	3.70	0.9998	0.2236	26.7955
		Biological process	GO:0016567	protein ubiquitination	8	7.9	0.0769	97	669	17375	2.14	1.0000	0.7619	75.4889
		Molecular function	GO:0004842	ubiquitin-protein transferase activity	5	5.0	0.1763	91	400	16438	2.26	1.0000	0.7474	93.0944

Table S23: C-III Male_Effect of Nutrition (only Sham)

Rank	Score	Category	Term ID	Term name	Count	%	PValue	List Total	Pop Hits	Pop Total	Fold Enrichment	Bonferroni	Benjamini	FDR
Cluster 1	5.98	Cellular component	GO:0043230	extracellular organelle	44	40.0	0.0000	97	2666	13535	2.30	0.0000	0.0000	0.0000
		Cellular component	GO:1903561	extracellular vesicle	43	39.1	0.0000	97	2661	13535	2.25	0.0000	0.0000	0.0001
		Cellular component	GO:0070062	extracellular exosome	41	37.3	0.0000	97	2646	13535	2.16	0.0002	0.0001	0.0007
		Cellular component	GO:0031988	membrane-bounded vesicle	45	40.9	0.0000	97	3321	13535	1.89	0.0015	0.0004	0.0058
		Cellular component	GO:0044421	extracellular region part	48	43.6	0.0000	97	3710	13535	1.81	0.0020	0.0004	0.0075
		Cellular component	GO:0005576	extracellular region	48	43.6	0.0001	97	4113	13535	1.63	0.0363	0.0061	0.1378
Cluster 2	2.33	Biological process	GO:1901607	alpha-amino acid biosynthetic process	6	5.5	0.0001	101	73	17375	14.14	0.1527	0.1527	0.1116
		Biological process	GO:0006082	organic acid metabolic process	15	13.6	0.0005	101	875	17375	2.95	0.7140	0.4652	0.8398
		Biological process	GO:1901605	alpha-amino acid metabolic process	7	6.4	0.0009	101	194	17375	6.21	0.9057	0.5448	1.5782
		Biological process	GO:0044283	small molecule biosynthetic process	10	9.1	0.0012	101	453	17375	3.80	0.9579	0.5471	2.1120
		Biological process	GO:0043436	oxoacid metabolic process	13	11.8	0.0019	101	791	17375	2.83	0.9940	0.4000	3.3826
		Biological process	GO:0009086	methionine biosynthetic process	3	2.7	0.0037	101	16	17375	32.26	0.9999	0.4215	6.4227
		Biological process	GO:0006555	methionine metabolic process	3	2.7	0.0047	101	18	17375	28.67	1.0000	0.4054	8.0613
		Biological process	GO:0008652	cellular amino acid biosynthetic process	4	3.6	0.0048	101	59	17375	11.66	1.0000	0.3952	8.1217
		Biological process	GO:0019752	carboxylic acid metabolic process	12	10.9	0.0055	101	787	17375	2.62	1.0000	0.4187	9.3969
		Biological process	GO:0006520	cellular amino acid metabolic process	6	5.5	0.0081	101	215	17375	4.80	1.0000	0.4471	13.3855
		Biological process	GO:0009067	aspartate family amino acid biosynthetic process	3	2.7	0.0105	101	27	17375	19.11	1.0000	0.4530	17.0543
		Biological process	GO:0016053	organic acid biosynthetic process	6	5.5	0.0167	101	258	17375	4.00	1.0000	0.5601	25.8273
		Biological process	GO:0009066	aspartate family amino acid metabolic process	3	2.7	0.0312	101	48	17375	10.75	1.0000	0.6614	42.9787
		Biological process	GO:0046394	carboxylic acid biosynthetic process	5	4.5	0.0511	101	242	17375	3.55	1.0000	0.7667	60.6047
		Molecular function	GO:0016810	hydrolase activity, acting on carbon-nitrogen (but not peptide) bonds	3	2.7	0.1786	101	126	16438	3.88	1.0000	0.8070	93.8309

Table S24: E-III Female_Effect of Nutrition (only Sham)

Rank	Score	Category	Term ID	Term name	Count	%	PValue	List Total	Pop Hits	Pop Total	Fold Enrichment	Bonferroni	Benjamini	FDR
Cluster 1	3.59	Cellular component	GO:0070062	extracellular exosome	31	38.3	0.0000	76	2646	13535	2.09	0.0105	0.0105	0.0460
		Cellular component	GO:1903561	extracellular vesicle	31	38.3	0.0000	76	2661	13535	2.07	0.0117	0.0059	0.0514
		Cellular component	GO:0043230	extracellular organelle	31	38.3	0.0000	76	2666	13535	2.07	0.0121	0.0041	0.0534
		Cellular component	GO:0031988	membrane-bounded vesicle	33	40.7	0.0004	76	3321	13535	1.77	0.1209	0.0317	0.5612
		Cellular component	GO:0044421	extracellular region part	33	40.7	0.0031	76	3710	13535	1.58	0.6138	0.1733	4.0706
		Cellular component	GO:0005576	extracellular region	35	43.2	0.0044	76	4113	13535	1.52	0.7420	0.2021	5.7456
